# Carbocycle editing of ketones proceeds via a radical-mediated bidirectional C-C bond cleavage/coupling strategy

**DOI:** 10.1038/s41467-026-74317-0

**Published:** 2026-06-15

**Authors:** Ying-Jie Ma, Yuan Gao, Xin Chen, Le Liu, Xin-Hua Duan, Li-Na Guo

**Affiliations:** 1 School of Chemistry, Xi’an Key Laboratory of Sustainable Energy Material Chemistry, Engineering Research Center of Energy Storage Materials and Devices, Ministry of Education, Xi’an Jiaotong University, Xi’an, China; 2 State Key Laboratory of Natural Product Chemistry, Lanzhou University, Lanzhou, China

**Keywords:** Synthetic chemistry methodology, Synthetic chemistry methodology, Catalytic mechanisms

## Abstract

1,n-Difunctionalized alkyl linchpins are pivotal building blocks in organic synthesis, functional materials, and pharmaceuticals. However, their preparation currently relies predominantly on de novo synthesis, which is hampered by low step-economy and poor functional group compatibility. Herein, we report an efficient method based on a radical-mediated bidirectional C-C bond cleavage, which enables simple cyclic ketones to function as potential diradical linchpins via the formation of *gem*-diperoxides. It employs a stepwise and controllable tandem process involving alkoxy radical-induced *β*-scission and acyloxy radical-mediated decarboxylation, to achieve ring-opening, carbon shrinkage, and bidirectional functionalization of carbocycles. This carbocycle editing protocol features sustainable metal catalysis, a broad substrate scope, and excellent functional group compatibility. Notably, the cascade reaction enables facile and selective editing of various complex natural products and drug molecules. This bidirectional C-C bond cleavage/coupling strategy allows for rapid and modular synthesis of structurally diverse alkyl 1,n-dithiocyanides, 1,n-diazides, 1,n-dihalides, and unsymmetric 1,n-thiocyanate-azides (*n* ≥ 4).

## Abstract (web summary)

1,n-Difunctionalized alkyl linchpins are valuable synthetic intermediates, but current routes suffer from poor step economy and limited functional group tolerance. Here, the authors report a radical-mediated bidirectional C–C bond cleavage strategy that transforms cyclic ketones into diverse functionalized linchpins via *gem*-diperoxide intermediates.

## Introduction

**Fig. 1:**
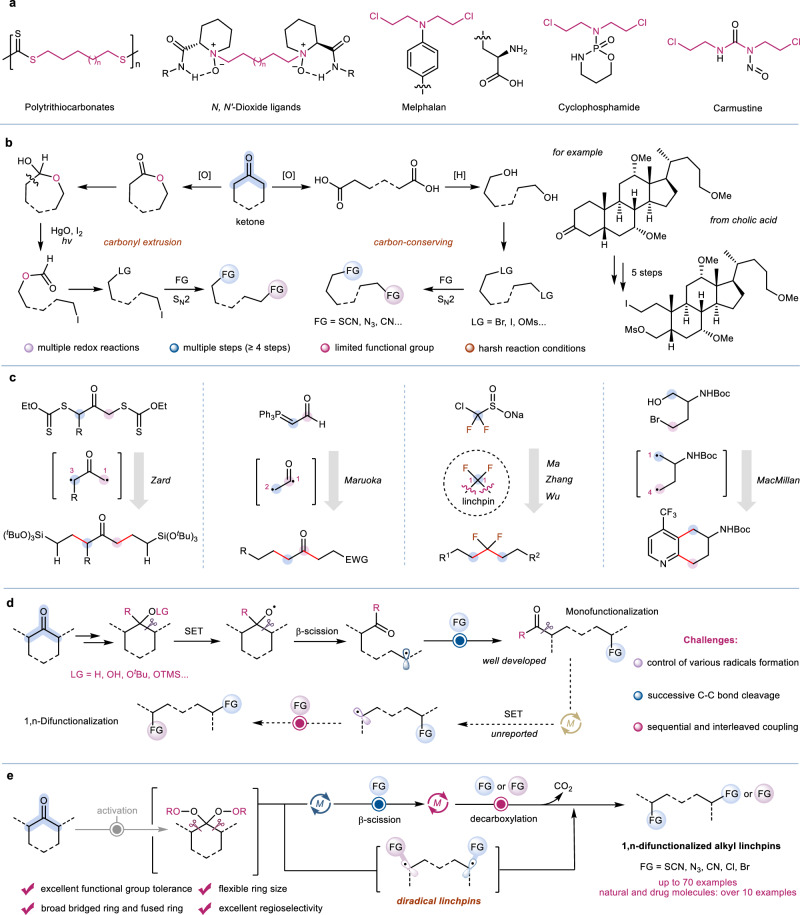
Carbocycle editing of ketones via radical-mediated bidirectional C-C bond cleavage/coupling. **a** Importance of 1,n-difunctionalized alkyl compounds. **b** Traditional synthetic methods for linchpins and limitations. **c** Known examples of bidirectional radical linchpins. **d** Alkoxy radical-mediated C-C cleavage for functionalized alkyl compounds. **e** This work: carbocycle editing of ketones via a radical-mediated bidirectional C-C bond cleavage/coupling strategy.

1,n-Difunctionalized alkyl linchpins constitute an important class of compounds with high utility across various fields, including organic synthesis^1,2^, functional materials^3,4^, and pharmaceutical molecules^5–8^ (Fig. 1a). However, their preparation currently relies predominantly on de novo synthesis, which is hampered by low step-economy and poor functional group compatibility. For instance, ketones are abundant chemicals and are widely found in natural products. Therefore, starting from readily available cyclic ketones to prepare 1,n-difunctionalized alkyl linchpins is highly attractive. However, this strategy typically requires multiple-step transformations, such as oxidation, reduction, substitution, and so on (Fig. 1b)^9–12^. Some of these protocols are plagued by harsh reaction conditions, competitive side reactions, limited functional group tolerance, and expensive reagents, all of which severely restrict their utility in organic synthesis. In recent years, some elegant bidirectional functionalization reactions using bifunctional reagents such as silyl dithianes, vinyl boronic esters, and lithiated *gem*-diborylalkanes as linchpins have been successfully developed^13–15^. However, these protocols rely on organometallic reagents, which still face challenges such as harsh reaction conditions and limited functional group compatibility.

Thanks to the vigorous development of radical chemistry, the radical-mediated bidirectional linchpin coupling has emerged as an attractive complementary strategy (Fig. 1c). For example, Zard’s group^16^ has disclosed a thermally promoted radical bidirectional fragment coupling route to unsymmetrical Si-containing ketones, employing bis-xanthate as the linchpin. Maruoka’s group^17^ has reported a photocatalyzed one-pot, two-step radical bidirectional elongation strategy to access 1,4-dicarbonyls, using formyl-stabilized ylide as an ambiphilic radical linchpin. The group of Ma, Zhang and Wu^18^ has successfully demonstrated a photocatalyzed synthesis of difluorinated hydrocarbons from alkenes, using ClCF_2_SO_2_Na as the linchpin. MacMillan’s group^19^ has reported a radical-mediated cyclization using bromoalcohols or diols as diradical precursors under photocatalysis. Despite these advances, the radical-mediated bidirectional linchpin coupling strategy has primarily focused on C-C bond formation, while C-heteroatom bond formation remains unexplored. Moreover, distal bidirectional linchpin coupling (*n* > 4) remains undeveloped and is highly challenging. Consequently, the development of new formal diradical linchpins and catalytic cascades to access distal 1,n-difunctionalized alkyl linchpins is both urgently needed and challenging.

In recent years, radical-mediated cyclic C-C bond cleavage has emerged as a powerful tool for the synthesis of various functionalized alkyl compounds. In this field, significant progress has been made in aromatization-driven C-C bond cleavage^20,21^ and in the alkoxy radical-mediated deconstruction and functionalization of cycloalkanols and related cyclic ketone derivatives^22–36^. In these reactions, aminyl radical cations or alkoxy radicals generated through a single-electron transfer (SET) process initiate *β*-scission, yielding reactive alkyl radicals and stable functional groups (ketones and esters). Although this mechanistic pathway has demonstrated significant potential for diverse functionalizations, it has thus far only enabled monodirectional functionalization (Fig. 1d). We wonder whether the stable functional group formed in situ can be further activated to generate new reactive alkyl radicals through a second C-C bond cleavage. If successful, this would achieve bidirectional functionalization, thereby providing a modular synthesis for diverse 1,n-difunctionalized alkyl linchpins. The challenges include precisely controlling the formation of various radicals, orchestrating successive C-C bond cleavage, and implementing a sequential and interleaved functionalization^37,38^. Building on our previous studies on C-C cleavage^39–42^, we aim to develop cyclic ketones as potential diradical linchpins through radical-mediated bidirectional fragmentation under abundant metal catalysis. Although C-C bond activation of ketones has received considerable attention over the past two decades^43^, *gem*-diperoxides—which are readily accessible from ketones—have remained underexplored as efficient candidates for such transformations.

In this work, we show that alkoxy-induced *β*-scission and acyloxy radical-triggered decarboxylation are successfully integrated under iron and copper catalysis by carefully modulating the stability of intermediates and the compatibility between the two catalytic cycles (Fig. 1e). The integration enables the ring-opening, carbon chain shortening, and bidirectional 1,n-difunctionalization of aliphatic ring skeletons, thereby expanding the scope of molecular transformations. Notably, this strategy displays exceptional compatibility across a wide range of ring sizes, functional groups, ring systems (monocyclic, bridged, and fused rings), and complex natural products with multiple carbonyl groups. This radical-mediated bidirectional C-C bond cleavage strategy provides a rapid and modular approach to synthesizing a variety of alkyl 1,n-dithiocyanides, 1,n-diazides, and 1,n-dihalides (*n* ≥ 4). Remarkably, a one-pot, two-step protocol has also been explored for the synthesis of unsymmetric azido-thiocyanato linchpins.

## Results and discussion

**Fig. 2:**
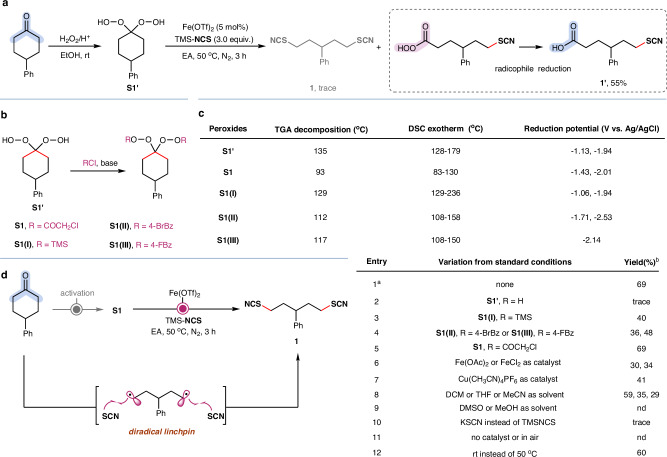
Activation of cyclic ketones and optimization of bidirectional C-C bond cleavage/dithiocyanation. **a** Preparation of *gem*-dihydroperoxide and its thiocyanation. **b** Diverse synthesis of *gem*-diperoxides. **c** Thermal and electrochemical behavior of various *gem*-diperoxides. **d** Optimization of the reaction conditions. ^a^ Reaction conditions: **S1** (0.20 mmol, 1.0 equiv.), TMSNCS (3.0 equiv.), Fe(OTf)_2_ (5 mol%), EA (2 mL), 50 °C, N_2_, 3 h. ^b^ Isolated yield.

Driven by this concept, we initially synthesized a *gem*-dihydroperoxide **S1’** derived from 4-phenylcyclohexanone, employing H_2_O_2_ as a green and sustainable oxidant (Fig. 2a)^37,44–46^. After extensive trials, it was found that the anticipated ring-opening/dithiocyanation product **1** could not be obtained under iron catalysis. Instead, the SCN-containing carboxylic acid **1’** was isolated with a yield of 55%. This outcome was attributed to the instability of the peroxoic acid intermediate^37^. Consequently, a series of *gem*-diperoxides with different leaving groups were prepared, including trimethylsilyl ether **S1(I)** and diesters (**S1,** **S1(II),** **S1(III)**) (Fig. 2b). Simultaneous thermal analysis (DSC-TGA) revealed that all *gem*-diperoxides exhibit relatively high thermal decomposition temperatures, with **S1** displaying the lowest stability and undergoing decomposition at approximately 93 °C (Fig. 2c; see Supplementary Figs. 6–10). This finding was corroborated by a DSC exothermic peak observed within the range of 83–130 °C. These results suggest that the aforementioned *gem*-diperoxides possess a certain degree of stability at ambient temperature^24,47^. Cyclic voltammetry revealed that acyl protection led to decreased reduction potentials, aligning with the observed hindered reduction reactivity (Supplementary Figs. 1–5). Notably, **S1** exhibited the highest reduction potential among the esters, yet it remained lower than that of the *gem*-dihydroperoxide **S1’**. In contrast, the TMS-ether **S1(I)** displayed a reduction potential similar to that of the *gem*-dihydroperoxide **S1’**. Fortunately, all of these *gem*-diperoxides efficiently underwent ring-opening/dithiocyanation via bidirectional C-C bond cleavage in the presence of 5 mol% of Fe(OTf)_2_ in EA at 50 °C for 3 h (Fig. 2d), yielding the one-carbon shrinkage, chain alkyl 1,5-dithiocyanide **1** in yields of 36%–69%, with *gem*-diperoxide **S1** performing the best (entries 2–5; see Supplementary Table 1). Consequently, **S1** was chosen as the model substrate to optimize the reaction conditions. Screening of commercially available iron and copper salts showed that Fe(OAc)_2_, FeCl_2_, and Cu(CH_3_CN)_4_PF_6_ were effective catalysts, with Fe(OTf)_2_ still being the optimal choice (entries 6 and 7; see Supplementary Table 2). Solvent screening revealed that reactions in DCM, THF and CH_3_CN all afforded the product **1** in yields of 29%–59%, while DMSO and MeOH were totally ineffective solvents for this transformation (entries 8 and 9; see Supplementary Table 3). Using inorganic KSCN instead of TMSNCS resulted in only trace amounts of product **1** (entry 10), with a certain amount of starting material **S1** remaining unreacted. Control experiment revealed that the catalyst is essential for this reaction (entry 11). Finally, performing the reaction at room temperature led to a slight reduction in yield, achieving a yield of 60% (entry 12).

**Fig. 3:**
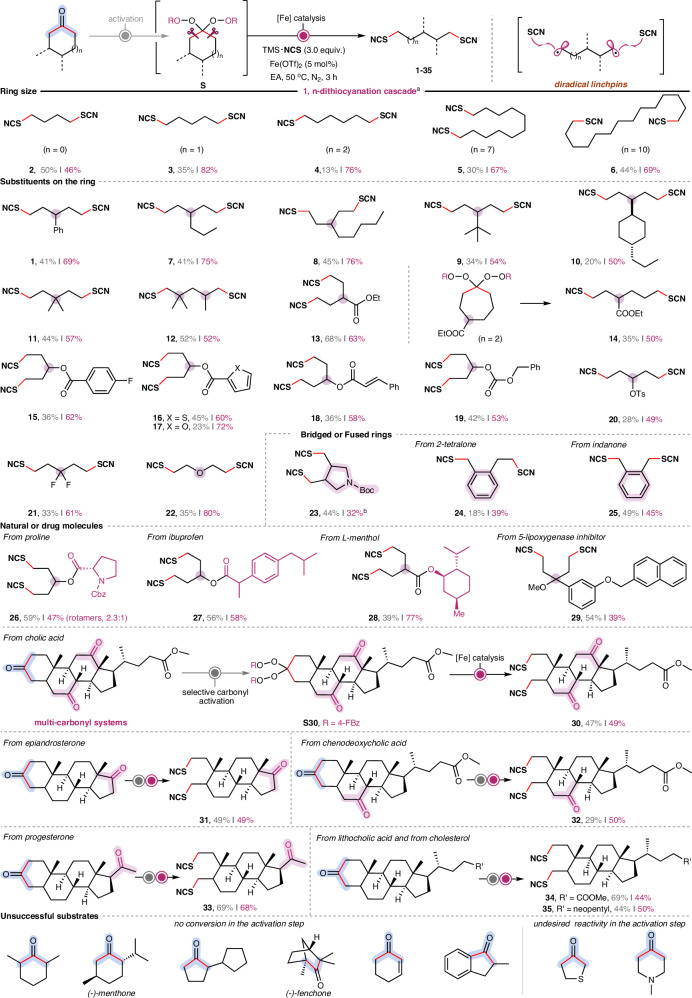
Substrate scope of 1,n-dithiocyanation. All isolated yields are provided for ketone activation (left) and difunctionalization (right). ^a^ Reaction conditions for dithiocyanation: **S** (0.20 mmol, 1.0 equiv.), TMSNCS (3.0 equiv.), Fe(OTf)_2_ (5 mol%), EA (2 mL), 50 °C, N_2_, 3 h. ^b^ dr could not be determined by ^1^H NMR.

With the optimal conditions established, the substrate scope of this ring-opening/1,n-dithiocyanation reaction was first evaluated. A variety of *gem*-diperoxides derived from cyclic ketones with different ring sizes and substituents on the aliphatic ring were efficiently engaged in this successive C-C bond cleavage and thiocyanatation, yielding the desired alkyl dithiocyanides **2**–**22** in moderate to good yields (Fig. 3). Substrates with ring sizes ranging from 5- to 7-membered rings, as well as 12- and 15-membered rings, were all effective, yielding one-carbon-shortened alkyl dithiocyanates **2**–**6** in 46%–82% yields. Substrates with various substituents on the aliphatic rings, including aryl (**1**), alkyl (**7**–**12**), esters (**13**–**14**) were all compatible with this reaction, yielding the corresponding products in good yields. Satisfactorily, substrates synthesized from esterified 4-hydroxycyclohexan-1-ones were efficiently engaged in the ring-opening/1,n-dithiocyanation reaction. A range of ester groups, including (hetero)aryl carboxylates (**15**–**17**), cinnamates (**18**), carbonates (**19**), and sulfonates (**20**), all exhibited excellent compatibility. Additionally, a 4,4-difluorocyclohexan-1-one-derived substrate was suitable, yielding product **21** in 61% yield. Beyond carbocycles, a substrate derived from tetrahydro-4*H*-pyran-4-one was also amenable, furnishing product **22** in 80% yield. Notably, this bidirectional C-C bond cleavage/coupling reaction was applicable to complex bridged (**23**) and fused ring (**24,** **25**) molecular skeletons. Substrates masked by natural products and pharmaceutical motifs—including proline (**26**), ibuprofen (**27**), menthol (**28**) and 5-lipoxygenase inhibitor (**29**)—also afforded the corresponding products in satisfactory yields. Complex steroidal ketones also reacted smoothly, yielding the desired products **30**–**35** in good yields. Remarkably, the dicarbonyl steroidal ketones, such as epiandrosterone (**31**), chenodeoxycholic acid (**32**), progesterone (**33**), and the tricarbonyl steroidal ketone cholic acid (**30**), all exhibited excellent regioselectivity in activation of cyclic ketones. This inherent regioselectivity facilitated the selective deconstruction and dithiocyanation of fused carbocycles. Typically, the reaction preferentially occurs at sites with minimal steric hindrance. For instance, under PMA-catalyzed H_2_O_2_ oxidation, compound **33** underwent selective reaction at the less hindered cyclic ketone, while the sterically encumbered acyclic ketone remained intact. A systematic evaluation of acetyl ketones revealed that *gem*-dihydroperoxide formation is highly sensitive to steric bulk, with yields progressively decreasing as *α*-substitution increases (see Supplementary Information Section 8 for details). This steric sensitivity also extends to *α*-substituted cyclic ketones (**30**–**32**); bulky substituents, such as those present in (-)-menthone, (-)-fenchone, and cyclopentyl-substituted systems, generally preclude oxidation. Furthermore, substrates containing nitrogen or sulfur heteroatoms are not compatible with this transformation, as these heteroatoms are preferentially oxidized by H_2_O_2_, thereby suppressing *gem*-dihydroperoxide formation.

**Fig. 4:**
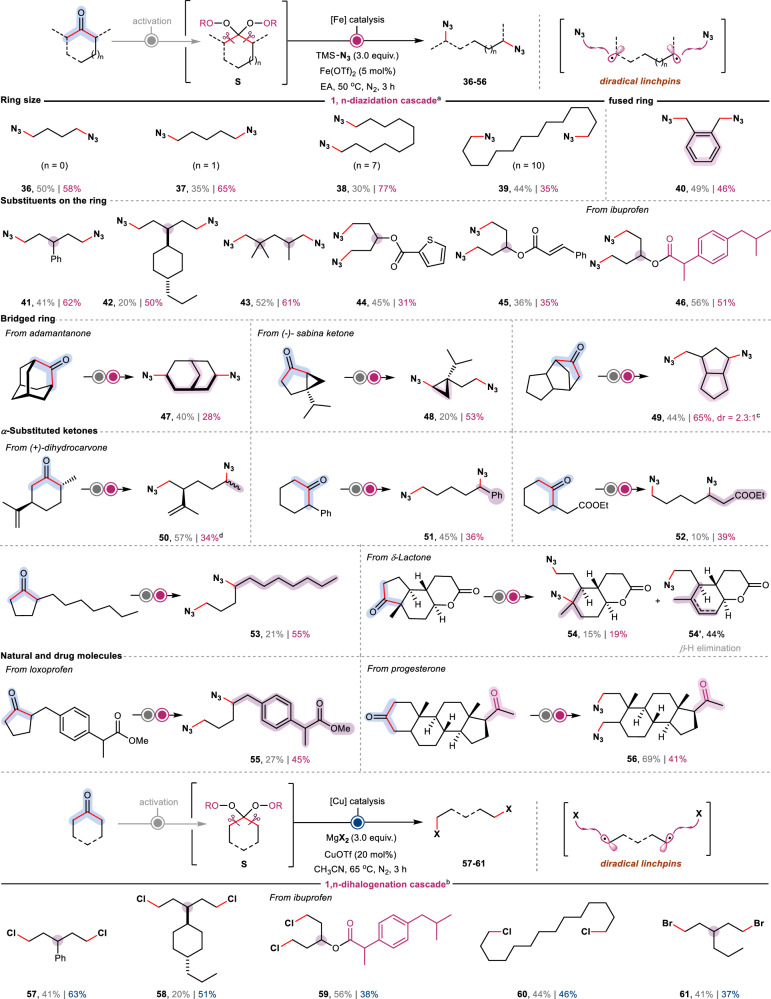
Substrate scope of 1,n-diazidation and 1,n-dihalogenation reaction. All isolated yields are provided for ketone activation (left) and difunctionalization (right). ^a^ Reaction conditions for diazidation: **S** (0.20 mmol, 1.0 equiv.), TMSN_3_ (3.0 equiv.), Fe(OTf)_2_ (5 mol%), EA (2 mL), 50 °C, N_2_, 3 h. ^b^ Reaction conditions for dihalogenation: **S** (0.20 mmol, 1.0 equiv.), MgX_2_ (3.0 equiv.), CuOTf (20 mol%), CH_3_CN (2 mL), 65 °C, N_2_, 3 h. ^c^ dr was determined by ^1^H NMR analysis. ^d^ dr could not be determined by ^1^H NMR due to peak overlapping.

Alkyl azides are valuable and versatile building blocks in organic synthesis due to their remarkable reactivity in various reactions, especially in click chemistry. Therefore, we turn to investigate the deconstructive bidirectional diazidation of cyclic ketones using TMSN_3_ as the “N_3_” source. Satisfactorily, the streamlined iron-catalyzed system proved equally effective for the diazidation reactions (Fig. 4; see Supplementary Tables 7–10). By activating ketones to *gem*-diperoxides, the reaction proceeded smoothly with TMSN_3_, affording the desired alkyl diazides **36**–**56** in satisfactory yields. Both substrates with varying ring sizes (**36**–**39**) and those bearing different substituents on the aliphatic ring (**41**–**46**) delivered the corresponding products with yields ranging from 31% to 77%. *Gem*-diperoxides derived from bridged ketones, such as adamantane (**47**), (-)-sabina ketone (**48**), and tricyclic ketone (**49**) also produced the expected alkyl diazides in reasonable yields. Additionally, *gem*-diperoxides derived from *α*-substituted cyclohexanones were also compatible with this transformation, albeit with relatively lower yields for the desired products (**50**–**53**). Notably, the *δ*-lactone-derived substrate (**54**) exhibited an unexpected outcome, where *β*-H elimination occurred, leading to the formation of an internal alkene byproduct (**54’**), presumably due to the steric hindrance of the tertiary radical intermediate. Finally, substrates derived from pharmaceutical molecules, including loxoprofen (**55**) and progesterone (**56**), successfully underwent this cascade reaction to yield the corresponding alkyl diazides in 45% and 41% yields, respectively. Furthermore, the bidirectional C-C bond cleavage/dihalogenation cascade reaction was investigated (see Supplementary Tables 11–16). We were delighted to find that **S1** was efficiently converted into the valuable 1,5-dichloride **57** in 63% yield under a copper-catalyzed system using MgCl_2_ as the “Cl” source. This outcome proceeds via alkoxy radical-mediated C-C cleavage followed by decarboxylative halogenation^48–50^, and is attributed to a favorable reductive elimination pathway involving an alkyl-Cu^III^-Cl intermediate^51^. Subsequently, several other ketones were subjected to this transformation, yielding a range of distal 1,n-dichlorides (**57**–**60**) in moderate yields. Replacing MgCl_2_ with MgBr_2_ enabled the synthesis of the 1,5-dibromoalkane **61**, albeit with a slightly low yield.

**Fig. 5:**
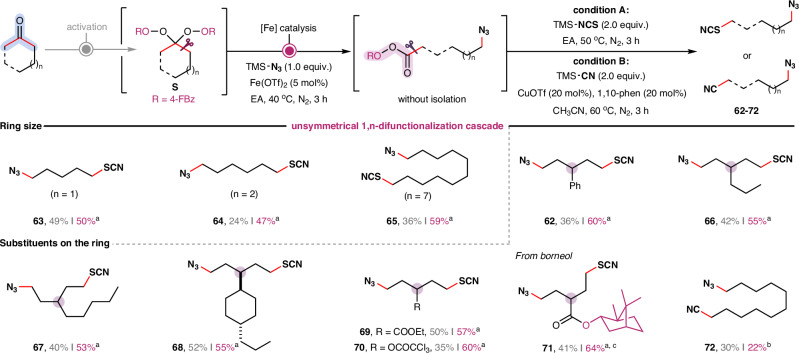
Substrate scope of unsymmetrical 1,n-difunctionalization reaction. All isolated yields are provided for ketone activation (left) and difunctionalization (right). ^a^ Condition A: **S** (0.20 mmol, 1.0 equiv.), TMSN_3_ (1.0 equiv.), Fe(OTf)_2_ (5 mol%), EA (1 mL), 40 °C, N_2_, 3 h, then TMSNCS (2.0 equiv.), EA (1 mL), 50 °C, N_2_, 3 h. ^b^ Condition B: **S** (0.20 mmol, 1.0 equiv.), TMSN_3_ (1.0 equiv.), Fe(OTf)_2_ (5 mol%), EA (1 mL), 40 °C, N_2_, 3 h, then TMSCN (2.0 equiv.), CuOTf (20 mol%), 1,10-phen (20 mol%), CH_3_CN (2 mL), 60 °C, N_2_, 3 h. ^c^ dr could not be determined by ^1^H NMR due to peak overlapping.

To further expand the scope of the bidirectional C-C bond cleavage and functionalization strategy, we explored a more challenging unsymmetric 1,n-difunctionalization cascade. After extensive trials, we successfully achieved a sequential C-C bond cleavage/azidation followed by C-C bond cleavage/thiocyanation, yielding unsymmetrical N_3_, SCN-containing alkyl compounds in moderate yields (Fig. 5). It is worth noting that the use of “4-FBz” as a protecting group for *gem*-hydroperoxide is particularly critical, as it enables the formation of a relatively stable N_3_-containing peroxide ester intermediate in the first step (see Supplementary Tables 17–19). This finding establishes a foundation for the one-pot, two-step strategy toward unsymmetrical alkyl difunctionalization molecules. A variety of 4-FBz-protected *gem*-diperoxides with different ring sizes (**63**–**65**) and substituents (**62,** **66**–**70**) successfully underwent this cascade reaction using TMSN_3_ and TMSNCS as nucleophiles, yielding the corresponding azido-thiocyanato products **62**–**70** in acceptable yields. A norbornane-masked substrate also afforded the expected product **71** in 64% yield. Moreover, a stepwise protocol involving *β*-scission/azidation followed by decarboxylative cyanation was also feasible. After the iron-catalyzed azidation, the cyanation could be conducted under a copper/1,10-phen catalyst system without the need for intermediate purification. Using TMSCN as the “cyano” source, the azido–cyano product **72** was successfully obtained, albeit with a somewhat lower yield.

**Fig. 6:**
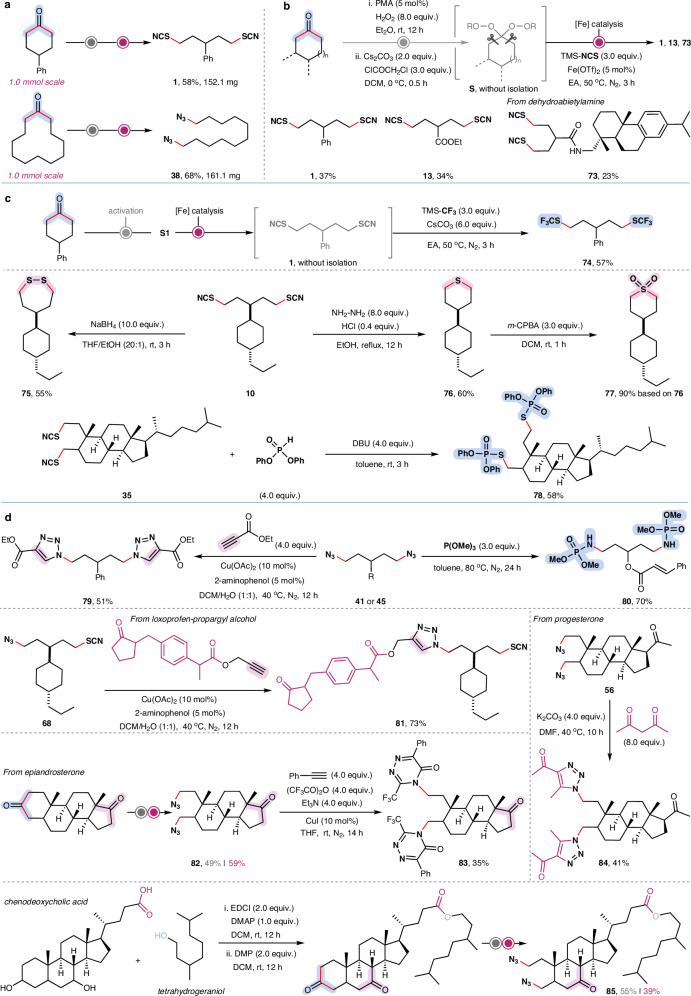
Scale-up synthesis and synthetic applications. **a** Scale-up syntheses. **b** One-pot ketones difunctionalization. **c** Derivatizations of alkyl dithiocyanides. **d** Derivatizations of alkyl azides.

To demonstrate the utility of these one-pot reactions, we carried out large-scale syntheses and explored versatile diversifications (Fig. 6). When the 1,n-dithiocyanation reaction of **S1** and 1,n-diazidation reaction of **S5** were scaled up to 1.0 mmol, satisfactory yields were still achieved (Fig. 6a). The feasibility of a one-pot transformation from cyclic ketones to the final difunctionalized alkyl products has also been demonstrated (Fig. 6b). This streamlined procedure requires only simple workup and concentration, obviating the need for column chromatography purification. The diversifications of alkyl 1,5-dithiocyanides and 1,5-diazides were then investigated (Figs. 6c and 6d). A one-pot, two-step protocol was successfully executed, involving the 1,5-dithiocyanation of **S1** followed by reacting with TMSCF_3_ under alkaline conditions, yielding the 1,5-dithiotrifluoromethyl compound **74** with a 57% yield. Additionally, treatment of 1,5-dithiocyanide **10** with an excess of NaBH_4_ resulted in the formation of seven-membered disulfide **75** in a 55% yield. Hydrazinolysis of **10** with hydrazine in ethanol afforded the cyclic thioether **76** in 60% yield. Subsequent oxidation of **76** with an excess of *m-*CPBA yielded the corresponding sulfone **77** in 90% yield. These cyclization transformations highlight the application potential of our strategy for molecular editing, offering an alternative pathway to Dong’s elegant example of carbonyl-to-sulfur swap in cyclic ketones^52^. Employing diphenyl phosphite as a nucleophile, the cholesterol-based dithiocyanate **35** was successfully transformed into phosphorodithioate **78** with a yield of 58%. Furthermore, 1,5-diazide **41** could undergo dual click reactions with ethyl propiolate under copper catalysis, yielding the bis-triazole **79** in 51% yield. Treatment of 1,5-diazide **45** with P(OMe)_3_ afforded bis-phosphoramide **80** in 70% yield. Satisfactorily, treating the N_3_- and SCN-containing product **68** with the loxoprofen-masked propargyl alcohol led to the triazole product **81** in 73% yield, retaining the “SCN” group as a potential handle for further modification. Epiandrosterone was further elaborated to the alkyl diazide **82**, which was subsequently converted to compound **83** featuring a 1,2,4-triazin-5(4*H*)-one core―a structural motif prevalent in natural products and pharmacologically active molecules^53^. Meanwhile, the progesterone-derived diazide **56** readily furnished the corresponding triazole **84** via click chemistry. In a further extension, a diketone prepared from chenodeoxycholic acid and tetrahydrogeraniol through ester condensation followed by DMP oxidation was transformed into the alkyl diazide **85**, wherein the azide groups serve as versatile handles for subsequent functionalization. All of these transformations highlight the significant potential of the bidirectional C-C bond cleavage/functionalization strategy for molecular editing and the synthesis of complex 1,n-difunctionalized alkyl molecules.

**Fig. 7:**
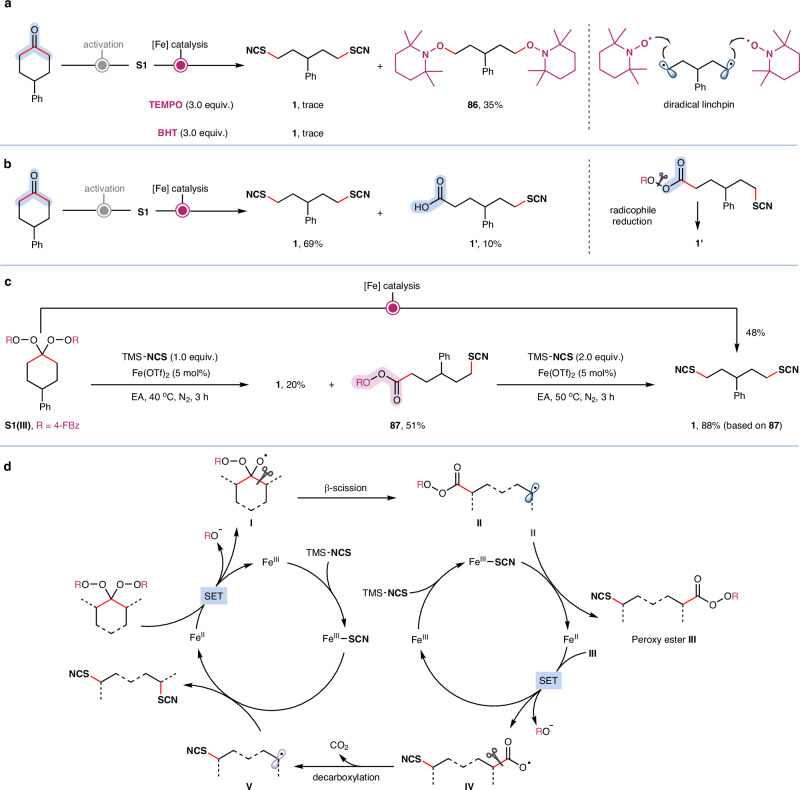
Mechanistic studies and proposed mechanisms. **a** Radical trapping and inhibiting experiments. **b** Detection of byproduct. **c** Detection of key intermediate. **d** Proposed mechanism.

To elucidate the underlying mechanism, a series of control experiments were performed (Fig. 7). When **S1** was reacted with TMSNCS in the presence of TEMPO (3.0 equiv.), only a trace amount of product **1** was detected, along with the TEMPO-adduct **86** isolated in 35% yield (Fig. 7a). These findings suggest that the reaction proceeds via a radical pathway and involves the formation of a formal diradical intermediate. Moreover, the addition of BHT (3.0 equiv.) resulted in only trace amounts of product **1**, further supporting a radical mechanism for this reaction. On the other hand, the reaction of **S1** and TMSNCS not only afforded the desired product **1** in 69% yield, but also produced the byproduct **1’** in a 10% yield (Fig. 7b). We speculate that the carboxylic acid **1’** was formed from a peroxyester intermediate, which was generated by the initial alkoxy radical-mediated *β*-scission. Fortunately, the peroxyester **87** was successfully isolated in 51% yield using compound **S1(III)** instead of **S1**. The peroxyester **87** can further transform into the target product **1** in 88% yield (based on **87**), thereby confirming that the peroxyester is indeed a key intermediate in this reaction (Fig. 7c). Based on the above results and literature, a probable mechanism is proposed for the 1,n-dithiocyanation (Fig. 7d). Initially, single-electron reduction (SER) of *gem-*diperoxide by Fe^II^ generates an alkoxy radical **I** and Fe^III^. Subsequently, the alkoxy radical **I** undergoes *β*-scission to produce an alkyl radical **II**. Meanwhile, the Fe^III^ species interacts with TMSNCS to form a Fe^III^SCN complex^54^. Next, the alkyl radical **II** reacts with Fe^III^SCN complex via an inner- or outer-sphere process^55^ to produce a peroxyester **III** and regenerate Fe^II^. The peroxyester **III** further undergoes a SER process with Fe^II^, yielding an acyloxy radical **IV** and Fe^III^. The intermediate **IV** then decarboxylates to generate a one-carbon-shortened alkyl radical **V**. Finally, the alkyl radical **V** reacts with the Fe^III^SCN complex to produce the desired product and regenerate the Fe^II^ species. This bidirectional C-C bond cleavage/dithiocyanation reaction involves two consecutive catalytic cycles.

In conclusion, we have developed an efficient radical-mediated bidirectional C-C bond cleavage/coupling strategy for carbocycle editing, offering a straightforward approach to synthesizing 1,n-difunctionalized alkyl linchpins. A wide range of *gem*-diperoxides, easily available from cyclic ketones, have been successfully explored as efficient bidirectional radical linchpins. These compounds undergo a tandem alkoxy radical-induced *β*-scission/coupling and acyloxy radical-mediated decarboxylation/coupling under iron or copper catalysis, delivering one-carbon-shortened alkyl 1,n-dithiocyanides, 1,n-diazides, and 1,n-dihalides in good yields with excellent functional group tolerance (*n* ≥ 4). Additionally, a one-pot, two-step protocol has been developed to access unsymmetric aliphatic 1,n-thiocyanate-azides. This protocol is highlighted by its broad substrate scope, accommodating diverse ring sizes, functional groups, and ring systems (monocyclic, bridged, and fused rings). Remarkably, dicarbonyl and tricarbonyl steroidal ketones exhibit outstanding regioselectivity in the activation of cyclic ketones, enabling selective deconstruction and functionalization of fused carbocycles, preferentially occurring at sites with minimal steric hindrance. This bidirectional C-C bond cleavage/coupling strategy provides an efficient approach for the synthesis of complex 1,n-difunctionalized alkyl molecules and for molecular editing. This research represents a prime example of radical-mediated bidirectional linchpin coupling and overcomes the limitation of restricted chain length in known reactions.

## Methods

### General procedures for 1,n-dithiocyanation and 1,n-diazidation

A 10 mL oven-dried Schlenk-tube equipped with a magnetic stirrer was charged with Fe(OTf)_2_ (0.01 mmol, 5 mol%). Then, the tube was evacuated and backfilled with nitrogen (three times). Subsequently, a solution of *gem*-diperoxides **S** (0.20 mmol, 1.0 equiv.) and TMSNCS or TMSN_3_ (0.60 mmol, 3.0 equiv.) in EA (2.0 mL) was added by a syringe. The reaction mixture was stirred at 50 °C for 3 h. After that, the reaction mixture was concentrated in vacuo and purified by flash column chromatography on silica gel according to the general procedure, using a gradient eluent of petroleum ether/ethyl acetate (10:1 to 4:1) to afford products **1**–**35**, and a gradient eluent of petroleum ether/dichloromethane (5:1 to 1:1) to afford products **36**–**56**.

### General procedures for 1,n-dihalogenation

A 10 mL oven-dried Schlenk-tube equipped with a magnetic stirrer was charged with CuOTf (0.04 mmol, 20 mol%). Then, the tube was evacuated and backfilled with nitrogen (three times). Subsequently, a solution of *gem*-diperoxides **S** (0.20 mmol, 1.0 equiv.) and MgCl_2_ or MgBr_2_ (0.60 mmol, 3.0 equiv.) in CH_3_CN (2.0 mL) was added by a syringe. The reaction mixture was stirred at 65 °C for 3 h. After that, the reaction mixture was concentrated in vacuo and purified by flash column chromatography on silica gel according to the general procedure, using a gradient eluent of petroleum ether/dichloromethane (5:1 to 2:1) to give the desired products **57**–**61**.

## Supplementary information

Supplementary Information

Transparent Peer Review file

## Data Availability

All data supporting the findings of this study are available within the article and its Supplementary Information. All data are also available from the corresponding author upon request.
