# Supplementary material for: Carbocycle editing of ketones proceeds via a radical-mediated bidirectional C-C bond cleavage/coupling strategy
Source: Nat Commun. 2026 Jun 15;17:7537. doi: 10.1038/s41467-026-74317-0 (PMC13407898; doi:10.1038/s41467-026-74317-0)
Supplement: Supplementary file 1 — Supplementary Information [file 41467_2026_74317_MOESM1_ESM.pdf]

Supplementary Information

Carbocycle Editing of Ketones Proceeds via a Radical-Mediated Bidirectional C-C Bond Cleavage/Coupling Strategy

Ying-Jie Ma[1], Yuan Gao[1], Xin Chen[1], Le Liu[1], Xin-Hua Duan[1,2], and Li-Na Guo[1,*]

[1]Department of Chemistry, School of Chemistry, Xi'an Key Laboratory of Sustainable Energy Material Chemistry and Engineering Research Center of Energy Storage Materials and Devices, Ministry of Education, Xi'an Jiaotong University, Xi'an 710049, China.

[2]State Key Laboratory of Natural Product Chemistry, Lanzhou University, Lanzhou 730000, China.

*Corresponding authors. E-mail: guoln81@xjtu.edu.cn

Table of Contents

1. General information 4

2. Starting materials 5

2.1 List of *gem*-diperoxides 5

2.2 General procedure for the preparation of *gem*-diperoxides 7

2.2.1 Preparation of *gem*-dihydroperoxides 7

2.2.2 Preparation of *gem*-diperoxides 8

3. Optimization of reaction conditions 9

3.1 General procedure for 1, n-difunctionalization of *gem*-diperoxides via bidirectional C-C bond cleavage . 9

3.2 Optimization of distal dithiocyanation 9

3.3 Optimization of distal diazidation 13

3.4 Optimization of distal dihalogenation 15

3.5 General procedure for unsymmetrical 1,n-difunctionaliztion of *gem*-diperoxides 18

3.6 Optimization of azidation-thiocyanation reaction 18

4. General procedures for Fig. 3, Fig.4 and Fig.5 20

4.1 Representative procedure for 1,n-dithiocyanation reaction 20

4.2 Representative procedure for 1,n-diazidation reaction 20

4.3 Representative procedure for 1,n-dihalogenation reaction 20

4.4 Representative procedure for the azidation and thiocyanation reaction 21

4.5 Representative procedure for the azidation and cyanation reaction 21

5. Synthetic applications 22

6. Scale-up synthesis 27

6.1 Large-scale synthesis of dithiocyanation 27

6.2 Large-scale synthesis of diazidation 27

7. Mechanistic investigation 29

7.1 Radical inhibiting experiment 29

7.2 Detection of byproducts 29

7.3 Detection of intermediate 30

7.4 Cyclic voltammetry data 31

7.5 Simultaneous thermal analysis (DSC-TGA) of *gem*-diperoxides 33

8. Investigation of reactivity of *α*-substituted ketones 39

8.1 Oxidation of progesterone 39

8.2 Oxidation of acyclic ketones and α-substituted ketones 39

9. Experimental and characterization data 41

9.1 Characterization data of starting materials 41

9.2 Characterization data of products 65

10. NMR spectra of starting materials 94

11. NMR spectra of products 176

Supplementary References 275

1. General information

Unless otherwise noted, all reactions were carried out under nitrogen atmosphere. Reagents and solvents obtained from commercial suppliers were used without further purification. Analytical TLC: aluminum backed plates pre-coated (0.25 mm) with Merck Silica Gel 60F-254. Products containing azide and thiocyanate groups were visualized by exposure to iodine vapor, whereas *gem*-diperoxides could be detected using a chromogenic reagent composed of DMPD·2HCl/acetic acid (1.5:1, w/w) in methanol/water (5:1, v/v). Column chromatography purifications were carried out using 200–300 mesh silica gel. ^{1}H NMR, ^{13}C NMR, ^{19}F NMR and ^{31}P NMR spectra were recorded on a JNM-ECZ400S/L1 400 MHz spectrometer or Bruker AM 400 MHz spectrometer at ambient temperature. Coupling constants are reported in Hz with multiplicities denoted as s (singlet), d (doublet), t (triplet), q (quartet), dd (doublet of doublets), td (triplet of doublets), m (multiplet) and bs (broad). HRMS was obtained on an Agilent 6210 ESI/TOF MS with an ESI source. Cyclic voltammetry was conducted on a Princeton PARSTAT VersaSCAN electrochemical workstation. Simultaneous thermal analysis (DSC-TGA) was performed using a METTLER TOLEDO TGA/DSC 3+ instrument.

2. Starting materials

2.1 List of *gem*-diperoxides[a, b]

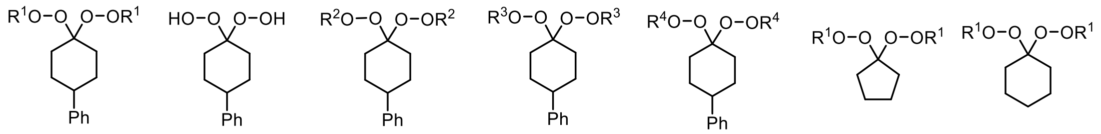

S1, 41% **S1'**, 52% **S1(I)**, 30% **S1(II)**, 34% **S1(III)**, 36% **S2**, 50% **S3**, 35%

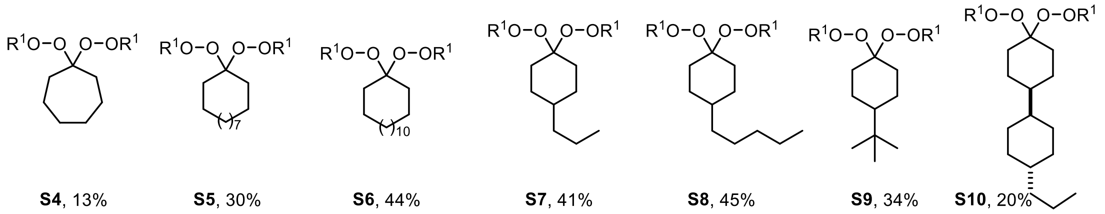

S4, 13% **S5**, 30% **S6**, 44% **S7**, 41% **S8**, 45% **S9**, 34% **S10**, 20%

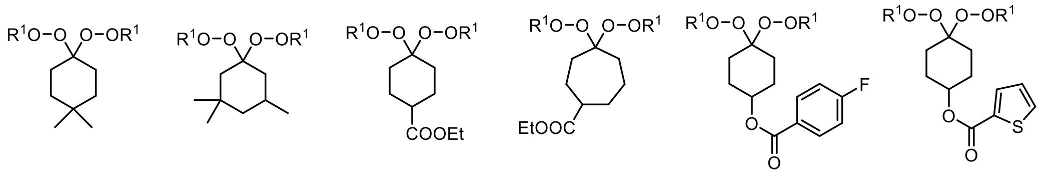

S11, 44% **S12**, 52% **S13**, 68% **S14**, 35% **S15**, 36% **S16**, 45%

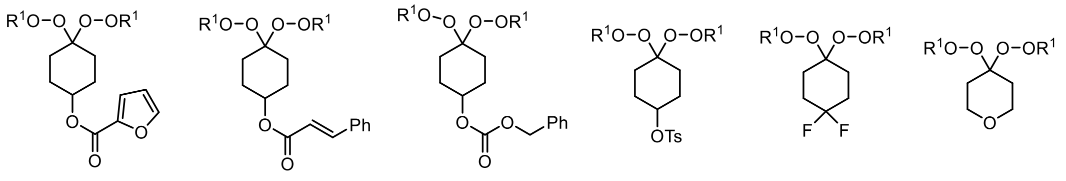

S17, 23% **S18**, 36% **S19**, 42% **S20**, 28% **S21**, 33% **S22**, 35%

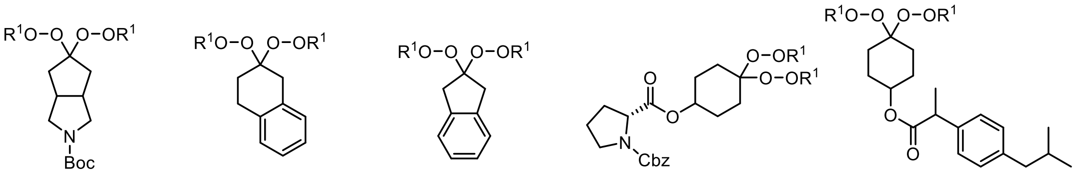

S23, 44% **S24**, 18% **S25**, 49% **S26**, 59% **S27**, 56%

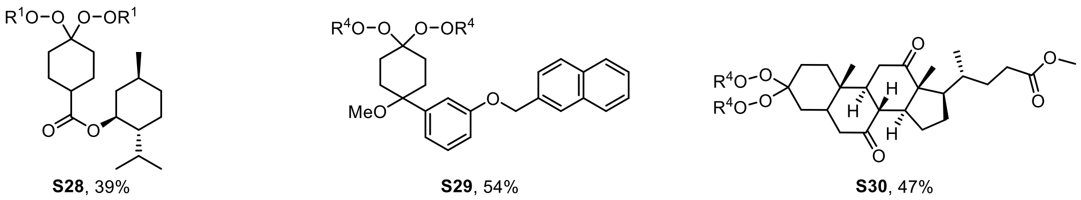

S28, 39% **S29**, 54% **S30**, 47%

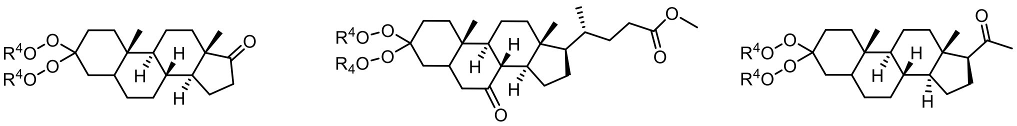

S31, 49% **S32**, 29% **S33**, 69%

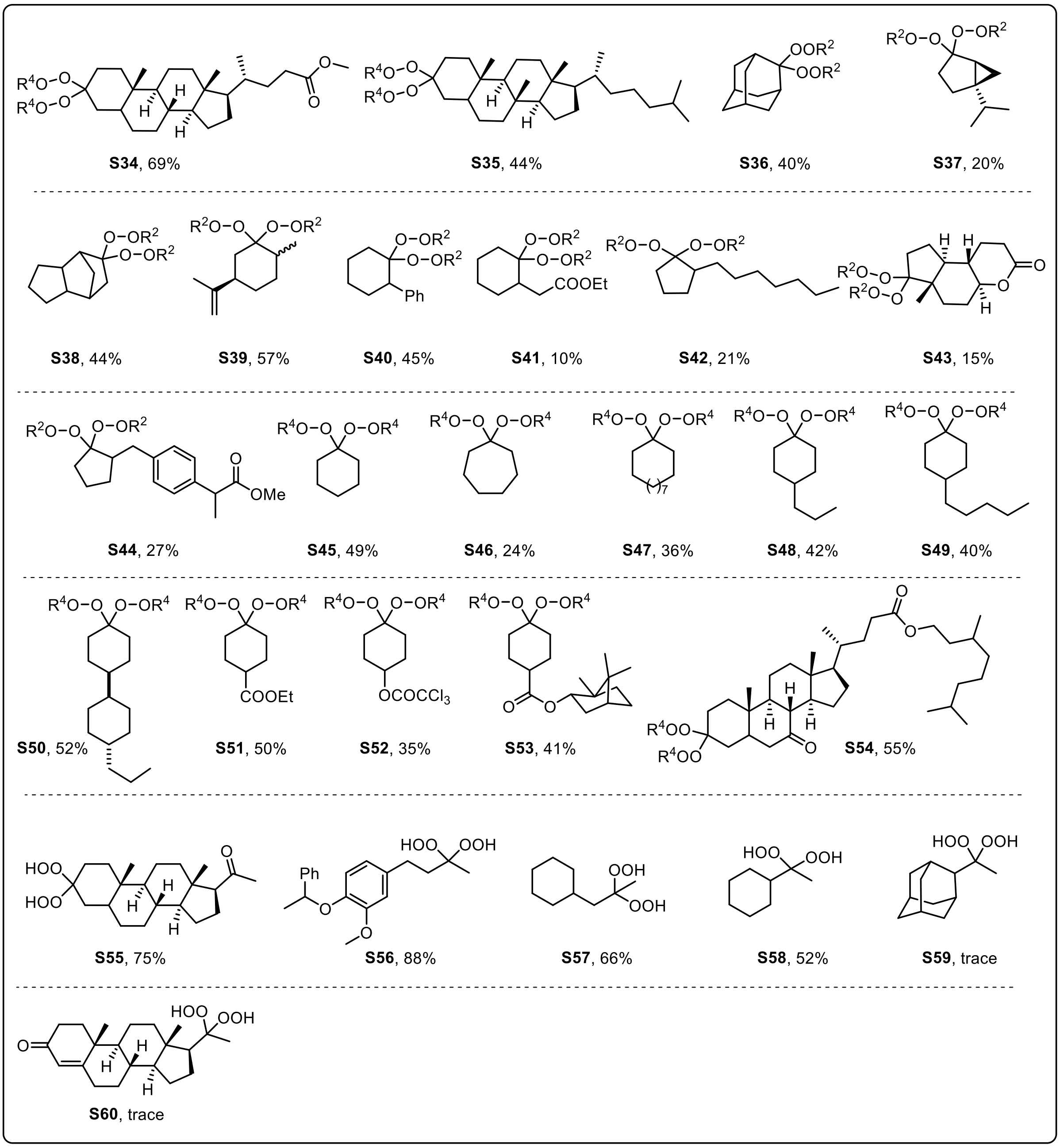

[a] *Gem-diperoxides*: R^1 = $COCH_2Cl$, R^2 = TMS, R^3 = 4-BrBz, R^4 = 4-FBz. [b] Isolated total yield over two steps (based on the starting ketone).

Note:

1. *Gem*-diperoxides can be stored long-term at -20 °C and remain stable under such conditions.

2. *Gem*-diperoxides should not be exposed to strong reducing agents or prolonged direct sunlight.

3. Handle with care; avoid strong impacts or rough handling.

2.2 General procedure for the preparation of *gem*-diperoxides

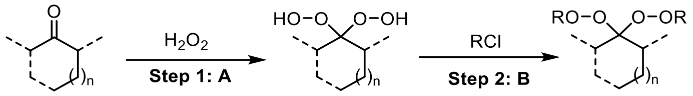

2.2.1 Preparation of *gem*-dihydroperoxides

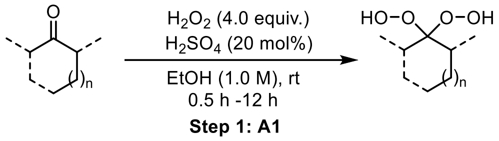

Step 1: A1[1]

To a 100 mL reaction tube was added a solution of H_2O_2 (30 wt% in H_2O, 4.0 equiv.), and conc. H_2SO_4 (20 mol%). Then a solution of cyclanone (1.0 equiv.) in EtOH (1.0 M) was added dropwise. The reaction mixture was stirred vigorously at room temperature for 0.5–12 h and monitored by TLC. The aqueous layer was extracted with DCM (3 × 50 mL). The combined organic layer was washed with brine, dried over Na_2SO_4 and concentrated to afford residue (the temperature should not exceed 30 °C), which was purified by flash column chromatography on silica gel, using a gradient eluent of petroleum ether/ethyl acetate (10:1 to 3:1) to give *gem*-dihydroperoxides.

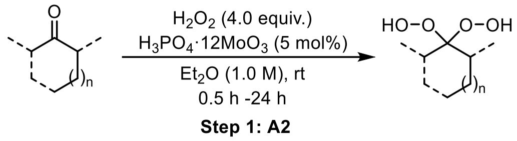

Step 1: A2[2]

To a 100 mL reaction tube was added a solution of H_2O_2 (30 wt% in H_2O, 4.0 equiv.) and $H_3PO_4 \cdot 12MoO_3$ (5 mol%). Then a solution of cyclanone (1.0 equiv.) in Et_2O (1.0 M) was added dropwise. The reaction mixture was stirred vigorously at room temperature for 0.5–24 h and monitored by TLC. The aqueous layer was extracted with DCM (3 × 50 mL). The combined organic layer was washed with brine, dried over Na_2SO_4 and concentrated to afford residue (the temperature should not exceed 30 °C), which was purified by column chromatography on silica gel, using a gradient eluent of petroleum ether/ethyl acetate (10:1 to 3:1) to give *gem*-dihydroperoxides.

2.2.2 Preparation of *gem*-diperoxides

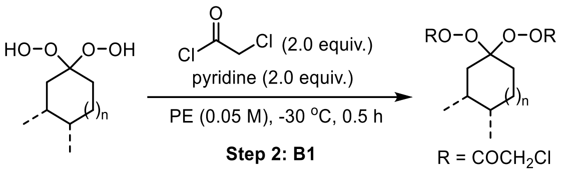

Step 2: B1

To a 100 mL round-bottom flask was added a solution of *gem*-dihydroperoxide (1.0 equiv.) in DCM (2–5 mL), together with petroleum ether (PE) (0.05 M). The mixture was stirred at -30 °C for 5 min, then pyridine (2.0 equiv.) and chloroacetyl chloride (2.0 equiv.) were added to the mixture dropwise. The mixture was stirred for 0.5 h. After the reaction completed, the mixture was filtered through a Buchner funnel and the residue was rinsed with PE/DCM (1:1, 100–200 mL). The filtrate was concentrated to afford residue (the temperature should not exceed 30 °C), which was purified by column chromatography on silica gel, eluting with petroleum ether/dichloromethane (5:1 to 1:1) to give the target products *gem*-diperoxides.

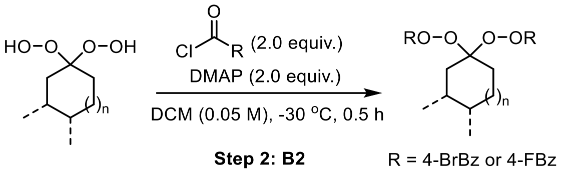

Step 2: B2

To a 100 mL round-bottom flask was added a solution of *gem*-dihydroperoxide (1.0 equiv.) in DCM (0.05 M). The mixture was stirred at -30 °C for 5 min, then DMAP (2.0 equiv.) and RCOCl (2.0 equiv.) were added to the mixture dropwise. The mixture was stirred for 0.5 h. After completion of the reaction, it was cooled to 0 °C and quenched with H_2O and extracted with EtOAc. The extract was washed with brine and dried over Na_2SO_4 and concentrated to afford residue (the temperature of concentration should not exceed 30 °C) and which was purified by column chromatography on silica gel, eluting with petroleum ether/ethyl acetate (20:1 to 5:1) to give the target products *gem*-diperoxides.

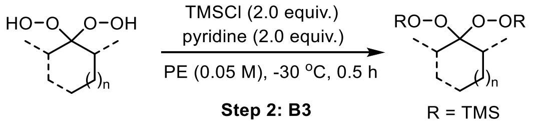

Step 2: B3

To a 100 mL round-bottom flask was added a solution of *gem*-diperoxides (1.0 equiv.) in DCM (2–5 mL), together with PE (0.05 M). The mixture was stirred at -30 °C for 5 min, then pyridine (2.0 equiv.) and TMSCl (2.0 equiv.) were added to the mixture dropwise. The mixture was stirred for 0.5 h. After the reaction completed, the mixture was filtered by a Buchner funnel and residue was rinsed with PE/DCM (1:1, 100–200 mL). The filtrate was concentrated to afford residue (the temperature of concentration should not exceed 30 °C), which was purified by column chromatography on silica gel, eluting with petroleum ether/dichloromethane (5:1 to 1:1) to give the target products *gem*-diperoxides.

3. Optimization of reaction conditions

3.1 General procedure for 1, n-difunctionalization of *gem*-diperoxides via bidirectional C-C bond cleavage

A 10 mL oven-dried Schlenk-tube equipped with a magnetic stirrer was charged with catalyst. Then, the tube was evacuated and backfilled with nitrogen (three times). Subsequently, a solution of *gem*-diperoxides (0.20 mmol, 1.0 equiv.) and nucleophiles (0.60 mmol, 3.0 equiv.) in solvent (2.0 mL) was added by a syringe. The reaction mixture was stirred at a certain temperature for 3 h. After that, the reaction mixture was concentrated in vacuo and purified by flash chromatography on silica gel, eluting with petroleum ether/ethyl acetate (5:1) to give desired product **1**, petroleum ether/dichloromethane (4:1) to afford product **41** and petroleum ether/dichloromethane (5:1) to afford product **57**.

3.2 Optimization of distal dithiocyanation

Supplementary Table 1: *Evaluation of leaving group* [a]

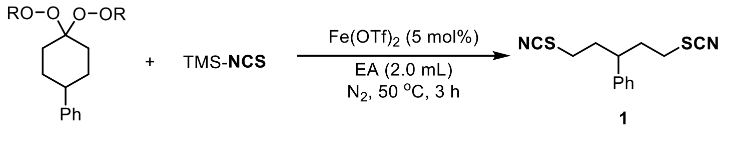

Entry	S	Yield (%)[b]
1	**S1'**	trace
2	**S1(I)**	40
3	**S1**	**69**
4	**S1(II)**	36
5	**S1(III)**	48

[a] Reaction conditions: $Fe(OTf)_2$ (5 mol%), *gem*-diperoxides (0.2 mmol, 1.0 equiv.), TMSNCS (0.6 mmol, 3.0 equiv.), EA (2.0 mL), 50 °C, for 3 h, under N_2. [b] Isolated yield.

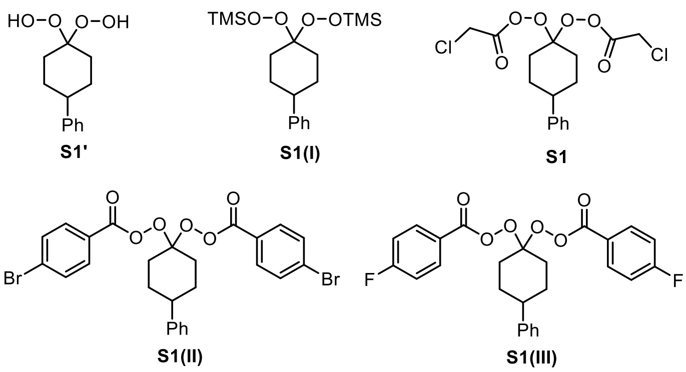

Supplementary Table 2: *Evaluation of catalysts* [a]

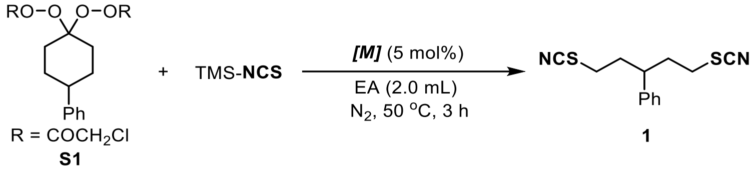

Entry	[M]	Yield (%)[b]
1	**$Fe(OTf)_2$**	**69**
2	$Fe(OTf)_3$	64
3	$Fe(OTs)_3$	55
4	$Fe(OAc)_2$	30
5	$Fe(acac)_2$	6
6	$FeCl_2$	34
7	$Fe(NTf)_2$	25
8	$Cu(CH_3CN)_4PF_6$	41
9	$Cu(CH_3CN)_4BF_4$	29
10	CuOTf	33
11	$NiBr_2(PPh_3)_2$	nd
12	-	nd

[a] Reaction conditions: [M] (5 mol%), **S1** (0.2 mmol, 1.0 equiv.), TMSNCS (0.6 mmol, 3.0 equiv.), EA (2.0 mL), 50 °C, for 3 h, under N_2. [b] Isolated yield.

Supplementary Table 3: *Evaluation of solvent* [a]

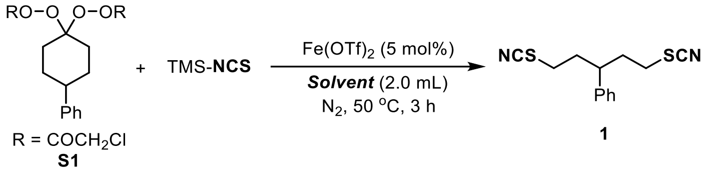

Entry	Solvent	Yield (%)[b]
1	DMSO	nd
2	DMF	trace
3	CH_3CN	29
4	CH_3OH	nd
5	Acetone	5
**6**	**EA**	**69**
7	DCM	59
8	THF	35
9	2-Me THF	18
10	MTBE	30
11	1,4-Dioxane	35
12	Et_2O	7
13	PhCl	49

[a] Reaction conditions: $Fe(OTf)_2$ (5 mol%), **S1** (0.2 mmol, 1.0 equiv.), TMSNCS (0.6 mmol, 3.0 equiv.), Solvent (2.0 mL), 50 °C, for 3 h, under N_2. [b] Isolated yield.

Supplementary Table 4: *Evaluation of ratio* [a]

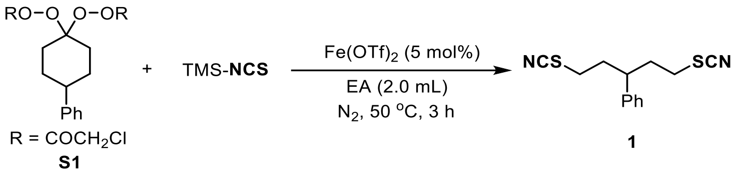

Entry	**S1**: TMSNCS	Yield (%)[b]
1	1:2	58
**2**	**1:3**	**69**
3	1:4	65

[a] Reaction conditions: $Fe(OTf)_2$ (5 mol%), **S1** (0.2 mmol, 1.0 equiv.), TMSNCS (x equiv.), EA (2.0 mL), 50 °C, for 3 h, under N_2. [b] Isolated yield.

Supplementary Table 5: *Evaluation of temperature* [a]

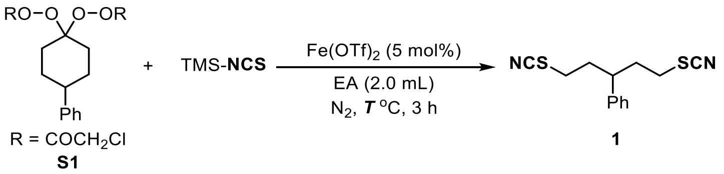

Entry	T	Yield (%)[b]
1	rt	60
2	40	64
**3**	**50**	**69**
4	70	57

[a] Reaction conditions: $Fe(OTf)_2$ (5 mol%), **S1** (0.2 mmol, 1.0 equiv.), TMSNCS (0.6 mmol, 3.0 equiv.), EA (2.0 mL), T °C, for 3 h, under N_2. [b] Isolated yield.

Supplementary Table 6: *Evaluation of "SCN" source [a]*

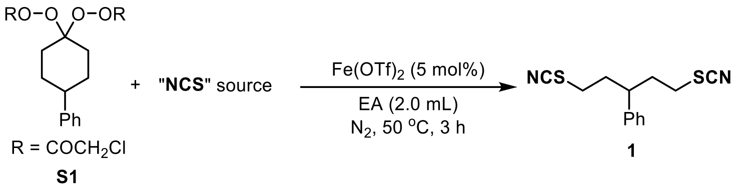

Entry	Source	Yield (%)[b]
**1**	**TMSNCS**	**69**
2	KSCN	trace
3	NH_4SCN	trace

[a] Reaction conditions: $Fe(OTf)_2$ (5 mol%), **S1** (0.2 mmol, 1.0 equiv.), "SCN" source (3.0 equiv.), EA (2.0 mL), 50 °C, for 3 h, under N_2. [b] Isolated yield.

3.3 Optimization of distal diazidation

Supplementary Table 7: *Evaluation of solvents [a]*

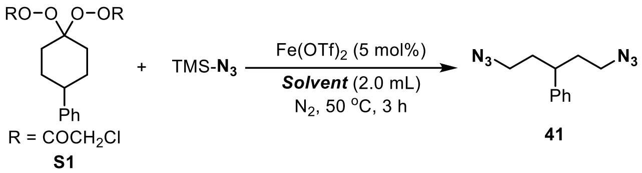

Entry	Solvent	Yield (%)[b]
1	DMSO	nd
2	DMF	nd
3	CH_3CN	trace
4	CH_3OH	trace
5	Acetone	nd
**6**	**EA**	**62**
7	DCM	trace
8	THF	49
9	2-Me THF	44
10	MTBE	32
11	1,4-Dioxane	25
12	Et_2O	49
13	DME	26
14	PhCl	trace

[a] Reaction conditions: $Fe(OTf)_2$ (5 mol%), **S1** (0.2 mmol, 1.0 equiv.), $TMSN_3$ (0.6 mmol, 3.0 equiv.), Solvent (2.0 mL), 50 °C, for 3 h, under N_2. [b] Isolated yield.

Supplementary Table 8: *Evaluation of catalysts* [a]

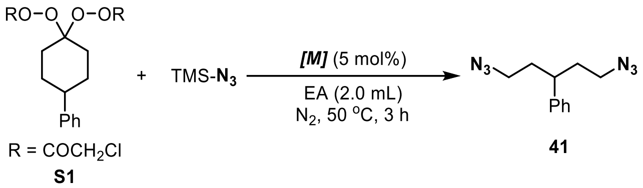

Entry	[M]	Yield (%)[b]
**1**	**$Fe(OTf)_2$**	**62**
2	$Fe(OTs)_3$	15
3	$Fe(OAc)_2$	5
4	$Fe(acac)_2$	7
5	$FeCl_2$	trace
6	$Cu(CH_3CN)_4PF_6$	trace
7	$Cu(CH_3CN)_4$ BF_4	trace
8	CuOTf	trace
9	$NiBr_2(PPh_3)_2$	nd

[a] Reaction conditions: [M] (5 mol%), **S1** (0.2 mmol, 1.0 equiv.), $TMSN_3$ (0.6 mmol, 3.0 equiv.), EA (2.0 mL), 50 °C, for 3 h, under N_2. [b] Isolated yield.

Supplementary Table 9: *Evaluation of ratio* [a]

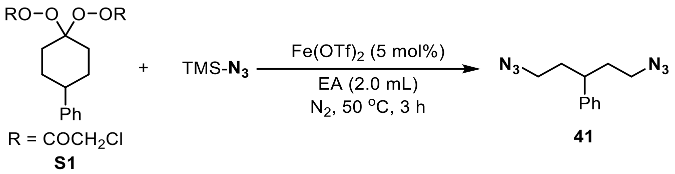

Entry	**1a**: $TMSN_3$	Yield (%)[b]
1	1:2	47
**2**	**1:3**	**62**
3	1:4	58

[a] Reaction conditions: $Fe(OTf)_2$ (5 mol%), **S1** (0.2 mmol, 1.0 equiv.), $TMSN_3$ (x equiv.), EA (2.0 mL), 50 °C, for 3 h, under N_2. [b] Isolated yield.

Supplementary Table 10: *Evaluation of temperature* [a]

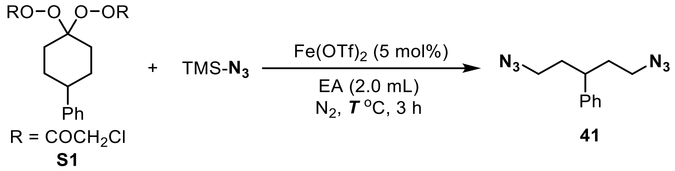

Entry	T	Yield (%)[b]
1	rt	38
2	40	59
**3**	**50**	**62**
4	70	57

[a] Reaction conditions: $Fe(OTf)_2$ (5 mol%), **S1** (0.2 mmol, 1.0 equiv.), $TMSN_3$ (0.6 mmol, 3.0 equiv.), EA (2.0 mL), T °C, for 3 h, under N_2. [b] Isolated yield.

3.4 Optimization of distal dihalogenation

Supplementary Table 11*: Evaluation of solvents* [a]

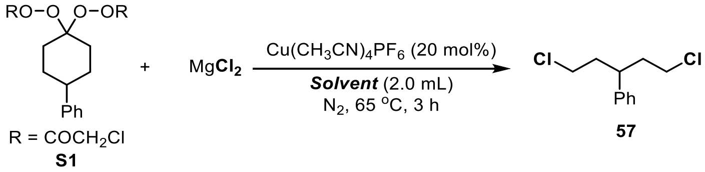

Entry	Solvent	Yield (%)[b]
1	DMSO	<5
2	DMF	15
3	CH_3NO_3	nd
**4**	**CH_3CN**	**56**
5	Acetone	nd
6	THF	51
7	2-Me THF	nd
8	1,4-Dioxane	37
9	EA	22
10	DCE	nd
11	MTBE	21
12	DCM	nd

[a] Reaction conditions: $Cu(CH_3CN)_4PF_6$ (20 mol%), **S1** (0.2 mmol, 1.0 equiv.), $MgCl_2$ (0.6 mmol, 3.0 equiv.), solvent (2.0 mL), 65 °C, for 3 h, under N_2. [b] Isolated yield.

Supplementary Table 12: *Evaluation of catalysts* [a]

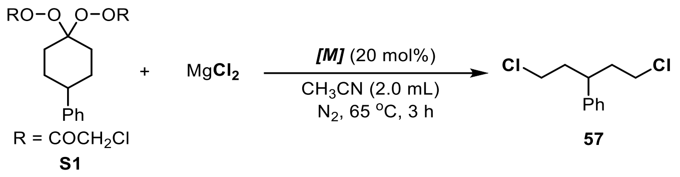

Entry	[M]	Yield (%)[b]
1	$Cu(CH_3CN)_4PF_6$	56
2	$Cu(CH_3CN)_4BF_4$	50
**3**	**CuOTf**	**63**
4	$Cu(acac)_2$	10
5	CuCl	trace
6	$Cu(CH_3COO)_2{\cdot}H_2O$	trace
7	$Fe(OTf)_2$	trace
8	$NiBr_2(PPh_3)_2$	nd
9	$NiCl_2$	trace

[a] Reaction conditions: [M] (20 mol%), **S1** (0.2 mmol, 1.0 equiv.), $MgCl_2$ (0.6 mmol, 3.0 equiv.), CH_3CN (2.0 mL), 65 °C, for 3 h, under N_2. [b] Isolated yield.

Supplementary Table 13: *Evaluation of ratio* [a]

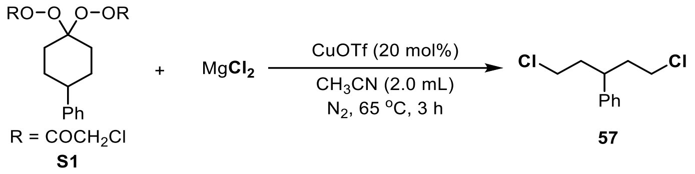

Entry	**S1**: $MgCl_2$	Yield (%)[b]
1	1:2	48
**2**	**1:3**	**63**
3	1:4	61

[a] Reaction conditions: CuOTf (20 mol%), **S1** (0.2 mmol, 1.0 equiv.), $MgCl_2$ (x equiv.), CH_3CN (2.0 mL), 65 °C, for 3 h, under N_2. [b] Isolated yield.

Supplementary Table 14: *Evaluation of source* [a]

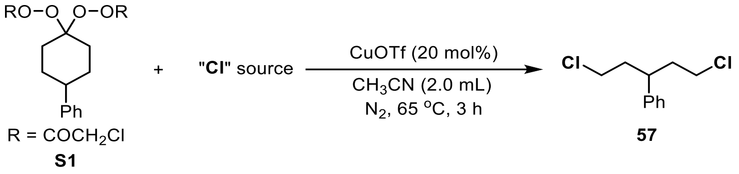

Entry	"Cl" source	Yield (%)[b]
1	HCl	nd
**2**	**$MgCl_2$**	**63**
3	TMSCl	trace

[a] Reaction conditions: CuOTf (20 mol%), **S1** (0.2 mmol, 1.0 equiv.), source (0.6 mmol, 3.0 equiv.), CH_3CN (2.0 mL), 65 °C, for 3 h, under N_2. [b] Isolated yield.

Supplementary Table 15: *Evaluation of temperature* [a]

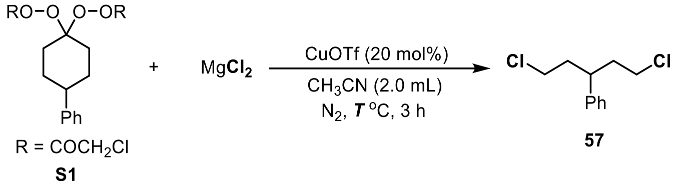

Entry	T	Yield (%)[b]
1	rt	nd
2	50	56
**3**	**65**	**63**
4	80	60

[a] Reaction conditions: CuOTf (20 mol%), **S1** (0.2 mmol, 1.0 equiv.), $MgCl_2$ (0.6 mmol, 3.0 equiv.), CH_3CN (2.0 mL), T °C, for 3 h, under N_2. [b] Isolated yield.

Supplementary Table 16: *Evaluation of catalyst loading* [a]

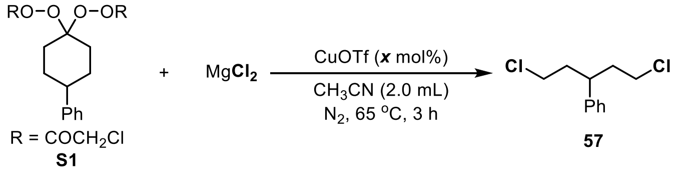

Entry	x	Yield (%)[b]
1	5	42
2	10	58
**3**	**20**	**63**

[a] Reaction conditions: CuOTf (x mol%), **S1** (0.2 mmol, 1.0 equiv.), $MgCl_2$ (0.6 mmol, 3.0 equiv.), CH_3CN (2.0 mL), 65 °C, for 3 h, under N_2. [b] Isolated yield.

3.5 General procedure for unsymmetrical 1,n-difunctionaliztion of *gem*-diperoxides

A 10 mL oven-dried Schlenk-tube equipped with a magnetic stirrer was charged with $Fe(OTf)_2$ (5 mol%). Then, the tube was evacuated and backfilled with nitrogen (three times). Subsequently, a solution of *gem*-diperoxides **S1(III)** (0.20 mmol, 1.0 equiv.) and $TMSN_3$ (0.20 mmol, 1.0 equiv.) in solvent (1.0 mL) was added by a syringe. The reaction mixture was stirred at 40 °C for 3 h. After that, TMSNCS (x equiv.) in solvent (1.0 mL) was added and the mixture was stirred at 50 °C for 3 h. The reaction mixture was concentrated in vacuo and purified by flash chromatography on silica gel, eluting with petroleum ether/ethyl acetate (10:1) to give desired product.

3.6 Optimization of azidation-thiocyanation reaction

Supplementary Table 17: *Evaluation of solvent*[a]

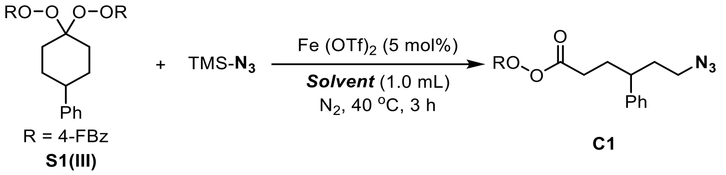

Entry	Solvent	Assay yield (%)[b]
1	DMSO	trace
2	CH_3CN	20
3	Acetone	trace
4	THF	30
5	2-Me THF	51
6	1,4-dioxane	15
**7**	**EA**	**84**
8	DCE	nd
9	MTBE	trace
10	DCM	nd
11	Toluene	nd

[a] Reaction conditions: $Fe(OTf)_2$ (5 mol%), **S1(III)** (0.2 mmol, 1.0 equiv.), $TMSN_3$ (0.2 mmol, 1.0 equiv.), solvent (1.0 mL), 40 °C, for 3 h, under N_2. [b] NMR yield, ext. std. = mesitylene.

Supplementary Table 18: *Evaluation of solvent* [a]

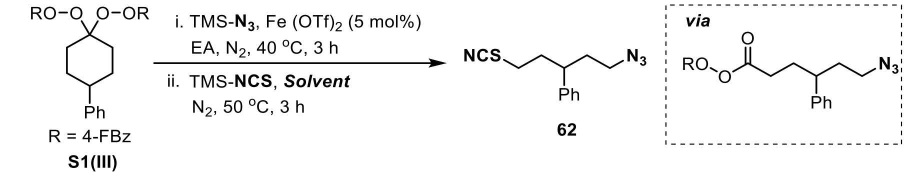

Entry	Solvent (1.0 mL)	Yield (%)[b]
1	CH_3CN	<5
2	THF	20
3	2-Me THF	23
4	1,4-dioxane	<5
**5**	**EA**	**53**

[a] Reaction conditions: $Fe(OTf)_2$ (5 mol%), **S1(III)** (0.2 mmol, 1.0 equiv.), $TMSN_3$ (0.2 mmol, 1.0 equiv.), EA (1.0 mL), 40 °C, for 3 h, under N_2. Then solvent (1.0 mL), TMSNCS (0.2 mmol, 1.0 equiv.), 50 °C, for 3 h, under N_2. [b]Isolated yield.

Supplementary Table 19: *Screening of amounts of TMSNCS* [a]

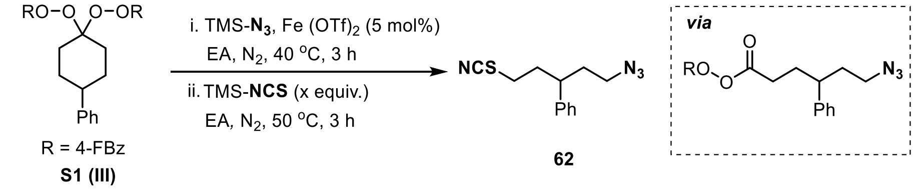

Entry	x	Yield (%)[b]
1	1	53
2	1.2	55
3	1.5	55
**4**	**2.0**	**60**

[a] Reaction conditions: $Fe(OTf)_2$ (5 mol%), **S1((III)** (0.2 mmol, 1.0 equiv.), $TMSN_3$ (0.2 mmol, 1.0 equiv.), EA (1.0 mL), 40 °C, for 3 h, under N_2. Then EA (1.0 mL), TMSNCS (x equiv.), 50 °C, for 3 h, under N_2. [b] Isolated yield.

Supplementary Table 20: *Exchange addition sequence of nucleophiles* [a]

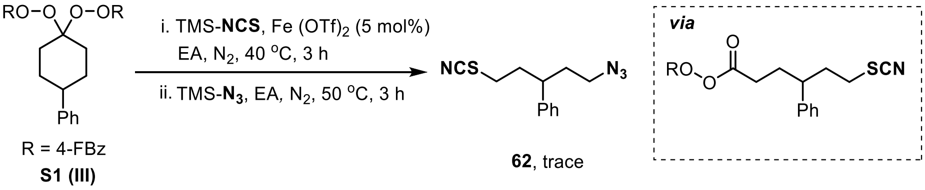

[a] Reaction conditions: $Fe(OTf)_2$ (5 mol%), **S1((III)** (0.2 mmol, 1.0 equiv.), TMSNCS (0.2 mmol, 1.0 equiv.), EA (1.0 mL), 40 °C, for 3 h, under N_2. Then EA (1.0 mL), $TMSN_3$ (2.0 equiv.), 50 °C, for 3 h, under N_2.

4. General procedures for Fig. 3, Fig.4 and Fig.5

4.1 Representative procedure for 1,n-dithiocyanation reaction

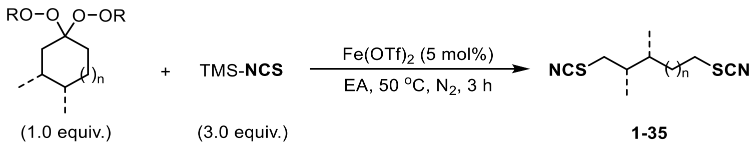

1-35

A 10 mL oven-dried Schlenk-tube equipped with a magnetic stirrer was charged with $Fe(OTf)_2$ (0.01 mmol, 5 mol%). Then, the tube was evacuated and backfilled with nitrogen (three times). Subsequently, a solution of *gem*-diperoxides (0.20 mmol, 1.0 equiv.) and TMSNCS (0.60 mmol, 3.0 equiv.) in EA (2.0 mL) was added by a syringe. The reaction mixture was stirred at 50 °C for 3 h. After that, the reaction mixture was concentrated in vacuo and purified by flash column chromatography on silica gel according to the general procedure, using a gradient eluent of petroleum ether/ethyl acetate (10:1 to 4:1) to afford products **1**–**35**.

4.2 Representative procedure for 1,n-diazidation reaction

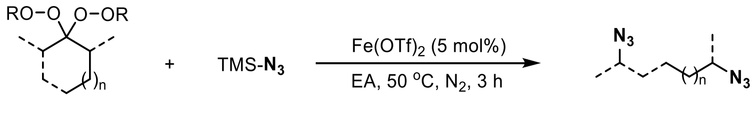

36-56

A 10 mL oven-dried Schlenk-tube equipped with a magnetic stirrer was charged with $Fe(OTf)_2$ (0.01 mmol, 5 mol%). Then, the tube was evacuated and backfilled with nitrogen (three times). Subsequently, a solution of *gem*-diperoxides (0.20 mmol, 1.0 equiv.) and $TMSN_3$ (0.60 mmol, 3.0 equiv.) in EA (2.0 mL) was added by a syringe. The reaction mixture was stirred at 50 °C for 3 h. After that, the reaction mixture was concentrated in vacuo and purified by flash column chromatography on silica gel according to the general procedure, using a gradient eluent of petroleum ether/dichloromethane (5:1 to 1:1) to afford products **36**–**56**.

4.3 Representative procedure for 1,n-dihalogenation reaction

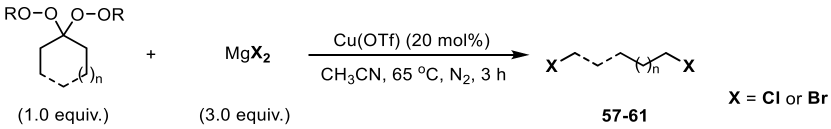

57-61

A 10 mL oven-dried Schlenk-tube equipped with a magnetic stirrer was charged with CuOTf (0.04 mmol, 20 mol%). Then, the tube was evacuated and backfilled with nitrogen (three times). Subsequently, a

solution of *gem*-diperoxides (0.20 mmol, 1.0 equiv.) and $MgCl_2$ or $MgBr_2$ (0.60 mmol, 3.0 equiv.) in CH_3CN (2.0 mL) was added by a syringe. The reaction mixture was stirred at 65 °C for 3 h. After that, the reaction mixture was concentrated in vacuo and purified by flash column chromatography on silica gel according to the general procedure, using a gradient eluent of petroleum ether/dichloromethane (5:1 to 2:1) to give the desired products **57**–**61**.

4.4 Representative procedure for the azidation and thiocyanation reaction

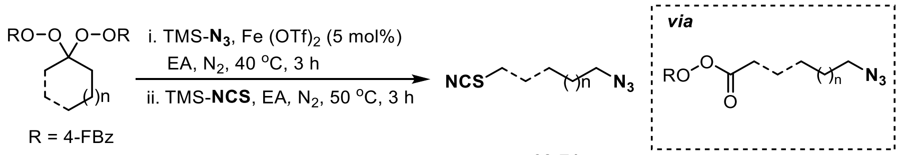

62-71

A 10 mL oven-dried Schlenk-tube equipped with a magnetic stirrer was charged with $Fe(OTf)_2$ (0.01 mmol, 5 mol%). Then, the tube was evacuated and backfilled with nitrogen (three times). Subsequently, a solution of *gem*-diperoxides (0.20 mmol, 1.0 equiv.) and $TMSN_3$ (0.20 mmol, 1.0 equiv.) in EA (1.0 mL) was added by a syringe. The reaction mixture was stirred at 40 °C for 3 h. After that, TMSNCS (2.0 equiv.) in EA (1.0 mL) was added and stirred at 50 °C for 3 h. The reaction mixture was concentrated in vacuo and purified by flash chromatography on silica gel using a gradient eluent of petroleum ether/ethyl acetate (10:1 to 5:1) to give desired product **62-71**.

4.5 Representative procedure for the azidation and cyanation reaction

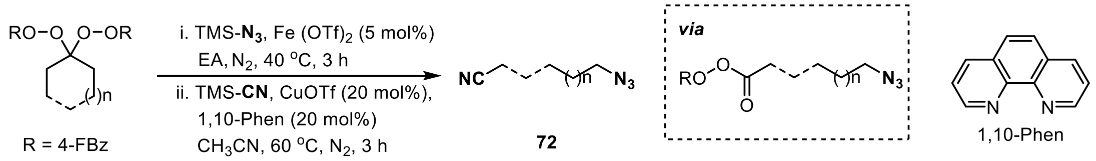

72

A 10 mL oven-dried Schlenk-tube equipped with a magnetic stirrer was charged with $Fe(OTf)_2$ (0.01 mmol, 5 mol%). Then, the tube was evacuated and backfilled with nitrogen (three times). Subsequently, a solution of *gem*-diperoxides (0.20 mmol, 1.0 equiv.) and $TMSN_3$ (0.20 mmol, 1.0 equiv.) in EA (1.0 mL) was added by a syringe. The reaction mixture was stirred at 40 °C for 3 h. After that, 2 mL of Na_2CO_3 (1M) was added to quench the reaction and extract it with ethyl acetate (5 mL × 3). The organic layer was concentrated in vacuo (the temperature should not exceed 30 °C).

Then, A 10 mL oven-dried Schlenk-tube equipped with a magnetic stirrer was charged with CuOTf (20 mol%), 1,10-Phen (20 mol%), and backfilled with the atmosphere with nitrogen three times. Add TMSCN (2.0 equiv.) and transfer the dried reaction mixture from the first step via syringe using MeCN (2 mL) and

stirred at 60 °C for 3 h. The reaction mixture was concentrated in vacuo and purified by flash chromatography on silica gel, eluting with petroleum ether/ethyl acetate (15:1) to give desired product **72**.

5. Synthetic applications

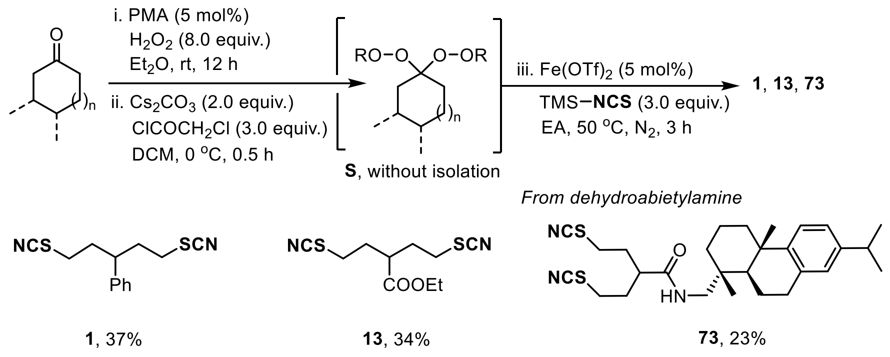

Step 1: To a 10 mL reaction tube was added a solution of H_2O_2 (30 wt% in H_2O, 8.0 equiv.), and PMA (5 mol%). Then a solution of cyclanone (0.4 mmol, 1.0 equiv.) in Et_2O (0.5 M) was added dropwise. The reaction mixture was stirred vigorously at room temperature for 12 h. The aqueous layer was extracted with DCM (3 × 10 mL). The combined organic layer was washed with brine, dried over Na_2SO_4 and concentrated to afford residue (the temperature should not exceed 30 °C).

Step 2: To a 10 mL reaction tube was added a solution of residue (*gem*-dihydroperoxide) in DCM (0.1 M). The mixture was stirred at 0 °C for 5 min, then Cs_2CO_3 (0.8 mmol, 2.0 equiv.) and chloroacetyl chloride (1.2 mmol, 3.0 equiv.) were added to the mixture dropwise. The mixture was stirred for 0.5 h. After the reaction was completed, the mixture was filtered and residue was rinsed with DCM (5–10 mL). The filtrate was concentrated to afford residue *gem*-diperoxides **S** (the temperature should not exceed 30 °C).

Step 3: A 10 mL oven-dried Schlenk-tube equipped with a magnetic stirrer was charged with $Fe(OTf)_2$ (0.02 mmol, 5 mol%). Then, the tube was evacuated and backfilled with nitrogen (three times). Subsequently, a solution of *gem*-diperoxides **S** and TMSNCS (1.2 mmol, 3.0 equiv.) in EA (4.0 mL) was added by a syringe. The reaction mixture was stirred at 50 °C for 3 h. After that, the reaction mixture was concentrated in vacuo and purified by flash chromatography on silica gel (petroleum ether/ethyl acetate = 10:1 to 5:1) to give products **1** (colorless oil, 37% yield, 38.7 mg), **13** (colorless oil, 34% yield, 35.2 mg) and **73** (colorless oil, 23% yield, 45.6 mg).

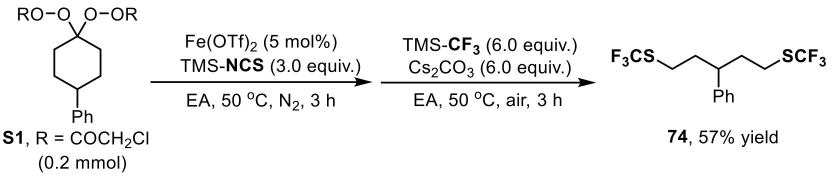

Step 1: A 10 mL oven-dried Schlenk-tube equipped with a magnetic stirrer was charged with $Fe(OTf)_2$ (0.01 mmol, 5 mol%). Then, the tube was evacuated and backfilled with nitrogen (three times). Subsequently, a solution of *gem*-diperoxides **S1** (0.2 mmol, 1.0 equiv.) and TMSNCS (0.60 mmol, 3.0 equiv.) in EA (2.0 mL) were added by a syringe and stirred at 50 °C for 3 h.

Step 2: Then Cs_2CO_3 (1.2 mmol, 6.0 equiv.) and $TMSCF_3$ (1.2 mmol, 6.0 equiv.) were added and the mixture was then stirred at 50 °C for 24 h in air. The reaction mixture was concentrated in vacuo and purified by flash chromatography on silica gel, eluting with petroleum ether/dichloromethane (5:1) to yield the title compound **74** (colorless oil, 57% yield, 39.8 mg).

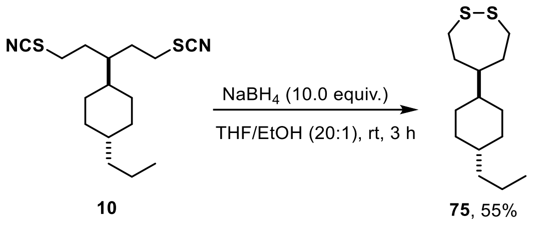

To a 10 mL oven-dried Schlenk-tube equipped with a magnetic stirrer, a solution of compound **10** (0.20 mmol, 1.0 equiv.) in THF/EtOH (2 mL, 20:1, v/v) was added. Subsequently, $NaBH_4$ (2.0 mmol, 10.0 equiv.) was added slowly and the mixture was stirred at room temperature for 3 h. The reaction mixture was concentrated in vacuo and purified by flash chromatography on silica gel (petroleum ether: ethyl acetate = 40:1) yielding the title compound **75** (colorless oil, 55% yield, 28.6 mg).

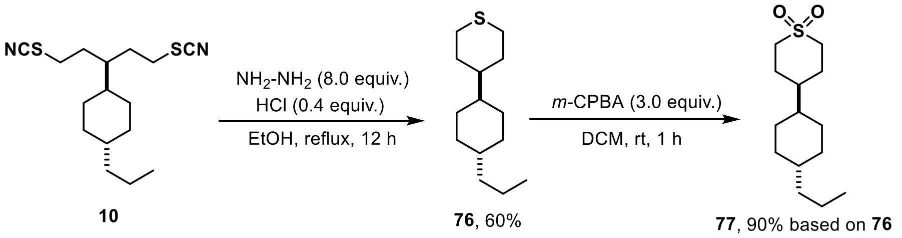

Step 1: To a 10 mL oven-dried Schlenk-tube equipped with a magnetic stirrer, a solution of compound **10** (0.20 mmol, 1.0 equiv.) in EtOH (2 mL) was added. Subsequently, NH_2-NH_2 (1.6 mmol, 8.0 equiv., 1.0 M in EtOH) along with HCl (0.4 equiv.) was added slowly. The reaction mixture was stirred at 78 °C for 12 h. After the reaction was completed, the reaction mixture was concentrated in vacuo and purified by

flash chromatography on silica gel (petroleum ether/ethyl acetate = 40:1) to yield the title compound **76** (colorless oil, 60% yield, 27.2 mg).

Step 2: To a 10 mL oven-dried Schlenk-tube equipped with a magnetic stirrer, a solution of **76** (0.12 mmol, 1.0 equiv.) in DCM (2 mL) was added. Subsequently, *m*-CPBA (3.0 equiv.) was added and stirred at room temperature for 3 h. The reaction mixture was concentrated in vacuo and purified by flash chromatography on silica gel (petroleum ether/ethyl acetate = 5:1) to yield the title compound **77** (white solid, 28.1 mg) in 90% yield based on **76**.

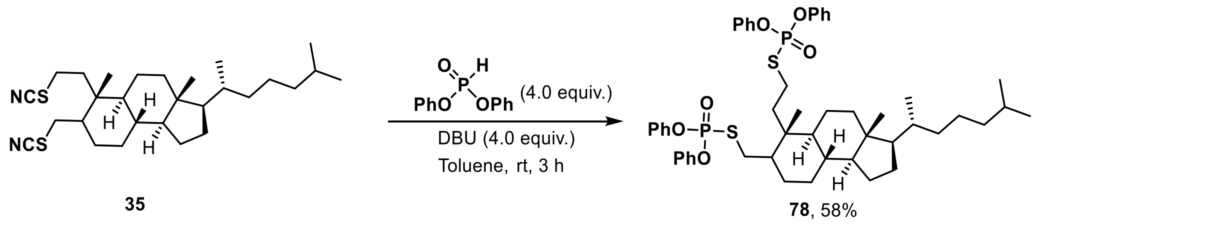

To a 10 mL oven-dried Schlenk-tube equipped with a magnetic stirrer, a solution of compound **35** (0.30 mmol, 1.0 equiv.) in toluene (3 mL) was added. Subsequently, DBU (1.2 mmol, 4.0 equiv.) and diphenyl phosphite (1.2 mmol, 4.0 equiv.) were added slowly. The reaction mixture was stirred at room temperature for 3 h. After the reaction was completed, the reaction mixture was concentrated in vacuo and purified by flash chromatography on silica gel (petroleum ether/ethyl acetate = 20:1 to 10:1) to yield the title compound **78** (colorless oil, 58% yield, 158.2 mg).

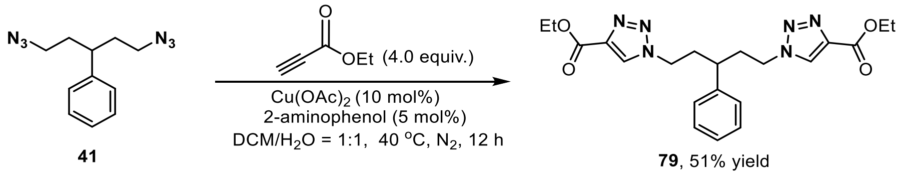

A 10 mL oven-dried Schlenk-tube equipped with a magnetic stirrer was charged with $Cu(OAc)_2$ (0.02 mmol, 10 mol%) and 2-aminophenol (0.01 mmol, 5 mol%). Then, the tube was evacuated and backfilled with nitrogen (three times). Subsequently, a solution of compound **41** (0.20 mmol, 1.0 equiv.) and alkyne (0.80 mmol, 4.0 equiv.) in DCM (1 mL) together with H_2O (1 mL) was added by a syringe, and stirred at 40 °C for 12 h. After the reaction was completed, the reaction mixture was concentrated in vacuo and purified by normal phase column chromatography (petroleum ether/ethyl acetate = 4:1 to 1:1) to yield the title compound **79** (yellow oil, 51% yield, 43.5 mg).

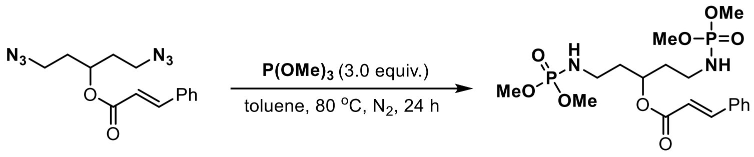

45

80, 70% yield

To a 10 mL oven-dried Schlenk-tube equipped with a magnetic stirrer and backfilled with nitrogen (three times). Subsequently, a solution of compound **45** (0.20 mmol, 1.0 equiv.) and $P(OMe)_3$ (0.60 mmol, 3.0 equiv.) in toluene (1 mL) was added by a syringe and stirred at 80 °C for 24 h. The reaction mixture was concentrated in vacuo and purified by phase column chromatography (petroleum ether/ethyl acetate = 4:1 to 1:1) to yield the title compound **80** (yellow oil, 70% yield, 64.9 mg).

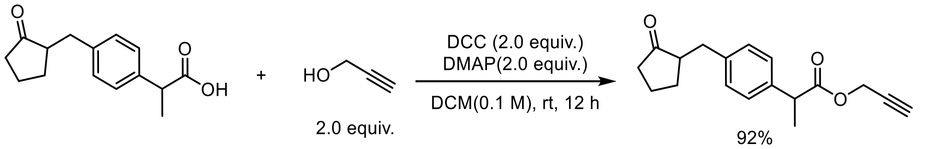

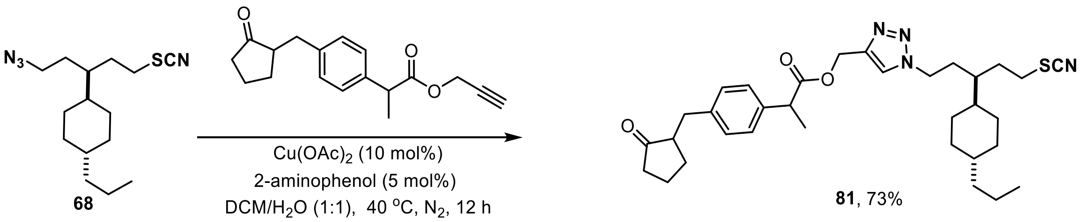

68

81, 73%

Step 1: To a 100 mL reaction tube was added loxoprofen (5.0 mmol, 1.0 equiv.), DCC (10.0 mmol, 2.0 equiv.) and DMAP (10.0 mmol, 2.0 equiv.). Then added a solution of propargyl alcohol (10.0 mmol, 2.0 equiv.) in DCM (0.1 M) was added dropwise. The reaction mixture was stirred vigorously at room temperature for 12 h and detected by TLC. The combined organic layer was washed with brine, dried over Na_2SO_4 and concentrated to afford a residue, which was purified by column chromatography on silica gel (petroleum ether/ethyl acetate = 10:1) to give the target product with a yield of 92%.

Step 2: A 10 mL oven-dried Schlenk-tube equipped with a magnetic stirrer was charged with $Cu(OAc)_2$ (0.02 mmol, 10 mol%) and 2-aminophenol (0.01 mmol, 5 mol%). Then, the tube was evacuated and backfilled with nitrogen (three times). Subsequently, a solution of compound **68** (0.20 mmol, 1.0 equiv.) and alkyne (0.40 mmol, 2.0 equiv.) in DCM (1 mL) along with H_2O (1 mL) was added by a syringe. The reaction mixture was stirred at 40 °C for 12 h. After that, the reaction mixture was extracted with ethyl acetate (5 mL × 3), concentrated in vacuo and purified by flash chromatography on silica gel (petroleum ether/ethyl acetate = 4:1 to 2:1) to give the desired product **81** (colorless oil, 73% yield, 84.5 mg).

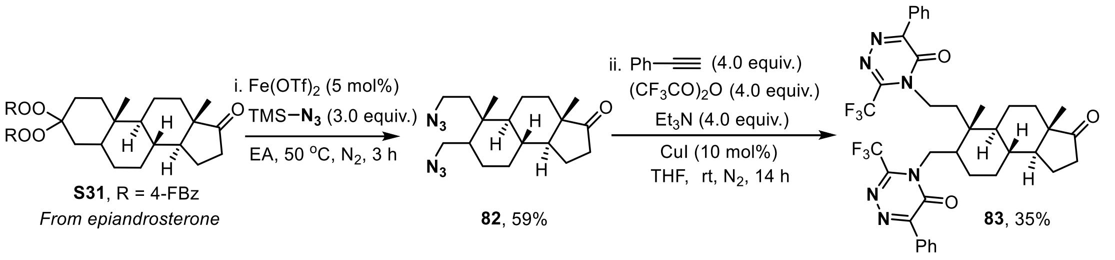

Step 1: A 10 mL oven-dried Schlenk-tube equipped with a magnetic stirrer was charged with $Fe(OTf)_2$ (0.01 mmol, 5 mol%). Then, the tube was evacuated and backfilled with nitrogen (three times). Subsequently, a solution of **S31** (0.2 mmol, 1.0 equiv.) and $TMSN_3$ (0.6 mmol, 3.0 equiv.) in EA (2.0 mL) was added by a syringe. The reaction mixture was stirred at 50 °C for 3 h. After that, the reaction mixture was concentrated in vacuo and purified by flash chromatography on silica gel (petroleum ether/ethyl acetate = 20:1 to 10:1) to give product **82** (colorless oil, 59% yield, 40.5 mg).

Step 2: In a glove box filled with nitrogen, to an oven-dried 5 mL pressure tube equipped with a stirring bar were added CuI (10 mol %), ethynylbenzene (0.48 mmol, 4.0 equiv), azide compound **82** (40.5 mg, 0.12 mmol), $(CF_3CO)_2O$ (0.48 mmol, 4.0 equiv), Et_3N (0.48 mmol, 4.0 equiv) and THF (1.0 mL). The tube was stirred at ambient temperature for 14 h. The residue was purified by flash chromatography (petroleum ether/ethyl acetate = 4:1) to obtain the product **83** (colorless oil, 35% yield, 30.6 mg).

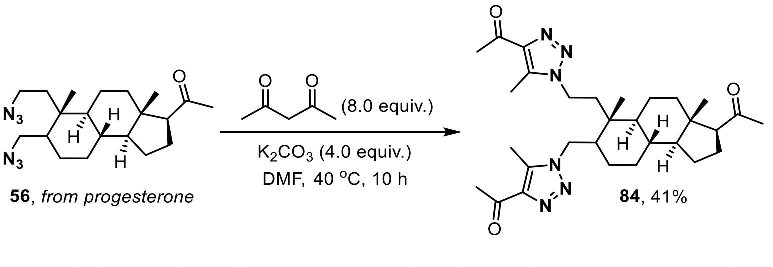

To a DMF (2.0 mL) solution of the azide **56** (0.2 mmol) and acetylacetone (1.6 mmol) was added K_2CO_3 (0.8 mmol), and the reaction mixture was stirred at 40 °C for 10 h. The reaction mixture was quenched with water, and then extracted twice with ethyl acetate. The combined organic extracts were washed with water and brine, dried over $MgSO_4$. The solvent was evaporated and the residue was purified by flash chromatography on silica gel using petroleum ether and ethyl acetate (5:1 to 2:1) to give the corresponding product **84** (colorless oil, 41% yield, 43.9 mg).

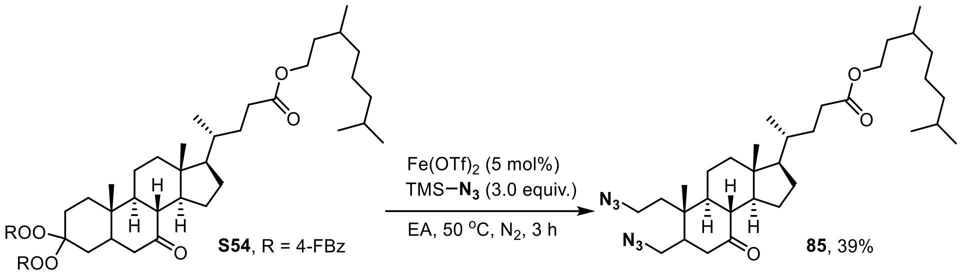

A 10 mL oven-dried Schlenk-tube equipped with a magnetic stirrer was charged with $Fe(OTf)_2$ (0.01 mmol, 5 mol%). Then, the tube was evacuated and backfilled with nitrogen (three times). Subsequently, a solution of **S54** (0.2 mmol, 1.0 equiv.) and $TMSN_3$ (0.6 mmol, 3.0 equiv.) in EA (2.0 mL) was added by a syringe. The reaction mixture was stirred at 50 °C for 3 h. After that, the reaction mixture was concentrated in vacuo and purified by flash chromatography on silica gel using petroleum ether and ethyl acetate (20:1 to 10:1) to give products **85** (colorless oil, 39% yield, 45.5 mg).

6. Scale-up synthesis

6.1 Large-scale synthesis of dithiocyanation

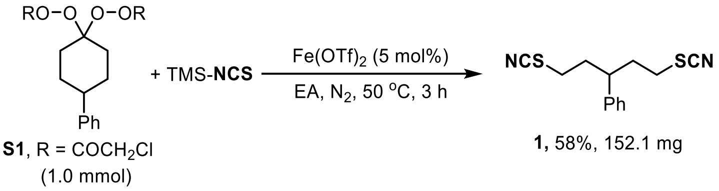

A 50 mL oven-dried Schlenk-tube equipped with a magnetic stirrer was charged with $Fe(OTf)_2$ (0.05 mmol, 5 mol%). Then, the tube was evacuated and backfilled with nitrogen (three times). Subsequently, a solution of *gem*-diperoxide **S1** (1.0 mmol, 1.0 equiv.) and TMSNCS (3.0 mmol, 3.0 equiv.) in EA (10.0 mL) was added by a syringe. The reaction mixture was stirred at 50 °C for 3 h. After that, the reaction mixture was concentrated in vacuo and purified by flash chromatography on silica gel (petroleum ether/ethyl acetate = 5:1). The desired product **1** was obtained (58% yield, 152.1 mg).

6.2 Large-scale synthesis of diazidation

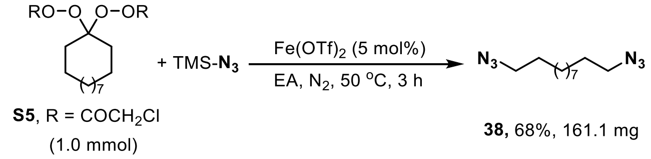

A 50 mL oven-dried Schlenk-tube equipped with a magnetic stirrer was charged with $Fe(OTf)_2$ (0.05 mmol, 5 mol%). Then, the tube was evacuated and backfilled with nitrogen (three times). Subsequently, a solution of *gem*-diperoxide **S5** (1.0 mmol, 1.0 equiv.) and $TMSN_3$ (3.0 mmol, 3.0 equiv.) in EA (10.0

mL) was added by a syringe. The reaction mixture was stirred at 50 °C for 3 h. After that, the reaction mixture was concentrated in vacuo and purified by flash chromatography on silica gel (petroleum ether/dichloromethane = 5:1). The desired product **38** was obtained (68% yield, 161.1 mg).

7. Mechanistic investigation

7.1 Radical inhibiting experiment

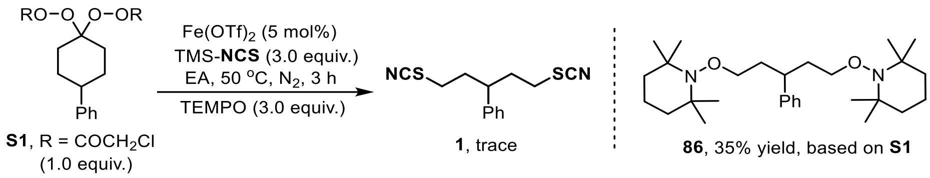

a) A 10 mL oven-dried Schlenk-tube equipped with a magnetic stirrer was charged with $Fe(OTf)_2$ (0.02 mmol, 5 mol%) and TEMPO (1.2 mmol, 3.0 equiv.). Then, the tube was evacuated and backfilled with nitrogen (three times). Subsequently, a solution of **S1** (0.40 mmol, 1.0 equiv.) and TMSNCS (1.2 mmol, 3.0 equiv.) in EA (4.0 mL) was added by a syringe. The reaction mixture was stirred at 50 °C for 3 h.

When TEMPO was added into the reaction of **S1** with TMSNCS under the standard conditions, only trace amount of **1** was detected, along with the TEMPO adduct **86** isolated in 35% yield. This result indicates that a diradical pathway might be involved in this transformation.

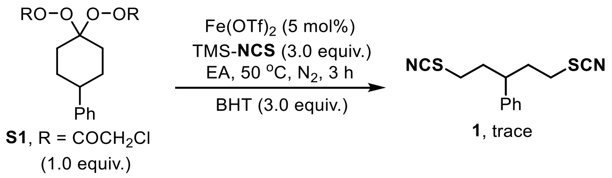

b) A 10 mL oven-dried Schlenk-tube equipped with a magnetic stirrer was charged with $Fe(OTf)_2$ (0.02 mmol, 5 mol%) and BHT (1.2 mmol, 3.0 equiv.). Then, the tube was evacuated and backfilled with nitrogen (three times). Subsequently, a solution of **S1** (0.40 mmol, 1.0 equiv.) and TMSNCS (1.20 mmol, 3.0 equiv.) in EA (4.0 mL) was added by a syringe. The reaction mixture was stirred at 50 °C for 3 h.

When BHT was added into the reaction of **S1** with TMSNCS under the standard conditions, only a trace amount of compound **1** was detected. This result indicates that a diradical pathway might be involved in this transformation.

7.2 Detection of byproducts

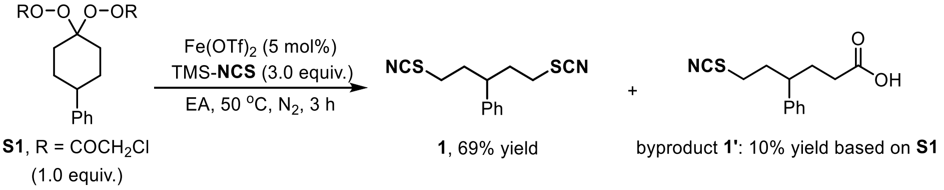

A 10 mL oven-dried Schlenk-tube equipped with a magnetic stirrer was charged with $Fe(OTf)_2$ (0.01 mmol, 5 mol%). Then, the tube was evacuated and backfilled with nitrogen (three times). Subsequently,

a solution of **S1** (0.20 mmol, 1.0 equiv.) and TMSNCS (0.60 mmol, 3.0 equiv.) in EA (2.0 mL) was added by a syringe. The reaction mixture was stirred at 50 °C for 3 h. Then, the reaction mixture was concentrated in vacuo and purified by flash chromatography on silica gel. The desired product **1** was obtained in 69% yield, along with byproduct **1'** in 10% yield.

1' was detected with the yield of 10%. These results indicate that peroxycarboxylic esters may be involved in this reaction.

7.3 Detection of intermediate

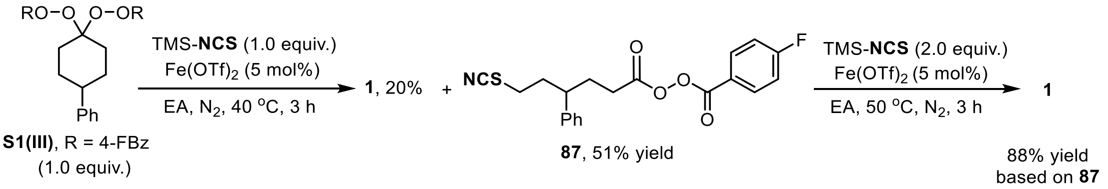

Step 1: A 10 mL oven-dried Schlenk-tube equipped with a magnetic stirrer was charged with $Fe(OTf)_2$ (3.5 mg, 5 mol%). Then, the tube was evacuated and backfilled with nitrogen (three times). Subsequently, a solution of *gem*-diperoxide **S1(III)** (0.20 mmol, 1.0 equiv.) and TMSNCS (0.20 mmol, 1.0 equiv.) in EA (2.0 mL) was added by a syringe. The reaction mixture was stirred at 40 °C for 12 h. Then, the reaction mixture was concentrated in vacuo (the temperature of concentration should not exceed 30 °C) and purified by flash chromatography on silica gel (petroleum ether/ ethyl acetate = 10:1). The desired product **87** was obtained in 51% yield (colorless oil, 39.5 mg).

Step 2: A 10 mL oven-dried Schlenk-tube equipped with a magnetic stirrer was charged with $Fe(OTf)_2$ (1.7 mg, 5 mol%). Then, the tube was evacuated and backfilled with nitrogen (three times). Subsequently, a solution of **87** (0.10 mmol, 39.5 mg, 1.0 equiv.) and TMSNCS (0.20 mmol, 2.0 equiv.) in EA (1.0 mL) was added by a syringe. The reaction mixture was stirred at 50 °C for 3 h and concentrated in vacuo and purified by flash chromatography on silica gel (petroleum ether/ ethyl acetate = 5:1). The desired product **1** was obtained (88% yield based on **87**, 23.8 mg).

These results indicated that a radical intermediate **87** might be as the intermediate involved in this transformation.

7.4 Cyclic voltammetry data

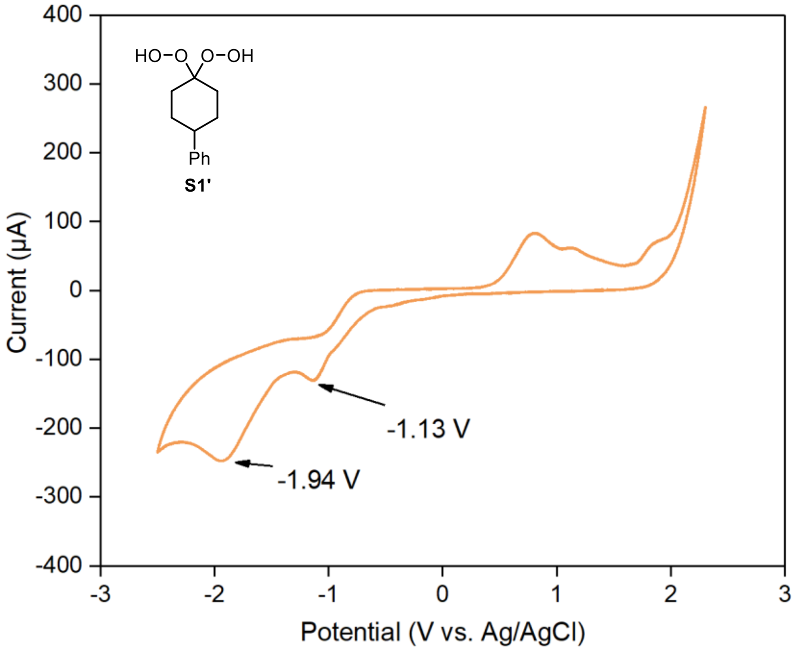

Supplementary Fig. 1|Cyclic voltammogram of **S1'** (0.2 mmol) in a 0.05 M solution of NBu_4PF_6 in anhydrous MeCN (4 mL) at a scan rate of 100 mV.s^{-1}.

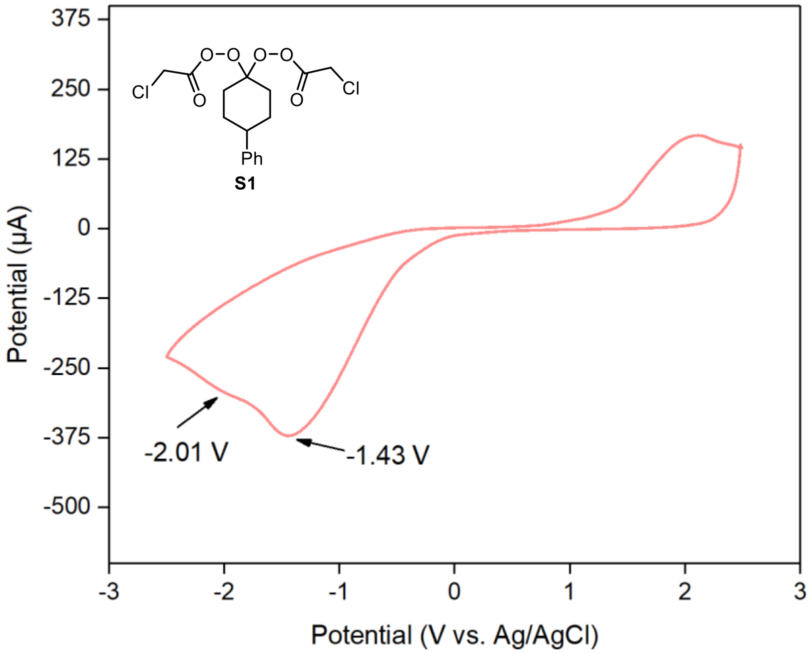

Supplementary Fig. 2| Cyclic voltammogram of **S1** (0.2 mmol) in a 0.05 M solution of NBu_4PF_6 in anhydrous MeCN (4 mL) at a scan rate of 100 mV.s^{-1}.

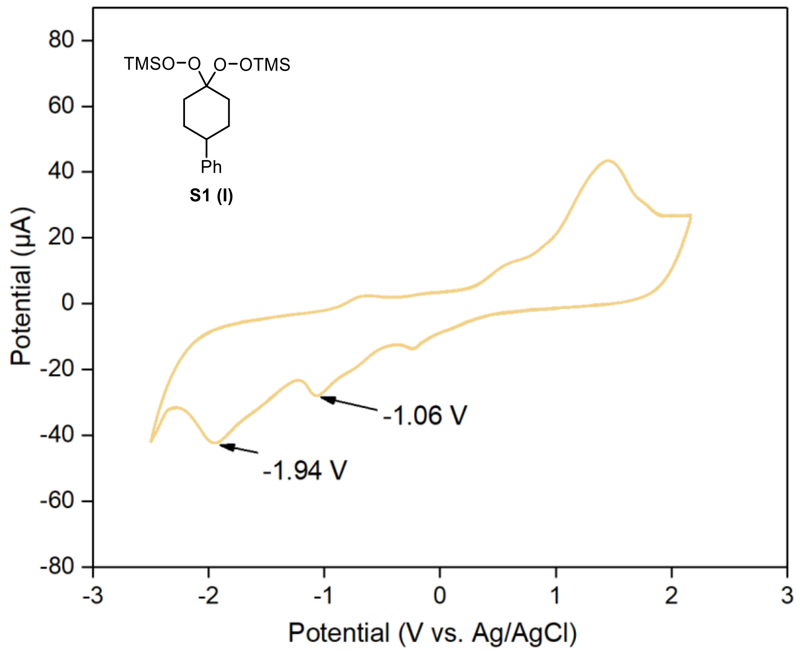

Supplementary Fig. 3| Cyclic voltammogram of **S1 (I)** (0.2 mmol) in a 0.05 M solution of NBu_4PF_6 in anhydrous MeCN (4 mL) at a scan rate of 100 mV.s^{-1}.

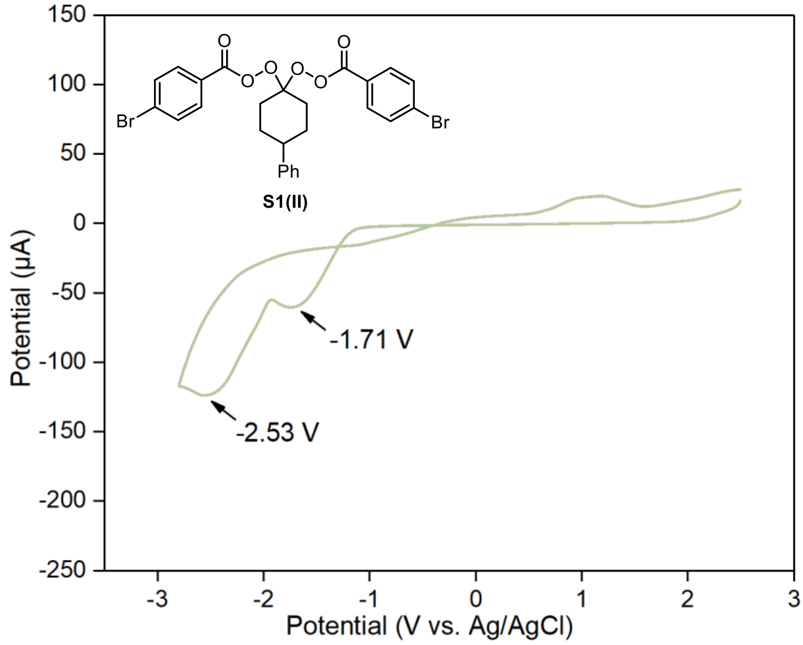

Supplementary Fig. 4| Cyclic voltammogram of **S1 (II)** (0.2 mmol) in a 0.05 M solution of NBu_4PF_6 in anhydrous MeCN (4 mL) at a scan rate of 100 mV.s^{-1}.

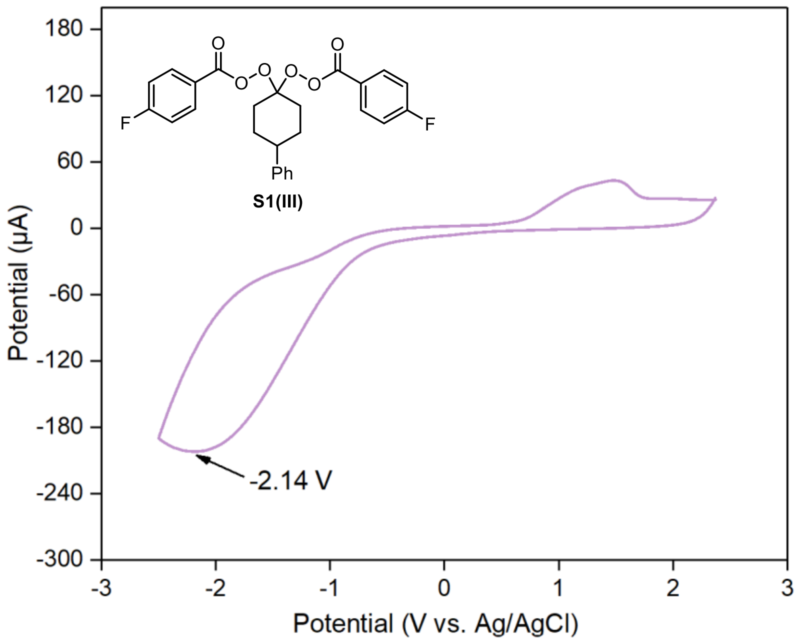

Supplementary Fig. 5| Cyclic voltammogram of **S1 (III)** (0.2 mmol) in a 0.05 M solution of NBu_4PF_6 in anhydrous MeCN (4 mL) at a scan rate of 100 mV.s^{-1}.

7.5 Simultaneous thermal analysis (DSC-TGA) of *gem*-diperoxides

Simultaneous Thermal Analysis (DSC-TGA) refers to the simultaneous application of Differential Scanning Calorimetry (DSC) and Thermogravimetry (TGA) to one and the same sample in a single instrument. Here, we measured *gem*-diperoxides under nitrogen. The measurement groups used aluminum crucibles with pierced lids (open crucibles) as a container.

Gas flow velocity: 100.0 mL/min

Heating region: from 35 ºC to 800 ºC

Heating rate: 10 K/min

Gem-dihydroperoxides S1' date

Gem-dihydroperoxides **S1'** (white solid, 4.3230 mg) was placed in an Al-crucible for the DSC-TGA measurement. TGA analysis established the onset of thermal decomposition at 135 °C. This decomposition event is directly associated with the broad exothermic peak observed in the DSC curve between 128 °C and 179 °C.

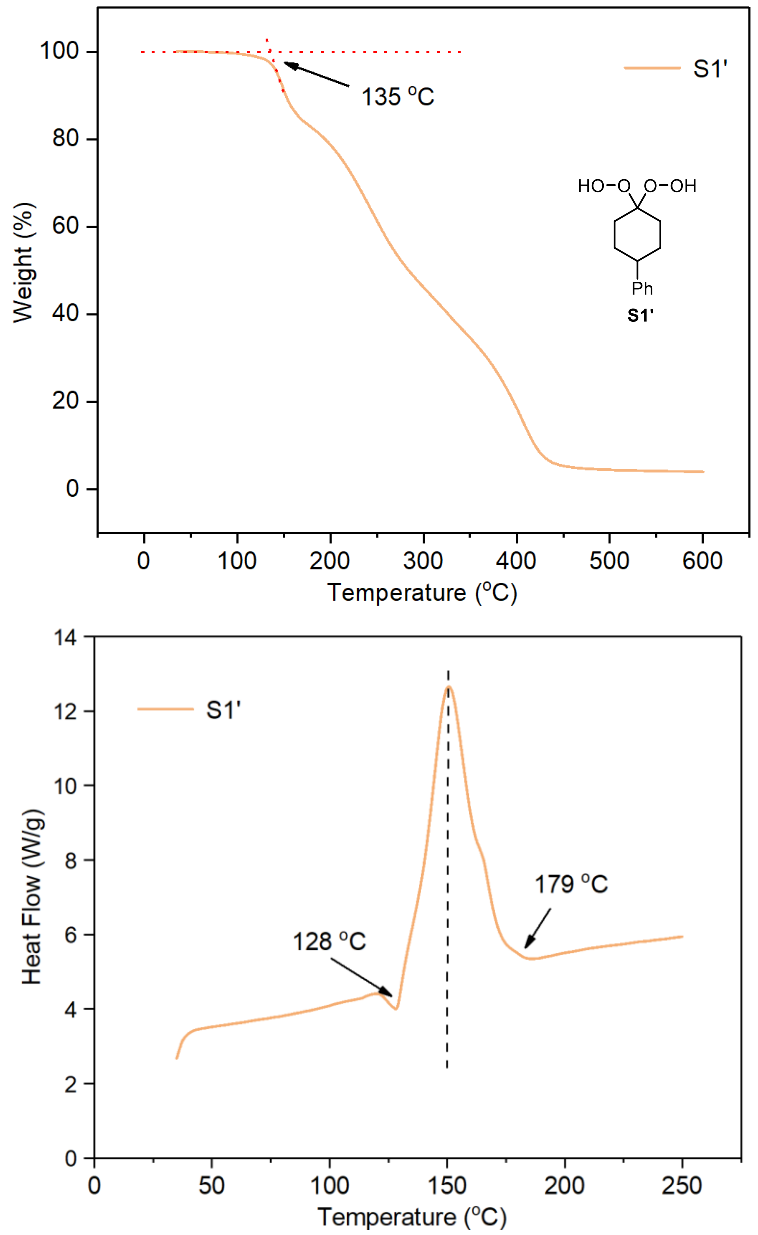

Supplementary Fig. 6| Simultaneous thermal analysis of **S1'**.

Gem-diperoxides S1 date

Gem-dihydroperoxides **S1** (colorless oil, 3.5920 mg) was placed in an Al-crucible for the DSC-TGA measurement. TGA analysis established the onset of thermal decomposition at 93 °C. This decomposition event is directly associated with the broad exothermic peak observed in the DSC curve between 83 °C and 130 °C.

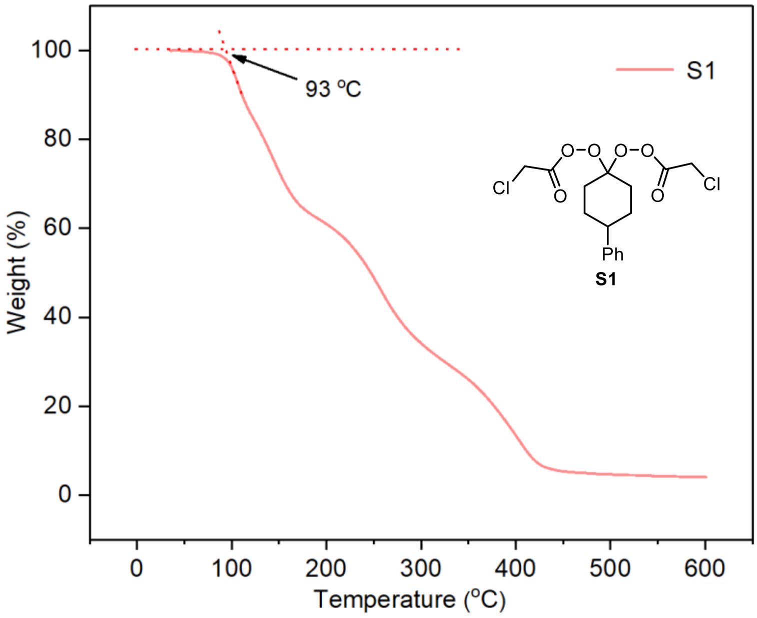

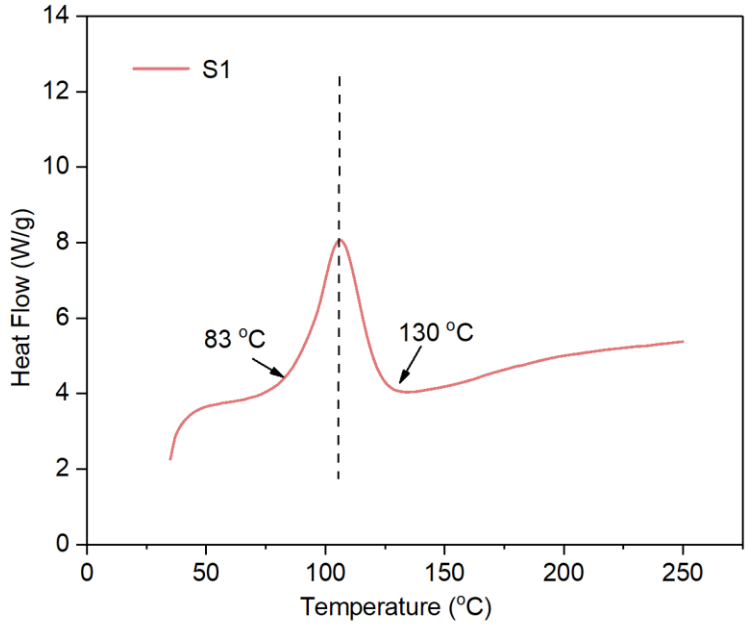

Supplementary Fig. 7| Simultaneous thermal analysis of **S1**.

Gem-diperoxides S1 (I) date

Gem-dihydroperoxides **S1 (I)** (colorless oil, 3.1470 mg) was placed in an Al-crucible for the DSC-TGA measurement. TGA analysis established the onset of thermal decomposition at 129 °C. This decomposition event is directly associated with the broad exothermic peak observed in the DSC curve between 129 °C and 236 °C.

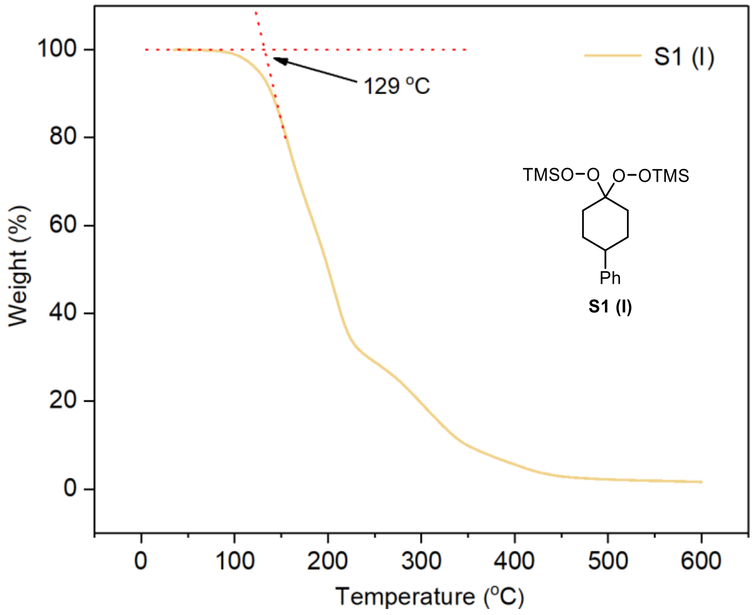

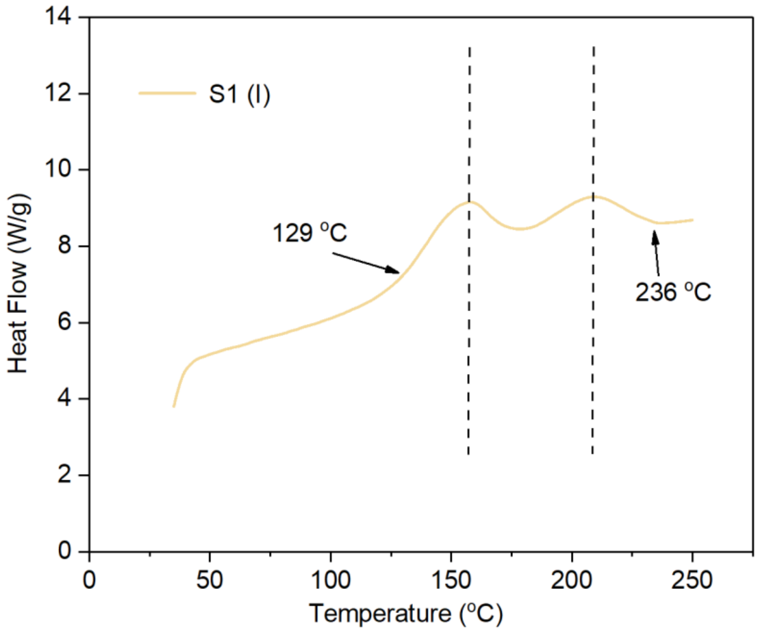

Supplementary Fig. 8| Simultaneous thermal analysis of **S1 (I)**.

Gem-diperoxides S1 (II) date

Gem-dihydroperoxides **S1 (II)** (colorless oil, 3.3630 mg) was placed in an Al-crucible for the DSC-TGA measurement. TGA analysis established the onset of thermal decomposition at 112 °C. This decomposition event is directly associated with the broad exothermic peak observed in the DSC curve between 108 °C and 158 °C.

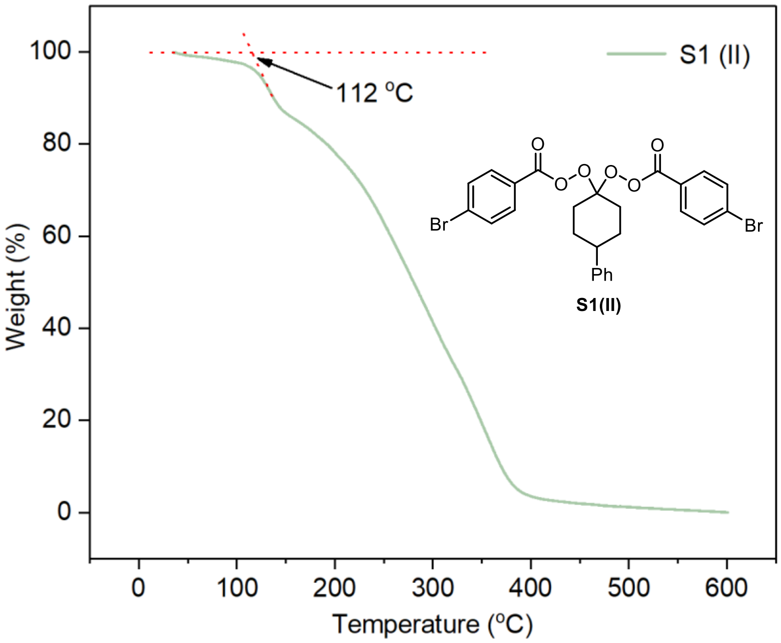

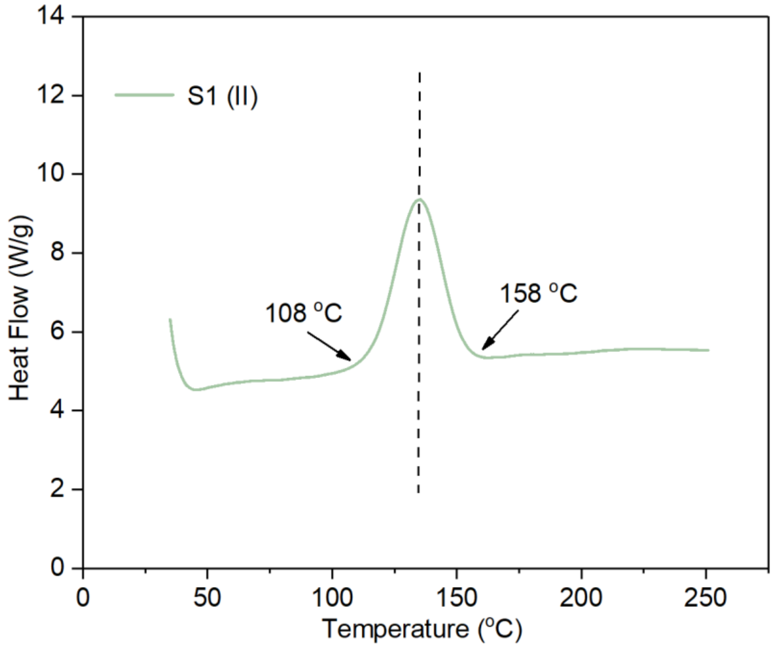

Supplementary Fig. 9| Simultaneous thermal analysis of **S1 (II)**.

Gem-diperoxides S1 (III) date

Gem-dihydroperoxides **S1 (III)** (colorless oil, 4.2630 mg) was placed in an Al-crucible for the DSC-TGA measurement. TGA analysis established the onset of thermal decomposition at 117 °C. This decomposition event is directly associated with the broad exothermic peak observed in the DSC curve between 108 °C and 150 °C.

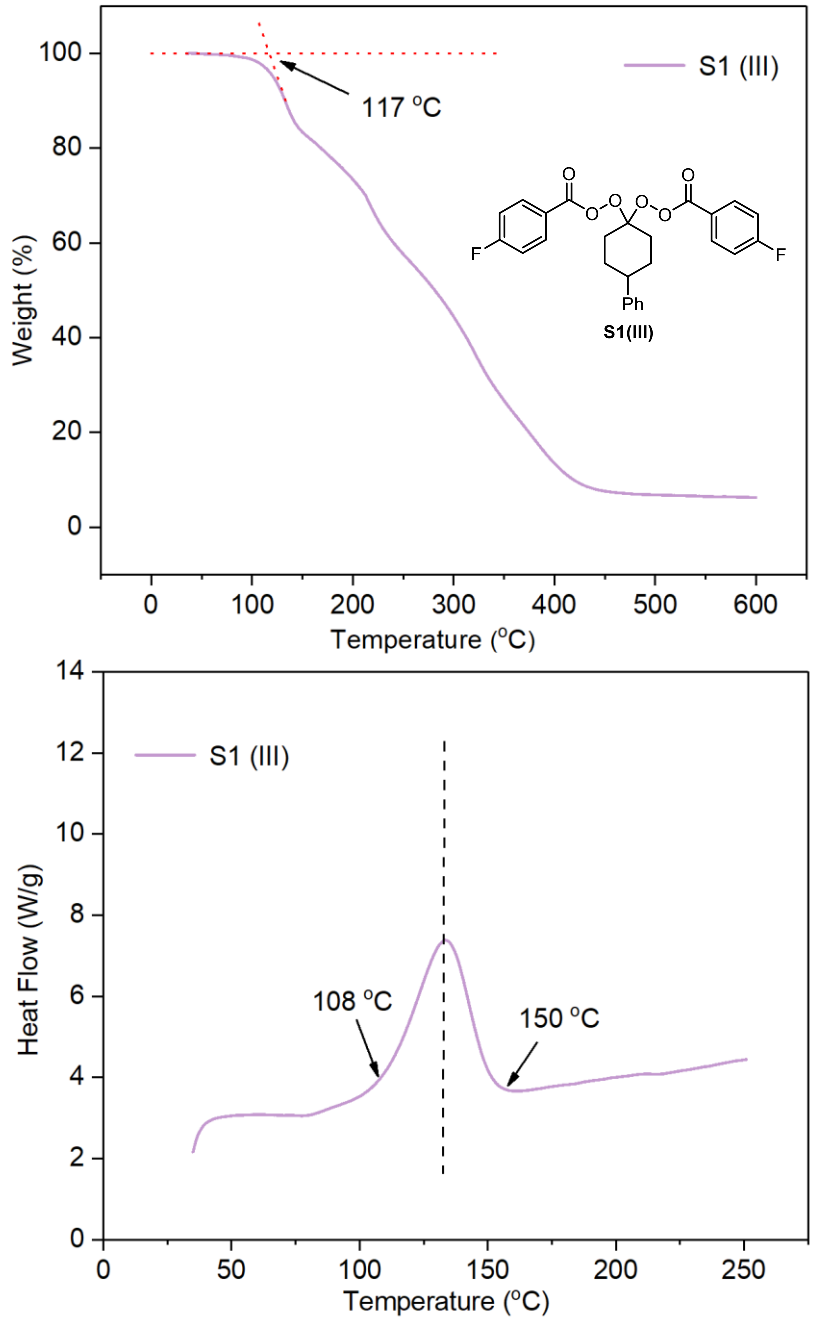

Supplementary Fig. 10| Simultaneous thermal analysis of **S1 (III)**.

8. Investigation of reactivity of *α*-substituted ketones

8.1 Oxidation of progesterone

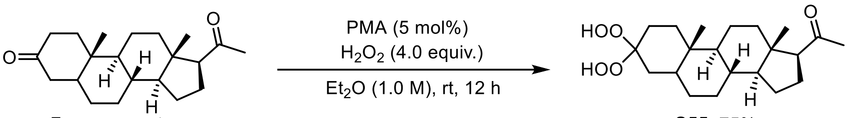

From progesterone **S55**, 75%

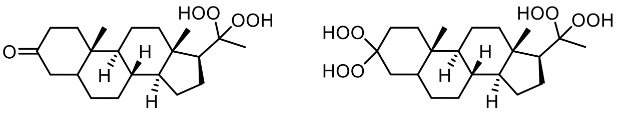

S55-1, ND **S55-2**, ND

To a 50 mL reaction tube was added a solution of H_2O_2 (30 wt% in H_2O, 4.0 equiv.) and PMA (5 mol%). Then a solution of ketone (2.0 mmol, 1.0 equiv.) in Et_2O (1.0 M) was added dropwise. The reaction mixture was stirred vigorously at room temperature for 12 h. The aqueous layer was extracted with DCM (3 × 30 mL). The combined organic layer was washed with brine, dried over Na_2SO_4 and concentrated to afford residue (the temperature should not exceed 30 °C), which was purified by column chromatography on silica gel (petroleum ether/ethyl acetate = 5:1) to give *gem*-dihydroperoxides **S55** (colorless oil, 75% yield, 0.54 g). Analysis of the product showed no evidence of the formation of **S55-1** or **S55-2**.

8.2 Oxidation of acyclic ketones and α-substituted ketones

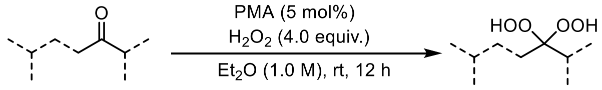

From vanillylacetone *From progesterone*

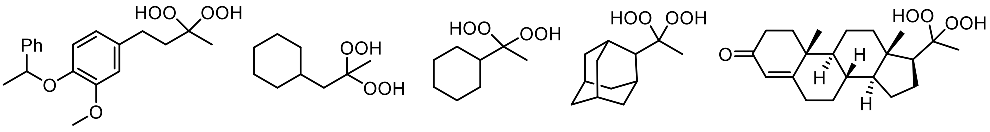

S56, 88% **S57**, 66% **S58**, 52% **S59**, trace **S60**, trace

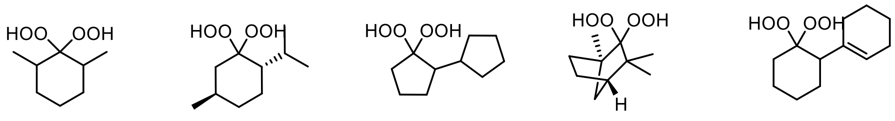

S61, ND **S62**, ND **S63**, ND **S64**, ND **S65**, ND

To a 50 mL reaction tube was added a solution of H_2O_2 (30% wt in H_2O, 4.0 equiv.), and PMA (5 mol%). Then a solution of ketone (2.0 mmol, 1.0 equiv.) in Et_2O (1.0 M) was added dropwise. The reaction mixture was stirred vigorously at room temperature for 12 h. The aqueous layer was extracted with DCM (3 × 30 mL). The combined organic layer was washed with brine, dried over Na_2SO_4 and concentrated to afford residue (the temperature should not exceed 30 °C), which was purified by column chromatography

on silica gel (petroleum ether/ethyl acetate = 10:1 to 3:1) to give *gem*-dihydroperoxides **S56** (colorless oil, 88% yield, 0.61 g), **S57** (colorless oil, 66% yield, 0.25 g), **S58** (colorless oil, 52% yield, 0.18 g), **S59** (trace), **S60** (trace), **S61-S65** (0 %)**.**

The results show that as the steric bulk increases, the yield of the corresponding *gem*-dihydroperoxide gradually decreases. In particular, substrates with substantial steric demand, such as the adamantyl-substituted ketone and progesterone itself, failed to yield any desired oxidation product. For α-substituted cyclic ketones, the observed poor reactivity can be attributed to steric factor.

9. Experimental and characterization data

9.1 Characterization data of starting materials

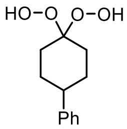

(4,4-Dihydroperoxycyclohexyl)benzene (S1') Prepared according to general procedure **A1** from 4-phenylcyclohexanone (10.0 mmol). After purification by flash column chromatography using a gradient eluent of petroleum ether/ethyl acetate (10:1 to 5:1), the title compound was isolated as a white solid (52% yield, 1.15 g); R_f= 0.3 (PE: EA = 5:1); ^{1}H NMR (400 MHz, $CDCl_3$) δ 8.66 (bs, 2H), 7.31-7.26 (m, 2H), 7.22-7.18 (m, 3H), 2.63-2.58 (m, 1H), 2.43-2.38 (m, 2H), 1.90-1.64 (m, 6H); ^{13}C NMR (100 MHz, $CDCl_3$) δ 145.8, 128.4, 126.8, 126.3, 110.2, 43.5, 29.9, 29.6 ppm; HRMS (ESI-TOF) *m/z* calcd. for $C_{12}H_{16}O_4Na^+$ (M+Na^+) 247.0941, found 247.0951.

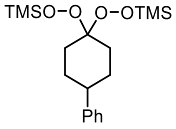

4-Phenyl-1,1-bis[(trimethylsilyl)dioxidanyl]cyclohexane (S1(I)) Prepared according to general procedure **A1** and **B3** from 4-phenylcyclohexanone (10.0 mmol). After purification by flash column chromatography using a gradient eluent of petroleum ether/dichloromethane (10:1 to 5:1), the title compound was isolated as a colorless oil (30% yield, 1.11 g, two steps from ketone); R_f= 0.9 (PE: DCM = 5:1); ^{1}H NMR (400 MHz, $CDCl_3$) δ 7.32-7.28 (m, 2H), 7.25-7.18 (m, 3H), 2.59-2.53 (m, 1H), 2.44-2.39 (m, 2H), 1.82-1.66 (m, 4H), 1.62-1.52 (m, 2H), 0.28 (s, 9H), 0.23 (s, 9H); ^{13}C NMR (100 MHz, $CDCl_3$) δ 146.5, 128.4, 126.8, 126.1, 109.0, 43.7, 30.4, 30.2, -1.0, -1.1 ppm; HRMS (ESI-TOF) *m/z* calcd. for $C_{18}H_{32}O_4Si_2Na^+$ (M+Na^+) 391.1731, found 391.1741.

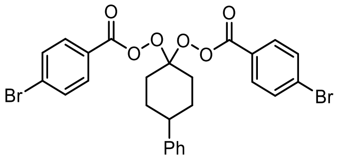

4-Phenylcyclohexane-1,1-diyl bis(4-bromobenzoperoxoate) (S1(II)) Prepared according to general procedure **A1** and **B2** from 4-phenylcyclohexanone (10.0 mmol). After purification by flash column chromatography using petroleum ether/dichloromethane (1:1), the title compound was isolated as a white solid (34% yield, 2.00 g, two steps from ketone); R_f= 0.4 (PE: DCM = 1:1); ^{1}H NMR (400 MHz, $CDCl_3$)

δ 7.85-7.83 (m, 4H), 7.62-7.58 (m, 4H), 7.34-7.20 (m, 5H), 2.73-2.66 (m, 1H), 2.61-2.57 (m, 2H), 2.06-1.93 (m, 6H); ^{13}C NMR (100 MHz, $CDCl_3$) δ 163.1, 162.9, 145.1, 132.0, 130.74, 130.70, 128.8, 128.4, 126.7, 126.4, 125.8, 125.7, 112.1, 43.0, 30.1, 29.6 ppm; HRMS (ESI-TOF) *m*/*z* calcd. for $C_{26}H_{22}Br_2O_6Na^+$ (M+Na$^+$) 610.9675, found 610.9688.

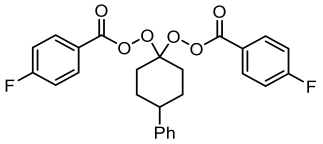

4-Phenylcyclohexane-1,1-diyl bis(4-fluorobenzoperoxoate) (S1(III)) Prepared according to general procedure **A1** and **B2** from 4-phenylcyclohexanone (10.0 mmol). After purification by flash column chromatography using petroleum ether/dichloromethane (1:1), the title compound was isolated as a white solid (36% yield, 1.71 g, two steps from ketone); R_f= 0.4 (PE: DCM = 1:1); ^{1}H NMR (400 MHz, $CDCl_3$) δ 8.03-7.99 (m, 4H), 7.34-7.20 (m, 5H), 7.16-7.11 (m, 4H), 2.73-2.66 (m, 1H), 2.61-2.58 (m, 2H), 2.04-1.94 (m, 6H); ^{13}C NMR (100 MHz, $CDCl_3$) δ 166.0 (d, J = 255.3 Hz), 162.9, 162.7, 145.2, 132.0 (d, J = 9.6 Hz), 131.9 (d, J = 9.6 Hz), 128.5, 126.8, 126.4, 123.2, 123.1, 115.9 (d, J = 22.0 Hz), 112.1, 43.1, 30.2, 29.7 ppm; ^{19}F NMR (376 MHz, $CDCl_3$) δ -103.54, -103.56; HRMS (ESI-TOF) *m*/*z* calcd. for $C_{26}H_{22}F_2O_6Na^+$ (M+Na$^+$) 491.1277, found 491.1277.

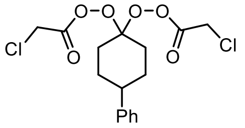

4-Phenylcyclohexane-1,1-diyl bis(2-chloroethaneperoxoate) (S1) Prepared according to general procedure **A1** and **B1** from 4-phenylcyclohexanone (10.0 mmol). After purification by flash column chromatography using petroleum ether/dichloromethane (1:1), the title compound was isolated as a colorless oil (41% yield, 1.54 g, two steps from ketone); R_f= 0.5 (PE: DCM = 1:1); ^{1}H NMR (400 MHz, $CDCl_3$) δ 7.34-7.28 (m, 2H), 7.25-7.19 (m, 3H), 4.12 (s, 2H), 4.10 (s, 2H), 2.66-2.59 (m, 1H), 2.51-2.41 (m, 2H), 1.93-1.75 (m, 6H); ^{13}C NMR (100 MHz, $CDCl_3$) δ 164.4, 164.3, 144.9, 128.5, 126.7, 126.5, 112.5, 42.9, 38.0, 37.9, 29.9, 29.5 ppm; HRMS (ESI-TOF) *m*/*z* calcd. for $C_{16}H_{18}Cl_2O_6Na^+$ (M+Na$^+$) 399.0373, found 399.0379.

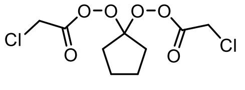

Cyclopentane-1,1-diyl bis(2-chloroethaneperoxoate) (S2) Prepared according to general procedure **A1** and **B1** from cyclopentanone (10.0 mmol). After purification by flash column chromatography using

petroleum ether/dichloromethane (1:1), the title compound was isolated as a colorless oil (50% yield, 1.45 g, two steps from ketone); R_f= 0.4 (PE: DCM = 1:1); ^{1}H NMR (400 MHz, $CDCl_3$) δ 4.09 (s, 4H), 2.14-2.10 (m, 4H), 1.86-1.83 (m, 4H); ^{13}C NMR (100 MHz, $CDCl_3$) δ 164.3, 123.3, 37.9, 33.7, 24.5 ppm; HRMS (ESI-TOF) *m/z* calcd. for $C_9H_{12}Cl_2O_6Na^+$ (M+Na$^+$) 308.9903, found 308.9917.

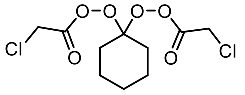

Cyclohexane-1,1-diyl bis(2-chloroethaneperoxoate) (S3) Prepared according to general procedure **A1** and **B1** from cyclohexanone (10.0 mmol). After purification by flash column chromatography using petroleum ether/dichloromethane (1:1), the title compound was isolated as a white solid (35% yield, 1.03 g, two steps from ketone); R_f= 0.4 (PE: DCM = 1:1); ^{1}H NMR (400 MHz, $CDCl_3$) δ 4.07 (s, 4H), 1.95-1.92 (m, 4H), 1.65-1.59 (m, 4H), 1.51-1.45 (m, 2H); ^{13}C NMR (100 MHz, $CDCl_3$) δ 164.3, 113.0, 37.9, 29.7, 24.7, 22.1 ppm; HRMS (ESI-TOF) *m/z* calcd. for $C_{10}H_{14}Cl_2O_6Na^+$ (M+Na$^+$) 323.0060, found 323.0050.

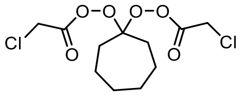

Cycloheptane-1,1-diyl bis(2-chloroethaneperoxoate) (S4) Prepared according to general procedure **A1** and **B1** from cycloheptanone (10.0 mmol). After purification by flash column chromatography using petroleum ether/dichloromethane (1:1), the title compound was isolated as a colorless oil (13% yield, 0.41 g, two steps from ketone); R_f= 0.5 (PE: DCM = 1:1); ^{1}H NMR (400 MHz, $CDCl_3$) δ 4.07 (s, 4H), 2.08-2.05 (m, 4H), 1.67-1.58 (m, 8H); ^{13}C NMR (100 MHz, $CDCl_3$) δ 164.3, 117.9, 38.0, 32.8, 29.7, 22.5 ppm; HRMS (ESI-TOF) *m/z* calcd. for $C_{11}H_{16}Cl_2O_6Na^+$ (M+Na$^+$) 337.0216, found 337.0222.

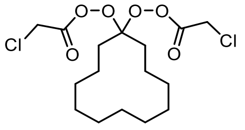

Cyclododecane-1,1-diyl bis(2-chloroethaneperoxoate) (S5) Prepared according to general procedure **A1** and **B1** from cyclododecanone (10.0 mmol). After purification by flash column chromatography using petroleum ether/dichloromethane (1:1), the title compound was isolated as a white solid (30% yield, 1.15 g, two steps from ketone); R_f= 0.5 (PE: DCM = 1:1); ^{1}H NMR (400 MHz, $CDCl_3$) δ 4.08 (s, 4H), 1.83-1.79 (m, 4H), 1.55-1.52 (m, 4H), 1.42-1.35 (m, 14H); ^{13}C NMR (100 MHz, $CDCl_3$) δ 164.3, 117.0, 38.0,

26.3, 25.9, 25.7, 22.1, 21.7, 19.2 ppm; HRMS (ESI-TOF) *m/z* calcd. for $C_{16}H_{26}Cl_2O_6Na^+$ ($M+Na^+$) 407.0999, found 407.1002.

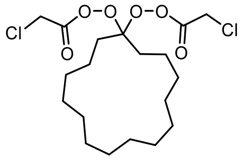

Cyclopentadecane-1,1-diyl bis(2-chloroethaneperoxoate) (S6) Prepared according to general procedure **A1** and **B1** from cyclopentadecanone (10.0 mmol). After purification by flash column chromatography using petroleum ether/dichloromethane (1:1), the title compound was isolated as a colorless oil (44% yield, 1.85 g, two steps from ketone); R_f= 0.6 (PE: DCM = 1:1); ^{1}H NMR (400 MHz, $CDCl_3$) δ 4.08 (s, 4H), 1.84-1.80 (m, 4H), 1.47-1.27 (m, 24H); ^{13}C NMR (100 MHz, $CDCl_3$) δ 164.3, 116.7, 38.0, 29.2, 27.2, 26.7, 26.4, 26.2, 21.5 ppm; HRMS (ESI-TOF) *m/z* calcd. for $C_{19}H_{32}Cl_2O_6Na^+$ ($M+Na^+$) 449.1468, found 449.1472.

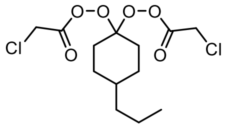

4-Propylcyclohexane-1,1-diyl bis(2-chloroethaneperoxoate) (S7) Prepared according to general procedure **A1** and **B1** from 4-propylcyclohexanone (10.0 mmol). After purification by flash column chromatography using petroleum ether/dichloromethane (1:1), the title compound was isolated as a colorless oil (41% yield, 1.41 g, two steps from ketone); R_f= 0.4 (PE: DCM = 1:1); ^{1}H NMR (400 MHz, $CDCl_3$) δ 4.08 (s, 2H), 4.07 (s, 2H), 2.27-2.21 (m, 2H), 1.75-1.64 (m, 4H), 1.37-1.15 (m, 7H), 0.86 (t, *J* = 7.2 Hz, 3H); ^{13}C NMR (100 MHz, $CDCl_3$) δ 164.3, 113.2, 37.93, 37.88, 35.8, 29.3, 28.2, 20.0, 14.1 ppm; HRMS (ESI-TOF) *m/z* calcd. for $C_{13}H_{20}Cl_2O_6Na^+$ ($M+Na^+$) 365.0529, found 365.0521.

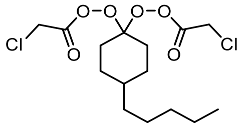

4-Pentylcyclohexane-1,1-diyl bis(2-chloroethaneperoxoate) (S8) Prepared according to general procedure **A1** and **B1** from 4-pentylcyclohexanone (10.0 mmol). After purification by flash column chromatography using petroleum ether/dichloromethane (1:1), the title compound was isolated as a colorless oil (45% yield, 1.70 g, two steps from ketone); R_f= 0.5 (PE: DCM = 1:1); ^{1}H NMR (400 MHz, $CDCl_3$) δ 4.08-4.07 (m, 4H), 2.27-2.23 (m, 2H), 1.76-1.66 (m, 4H), 1.35-1.18 (m, 11H), 0.86 (t, *J* = 6.8

Hz, 3H); ^{13}C NMR (100 MHz, $CDCl_3$) δ 164.4, 164.3, 113.2, 38.0, 37.9, 36.2, 35.7, 31.9, 29.3, 28.3, 26.6, 22.6, 14.0 ppm; HRMS (ESI-TOF) *m/z* calcd. for $C_{15}H_{24}Cl_2O_6Na^+$ (M+Na^+) 393.0842, found 393.0844.

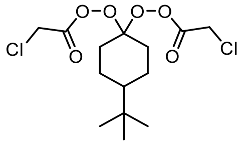

4-(Tert-butyl)cyclohexane-1,1-diyl bis(2-chloroethaneperoxoate) (S9) Prepared according to general procedure **A1** and **B1** from 4-tert-butylcyclohexanone (10.0 mmol). After purification by flash column chromatography using petroleum ether/dichloromethane (1:1), the title compound was isolated as a colorless oil (34% yield, 1.21 g, two steps from ketone); R_f= 0.5 (PE: DCM = 1:1); ^{1}H NMR (400 MHz, $CDCl_3$) δ 4.09 (s, 2H), 4.08 (s, 2H), 2.36-2.30 (m, 2H), 1.81-1.61 (m, 4H), 1.37-1.22 (m, 2H), 1.13-1.05 (m, 1H), 0.85 (s, 9H); ^{13}C NMR (100 MHz, $CDCl_3$) δ 164.4, 112.9, 46.9, 38.0, 37.9, 32.2, 30.0, 27.4, 23.1 ppm; HRMS (ESI-TOF) *m/z* calcd. for $C_{14}H_{22}Cl_2O_6Na^+$ (M+Na^+) 379.0686, found 379.0690.

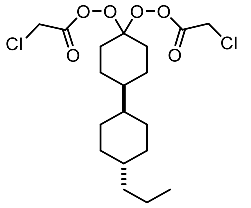

1-[(2-Chloroacetyl)peroxy]-4-(4-propylcyclohexyl)cyclohexyl 2-chloroethan-1-peroxoate (S10) Prepared according to general procedure **A1** and **B1** from 4-propyldicyclohexylanone (10.0 mmol). After purification by flash column chromatography using petroleum ether/dichloromethane (1:1), the title compound was isolated as a white solid (20% yield, 0.89 g, two steps from ketone); R_f= 0.6 (PE: DCM = 1:1); ^{1}H NMR (400 MHz, $CDCl_3$) δ 4.09 (s, 2H), 4.08 (s, 2H), 2.31-2.27 (m, 2H), 1.76-1.64 (m, 8H), 1.37-1.24 (m, 4H), 1.19-1.05 (m, 5H), 1.01-0.93 (m, 2H), 0.87-0.79 (m, 5H); ^{13}C NMR (100 MHz, $CDCl_3$) δ 164.4, 113.2, 42.1, 41.9, 39.6, 38.0, 37.9, 37.4, 33.3, 30.1, 29.7, 25.5, 20.0, 14.4 ppm; HRMS (ESI-TOF) *m/z* calcd. for $C_{19}H_{30}Cl_2O_6Na^+$ (M+Na^+) 447.1312, found 447.1315.

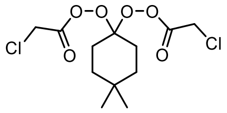

4,4-Dimethylcyclohexane-1,1-diyl bis(2-chloroethaneperoxoate) (S11) Prepared according to general procedure **A1** and **B1** from 4,4-dimethylcyclohexan-1-one (10.0 mmol). After purification by flash column chromatography using petroleum ether/dichloromethane (1:1), the title compound was isolated as a colorless oil (44% yield, 1.45 g, two steps from ketone); R_f= 0.5 (PE: DCM = 1:1); ^{1}H NMR (400 MHz,

$CDCl_3$) δ 4.09 (s, 4H), 2.01-1.97 (m, 4H), 1.44-1.42 (m, 4H), 0.97 (s, 6H); ^{13}C NMR (100 MHz, $CDCl_3$) δ 164.3, 113.1, 38.0, 34.8, 29.6, 27.6, 26.0 ppm; HRMS (ESI-TOF) *m/z* calcd. for $C_{12}H_{18}Cl_2O_6Na^+$ ($M+Na^+$) 351.0373, found 351.0385.

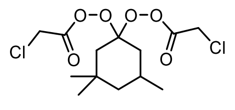

3,3,5-Trimethylcyclohexane-1,1-diyl bis(2-chloroethaneperoxoate) (S12) Prepared according to general procedure **A1** and **B1** from 3,3,5-trimethylcyclohexanone (10.0 mmol). After purification by flash column chromatography using petroleum ether/dichloromethane (1:1), the title compound was isolated as a colorless oil (52% yield, 1.77 g, two steps from ketone); R_f= 0.5 (PE: DCM = 1:1); ^{1}H NMR (400 MHz, $CDCl_3$) δ 4.09 (s, 2H), 4.08 (s, 2H), 2.26-2.21 (m, 1H), 2.07-2.03 (m, 1H), 1.94-1.84 (m, 1H), 1.48 (d, J = 14.2 Hz, 2H), 1.26-1.19 (m, 1H), 1.04 (s, 3H), 0.99 (s, 3H), 0.95-0.88 (m, 4H); ^{13}C NMR (100 MHz, $CDCl_3$) δ 164.4, 164.1, 114.1, 47.6, 40.6, 37.99, 37.96, 37.5, 33.3, 31.7, 26.1, 25.3, 21.7 ppm; HRMS (ESI-TOF) *m/z* calcd. for $C_{13}H_{20}Cl_2O_6Na^+$ ($M+Na^+$) 365.0529, found 365.0527.

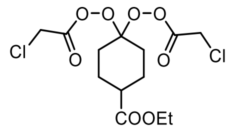

Ethyl 4,4-bis((2-chloroacetyl)peroxy)cyclohexane-1-carboxylate (S13) Prepared according to general procedure **A2** and **B1** from 3,3,5-trimethylcyclohexanone (3.0 mmol). After purification by flash column chromatography using petroleum ether/dichloromethane (1:1), the title compound was isolated as a colorless oil (68% yield, 0.77 g, two steps from ketone); R_f= 0.2 (PE: DCM = 1:1); ^{1}H NMR (400 MHz, $CDCl_3$) δ 4.16-4.11 (m, 2H), 4.09-4.08 (m, 4H), 2.47-2.41 (m, 1H), 2.28-2.24 (m, 2H), 2.01-1.92 (m, 2H), 1.89-1.79 (m, 4H), 1.27-1.23 (m, 3H); ^{13}C NMR (100 MHz, $CDCl_3$) δ 174.0, 164.2, 112.1, 60.7, 40.8, 37.9, 28.3, 24.3, 14.2 ppm; HRMS (ESI-TOF) *m/z* calcd. for $C_{13}H_{18}Cl_2O_8Na^+$ ($M+Na^+$) 395.0271, found 395.0269.

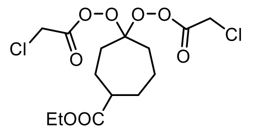

Ethyl 4,4-bis((2-chloroacetyl)peroxy)cycloheptane-1-carboxylate (S14) Prepared according to general procedure **A2** and **B1** from ethyl 4-oxocycloheptanecarboxylate[3] (2.0 mmol). After purification by flash column chromatography using petroleum ether/dichloromethane (1:1), the title compound was isolated as

a colorless oil (35% yield, 0.27 g, two steps from ketone); R_f= 0.4 (PE: DCM = 1:1); ^{1}H NMR (400 MHz, $CDCl_3$) δ 4.14-4.09 (m, 6H), 2.53-2.43 (m, 1H), 2.34-2.18 (m, 2H), 2.06-1.92 (m, 4H), 1.92-1.79 (m, 2H), 1.71-1.54 (m, 2H), 1.26-1.22 (m, 3H); ^{13}C NMR (100 MHz, $CDCl_3$) δ 175.1, 164.3, 164.2, 117.0, 60.6, 45.4, 37.9, 32.9, 31.7, 30.2, 28.3, 24.3, 20.8, 14.1 ppm; HRMS (ESI-TOF) *m/z* calcd. for $C_{14}H_{21}Cl_2O_8^+$ (M+H$^+$) 387.0608, found 387.0608.

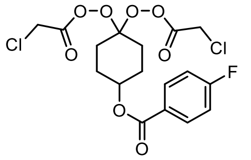

4-Fluorophenyl 4,4-bis((2-chloroacetyl)peroxy)cyclohexane-1-carboxylate (S15) Prepared according to general procedure **A2** and **B1** from the ketone (3.0 mmol) obtained by the reaction of 4-hydroxycyclohexanone with 4-fluorobenzoic acid. After purification by flash column chromatography using petroleum ether/dichloromethane (1:1), the title compound was isolated as a colorless oil (36% yield, 0.47 g, two steps from ketone); R_f= 0.3 (PE: DCM = 1:1); ^{1}H NMR (400 MHz, $CDCl_3$) δ 8.06-8.02 (m, 2H), 7.14-7.10 (m, 2H), 5.23-5.21 (m, 1H), 4.11 (s, 4H), 2.27-2.11 (m, 4H), 2.03-2.00 (m, 4H); ^{13}C NMR (100 MHz, $CDCl_3$) δ 165.8 (d, J = 253.6 Hz), 164.7, 164.2, 132.1 (d, J = 9.4 Hz), 126.3 (d, J = 2.2 Hz), 115.6 (d, J = 22.0 Hz), 112.0, 69.3, 37.9, 26.6, 25.9 ppm; ^{19}F NMR (376 MHz, $CDCl_3$) δ -105.04; HRMS (ESI-TOF) *m/z* calcd. for $C_{17}H_{17}Cl_2O_8FNa^+$ (M+Na$^+$) 461.0177, found 461.0172.

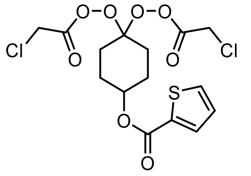

4,4-Bis((2-chloroacetyl)peroxy)cyclohexyl thiophene-2-carboxylate (S16) Prepared according to general procedure **A2** and **B1** from the ketone (3.0 mmol) obtained by the reaction of 4-hydroxycyclohexanone with 2-thiophenecarboxylic acid. After purification by flash column chromatography using petroleum ether/dichloromethane (1:1), the title compound was isolated as a colorless oil (45% yield, 0.58 g, two steps from ketone); R_f= 0.2 (PE: DCM = 1:1); ^{1}H NMR (400 MHz, $CDCl_3$) δ 7.81-7.79 (m, 1H), 7.59-7.57 (m, 1H), 7.13-7.10 (m, 1H), 5.22-5.17 (m, 1H), 4.11 (s, 4H), 2.24-2.18 (m, 2H), 2.17-2.10 (m, 2H), 2.03-1.96 (m, 4H); ^{13}C NMR (100 MHz, $CDCl_3$) δ 164.2, 161.3, 133.5, 132.6, 127.8, 112.0, 69.2, 37.9, 26.6, 25.7 ppm; HRMS (ESI-TOF) *m/z* calcd. for $C_{15}H_{16}Cl_2O_8SNa^+$ (M+Na$^+$) 448.9835, found 448.9826.

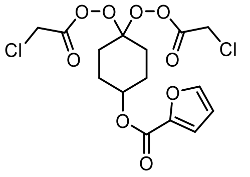

4,4-Bis((2-chloroacetyl)peroxy)cyclohexyl furan-2-carboxylate (S17) Prepared according to general procedure **A2** and **B1** from ketone (3.0 mmol) obtained by the reaction of 4-hydroxycyclohexanone with 2-furoic acid. After purification by flash column chromatography using petroleum ether/dichloromethane (1:1), the title compound was isolated as a colorless oil (23% yield, 0.28 g, two steps from ketone); R_f= 0.2 (PE: DCM = 1:1); ^{1}H NMR (400 MHz, $CDCl_3$) δ 7.60-7.58 (m, 1H), 7.20-7.16 (m, 1H), 6.53-6.51 (m, 1H), 5.23-5.18 (m, 1H), 4.12-4.10 (m, 4H), 2.26-2.19 (m, 2H), 2.16-2.08 (m, 2H), 2.02-1.95 (m, 4H); ^{13}C NMR (100 MHz, $CDCl_3$) δ 164.2, 157.9, 146.5, 144.4, 118.2, 111.9, 69.2, 37.9, 26.6, 25.8 ppm; HRMS (ESI-TOF) *m/z* calcd. for $C_{15}H_{16}Cl_2O_9Na^+$ (M+Na^+) 433.0064, found 433.0063.

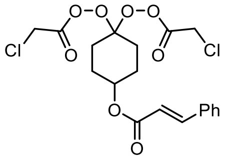

4,4-Bis((2-chloroacetyl)peroxy)cyclohexyl cinnamate (S18) Prepared according to general procedure **A2** and **B1** from ketone (3.0 mmol) obtained by the reaction of 4-hydroxycyclohexanone with trans-cinnamic acid. After purification by flash column chromatography using petroleum ether/dichloromethane (1:1), the title compound was isolated as a colorless oil (36% yield, 0.49 g, two steps from ketone); R_f= 0.3 (PE: DCM = 1:1); ^{1}H NMR (400 MHz, $CDCl_3$) δ 7.69 (d, *J* = 16.2 Hz, 1H), 7.55-7.53 (m, 2H), 7.40-7.39 (m, 3H), 6.44 (d, *J* = 16.0 Hz, 1H), 5.13-5.10 (m, 1H), 4.11 (s, 4H), 2.25-2.08 (m, 4H), 1.98-1.91 (m, 4H); ^{13}C NMR (100 MHz, $CDCl_3$) δ 166.2, 164.2, 145.2, 134.2, 130.5, 128.9, 128.1, 117.9, 112.1, 68.6, 37.9, 26.6, 25.9 ppm; HRMS (ESI-TOF) *m/z* calcd. for $C_{19}H_{20}Cl_2O_8Na^+$ (M+Na^+) 469.0427, found 469.0436.

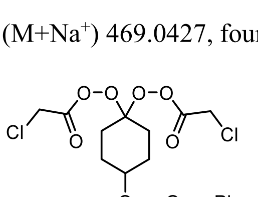

4-(((Benzyloxy)carbonyl)oxy)cyclohexane-1,1-diyl bis(2-chloroethaneperoxoate) (S19) Prepared according to general procedure **A2** and **B1** from the ketone (3.0 mmol) obtained by the reaction of 4-hydroxycyclohexanone with benzyl chloroformate. After purification by flash column chromatography

using petroleum ether/dichloromethane (1:1), the title compound was isolated as a colorless oil (42% yield, 0.57 g, two steps from ketone); R_f= 0.2 (PE: DCM = 1:1); ^{1}H NMR (400 MHz, $CDCl_3$) δ 7.38-7.34 (m, 5H), 5.15 (s, 2H), 4.88-4.85 (m, 1H), 4.08 (s, 4H), 2.19-2.03 (m, 4H), 1.98-1.89 (m, 4H); ^{13}C NMR (100 MHz, $CDCl_3$) δ 164.1, 154.3, 134.9, 128.6, 128.3, 111.8, 72.5, 69.6, 37.8, 26.4, 25.4 ppm; HRMS (ESI-TOF) *m/z* calcd. for $C_{18}H_{20}Cl_2O_9Na^+$ (M+Na$^+$) 473.0377, found 473.0378.

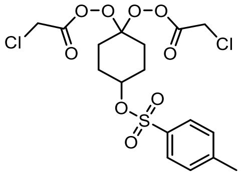

4-(Tosyloxy)cyclohexane-1,1-diyl bis(2-chloroethaneperoxoate) (S20) Prepared according to general procedure **A2** and **B1** from ketone (3.0 mmol) obtained by the reaction of 4-hydroxycyclohexanone with tosyl chloride. After purification by flash column chromatography using petroleum ether/dichloromethane (1:1), the title compound was isolated as a colorless oil (28% yield, 0.39g, two steps from ketone); R_f= 0.2 (PE: DCM = 1:1); ^{1}H NMR (400 MHz, $CDCl_3$) δ 7.77 (d, *J* = 8.4 Hz, 2H), 7.34 (d, *J* = 8.4 Hz, 2H), 4.72-4.68 (m, 1H), 4.07 (s, 2H), 4.06 (s, 2H), 2.43 (s, 3H), 2.10-2.01 (m, 4H), 1.92-1.75 (m, 4H); ^{13}C NMR (100 MHz, $CDCl_3$) δ 164.1, 164.0, 144.9, 133.8, 129.9, 127.5, 111.4, 76.6, 37.8, 27.2, 24.9, 21.6 ppm; HRMS (ESI-TOF) *m/z* calcd. for $C_{17}H_{20}Cl_2O_9SNa^+$ (M+Na$^+$) 493.0097, found 493.0090.

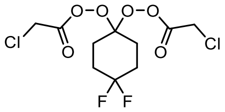

4,4-Difluorocyclohexane-1,1-diyl bis(2-chloroethaneperoxoate) (S21) Prepared according to general procedure **A1** and **B1** from 4,4-difluorocyclohexanone (4.0 mmol). After purification by flash column chromatography using petroleum ether/dichloromethane (1:1), the title compound was isolated as a colorless oil (33% yield, 0.44 g, two steps from ketone); R_f= 0.4 (PE: DCM = 1:1); ^{1}H NMR (400 MHz, $CDCl_3$) δ 4.10 (s, 2H), 4.09 (s, 2H), 2.23-2.20 (m, 4H), 2.14-2.04 (m, 4H); ^{13}C NMR (100 MHz, $CDCl_3$) δ 164.0, 121.7 (t, *J* = 240.3 Hz), 111.0, 37.8, 29.8 (t, *J* = 25.7 Hz), 26.4 (t, *J* = 5.2 Hz) ppm; ^{19}F NMR (376 MHz, $CDCl_3$) δ -99.73 (t, *J* = 12.2 Hz); HRMS (ESI-TOF) *m/z* calcd. for $C_{10}H_{12}Cl_2F_2O_6Na^+$ (M+Na$^+$) 358.9871, found 358.9876.

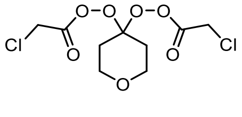

Tetrahydro-2*H*-pyran-4,4-diyl bis(2-chloroethaneperoxoate) (S22) Prepared according to general procedure **A1** and **B1** from tetrahydro-4*H*-pyran-4-on (10.0 mmol). After purification by flash column chromatography using petroleum ether/dichloromethane (1:1), the title compound was isolated as a colorless oil (35% yield, 1.05 g, two steps from ketone); R_f= 0.3 (PE: DCM = 1:1); ^{1}H NMR (400 MHz, $CDCl_3$) δ 4.09 (s, 4H), 3.80-3.77 (m, 4H), 2.12-2.09 (m, 4H); ^{13}C NMR (100 MHz, $CDCl_3$) δ 164.1, 110.4, 63.9, 37.8, 30.5 ppm; HRMS (ESI-TOF) *m/z* calcd. for $C_9H_{12}Cl_2O_7Na^+$ (M+Na$^+$) 324.9852, found 324.9856.

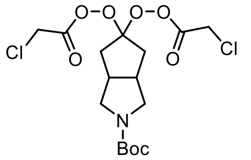

Tert-butyl 5,5-bis((2-chloroacetyl)peroxy)hexahydrocyclopenta[*c*]pyrrole-2(1*H*)-carboxylate (S23) Prepared according to general procedure **A2** and **B1** from *N*-boc-hexahydro-5-oxocyclopenta[c]pyrrole (10.0 mmol). After purification by flash column chromatography using petroleum ether/dichloromethane (1:1), the title compound was isolated as a colorless oil (44% yield, 1.91 g, dr could not be determined by ^{1}H NMR due to peak overlapping, two steps from ketone); R_f= 0.6 (PE: DCM = 1:1); ^{1}H NMR (400 MHz, $CDCl_3$) δ 4.06 (s, 2H), 4.05 (s, 2H), 3.53-3.49 (m, 2H), 3.33-3.21 (m, 2H), 2.86-2.83 (m, 2H), 2.44-2.38 (m, 2H), 2.07-2.02 (m, 2H), 1.41 (s, 9H); ^{13}C NMR (100 MHz, $CDCl_3$) δ 164.1, 163.9, 154.4, 122.8, 79.6, 50.7, 50.4, 41.3, 40.5, 37.8, 37.7, 37.6, 28.3 ppm; HRMS (ESI-TOF) *m/z* calcd. for $C_{16}H_{24}Cl_2NO_8^+$ (M+H$^+$) 428.0873, found 428.0875.

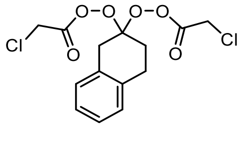

1,2,3,4-Tetrahydronaphthalene-2,2-diyl bis(2-chloroethaneperoxoate) (S24) Prepared according to general procedure **A1** and **B1** from *β*-tetralone (3.0 mmol). After purification by flash column chromatography using petroleum ether/dichloromethane (1:1), the title compound was isolated as a colorless oil (18% yield, 0.19 g, two steps from ketone); R_f= 0.6 (PE: DCM = 1:1); ^{1}H NMR (400 MHz, $CDCl_3$) δ 7.21-7.08 (m, 4H), 4.13 (s, 2H), 4.12 (s, 2H), 3.35 (s, 2H), 2.98 (t, *J* = 6.8 Hz, 2H), 2.30 (t, *J* = 6.8 Hz, 2H); ^{13}C NMR (100 MHz, $CDCl_3$) δ 164.2, 134.5, 130.7, 128.8, 128.4, 126.8, 126.5, 112.9, 37.9, 34.5, 27.1, 26.2 ppm; HRMS (ESI-TOF) *m/z* calcd. for $C_{14}H_{14}Cl_2O_6Na^+$ (M+Na$^+$) 371.0060, found 371.0056.

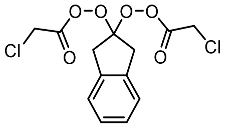

2,3-Dihydro-1*H*-indene-2,2-diyl bis(2-chloroethaneperoxoate) (S25) Prepared according to general procedure **A1** and **B1** from 2-indanone (10.0 mmol). After purification by flash column chromatography using petroleum ether/dichloromethane (1:1), the title compound was isolated as a colorless oil (49% yield, 1.62 g, two steps from ketone); R_f= 0.6 (PE: DCM = 1:1); ^{1}H NMR (400 MHz, $CDCl_3$) δ 7.25-7.19 (m, 4H), 4.12 (s, 4H), 3.51 (s, 4H); ^{13}C NMR (100 MHz, $CDCl_3$) δ 164.1, 137.2, 127.6, 124.8, 121.4, 39.8, 37.9 ppm; HRMS (ESI-TOF) *m*/*z* calcd. for $C_{13}H_{12}Cl_2O_6Na^+$ (M+Na$^+$) 356.9903, found 356.9919.

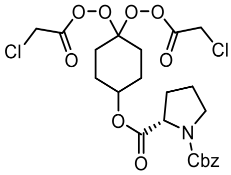

1-Benzyl 2-(4,4-bis((2-chloroacetyl)peroxy)cyclohexyl) (S)-pyrrolidine-1,2-dicarboxylate (S26) Prepared according to general procedure **A2** and **B1** from ketone (3.0 mmol) obtained by the reaction of 4-hydroxycyclohexanone with *N*-benzyloxycarbonyl-proline. After purification by flash column chromatography using petroleum ether/dichloromethane (1:1 to 1:2), the title compound was isolated as a colorless oil (59% yield, 1.00 g, two steps from ketone); R_f= 0.2 (PE: DCM = 1:2); ^{1}H NMR (400 MHz, $CDCl_3$) δ 7.37-7.27 (m, 5H), 5.18-5.05 (m, 2H), 5.04-4.98 (m, 0.58H), 4.88-4.83 (m, 0.42H), 4.38-4.30 (m, 1H), 4.09 (s, 4H), 3.65-3.47 (m, 2H), 2.39-2.19 (m, 2H), 2.15-2.04 (m, 2H), 2.01-1.81 (m, 6H), 1.71-1.55 (m, 2H); ^{13}C NMR (100 MHz, $CDCl_3$) δ 172.2, 172.0, 164.31, 164.29, 164.26, 164.24, 155.0, 154.4, 136.7, 136.5, 128.6, 128.5, 128.2, 128.1, 128.0, 112.1, 111.9, 69.4, 69.3, 67.2, 59.5, 59.0, 47.1, 46.6, 38.00, 37.98, 31.1, 30.1, 26.51, 26.50, 26.39, 26.37, 26.33, 25.8, 25.7, 25.6, 24.5, 23.7 ppm; HRMS (ESI-TOF) *m*/*z* calcd. for $C_{23}H_{27}Cl_2NO_{10}Na^+$ (M+Na$^+$) 570.0904, found 570.0909.

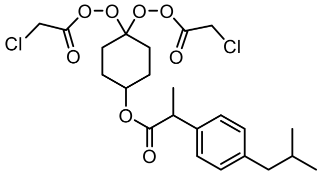

4,4-Bis((2-chloroacetyl)peroxy)cyclohexyl 2-(4-isobutylphenyl)propanoate (S27) Prepared according to general procedure **A2** and **B1** from ketone (3.0 mmol) obtained by the reaction of 4-hydroxycyclohexanone with ibuprofen. After purification by flash column chromatography using

petroleum ether/dichloromethane (1:1), the title compound was isolated as a colorless oil (56% yield, 0.85 g, two steps from ketone); R_f= 0.3 (PE: DCM = 1:1); ^{1}H NMR (400 MHz, $CDCl_3$) δ 7.19-7.17 (m, 2H), 7.09-7.07 (m, 2H), 4.97-4.93 (m, 1H), 4.06 (s, 4H), 3.68 (q, *J* = 7.2 Hz, 1H), 2.43 (d, *J* = 7.2 Hz, 2H), 2.03-1.71 (m, 9H), 1.48 (d, *J* = 7.2 Hz, 3H), 0.87 (d, *J* = 6.8 Hz, 6H); ^{13}C NMR (100 MHz, $CDCl_3$) δ 173.9, 164.15, 164.11, 140.6, 137.5, 129.3, 126.9, 111.9, 68.2, 45.2, 44.9, 37.8, 30.1, 26.3, 26.0, 25.3, 25.2, 22.3, 17.8 ppm; HRMS (ESI-TOF) *m/z* calcd. for $C_{23}H_{30}Cl_2O_8Na^+$ (M+Na^+) 527.1210, found 527.1227.

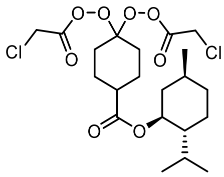

(2r,5s)-2-Isopropyl-5-methylcyclohexyl 4,4-bis((2-chloroacetyl)peroxy)cyclohexane-1-carboxylate (S28) Prepared according to general procedure **A2** and **B1** from ketone (3.0 mmol) obtained by the reaction of 4-hydroxycyclohexanone with *L*-menthol. After purification by flash column chromatography using petroleum ether/dichloromethane (1:1), the title compound was isolated as a colorless oil (39% yield, 0.56 g, two steps from ketone); R_f= 0.3 (PE: DCM = 1:1); ^{1}H NMR (400 MHz, $CDCl_3$) δ 4.70-4.64 (td, *J* = 10.8, 4.4 Hz, 1H), 4.09 (s, 2H), 4.08 (s, 2H), 2.46-2.40 (m, 1H), 2.29-2.24 (m, 2H), 1.98-1.93 (m, 3H), 1.86-1.81 (m, 5H), 1.69-1.64 (m, 2H), 1.51-1.44 (m, 1H), 1.41-1.33 (m, 1H), 1.06-0.87 (m, 9H), 0.73 (d, *J* = 6.9 Hz, 3H); ^{13}C NMR (100 MHz, $CDCl_3$) δ 173.5, 164.2, 164.1, 112.2, 74.4, 46.9, 41.1, 40.8, 37.91, 37.87, 34.2, 31.3, 28.4, 26.2, 24.4, 24.3, 23.2, 22.0, 20.8, 16.1 ppm; HRMS (ESI-TOF) *m/z* calcd. for $C_{21}H_{32}Cl_2O_8Na^+$ (M+Na^+) 505.1366, found 505.1361.

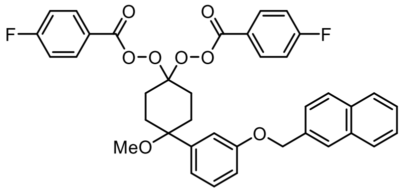

4-Methoxy-4-(3-(naphthalen-2-ylmethoxy)phenyl)cyclohexane-1,1-diyl bis(4-fluorobenzoperoxoate) (S29) Prepared according to general procedure **A2** and **B2** from ketone[4] (10.0 mmol). After purification by flash column chromatography using petroleum ether/ethyl acetate (10:1), the title compound was isolated as a white solid (54% yield, 3.54 g); R_f= 0.7 (PE: EA = 10:1); ^{1}H NMR (400 MHz, $CDCl_3$) δ 8.05-8.00 (m, 4H), 7.92 (s, 1H), 7.89-7.84 (m, 3H), 7.58-7.56 (m, 1H), 7.50-7.48 (m, 2H), 7.32 (t, *J* = 8.0 Hz, 1H), 7.16-7.12 (m, 5H), 7.07-7.04 (m, 1H), 6.99-6.96 (m, 1H), 5.26 (s, 2H), 3.03 (s, 3H), 2.44-2.33

(m, 4H), 2.20-2.11 (m, 4H); ^{13}C NMR (100 MHz, $CDCl_3$) δ 165.95 (d, J = 255.7 Hz), 162.8, 126.7, 158.9, 145.5, 134.3, 133.2, 133.0, 131.96 (d, J = 9.4 Hz), 131.93 (d, J = 9.4 Hz), 129.5, 128.3, 127.8, 127.6, 126.4, 126.2, 126.0, 125.3, 123.18 (d, J = 3.1 Hz), 118.6, 115.92 (d, J = 22.1 Hz), 115.89 (d, J = 22.1 Hz), 113.4, 113.1, 112.1, 76.2, 70.0, 49.9, 31.1, 25.7 ppm; ^{19}F NMR (376 MHz, $CDCl_3$) δ -103.59, -103.71. HRMS (ESI-TOF) m/z calcd. for $C_{38}H_{32}F_2O_8Na^+$ (M+Na$^+$) 677.1958, found 677.1969.

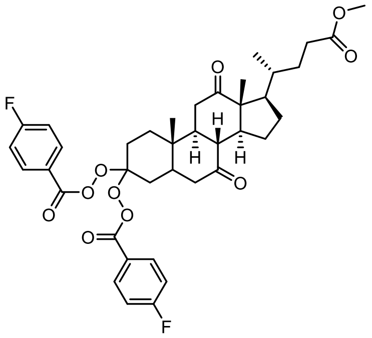

Methyl (4r)-4-((8r,9s,10s,13r,14s,17r)-3,3-bis((4-fluorobenzoyl)peroxy)-10,13-dimethyl-7,12-dioxo-hexadecahydro-1*H*-cyclopenta[*a*]phenanthren-17-yl)pentanoate (S30) Prepared according to general procedure **A2** and **B2** from cholic acid[4] (3.0 mmol). After purification by flash column chromatography using petroleum ether/ethyl acetate (10:1 to 5:1), the title compound was isolated as a white solid (47% yield, 1.00 g, two steps from ketone); R_f = 0.6 (PE: EA = 5:1); ^{1}H NMR (400 MHz, $CDCl_3$) δ 7.97-7.92 (m, 4H), 7.16-7.09 (m, 4H), 3.66 (s, 3H), 2.97-2.92 (m, 1H), 2.87-2.73 (m, 2H), 2.43-2.19 (m, 7H), 2.09-1.99 (m, 4H), 1.88-1.66 (m, 6H), 1.42-1.18 (m, 7H), 1.04 (s, 3H), 0.84 (d, J = 6.6 Hz, 3H); ^{13}C NMR (100 MHz, $CDCl_3$) δ 211.9, 208.9, 174.6, 166.1 (d, J = 250.4 Hz), 162.7, 162.5, 132.04 (d, J = 9.4 Hz), 132.00 (d, J = 9.4 Hz), 123.00, 122.95, 116.07 (d, J = 22.2 Hz), 115.98 (d, J = 22.2 Hz), 111.9, 56.8, 51.54, 51.48, 48.9, 45.6, 45.1, 44.5, 43.0, 38.5, 36.1, 35.5, 31.9, 31.34, 31.28, 30.4, 27.6, 25.1, 24.2, 22.1, 18.6, 11.8 ppm; ^{19}F NMR (376 MHz, $CDCl_3$) δ -103.37, -103.66. HRMS (ESI-TOF) m/z calcd. for $C_{39}H_{44}F_2O_{10}Na^+$ (M+Na$^+$) 733.2795, found 733.2803.

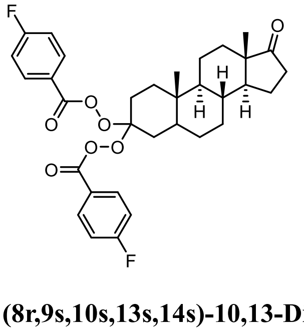

(8r,9s,10s,13s,14s)-10,13-Dimethyl-17-oxohexadecahydro-1*H*-cyclopenta[*a*]phenanthrene-3,3-diyl bis(4-fluorobenzoperoxoate) (S31) Prepared according to general procedure **A2** and **B2** from

epiandrosterone[4] (3.0 mmol). After purification by flash column chromatography using petroleum ether/ethyl acetate (10:1 to 6:1), the title compound was isolated as a white solid (49% yield, 0.85 g, two steps from ketone); R_f= 0.5 (PE: EA = 6:1); ^{1}H NMR (400 MHz, $CDCl_3$) δ 7.99-7.95 (m, 4H), 7.15-7.09 (m, 4H), 2.46-2.39 (m, 1H), 2.34-2.30 (m, 1H), 2.11-2.02 (m, 2H), 1.98-1.77 (m, 6H), 1.67-1.50 (m, 4H), 1.42-1.21 (m, 6H), 1.08-1.02 (m, 1H), 0.91-0.82 (m, 7H); ^{13}C NMR (100 MHz, $CDCl_3$) δ 221.0, 166.0 (d, *J* = 254.1 Hz), 162.9, 162.7, 132.00 (d, *J* = 9.6 Hz), 131.95 (d, *J* = 9.6 Hz), 123.27 (d, *J* = 3.0 Hz), 123.19 (d, *J* = 3.0 Hz), 15.97 (d, *J* = 22.2 Hz), 115.94 (d, *J* = 22.2 Hz), 112.7, 53.6, 51.2, 47.7, 42.1, 35.9, 35.8, 34.9, 34.5, 32.6, 31.4, 30.5, 27.8, 25.8, 21.7, 20.4, 13.8, 11.5 ppm; ^{19}F NMR (376 MHz, $CDCl_3$) δ -103.73, 103.80. HRMS (ESI-TOF) *m/z* calcd. for $C_{33}H_{36}F_2O_7Na^+$ (M+Na^+) 605.2321, found 605.2330.

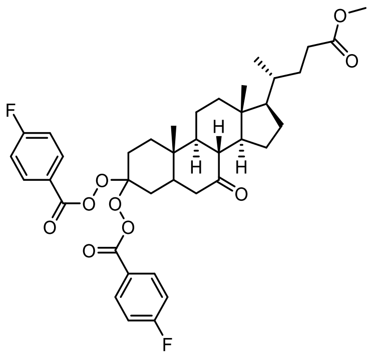

Methyl (4r)-4-((8r,9s,10s,13r,14s,17r)-3,3-bis((4-fluorobenzoyl)peroxy)-10,13-dimethyl-7-oxohexadecahydro-1*H*-cyclopenta[*a*]phenanthren-17-yl)pentanoate (S32) Prepared according to general procedure **A2** and **B2** from chenodeoxycholic acid[4] (3.0 mmol). After purification by flash column chromatography using petroleum ether/ethyl acetate (10:1 to 5:1), the title compound was isolated as a white solid (29% yield, 0.61 g, two steps from ketone); R_f= 0.6 (PE: EA = 5:1); ^{1}H NMR (400 MHz, $CDCl_3$) δ 7.96-7.92 (m, 4H), 7.13-7.08 (m, 4H), 3.64 (s, 3H), 2.91-2.86 (m, 1H), 2.42 (t, *J* = 11.2 Hz, 1H), 2.37-2.16 (m, 6H), 2.01-1.86 (m, 3H), 1.81-1.71 (m, 5H), 1.65-1.55 (m, 2H), 1.48-1.18 (m, 8H), 1.15-1.07 (m, 2H), 0.97-0.85 (m, 4H), 0.64 (s, 3H); ^{13}C NMR (100 MHz, $CDCl_3$) δ 211.3, 174.5, 166.0 (d, *J* = 255.6 Hz), 162.6, 131.98 (d, *J* = 9.6 Hz), 131.93 (d, *J* = 9.6 Hz), 123.06 (d, *J* = 3.2 Hz), 122.99 (d, *J* = 3.2 Hz), 155.97 (d, *J* = 22.2 Hz), 115.91 (d, *J* = 22.2 Hz), 112.4, 54.7, 51.4, 49.4, 48.7, 44.5, 43.8, 42.5, 42.3, 38.7, 35.4, 35.1, 31.8, 31.4, 30.93, 30.87, 28.2, 24.7, 24.3, 22.6, 21.8, 18.3, 12.0 ppm; ^{19}F NMR (376 MHz, $CDCl_3$) δ -103.53, -103.70. HRMS (ESI-TOF) *m/z* calcd. for $C_{39}H_{46}F_2O_9Na^+$ (M+Na^+) 719.3002, found 719.3010.

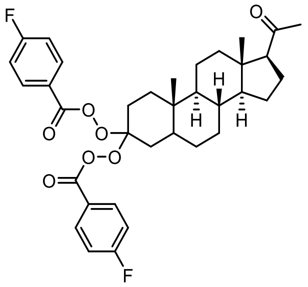

(8r,9s,10s,13s,14s)-17-Acetyl-10,13-dimethylhexadecahydro-1*H*-cyclopenta[*a*]phenanthrene-3,3-diyl bis(4-fluorobenzoperoxoate) (S33) Prepared according to general procedure **A2** and **B2** from progesterone[4] (3.0 mmol). After purification by flash column chromatography using petroleum ether/dichloromethane (1:1), the title compound was isolated as a white solid (69% yield, 1.26 g, two steps from ketone); R_f= 0.5 (PE: DCM = 1:1); ^{1}H NMR (400 MHz, $CDCl_3$) δ 7.99-7.96 (m, 4H), 7.15-7.10 (m, 4H), 2.52 (t, *J* = 9.1 Hz, 1H), 2.33-2.29 (m, 1H), 2.18-2.06 (m, 4H), 2.06-1.86 (m, 3H), 1.82-1.57 (m, 7H), 1.44-1.15 (m, 8H), 1.02-0.94 (m, 1H), 0.91-0.78 (m, 4H), 0.60 (s, 3H); ^{13}C NMR (100 MHz, $CDCl_3$) δ 209.6, 166.0 (d, *J* = 254.1 Hz), 162.9, 162.8, 132.01 (d, *J* = 9.5 Hz), 131.96 (d, *J* = 9.5 Hz), 123.31 (d, *J* = 3.2 Hz), 123.22 (d, *J* = 3.2 Hz), 115.98 (d, *J* = 22.2 Hz), 115.94 (d, *J* = 22.2 Hz), 112.8, 63.7, 56.4, 53.4, 44.1, 42.1, 38.9, 35.8, 35.4, 34.5, 32.6, 31.6, 31.5, 28.0, 25.8, 24. 3, 22.8, 21.2, 13.4, 11.5 ppm; ^{19}F NMR (376 MHz, $CDCl_3$) δ -103.75, -103.84. HRMS (ESI-TOF) *m/z* calcd. for $C_{35}H_{40}F_2O_7Na^+$ (M+Na^+) 633.2634, found 633.2642.

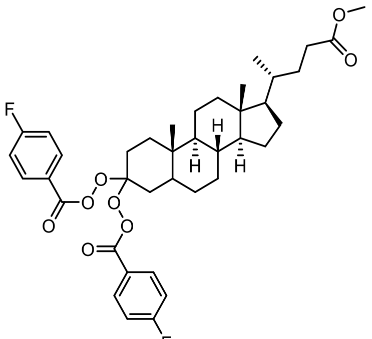

Methyl (4r)-4-((8r,9s,10s,13r,14s,17r)-3,3-bis((4-fluorobenzoyl)peroxy)-10,13-dimethylhexadecahydro-1*H*-cyclopenta[*a*]phenanthren-17-yl)pentanoate (S34) Prepared according to general procedure **A2** and **B2** from lithocholic acid[4] (3.0 mmol). After purification by flash column chromatography using petroleum ether/dichloromethane (5:1 to 1:1), the title compound was isolated as a white solid (69% yield, 1.41 g, two steps from ketone); R_f= 0.4 (PE: DCM = 3:1); ^{1}H NMR (400 MHz, $CDCl_3$) δ 8.01-7.95 (m, 4H), 7.16-7.10 (m, 4H), 3.66 (s, 3H), 2.37-2.18 (m, 4H), 2.04-1.74 (m, 8H), 1.67-1.54 (m, 2H), 1.49-1.25 (m, 10H), 1.18-1.06 (m, 7H), 0.91 (d, *J* = 6.4 Hz, 3H), 0.65 (s, 3H); ^{13}C NMR (100 MHz, $CDCl_3$) δ 174.8,

166.1 (d, J = 251.4 Hz), 163.0, 162.8, 132.09 (d, J = 9.2 Hz), 131.99 (d, J = 9.2 Hz), 123.4, 123.3, 116.0 (d, J = 22.0 Hz), 113.6, 56.3, 56.0, 51.5, 42.7, 40.0, 39.5, 35.6, 35.4, 34.9, 32.5, 31.1, 31.0, 30.9, 28.2, 26.3, 26.0, 24.8, 24.2, 23.0, 21.0, 18.3, 12.1 ppm; ^{19}F NMR (376 MHz, $CDCl_3$) δ -103.89. HRMS (ESI-TOF) *m/z* calcd. for $C_{39}H_{48}F_2O_8Na^+$ (M+Na^+) 705.3209, found 705.3218.

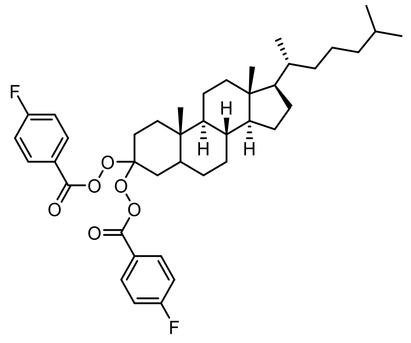

(8r,9s,10s,13r,14s,17r)-10,13-Dimethyl-17-((r)-6-methylheptan-2-yl)hexadecahydro-1*H*-cyclo penta[*a*]phenanthrene-3,3-diyl bis(4-fluorobenzoperoxoate) (S35) Prepared according to general procedure **A2** and **B2** from Cholesterol[4] (3.0 mmol). After purification by flash column chromatography using petroleum ether/ethyl acetate (40:1 to 25:1), the title compound was isolated as a white solid (44% yield, 0.89 g, two steps from ketone); R_f= 0.4 (PE: EA = 25:1); ^{1}H NMR (400 MHz, $CDCl_3$) δ 8.00-7.96 (m, 4H), 7.15-7.11 (m, 4H), 2.33-2.29 (m, 1H), 2.08-2.05 (m, 1H), 1.99-1.90 (m, 2H), 1.86-1.76 (m, 2H), 1.66-1.47 (m, 8H), 1.40-1.23 (m, 9H), 1.06-0.80 (m, 20H), 0.66 (s, 3H); ^{13}C NMR (100 MHz, $CDCl_3$) δ 166.1 (d, J = 254.8 Hz), 163.0, 162.8, 132.06 (d, J = 9.4 Hz), 132.01 (d, J = 9.4 Hz), 123.4, 123.3, 116.00 (d, J = 22.2 Hz), 115.97 (d, J = 22.2 Hz), 113.0, 56.3, 56.2, 53.6, 42.6, 42.2, 39.9, 39.5, 36.2, 35.8, 35.4, 34.6, 32.7, 31.7, 28.23, 28.16, 28.0, 25.9, 24.2, 23.9, 22.8, 22.6, 21.2, 18.7, 12.1, 11.6 ppm; ^{19}F NMR (376 MHz, $CDCl_3$) δ -103.88, -103.93. HRMS (ESI-TOF) *m/z* calcd. for$C_{41}H_{54}F_2O_6Na^+$ (M+Na^+) 703.3781, found 703.3790.

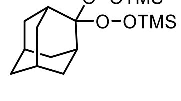

2,2-Bis[(trimethylsilyl)dioxidanyl]tricyclo[3.3.1.1^{3,7}]decane (S36) Prepared according to general procedure **A1B3** from 2-adamantanone (10.0 mmol). After purification by flash column chromatography using petroleum ether/dichloromethane (10:1 to 5:1), the title compound was isolated as a colorless oil (40% yield, 1.36 g, two steps from ketone); R_f= 0.9 (PE: DCM = 5:1); ^{1}H NMR (400 MHz, $CDCl_3$) δ 2.38-2.37 (m, 2H), 1.97-1.93 (m, 4H), 1.83-1.81 (m, 2H), 1.67-1.58 (m, 6H), 0.21 (s, 18H); ^{13}C NMR

(100 MHz, $CDCl_3$) δ 110.8, 37.4, 33.9, 31.7, 27.2, -1.11 ppm; HRMS (ESI-TOF) *m/z* calcd. for $C_{16}H_{32}Si_2O_4Na^+$ ($M+Na^+$) 367.1731, found 367.1734.

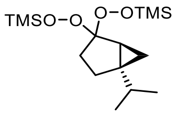

(1aR,4aS)-1a-(Prop-2-yl)-4,4-bis[(trimethylsilyl)dioxidanyl]-2,3,4,4a-tetrahydro-1a*H*-cyclopropa [1,2-*a*][5]annulene (S37) Prepared according to general procedure **A1** and **B3** from sabinene[5] (6.0 mmol). After purification by flash column chromatography using petroleum ether/dichloromethane (20:1), the title compound was isolated as a colorless oil (20% yield, 0.39 g, two steps from ketone); R_f= 0.9 (PE); ^1H NMR (400 MHz, $CDCl_3$) δ 2.07-2.02 (m, 1H), 1.82-1.74 (m, 1H), 1.70-1.65 (m, 1H), 1.61-1.58 (m, 1H), 1.48-1.36 (m, 2H), 0.94 (d, *J* = 6.8 Hz, 3H), 0.84 (d, *J* = 6.8 Hz, 3H), 0.61-0.55 (m, 2H), 0.23 (s, 9H), 0.22 (s, 9H); ^{13}C NMR (100 MHz, $CDCl_3$) δ 120.3, 33.6, 32.0, 28.1, 26.0, 25.8, 19.7, 19.4, 12.4, -1.0, -1.1 ppm; HRMS (ESI-TOF) *m/z* calcd. for $C_{15}H_{33}O_4Si_2^+$ ($M+H^+$) 333.1912, found 333.1916.

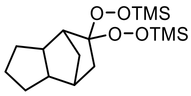

8,8-Bis[(trimethylsilyl)dioxidanyl]tricyclo[5.2.1.02,6]decane (S38) Prepared according to general procedure **A1** and **B3** from tricyclo[5.2.1.02,6]decan-8-one (10.0 mmol). After purification by flash column chromatography using petroleum ether/dichloromethane (15:1 to 5:1), the title compound was isolated as a colorless oil (44% yield, 1.50 g, two steps from ketone); R_f= 0.9 (PE: DCM = 5:1); ^1H NMR (400 MHz, $CDCl_3$) δ 2.40-2.36 (m, 2H), 2.00-1.80 (m, 5H), 1.69-1.62 (m, 1H), 1.48-1.38 (m, 3H), 1.28-1.20 (m, 1H), 0.98-0.84 (m, 2H), 0.22 (s, 18H); ^{13}C NMR (100 MHz, $CDCl_3$) δ 118.3, 47.4, 47.2, 40.4, 40.1, 39.1, 32.4, 31.6, 31.5, 27.4, -1.0 ppm; HRMS (ESI-TOF) *m/z* calcd. for $C_{16}H_{32}Si_2O_4Na^+$ ($M+Na^+$) 367.1731, found 367.1712.

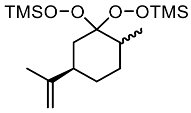

(1aR,4aS)-1*a*-(Prop-2-yl)-4,4-bis[(trimethylsilyl)dioxidanyl]-2,3,4,4*a*-tetrahydro-1a*H*-cyclopropa [1,2-*a*][5]annulene (S39) Prepared according to general procedure **A1** and **B3** from (+)-dihydrocarvone (10.0 mmol). After purification by flash column chromatography using petroleum ether/dichloromethane (15:1), the title compound was isolated as a colorless oil (57% yield, dr = 1.7:1, dr was determined by ^1H NMR analysis, 1.96 g, two steps from ketone); R_f= 0.9 (PE); ^1H NMR (400 MHz, $CDCl_3$) δ 4.71 (s, 2H),

2.74-2.70 (m, 0.64H), 2.43-2.39 (m, 0.36H), 2.24-2.13 (m, 1.27H), 1.95-1.84 (m, 0.73H), 1.76-1.73 (m, 3H), 1.65-1.59 (m, 1H), 1.53-1.16 (m, 4H), 1.02 (d, J = 7.2 Hz, 1.1H), 0.96 (d, J = 6.8 Hz, 1.9H), 0.24-0.20 (m, 18H); ^{13}C NMR (100 MHz, $CDCl_3$) δ 149.7, 149.6, 112.0, 110.8, 108.5, 108.4, 41.2, 41.00, 38.9, 34.3, 32.2, 30.94, 30.92, 29.9, 29.4, 25.0, 21.1, 21.0, 14.6, 14.2, -1.05, -1.07, -1.10, -1.15 ppm; HRMS (ESI-TOF) *m/z* calcd. for $C_{16}H_{34}O_4Si_2Na^+$ (M+Na$^+$) 369.1888, found 369.1896.

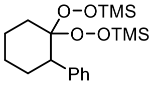

2-Phenyl-1,1-bis[(trimethylsilyl)dioxidanyl]cyclohexane (S40) Prepared according to general procedure **A1** and **B3** from 2-phenylcyclohexanone (10.0 mmol). After purification by flash column chromatography using petroleum ether/dichloromethane (5:1 to 3:1), the title compound was isolated as a colorless oil (45% yield, 1.60 g, two steps from ketone); R_f= 0.9 (PE: DCM = 5:1); ^{1}H NMR (400 MHz, $CDCl_3$) δ 7.29-7.15 (m, 5H), 3.14-3.09 (m, 1H), 2.72-2.65 (m, 1H), 2.04-1.96 (m, 1H), 1.83-1.77 (m, 2H), 1.70-1.63 (m, 2H), 1.45-1.29 (m, 2H), 0.09-0.06 (m, 18H); ^{13}C NMR (100 MHz, $CDCl_3$) δ 140.6, 129.7, 127.1, 126.1, 110.3, 49.9, 30.4, 29.4, 25.5, 22.5, -1.2 -1.3 ppm; HRMS (ESI-TOF) *m/z* calcd. for $C_{18}H_{32}Si_2O_4Na^+$ (M+Na$^+$) 391.1731, found 391.1732.

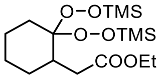

Ethyl {2,2-bis[(trimethylsilyl)dioxidanyl]cyclohexyl}acetate (S41) Prepared according to general procedure **A2** and **B3** from ethyl 2-cyclohexanoneacetate (5.0 mmol). After purification by flash column chromatography using petroleum ether/dichloromethane (5:1 to 1:1), the title compound was isolated as a colorless oil (10% yield, 0.19 g, two steps from ketone); R_f= 0.6 (PE: DCM = 2:1); ^{1}H NMR (400 MHz, $CDCl_3$) δ 4.12 (q, J = 7.2 Hz, 2H), 2.75-2.70 (m, 1H), 2.66-2.59 (m, 1H), 2.26-2.20 (m, 1H), 1.88-1.76 (m, 2H), 1.71-1.62 (m, 1H), 1.57-1.35 (m, 5H), 1.24 (t, J = 7.2 Hz, 3H), 0.19 (s, 9H), 0.18 (s, 9H); ^{13}C NMR (100 MHz, $CDCl_3$) δ 173.2, 110.3, 60.2, 36.6, 33.7, 27.9, 27.5, 22.4, 22.1, 14.2, -1.18, -1.21 ppm; HRMS (ESI-TOF) *m/z* calcd. for $C_{16}H_{34}O_6Si_2Na^+$ (M+Na$^+$) 401.1786, found 401.1797.

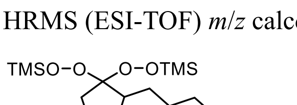

2-Heptyl-1,1-bis[(trimethylsilyl)dioxidanyl]cyclopentane (S42) Prepared according to general procedure **A1** and **B3** from 2-*N*-heptylcyclopentanone (10.0 mmol). After purification by flash column chromatography using petroleum ether/dichloromethane (15:1 to 10:1), the title compound was isolated as a colorless oil (21% yield, 0.79 g, two steps from ketone); R_f= 0.9 (PE); ^{1}H NMR (400 MHz, $CDCl_3$)

δ 2.30-2.25 (m, 1H), 2.06-1.88 (m, 3H), 1.72-1.67 (m, 2H), 1.59-1.51 (m, 1H), 1.34-1.25 (m, 11H), 1.18-1.10 (m, 1H), 0.88 (t, *J* = 6.8 Hz, 3H), 0.22 (s, 9H), 0.21 (s, 9H); ^{13}C NMR (100 MHz, $CDCl_3$) δ 119.7, 47.6, 33.9, 32.2, 31.9, 29.7, 29.33, 29.30, 28.3, 22.7, 14.1, -1.1 ppm; HRMS (ESI-TOF) *m*/*z* calcd. for $C_{18}H_{40}O_4Si_2Na^+$ (M+Na$^+$) 399.2357, found 399.2365.

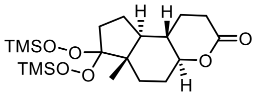

(4aS,6aS,9aS,9bS)-6a-Methyl-7,7-bis[(trimethylsilyl)dioxidanyl]dodecahydrocyclopenta[1,2-*f*] chromen-3-one (S43) Prepared according to general procedure **A2** and **B3** from delta-lactone (10.0 mmol). After purification by flash column chromatography using petroleum ether/dichloromethane (5:1 to 1:1), the title compound was isolated as a colorless oil (15% yield, 0.62 g, two steps from ketone); R_f = 0.8 (PE: DCM = 1:1); ^{1}H NMR (400 MHz, $CDCl_3$) δ 4.45-4.43 (m, 1H), 2.54-2.48 (m, 1H), 2.44-2.33 (m, 1H), 2.05-1.89 (m, 4H), 1.81-1.63 (m, 4H), 1.45-1.37 (m, 1H), 1.03 (s, 3H), 0.88-0.82 (m, 3H), 0.19 (s, 18H); ^{13}C NMR (100 MHz, $CDCl_3$) δ 172.1, 119.1, 78.5, 49.6, 41.4, 32.8, 27.4, 27.1, 26.6, 26.2, 23.4, 21.9, 12.8, -1.17, -1.21 ppm; HRMS (ESI-TOF) *m*/*z* calcd. for $C_{19}H_{36}O_6Si_2Na^+$ (M+Na$^+$) 439.1943, found 439.1945.

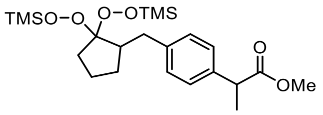

Methyl 2-[4-({2,2-bis[(trimethylsilyl)dioxidanyl]cyclopentyl}methyl)phenyl]propanoate (S44) Prepared according to general procedure **A2** and **B3** from loxoprofen (8.0 mmol). After purification by flash column chromatography using petroleum ether/dichloromethane (5:1 to 1:1), the title compound was isolated as a colorless oil (27% yield, 0.99 g, two steps from ketone); R_f= 0.3 (PE: DCM = 1:1); ^{1}H NMR (400 MHz, $CDCl_3$) δ 7.20-7.18 (m, 2H), 7.14-7.12 (m, 2H), 3.72-3.65 (m, 4H), 3.22-3.08 (m, 1H), 2.60-2.52 (m, 1H), 2.38-2.29 (m, 1H), 2.16-2.10 (m, 1H), 1.99-1.93 (m, 1H), 1.80-1.67 (m, 2H), 1.54-1.36 (m, 5H), 0.25 (s, 9H), 0.23 (s, 9H); ^{13}C NMR (100 MHz, $CDCl_3$) δ 175.2, 140.8, 137.8, 129.2, 129.1, 127.3, 127.2, 119.2, 51.9, 49.8, 45.0, 35.3, 33.7, 31.7, 22.5, 18.6, -1.09, -1.13 ppm; HRMS (ESI-TOF) *m*/*z* calcd. for $C_{22}H_{38}O_6Si_2Na^+$ (M+Na$^+$) 477.2099, found 477.2098.

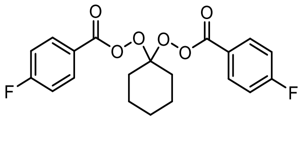

Cyclohexane-1,1-diyl bis(4-fluorobenzoperoxoate) (S45) Prepared according to general procedure **A1** and **B2** from cyclohexanone (10.0 mmol). After purification by flash column chromatography using petroleum ether/dichloromethane (5:1 to 1:1), the title compound was isolated as a white solid (49% yield, 1.92 g, two steps from ketone); R_f= 0.4 (PE: DCM = 3:1); ^{1}H NMR (400 MHz, $CDCl_3$) δ 8.00-7.97 (m, 4H), 7.15-7.11 (m, 4H); 2.13-2.09 (m, 4H), 1.77-1.71 (m, 4H), 1.59-1.53 (m, 2H); ^{13}C NMR (100 MHz, $CDCl_3$) δ 166.1 (d, J = 262.1 Hz), 162.9, 132.0 (d, J = 8.9 Hz), 123.3, 116.0 (d, J = 22.2 Hz), 112.7, 30.1. 25.0, 22.3 ppm; ^{19}F NMR (376 MHz, $CDCl_3$) δ -103.93; HRMS (ESI-TOF) *m/z* calcd. for $C_{20}H_{18}F_2O_6Na^+$ (M+Na$^+$) 415.0964, found 414.0964.

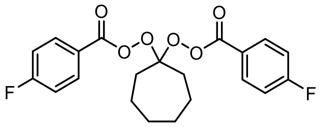

Cycloheptane-1,1-diyl bis(4-fluorobenzoperoxoate) (S46) Prepared according to general procedure **A1** and **B2** from cycloheptanone (10.0 mmol). After purification by flash column chromatography using petroleum ether/dichloromethane (5:1 to 1:1), the title compound was isolated as a white solid (24% yield, 0.97 g, two steps from ketone); R_f= 0.4 (PE: DCM = 3:1); ^{1}H NMR (400 MHz, $CDCl_3$) δ 7.98-7.94 (m, 4H), 7.13-7.08 (m, 4H), 2.23-2.20 (m, 4H), 1.74-1.70 (m, 4H), 1.64-1.62 (m, 4H); ^{13}C NMR (100 MHz, $CDCl_3$) δ 165.9 (d, J = 254.1 Hz), 162.8, 131.9 (d, J = 9.6 Hz), 123.3 (d, J = 3.8 Hz), 117.6, 115.9 (d, J = 22.2 Hz), 33.2, 29.8, 22.6 ppm; ^{19}F NMR (376 MHz, $CDCl_3$) δ -103.93; HRMS (ESI-TOF) *m/z* calcd. for $C_{21}H_{20}F_2O_6Na^+$ (M+Na$^+$) 429.1120, found 429.1116.

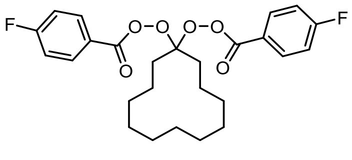

Cyclododecane-1,1-diyl bis(4-fluorobenzoperoxoate) (S47) Prepared according to general procedure **A1** and **B2** from cyclododecanone (10.0 mmol). After purification by flash column chromatography using petroleum ether/dichloromethane (5:1 to 1:1), the title compound was isolated as a white solid (36% yield, 1.71 g, two steps from ketone); R_f= 0.4 (PE: DCM = 3:1); ^{1}H NMR (400 MHz, $CDCl_3$) δ 7.90-7.87 (m, 4H), 7.06-7.02 (m, 4H), 1.92-1.88 (m, 4H), 1.61-1.51 (m, 4H), 1.40-1.13 (m, 14H); ^{13}C NMR (100 MHz, $CDCl_3$) δ 166.0 (d, J = 255.3 Hz), 162.7, 132.0 (d, J = 9.3 Hz), 123.5 (d, J = 3.2 Hz), 116.6, 116.0 (d, J = 22.2 Hz), 26.8, 26.0, 25.9, 22.2, 21.9, 19.4 ppm; ^{19}F NMR (376 MHz, $CDCl_3$) δ -104.01; HRMS (ESI-TOF) *m/z* calcd. for $C_{26}H_{30}F_2O_6Na^+$ (M+Na$^+$) 499.1903, found 499.1900.

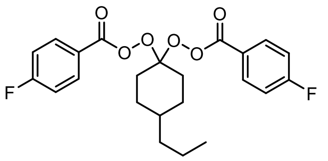

4-Propylcyclohexane-1,1-diyl bis(4-fluorobenzoperoxoate) (S48) Prepared according to general procedure **A1** and **B2** from 4-propylcyclohexanone (10.0 mmol). After purification by flash column chromatography using petroleum ether/dichloromethane (5:1 to 1:1), the title compound was isolated as a white solid (42% yield, 1.82g, two steps from ketone); R_f= 0.4 (PE: DCM = 3:1); ^{1}H NMR (400 MHz, $CDCl_3$) δ 8.00-7.96 (m, 4H), 7.15-7.10 (m, 4H), 2.44-2.40 (m, 2H), 1.88-1.77 (m, 4H), 1.40-1.24 (m, 7H), 0.89 (t, J = 6.7 Hz, 3H); ^{13}C NMR (100 MHz, $CDCl_3$) δ 166.0 (d, J = 255.8 Hz), 162.9, 162.7, 131.99 (d, J = 9.4 Hz), 131.94 (d, J = 9.4 Hz), 123.37 (d, J = 3.2 Hz), 123.27 (d, J = 3.2 Hz), 115.93 (d, J = 22.1 Hz), 115.92 (d, J = 22.1 Hz), 112.8, 38.1, 36.1, 29.7, 28.5, 20.1, 14.2 ppm; ^{19}F NMR (376 MHz, $CDCl_3$) δ -103.92, -103.93; HRMS (ESI-TOF) *m/z* calcd. for $C_{23}H_{24}F_2O_6Na^+$ (M+Na$^+$) 457.1433, found 457.1435.

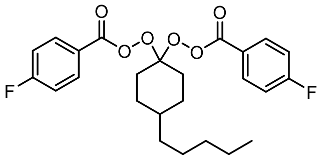

4-Pentylcyclohexane-1,1-diyl bis(4-fluorobenzoperoxoate) (S49) Prepared according to general procedure **A1** and **B2** from 4-pentylcyclohexanone (10.0 mmol). After purification by flash column chromatography using petroleum ether/dichloromethane (5:1 to 1:1), the title compound was isolated as a white solid (40% yield, 1.80 g, two steps from ketone); R_f= 0.4 (PE: DCM = 3:1); ^{1}H NMR (400 MHz, $CDCl_3$) δ 7.93-7.89 (m, 4H), 7.08-7.03 (m, 4H), 2.36-2.33 (m, 2H), 1.78-1.72 (m, 4H), 1.33-1.17 (m, 11H), 0.81 (t, J = 6.6 Hz, 3H); ^{13}C NMR (100 MHz, $CDCl_3$) δ 166.0 (d, J = 255.2 Hz), 162.9, 162.8, 132.02 (d, J = 9.4 Hz), 131.96 (d, J = 9.4 Hz), 123.39 (d, J = 3.2 Hz), 123.29 (d, J = 3.2 Hz), 115.95 (d, J = 22.2 Hz), 115.93 (d, J = 22.2 Hz), 112.9, 36.4, 35.8, 32.0, 29.7, 28.5, 26.7, 22.6, 14.0 ppm; ^{19}F NMR (376 MHz, $CDCl_3$) δ -103.93, -103.94; HRMS (ESI-TOF) *m/z* calcd. for $C_{25}H_{28}F_2O_6Na^+$ (M+Na$^+$) 485.1746, found 485.1743.

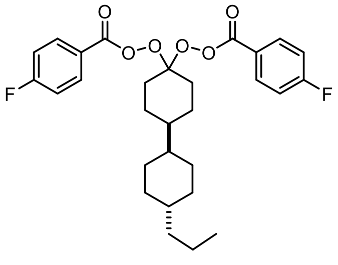

1-{[(4-Fluorophenyl)carbonyl]peroxy}-4-(4-propylcyclohexyl)cyclohexyl (4-fluorophenyl) methanperoxoate (S50) Prepared according to general procedure **A1** and **B2** from 4-propyldicyclohexylanone (10.0 mmol). After purification by flash column chromatography using petroleum ether/ethyl acetate (40:1 to 25:1), the title compound was isolated as a colorless oil (52% yield, 2.67 g, two steps from ketone); R_f= 0.8 (PE: EA = 25:1); ^{1}H NMR (400 MHz, $CDCl_3$) δ 7.99-7.95 (m, 4H), 7.14-7.09 (m, 4H), 2.46-2.40 (m, 2H), 1.85-1.72 (m, 8H), 1.51-1.40 (m, 2H), 1.33-1.09 (m, 7H), 1.03-0.93 (m, 2H), 0.87-0.83 (m, 5H); ^{13}C NMR (100 MHz, $CDCl_3$) δ 166.0 (d, J = 255.2 Hz), 162.9, 162.7, 131.99 (d, J = 9.4 Hz), 131.93 (d, J = 9.4 Hz), 123.36 (d, J = 3.2 Hz), 123.26 (d, J = 3.2 Hz), 115.91 (d, J = 22.2 Hz), 115.89 (d, J = 22.2 Hz), 112.7, 42.2, 42.1, 39.7, 37.4, 33.3, 30.1, 30.0, 25.6, 20.0, 14.3 ppm; ^{19}F NMR (376 MHz, $CDCl_3$) δ -103.90, -103.92. HRMS (ESI-TOF) *m/z* calcd. for $C_{29}H_{34}F_2O_6Na^+$ (M+Na^+) 539.2216, found 539.2225.

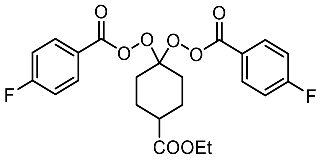

Ethyl 4,4-bis((4-fluorobenzoyl)peroxy)cyclohexane-1-carboxylate (S51) Prepared according to general procedure **A2** and **B2** from ethyl 4-oxocyclohexanecarboxylate (10.0 mmol). After purification by flash column chromatography using petroleum ether/ethyl acetate (20:1 to 10:1), the title compound was isolated as a colorless oil (50% yield, 2.31g, two steps from ketone); R_f= 0.4 (PE: EA = 10:1); ^{1}H NMR (400 MHz, $CDCl_3$) δ 8.00-7.95 (m, 4H), 7.16-7.10 (m, 4H), 4.16 (q, J = 7.2 Hz, 2H), 2.50-2.40 (m, 3H), 2.07-1.94 (m, 6H), 1.27 (t, J = 7.2 Hz, 3H); ^{13}C NMR (100 MHz, $CDCl_3$) δ 174.2, 166.1 (d, J = 255.7 Hz), 162.9, 162.6, 132.08 (d, J = 9.4 Hz), 132.01 (d, J = 9.4 Hz), 123.28, 123.13, 116.0 (d, J = 22.2 Hz), 111.8, 60.7, 41.1, 28.8, 24.5, 14.2 ppm; ^{19}F NMR (376 MHz, $CDCl_3$) δ -103.69, -103.76; HRMS (ESI-TOF) *m/z* calcd. for $C_{23}H_{22}F_2O_8Na^+$ (M+Na^+) 487.1175, found 487.1182.

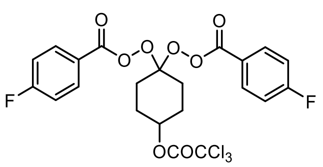

4,4-Bis((4-fluorobenzoyl)peroxy)cyclohexyl 2,2,2-trichloroacetate (S52) Prepared according to general procedure **A2** and **B2** from ketone (10.0 mmol) obtained by the reaction of 4-hydroxycyclohexanone with trichloroacetyl chloride. After purification by flash column chromatography using petroleum ether/ethyl acetate (20:1 to 10:1), the title compound was isolated as a colorless oil (35%

yield, 1.90 g, two steps from ketone); R_f= 0.3 (PE: EA = 10:1); ^{1}H NMR (400 MHz, $CDCl_3$) δ 7.92-7.88 (m, 4H), 7.08-7.02 (m, 4H), 5.15-5.13 (m, 1H), 2.25-2.21 (m, 4H), 2.05-1.95 (m, 4H); ^{13}C NMR (100 MHz, $CDCl_3$) δ 166.05 (d, J = 254.8 Hz), 166.01 (d, J = 254.8 Hz), 162.6, 162.5, 161.1, 131.97 (d, J = 9.5 Hz), 131.94 (d, J = 9.5 Hz), 122.97 (d, J = 3.2 Hz), 122.91 (d, J = 3.2 Hz), 116.00 (d, J = 22.2 Hz), 115.97 (d, J = 22.2 Hz), 111.1, 89.9, 74.5, 26.1, 25.5 ppm; ^{19}F NMR (376 MHz, $CDCl_3$) δ -103.35, -103.48; HRMS (ESI-TOF) *m/z* calcd. for $C_{22}H_{17}Cl_3F_2O_8Na^+$ (M+Na$^+$) 574.9849, found 574.9857.

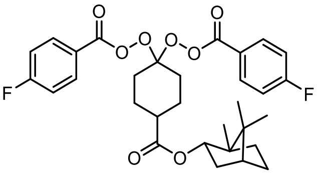

(1s ,2s, 4r)-1,7,7-Trimethylbicyclo[2.2.1]heptan-2-yl 4,4-bis((4-fluorobenzoyl)peroxy)cyclohexane-1-carboxylate (S53) Prepared according to general procedure **A2** and **B2** from ketone (10.0 mmol) obtained by the reaction of 4-hydroxycyclohexanone with borneol. After purification by flash column chromatography using petroleum ether/ethyl acetate (20:1 to 15:1), the title compound was isolated as a colorless oil (41%yield, 2.30 g, two steps from ketone); R_f= 0.5 (PE: EA = 15:1); ^{1}H NMR (400 MHz, $CDCl_3$) δ 7.99-7.95 (m, 4H), 7.14-7.10 (m, 4H), 4.93-4.89 (m, 1H), 2.55-2.52 (m, 1H), 2.42-2.33 (m, 3H), 2.09-1.88 (m, 7H), 1.77-1.67 (m, 2H), 1.34-1.19 (m, 2H), 0.96-0.82 (m, 10H); ^{13}C NMR (100 MHz, $CDCl_3$) δ 174.3, 166.04 (d, J = 255.4 Hz), 166.00 (d, J = 255.4 Hz), 162.8, 162.6, 131.99 (d, J = 9.4 Hz), 131.96 (d, J = 9.4 Hz), 123.22 (d, J = 3.2 Hz), 123.10 (d, J = 3.2 Hz), 115.97 (d, J = 22.1 Hz), 115.95 (d, J = 22.1 Hz), 111.8, 80.0, 48.8, 47.8, 44.8, 41.2, 36.9, 28.70, 28.66, 28.0, 27.1, 24.6, 24.4, 19.6, 18.8, 13.5 ppm; ^{19}F NMR (376 MHz, $CDCl_3$) δ -103.68, -103.78; HRMS (ESI-TOF) *m/z* calcd. for $C_{30}H_{34}F_2O_8Na^+$ (M+Na$^+$) 595.2114, found 595.2087.

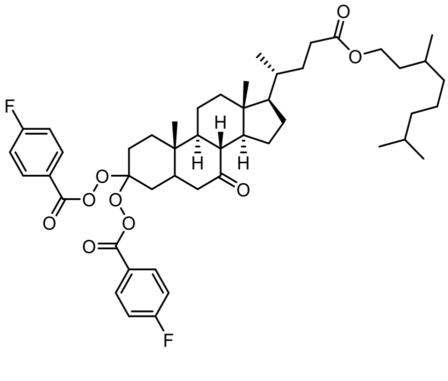

3,7-Dimethyloctyl (4R)-4-((8R,9S,10S,13R,14S,17R)-3,3-bis((4-fluorobenzoyl)peroxy)-10,13-dimethyl-7-oxohexadecahydro-1*H*-cyclopenta[*a*]phenanthren-17-yl)pentanoate (S54) Prepared

according to general procedure **A2** and **B2** from ketone (2.0 mmol) obtained by the reaction of chenodeoxycholic acid with tetrahydrogeraniol. After purification by flash column chromatography using petroleum ether/ethyl acetate (20:1 to 10:1), the title compound was isolated as a colorless oil (55%yield, 0.90 g, two steps from ketone); R_f= 0.6 (PE: EA = 10:1); ^{1}H NMR (400 MHz, $CDCl_3$) δ 7.98-7.93 (m, 4H), 7.16-7.10 (m, 4H), 4.13-4.04 (m, 2H), 2.92-2.88 (m, 1H), 2.44 (t, J = 11.2 Hz, 1H), 2.35-2.17 (m, 4H), 2.03-1.91 (m, 3H), 1.83-1.73 (m, 4H), 1.67-1.59 (m, 3H), 1.57-1.39 (m, 7H), 1.30-1.22 (m, 10H), 1.17-1.08 (m, 5H), 0.93-0.83 (m, 13H), 0.66 (s, 3H); ^{13}C NMR (100 MHz, $CDCl_3$) δ 211.3, 174.3, 166.1 (d, J = 255.8 Hz), 162.7, 132.04 (d, J = 9.5 Hz), 131.98 (d, J = 9.5 Hz), 123.11 (d, J = 3.2 Hz), 123.04 (d, J = 3.2 Hz), 116.02 (d, J = 22.2 Hz), 115.96 (d, J = 22.2 Hz), 112.4, 62.9, 54.8, 49.5, 48.7, 44.5, 43.9, 42.6, 42.4, 39.2, 38.7, 37.1, 35.53, 35.47, 35.2, 31.9, 31.4, 31.3, 31.0, 29.8, 28.2, 27.9, 24.7, 24.6, 24.3, 22.7, 22.6, 21.9, 19.5, 18.3, 12.0 ppm; ^{19}F NMR (376 MHz, $CDCl_3$) δ -103.54, -103.71; HRMS (ESI-TOF) *m/z* calcd. for $C_{48}H_{64}F_2O_9Na^+$ (M+Na^+) 845.4411, found 845.4413.

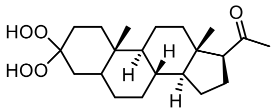

1-((8R,9S,10S,13S,14S)-3,3-Dihydroperoxy-10,13-dimethylhexadecahydro-1*H*-cyclopenta[*a*] phenanthren-17-yl)ethan-1-one (S55) Prepared according to general procedure **A2** from progesterone (2.0 mmol). After purification by flash column chromatography using petroleum ether/ethyl acetate (5:1), the title compound was isolated as a colorless oil (75%yield, 0.54 g); R_f= 0.3 (PE: EA = 4:1); ^{1}H NMR (400 MHz, DMSO-d_6) δ 10.89 (s, 1H), 10.84 (s, 1H), 2.57 (t, J = 8.8 Hz, 1H), 2.06-1.93 (m, 6H), 1.73-1.71 (m, 1H), 1.64-1.50 (m, 5H), 1.45-1.37 (m, 2H), 1.33-1.06 (m, 9H), 0.95-0.68 (m, 5H), 0.51 (s, 3H); ^{13}C NMR (100 MHz, DMSO-d_6) δ 209.0, 108.6, 63.1, 56.4, 53.9, 44.0, 42.1, 38.6, 35.8, 35.4, 34.9, 32.4, 32.0, 31.7, 28.2, 26.0, 24.4, 22.7, 21.2, 13.6, 11.8 ppm; HRMS (ESI-TOF) *m/z* calcd. for $C_{21}H_{34}O_5Na^+$ (M+Na^+) 389.2298, found 389.2297.

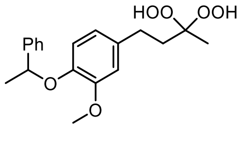

4-(3,3-Dihydroperoxybutyl)-2-methoxy-1-(1-phenylethoxy)benzene (S56) Prepared according to general procedure **A2** from ketone (2.0 mmol) obtained by the reaction of vanillylacetone with (1-bromoethyl)benzene. After purification by flash column chromatography using petroleum ether/ethyl acetate (5:1), the title compound was isolated as a colorless oil (88% yield, 0.61 g); R_f= 0.5 (PE: EA =

5:1); ^{1}H NMR (400 MHz, $CDCl_3$) δ 9.16 (s, 2H), 7.38-7.35 (m, 2H), 7.32-7.28 (m, 2H), 7.24-7.20 (m, 1H), 6.71-6.70 (m, 1H), 6.65-6.61 (m, 1H), 6.55-6.53 (m, 1H), 5.25 (q, J = 6.4 Hz, 1H), 3.85 (s, 3H), 2.63-2.59 (m, 2H), 1.99-1.94 (m, 2H), 1.66 (d, J = 6.4 Hz, 3H), 1.44 (s, 3H); ^{13}C NMR (100 MHz, $CDCl_3$) δ 149.6, 145.4, 142.9, 134.6, 128.5, 127.5, 125.7, 120.3, 116.2, 112.5, 111.7, 77.6, 56.2, 34.8, 29.7, 24.1, 17.9 ppm; HRMS (ESI-TOF) *m/z* calcd. for $C_{19}H_{24}O_6Na^+$ (M+Na^+) 371.1465, found 371.1476.

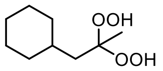

(2,2-Dihydroperoxypropyl)cyclohexane (S57) Prepared according to general procedure **A2** from cyclohexylacetone (2.0 mmol). After purification by flash column chromatography using petroleum ether/ethyl acetate (5:1), the title compound was isolated as a colorless oil (66% yield, 0.25 g); R_f = 0.4 (PE: EA = 5:1); ^{1}H NMR (400 MHz, $CDCl_3$) δ 8.63 (s, 2H), 1.83-1.78 (m, 2H), 1.71-1.62 (m, 4H), 1.60-1.44 (m, 4H), 1.39-1.11 (m, 4H), 1.03-0.89 (m, 2H); ^{13}C NMR (100 MHz, $CDCl_3$) δ 112.5, 40.1, 34.2, 33.5, 26.3, 26.2, 18.1 ppm; HRMS (ESI-TOF) *m/z* calcd. for $C_9H_{18}O_4Na^+$ (M+Na^+) 213.1097, found 213.1107.

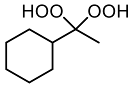

(1,1-Dihydroperoxyethyl)cyclohexane (S58) Prepared according to general procedure **A2** from 1-cyclohexylethan-1-one (2.0 mmol). After purification by flash column chromatography using petroleum ether/ethyl acetate (5:1), the title compound was isolated as a colorless oil (52% yield, 0.18 g); R_f = 0.4 (PE: EA = 5:1); ^{1}H NMR (400 MHz, $CDCl_3$) δ 8.99 (s, 2H), 2.01-1.93 (m, 1H), 1.83-1.75 (m, 4H), 1.71-1.66 (m, 1H), 1.34 (s, 3H), 1.28-1.21 (m, 2H), 1.18-1.03 (m, 3H); ^{13}C NMR (100 MHz, $CDCl_3$) δ 114.6, 40.3, 27.7, 26.3, 26.1, 14.6 ppm; HRMS (ESI-TOF) *m/z* calcd. for $C_8H_{17}O_4^+$ (M+H^+) 177.1121, found 177.1127.

9.2 Characterization data of products

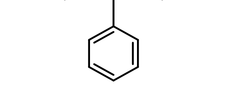

(1,5-Dithiocyanatopentan-3-yl)benzene (1) After purification by flash column chromatography using petroleum ether/ethyl acetate (5:1), the title compound was isolated as a colorless oil (69% yield, 36.2 mg); R_f = 0.5 (PE: EA = 7:1). ^{1}H NMR (400 MHz, $CDCl_3$) δ 7.37-7.33 (m, 2H), 7.29-7.25 (m, 1H), 7.16-7.15 (m, 2H), 2.96-2.88 (m, 1H), 2.84-2.77 (m, 2H), 2.68-2.61 (m, 2H), 2.24-2.13 (m, 4H); ^{13}C NMR

(100 MHz, $CDCl_3$) δ 140.3, 129.4, 127.7, 127.4, 111.8, 42.7, 36.4, 31.6 ppm; HRMS (ESI-TOF) *m/z* calcd. for $C_{13}H_{14}N_2S_2Na^+$ ($M+Na^+$) 285.0491, found 285.0497.

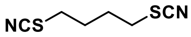

1,4-Dithiocyanatobutane (2) After purification by flash column chromatography using petroleum ether/ethyl acetate (5:1), the title compound was isolated as a colorless oil (46% yield, 15.8 mg); R_f = 0.5 (PE: EA = 5:1); ^{1}H NMR (400 MHz, $CDCl_3$) δ 3.05-2.98 (m, 4H), 2.07-2.00 (m, 4H); ^{13}C NMR (100 MHz, $CDCl_3$) δ 111.6, 33.0, 28.1 ppm; HRMS (ESI-TOF) *m/z* calcd. for $C_6H_8N_2S_2Na^+$ ($M+Na^+$) 195.0021, found 195.0033.

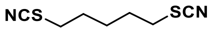

1,5-Dithiocyanatopentane (3) After purification by flash column chromatography using petroleum ether/ethyl acetate (5:1), the title compound was isolated as a colorless oil (82% yield, 30.5 mg); R_f = 0.5 (PE: EA = 5:1); ^{1}H NMR (400 MHz, $CDCl_3$) δ 2.97 (t, J = 7.2 Hz, 4H), 1.93-1.86 (m, 4H), 1.67-1.59 (m, 2H); ^{13}C NMR (100 MHz, $CDCl_3$) δ 112.0, 33.5, 29.1, 26.1 ppm; HRMS (ESI-TOF) *m/z* calcd. for $C_7H_{10}N_2S_2Na^+$ ($M+Na^+$) 209.0178, found 209.0186.

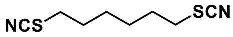

1,6-Dithiocyanatohexane (4) After purification by flash column chromatography using petroleum ether/ethyl acetate (5:1), the title compound was isolated as a colorless oil (76% yield, 30.3 mg); R_f = 0.5 (PE: EA = 5:1); ^{1}H NMR (400 MHz, $CDCl_3$) δ 2.95 (t, J = 7.2 Hz, 4H), 1.90-1.83 (m, 4H), 1.53-1.49 (m, 4H); ^{13}C NMR (100 MHz, $CDCl_3$) δ 112.2, 33.7, 29.6, 27.2 ppm; HRMS (ESI-TOF) *m/z* calcd. for $C_8H_{12}N_2S_2Na^+$ ($M+Na^+$) 223.0334, found 223.0341.

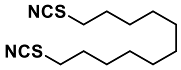

1,11-Dithiocyanatoundecane (5) After purification by flash column chromatography using petroleum ether/ethyl acetate (5:1), the title compound was isolated as a colorless oil (67% yield, 36.0 mg); R_f = 0.6 (PE: EA = 5:1); ^{1}H NMR (400 MHz, $CDCl_3$) δ 2.94 (t, J = 7.2 Hz, 4H), 1.85-1.78 (m, 4H), 1.45-1.39 (m, 4H), 1.35-1.27 (m, 10H); ^{13}C NMR (100 MHz, $CDCl_3$) δ 112.4, 34.0, 29.8, 29.3, 29.2, 28.8, 27.9 ppm; HRMS (ESI-TOF) *m/z* calcd. for $C_{13}H_{22}N_2S_2Na^+$ ($M+Na^+$) 293.1117, found 293.1118.

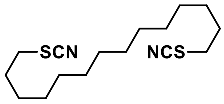

1,14-Dithiocyanatotetradecane (6) After purification by flash column chromatography using petroleum ether/ethyl acetate (5:1), the title compound was isolated as a colorless oil (69% yield, 43.2 mg); R_f= 0.4 (PE: EA = 10:1); ^{1}H NMR (400 MHz, $CDCl_3$) δ 2.93 (t, *J* = 7.2 Hz, 4H), 1.85-1.77 (m, 4H), 1.46-1.39 (m, 4H), 1.33-1.22 (m, 16H); ^{13}C NMR (100 MHz, $CDCl_3$) δ 112.4, 34.0, 29.8, 29.5, 29.4, 29.3, 28.8, 27.9 ppm; HRMS (ESI-TOF) *m*/*z* calcd. for $C_{16}H_{28}N_2S_2Na^+$ (M+Na^+) 335.1586, found 335.1594.

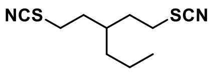

1-Thiocyanato-3-(2-thiocyanatoethyl)hexane (7) After purification by flash column chromatography using petroleum ether/ethyl acetate (5:1), the title compound was isolated as a colorless oil (75% yield, 34.2 mg); R_f= 0.4 (PE: EA =5:1); ^{1}H NMR (400 MHz, $CDCl_3$) δ 3.01-2.90 (m, 4H), 1.87-1.71 (m, 5H), 1.43-1.24 (m, 4H), 0.92 (t, *J* = 6.6 Hz, 3H); ^{13}C NMR (100 MHz, $CDCl_3$) δ 111.9, 34.8, 34.7, 33.5, 31.3, 19.2, 14.2 ppm; HRMS (ESI-TOF) *m*/*z* calcd. for $C_{10}H_{16}N_2S_2Na^+$ (M+Na^+) 251.0647, found 251.0643.

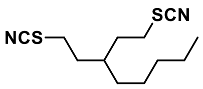

1-Thiocyanato-3-(2-thiocyanatoethyl)octane (8) After purification by flash column chromatography using petroleum ether/ethyl acetate (6:1), the title compound was isolated as a colorless oil (76% yield, 38.9 mg); R_f= 0.6 (PE: EA = 6:1); ^{1}H NMR (400 MHz, $CDCl_3$) δ 3.01-2.91 (m, 4H), 1.90-1.77 (m, 4H), 1.75-1.69 (m, 1H), 1.34-1.24 (m, 8H), 0.89 (t, *J* = 6.6 Hz, 3H); ^{13}C NMR (100 MHz, $CDCl_3$) δ 111.9, 35.1, 33.5, 32.5, 31.9, 31.4, 25.7, 22.5, 14.0 ppm; HRMS (ESI-TOF) *m*/*z* calcd. for $C_{12}H_{20}N_2S_2Na^+$ (M+Na^+) 279.0960, found 279.0966.

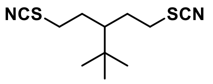

4,4-Dimethyl-1-thiocyanato-3-(2-thiocyanatoethyl)pentane (9) After purification by flash column chromatography using petroleum ether/ethyl acetate (7:1), the title compound was isolated as a colorless oil (54% yield, 26.1 mg); R_f= 0.5 (PE: EA = 7:1); ^{1}H NMR (400 MHz, $CDCl_3$) δ 3.06-2.90 (m, 4H), 2.11-2.02 (m, 2H), 1.64-1.55 (m, 2H), 1.29-1.24 (m, 1H), 0.94 (s, 9H); ^{13}C NMR (100 MHz, $CDCl_3$) δ 112.0, 46.1, 34.2, 33.7, 31.6, 27.5 ppm; HRMS (ESI-TOF) *m*/*z* calcd. for $C_{11}H_{18}N_2S_2Na^+$ (M+Na^+) 265.0804, found 265.0812.

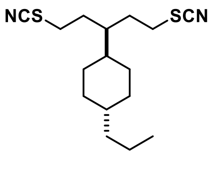

3-((Trans-4-propylcyclohexyl)pentane-1,5-diyl) dithiocyanate (10) After purification by flash column chromatography using petroleum ether/ethyl acetate (10:1), the title compound was isolated as a colorless oil (50% yield, 31.2 mg); R_f= 0.4 (PE: EA = 10:1); ^{1}H NMR (400 MHz, $CDCl_3$) δ 2.96 (m, 4H), 1.97-1.88 (m, 2H), 1.82-1.78 (m, 2H), 1.74-1.65 (m, 2H), 1.61-1.55 (m, 3H), 1.32-1.25 (m, 3H), 1.18-1.06 (m, 5H), 0.88-0.85 (m, 5H); ^{13}C NMR (100 MHz, $CDCl_3$) δ 112.0, 40.4, 39.5, 37.3, 33.1, 32.2, 30.8, 29.0, 19.9, 14.3 ppm; HRMS (ESI-TOF) *m/z* calcd. for $C_{16}H_{26}N_2S_2Na^+$ (M+Na^+) 333.1430, found 333.1429.

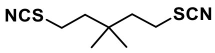

3,3-Dimethyl-1,5-dithiocyanatopentane (11) After purification by flash column chromatography using petroleum ether/ethyl acetate (6:1), the title compound was isolated as a colorless oil (57% yield, 24.5 mg); R_f= 0.4 (PE: EA = 6:1); ^{1}H NMR (400 MHz, $CDCl_3$) δ 2.92-2.88 (m, 4H), 1.81-1.77 (m, 4H), 0.99 (s, 6H); ^{13}C NMR (100 MHz, $CDCl_3$) δ 112.0, 42.2, 34.1, 29.2, 26.3 ppm; HRMS (ESI-TOF) *m/z* calcd. for $C_9H_{14}N_2S_2Na^+$ (M+Na^+) 237.0491, found 237.0482.

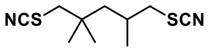

2,2,4-Trimethyl-1,5-dithiocyanatopentane (12) After purification by flash column chromatography using petroleum ether/ethyl acetate (6:1), the title compound was isolated as a colorless oil (52% yield, 23.6 mg); R_f= 0.4 (PE: EA = 6:1); ^{1}H NMR (400 MHz, $CDCl_3$) δ 2.98 (s, 2H), 2.94-2.91 (m, 1H), 2.86-2.80 (m, 1H), 2.00-1.92 (m, 1H), 1.57-1.51 (m, 1H), 1.38-1.30 (m, 1H), 1.15 (d, *J* = 6.6 Hz, 3H), 1.09 (s, 6H). ^{13}C NMR (100 MHz, $CDCl_3$) δ 113.3, 112.3, 47.8, 45.5, 42.6, 36.0, 30.1, 26.0, 25.9, 21.4 ppm; HRMS (ESI-TOF) *m/z* calcd. for $C_{10}H_{16}N_2S_2Na^+$ (M+Na^+) 251.0647, found 251.0651.

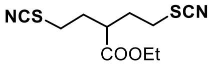

Ethyl 4-thiocyanato-2-(2-thiocyanatoethyl)butanoate (13) After purification by flash column chromatography using petroleum ether/ethyl acetate (5:1), the title compound was isolated as a colorless oil (63% yield, 32.5 mg); R_f= 0.4 (PE: EA =5:1); ^{1}H NMR (400 MHz, $CDCl_3$) δ 4.23-4.17 (m, 2H), 3.07-3.01 (m, 2H), 2.97-2.89 (m, 2H), 2.79-2.72 (m, 1H), 2.29-2.20 (m, 2H), 2.07-1.97 (m, 2H), 1.32-1.28 (m, 3H); ^{13}C NMR (100 MHz, $CDCl_3$) δ 173.0, 111.5, 61.5, 41.9, 32.0, 31.3, 14.2 ppm; HRMS (ESI-TOF) *m/z* calcd. for $C_{10}H_{14}N_2O_2S_2Na^+$ (M+Na^+) 281.0389, found 281.0380.

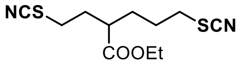

Ethyl 5-thiocyanato-2-(2-thiocyanatoethyl)pentanoate (14) After purification by flash column chromatography using petroleum ether/ethyl acetate (7:1), the title compound was isolated as a colorless oil (50% yield, 27.2 mg), R_f= 0.5 (PE: EA =7:1); ^{1}H NMR (400 MHz, $CDCl_3$) δ 4.25-4.14 (m, 2H), 3.07-2.95 (m, 4H), 2.63-2.56 (m, 1H), 2.27-2.17 (m, 1H), 2.05-1.82 (m, 4H), 1.75-1.65 (m, 1H), 1.29 (t, J = 7.2 Hz, 3H); ^{13}C NMR (100 MHz, $CDCl_3$) δ 173.8, 111.8, 111.6, 61.2, 43.1, 33.5, 32.2, 31.6, 30.2, 27.4, 14.2 ppm; HRMS (ESI-TOF) *m/z* calcd. for $C_{11}H_{16}N_2O_2S_2Na^+$ (M+Na^+) 295.0545, found 295.0550.

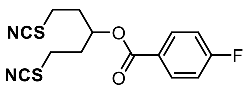

1,5-Dithiocyanatopentan-3-yl 4-fluorobenzoate (15) After purification by flash column chromatography using petroleum ether/ethyl acetate (5:1), the title compound was isolated as a colorless oil (62% yield, 40.3 mg); R_f= 0.4 (PE: EA =5:1); ^{1}H NMR (400 MHz, $CDCl_3$) δ 8.10-8.03 (m, 2H), 7.20-7.13 (m, 2H), 5.46-5.40 (m, 1H), 3.13-2.93 (m, 4H), 2.41-2.20 (m, 4H); ^{13}C NMR (100 MHz, $CDCl_3$) δ 166.3 (d, J = 254.6 Hz), 165.3, 132.5 (d, J = 9.6 Hz), 125.3, 116.1 (d, J = 22.0 Hz), 111.7, 70.7, 35.1, 30.0 ppm; ^{19}F NMR (376 MHz, $CDCl_3$) δ -103.72; HRMS (ESI-TOF) *m/z* calcd. for $C_{14}H_{13}FN_2O_2S_2Na^+$ (M+Na^+) 347.0295, found 347.0290.

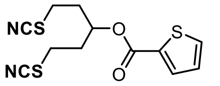

1,5-Dithiocyanatopentan-3-yl thiophene-2-carboxylate (16) After purification by flash column chromatography using petroleum ether/ethyl acetate (5:1), the title compound was isolated as a colorless oil (60% yield, 37.4 mg); R_f= 0.3 (PE: EA =5:1); ^{1}H NMR (400 MHz, $CDCl_3$) δ 7.83 (dd, J = 3.8, 1.6 Hz, 1H), 7.63 (dd, J = 5.0, 1.4 Hz, 1H), 7.16-7.13 (m, 1H), 5.41-5.35 (m, 1H), 3.12-2.93 (m, 4H), 2.36-2.18 (m, 4H); ^{13}C NMR (100 MHz, $CDCl_3$) δ 161.8, 134.4, 133.6, 132.2, 128.2, 111.6, 70.6, 34.9, 29.9 ppm; HRMS (ESI-TOF) *m/z* calcd. for $C_{12}H_{12}N_2O_2S_3Na^+$ (M+Na^+) 334.9953, found 334.9966.

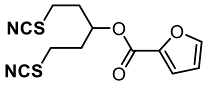

1,5-Dithiocyanatopentan-3-yl furan-2-carboxylate (17) After purification by flash column chromatography using petroleum ether/ethyl acetate (5:1), the title compound was isolated as a colorless oil (72% yield, 42.6 mg); R_f= 0.3 (PE: EA =5:1); ^{1}H NMR (400 MHz, $CDCl_3$) δ 7.62 (d, J = 1.6 Hz, 1H), 7.24 (d, J = 3.6 Hz, 1H), 6.56-6.55 (m, 1H), 5.41-5.37 (m, 1H), 3.11-2.92 (m, 4H), 2.36-2.18 (m, 4H); ^{13}C

NMR (100 MHz, $CDCl_3$) δ 158.2, 147.2, 143.4, 119.3, 112.2, 111.6, 70.4, 34.9, 29.8 ppm; HRMS (ESI-TOF) *m/z* calcd. for $C_{12}H_{12}N_2O_3S_2Na^+$ ($M+Na^+$) 319.0182, found 319.0180.

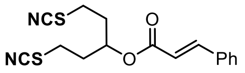

1,5-Dithiocyanatopentan-3-yl cinnamate (18) After purification by flash column chromatography using petroleum ether/ethyl acetate (5:1), the title compound was isolated as a colorless oil (58% yield, 38.5 mg); R_f= 0.4 (PE: EA =5:1); ^1H NMR (400 MHz, $CDCl_3$) δ 7.77-7.71 (m, 1H), 7.57-7.52 (m, 2H), 7.44-7.39 (m, 3H), 6.46-6.40 (m, 1H), 5.36-5.28 (m, 1H), 3.12-2.91 (m, 4H), 2.31-2.14 (m, 4H); ^{13}C NMR (100 MHz, $CDCl_3$) δ 166.6, 146.7, 133.8, 130.9, 129.0, 128.3, 116.5, 111.7, 69.8, 35.0, 29.9 ppm; HRMS (ESI-TOF) *m/z* calcd. for $C_{16}H_{16}N_2O_2S_2Na^+$ ($M+Na^+$) 355.0545, found 355.0559.

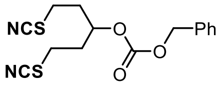

Benzyl (1,5-dithiocyanatopentan-3-yl) carbonate (19) After purification by flash column chromatography using petroleum ether/ethyl acetate (5:1), the title compound was isolated as a colorless oil (53% yield, 35.6 mg); R_f= 0.3 (PE: EA =5:1); ^1H NMR (400 MHz, $CDCl_3$) δ 7.42-7.34 (m, 5H), 5.17 (s, 2H), 5.05-4.99 (m, 1H), 3.06-2.88 (m, 4H), 2.26-2.11 (m, 4H); ^{13}C NMR (100 MHz, $CDCl_3$) δ 154.7, 134.5, 128.9, 128.7, 128.4, 111.5, 73.9, 70.3, 34.6, 29.5 ppm; HRMS (ESI-TOF) *m/z* calcd. for $C_{15}H_{16}N_2O_3S_2Na^+$ ($M+Na^+$) 359.0495, found 359.0496.

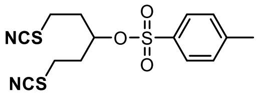

1,5-Dithiocyanatopentan-3-yl 4-methylbenzenesulfonate (20) After purification by flash column chromatography using petroleum ether/ethyl acetate (5:1), the title compound was isolated as a colorless oil (49% yield, 34.8 mg); R_f= 0.3 (PE: EA =5:1); ^1H NMR (400 MHz, $CDCl_3$) δ 7.80 (d, J = 8.2 Hz, 2H), 7.40 (d, J = 8.0 Hz, 2H), 4.91-4.85 (m, 1H), 3.08-2.98 (m, 2H), 2.98-2.83 (m, 2H), 2.47 (s, 3H), 2.22-2.05 (m, 4H); ^{13}C NMR (100 MHz, $CDCl_3$) δ 146.0, 132.8, 130.3, 127.7, 111.4, 34.9, 29.3, 21.7 ppm; HRMS (ESI-TOF) *m/z* calcd. for $C_{14}H_{16}N_2O_3S_3Na^+$ ($M+Na^+$) 379.0215, found 379.0216.

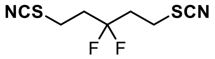

3,3-Difluoro-1,5-dithiocyanatopentane (21) After purification by flash column chromatography using petroleum ether/ethyl acetate (6:1), the title compound was isolated as a colorless oil (61% yield, 27.2 mg); R_f= 0.5 (PE: EA = 6:1); ^1H NMR (400 MHz, $CDCl_3$) δ 3.15-3.11 (m, 4H), 2.50-2.38 (m, 4H); ^{13}C

NMR (100 MHz, $CDCl_3$) δ 121.5 (t, J = 243.0 Hz), 111.2, 37.6 (t, J = 25.0 Hz), 25.9 (t, J = 5.8 Hz) ppm; ^{19}F NMR (376 MHz, $CDCl_3$) δ -100.86 (t, J = 16.5 Hz); HRMS (ESI-TOF) m/z calcd. for $C_7H_8F_2N_2S_2Na^+$ (M+Na$^+$) 244.9989, found 244.9996.

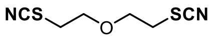

1-Thiocyanato-2-(2-thiocyanatoethoxy)ethane (22) After purification by flash column chromatography using petroleum ether/ethyl acetate (8:1), the title compound was isolated as a colorless oil (80% yield, 30.2 mg); R_f = 0.4 (PE: EA = 8:1); ^{1}H NMR (400 MHz, $CDCl_3$) δ 3.87 (t, J = 5.8 Hz, 4H), 3.17 (t, J = 5.8 Hz, 4H); ^{13}C NMR (100 MHz, $CDCl_3$) δ 111.9, 68.9, 33.5 ppm; HRMS (ESI-TOF) m/z calcd. for $C_6H_8N_2OS_2Na^+$ (M+Na$^+$) 210.9970, found 210.9985.

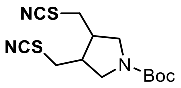

Tert-butyl 3,4-bis(thiocyanatomethyl)pyrrolidine-1-carboxylate (23) After purification by flash column chromatography using petroleum ether/ethyl acetate (5:1), the title compound was isolated as a colorless oil (32% yield, 20.1 mg, dr could not be determined by ^{1}H NMR due to peak overlapping); R_f = 0.4 (PE: EA = 5:1); ^{1}H NMR (400 MHz, $CDCl_3$) δ 3.70-3.60 (m, 2H), 3.39-3.24 (m, 2H), 3.17-3.09 (m, 2H), 2.92-2.81 (m, 4H), 1.47 (s, 9H); ^{13}C NMR (100 MHz, $CDCl_3$) δ 154.2, 110.8, 80.4, 49.1, 48.8, 42.4, 40.7, 32.4, 28.4 ppm; HRMS (ESI-TOF) m/z calcd. for $C_{13}H_{19}N_3O_2S_2Na^+$ (M+Na$^+$) 336.0811, found 336.0810.

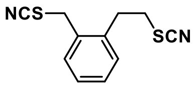

1-(2-Thiocyanatoethyl)-2-(thiocyanatomethyl)benzene (24) After purification by flash column chromatography using petroleum ether/ethyl acetate (8:1), the title compound was isolated as a colorless oil (39% yield, 18.3 mg); R_f = 0.4 (PE: EA = 8:1); ^{1}H NMR (400 MHz, $CDCl_3$) δ 7.40-7.32 (m, 3H), 7.29-7.27 (m, 1H), 4.25 (s, 2H), 3.22 (s, 4H); ^{13}C NMR (100 MHz, $CDCl_3$) δ 136.4, 132.1, 131.3, 130.3, 129.9, 128.2, 111.8, 111.2, 35.6, 34.2, 32.7 ppm; HRMS (ESI-TOF) m/z calcd. for $C_{11}H_{10}N_2S_2Na^+$ (M+Na$^+$) 257.0178, found 257.0176.

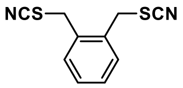

1,2-Bis(thiocyanatomethyl)benzene (25) After purification by flash column chromatography using petroleum ether/ethyl acetate (8:1), the title compound was isolated as a colorless oil (45% yield, 20.0 mg); R$_f$= 0.4 (PE: EA = 8:1); ^{1}H NMR (400 MHz, CDCl$_3$) δ 7.45-7.40 (m, 4H), 4.30 (s, 4H); ^{13}C NMR (100 MHz, CDCl$_3$) δ 132.6, 131.6, 130.1, 111.1, 35.1 ppm; HRMS (ESI-TOF) *m*/*z* calcd. for $C_{10}H_8N_2S_2Na^+$ (M+Na$^+$) 243.0021, found 243.0024.

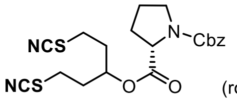

(rotamers 2.3:1)

1-Benzyl 2-(1,5-dithiocyanatopentan-3-yl) (S)-pyrrolidine-1,2-dicarboxylate (26) After purification by flash column chromatography using petroleum ether/ethyl acetate (4:1), the title compound was isolated as a colorless oil (47% yield, 40.7 mg); R$_f$= 0.2 (PE: EA =4:1); ^{1}H NMR (400 MHz, CDCl$_3$) δ 7.38-7.30 (m, 5H), 5.28-5.01 (m, 3H), 4.36-4.34 (m, 0.3H), 4.31-4.28 (m, 0.7H), 3.65-3.49 (m, 2H), 3.10-2.83 (m, 3H), 2.77-2.42 (m, 1H), 2.35-2.24 (m, 1H), 2.15-1.95 (m, 7H); ^{13}C NMR (100 MHz, CDCl$_3$) δ 172.8, 172.5, 154.8, 154.0, 136.5, 136.1, 128.6, 128.5, 128.4, 128.1, 128.0, 127.8, 112.1, 111.7, 111.6, 111.4, 70.5, 70.1, 67.2, 67.1, 59.3, 58.6, 47.0, 46.5, 34.9, 34.7, 34.6, 31.1, 30.0, 29.7, 29.6, 29.5, 29.3, 24.6, 23.5 ppm; HRMS (ESI-TOF) *m*/*z* calcd. for $C_{20}H_{23}N_3O_4S_2Na^+$ (M+Na$^+$) 456.1022, found 456.1027.

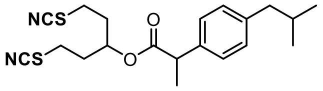

1,5-Dithiocyanatopentan-3-yl 2-(4-isobutylphenyl)propanoate (27) After purification by flash column chromatography using petroleum ether/ethyl acetate (7:1), the title compound was isolated as a colorless oil (58% yield, 45.2 mg); R$_f$= 0.4 (PE: EA =7:1); ^{1}H NMR (400 MHz, CDCl$_3$) δ 7.19-7.11 (m, 4H), 5.11-5.05 (m, 1H), 3.69 (q, *J* = 7.2 Hz, 1H), 2.95-2.77 (m, 2H), 2.51-2.45 (m, 3H), 2.25-2.04 (m, 3H), 2.00-1.77 (m, 3H), 1.50 (d, *J* = 7.0 Hz, 3H), 0.89 (d, *J* = 6.6 Hz, 6H); ^{13}C NMR (100 MHz, CDCl$_3$) δ 174.5, 141.4, 137.4, 129.8, 127.1, 111.8, 111.6, 69.7, 45.3, 45.0, 35.0, 34.6, 30.3, 30.0, 29.0, 22.4, 17.5 ppm; HRMS (ESI-TOF) *m*/*z* calcd. for $C_{20}H_{26}N_2O_2S_2Na^+$ (M+Na$^+$) 413.1328, found 413.1321.

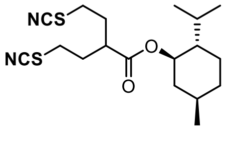

(2r, 5s)-2-Isopropyl-5-methylcyclohexyl 4-thiocyanato-2-(2-thiocyanatoethyl)butanoate (28) After purification by flash column chromatography using petroleum ether/ethyl acetate (5:1), the title compound was isolated as a colorless oil (77% yield, 56.6 mg); R$_f$= 0.4 (PE: EA =5:1); ^{1}H NMR (400

MHz, $CDCl_3$) δ 4.70 (td, *J* = 10.8, 4.2 Hz, 1H), 3.05-2.83 (m, 4H), 2.77-2.70 (m, 1H), 2.27-2.16 (m, 2H), 2. 05-1.91 (m, 3H), 1.82-1.74 (m, 1H), 1.70-1.66 (m, 2H), 1.53-1.38 (m, 2H), 1.08-0.82 (m, 9H), 0.74 (d, *J* = 7.0 Hz, 3H); ^{13}C NMR (100 MHz, $CDCl_3$) δ 172.5, 111.4, 75.6, 46.7, 42.3, 40.7, 33.9, 32.2, 31.8, 31.3, 31.2, 26.3, 23.0, 21.9, 20.7, 15.9 ppm; HRMS (ESI-TOF) *m/z* calcd. for $C_{18}H_{28}N_2O_2S_2Na^+$ (M+Na^+) 391.1484, found 391.1471.

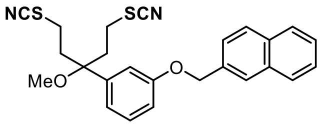

2-((3-(3-Methoxy-1,5-dithiocyanatopentan-3-yl)phenoxy)methyl)naphthalene (29) After purification by flash column chromatography using petroleum ether/ethyl acetate (10:1), the title compound was isolated as a colorless oil (39% yield, 35.1 mg); R_f= 0.5 (PE: EA =10:1); ^1H NMR (400 MHz, $CDCl_3$) δ 7.91-7.85 (m, 4H), 7.58-7.49 (m, 3H), 7.34-7.30 (m, 1H), 7.05-7.04 (m, 1H), 6.98-6.95 (m, 1H), 6.92-6.90 (m, 1H), 5.26 (s, 2H), 3.27 (s, 3H), 2.76-2.69 (m, 2H), 2.52-2.39 (m, 4H), 2.21-2.14 (m, 2H); ^{13}C NMR (100 MHz, $CDCl_3$) δ 159.0, 142.3, 134.1, 133.2, 133.0, 130.0, 128.4, 127.8, 127.7, 126.5, 126.3, 126.1, 125.3, 117.7, 113.9, 112.9, 112.0, 79.5, 70.1, 49.4, 37.4, 27.8 ppm; HRMS (ESI-TOF) *m/z* calcd. for $C_{25}H_{24}N_2O_2S_2Na^+$ (M+Na^+) 471.1171, found 471.1167.

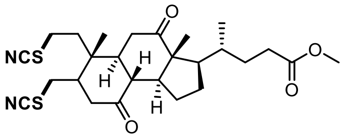

Methyl (4r)-4-((3R,3aR,5aS,6R,9aR,9bS)-3a,6-dimethyl-4,9-dioxo-6-(2-thiocyanatoethyl)-7-(thiocyanatomethyl)dodecahydro-1*H*-cyclopenta[*a*]naphthalen-3-yl)pentanoate (30) After purification by flash column chromatography using petroleum ether/ethyl acetate (3:1), the title compound was isolated as a colorless oil (49% yield, 49.6 mg); R_f= 0.4 (PE: EA = 3:1); ^1H NMR (400 MHz, $CDCl_3$) δ 3.65 (s, 3H), 3.20 (d, *J* = 12.1 Hz, 1H), 2.97-2.92 (m, 2H), 2.89-2.79 (m, 2H), 2.77-2.65 (m, 1H), 2.42-2.29 (m, 1H), 2.29-2.13 (m, 4H), 2.05-1.89 (m, 3H), 1.84-1.76 (m, 3H), 1.71-1.58 (m, 2H), 1.49 (s, 3H), 1.40-1.33 (m, 2H), 1.31-1.20 (m, 3H), 1.04 (s, 3H), 0.82 (d, *J* = 6.4 Hz, 3H). ^{13}C NMR (100 MHz, $CDCl_3$) δ 211.0, 208.0, 174.5, 111.7, 111.4, 56.7, 51.6, 51.5, 51.2, 49.2, 45.8, 45.6, 40.5, 40.4, 38.9, 38.5, 35.4, 32.7, 31.2, 30.3, 27.8, 27.5, 25.0, 19.5, 18.5, 11.8 ppm; HRMS (ESI-TOF) *m/z* calcd. for $C_{26}H_{36}N_2O_4S_2Na^+$ (M+Na^+) 527.2009, found 527.2017.

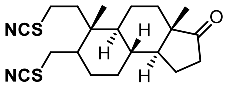

(3aS,5aS,6R,9aR,9bS)-3a,6-Dimethyl-6-(2-thiocyanatoethyl)-7-(thiocyanatomethyl)dodecahydro-3*H*-cyclopenta[*a*]naphthalen-3-one (31) After purification by flash column chromatography using petroleum ether/ethyl acetate (5:1), the title compound was isolated as a colorless oil (49% yield, 37.5mg); R_f= 0.4 (PE: EA = 5:1); ^{1}H NMR (400 MHz, $CDCl_3$) δ 3.37 (d, *J* = 13.2 Hz, 1H), 2.93-2.88 (m, 1H), 2.84-2.77 (m, 1H), 2.49-2.37 (m, 2H), 2.09-1.85 (m, 8H), 1.70-1.65 (m, 1H), 1.57-1.45 (m, 3H), 1.34-1.22 (m, 4H), 1.02-0.94 (m, 1H), 0.88 (s, 3H), 0.85 (s, 3H). ^{13}C NMR (100 MHz, $CDCl_3$) δ 220.1, 112.7, 111.9, 51.2, 47.8, 47.3, 43.5, 40.4, 36.9, 36.0, 35.7, 34.6, 31.3, 29.9, 27.2, 25.7, 21.7, 20.4, 16.0, 13.6 ppm; HRMS (ESI-TOF) *m/z* calcd. for $C_{20}H_{28}N_2OS_2Na^+$ (M+Na$^+$) 399.1535, found 399.1544.

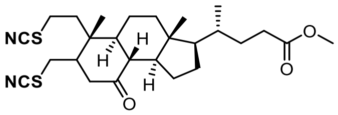

Methyl (4R)-4-((3R,3aR,5aS,6R,9aS,9bS)-3a,6-dimethyl-9-oxo-6-(2-thiocyanatoethyl)-7-(thiocyanatomethyl)dodecahydro-1*H*-cyclopenta[*a*]naphthalen-3-yl)pentanoate (32) After purification by flash column chromatography using petroleum ether/ethyl acetate (4:1), the title compound was isolated as a colorless oil (50% yield, 49.1 mg); R_f= 0.4 (PE: EA = 4:1) and detected by I_2; ^{1}H NMR (400 MHz, $CDCl_3$) δ 3.65 (s, 3H), 3.20 (d, *J* = 13.2 Hz, 1H), 2.97-2.91 (m, 2H), 2.82-2.78 (m, 1H), 2.59-2.56 (m, 1H), 2.48 (t, *J* = 11.3 Hz, 1H), 2.36-2.31 (m, 2H), 2.26-2.07 (m, 4H), 2.04-1.99 (m, 1H), 1.97-1.89 (m, 1H), 1.83-1.76 (m, 2H), 1.62-1.55 (m, 3H), 1.42-1.27 (m, 7H), 1.15-1.07 (m, 2H), 1.00-0.90 (m, 4H), 0.66 (s, 3H). ^{13}C NMR (100 MHz, $CDCl_3$) δ 210.4, 174.6, 112.0, 111.7, 54.7, 51.5, 49.9, 49.8, 48.5, 46.6, 42.3, 40.4, 39.9, 39.2, 38.6, 35.1, 32.9, 31.0, 30.9, 28.1, 28.0, 24.6, 22.2, 20.1, 18.3, 12.0 ppm; HRMS (ESI-TOF) *m/z* calcd. for $C_{26}H_{38}N_2O_3S_2Na^+$ (M+Na$^+$) 513.2216, found 513.2225.

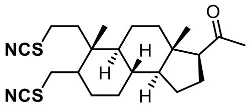

1-((3aS,5aS,6R,9aS,9bS)-3a,6-Dimethyl-6-(2-thiocyanatoethyl)-7-(thiocyanatomethyl)dodecahydro-1*H*-cyclopenta[*a*]naphthalen-3-yl)ethan-1-one (33) After purification by flash column chromatography using petroleum ether/ethyl acetate (4:1), the title compound was isolated as a colorless oil (68% yield, 55.0 mg); R_f= 0.6 (PE: EA = 4:1); ^{1}H NMR (400 MHz, $CDCl_3$) δ 3.35 (d, *J* = 13.2 Hz, 1H), 2.91-2.84 (m, 2H), 2.55-2.50 (m, 1H), 2.37 (t, *J* = 12.2 Hz, 1H), 2.20-1.99 (m, 8H), 1.91-1.83 (m, 1H), 1.83-1.76 (m, 1H), 1.71-1.62 (m, 3H), 1.48-1.35 (m, 2H), 1.27-1.12 (m, 4H), 0.97-0.85 (m, 5H), 0.60 (s, 3H). ^{13}C NMR (100 MHz, $CDCl_3$) δ 209.2, 112.8, 112.0,

63.5, 56.4, 47.4, 43.7, 43.4, 40.3, 38.6, 36.9, 36.2, 35.0, 31.5, 31.0, 27.4, 25.9, 24.3, 22.7, 21.1, 16.0, 13.2 ppm; HRMS (ESI-TOF) *m/z* calcd. for $C_{22}H_{32}N_2OS_2Na^+$ (M+Na^+) 427.1848, found 427.1859.

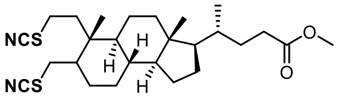

Methyl (4R)-4-((3R,3aR,5aS,6R,9aS,9bS)-3a,6-dimethyl-6-(2-thiocyanatoethyl)-7-(thiocyanatom - ethyl)dodecahydro-1*H*-cyclopenta[*a*]naphthalen-3-yl)pentanoate (34) After purification by flash column chromatography using petroleum ether/ethyl acetate (5:1), the title compound was isolated as a colorless oil (44% yield, 41.5mg); R_f= 0.6 (PE: EA = 5:1); ^{1}H NMR (400 MHz, $CDCl_3$) δ 3.65 (s, 3H), 3.15 (d, *J* = 13.2 Hz, 1H), 2.97-2.86 (m, 3H), 2.38-2.31 (m, 1H), 2.28-2.17 (m, 1H), 2.06-1.96 (m, 2H), 1.88-1.69 (m, 6H), 1.61-1.41 (m, 6H), 1.39-1.23 (m, 4H), 1.16-1.03 (m, 7H), 0.90 (d, *J* = 6.4 Hz, 3H), 0.65 (s, 3H). ^{13}C NMR (100 MHz, $CDCl_3$) δ 174.7, 112.3, 112.0, 56.0, 55.8, 51.5, 47.0, 42.4, 41.8, 39.8, 39.7, 39.5, 35.33, 35.29, 32.6, 31. 0, 30.9, 28.4, 28.0, 24.9, 24.0, 21.9, 21.5, 20.0, 18.2, 12.0 ppm; HRMS (ESI-TOF) *m/z* calcd. for $C_{26}H_{40}N_2O_2S_2Na^+$ (M+Na^+) 499.2423, found 499.2434.

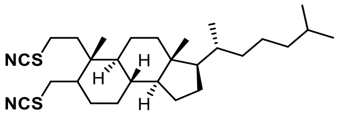

(3R,3aR,5aS,6R,9aS,9bS)-3a,6-Dimethyl-3-((R)-6-methylheptan-2-yl)-6-(2-thiocyanatoethyl)-7-(thiocyanatomethyl)dodecahydro-1*H*-cyclopenta[*a*]naphthalene (35) After purification by flash column chromatography using petroleum ether/ethyl acetate (10:1), the title compound was isolated as a colorless oil (50% yield, 47.6 mg); R_f= 0.5 (PE: EA = 10:1); ^{1}H NMR (400 MHz, $CDCl_3$) δ 3.35 (d, *J* = 10.8 Hz, 1H), 2.89-2.84 (m, 2H), 2.41-2.35 (m, 1H), 2.06-1.98 (m, 3H), 1.88-1.73 (m, 3H), 1.65-1.48 (m, 4H), 1.43-1.20 (m, 9H), 1.14-1.07 (m, 5H), 1.04-0.94 (m, 3H), 0.91-0.81 (m, 12H), 0.64 (s, 3H). ^{13}C NMR (100 MHz, $CDCl_3$) δ 112.9, 112.0, 56.3, 56.1, 47.6, 43.4, 42.2, 40.2, 39.6, 39.4, 36.9, 36.3, 36.0, 35.7, 35.0, 31.0, 28.1, 27.9, 27.5, 26.0, 24.1, 23.8, 22.8, 22.5, 21.1, 18.6, 16.0, 11.8 ppm; HRMS (ESI-TOF) *m/z* calcd. for $C_{28}H_{46}N_2S_2Na^+$ (M+Na^+) 497.2995, found 497.3004.

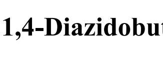

1,4-Diazidobutane (36) After purification by flash column chromatography using petroleum ether/dichloromethane (5:1), the title compound was isolated as a colorless oil (58% yield, 16.2 mg); R_f = 0.8 (PE: DCM = 5:1); ^{1}H NMR (400 MHz, $CDCl_3$) δ 3.33-3.31 (m, 4H), 1.69-1.67 (m, 4H); ^{13}C NMR

(100 MHz, $CDCl_3$) δ 50.9, 26.1 ppm; HRMS (ESI-TOF) *m/z* calcd. for $C_4H_8N_6Na^+$ ($M+Na^+$) 163.0703, found 163.0710.

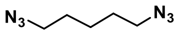

1,5-Diazidopentane (37) After purification by flash column chromatography using petroleum ether/dichloromethane (5:1), the title compound was isolated as a colorless oil (65% yield, 20.0 mg); R_f = 0.8 (PE: DCM = 5:1); ^{1}H NMR (400 MHz, $CDCl_3$) δ 3.29 (t, *J* = 6.8 Hz, 4H), 1.67-1.58 (m, 4H), 1.50-1.42 (m, 2H); ^{13}C NMR (100 MHz, $CDCl_3$) δ 51.2, 28.4, 23.9 ppm; HRMS (ESI-TOF) *m/z* calcd. for $C_5H_{10}N_6Na^+$ ($M+Na^+$) 177.0859, found 177.0866.

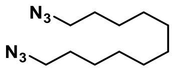

1,11-Diazidoundecane (38) After purification by flash column chromatography using petroleum ether/dichloromethane (5:1), the title compound was isolated as a colorless oil (77% yield, 36.6 mg); R_f = 0.9 (PE: DCM = 5:1); ^{1}H NMR (400 MHz, $CDCl_3$) δ 3.25 (t, *J* = 7.0 Hz, 4H), 1.63-1.56 (m, 4H), 1.38-1.25 (m, 14H); ^{13}C NMR (100 MHz, $CDCl_3$) δ 51.4, 29.4, 29.1, 28.8, 26.7 ppm; HRMS (ESI-TOF) *m/z* calcd. for $C_{11}H_{22}N_6Na^+$ ($M+Na^+$) 261.1798, found 261.1790.

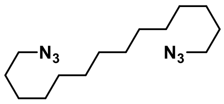

1,14-Diazidotetradecane (39) After purification by flash column chromatography using petroleum ether/dichloromethane (5:1), the title compound was isolated as a colorless oil (35% yield, 19.7 mg); R_f = 0.8 (PE: DCM = 5:1); ^{1}H NMR (400 MHz, $CDCl_3$) δ 3.25 (t, *J* = 6.9 Hz, 4H), 1.63-1.56 (m, 4H), 1.41-1.26 (m, 20H); ^{13}C NMR (100 MHz, $CDCl_3$) δ 51.5, 29.6, 29.50, 29.45, 29.1, 28.8, 26.7 ppm; HRMS (ESI-TOF) *m/z* calcd. for $C_{14}H_{28}N_6Na^+$ ($M+Na^+$) 303.2268, found 303.2272.

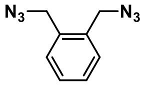

1,2-Bis(azidomethyl)benzene (40) After purification by flash column chromatography using petroleum ether/dichloromethane (5:1), the title compound was isolated as a colorless oil (46% yield, 17.3 mg); R_f = 0.6 (PE: DCM = 5:1); ^{1}H NMR (400 MHz, $CDCl_3$) δ 7.41-7.35 (m, 4H), 4.44 (s, 4H); ^{13}C NMR (100 MHz, $CDCl_3$) δ 133.8, 130.1, 129.0, 52.2 ppm; HRMS (ESI-TOF) *m/z* calcd. for $C_8H_8N_6Na^+$ ($M+Na^+$) 211.0703, found 211.0709.

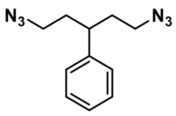

(1,5-Diazidopentan-3-yl)benzene (41) After purification by flash column chromatography using petroleum ether/dichloromethane (4:1), the title compound was isolated as a colorless oil (62% yield, 28.5 mg); R_f = 0.8 (PE: DCM = 5:1); ^{1}H NMR (400 MHz, $CDCl_3$) δ 7.35-7.31 (m, 2H), 7.26-7.22 (m, 1H), 7.16-7.14 (m, 2H), 3.20-3.13 (m, 2H), 3.07-3.00 (m, 2H), 2.84-2.77 (m, 1H), 1.97-1.86 (m, 4H); ^{13}C NMR (100 MHz, $CDCl_3$) δ 142.1, 128.9, 127.5, 127.0, 49.3, 40.2, 35.6 ppm; HRMS (ESI-TOF) *m*/*z* calcd. for $C_{11}H_{14}N_6Na^+$ (M+Na$^+$) 253.1172, found 253.1180.

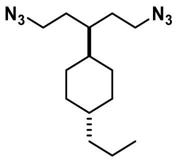

1-((Trans-4-propylcyclohexyl)-1,5-diazidopentane (42) After purification by flash column chromatography using petroleum ether/dichloromethane (5:1), the title compound was isolated as a colorless oil (50% yield, 27.8 mg); R_f = 0.7 (PE: DCM = 5:1); ^{1}H NMR (400 MHz, $CDCl_3$) δ 3.34-3.23 (m, 4H), 1.80-1.76 (m, 2H), 1.69-1.63 (m, 2H), 1.59-1.56 (m, 2H), 1.49-1.37 (m, 3H), 1.33-1.25 (m, 3H), 1.15-1.11 (m, 3H), 1.07-1.02 (m, 2H), 0.91-0.81 (m, 5H); ^{13}C NMR (100 MHz, $CDCl_3$) δ 49.9, 40.0, 39.6, 37.9, 37.5, 33.3, 29.9, 29.1, 20.0, 14.4 ppm; HRMS (ESI-TOF) *m*/*z* calcd. for $C_{14}H_{26}N_6Na^+$ (M+Na$^+$) 301.2111, found 301.2106.

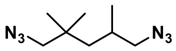

1,5-Diazido-2,2,4-trimethylpentane (43) After purification by flash column chromatography using petroleum ether/dichloromethane (5:1), the title compound was isolated as a colorless oil (61% yield, 24.1 mg); R_f = 0.7 (PE: DCM = 4:1); ^{1}H NMR (400 MHz, $CDCl_3$) δ 3.20-3.17 (m, 1H), 3.12-3.09 (m, 3H), 1.79-1.71 (m, 1H), 1.37-1.33 (m, 1H), 1.14-1.09 (m, 1H), 1.01 (d, *J* = 6.6 Hz, 3H), 0.95 (s, 6H); ^{13}C NMR (100 MHz, $CDCl_3$) δ 62.8, 59.1, 43.4, 35.8, 29.5, 25.3, 25.2, 20.5 ppm; HRMS (ESI-TOF) *m*/*z* calcd. for $C_8H_{16}N_6Na^+$ (M+Na$^+$) 219.1329, found 219.1340.

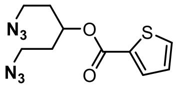

1,5-Diazidopentan-3-yl thiophene-2-carboxylate (44) After purification by flash column chromatography using petroleum ether/ethyl acetate (20:1), the title compound was isolated as a colorless

oil (31% yield, 17.4 mg); R_f= 0.4 (PE: EA = 20:1); ^{1}H NMR (400 MHz, $CDCl_3$) δ 7.60 (dd, *J* = 1.8, 0.8 Hz , 1H), 7.21 (dd, *J* = 3.5, 0.8 Hz, 1H), 6.53 (dd, *J* = 3.6, 1.6 Hz, 1H), 5.33-5.27 (m, 1H), 3.45-3.35 (m, 4H), 2.06-1.89 (m, 4H); ^{13}C NMR (100 MHz, $CDCl_3$) δ 158.2, 146.7, 144.2, 118.6, 112.1, 69.8, 47.7, 33.6 ppm; HRMS (ESI-TOF) *m*/*z* calcd. for $C_{10}H_{12}N_6O_2SNa^+$ (M+Na^+) 303.0635, found 303.0610.

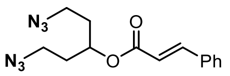

1,5-Diazidopentan-3-yl cinnamate (45) After purification by flash column chromatography using petroleum ether/dichloromethane (3:1), the title compound was isolated as a colorless oil (35% yield, 21.1 mg); R_f= 0.3 (PE: DCM = 3:1); ^{1}H NMR (400 MHz, $CDCl_3$) δ 7.72 (d, *J* = 16.0 Hz, 1H), 7.56-7.52 (m, 2H), 7.41-7.39 (m, 3H), 6.44 (d, *J* = 16.0 Hz, 1H), 5.27-5.17 (m, 1H), 3.47-3.35 (m, 4H), 2.01-1.84 (m, 4H); ^{13}C NMR (100 MHz, $CDCl_3$) δ 166.4, 145.7, 134.1, 130.5, 128.9, 128.2, 117.4, 69.0, 47.7, 33.6 ppm; HRMS (ESI-TOF) *m*/*z* calcd. for $C_{14}H_{16}N_6O_2Na^+$ (M+Na^+) 323.1227, found 323.1234.

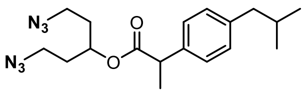

1,5-Diazidopentan-3-yl 2-(4-isobutylphenyl)propanoate (46) After purification by flash column chromatography using petroleum ether/dichloromethane (2:1), the title compound was isolated as a colorless oil (51% yield, 36.5 mg); R_f= 0.3 (PE: DCM = 2:1); ^{1}H NMR (400 MHz, $CDCl_3$) δ 7.21-7.19 (m, 2H), 7.12-7.10 (m, 2H), 5.04-4.98 (m, 1H), 3.68 (q, *J* = 7.0 Hz, 1H), 3.29 -3.20 (m, 2H), 3.00-2.81 (m, 2H), 2.45 (d, *J* = 7.2 Hz, 2H), 1.88-1.83 (m, 3H), 1.69 (q, *J* = 6.7 Hz, 2H), 1.50 (d, *J* = 7.1 Hz, 3H), 0.88 (d, *J* = 6.6 Hz, 6H); ^{13}C NMR (100 MHz, $CDCl_3$) δ 174.2, 140.9, 137.5, 129.4, 127.0, 68.8, 47.6, 47.1, 45.2, 44.9, 33.5, 33.4, 30.1, 22.3, 17.8 ppm; HRMS (ESI-TOF) *m*/*z* calcd. for $C_{18}H_{26}N_6O_2Na^+$ (M+Na^+) 381.2009, found 381.2021.

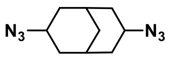

3,7-Diazidobicyclo[3.3.1]nonane (47) After purification by flash column chromatography using petroleum ether/dichloromethane (10:1 to 5:1), the title compound was isolated as a colorless oil (28% yield, 11.5 mg); R_f= 0.6 (PE: DCM = 5:1); ^{1}H NMR (400 MHz, $CDCl_3$) δ 3.76-3.67 (m, 2H), 2.24 (s, 2H), 2.07-2.02 (m, 4H), 1.62-1.55 (m, 4H), 1.52-1.50 (m, 2H); ^{13}C NMR (100 MHz, $CDCl_3$) δ 56.8, 36.5, 33.1, 29.1 ppm; HRMS (ESI-TOF) *m*/*z* calcd. for $C_9H_{14}N_6Na^+$ (M+Na^+) 229.1172, found 229.1176.

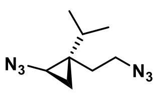

(1R,2R)-2-Azido-1-(2-azidoethyl)-1-isopropylcyclopropane (48) After purification by flash column chromatography using petroleum ether/dichloromethane (10:1 to 4:1), the title compound was isolated as a colorless oil (53% yield, 20.4 mg); R_f= 0.7 (PE: DCM = 4:1); ^{1}H NMR (400 MHz, $CDCl_3$) δ 3.38-3.25 (m, 2H), 1.86 (t, J = 7.6 Hz, 2H), 1.61-1.57 (m, 1H), 1.51-1.44 (m, 1H), 1.23 (t, J = 5.4 Hz, 1H), 1.12-1.09 (m, 1H), 0.94 (d, J = 6.8 Hz, 3H), 0.89 (d, J = 7.0 Hz, 3H); ^{13}C NMR (100 MHz, $CDCl_3$) δ 49.8, 36.9, 33.9, 27.0, 26.7, 20.8, 19.4, 19.1 ppm; HRMS (ESI-TOF) *m/z* calcd. for $C_8H_{14}N_6Na^+$ (M+Na^+) 217.1172, found 217.1168.

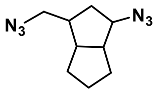

1-Azido-3-(azidomethyl)octahydropentalene (49) After purification by flash column chromatography using petroleum ether/dichloromethane (5:1), the title compound was isolated as a colorless oil (36% yield, 16.6 mg, dr = 2.3:1, dr was determined by ^{1}H NMR analysis); R_f= 0.6 (PE: DCM = 5:1); ^{1}H NMR (400 MHz, $CDCl_3$) δ 4.06-4.02 (m, 0.3H), 3.41-3.22 (m, 2.7H), 2.67-2.62 (m, 0.3 H), 2.46-2.39 (m, 0.7 H), 2.28-2.21 (m, 0.7 H), 2.16-2.08 (m, 1H), 1.99-1.86 (m, 0.7 H), 1.76-1.52 (m, 5.3H), 1.49-1.38 (m, 2.3 H); ^{13}C NMR (100 MHz, $CDCl_3$) δ 67.6, 65.0, 55.5, 55.4, 49.4, 47.5, 47.3, 46.4, 44.5, 44.0, 37.1, 32.6, 32.3, 31.6, 27.5, 27.2, 25.3 ppm; HRMS (ESI-TOF) *m/z* calcd. for $C_9H_{14}N_6Na^+$ (M+Na^+) 229.1172, found 229.1170.

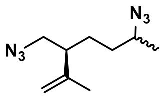

(S)-6-Azido-3-(azidomethyl)-2-methylhept-1-ene (50) After purification by flash column chromatography using petroleum ether/dichloromethane (5:1 to 3:1), the title compound was isolated as a colorless oil (34% yield, 14.2 mg, dr could not be determined by ^{1}H NMR due to peak overlapping); R_f = 0.7 (PE: DCM = 3:1); ^{1}H NMR (400 MHz, $CDCl_3$) δ 4.92 (s, 1H), 4.84 (s, 1H), 3.44-3.38 (m, 1H), 3.27-3.22 (m, 2H), 2.35-2.28 (m, 1H), 1.68 (s, 3H), 1.46-1.29 (m, 3H), 1.27-1.24 (m, 4H); ^{13}C NMR (100 MHz, $CDCl_3$) δ 143.9, 143.8, 114.2, 114.0, 58.0, 57.7, 54.3, 54.2, 46.8, 46.7, 33.7, 33.6, 26.7, 26.6, 19.5, 19.3, 18.7, 18.5 ppm; HRMS (ESI-TOF) *m/z* calcd. for $C_9H_{17}N_6^+$ (M+H^+) 209.1509, found 209.1515.

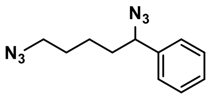

(1,5-Diazidopentyl)benzene (51) After purification by flash column chromatography using petroleum ether/dichloromethane (5:1), the title compound was isolated as a colorless oil (36% yield, 16.6 mg); R_f = 0.7 (PE: DCM = 5:1); ^{1}H NMR (400 MHz, $CDCl_3$) δ 7.40-7.25 (m, 5H), 4.44-4.40 (m, 1H), 3.27-3.23 (m, 2H), 1.90-1.17 (m, 2H), 1.65-1.54 (m, 2H), 1.54-1.40 (m, 1H), 1.40-1.33 (m, 1H); ^{13}C NMR (100 MHz, $CDCl_3$) δ 139.5, 128.8, 128.3, 126.8, 66.2, 51.2, 35.7, 28.5, 23.5 ppm; HRMS (ESI-TOF) *m*/*z* calcd. for $C_{11}H_{14}N_6Na^+$ ($M+Na^+$) 253.1172, found 253.1178.

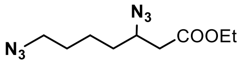

Ethyl 3,7-diazidoheptanoate (52) After purification by flash column chromatography using petroleum ether/ethyl acetate (10:1), the title compound was isolated as a colorless oil (39% yield, 18.7 mg); R_f= 0.6 (PE: EA = 10:1); ^{1}H NMR (400 MHz, $CDCl_3$) δ 4.19 (q, *J* = 7.2 Hz, 2H), 3.84-3.79 (m, 1H), 3.30 (t, *J* = 6.4 Hz, 2H), 2.51 (d, *J* = 7.8 Hz, 2H), 1.65-1.54 (m, 6H), 1.28 (t, *J* = 7.1 Hz, 3H); ^{13}C NMR (100 MHz, $CDCl_3$) δ 170.6, 61.0, 59.0, 51.2, 39.6, 34.0, 28.5, 23.2, 14.1 ppm; HRMS (ESI-TOF) *m*/*z* calcd. for $C_9H_{16}N_6O_2Na^+$ ($M+Na^+$) 263.1227, found 263.1228.

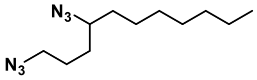

1,4-Diazidoundecane (53) After purification by flash column chromatography using petroleum ether/dichloromethane (5:1), the title compound was isolated as a colorless oil (55% yield, 26.1 mg); R_f = 0.8 (PE: DCM = 5:1); ^{1}H NMR (400 MHz, $CDCl_3$) δ 3.37-3.25 (m, 3H), 1.88-1.72 (m, 1H), 1.69-1.58 (m, 2H), 1.57-1.47 (m, 3H), 1.40-1.22 (m, 10H), 0.88 (t, *J* = 6.8 Hz, 3H); ^{13}C NMR (100 MHz, $CDCl_3$) δ 62.5, 51.1, 34.4, 31.7, 31.5, 29.3, 29.1, 26.0, 25.6, 22.6, 14.0 ppm; HRMS (ESI-TOF) *m*/*z* calcd. for $C_{11}H_{23}N_6^+$ ($M+H^+$) 239.1979, found 239.1987.

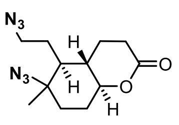

(4aS,5S,8aS)-6-Azido-5-(2-azidoethyl)-6-methyloctahydro-2*H*-chromen-2-one (54) After purification by flash column chromatography using petroleum ether/ethyl acetate (10:1 to 5:1), the title compound was isolated as a colorless oil (19% yield, 10.6 mg); R_f= 0.5 (PE: EA = 5:1); ^{1}H NMR (400 MHz, $CDCl_3$) δ 4.49-4.47 (m, 1H), 3.37 (t, *J* = 7.4 Hz, 2H), 2.63-2.47 (m, 2H), 2.08-1.71 (m, 8H), 1.65-1.58 (m, 1H), 1.53-1.47 (m, 1H), 1.39 (s, 3H); ^{13}C NMR (100 MHz, $CDCl_3$) δ 172.0, 77.5, 64.1, 51.3, 39.7, 33.6, 30.8, 27.4, 26.2, 25.9, 24.8, 22.1 ppm; HRMS (ESI-TOF) *m*/*z* calcd. for $C_{12}H_{19}N_6O_2^+$ ($M+H^+$) 279.1564, found 279.1576.

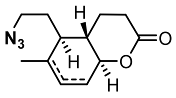

(**54’**, mixture containing products of *β*-hydride elimination) After purification by flash column chromatography using petroleum ether/ethyl acetate (10:1 to 5:1), the title compound was isolated as a colorless oil (44% yield, 20.8 mg); R_f = 0.5 (PE: EA = 5:1); ^{1}H NMR (400 MHz, $CDCl_3$) δ 5.31 (s, 0.3H), 4.91 (s, 0.35H), 4.75 (s, 0.35H), 4.70-4.59 (m, 0.66H), 4.50-4.46 (m, 0.34H), 3.41-3.19 (m, 2H), 2.65-2.46 (m, 2H), 2.35-2.27 (m, 1H), 2.12-1.54 (m, 9H), 1.46-1.38 (m, 1H); ^{13}C NMR (100 MHz, $CDCl_3$) δ 172.0, 170.9, 170.7, 144.4, 134.2, 118.5, 112.3, 77.5, 77.4, 75.0, 64.1, 51.3, 49.2, 44.0, 41.3, 39.7, 38.3, 33.8, 33.6, 31.1, 30.8, 30.4, 30.2, 29.7, 29.0, 28.60, 28.57, 27.4, 26.1, 25.9, 24.8, 22.1, 21.91, 21.88, 21.7 ppm; HRMS (ESI-TOF) *m/z* calcd. for $C_{12}H_{17}N_3O_2Na^+$ (M+Na^+) 258.1213, found 258.1220.

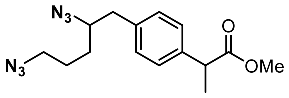

Methyl 2-(4-((r)-2,5-diazidopentyl)phenyl)propanoate (55) After purification by flash column chromatography using petroleum ether/ethyl acetate (10:1 to 5:1), the title compound was isolated as a colorless oil (45% yield, 28.8 mg); R_f= 0.5 (PE: EA = 10:1); ^{1}H NMR (400 MHz, $CDCl_3$) δ 7.26-7.24 (m, 2H), 7.18-7.15 (m, 2H), 3.72 (q, *J* = 7.2 Hz, 1H), 3.65 (s, 3H), 3.55-3.48 (m, 1H), 3.30 (t, *J* = 6.6 Hz, 2H), 2.85-2.77 (m, 2H), 1.85-1.75 (m, 1H), 1.69-1.60 (m, 2H), 1.57-1.48 (m, 4H); ^{13}C NMR (100 MHz, $CDCl_3$) δ 174.9, 139.1, 136.2, 129.4, 127.7, 63.5, 52.0, 51.0, 45.0, 40.4, 31.1, 25.6, 18.5 ppm; HRMS (ESI-TOF) *m/z* calcd. for $C_{15}H_{20}N_6O_2Na^+$ (M+Na^+) 339.1540, found 339.1550.

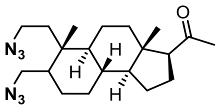

1-((3aS,5aS,6R,9aR,9bS)-6-(2-Azidoethyl)-7-(azidomethyl)-3a,6-dimethyldodecahydro-1*H*-cyclopenta[*a*]naphthalen-3-yl)ethan-1-one (56) After purification by flash column chromatography using petroleum ether/ethyl acetate (20:1 to 15:1), the title compound was isolated as a colorless oil (41% yield, 30.6 mg); R_f= 0.5 (PE: EA = 15:1); ^{1}H NMR (400 MHz, $CDCl_3$) δ 3.54 (dd, *J* = 12.2, 3.9 Hz, 1H), 3.33-3.19 (m, 2H), 2.96 (dd, *J* = 12.2, 8.8 Hz, 1H), 2.52 (t, *J* = 9.1 Hz, 1H), 2.11 (s, 3H), 1.82-1.59 (m, 8H), 1.54-1.38 (m, 4H), 1.29-1.10 (m, 4H), 0.94-0.83 (m, 2H), 0.80 (s, 3H), 0.59 (s, 3H); ^{13}C NMR (100 MHz, $CDCl_3$) δ 209.4, 63.6, 56.6, 53.2, 47.8, 46.0, 43.8, 42.8, 38.8, 37.8, 35.0, 34.7, 31.5, 31.2, 25.9,

24.3, 22.8, 21.0, 16.4, 13.3 ppm; HRMS (ESI-TOF) *m/z* calcd. for $C_{20}H_{32}N_6ONa^+$ (M+Na$^+$) 395.2530, found 395.2541.

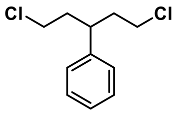

(1,5-Dichloropentan-3-yl)benzene (57) After purification by flash column chromatography using petroleum ether/dichloromethane (5:1), the title compound was isolated as a white solid (63% yield, 27.3 mg); R_f = 0.8 (PE: DCM = 5:1); ^{1}H NMR (400 MHz, $CDCl_3$) δ 7.35-7.30 (m, 2H), 7.25-7.23 (m, 1H), 7.20-7.17 (m, 2H), 3.44-3.37 (m, 2H), 3.27-3.21 (m, 2H), 3.12-3.05 (m, 1H), 2.16-2.02 (m, 4H); ^{13}C NMR (100 MHz, $CDCl_3$) δ 141.7, 128.8, 127.7, 127.0, 42.7, 40.1, 39.1 ppm; HRMS (ESI-TOF) *m/z* calcd. for $C_{11}H_{14}Cl_2Na^+$ (M+Na$^+$) 239.0365, found 239.0346.

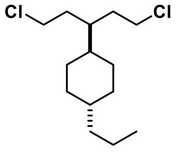

1-((Trans-4-propylcyclohexyl)-1,5-dichloropentane (58) After purification by flash column chromatography using petroleum ether/dichloromethane (5:1), the title compound was isolated as a colorless oil (51% yield, 27.0 mg); R_f = 0.8 (PE: DCM = 5:1); ^{1}H NMR (400 MHz, $CDCl_3$) δ 3.59-3.51 (m, 4H), 1.89-1.76 (m, 4H), 1.68-1.55 (m, 5H), 1.33-1.28 (m, 3H), 1.15-1.05 (m, 5H), 0.90-0.85 (m, 5H); ^{13}C NMR (100 MHz, $CDCl_3$) δ 43.6, 39.9, 39.7, 38.4, 37.6, 34.1, 33.4, 29.2, 20.1, 14.5 ppm; HRMS (ESI-TOF) *m/z* calcd. for $C_{14}H_{30}Cl_2N^+$ (M+NH$_4^+$) 282.1750, found 282.1731.

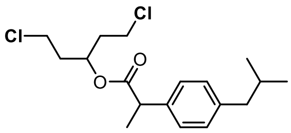

1,5-Dichloropentan-3-yl 2-(4-isobutylphenyl)propanoate (59) After purification by flash column chromatography using petroleum ether/dichloromethane (5:1 to 3:1), the title compound was isolated as a colorless oil (38% yield, 26.1 mg); R_f = 0.5 (PE: DCM = 3:1); ^{1}H NMR (400 MHz, $CDCl_3$) δ 7.18 (d, *J* = 8.0 Hz, 2H), 7.10 (d, *J* = 7.8 Hz, 2H), 5.19-5.13 (m, 1H), 3.68 (q, *J* = 7.0 Hz, 1H), 3.48-3.44 (m, 2H), 3.22-3.17 (m, 1H), 3.09-3.02 (m, 1H), 2.45 (d, *J* = 7.2 Hz, 2H), 2.10-1.81 (m, 5H), 1.49 (d, *J* = 7.0 Hz, 3H), 0.89 (d, *J* = 6.6 Hz, 6H); ^{13}C NMR (100 MHz, $CDCl_3$) δ 174.1, 140.8, 137.5, 129.4, 127.0, 69.0, 45.2, 44.9, 40.4, 39.9, 37.2, 37.0, 30.2, 22.29, 22.26, 17.9 ppm; HRMS (ESI-TOF) *m/z* calcd. for $C_{18}H_{30}Cl_2O_2N^+$ (M+NH$_4^+$) 362.1648, found 362.1630.

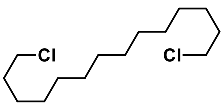

1,14-Dichlorotetradecane (60) After purification by flash column chromatography using petroleum ether/dichloromethane (5:1), the title compound was isolated as a colorless oil (46% yield, 24.5 mg); R_f = 0.8 (PE: DCM = 5:1); ^{1}H NMR (400 MHz, $CDCl_3$) δ 3.55-3.51 (m, 4H), 1.81-1.73 (m, 4H), 1.44-1.38 (m, 4H), 1.32-1.26 (m, 16H); ^{13}C NMR (100 MHz, $CDCl_3$) δ 45.2, 32.6, 29.6, 29.5, 29.4, 28.9, 26.9 ppm; HRMS (ESI-TOF) *m/z* calcd. for $C_{14}H_{32}Cl_2N^+$ ($M+NH_4^+$) 284.1906, found 284.1899.

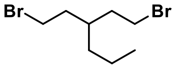

1-Bromo-3-(2-bromoethyl)hexane (61) After purification by flash column chromatography using petroleum ether/dichloromethane (5:1), the title compound was isolated as a colorless oil (37% yield, 19.9 mg); R_f= 0.8 (PE: DCM = 5:1); ^{1}H NMR (400 MHz, $CDCl_3$) δ 3.42 (t, *J* = 7.2 Hz, 4H), 1.87-1.72 (m, 5H), 1.35-1.27 (m, 4H), 0.91 (t, *J* = 6.8 Hz, 3H); ^{13}C NMR (100 MHz, $CDCl_3$) δ 36.6, 35.3, 34.8, 31.3, 19.3, 14.3 ppm; HRMS (ESI-TOF) *m/z* calcd. for $C_8H_{20}Br_2N^+$ ($M+NH_4^+$) 287.9957, found 287.9949.

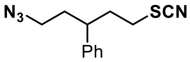

(1-Azido-5-isothiocyanatopentan-3-yl)benzene (62) After purification by flash column chromatography using petroleum ether/ethyl acetate (20:1 to 10:1), the title compound was isolated as a colorless oil (60% yield, 29.5 mg); R_f= 0.6 (PE: EA= 10:1); ^{1}H NMR (400 MHz, $CDCl_3$) δ 7.35-7.32 (m, 2H), 7.27-7.23 (m, 1H), 7.15 (d, *J* = 7.4 Hz, 2H), 3.23-3.17 (m, 1H), 3.07-3.01 (m, 1H), 2.90-2.75 (m, 2H), 2.67-2.59 (m, 1H), 2.21-2.07 (m, 2H), 2.00-1.81 (m, 2H); ^{13}C NMR (100 MHz, $CDCl_3$) δ 141.2, 129.1, 127.5, 127.3, 112.0, 49.1, 41.5, 36.4, 35.6, 31.7 ppm; HRMS (ESI-TOF) *m/z* calcd. for $C_{12}H_{14}N_4SNa^+$ ($M+Na^+$) 269.0831, found 269.0840.

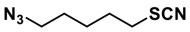

1-Azido-5-thiocyanatopentane (63) After purification by flash column chromatography using petroleum ether/ethyl acetate (20:1 to 10:1), the title compound was isolated as a colorless oil (50% yield, 17.1 mg); R_f= 0.6 (PE: EA= 10:1); ^{1}H NMR (400 MHz, $CDCl_3$) δ 3.31 (t, *J* = 6.5 Hz, 2H), 2.95 (t, *J* = 7.2 Hz, 2H), 1.90-1.82 (m, 2H), 1.66-1.52 (m, 4H); ^{13}C NMR (100 MHz, $CDCl_3$) δ 112.1, 51.0, 33.7, 29.4, 28.2, 25.1 ppm; HRMS (ESI-TOF) *m/z* calcd. for $C_6H_{10}N_4SNa^+$ ($M+Na^+$) 193.0518, found 193.0527.

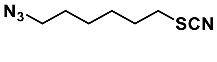

1-Azido-6-thiocyanatohexane (64) After purification by flash column chromatography using petroleum ether/ethyl acetate (20:1 to 10:1), the title compound was isolated as a colorless oil (52% yield, 19.2 mg); R_f= 0.6 (PE: EA= 10:1); ^{1}H NMR (400 MHz, $CDCl_3$) δ 3.28 (t, J = 6.9 Hz, 2H), 2.94 (t, J = 7.2 Hz, 2H), 1.88-1.80 (m, 2H), 1.66-1.58 (m, 2H), 1.50-1.39 (m, 4H); ^{13}C NMR (100 MHz, $CDCl_3$) δ 112.2, 51.2, 33.8, 29.7, 28.6, 27.4, 26.0 ppm; HRMS (ESI-TOF) m/z calcd. for $C_7H_{12}N_4SNa^+$ ($M+Na^+$) 207.0675, found 207.0672.

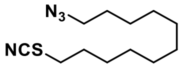

1-Azido-11-thiocyanatoundecane (65) After purification by flash column chromatography using petroleum ether/ethyl acetate (20:1 to 10:1), the title compound was isolated as a colorless oil (61% yield, 30.7 mg); R_f= 0.7 (PE: EA= 10:1); ^{1}H NMR (400 MHz, $CDCl_3$) δ 3.25 (t, J = 6.9 Hz, 2H), 2.94 (t, J = 7.2 Hz, 2H), 1.83-1.80 (m, 2H), 1.63-1.55 (m, 2H), 1.45-1.41 (m, 2H), 1.38-1.25 (m, 12H); ^{13}C NMR (100 MHz, $CDCl_3$) δ 112.4, 51.4, 34.0, 29.8, 29.33, 29.30, 29.2, 29.1, 28.80, 28.78, 27.9, 26.6 ppm; HRMS (ESI-TOF) m/z calcd. for $C_{12}H_{22}N_4SNa^+$ ($M+Na^+$) 277.1457, found 277.1453.

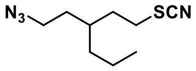

1-Azido-3-(2-thiocyanatoethyl)hexane (66) After purification by flash column chromatography using petroleum ether/ethyl acetate (20:1 to 10:1), the title compound was isolated as a colorless oil (55% yield, 23.3 mg); R_f= 0.7 (PE: EA= 10:1); ^{1}H NMR (400 MHz, $CDCl_3$) δ 3.27 (t, J = 7.1 Hz, 2H), 2.89 (t, J = 7.1 Hz, 2H), 1.77-1.70 (m, 2H), 1.60-1.48 (m, 3H), 1.28-1.19 (m, 4H), 0.86 (t, J = 6.9 Hz, 3H); ^{13}C NMR (100 MHz, $CDCl_3$) δ 111.0, 48.0, 34.1, 32.82, 32.77, 31.3, 30.6, 18.3, 13.2 ppm; HRMS (ESI-TOF) m/z calcd. for $C_9H_{16}N_4SNa^+$ ($M+Na^+$) 235.0988, found 235.0991.

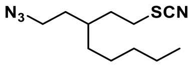

1-Azido-3-(2-thiocyanatoethyl)octane (67) After purification by flash column chromatography using petroleum ether/ethyl acetate (30:1 to 15:1), the title compound was isolated as a colorless oil (53% yield, 25.4 mg); R_f= 0.5 (PE: EA= 15:1); ^{1}H NMR (400 MHz, $CDCl_3$) δ 3.33 (t, J = 7.1 Hz, 2H), 2.95 (t, J = 7.5 Hz, 2H), 1.84-1.78 (m, 2H), 1.66-1.54 (m, 3H), 1.34-1.32 (m, 8H), 0.89 (t, J = 6.5 Hz, 3H); ^{13}C NMR (100 MHz, $CDCl_3$) δ 112.1, 49.1, 34.0, 33.9, 32.8, 32.3, 32.0, 31.6, 25.8, 22.5, 14.0 ppm; HRMS (ESI-TOF) m/z calcd. for $C_{11}H_{20}N_4SNa^+$ ($M+Na^+$) 263.1301, found 263.1298.

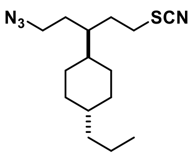

(S)-1-((trans-4-Propylcyclohexyl)-1-azido-5-thiocyanatopentane (68) After purification by flash column chromatography using petroleum ether/ethyl acetate (30:1 to 20:1), the title compound was isolated as a colorless oil (55% yield, 32.4 mg); R_f= 0.7 (PE: EA = 20:1); ^{1}H NMR (400 MHz, $CDCl_3$) δ 3.35-3.28 (m, 2H), 2.99-2.90 (m, 2H), 1.93-1.84 (m, 1H), 1.80-1.77 (m, 2H), 1.73-1.66 (m, 2H), 1.61-1.56 (m, 2H), 1.49-1.41 (m, 2H), 1.35-1.25 (m, 3H), 1.21-1.03 (m, 5H), 0.91-0.82 (m, 5H); ^{13}C NMR (100 MHz, $CDCl_3$) δ 112.1, 49.8, 40.0, 39.5, 39.4, 37.4, 33.2, 32.5, 31.3, 29.8, 29.17, 29.15, 20.0, 14.3 ppm; HRMS (ESI-TOF) *m*/*z* calcd. for $C_{15}H_{26}N_4SNa^+$ (M+Na^+) 317.1770, found 317.1773.

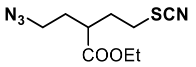

Ethyl 4-azido-2-(2-thiocyanatoethyl)butanoate (69) After purification by flash column chromatography using petroleum ether/ethyl acetate (20:1 to 8:1), the title compound was isolated as a colorless oil (57% yield, 27.5 mg); R_f= 0.5 (PE: EA = 8:1); ^{1}H NMR (400 MHz, $CDCl_3$) δ 4.19 (q, J = 7.1 Hz, 2H), 3.43-3.33 (m, 2H), 3.05-2.87 (m, 2H), 2.71-2.65 (m, 1H), 2.25-2.19 (m, 1H), 2.05-1.92 (m, 2H), 1.80-1.72 (m, 1H), 1.29 (t, J = 7.2 Hz, 3H); ^{13}C NMR (100 MHz, $CDCl_3$) δ 173.7, 111.6, 61.2, 49.1, 41.1, 32.1, 31.5, 31.2, 14.2 ppm; HRMS (ESI-TOF) *m*/*z* calcd. for $C_9H_{14}N_4O_2SNa^+$ (M+Na^+) 265.0730, found 265.0725.

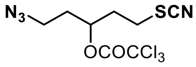

1-Azido-5-thiocyanatopentan-3-yl 2,2,2-trichloroacetate (70) After purification by flash column chromatography using petroleum ether/ethyl acetate (20:1 to 8:1), the title compound was isolated as a colorless oil (60% yield, 39.4 mg); R_f= 0.3 (PE: EA = 8:1); ^{1}H NMR (400 MHz, $CDCl_3$) δ 5.34-5.24 (m, 1H), 3.53-3.41 (m, 2H), 3.11-3.04 (m, 1H), 2.95-2.92 (m, 1H), 2.40-2.14 (m, 2H), 2.03-1.93 (m, 2H); ^{13}C NMR (100 MHz, $CDCl_3$) δ 161.6, 111.2, 89.6, 75.3, 47.1, 34.4, 33.0, 29.4 ppm; HRMS (ESI-TOF) *m*/*z* calcd. for $C_8H_9Cl_3N_4O_2SNa^+$ (M+Na^+) 352.0404, found 352.0399.

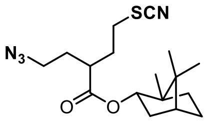

(1r,2s,4r)-1,7,7-Trimethylbicyclo[2.2.1]heptan-2-yl 4-azido-2-(2-thiocyanatoethyl)butanoate (71) After purification by flash column chromatography using petroleum ether/ethyl acetate (20:1 to 7:1), the

title compound was isolated as a colorless oil (64% yield, 44.4 mg, dr could not be determined by ^{1}H NMR due to peak overlapping); R_f= 0.4 (PE: EA= 7:1); ^{1}H NMR (400 MHz, $CDCl_3$) δ 4.98-4.85 (m, 1H), 3.44-3.33 (m, 2H), 3.06-2.94 (m, 1H), 2.95-2.79 (m, 1H), 2.73-2.65 (m, 1H), 2.43-2.33 (m, 1H), 2.27-2.09 (m, 1H), 2.02-1.86 (m, 3H), 1.78-1.71 (m, 3H), 1.39-1.27 (m, 2H), 0.95-0.83 (m, 10H); ^{13}C NMR (100 MHz, $CDCl_3$) δ 173.9, 111.6, 81.03, 80.99, 49.1, 48.84, 48.79, 47.9, 44.9, 44.8, 41.41, 41.36, 37.1, 37.0, 32.3, 32.2, 31.5, 31.4, 31.3, 28.10, 28.07, 27.2, 19.7, 18.8, 13.7, 13.6 ppm; HRMS (ESI-TOF) *m/z* calcd. for $C_{17}H_{26}N_4O_2SNa^+$ (M+Na$^+$) 373.1669, found 373.1677.

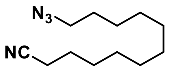

12-Azidododecanenitrile (72) After purification by flash column chromatography using petroleum ether/ethyl acetate (15:1 to 7:1), the title compound was isolated as a colorless oil (22% yield, 9.9 mg); R_f= 0.5 (PE: EA = 7:1); ^{1}H NMR (400 MHz, $CDCl_3$) δ 3.25 (t, *J* = 6.9 Hz, 2H), 2.33 (t, *J* = 7.1 Hz, 2H), 1.69-1.58 (m, 4H), 1.48-1.38 (m, 2H), 1.36-1.25 (m, 12H); ^{13}C NMR (100 MHz, $CDCl_3$) δ 119.8, 51.5, 29.4, 29.3, 29.2, 29.1, 28.8, 28.7, 28.6, 26.7, 25.3, 17.1 ppm; HRMS (ESI-TOF) *m/z* calcd. for $C_{12}H_{22}N_4Na^+$ (M+Na$^+$) 245.1737, found 245.1740.

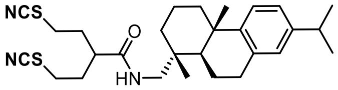

***N*-(((1R,4aS,10aR)-7-Isopropyl-1,4a-dimethyl-1,2,3,4,4a,9,10,10a-octahydrophenanthren-1-yl)methyl)-4-thiocyanato-2-(2-thiocyanatoethyl)butanamide (73)** After purification by flash column chromatography using petroleum ether/ethyl acetate (10:1 to 5:1), the title compound was isolated as a colorless oil (23% yield, 45.6 mg); R_f= 0.4 (PE: EA = 5:1); ^{1}H NMR (400 MHz, $CDCl_3$) δ 7.15 (d, *J* = 8.2 Hz, 1H), 6.99 (d, *J* = 8.0 Hz, 1H), 6.88 (s, 1H), 5.82 (t, *J* = 6.4 Hz, 1H), 3.29-3.24 (m, 1H), 3.18-3.13 (m, 1H), 3.04-2.75 (m, 7H), 2.65-2.59 (m, 1H), 2.32-2.20 (m, 3H), 1.97-1.87 (m, 3H), 1.79-1.68 (m, 3H), 1.37-1.24 (m, 4H), 1.22 (d, *J* = 6.8 Hz, 6H), 1.21 (s, 3H), 0.95 (s, 3H); ^{13}C NMR (100 MHz, $CDCl_3$) δ 172.2, 147.0, 145.8, 134.4, 126.8, 124.0, 123.9, 111.94, 111.89, 50.2, 45.6, 43.2, 38.4, 37.3, 37.2, 36.4, 33.4, 32.6, 31.4, 31.2, 29.7, 25.0, 23.99, 23.95, 19.0, 18.6, 18.4 ppm; HRMS (ESI-TOF) *m/z* calcd. for $C_{28}H_{40}N_3OS_2^+$ (M+H$^+$) 498.2613, found 498.2617.

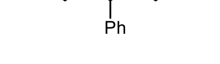

(3-Phenylpentane-1,5-diyl)bis((trifluoromethyl)sulfane) (74) After purification by flash column chromatography using petroleum ether/dichloromethane (20:1 to 5:1), the title compound was isolated as

a colorless oil (57% yield, 39.8 mg); R_f= 0.6 (PE: DCM = 5:1); ^{1}H NMR (400 MHz, $CDCl_3$) δ 7.36-7.32 (m, 2H), 7.27-7.24 (m, 1H), 7.15-7.13 (m, 2H), 2.88-2.81 (m, 1H), 2.75-2.69 (m, 2H), 2.65-2.57 (m, 2H), 2.08-1.96 (m, 4H); ^{13}C NMR (100 MHz, $CDCl_3$) δ 141.3, 131.0 (q, J = 306.8 Hz), 129.1, 127.5, 127.3, 43.4, 36.2, 27.7 ppm; ^{19}F NMR (376 MHz, $CDCl_3$) δ -40.87; HRMS (ESI-TOF) *m*/*z* calcd. for $C_{13}H_{18}F_6S_2N^+$ (M+NH_4^+) 366.0779, found 366.0796.

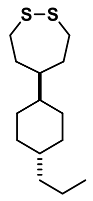

5-((trans-4-Propylcyclohexyl)-1,2-dithiepane (75) After purification by flash column chromatography using petroleum ether/ethyl acetate (40:1), the title compound was isolated as a colorless oil (55% yield, 28.6 mg); R_f= 0.9 (PE: EA = 40:1); ^{1}H NMR (400 MHz, $CDCl_3$) δ 3.00 (dt, J = 12.8, 4.0 Hz, 2H), 2.56 (td, J = 12.8, 4.2 Hz, 2H), 2.06-1.97 (m, 2H), 1.87-1.73 (m, 4H), 1.67-1.57 (m, 3H), 1.34-1.24 (m, 3H), 1.16-1.03 (m, 5H), 0.90-0.82 (m, 5H). ^{13}C NMR (100 MHz, $CDCl_3$) δ 45.4, 42.5, 39.7, 38.0, 37.5, 33.7, 33.3, 29.1, 20.0, 14.4 ppm; HRMS (ESI-TOF) *m*/*z* calcd. for $C_{14}H_{27}S_2^+$ (M+H^+) 259.1549, found 259.1562.

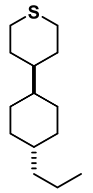

4-((Trans-4-propylcyclohexyl)tetrahydro-2*H*-thiopyran (**76**) After purification by flash column chromatography using petroleum ether/ethyl acetate (40:1), the title compound was isolated as a colorless oil (60% yield, 27.2 mg); R_f= 0.9 (PE: EA = 40:1); ^{1}H NMR (400 MHz, $CDCl_3$) δ 3.01 (dt, J = 12.8, 4.0 Hz, 2H), 2.56 (td, J = 12.6, 4.2 Hz, 2H), 2.02-1.98 (m, 2H), 1.87-1.75 (m, 4H), 1.66-1.60 (m, 3H), 1.33-1.25 (m, 3H), 1.16-1.07 (m, 5H), 0.90-0.82 (m, 5H). ^{13}C NMR (100 MHz, $CDCl_3$) δ 45.5, 42.5, 39.7, 38.1, 37.6, 33.7, 33.3, 29.1, 20.0, 14.4 ppm; HRMS (ESI-TOF) *m*/*z* calcd. for $C_{14}H_{27}S^+$ (M+H^+) 227.1828, found 227.1827.

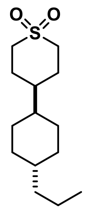

4-((Trans-4-propylcyclohexyl)tetrahydro-2*H*-thiopyran 1,1-dioxide (77) After purification by flash column chromatography using petroleum ether/ethyl acetate (5:1), the title compound was isolated as a white solid (90% yield, 28.1 mg, m.p. 66-68 °C); R_f= 0.5 (PE: EA = 5:1); ^{1}H NMR (400 MHz, $CDCl_3$) δ 3.57-3.51 (m, 1H), 3.41-3.20 (m, 3H), 2.07-2.00 (m, 1H), 1.94-1.89 (m, 2H), 1.86-1.75 (m, 4H), 1.65-1.57 (m, 2H), 1.33-1.24 (m, 3H), 1.17-0.99 (m, 5H), 0.91-0.81 (m, 5H). ^{13}C NMR (100 MHz, $CDCl_3$) δ 65.1, 44.8, 41.9, 39.5, 37.4, 34.4, 33.4, 33.11, 33.09, 29.3, 28.9, 26.3, 19.9, 14.3 ppm; HRMS (ESI-TOF) *m/z* calcd. for $C_{14}H_{27}O_2S^+$ (M+H$^+$) 259.1726, found 259.1727.

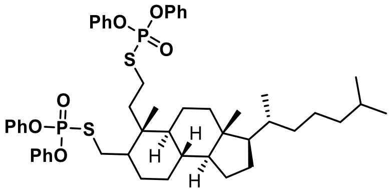

S-(2-((3R,3aR,5aS,6R,9aS,9bS)-7-(((diphenoxyphosphoryl)thio)methyl)-3a,6-dimethyl-3-((R)-6-methylheptan-2-yl)dodecahydro-1*H*-cyclopenta[*a*]naphthalen-6-yl)ethyl) O,O-diphenyl phosphorothioate (78) After purification by flash column chromatography using petroleum ether/ethyl acetate (20:1 to 10:1), the title compound was isolated as a colorless oil (58% yield, 158.2 mg); R_f= 0.5 (PE: EA = 10:1); ^{1}H NMR (400 MHz, $CDCl_3$) δ 7.38-7.27 (m, 16H), 7.24-7.18 (m, 4H), 3.14 (td, *J* = 12.8, 2.6 Hz, 1H), 2.88-2.69 (m, 2H), 2.46-2.35 (m, 1H), 1.96-1.77 (m, 3H), 1.68-1.44 (m, 5H), 1.39-0.97 (m, 16H), 0.95-0.86 (m, 10H), 0.75-0.61 (m, 8H). ^{13}C NMR (100 MHz, $CDCl_3$) δ 150.2 (overlap), 150.1 (overlap), 150.0, 149.9 (overlap), 129.7 (overlap), 129.6 (overlap), 125.6 (overlap), 125.5 (overlap), 125.4 (overlap), 120.9, 120.8 (overlap), 120.7, 120.6 (overlap), 56.3, 56.0, 47.3, 43.3 (d, *J* = 4.4 Hz), 42.1, 40.2, 39.6, 39.4, 37.6 (d, *J* = 4.9 Hz), 36.0, 35.6, 34.9, 33.7 (d, *J* = 4.0 Hz), 31.0, 28.1, 27.9, 25.9, 25.5 (d, *J* = 4.3 Hz), 24.0, 23.7, 22.7, 22.5, 20.8, 18.6, 15.7, 11.8 ppm; ^{31}P NMR (161 MHz, $CDCl_3$) δ 22.07, 21.87; HRMS (ESI-TOF) *m/z* calcd. for $C_{50}H_{66}O_6P_2S_2Na^+$ (M+Na$^+$) 911.3668, found 911.3676.

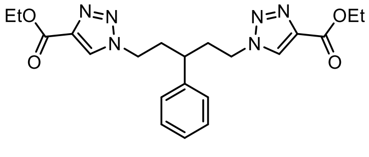

Diethyl 1,1'-(3-phenylpentane-1,5-diyl)bis(1*H*-1,2,3-triazole-4-carboxylate) (79) After purification by flash column chromatography using petroleum ether/ethyl acetate (4:1 to 1:1), the title compound was isolated as a yellow oil (51% yield, 43.5 mg); R_f= 0.3 (PE: EA = 1:1); ^{1}H NMR (400 MHz, $CDCl_3$) δ 7.89-7.88 (m, 2H), 7.34-7.30 (m, 2H), 7.26-7.22 (m, 1H), 7.13-7.10 (m, 2H), 4.37-4.32 (m, 4H), 4.18-

4.12 (m, 4H), 2.58-2.52 (m, 1H), 2.41-2.31 (m, 2H), 2.27-2.17 (m, 2H), 1.37-1.33 (m, 6H); ^{13}C NMR (100 MHz, $CDCl_3$) δ 160.5, 140.4, 140.0, 129.3, 127.6, 127.43, 127.38, 61.1, 48.5, 40.4, 36.6, 14.2 ppm; HRMS (ESI-TOF) *m/z* calcd. for $C_{21}H_{26}N_6O_4Na^+$ (M+Na^+) 449.1908, found 449.1915.

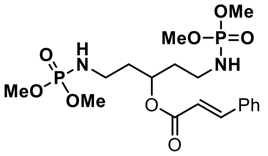

1,5-Bis((dimethoxyphosphoryl)amino)pentan-3-yl cinnamate (80) After purification by flash column chromatography using petroleum ether/ethyl acetate (4:1 to 1:1), the title compound was isolated as a yellow oil (70% yield, 64.9 mg);R_f= 0.2 (PE: EA = 1:1); ^{1}H NMR (400 MHz, $CDCl_3$) δ 7.65 (d, *J* = 16.0 Hz, 1H), 7.51-7.49 (m, 2H), 7.37-7.35 (m, 3H), 6.39 (d, *J* = 16.0 Hz, 1H), 5.23-5.20 (m, 1H), 3.68 (s, 6H), 3.65 (s, 6H), 3.41 (bs, 2H), 3.04-2.84 (m, 4H), 1.83-1.77 (m, 4H); ^{13}C NMR (100 MHz, $CDCl_3$) δ 167.1, 145.5, 134.0, 130.5, 128.9, 128.1, 117.4, 68.9, 53.0 (d, *J* = 5.7 Hz), 37.4, 36.4 (d, *J* = 5.1 Hz) ppm; ^{31}P NMR (161 MHz, $CDCl_3$) δ 12.42; HRMS (ESI-TOF) *m/z* calcd. for $C_{18}H_{30}N_2O_8P_2Na^+$ (M+Na^+) 487.1370, found 487.1380.

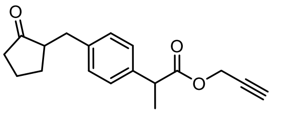

Prop-2-yn-1-yl 2-(4-((2-oxocyclopentyl)methyl)phenyl)propanoate After purification by flash column chromatography using petroleum ether/ethyl acetate (10:1), the title compound was isolated as a colorless oil (92%, 2.61 g); R_f= 0.6 (PE: EA = 10:1); ^{1}H NMR (400 MHz, $CDCl_3$) δ 7.21-7.19 (m, 2H), 7.12-7.10 (m, 2H), 4.72-4.57 (m, 2H), 3.73 (q, *J* = 7.2 Hz, 1H), 3.11 (dd, *J* = 14.2, 4.0 Hz, 1H), 2.53-2.47 (m, 1H), 2.44 (t, *J* = 2.4 Hz, 1H), 2.36-2.28 (m, 2H), 2.14-2.03 (m, 2H), 1.99-1.90 (m, 1H), 1.76-1.67 (m, 1H), 1.59-1.48 (m, 4H); ^{13}C NMR (100 MHz, $CDCl_3$) δ 220.0, 173.6, 138.9, 137.7, 129.1, 127.4, 77.5, 74.8, 52.1, 50.8, 44.7, 38.0, 35.1, 29.1, 20.4, 18.4 ppm.

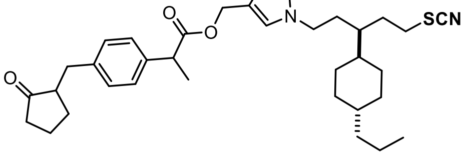

(1-((S)-3-((1s,4S)-4-Propylcyclohexyl)-5-thiocyanatopentyl)-1*H*-1,2,3-triazol-4-yl)methyl 2-(4-((2-oxocyclopentyl)methyl)phenyl)propanoate (81) After purification by flash column chromatography using petroleum ether/ethyl acetate (4:1 to 2:1), the title compound was isolated as a colorless oil (73%

yield, 84.5 mg); R_f= 0.3 (PE: EA = 2:1); ^{1}H NMR (400 MHz, $CDCl_3$) δ 7.48 (s, 1H), 7.18-7.16 (m, 2H), 7.10-7.08 (m, 2H), 5.24-5.12 (m, 2H), 4.32 (t, J = 7.2 Hz, 2H), 3.69 (q, J = 7.2 Hz, 1H), 3.08 (dd, J = 14.0, 4.2 Hz, 1H), 2.94-2.81 (m, 2H), 2.52-2.46 (m, 1H), 2.35-2.28 (m, 2H), 2.13-2.04 (m, 2H), 1.97-1.85 (m, 3H), 1.80-1.66 (m, 5H), 1.58-1.45 (m, 6H), 1.33-1.22 (m, 4H), 1.15-1.01 (m, 5H), 0.89-0.81 (m, 5H); ^{13}C NMR (100 MHz, $CDCl_3$) δ 220.0, 174.4, 142.9, 138.9, 137.9, 129.0, 127.5, 123.5, 112.0, 57.9, 50.8, 48.7, 44.8, 39.7, 39.4, 39.01, 38.99, 38.1, 37.3, 35.1, 33.1, 32.1, 31.2, 31.1, 29.2, 29.1, 29.0, 20.4, 19.9, 18.4, 14.3 ppm; HRMS (ESI-TOF) m/z calcd. for $C_{33}H_{46}N_4O_3SNa^+$ (M+Na^+) 601.3183, found 601.3190.

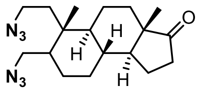

(3aS,5aS,6R,9aR,9bS)-6-(2-Azidoethyl)-7-(azidomethyl)-3a,6-dimethyldodecahydro-3*H*-cyclopenta[*a*]naphthalen-3-one (82) After purification by flash column chromatography using petroleum ether/ethyl acetate (20:1 to 10:1), the title compound was isolated as a colorless oil (59% yield, 40.5 mg); R_f= 0.5 (PE: EA = 10:1); ^{1}H NMR (400 MHz, $CDCl_3$) δ 3.57 (dd, J = 12.2, 4.0 Hz, 1H), 3.33-3.19 (m, 2H), 2.99 (dd, J = 12.2, 9.0 Hz, 1H), 2.48-2.41 (m, 1H), 2.12-2.03 (m, 1H), 1.98-1.92 (m, 1H), 1.89-1.81 (m, 3H), 1.78-1.64 (m, 2H), 1.59-1.49 (m, 4H), 1.43-1.23 (m, 4H), 0.97-0.90 (m, 2H), 0.85 (s, 3H), 0.83 (s, 3H); ^{13}C NMR (100 MHz, $CDCl_3$) δ 220.5, 53.1, 51.2, 48.0, 47.3, 45.8, 42.7, 37.9, 35.7, 34.61, 34.55, 31.2, 30.0, 25.6, 21.6, 20.2, 16.3, 13.5 ppm; HRMS (ESI-TOF) m/z calcd. for $C_{18}H_{29}N_6O^+$ (M+H^+) 345.2397, found 345.2409.

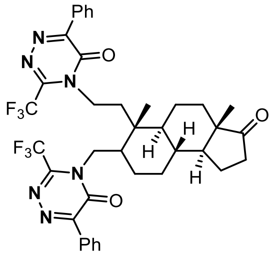

4-(((3aS,5aS,6R,9aR,9bS)-3a,6-Dimethyl-3-oxo-6-(2-(5-oxo-6-phenyl-3-(trifluoromethyl)-1,2,4-triazin-4(5*H*)-yl)ethyl)dodecahydro-1*H*-cyclopenta[*a*]naphthalen-7-yl)methyl)-6-phenyl-3-(trifluoromethyl)-1,2,4-triazin-5(4*H*)-one (83) After purification by flash column chromatography using petroleum ether/ethyl acetate (4:1), the title compound was isolated as a colorless oil (35%, 30.6 mg); R_f= 0.3 (PE: EA = 4:1); ^{1}H NMR (400 MHz, $CDCl_3$) δ 8.31-8.26 (m, 4H), 7.56-7.52 (m, 2H), 7.50-7.45 (m, 4H), 4.95-4.90 (m, 1H), 4.86-4.79 (m, 1H), 4.12-4.04 (m, 1H), 4.00-3.94 (m, 1H), 2.49-2.31 (m, 2H), 2.12-1.99 (m, 2H), 1.93-1.79 (m, 5H), 1.60-1.29 (m, 7H), 1.17-1.09 (m, 2H), 1.00 (s, 3H), 0.88 (s,

3H); ^{13}C NMR (100 MHz, CDCl$_3$) δ 220.2, 158.3, 158.2, 152.0, 151.9, 143.1 (q, J = 34.0 Hz), 132.01, 131.97, 131.4, 129.6, 128.43, 128.41, 117.9 (q, J = 279.8 Hz), 51.4, 48.6, 47.6, 46.9, 41.8, 39.1, 38.4, 35.7, 35.1, 33.2, 31.4, 29.7, 22.6, 21.6, 20.4, 15.9, 13.8 ppm; ^{19}F NMR (376 MHz, CDCl$_3$) δ -65.11. HRMS (ESI-TOF) m/z calcd. for $C_{38}H_{39}F_6N_6O_3^+$ (M+H$^+$) 741.2982, found 741.2983.

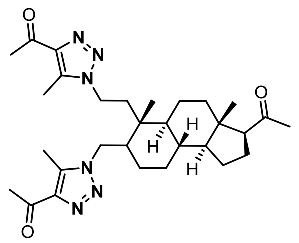

1-((3S,3aS,5aS,6R,9aR,9bS)-6-(2-(4-Acetyl-5-methyl-1*H*-1,2,3-triazol-1-yl)ethyl)-7-((4-acetyl-5-methyl-1*H*-1,2,3-triazol-1-yl)methyl)-3a,6-dimethyldodecahydro-1*H*-cyclopenta[*a*]naphthalen-3-yl)ethan-1-one (84) After purification by flash column chromatography using petroleum ether/ethyl acetate (5:1 to 2:1), the title compound was isolated as a colorless oil (41%, 43.9 mg); R_f= 0.3 (PE: EA = 2:1); ^{1}H NMR (400 MHz, CDCl$_3$) δ 4.27-4.19 (m, 2H), 3.58 (dd, J = 12.4, 4.4 Hz, 1H), 3.08 (dd, J = 12.4, 8.0 Hz, 1H), 2.68 (s, 3H), 2.59 (s, 3H), 2.19-2.08 (m, 6H), 2.01-1.89 (m, 3H), 1.81-1.65 (m, 5H), 1.62-1.57 (m, 3H), 1.39-1.11 (m, 9H), 0.95-0.83 (m, 5H), 0.62 (s, 3H); ^{13}C NMR (100 MHz, CDCl$_3$) δ 209.3, 194.5, 136.7, 136.2, 63.6, 56.7, 53.4, 47.8, 43.9, 42.9, 42.3, 38.8, 38.2, 36.3, 35.0, 31.5, 31.3, 29.7, 27.7, 26.2, 24.4, 22.8, 21.1, 16.3, 13.3, 9.0 ppm; HRMS (ESI-TOF) m/z calcd. for $C_{30}H_{44}N_6O_3Na^+$ (M+Na$^+$) 559.3367, found 559.3378.

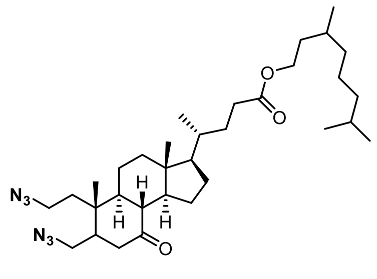

3,7-Dimethyloctyl (4R)-4-((3R,3aR,5aS,6R,9aS,9bS)-6-(2-azidoethyl)-7-(azidomethyl)-3a,6-dimethyl-9-oxododecahydro-1*H*-cyclopenta[*a*]naphthalen-3-yl)pentanoate (85) After purification by flash column chromatography using petroleum ether/ethyl acetate (20:1 to 10:1), the title compound was isolated as a colorless oil (39% yield, 45.5 mg); R_f= 0.6 (PE: EA = 10:1); ^{1}H NMR (400 MHz, CDCl$_3$) δ 4.11-4.02 (m, 2H), 3.45-3.32 (m, 2H), 3.05 (t, J = 12.4 Hz, 1H), 2.70-2.65 (m, 1H), 2.43-2.28 (m, 3H), 2.22-2.15 (m, 2H), 2.03-1.88 (m, 3H), 1.82-1.74 (m, 2H), 1.68-1.46 (m, 6H), 1.41-1.21 (m, 13H), 1.19-1.02 (m, 5H), 0.94-0.84 (m, 13H), 0.64 (s, 3H); ^{13}C NMR (100 MHz, CDCl$_3$) δ 211.0, 174.2, 62.8, 54.7,

50.5, 49.8, 49.5, 48.7, 46.8, 46.2, 42.4, 41.2, 39.1, 38.7, 37.5, 37.1, 36.7, 35.5, 35.1, 31.3, 30.9, 29.8, 28.2, 27.9, 24.7, 24.6, 22.6, 22.5, 22.1, 20.1, 19.5, 18.3, 12.0 ppm; HRMS (ESI-TOF) *m/z* calcd. for $C_{33}H_{56}N_6O_3Na^+$ (M+Na$^+$) 607.4306, found 607.4312.

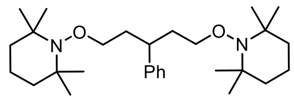

1,1'-((3-Phenylpentane-1,5-diyl)bis(oxy))bis(2,2,6,6-tetramethylpiperidine) (86) After purification by flash column chromatography using petroleum ether/ethyl acetate (25:1), the title compound was isolated as a colorless oil (35% yield, 63.3 mg); R_f = 0.5 (PE: EA = 25:1); ^{1}H NMR (400 MHz, $CDCl_3$) δ 7.26-7.12 (m, 5H), 3.65-3.55 (m, 4H), 2.85-2.78 (m, 1H), 1.95-1.90 (m, 2H), 1.79-1.72 (m, 2H), 1.58-1.25 (m, 12H), 1.07-1.02 (m, 24H); ^{13}C NMR (100 MHz, $CDCl_3$) δ 145.2, 128.2, 127.6, 125.9, 74.8, 59.5, 39.62, 39.56, 39.5, 35.8, 33.0, 32.8, 20.1, 20.0, 17.1 ppm; HRMS (ESI-TOF) *m/z* calcd. for $C_{29}H_{51}N_2O_2^+$ (M+H$^+$) 459.3945, found 459.3956.

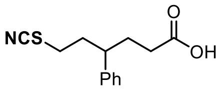

6-(Cyanosulfanyl)-4-phenylhexanoic acid (1') After purification by flash column chromatography using petroleum ether/ethyl acetate (1:1), the title compound was isolated as a light yellow oil (10% yield, 5.0 mg); R_f = 0.2 (PE: EA = 1:1); ^{1}H NMR (400 MHz, $CDCl_3$) δ 7.34-7.30 (m, 2H), 7.25-7.22 (m, 1H), 7.14-7.12 (m, 2H), 2.82-2.72 (m, 2H), 2.68-2.60 (m, 1H), 2.21-2.17 (m, 2H), 2.12-1.85 (m, 4H); ^{13}C NMR (100 MHz, $CDCl_3$) δ 178.6, 141.5, 129.1, 127.6, 127.3, 112.1, 43.6, 36.5, 31.8, 31.7, 31.2 ppm; HRMS (ESI-TOF) *m/z* calcd. for $C_{13}H_{14}NO_2S^-$(M-H$^+$) 248.0751, found 248.0763.

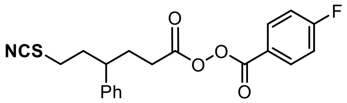

4-Fluorobenzoic 4-phenyl-6-thiocyanatohexanoic peroxyanhydride (87) After purification by flash column chromatography using petroleum ether/ethyl acetate (10:1), the title compound was isolated as a colorless oil (51% yield, 39.5 mg); R_f = 0.4 (PE: EA = 10:1); ^{1}H NMR (400 MHz, $CDCl_3$) δ 8.04-8.00 (m, 2H), 7.37-7.33 (m, 2H), 7.28-7.25 (m, 1H), 7.20-7.14 (m, 4H), 2.89-2.76 (m, 2H), 2.71-2.64 (m, 1H), 2.36-2.31 (m, 2H), 2.24-2.08 (m, 3H), 2.05-1.97 (m, 1H); ^{13}C NMR (100 MHz, $CDCl_3$) δ 168.8, 166.4 (d, *J* = 256.8 Hz), 162.0, 141.0, 132.4 (d, *J* = 9.6 Hz), 129.2, 127.6, 127.5, 121.6 (d, *J* = 3.2 Hz), 116.2 (d, *J*

= 22.2 Hz), 112.0, 43.4, 36.5, 31.8, 31.5, 27.9 ppm; ^{19}F NMR (376 MHz, $CDCl_3$) δ -102.25; HRMS (ESI-TOF) *m/z* calcd. for $C_{20}H_{18}FNO_4SNa^+$ (M+Na^+) 410.0833, found 410.0840.

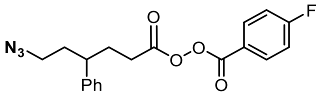

6-Azido-4-phenylhexanoic 4-fluorobenzoic peroxyanhydride (C1) After purification by flash column chromatography using petroleum ether/dichloromethane (4:1 to 2:1), the title compound was isolated as a colorless oil, R_f = 0.8 (PE: DCM = 2:1); ^{1}H NMR (400 MHz, $CDCl_3$) δ 7.98-7.94 (m, 2H), 7.30-7.26 (m, 2H), 7.21-7.17 (m, 1H), 7.13-7.06 (m, 4H), 3.15-3.09 (m, 1H), 3.03-2.96 (m, 1H), 2.77-2.69 (m, 1H), 2.26 (t, *J* = 7.7 Hz, 2H), 2.14-2.06 (m, 1H), 1.97-1.91 (m, 2H), 1.87-1.77 (m, 1H); ^{13}C NMR (100 MHz, $CDCl_3$) δ 168.9, 166.4 (d, *J* = 256.7 Hz), 162.0, 141.9, 132.4 (d, *J* = 9.6 Hz), 128.9, 127.6, 127.1, 121.7 (d, *J* = 3.1 Hz), 116.2 (d, *J* = 22.1 Hz), 49.3, 42.2, 35.6, 31.5, 28.1 ppm; ^{19}F NMR (376 MHz, $CDCl_3$) δ -102.47.

10. NMR spectra of starting materials

^{1}H NMR (400 MHz, $CDCl_3$) of **S1'**

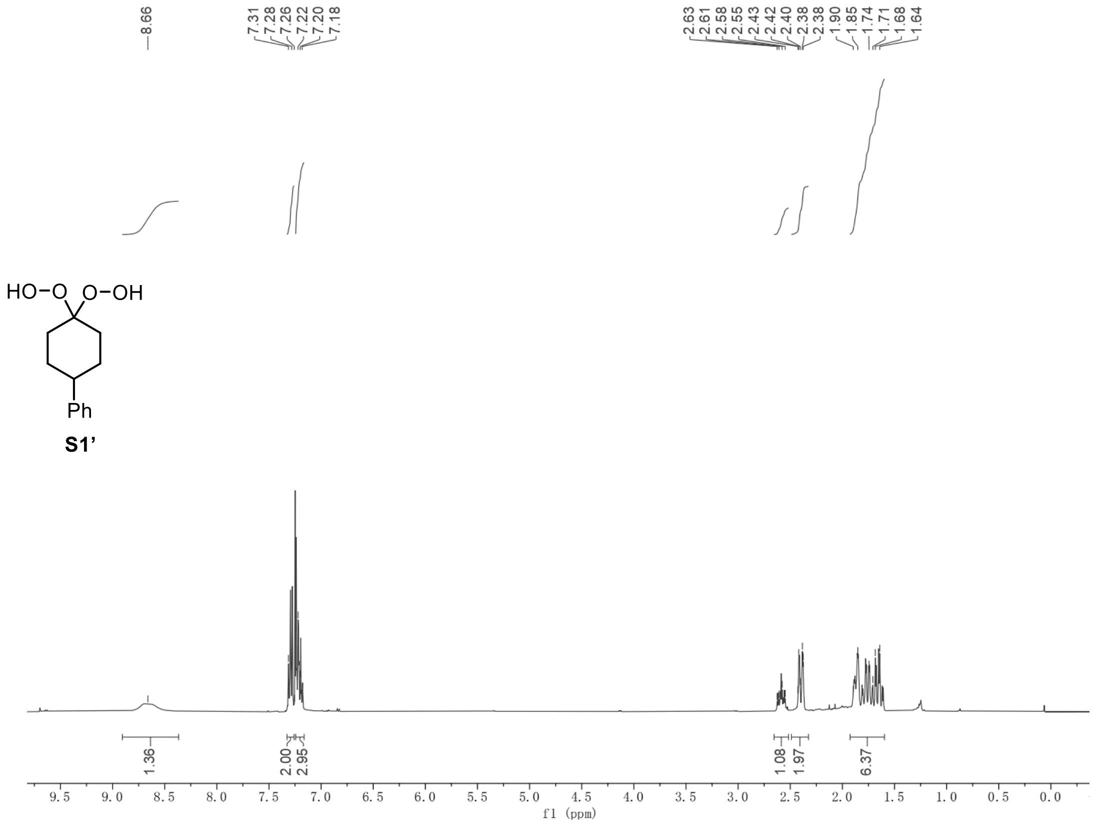

^{13}C NMR (100 MHz, $CDCl_3$) of **S1'**

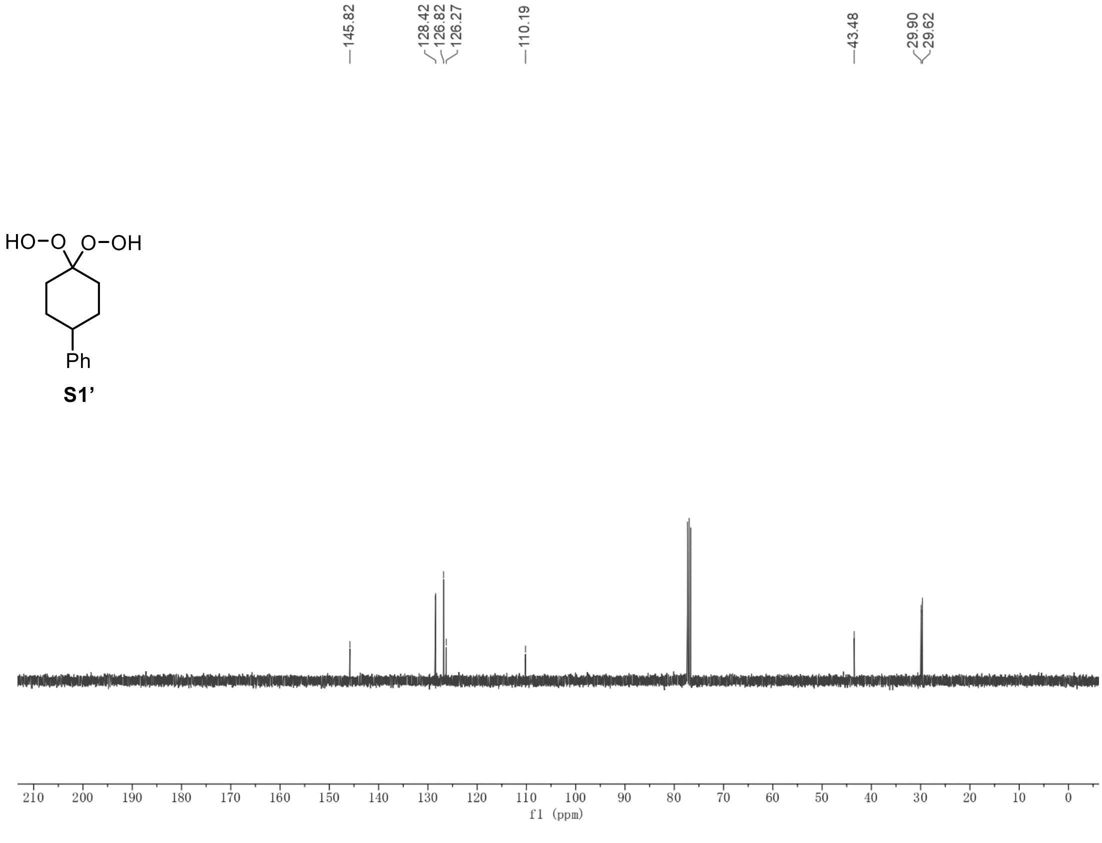

^{1}**H NMR** (400 MHz, $CDCl_3$) of **S1 (I)**

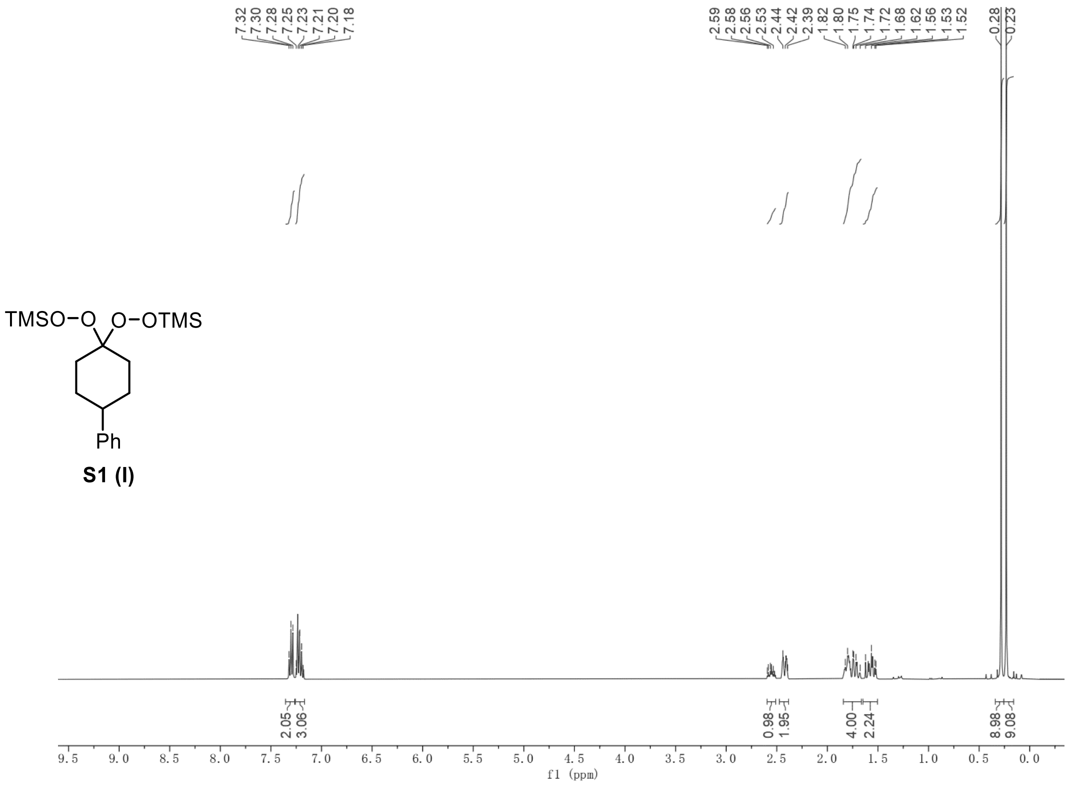

^{13}**C NMR** (100 MHz, $CDCl_3$) of **S1 (I)**

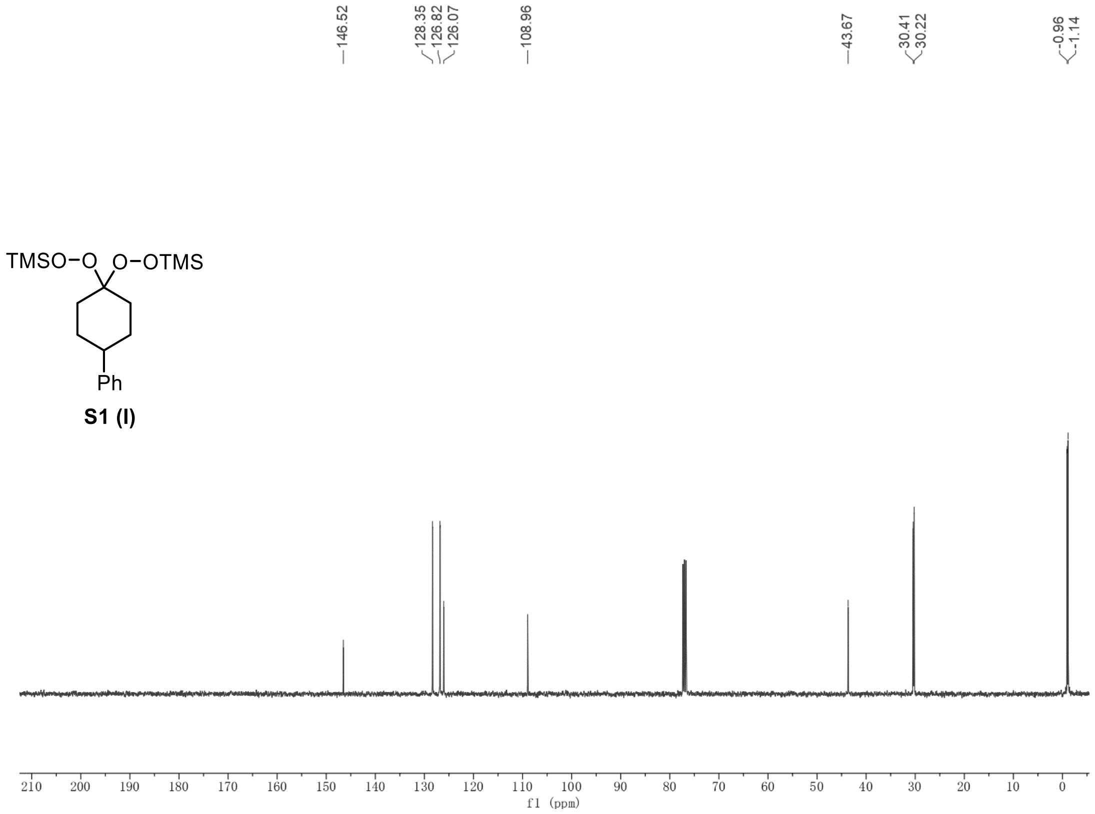

^{1}H NMR (400 MHz, CDCl$_3$) of **S1(II)**

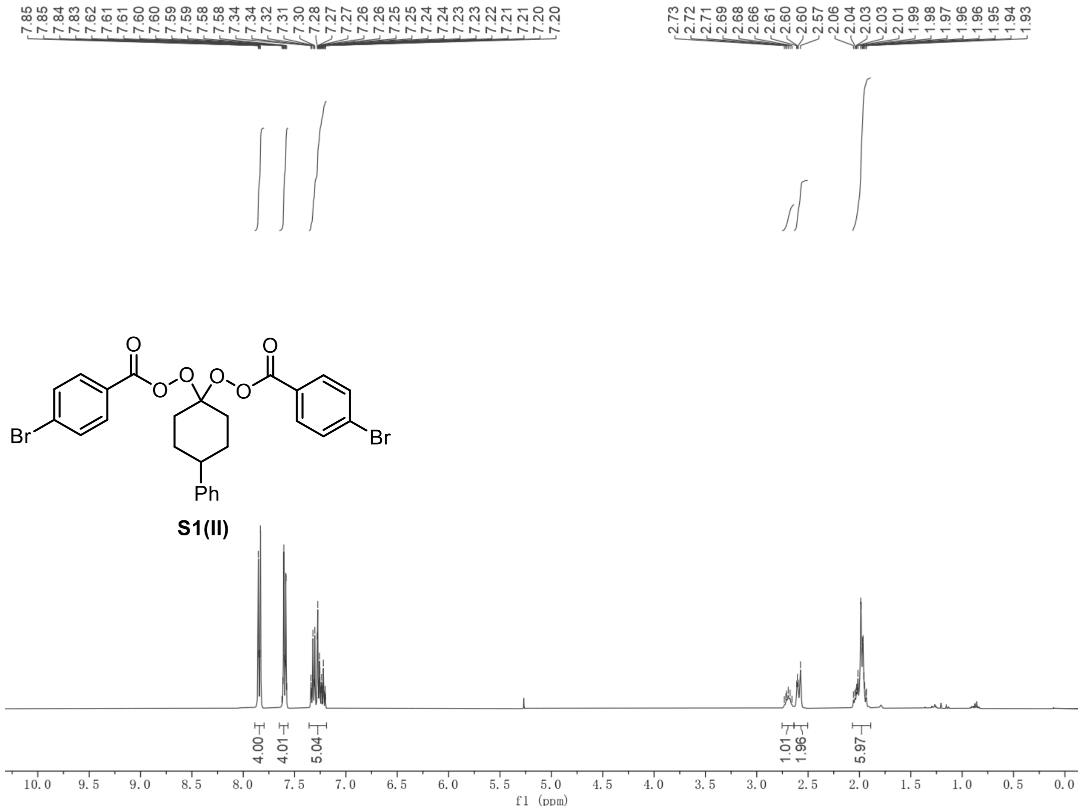

^{13}C NMR (100 MHz, CDCl$_3$) of **S1(II)**

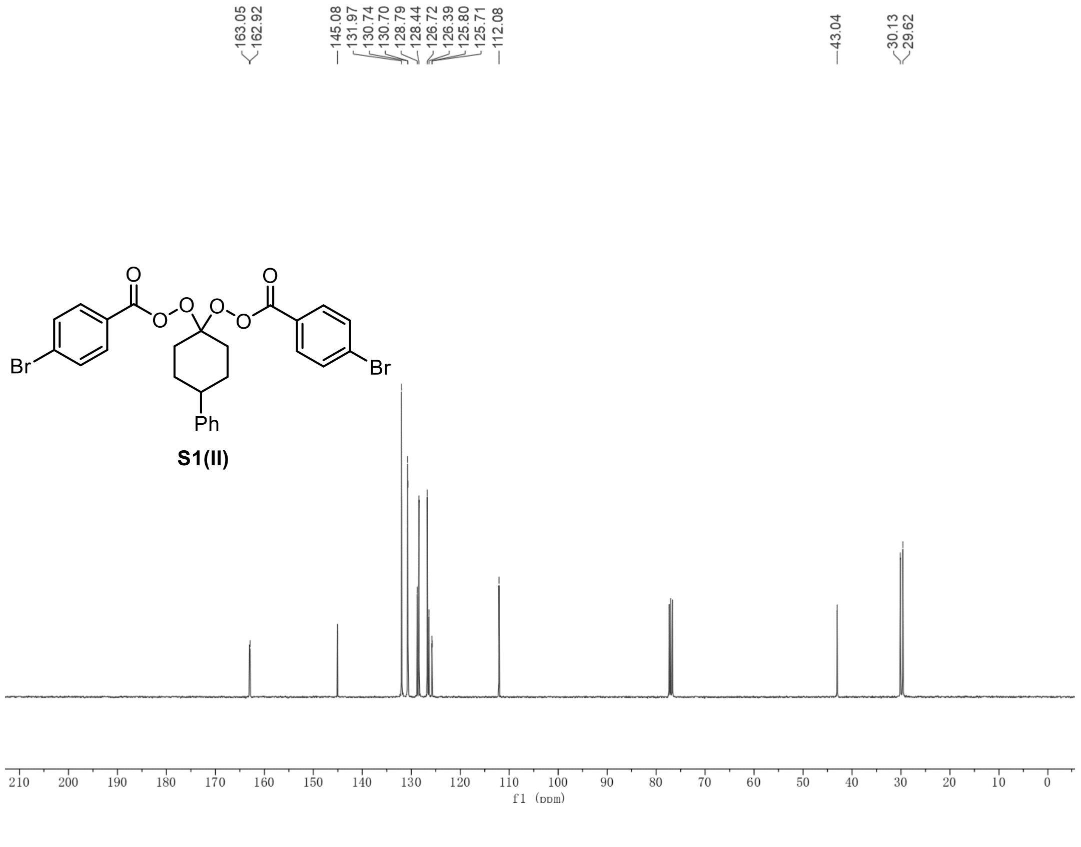

^{1}H NMR (400 MHz, $CDCl_3$) of **S1(III)**

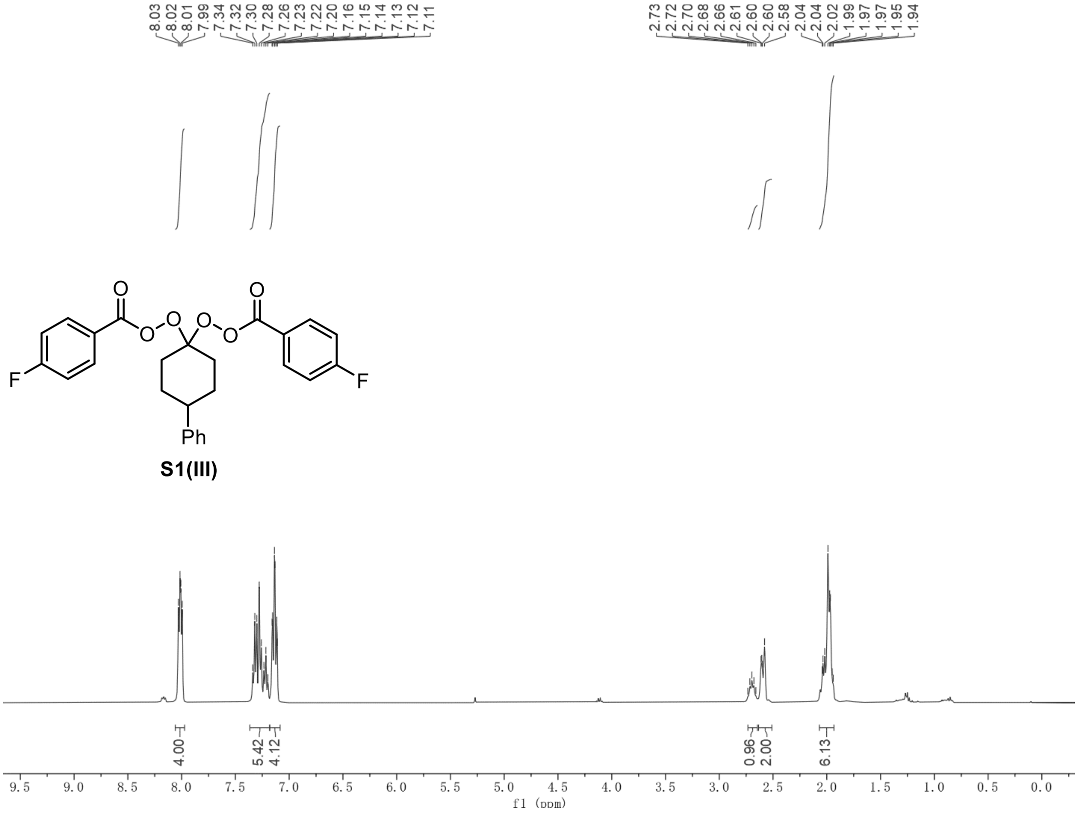

^{19}F NMR (376 MHz, $CDCl_3$) of **S1(III)**

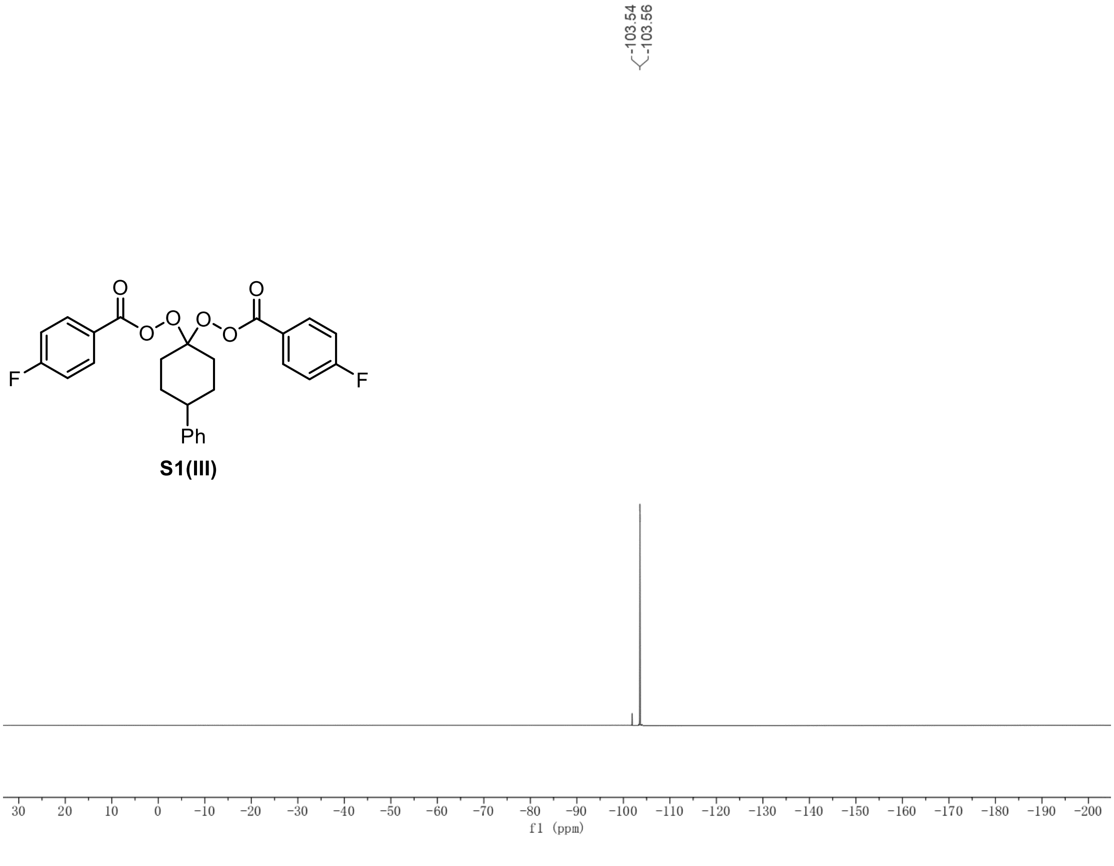

^{13}C NMR (100 MHz, $CDCl_3$) of **S1(III)**

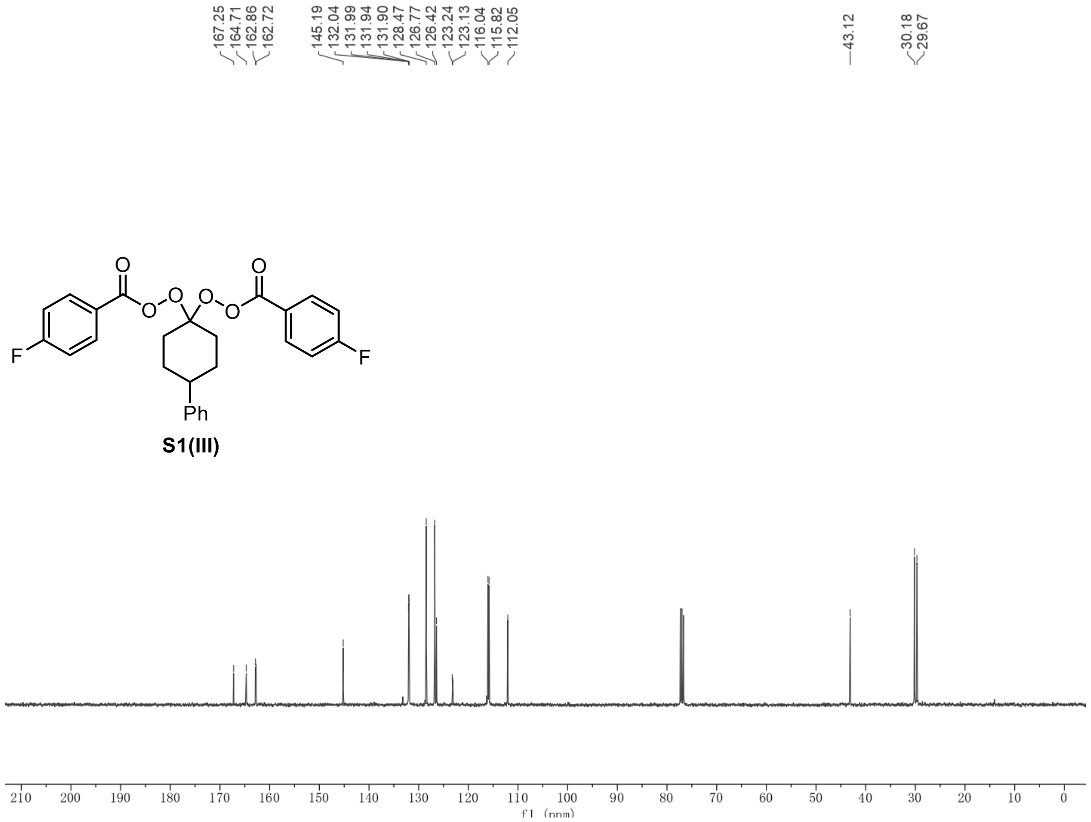

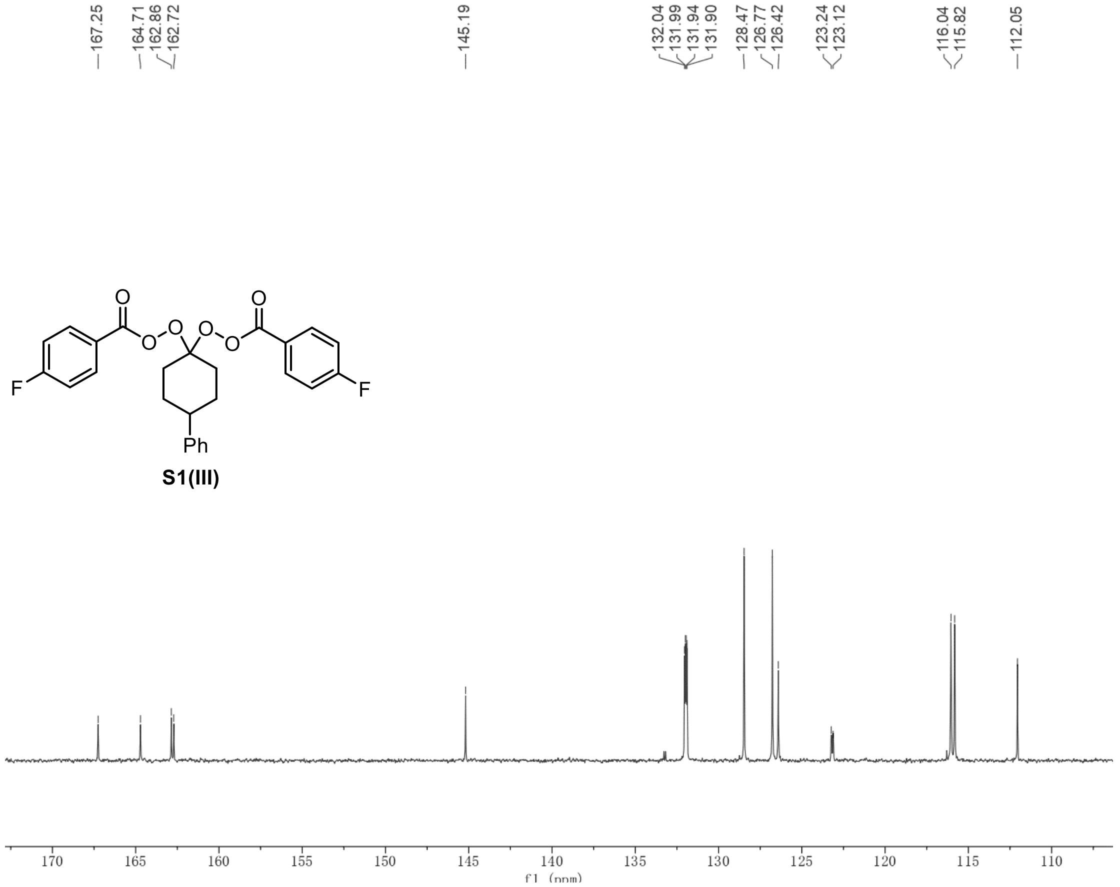

^{1}**H NMR** (400 MHz, $CDCl_3$) of **S1**

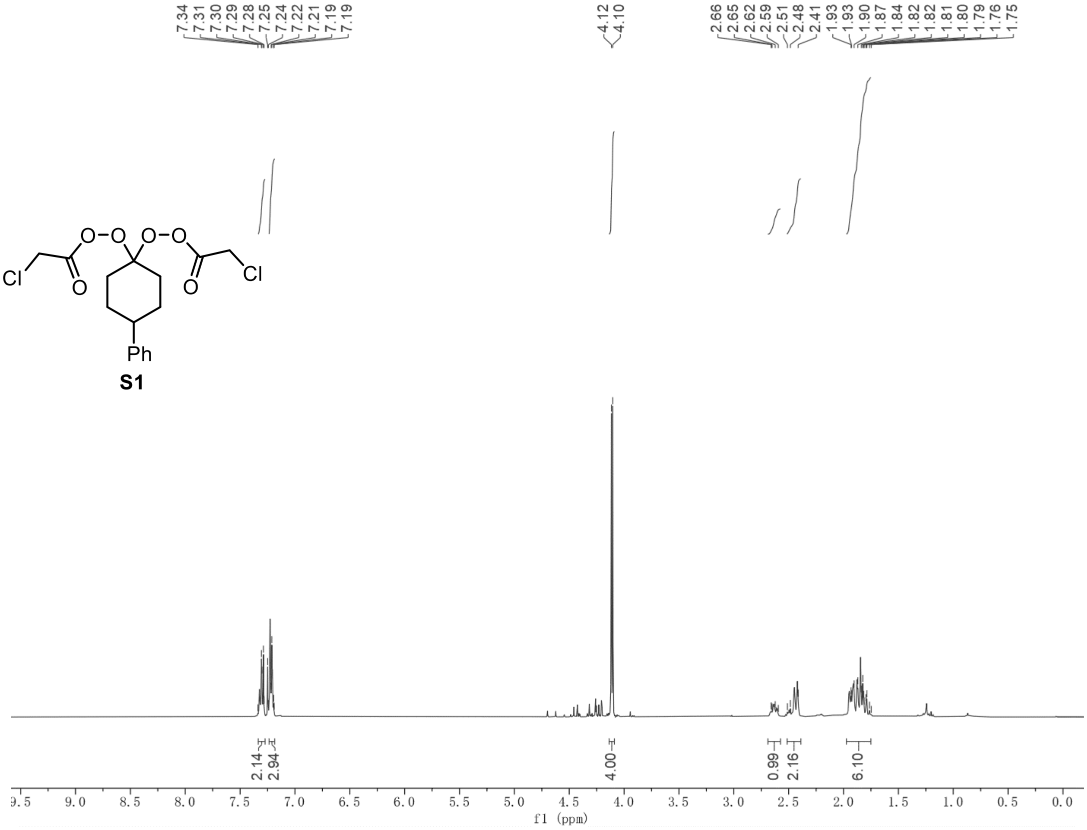

^{13}**C NMR** (100 MHz, $CDCl_3$) of **S1**

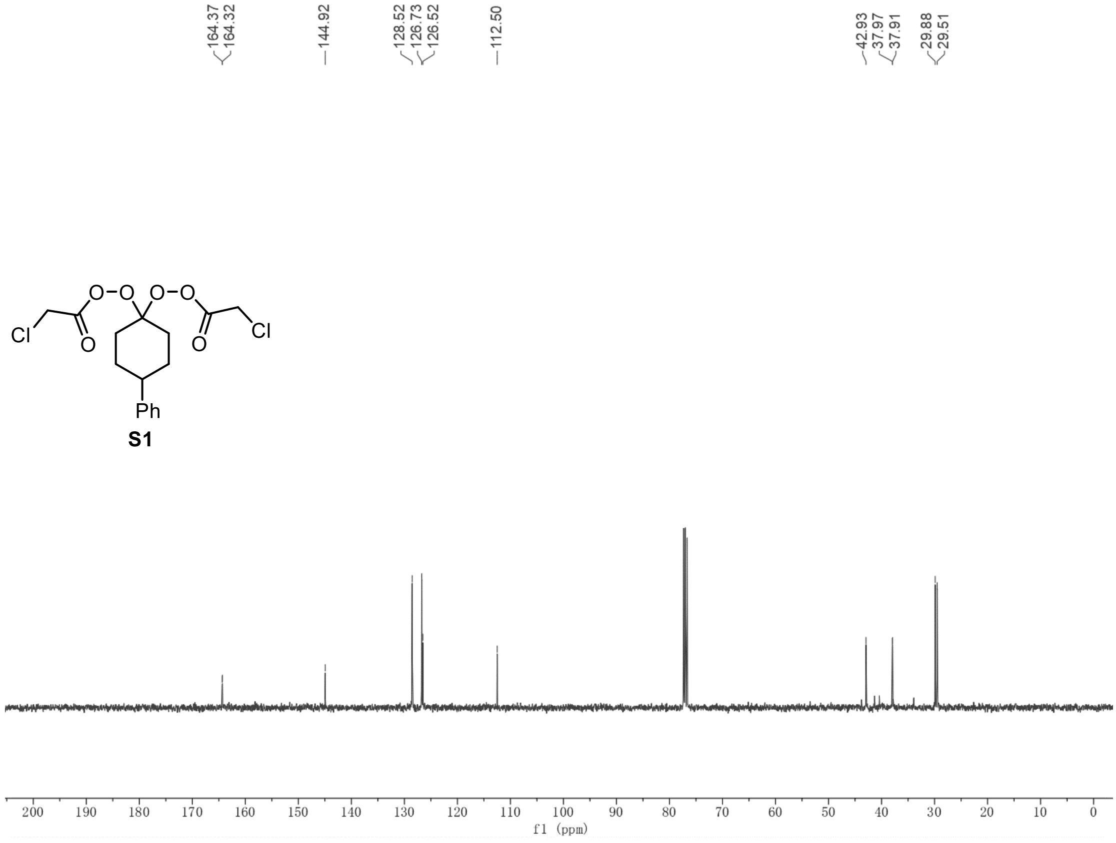

^{1}H NMR (400 MHz, $CDCl_3$) of **S2**

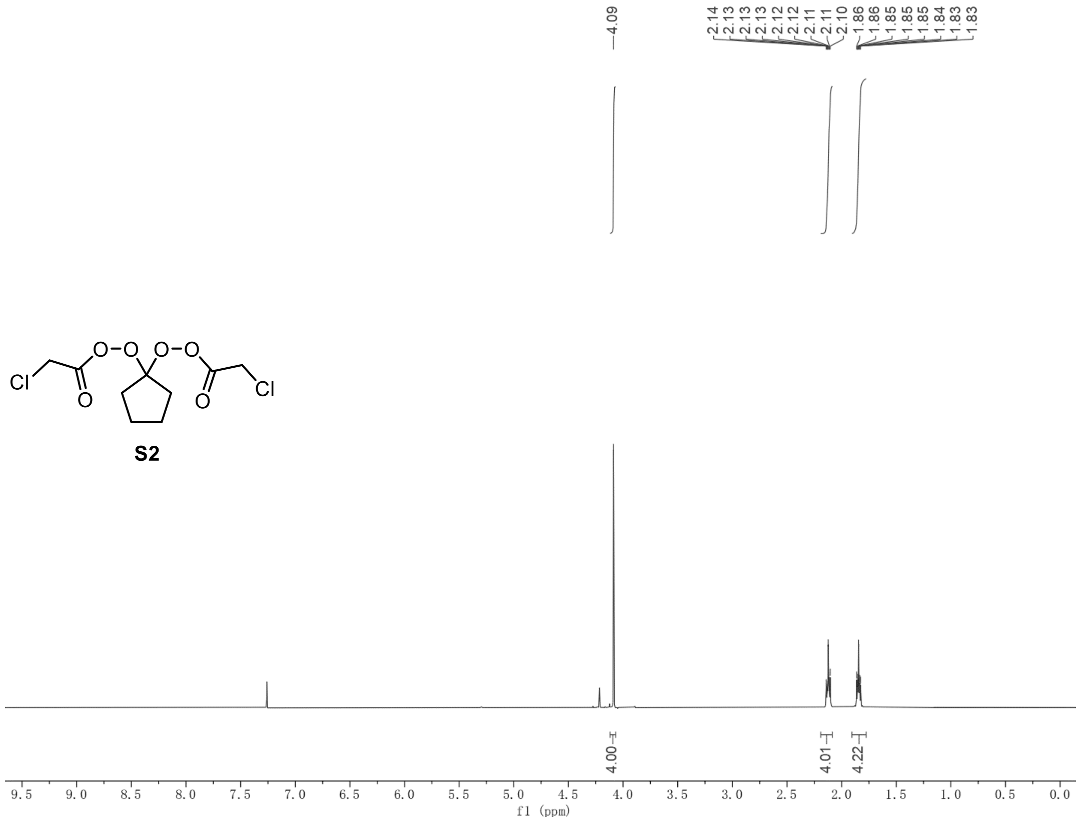

^{13}C NMR (100 MHz, $CDCl_3$) of **S2**

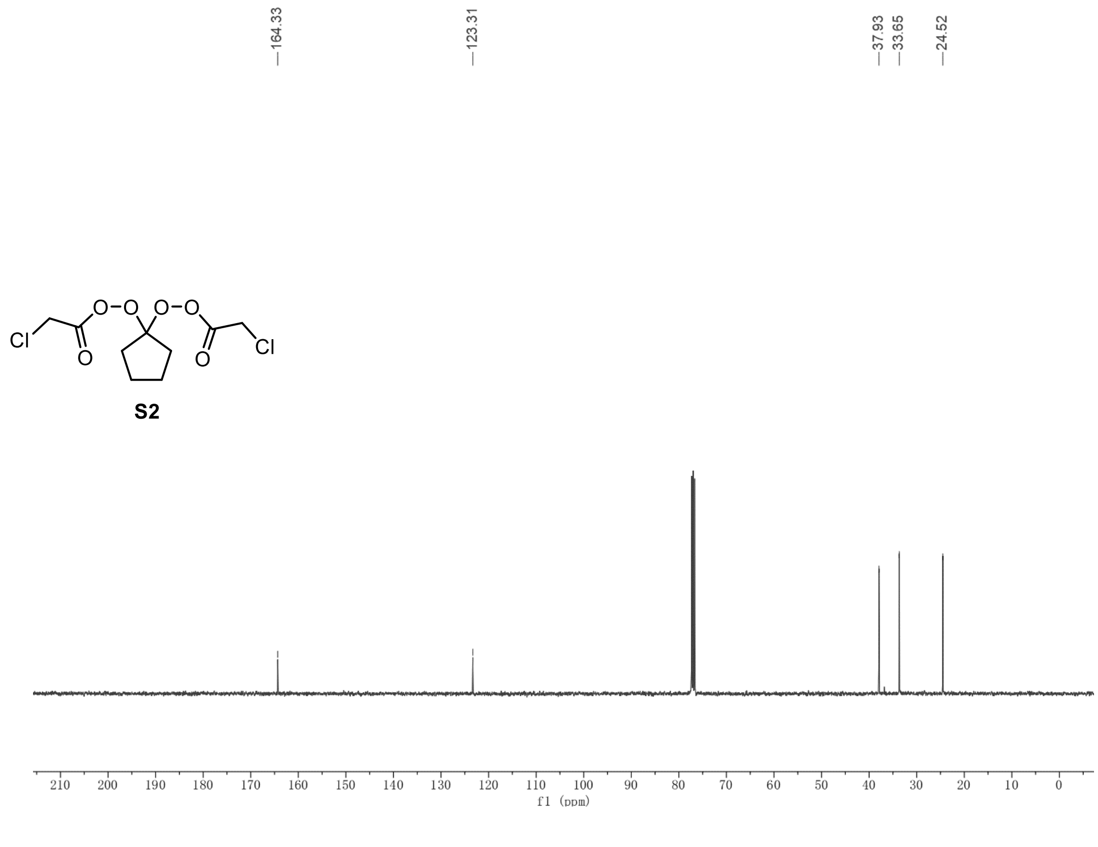

^{1}H NMR (400 MHz, $CDCl_3$) of **S3**

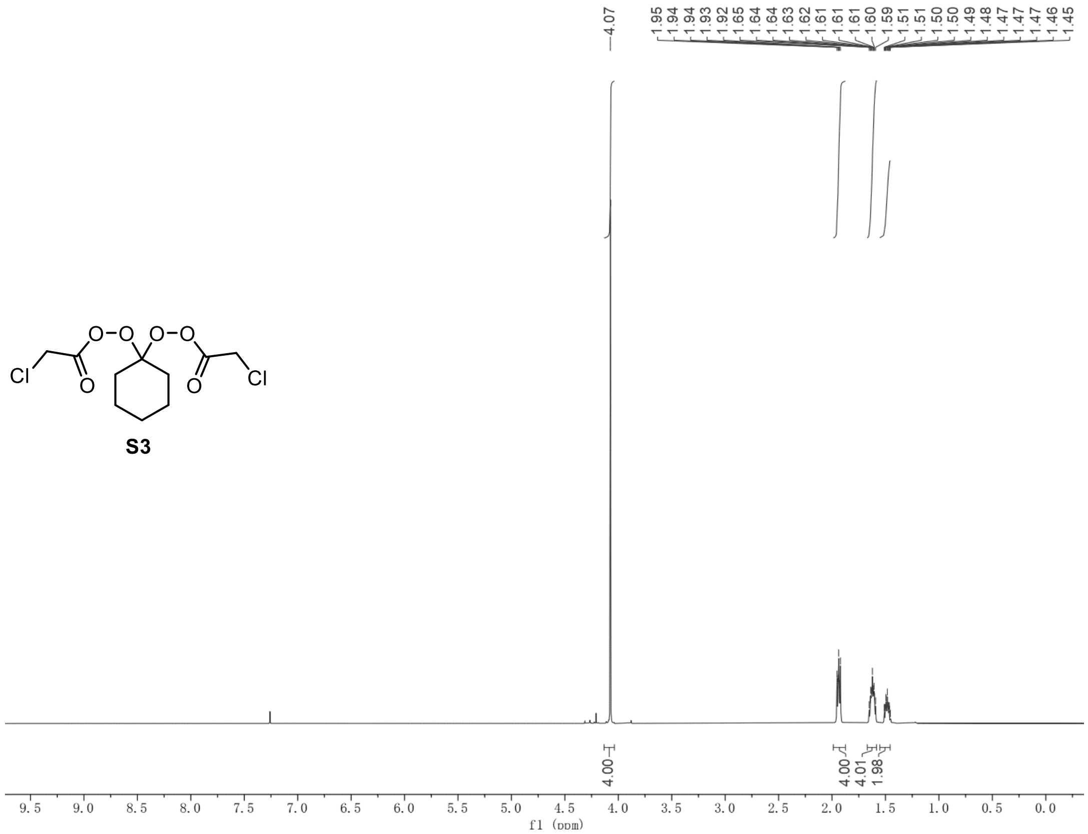

^{13}C NMR (100 MHz, $CDCl_3$) of **S3**

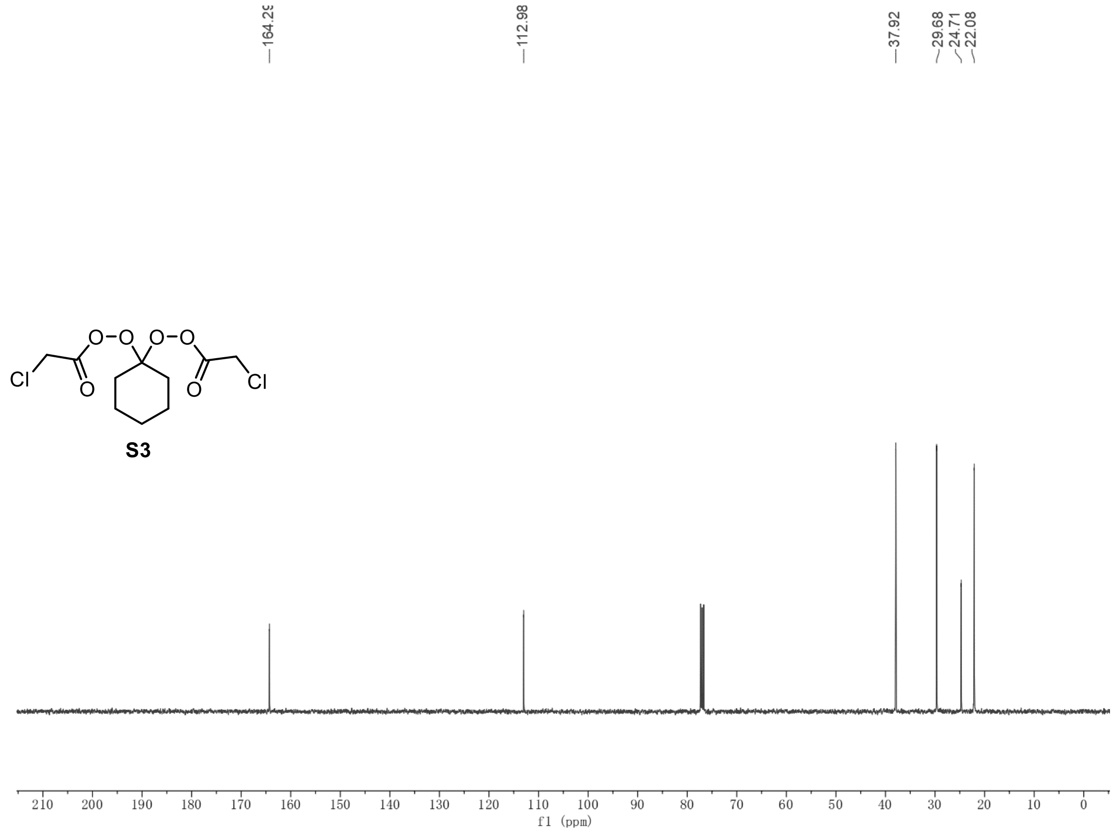

^{1}H NMR (400 MHz, $CDCl_3$) of **S4**

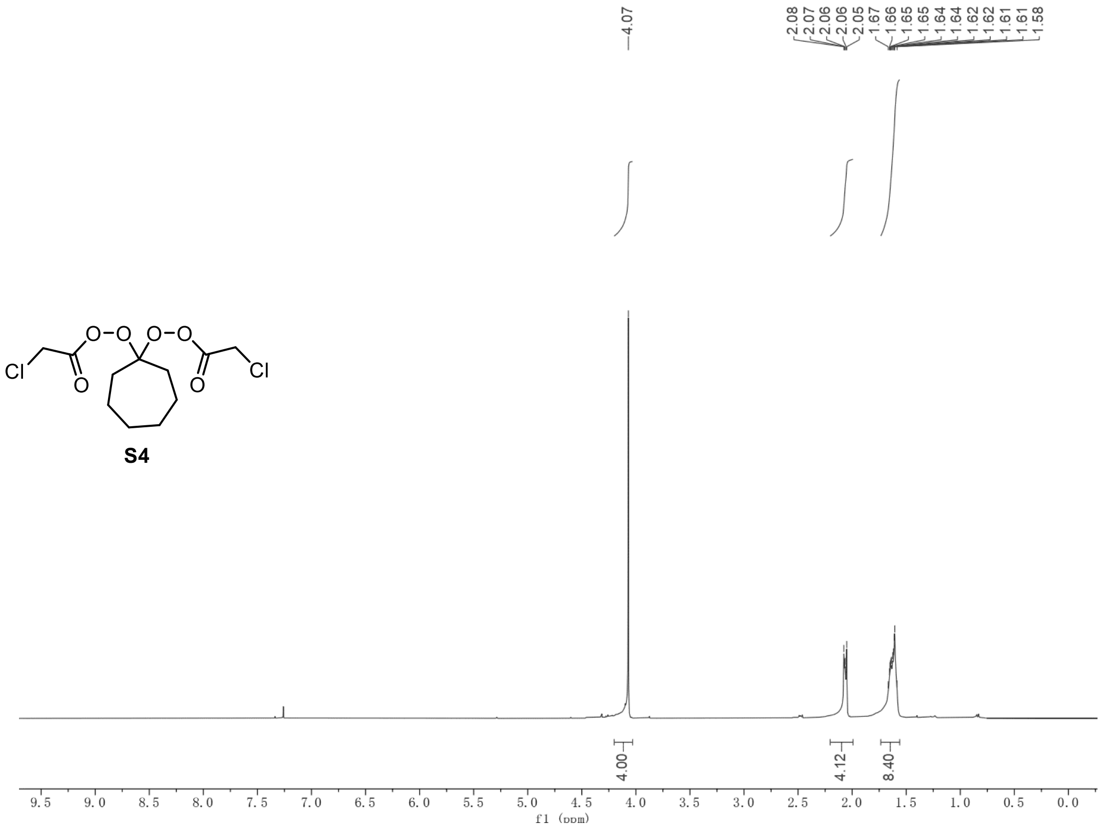

^{13}C NMR (100 MHz, $CDCl_3$) of **S4**

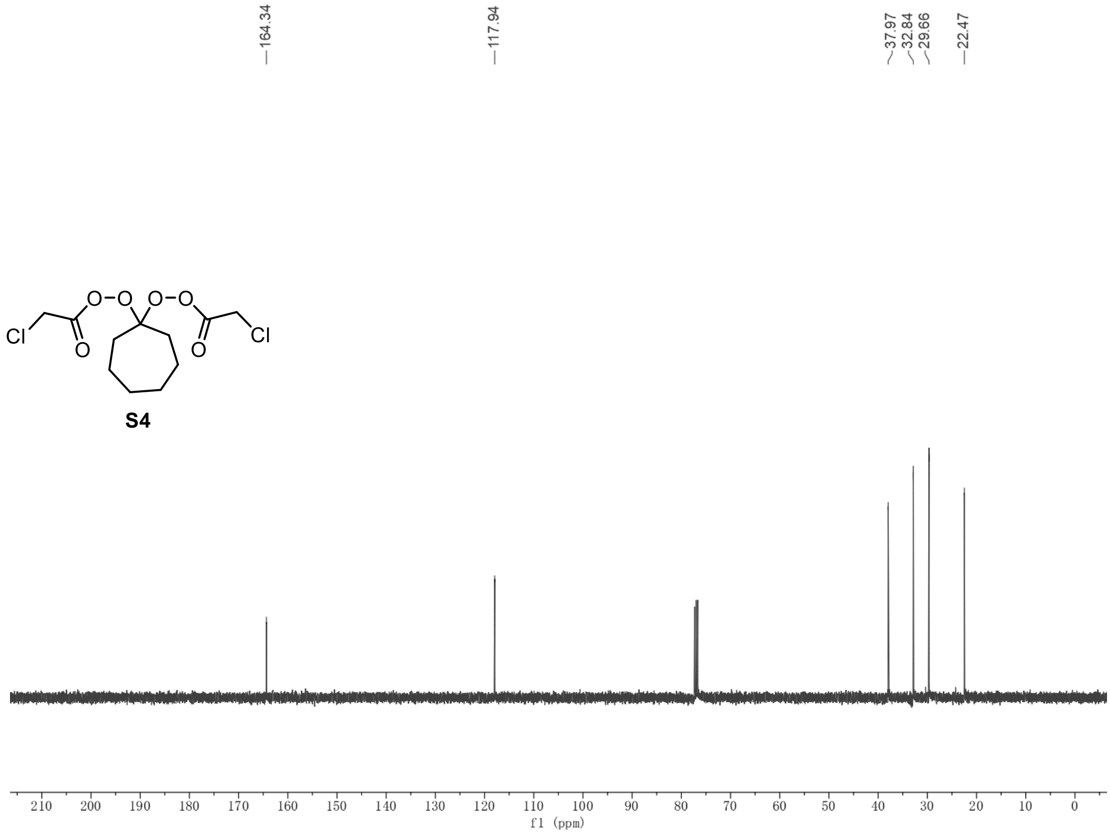

^{1}H NMR (400 MHz, $CDCl_3$) of **S5**

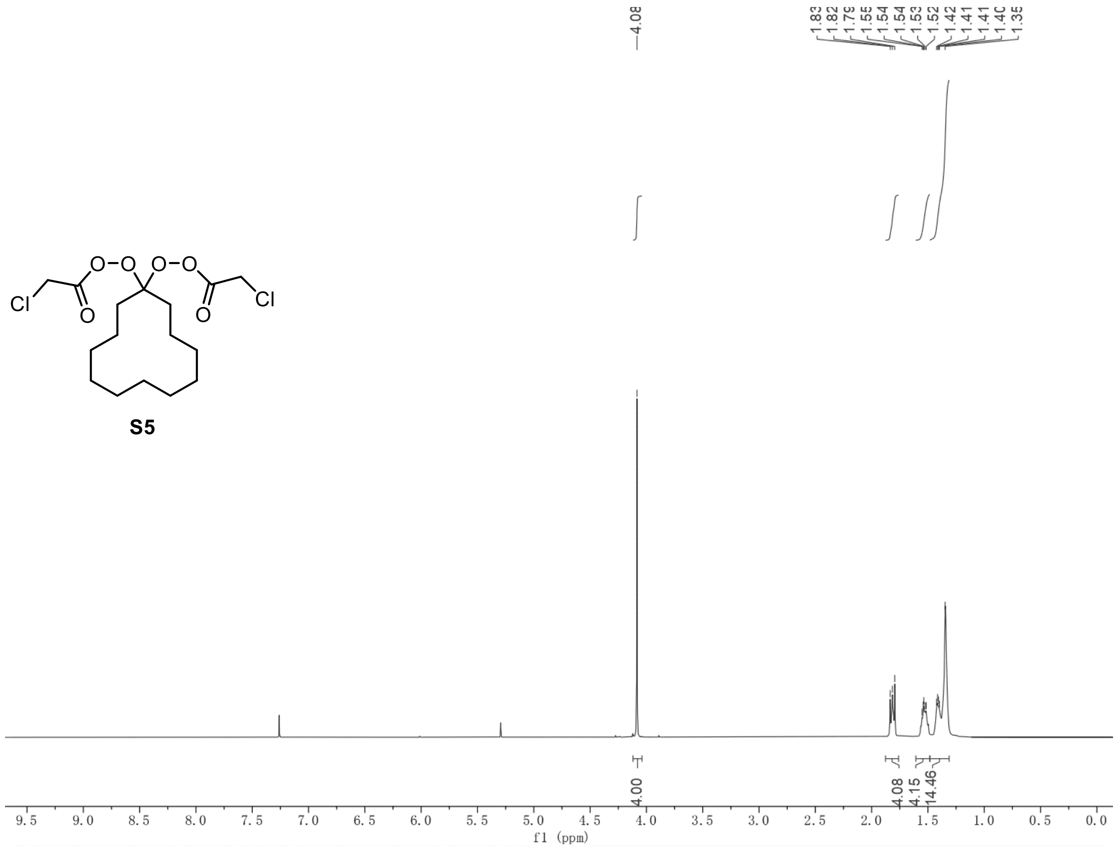

^{13}C NMR (100 MHz, $CDCl_3$) of **S5**

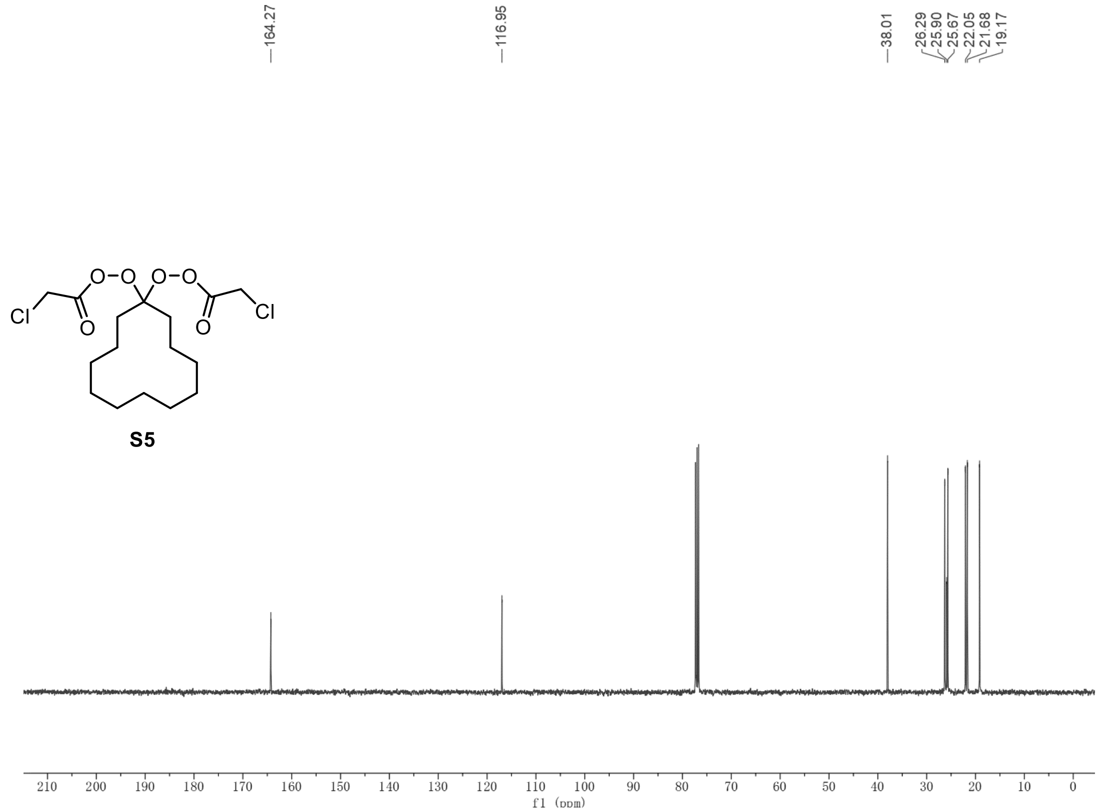

^{1}H NMR (400 MHz, $CDCl_3$) of **S6**

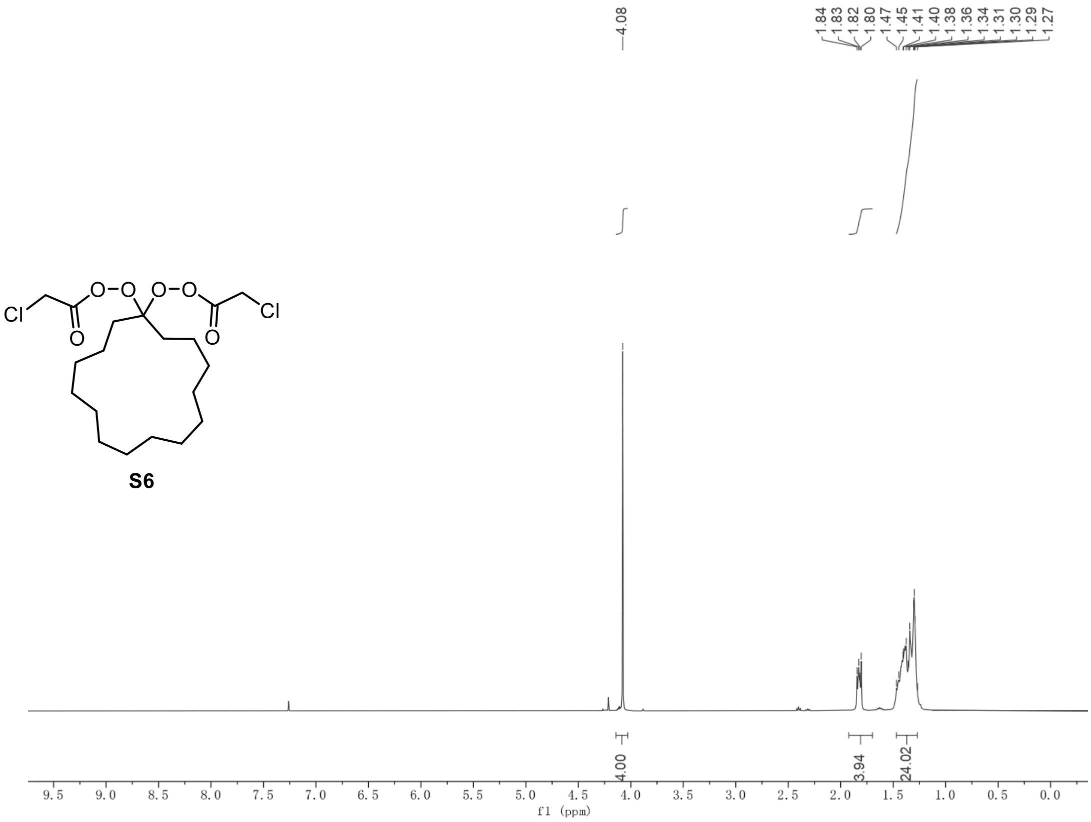

^{13}C NMR (100 MHz, $CDCl_3$) of **S6**

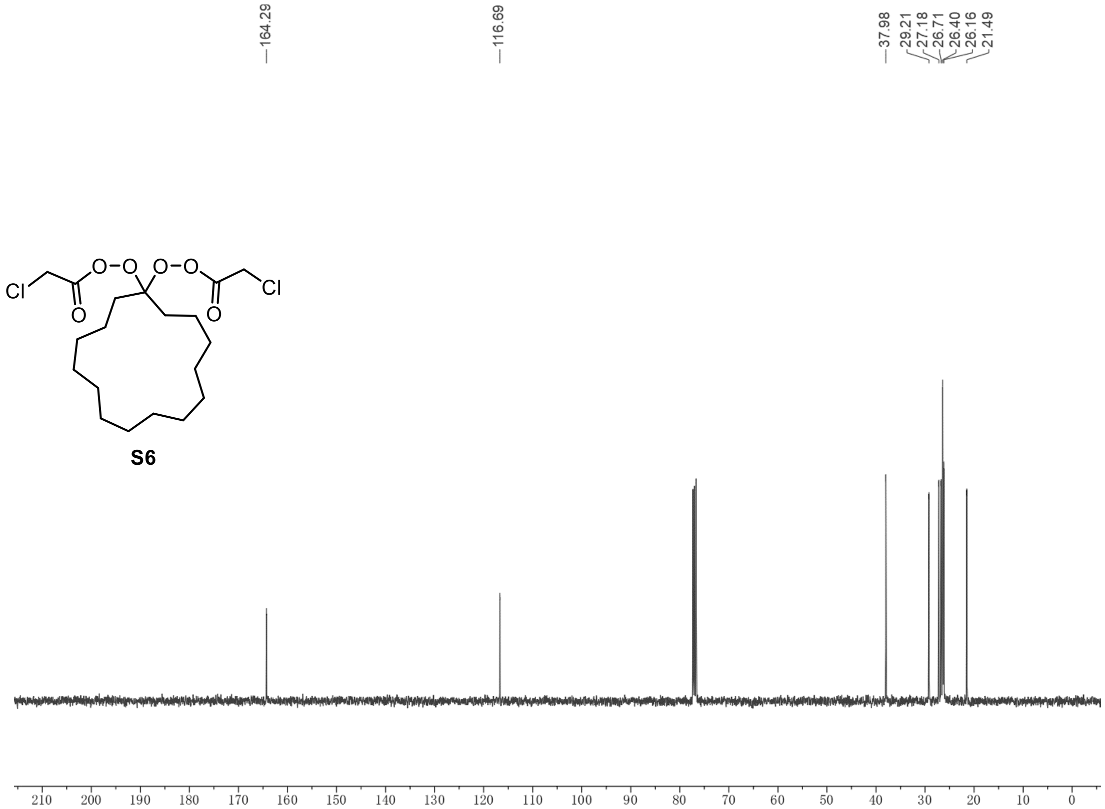

^{1}H NMR (400 MHz, $CDCl_3$) of **S7**

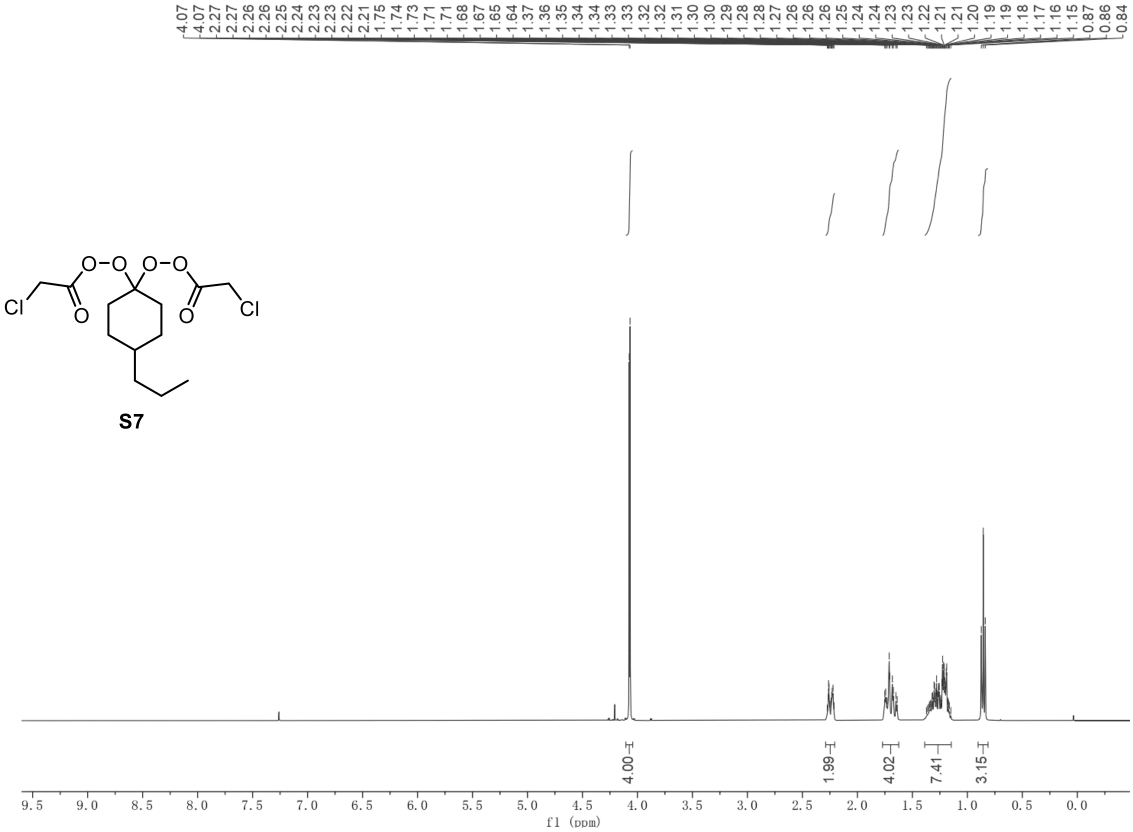

^{13}C NMR (100 MHz, $CDCl_3$) of **S7**

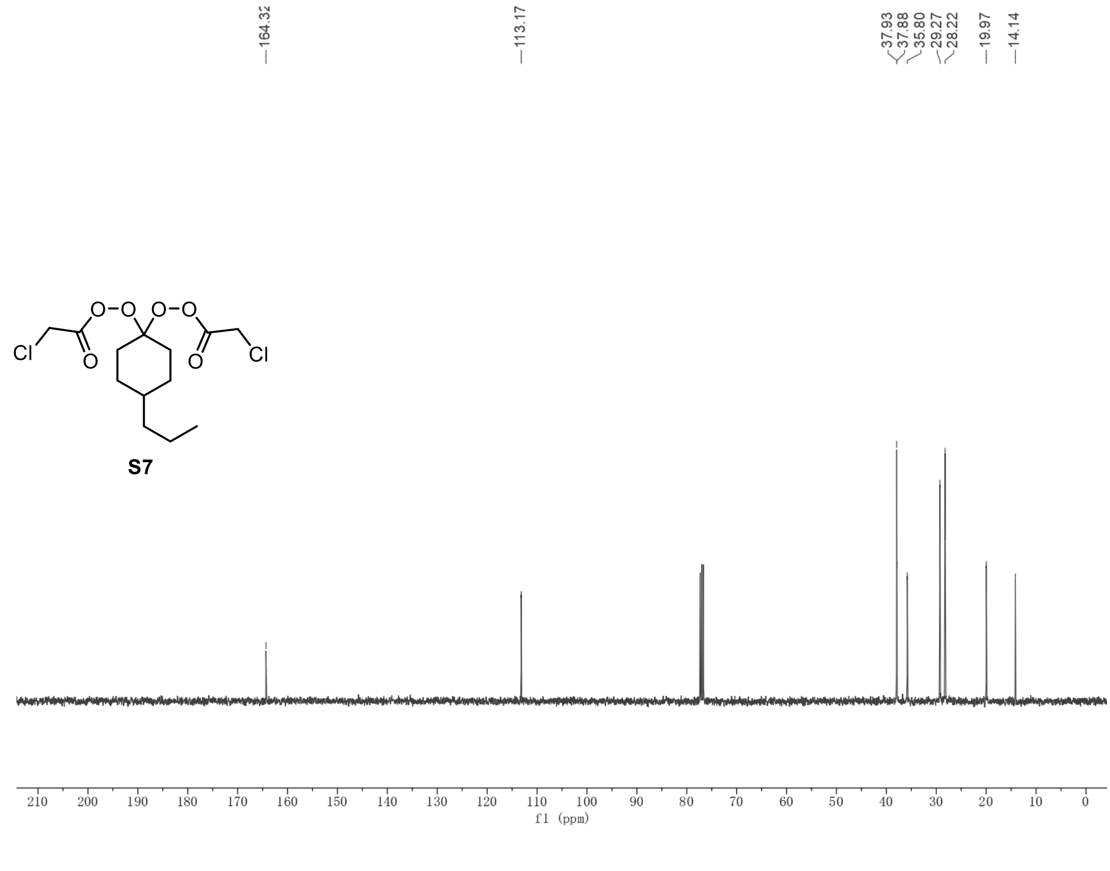

¹H NMR (400 MHz, $CDCl_3$) of **S8**

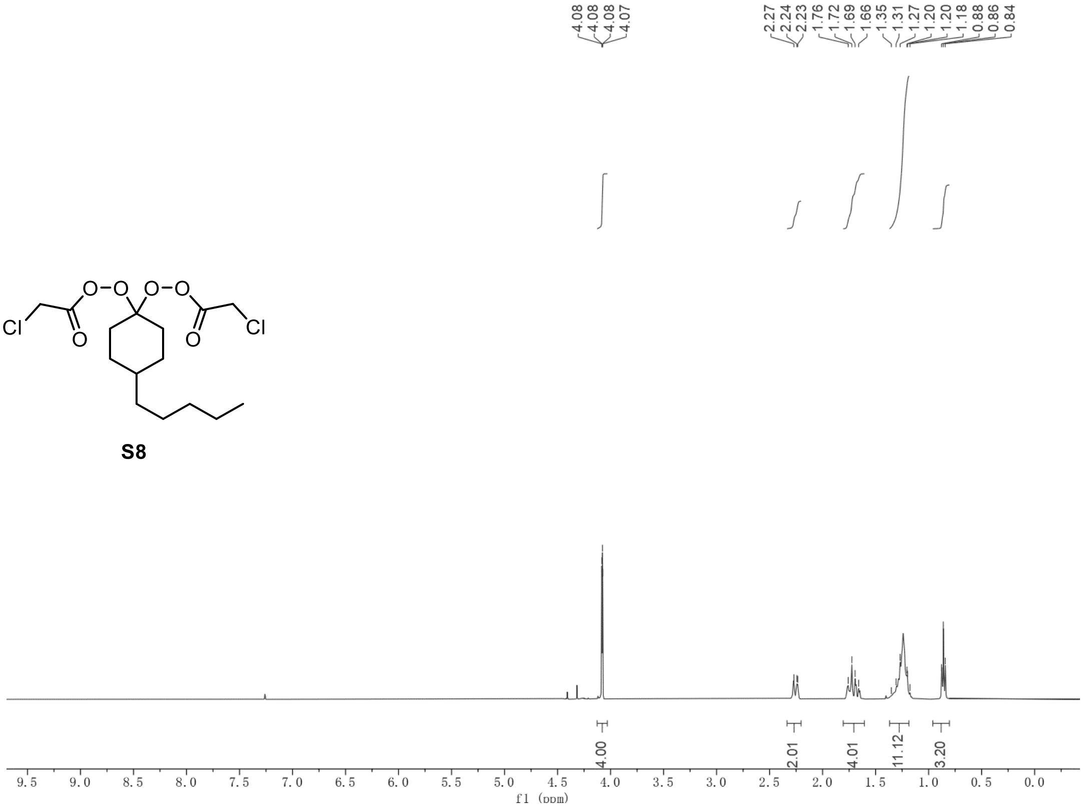

¹³C NMR (100 MHz, $CDCl_3$) of **S8**

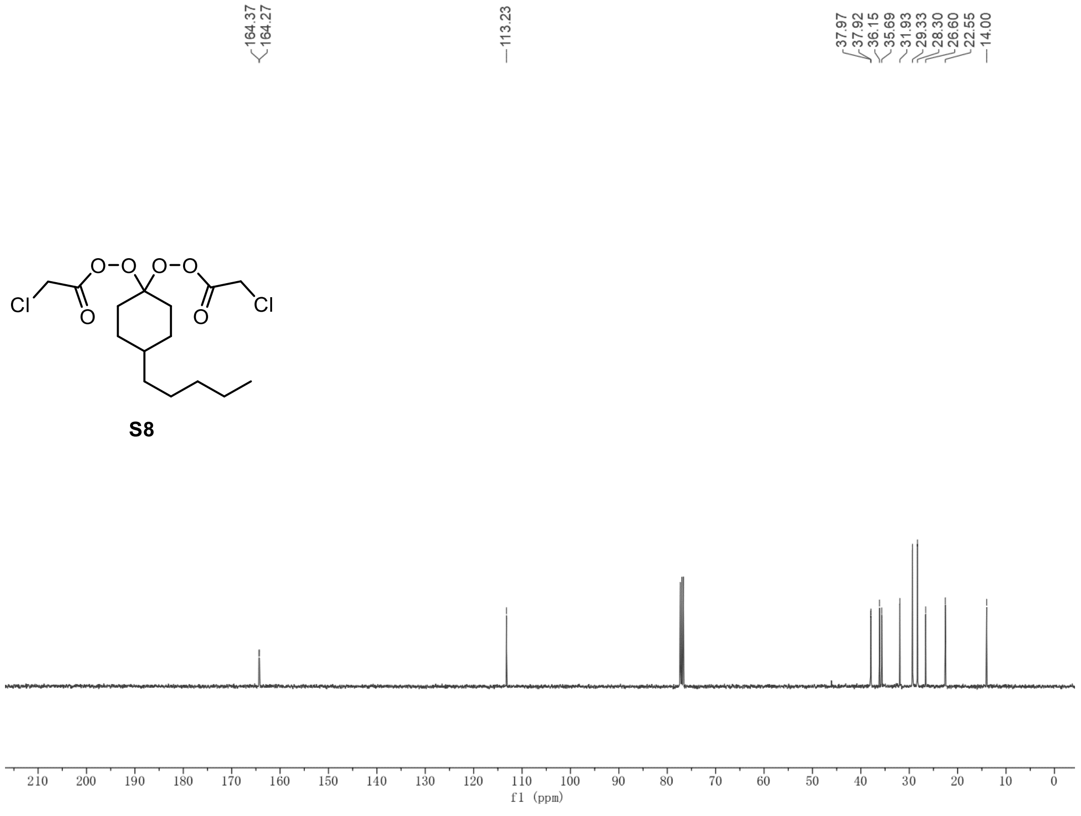

^{1}H NMR (400 MHz, $CDCl_3$) of **S9**

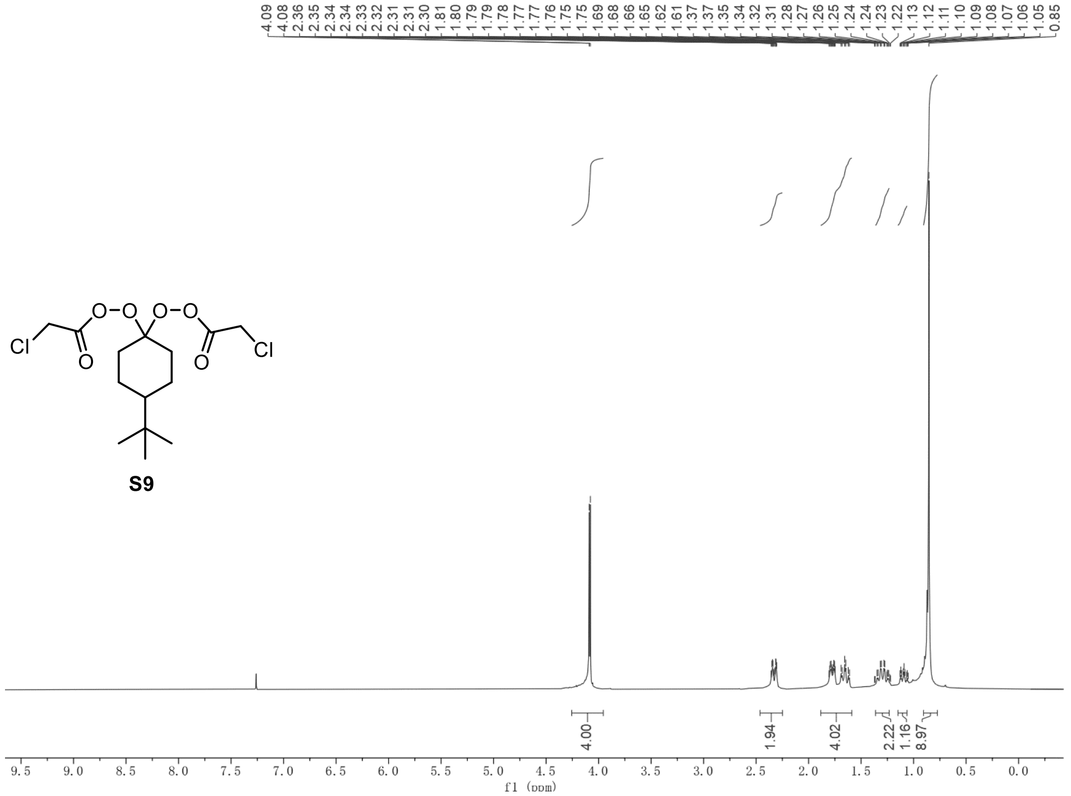

^{13}C NMR (100 MHz, $CDCl_3$) of **S9**

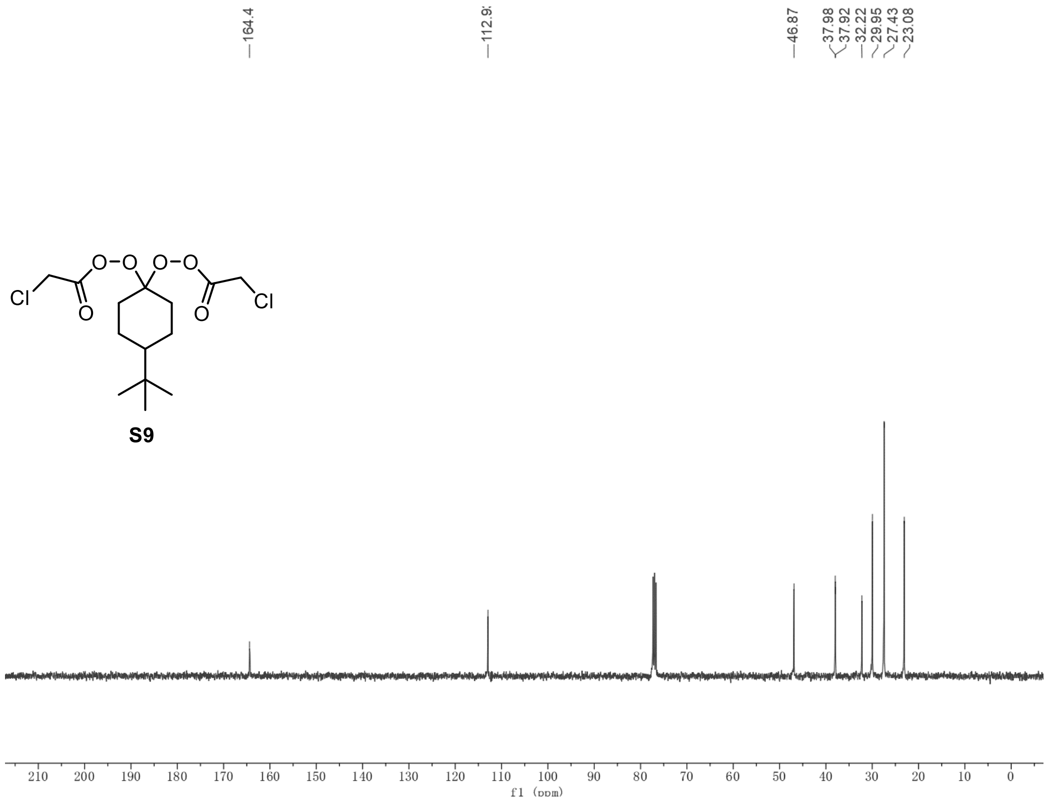

^{1}H NMR (400 MHz, $CDCl_3$) of **S10**

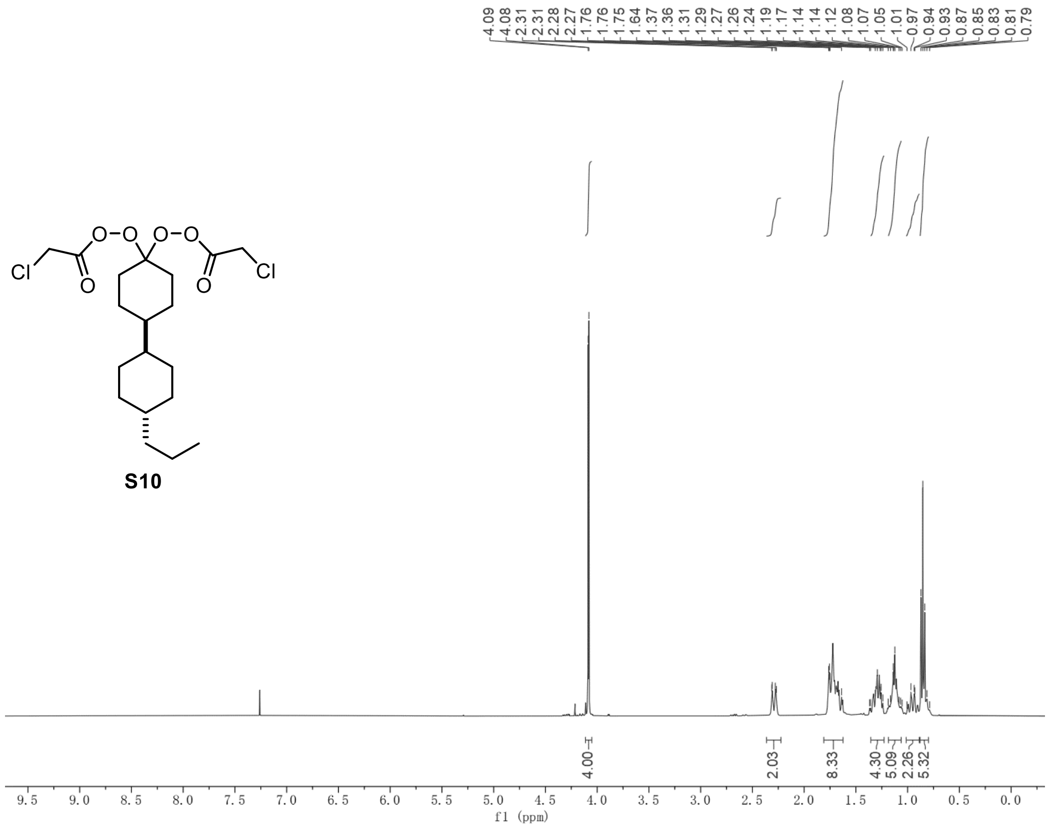

^{13}C NMR (100 MHz, $CDCl_3$) of **S10**

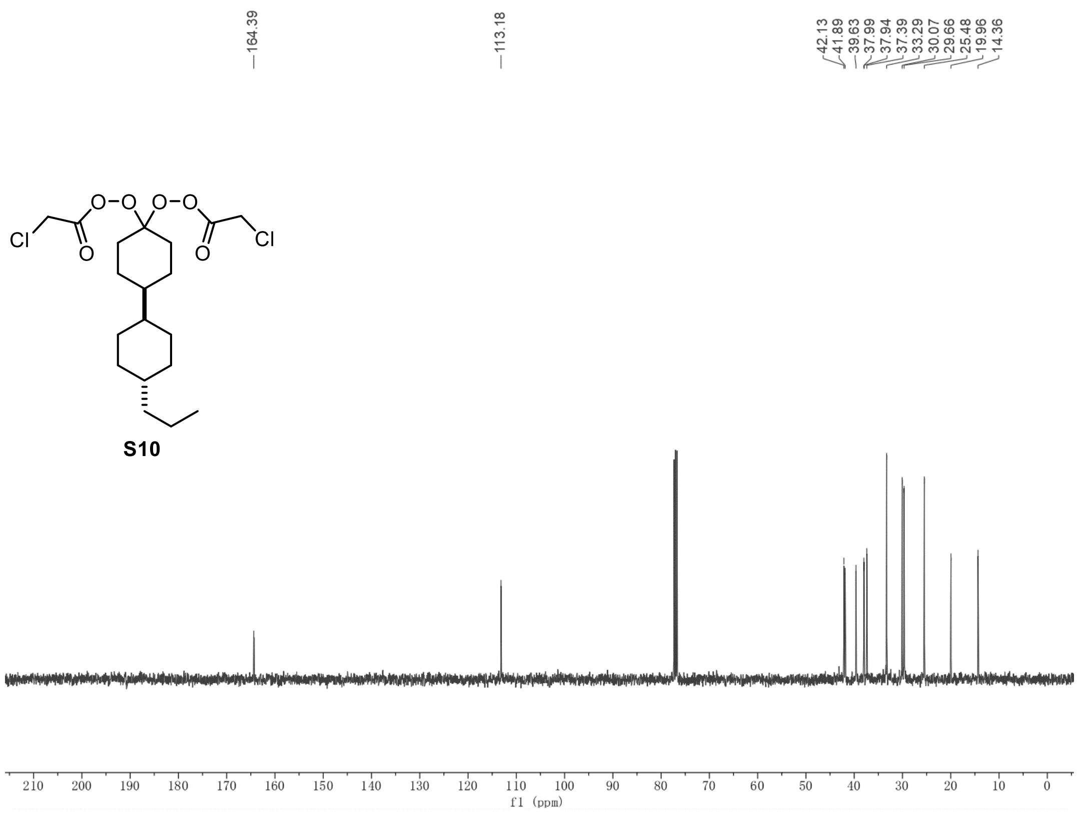

^{1}H NMR (400 MHz, $CDCl_3$) of **S11**

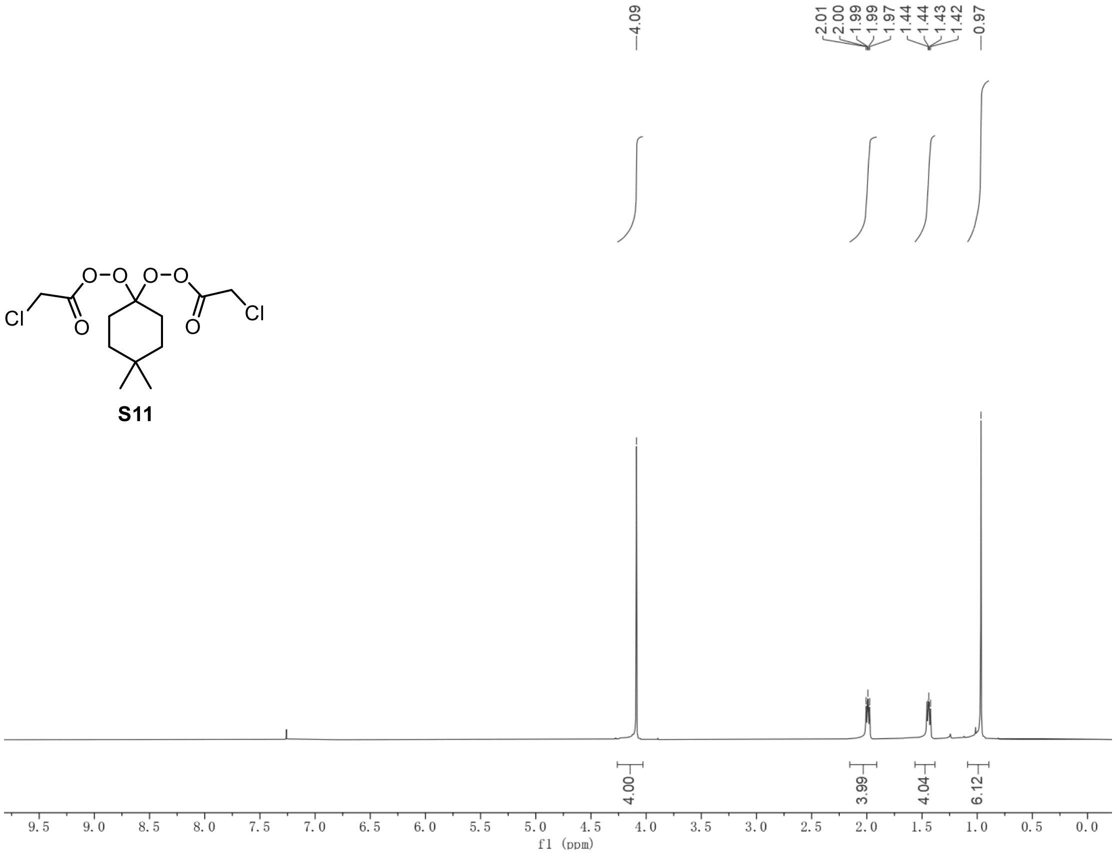

^{13}C NMR (100 MHz, $CDCl_3$) of **S11**

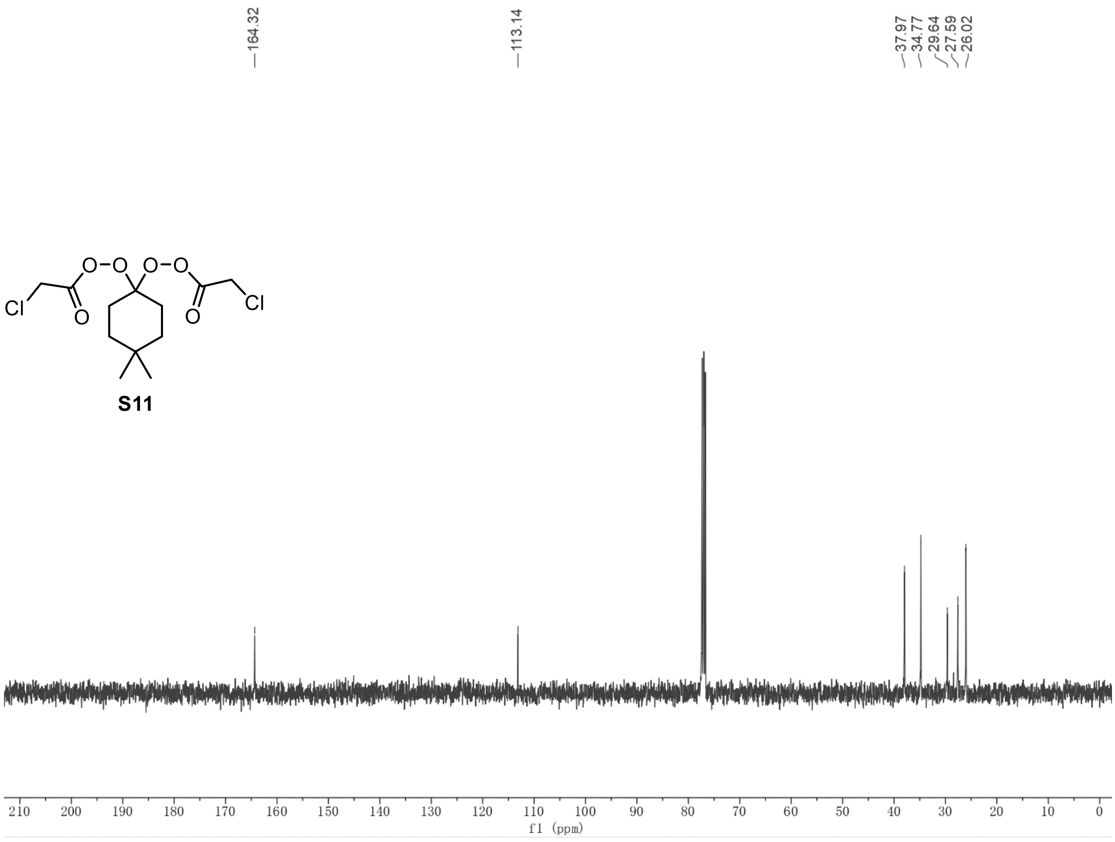

¹H NMR (400 MHz, $CDCl_3$) of **S12**

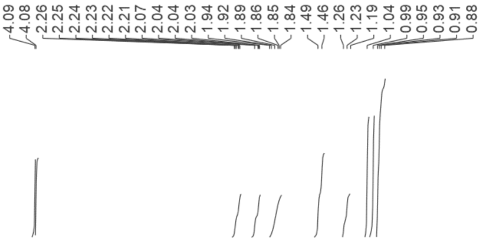

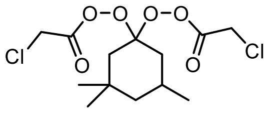

S12

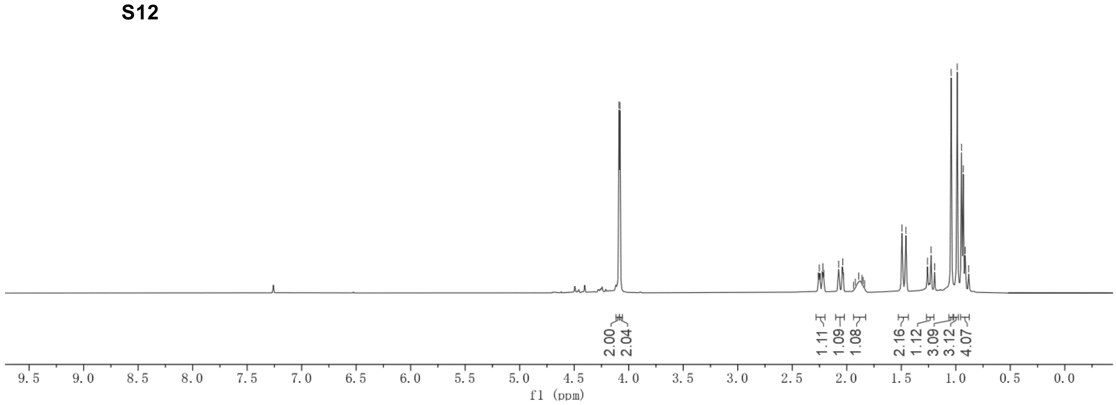

¹³C NMR (100 MHz, $CDCl_3$) of **S12**

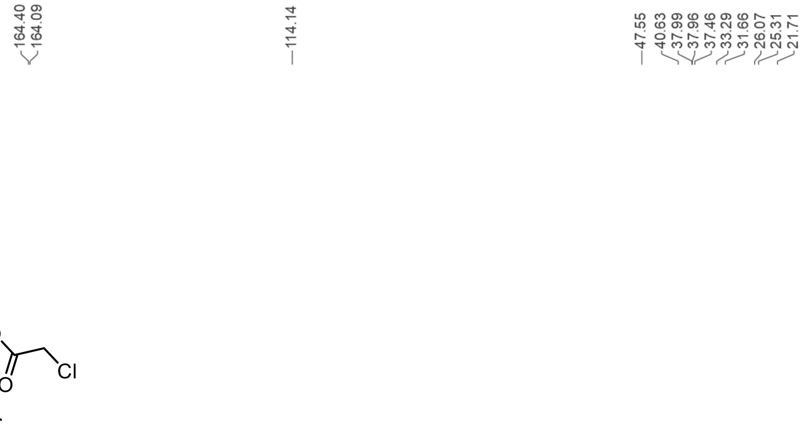

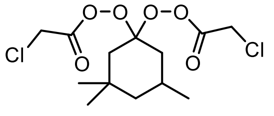

S12

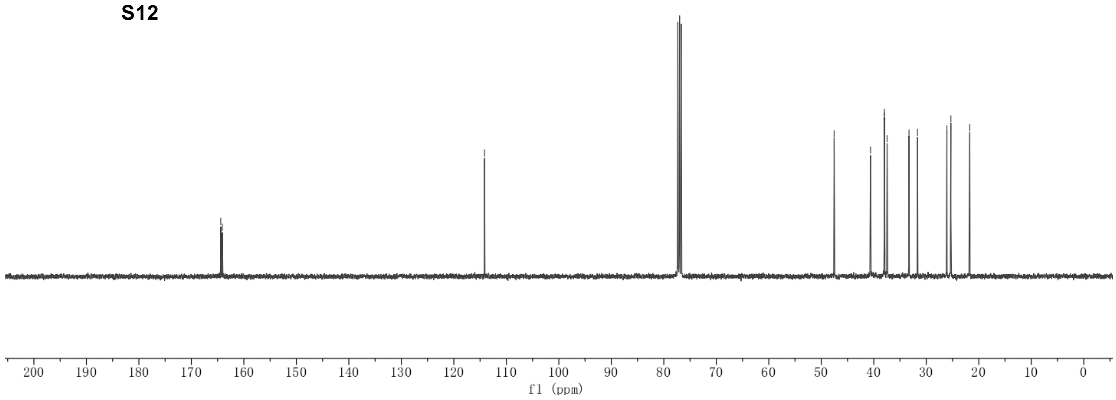

^{1}H NMR (400 MHz, $CDCl_3$) of **S13**

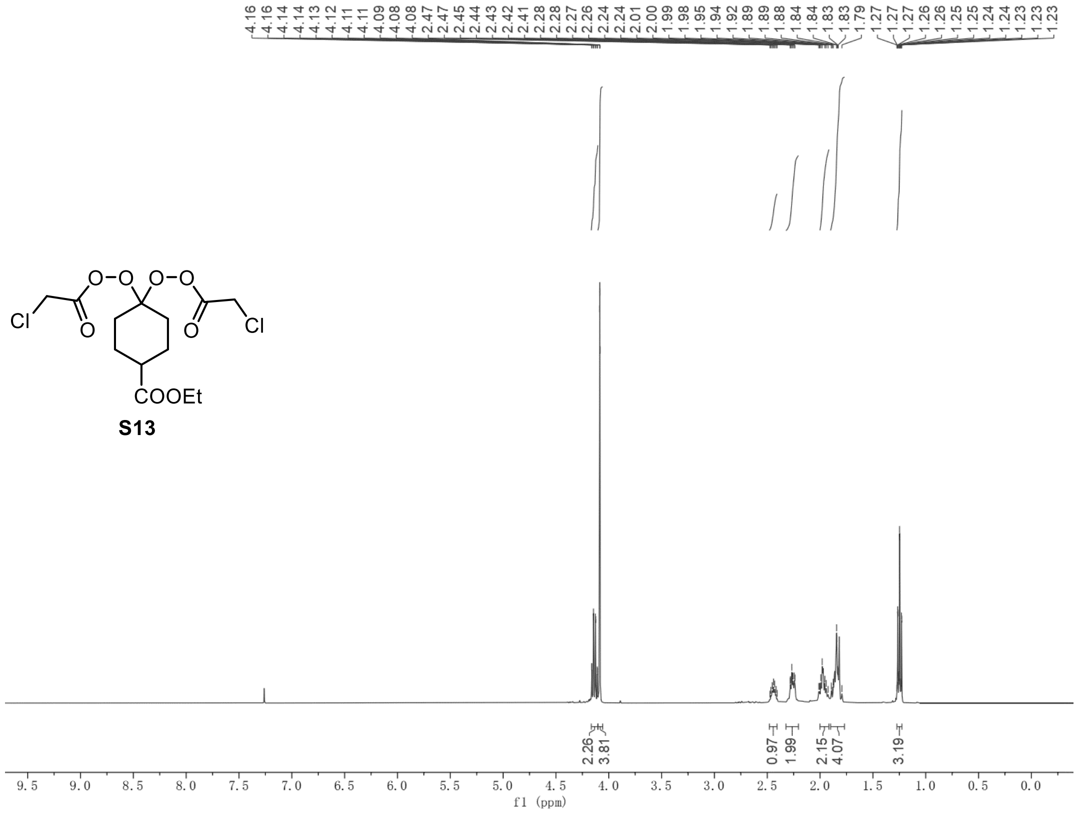

S13

^{13}C NMR (100 MHz, $CDCl_3$) of **S13**

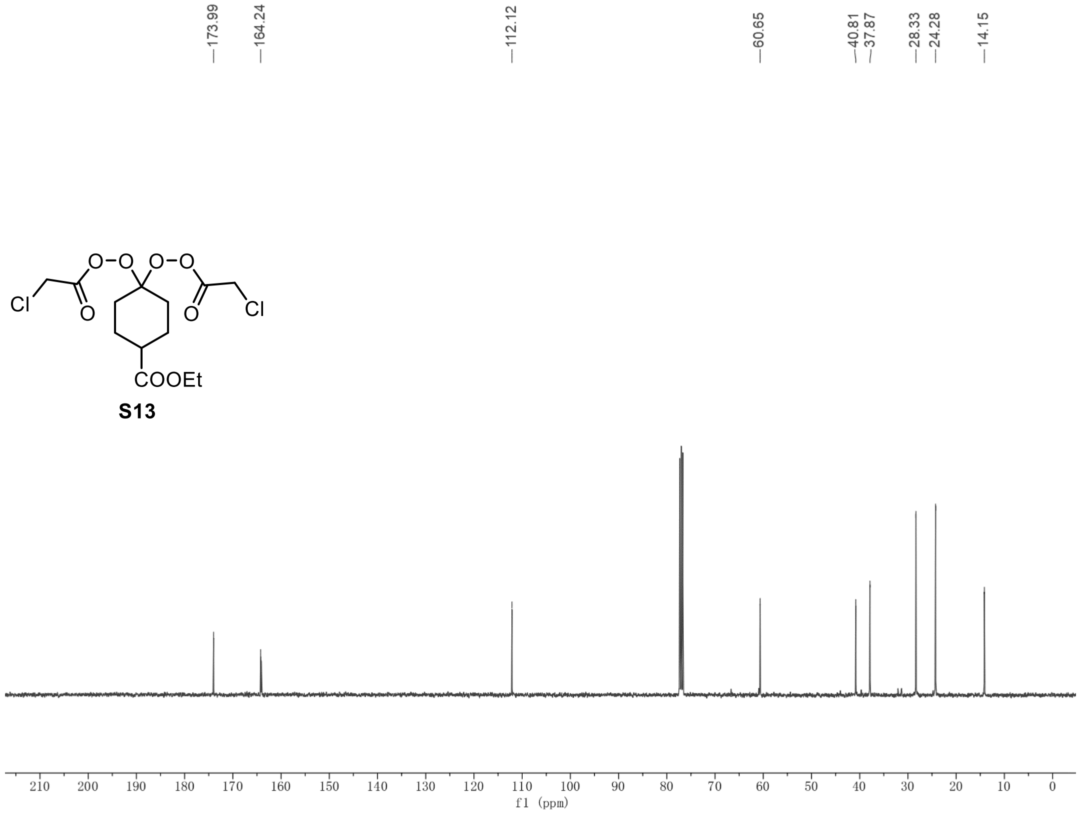

S13

^{1}H NMR (400 MHz, $CDCl_3$) of **S14**

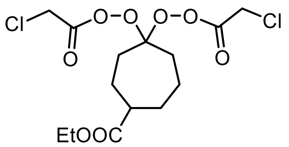

S14

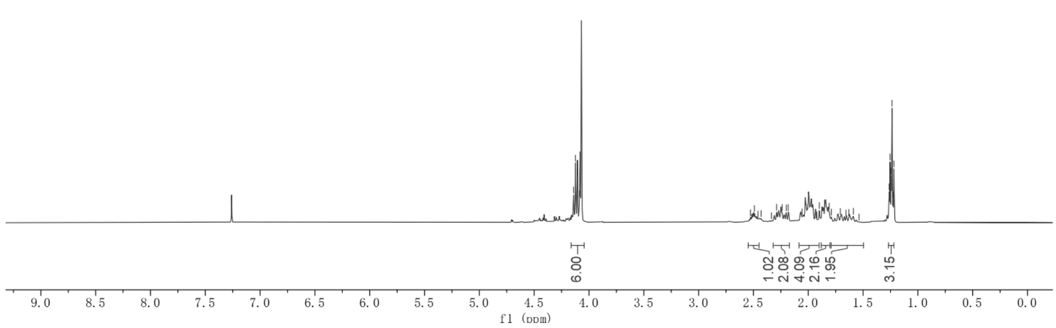

^{13}C NMR (100 MHz, $CDCl_3$) of **S14**

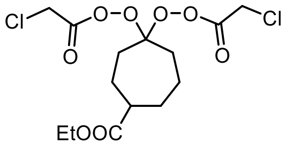

S14

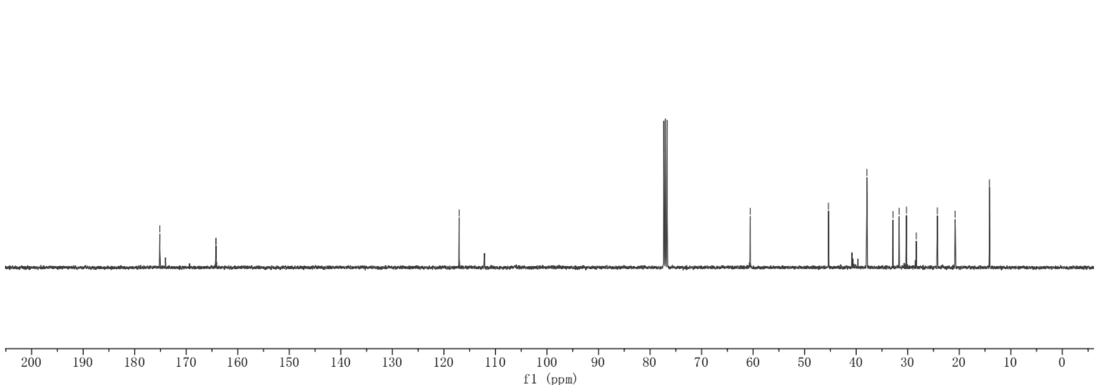

^{1}H NMR (400 MHz, $CDCl_3$) of **S15**

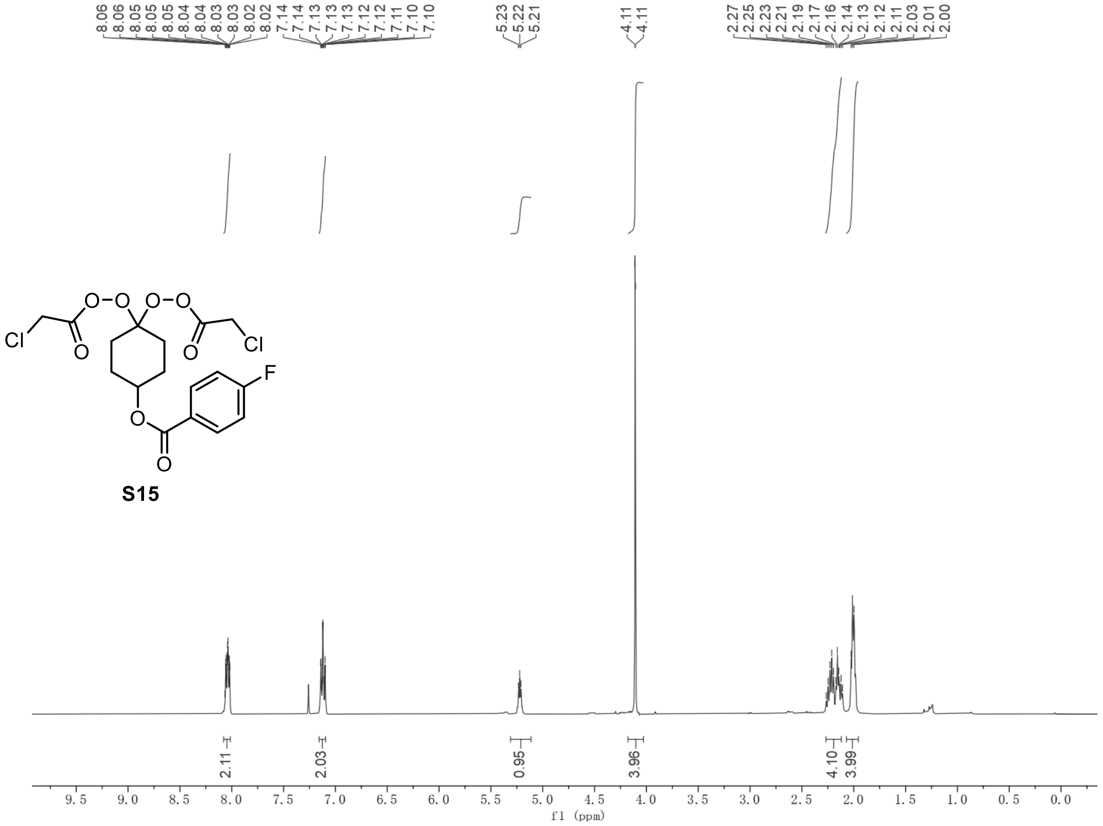

^{19}F NMR (376 MHz, $CDCl_3$) of **S15**

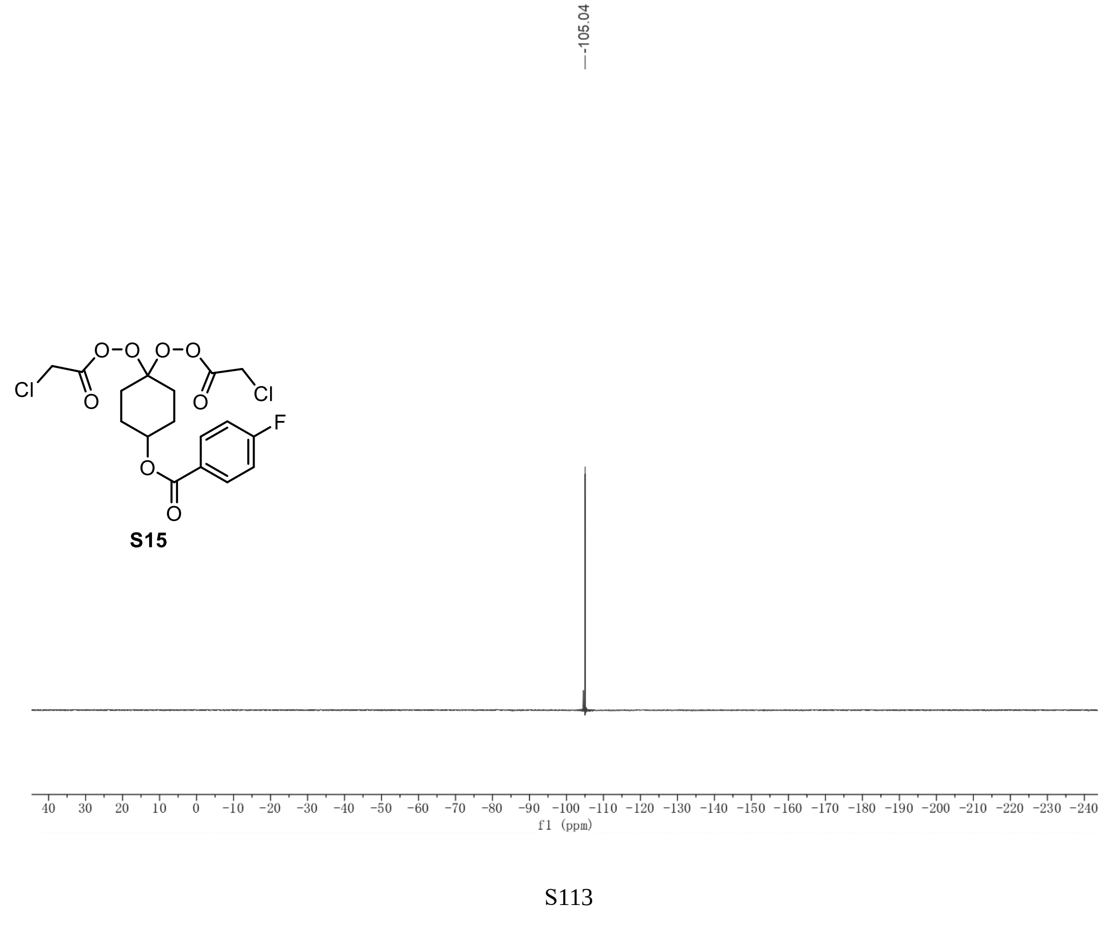

^{13}C NMR (100 MHz, $CDCl_3$) of **S15**

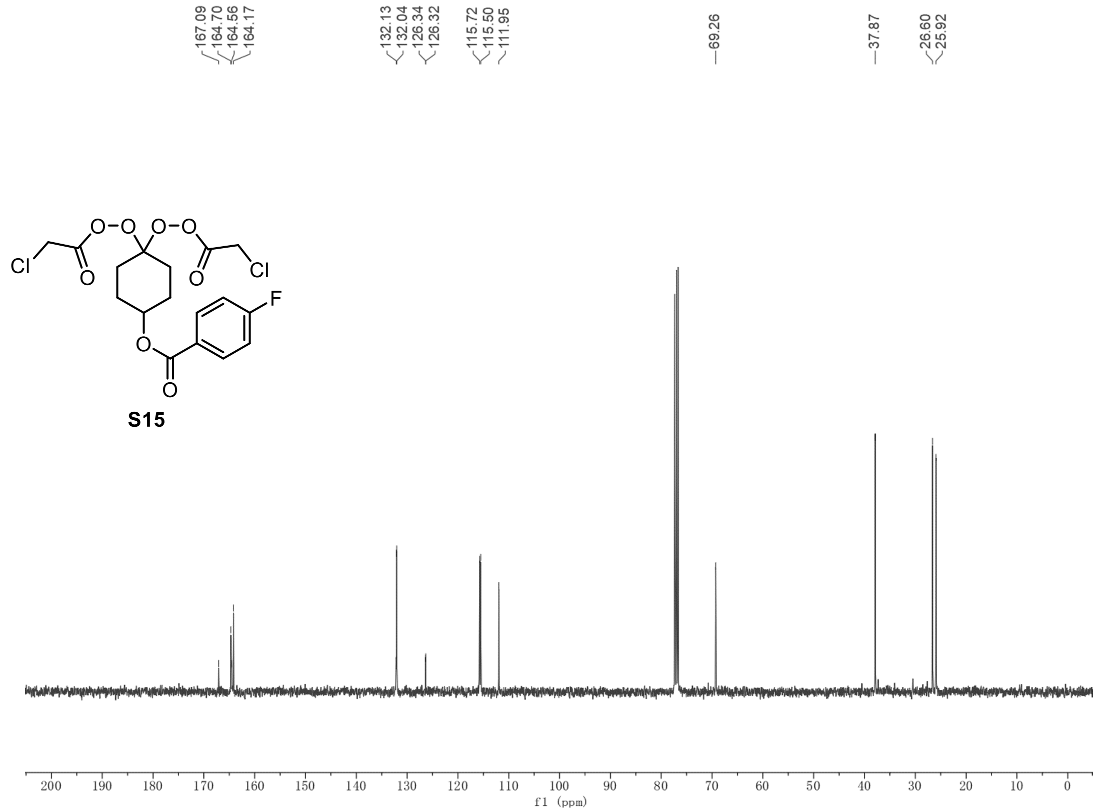

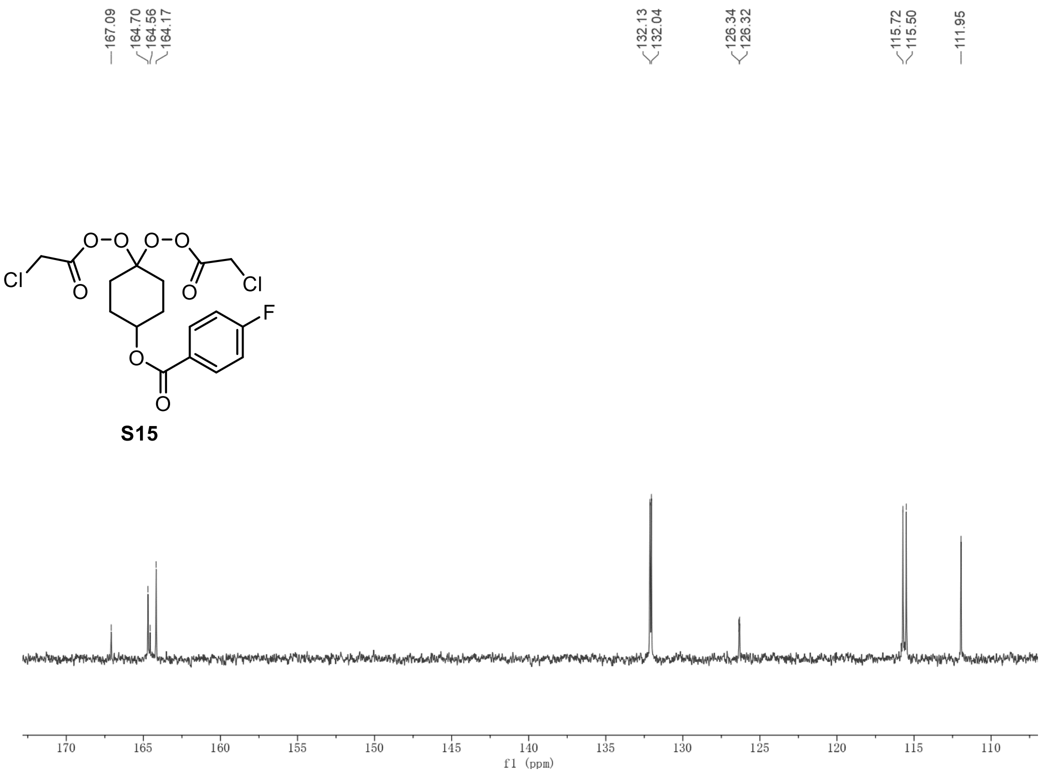

^{1}H NMR (400 MHz, $CDCl_3$) of **S16**

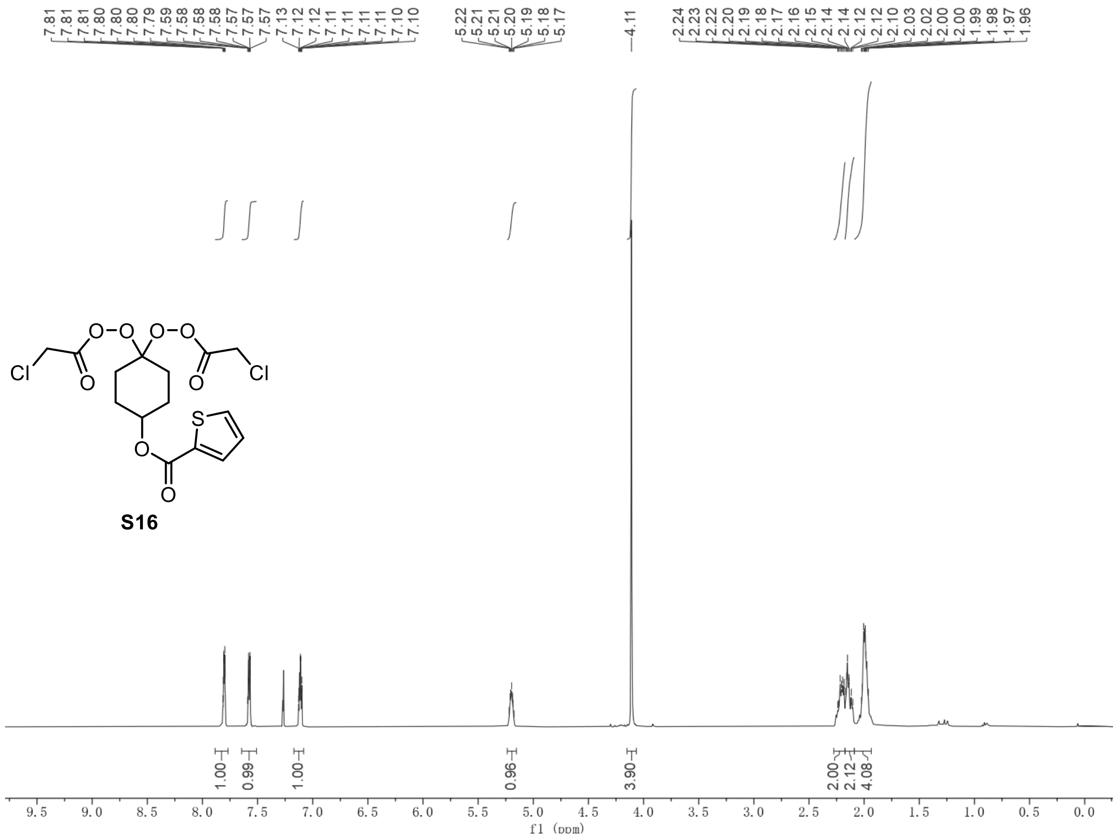

^{13}C NMR (100 MHz, $CDCl_3$) of **S16**

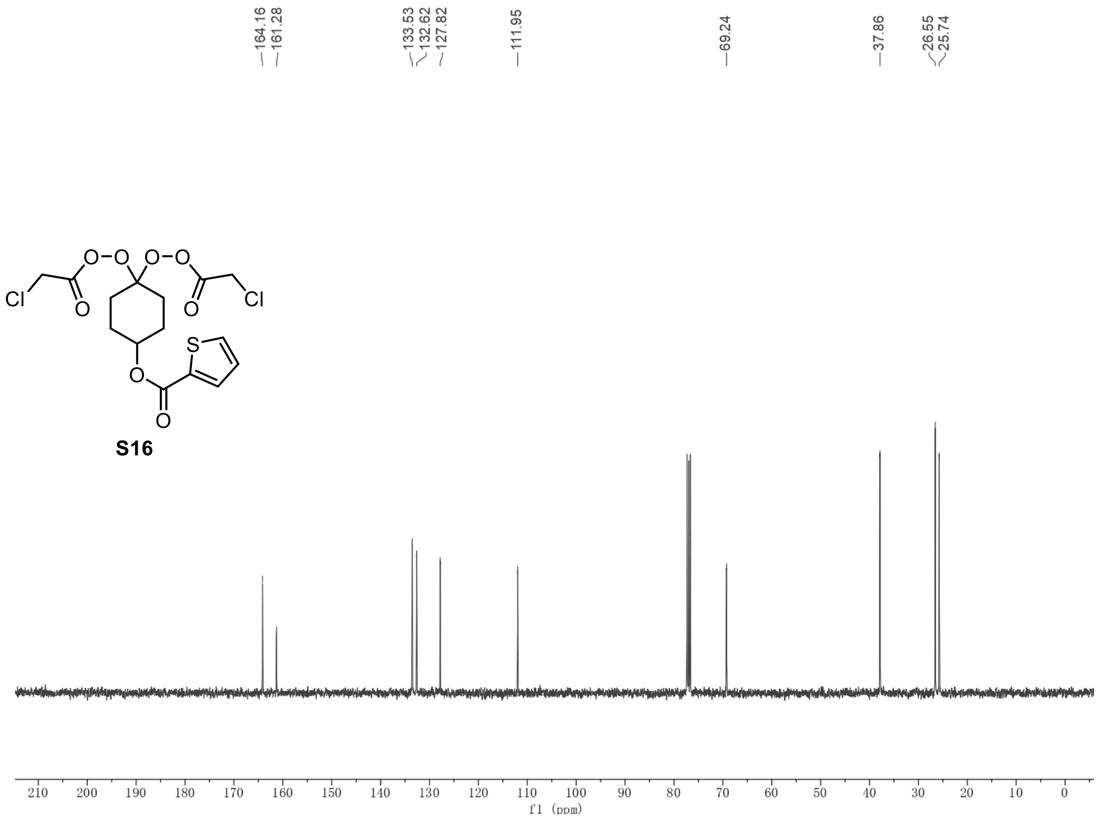

^{1}H NMR (400 MHz, $CDCl_3$) of **S17**

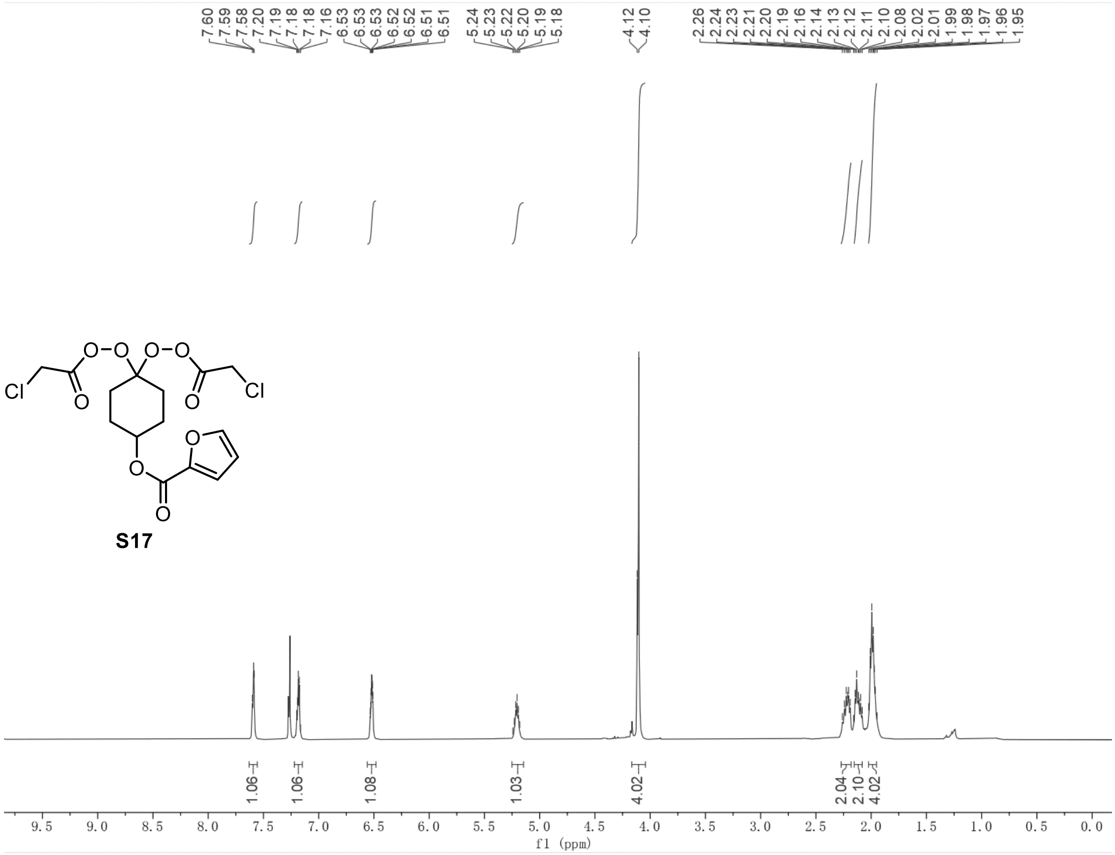

^{13}C NMR (100 MHz, $CDCl_3$) of **S17**

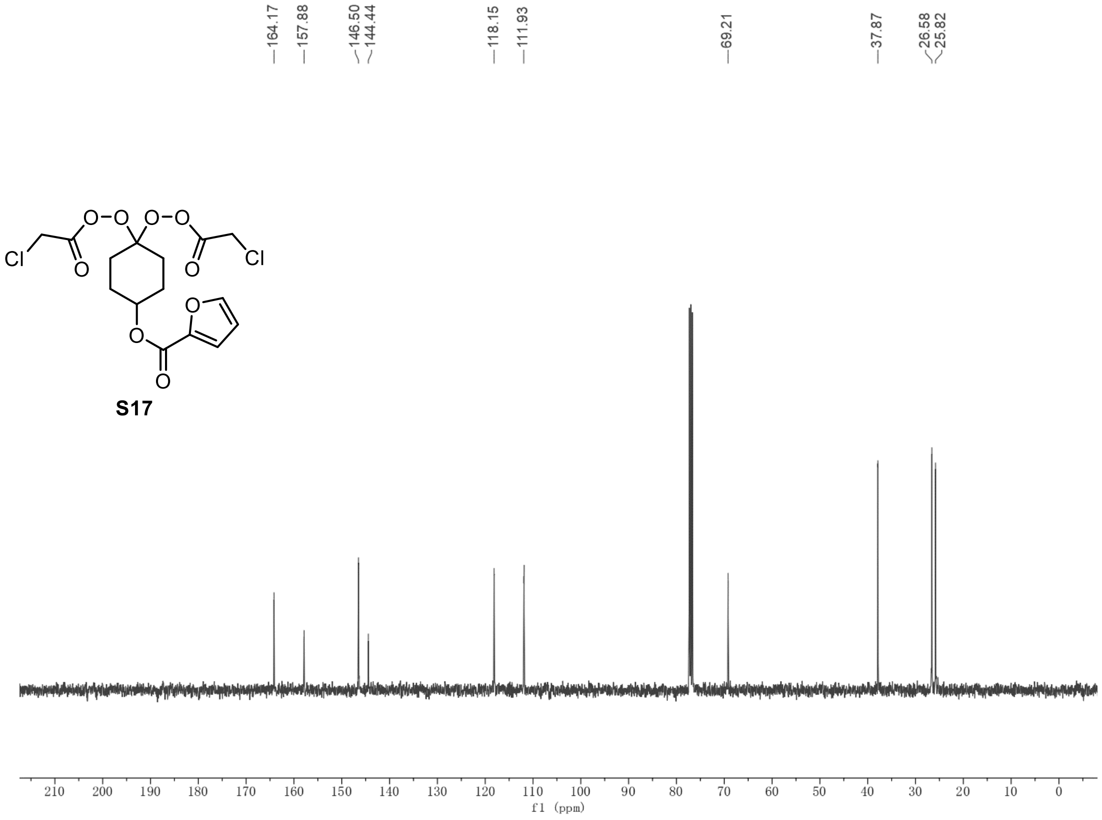

^{1}H NMR (400 MHz, $CDCl_3$) of **S18**

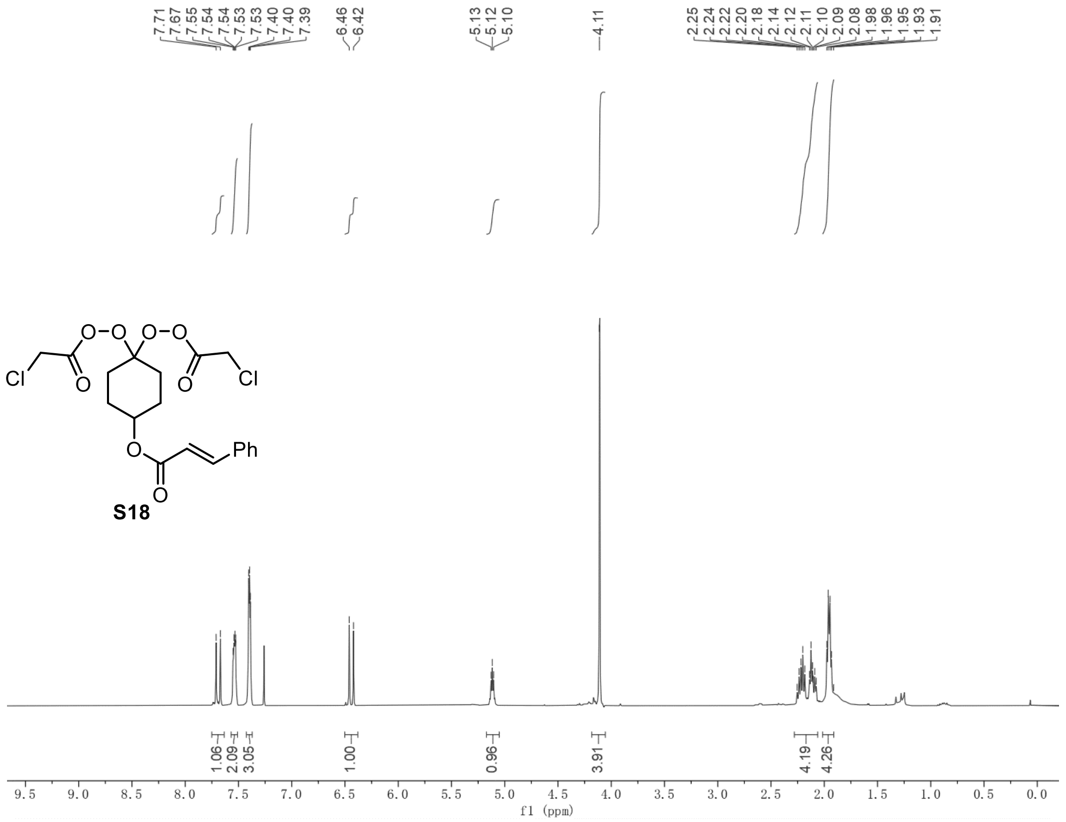

^{13}C NMR (100 MHz, $CDCl_3$) of **S18**

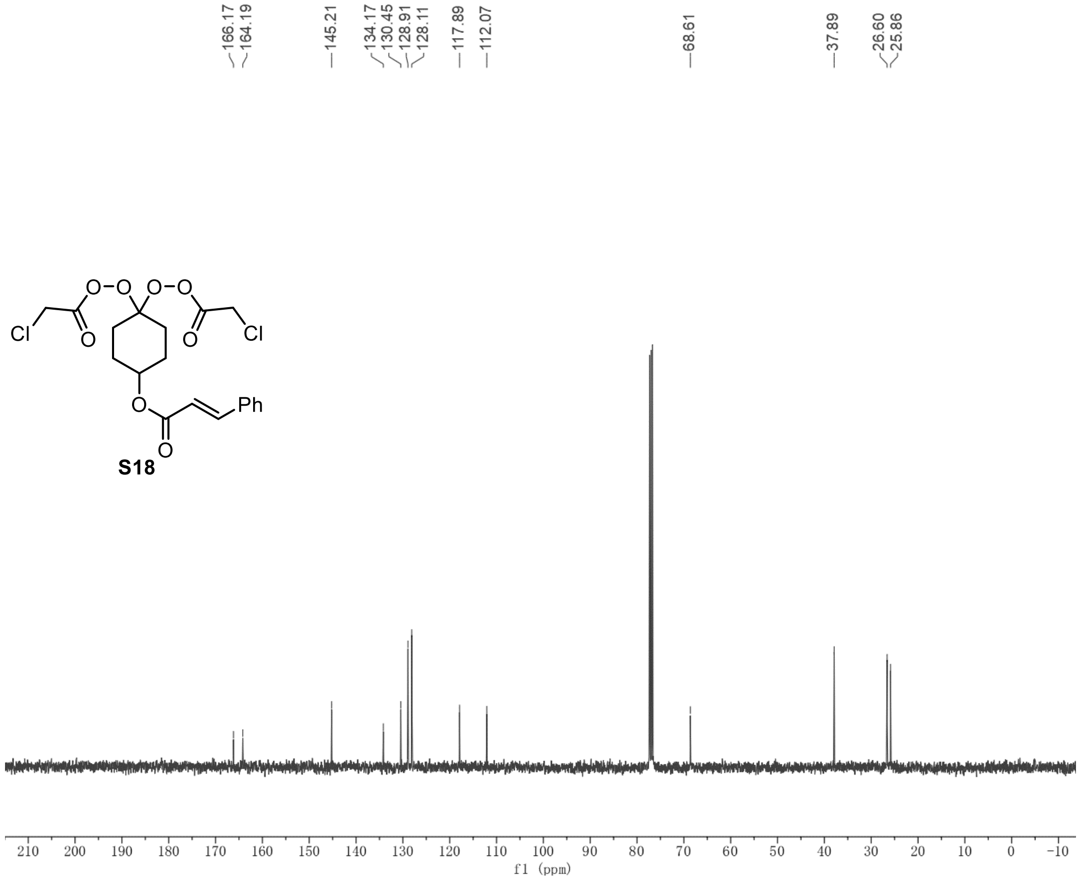

^{1}H NMR (400 MHz, $CDCl_3$) of **S19**

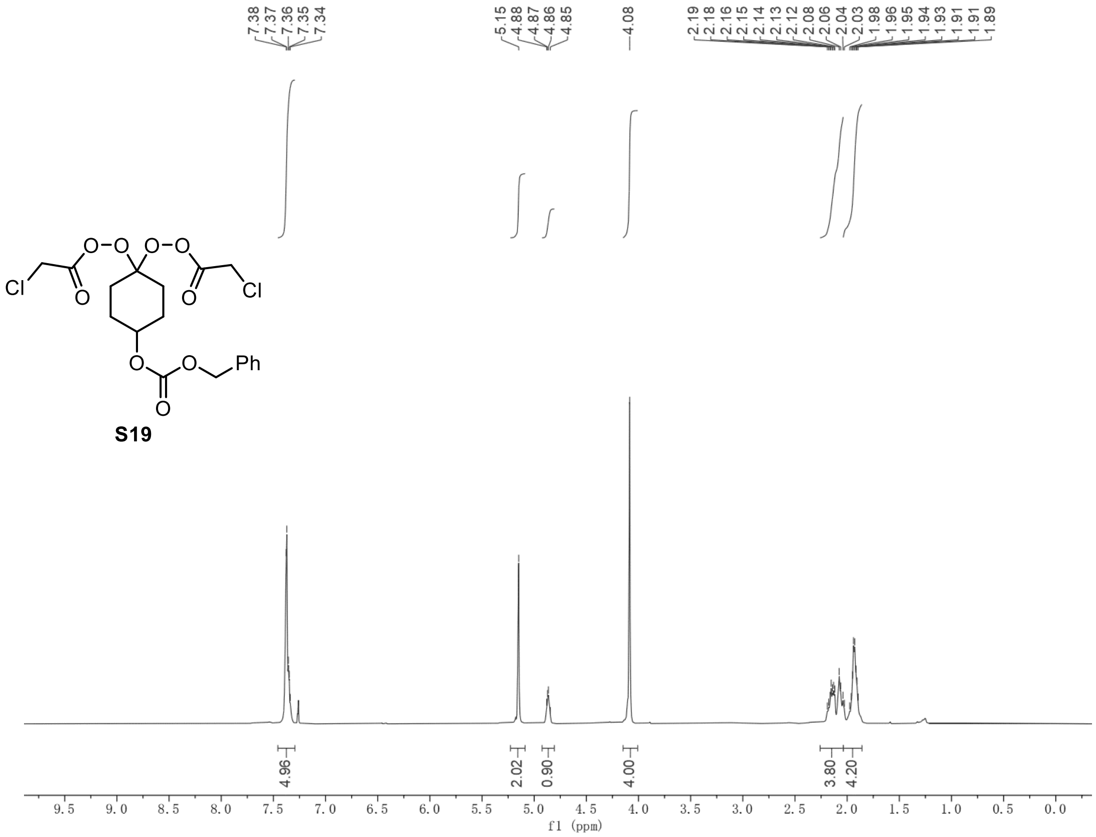

^{13}C NMR (100 MHz, $CDCl_3$) of **S19**

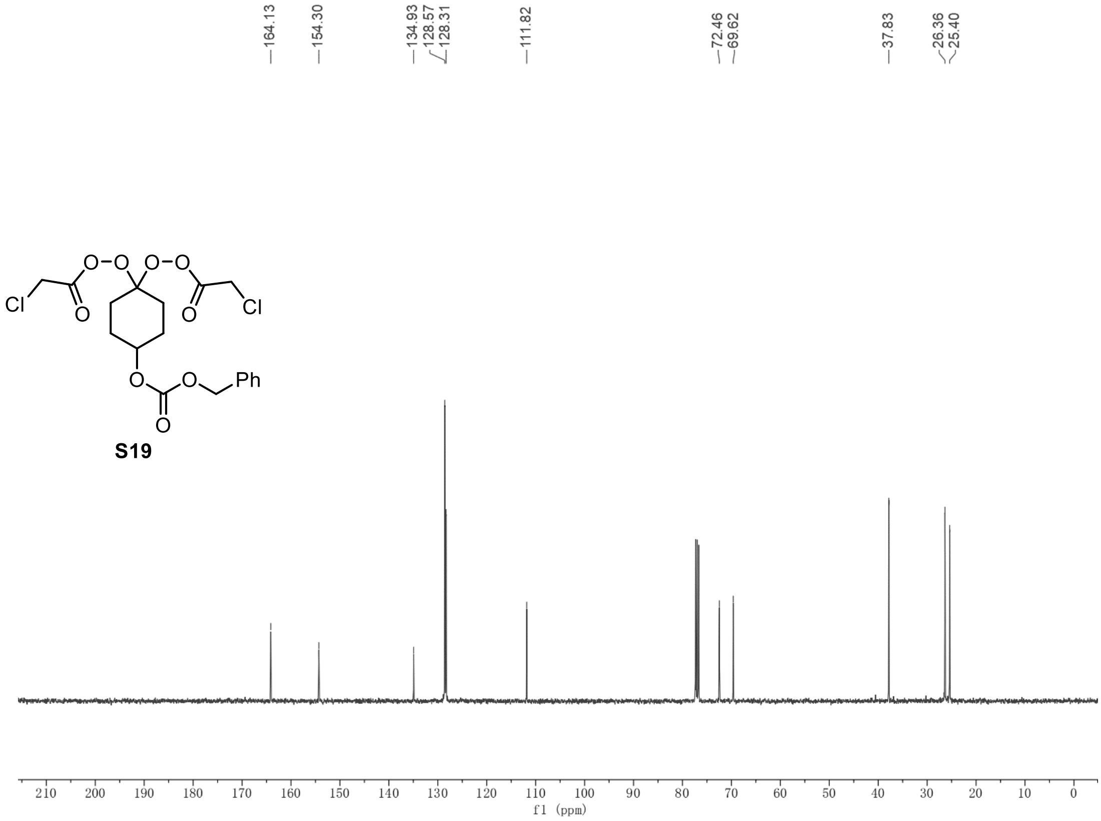

^{1}H NMR (400 MHz, $CDCl_3$) of **S20**

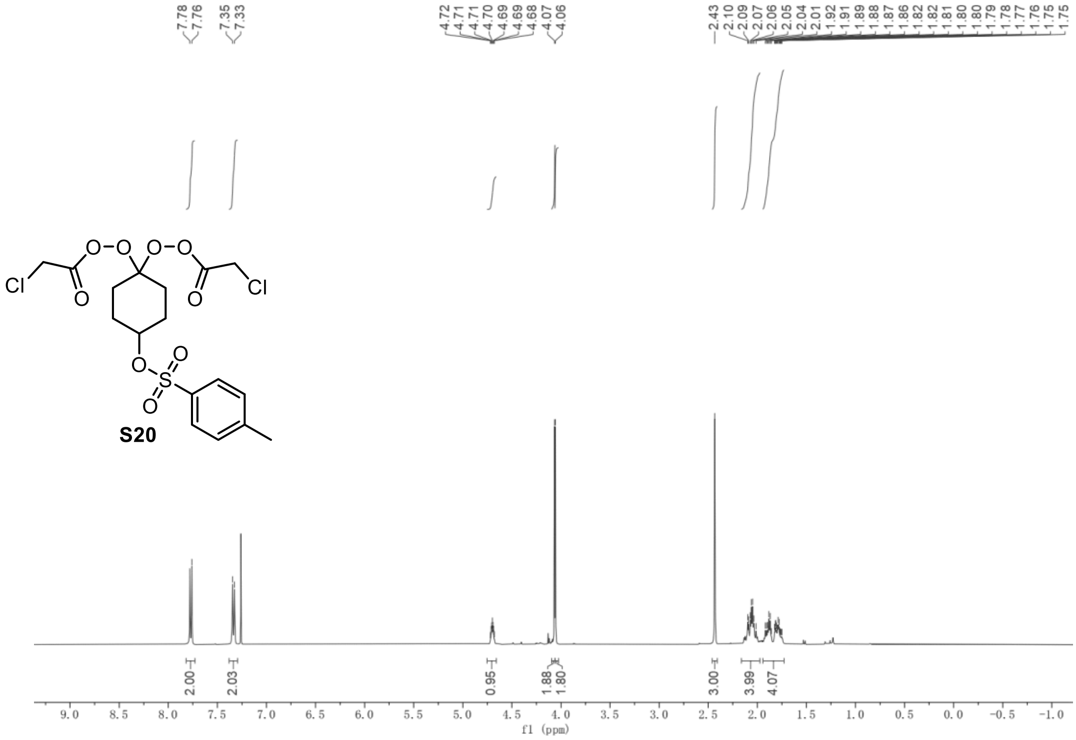

^{13}C NMR (100 MHz, $CDCl_3$) of **S20**

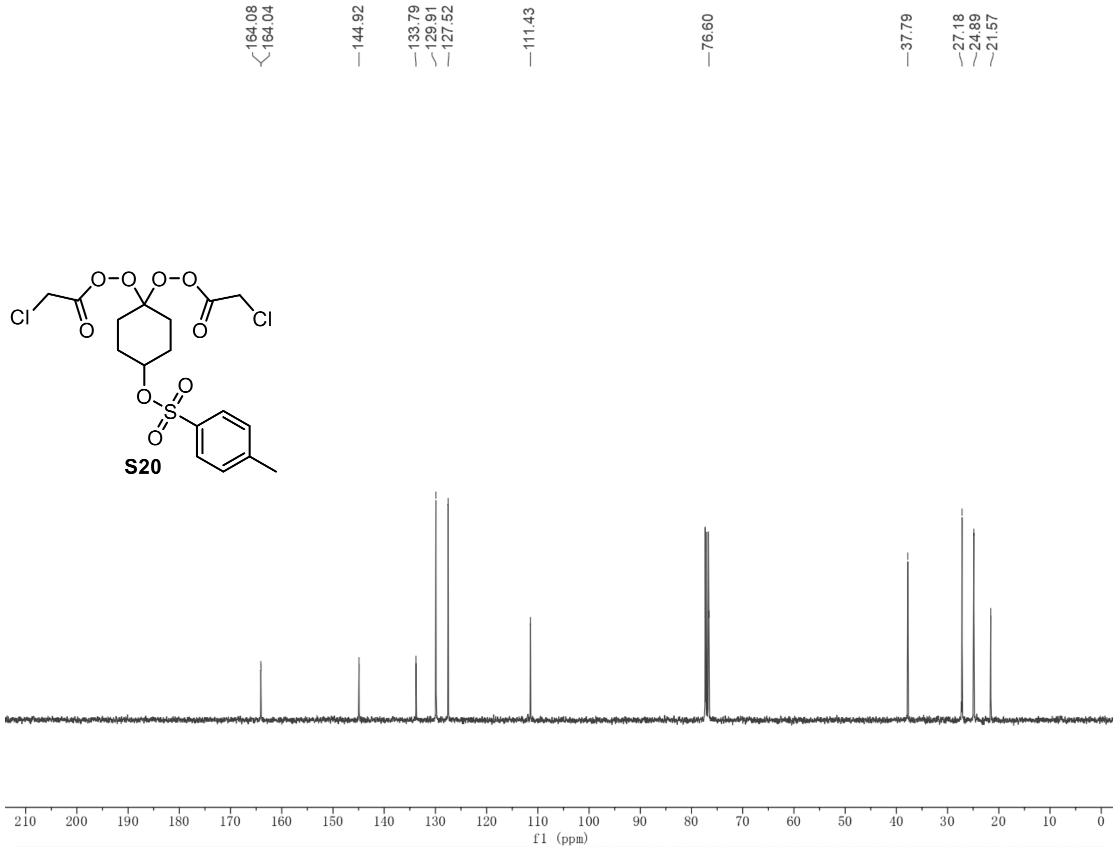

^{1}H NMR (400 MHz, $CDCl_3$) of **S21**

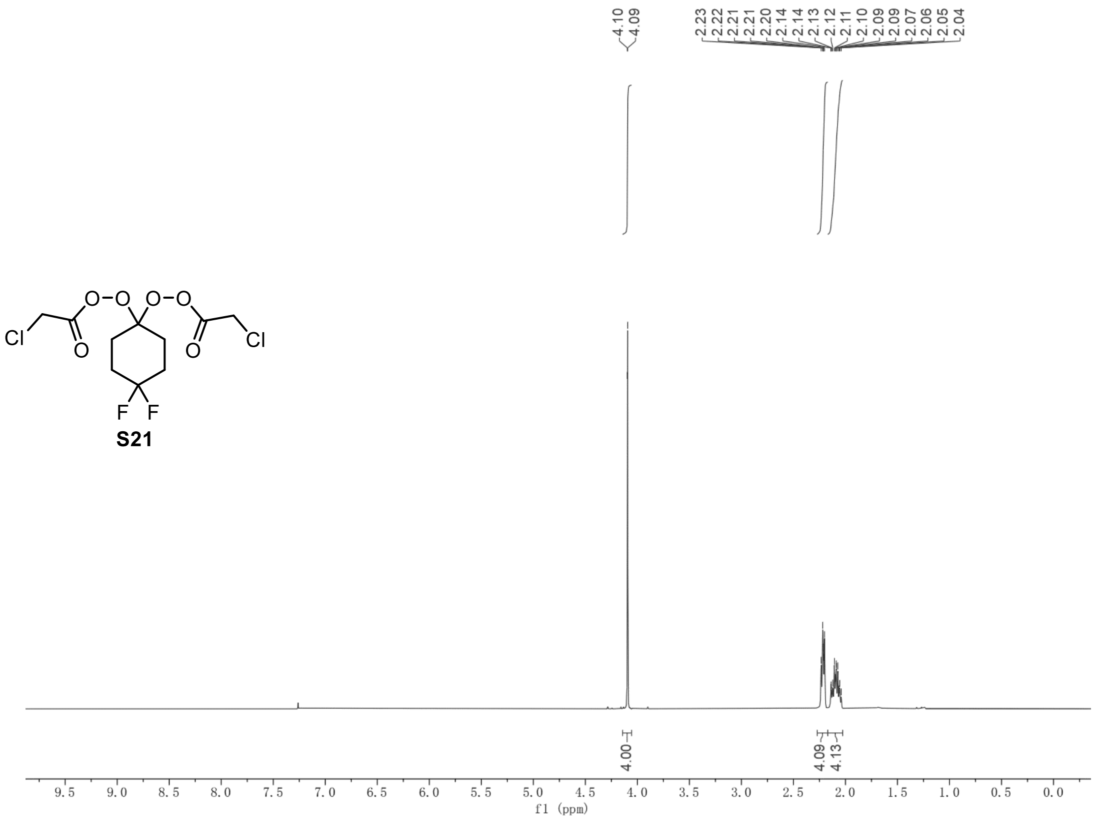

^{13}C NMR (100 MHz, $CDCl_3$) of **S21**

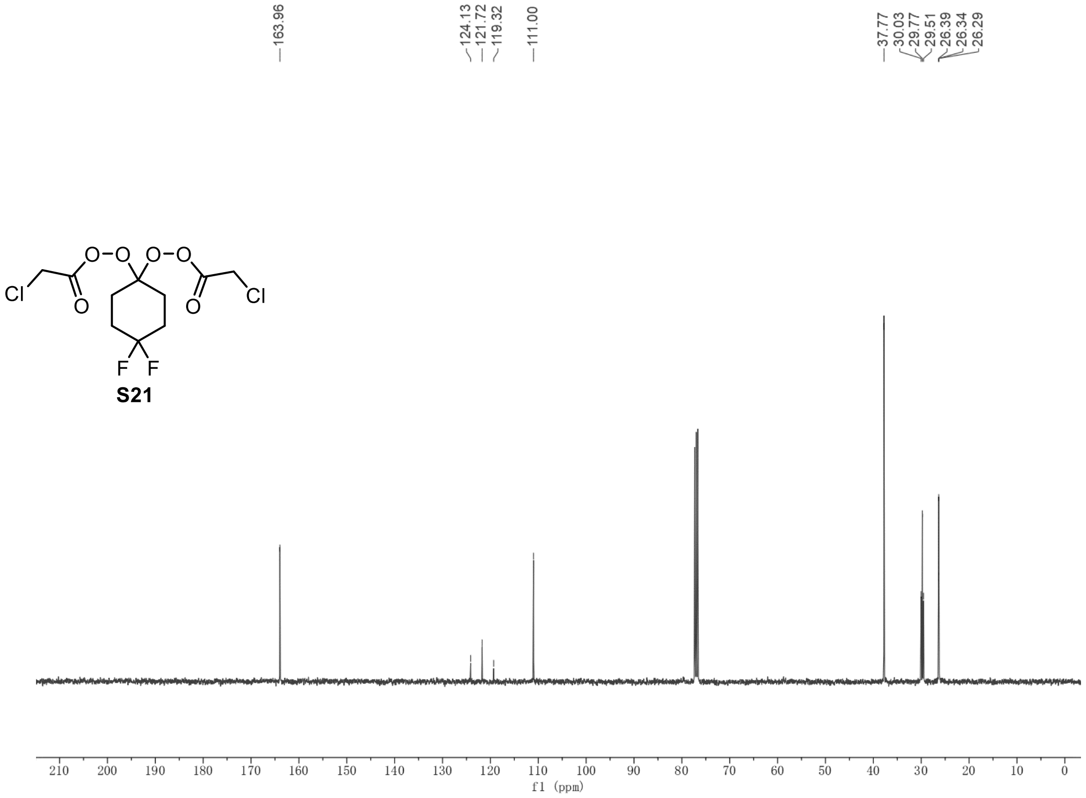

^{19}**F NMR** (376 MHz, $CDCl_3$) of **S21**

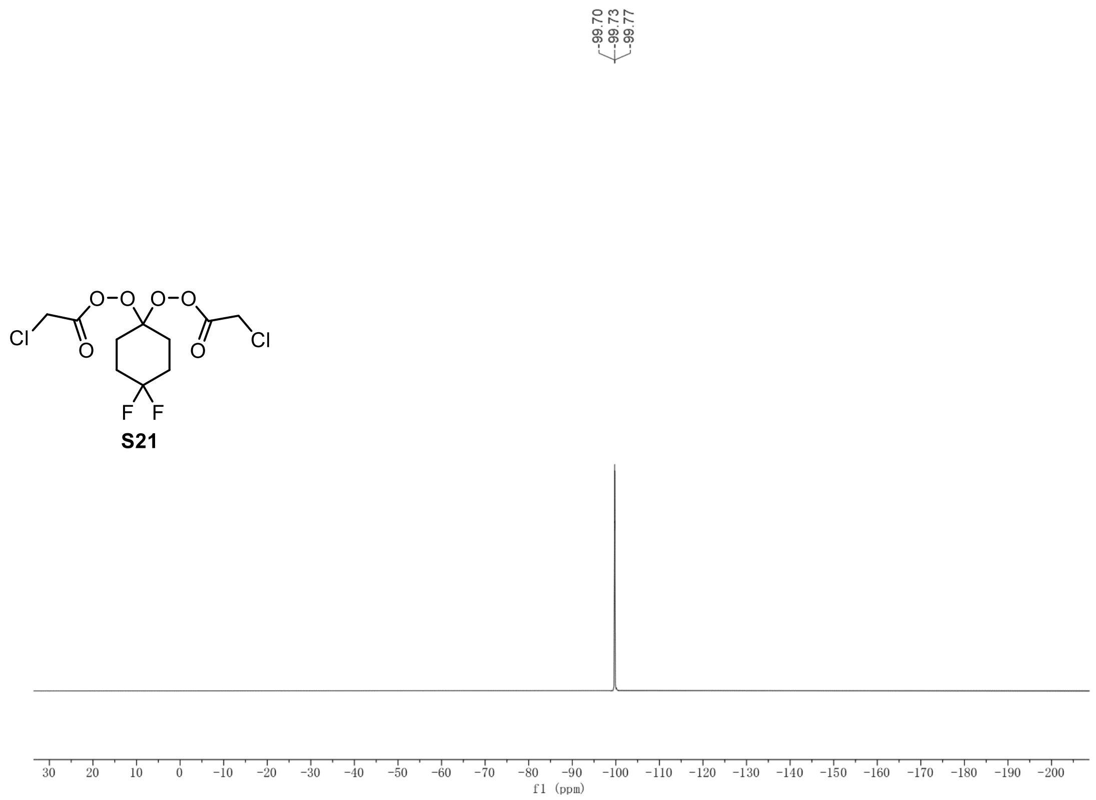

^{1}H NMR (400 MHz, $CDCl_3$) of **S22**

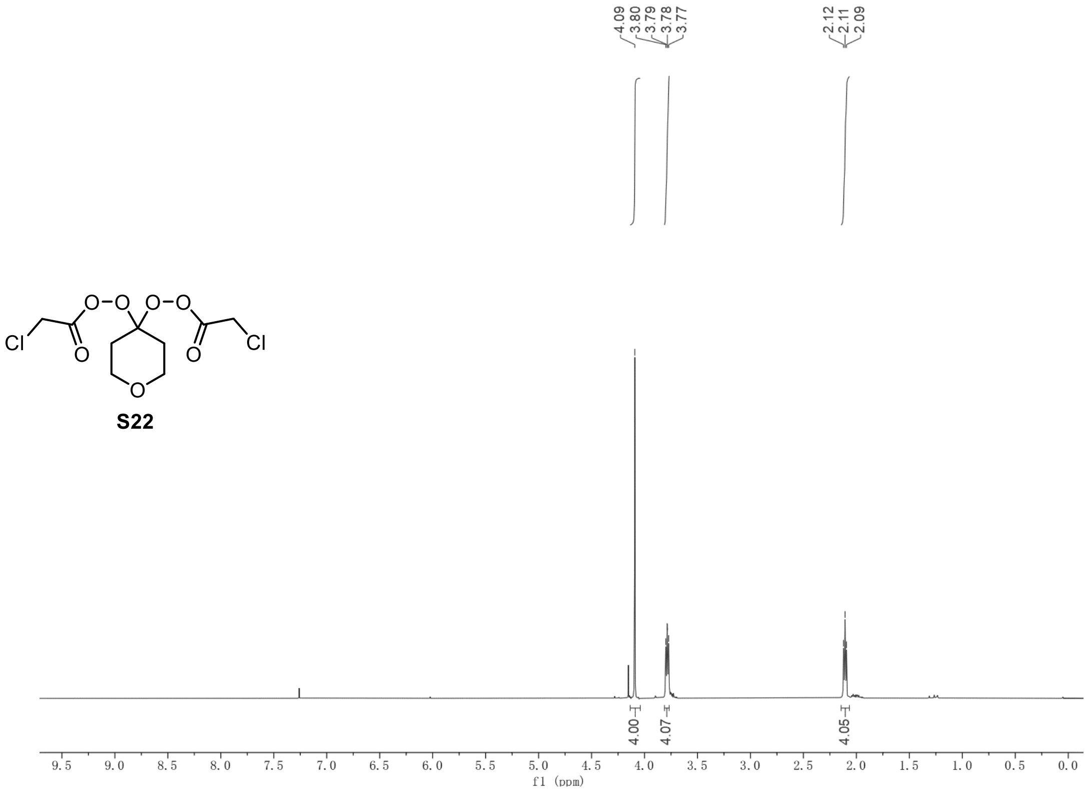

S22

^{13}C NMR (100 MHz, $CDCl_3$) of **S22**

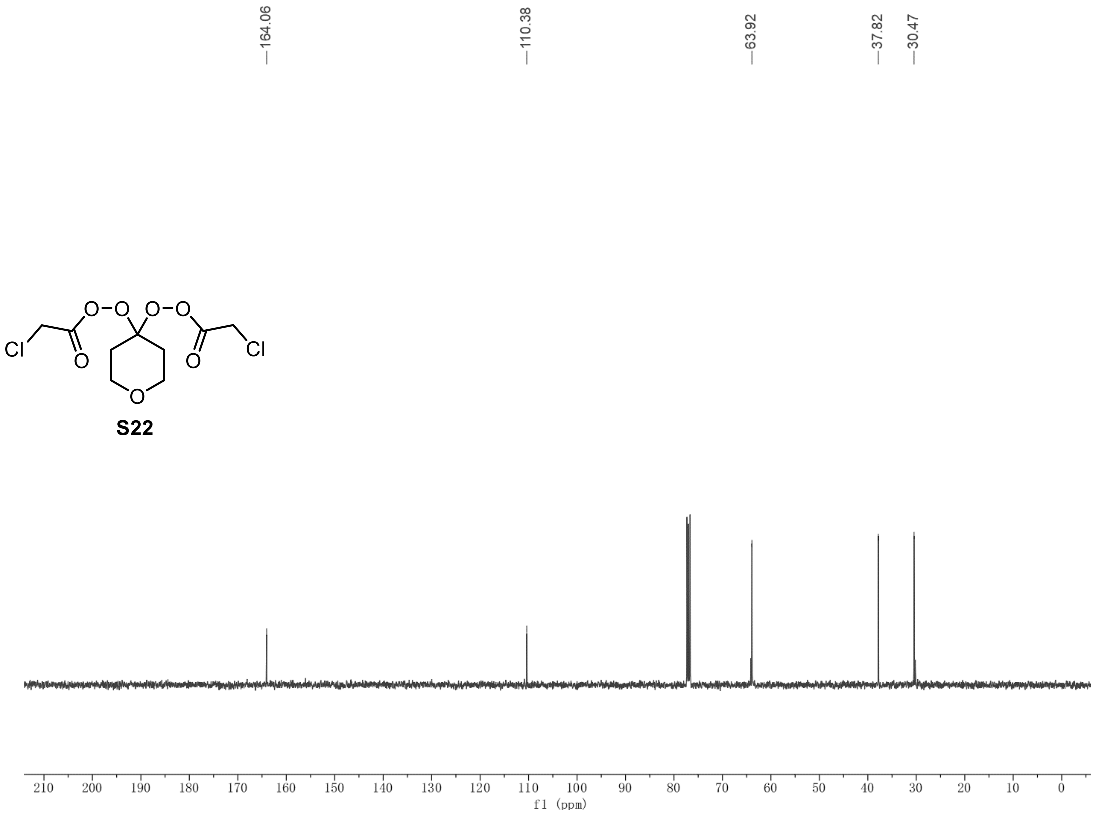

S22

^{1}H NMR (400 MHz, $CDCl_3$) of **S23**

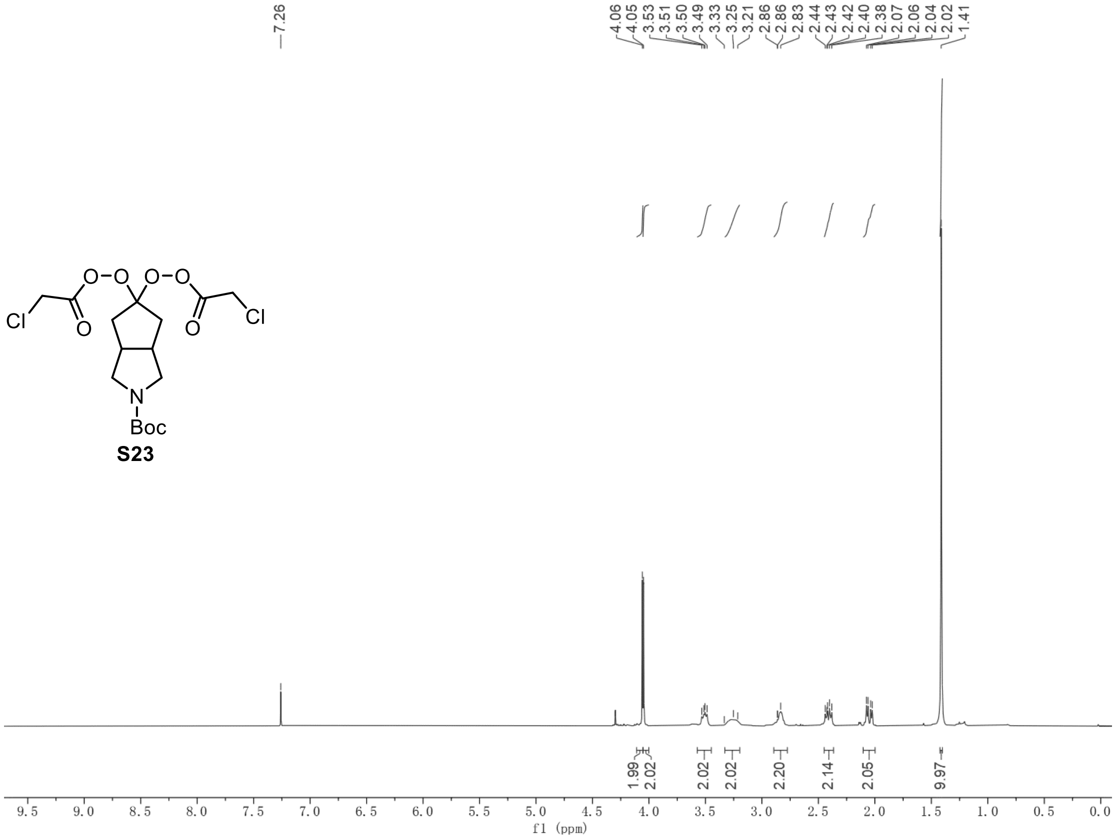

^{13}C NMR (100 MHz, $CDCl_3$) of **S23**

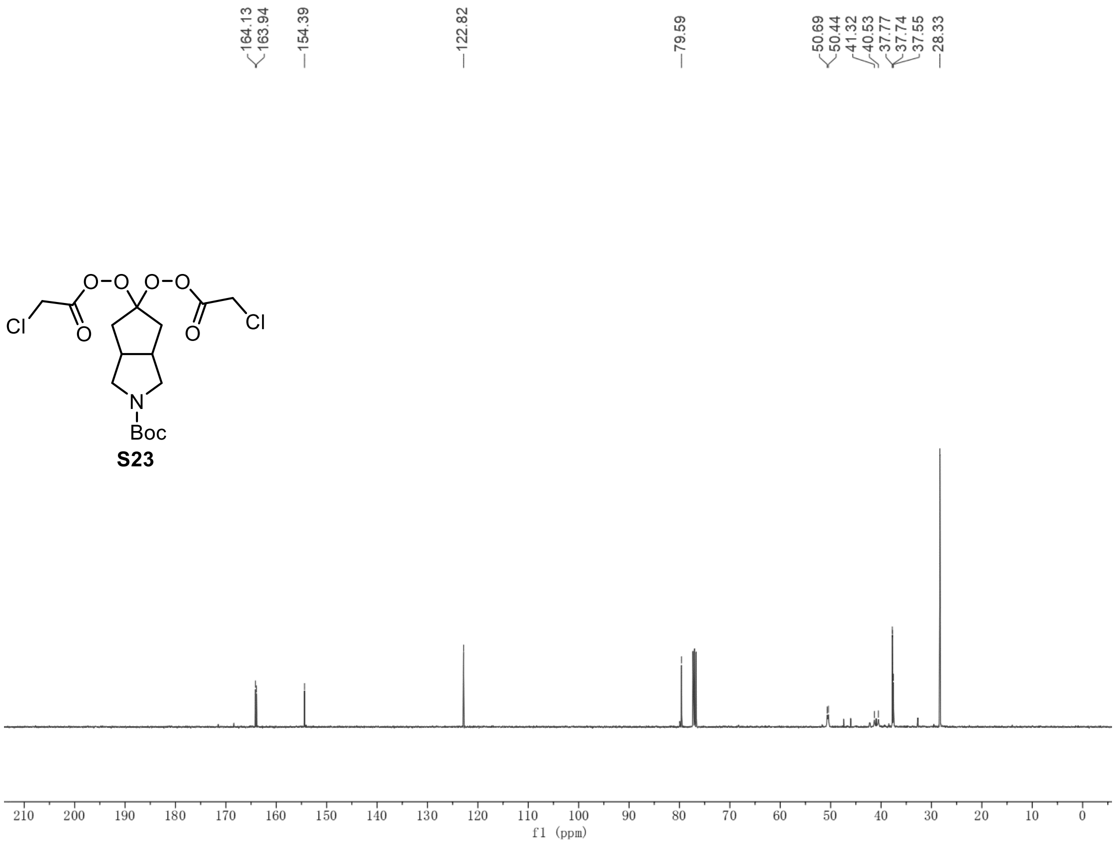

^{1}H NMR (400 MHz, $CDCl_3$) of **S24**

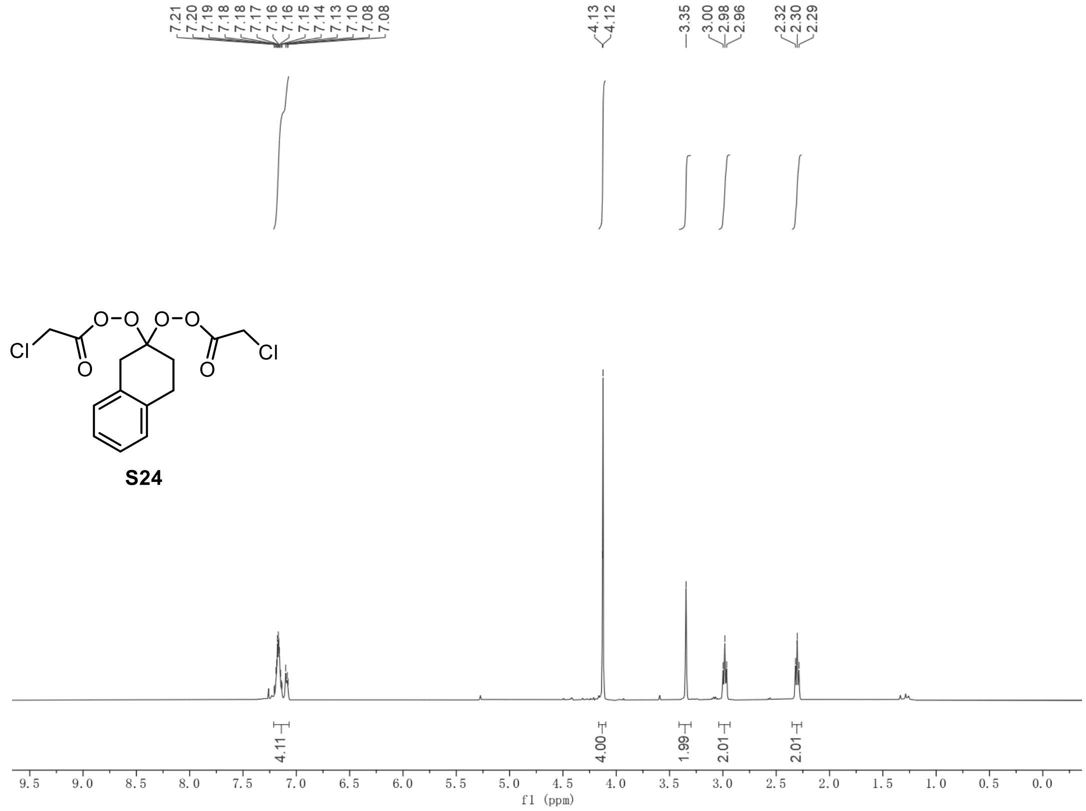

^{13}C NMR (100 MHz, $CDCl_3$) of **S24**

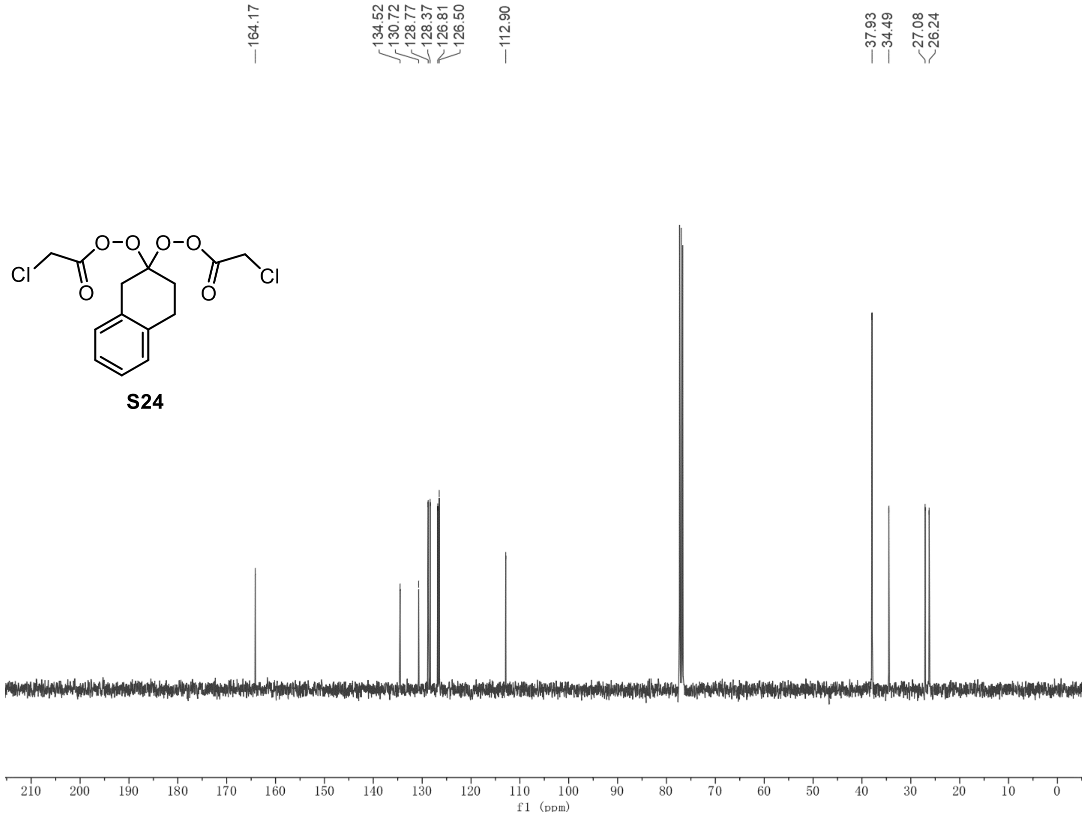

^{1}**H NMR** (400 MHz, $CDCl_3$) of **S25**

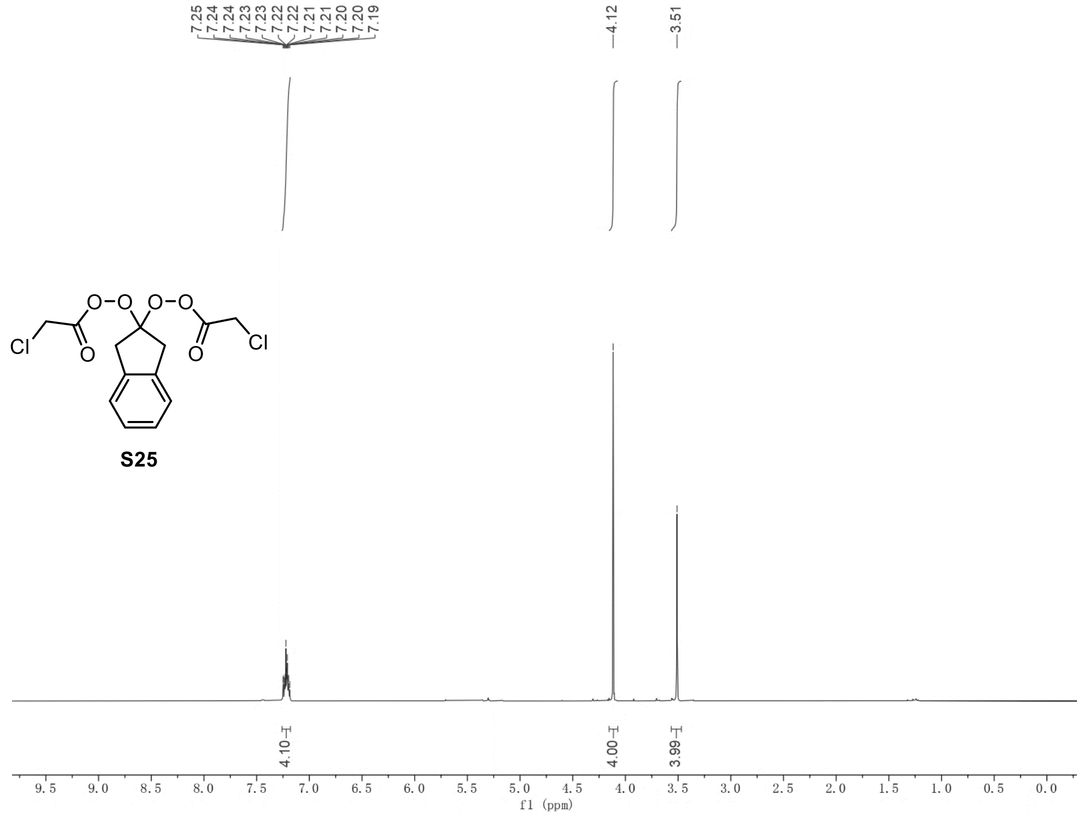

^{13}**C NMR** (100 MHz, $CDCl_3$) of **S25**

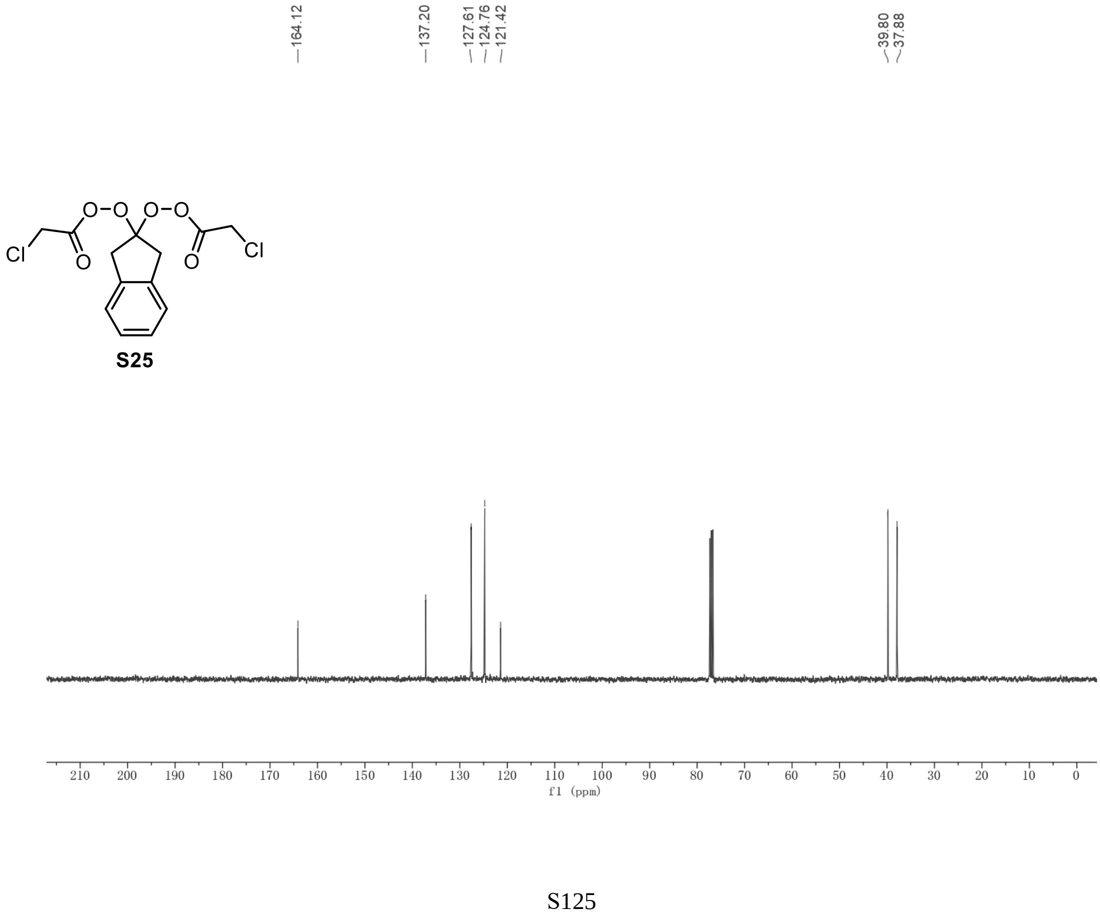

^{1}H NMR (400 MHz, $CDCl_3$) of **S26**

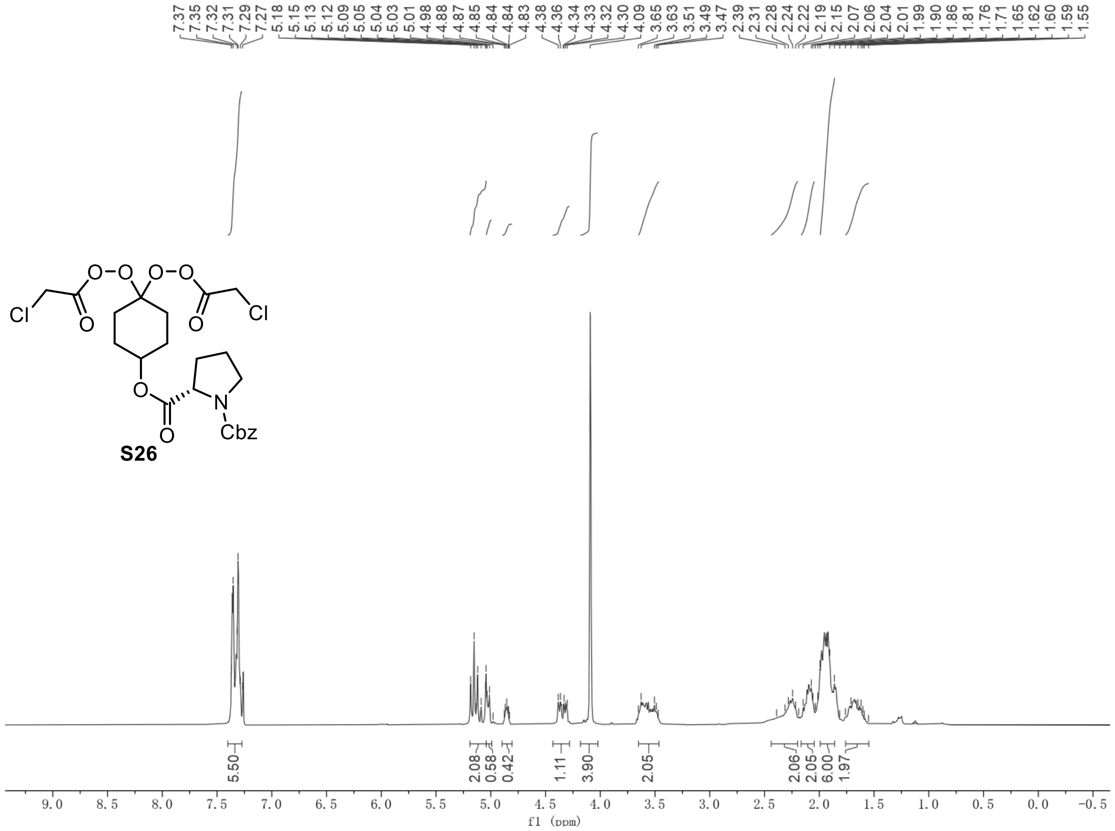

^{13}C NMR (100 MHz, $CDCl_3$) of **S26**

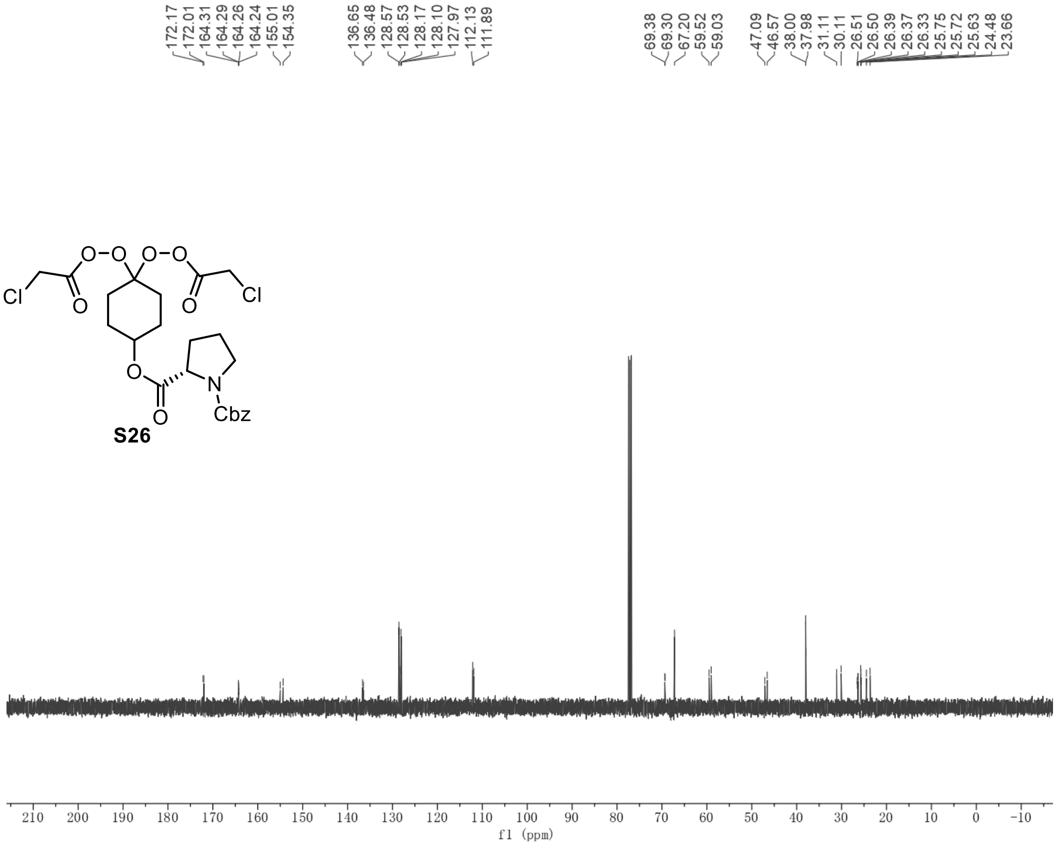

^{1}H NMR (400 MHz, $CDCl_3$) of **S27**

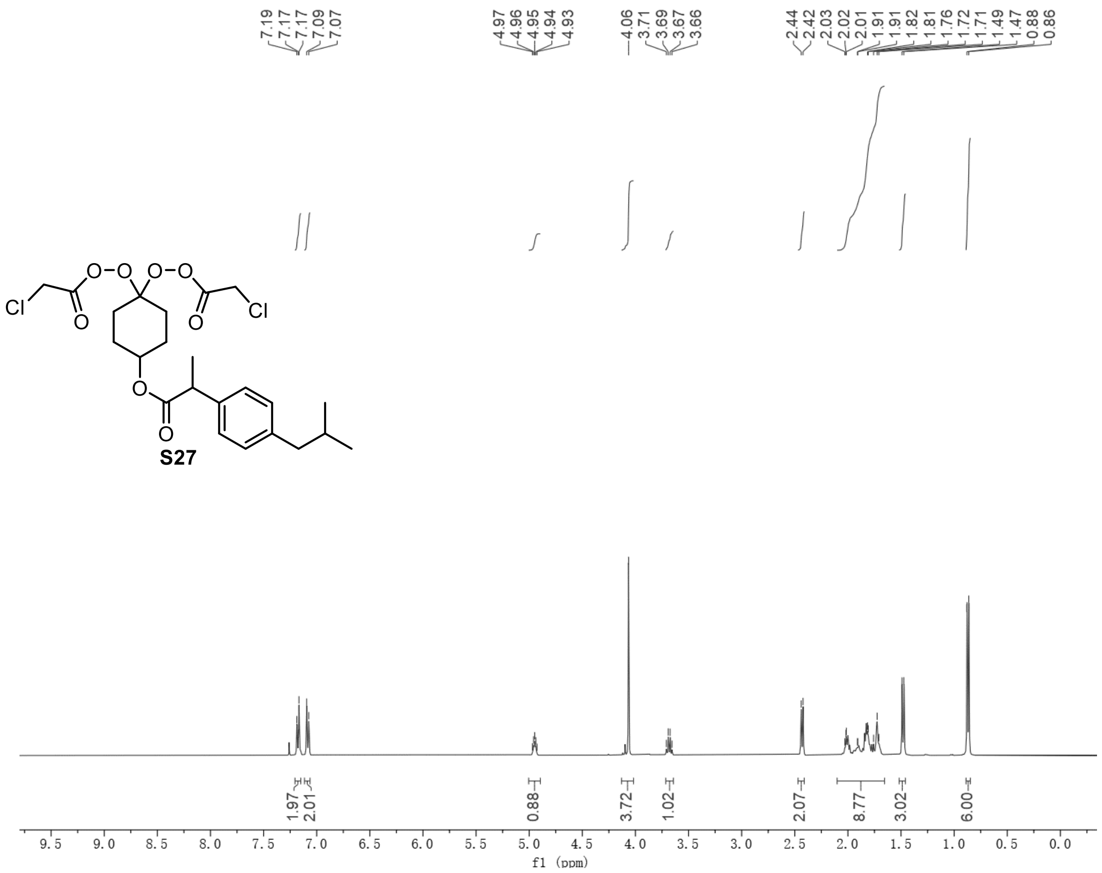

^{13}C NMR (100 MHz, $CDCl_3$) of **S27**

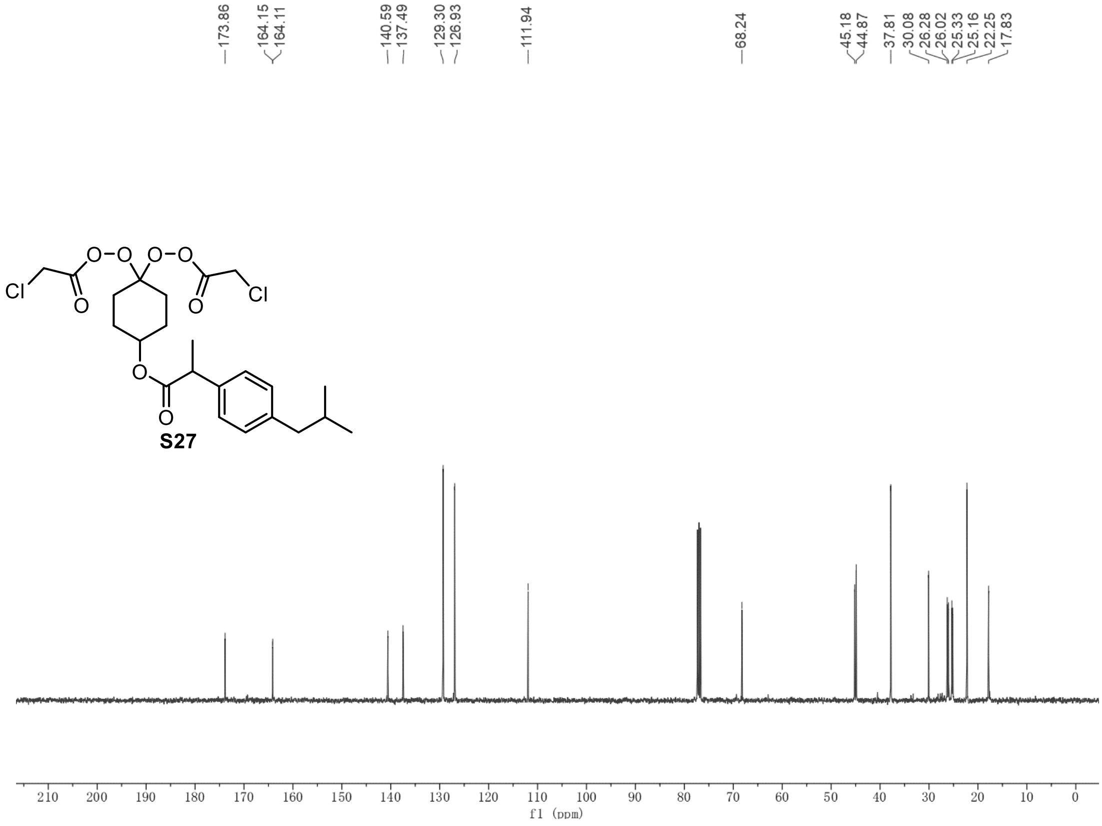

^{1}H NMR (400 MHz, $CDCl_3$) of **S28**

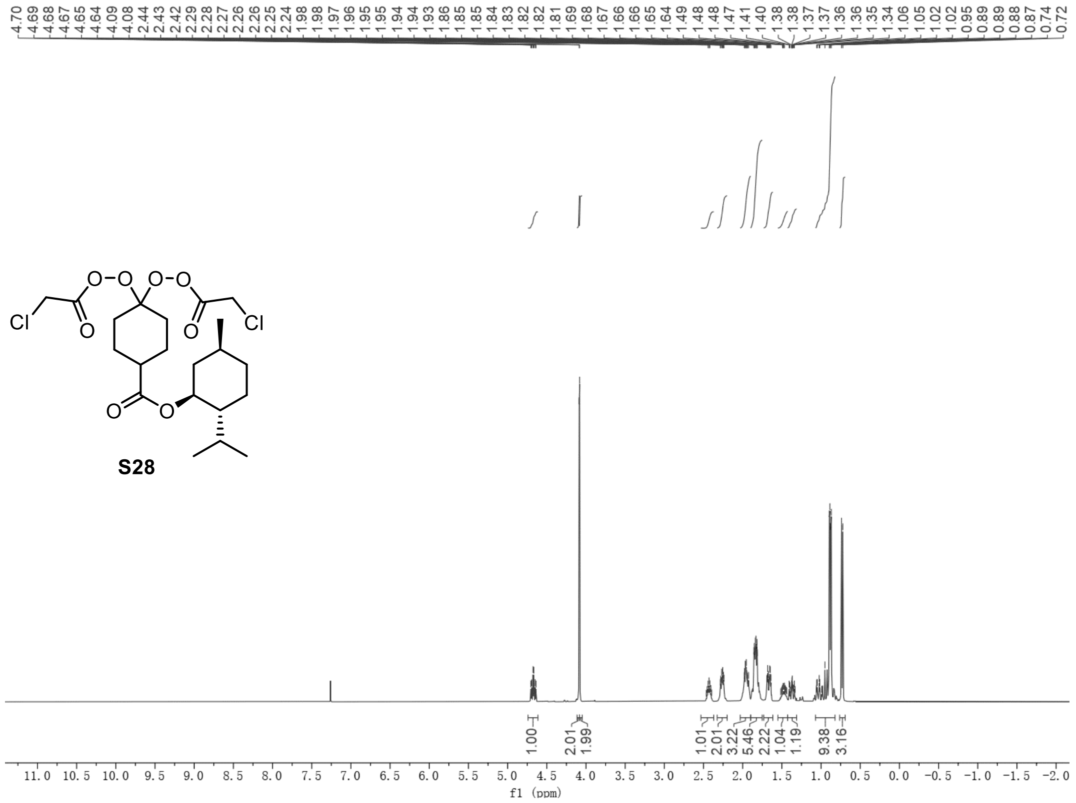

^{13}C NMR (100 MHz, $CDCl_3$) of **S28**

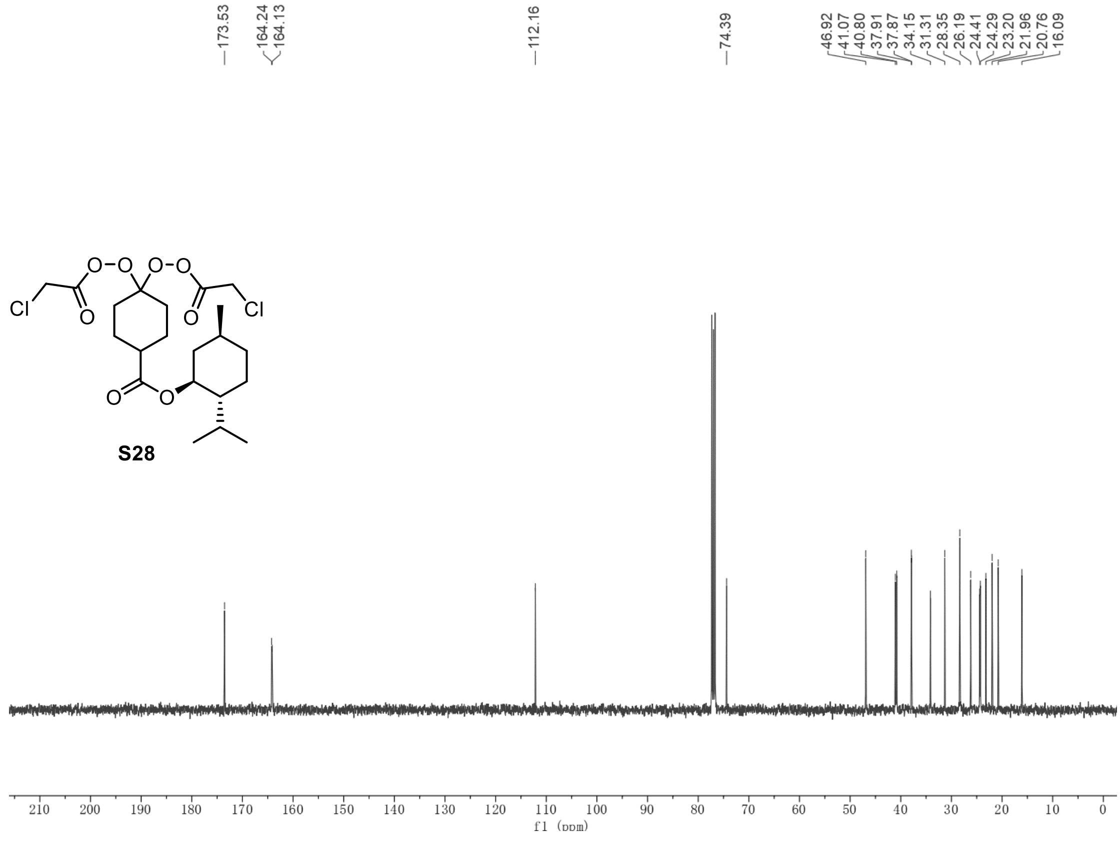

¹H NMR (400 MHz, $CDCl_3$) of **S29**

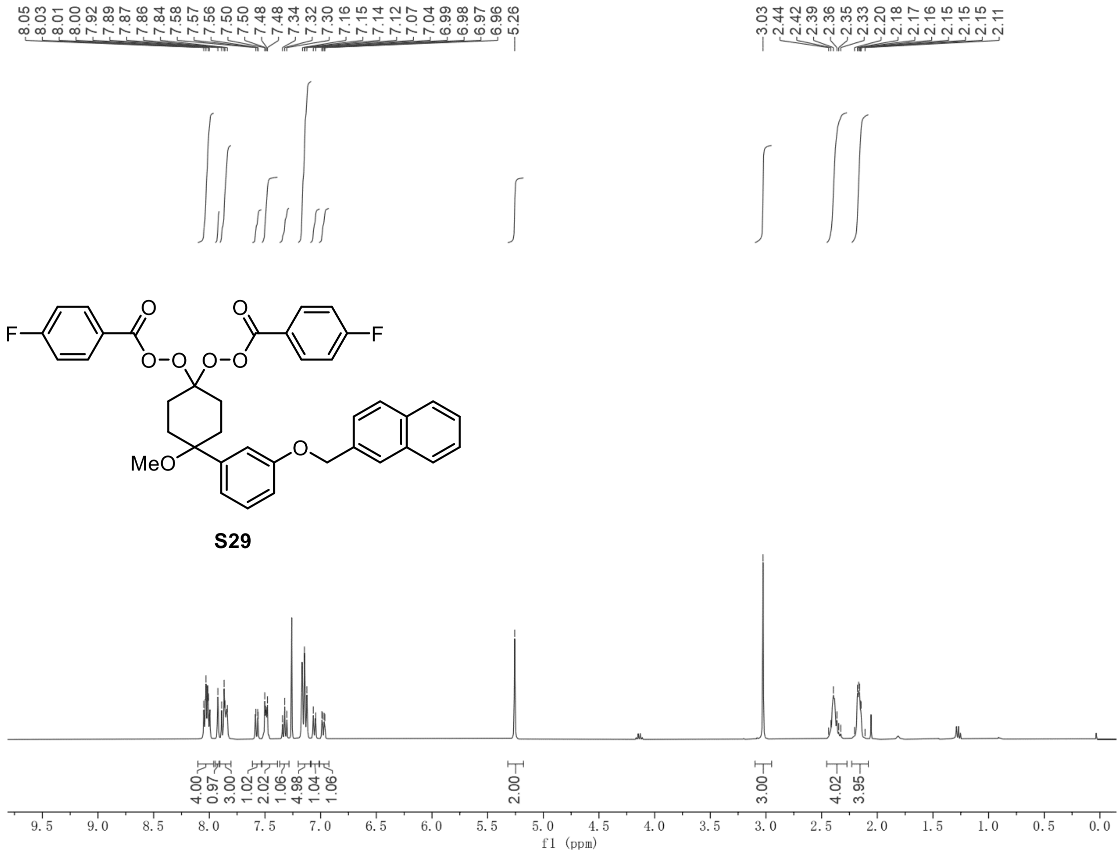

¹⁹F NMR (376 MHz, $CDCl_3$) of **S29**

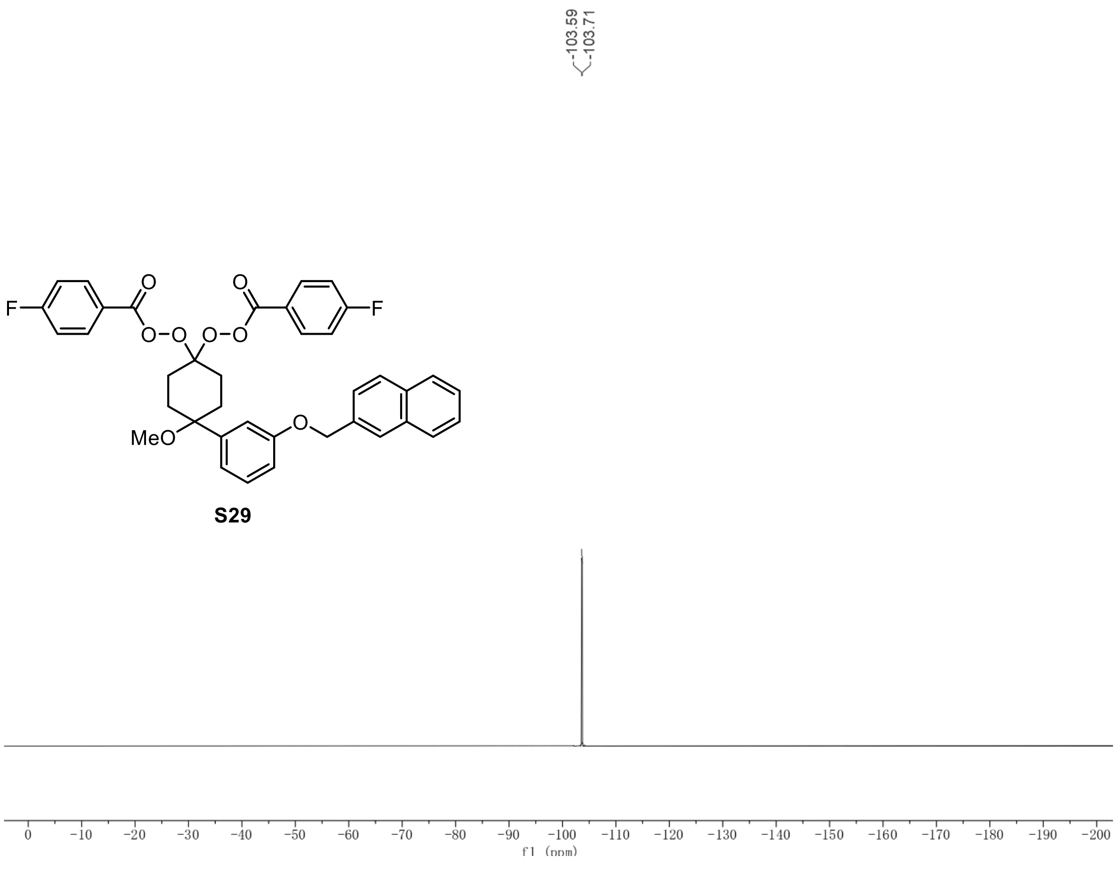

^{13}C NMR (100 MHz, $CDCl_3$) of **S29**

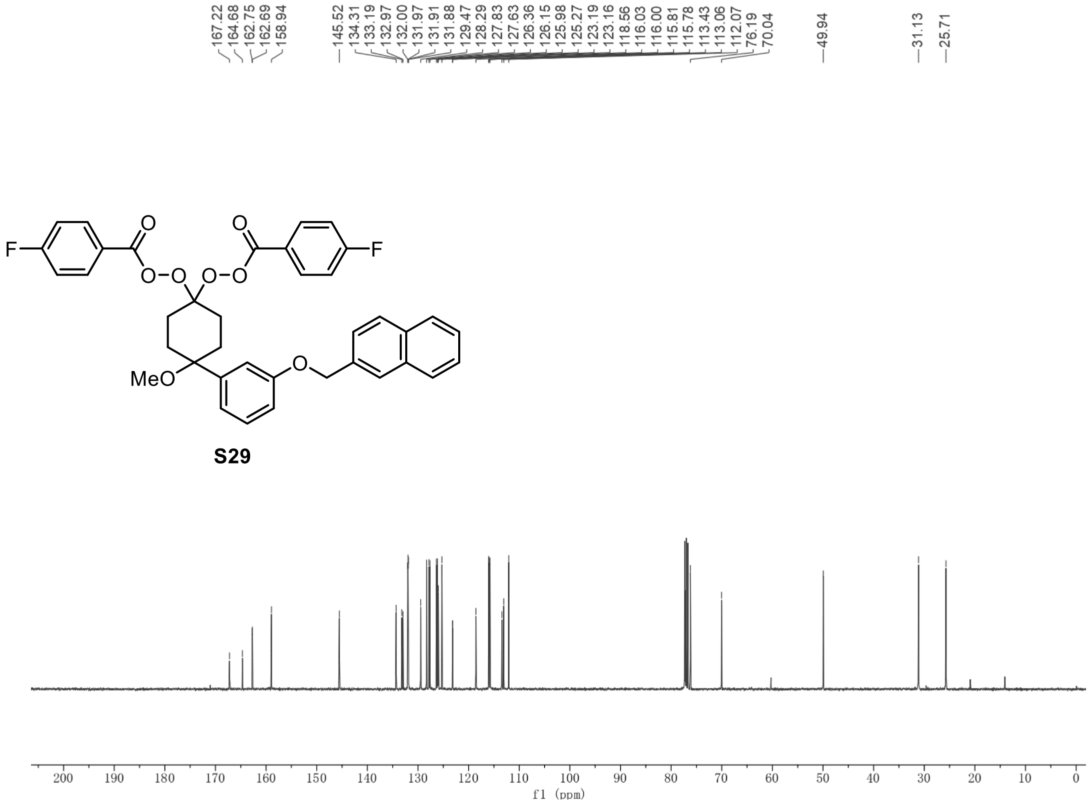

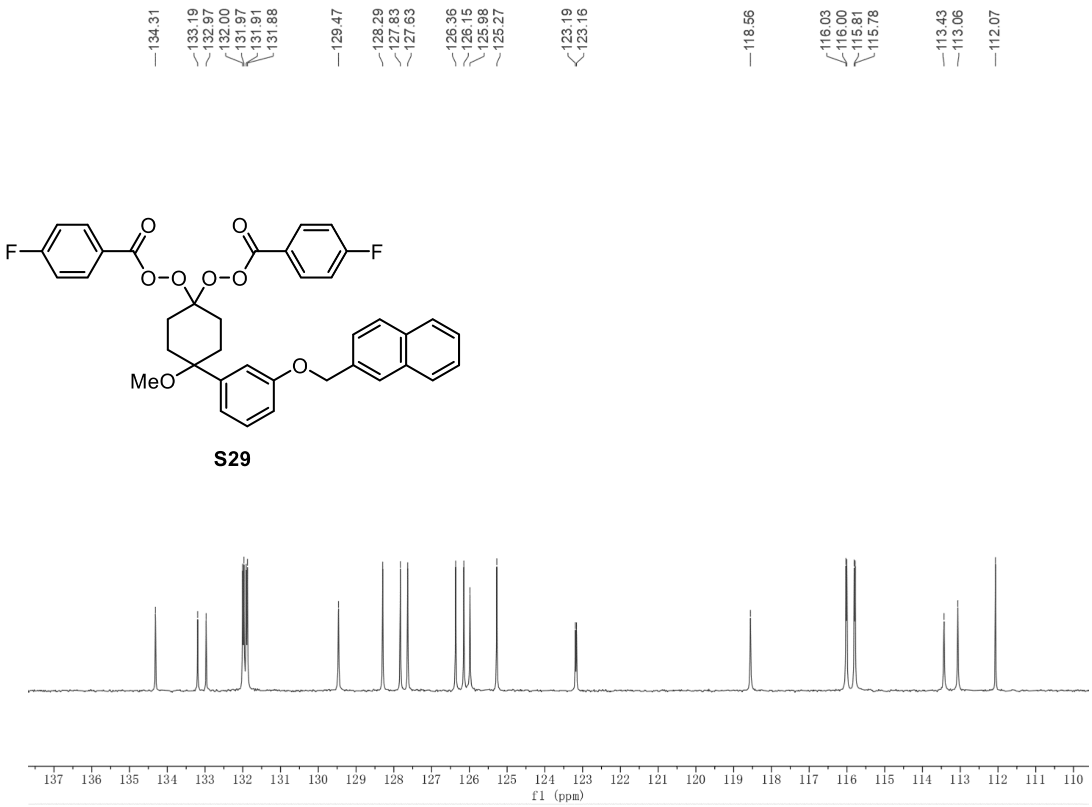

^{1}H NMR (400 MHz, $CDCl_3$) of **S30**

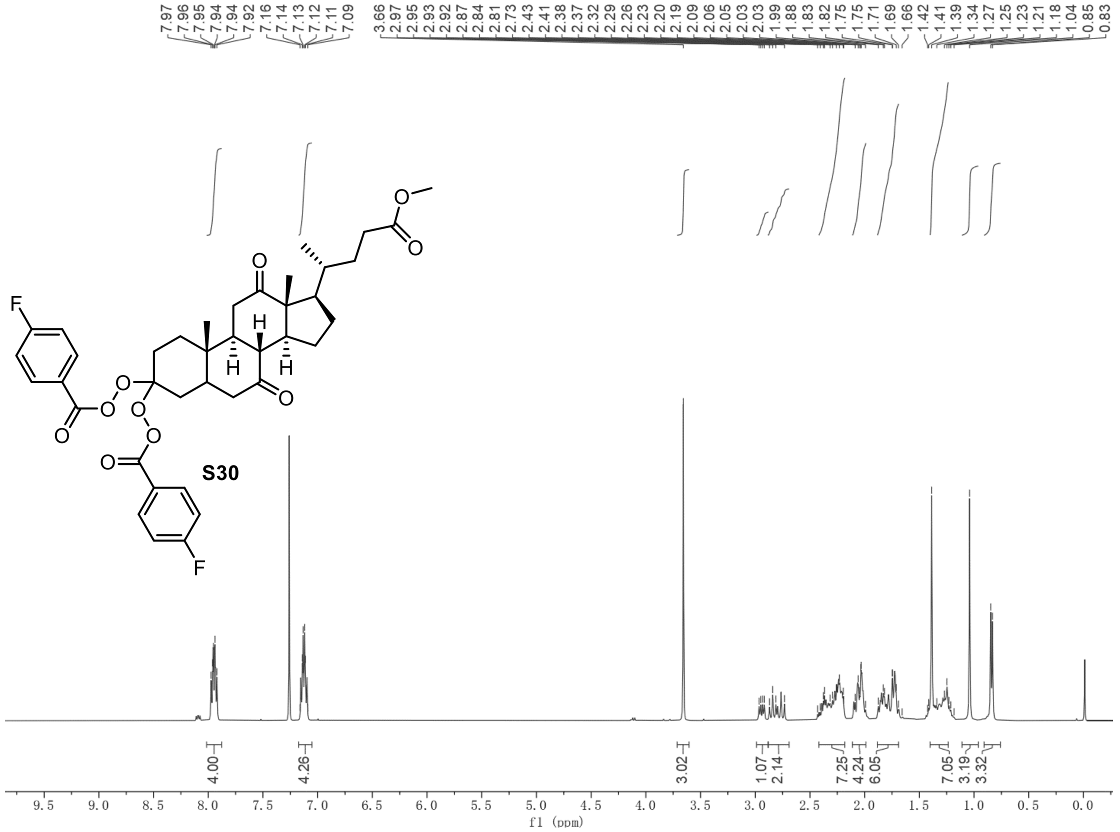

^{19}F NMR (376 MHz, $CDCl_3$) of **S30**

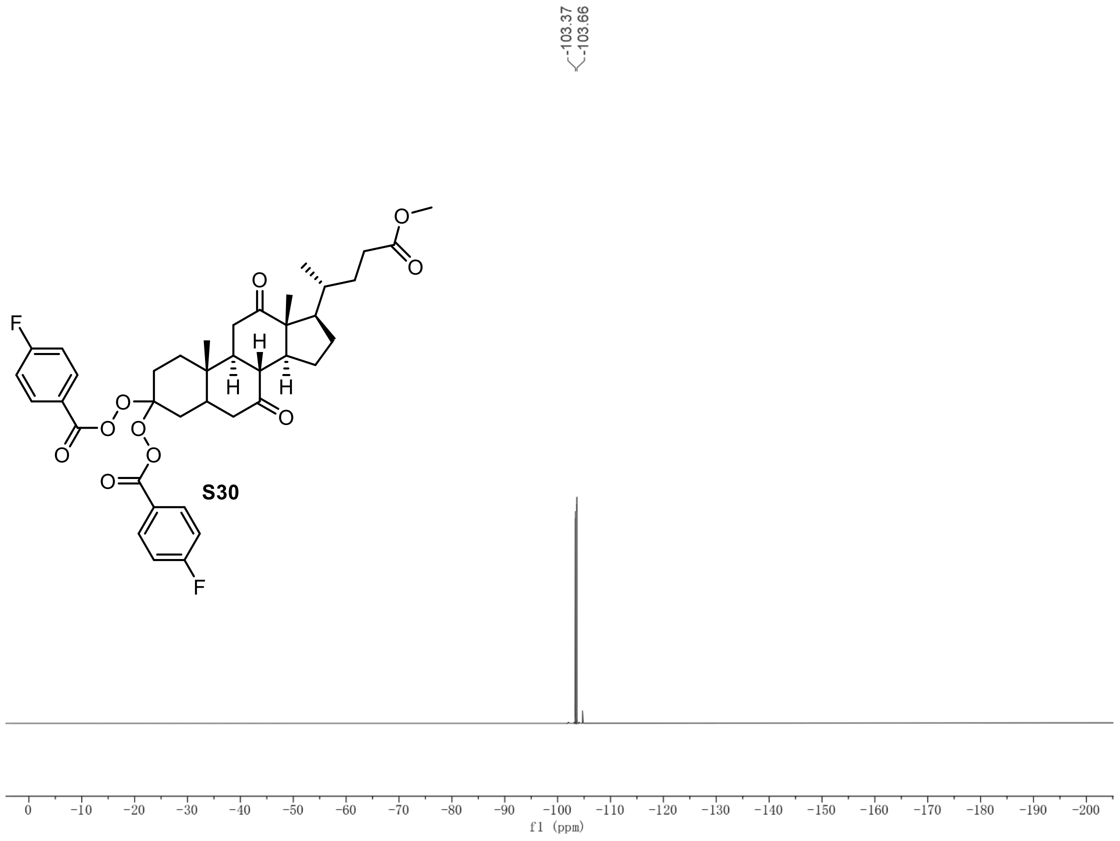

^{13}C NMR (100 MHz, $CDCl_3$) of **S30**

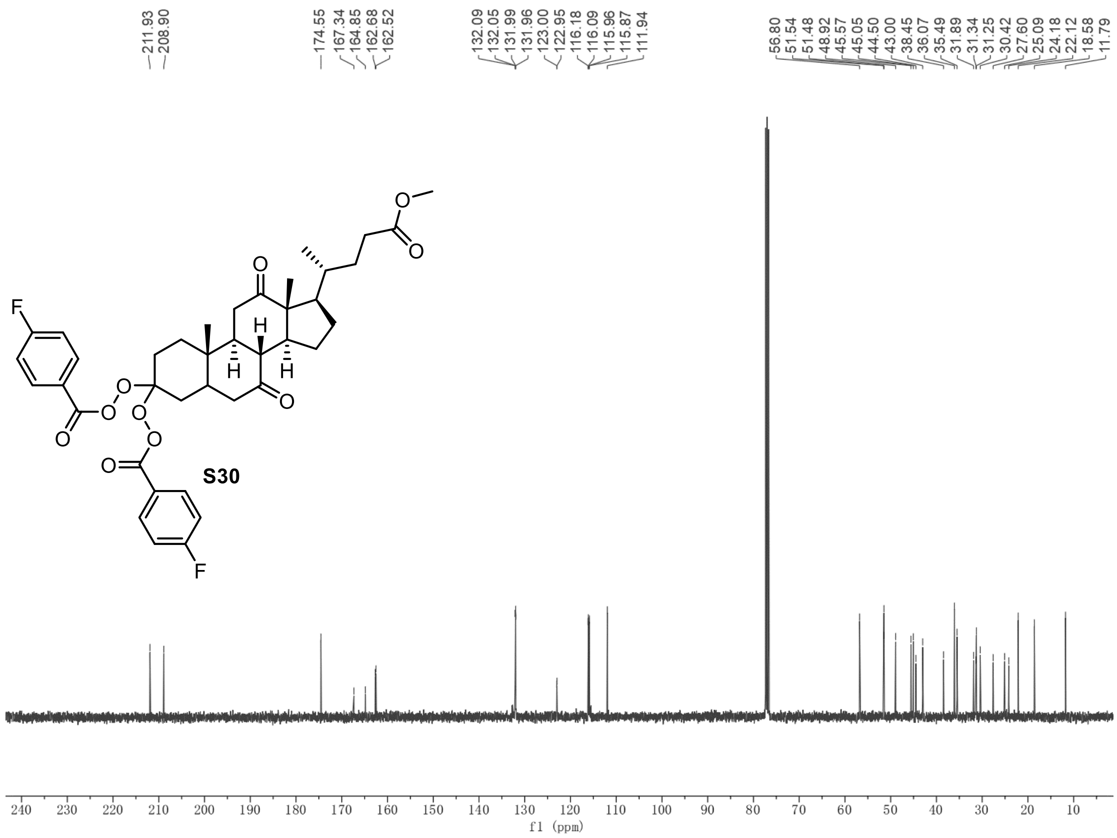

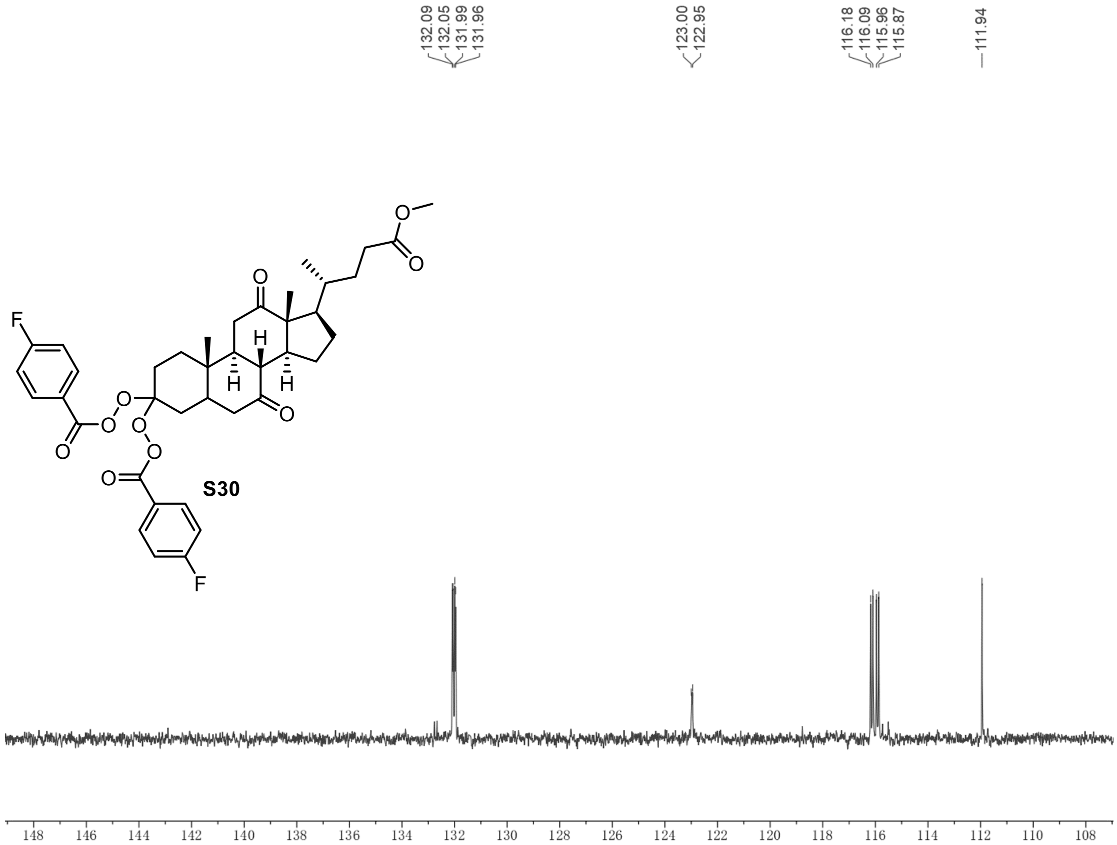

^{1}H NMR (400 MHz, $CDCl_3$) of **S31**

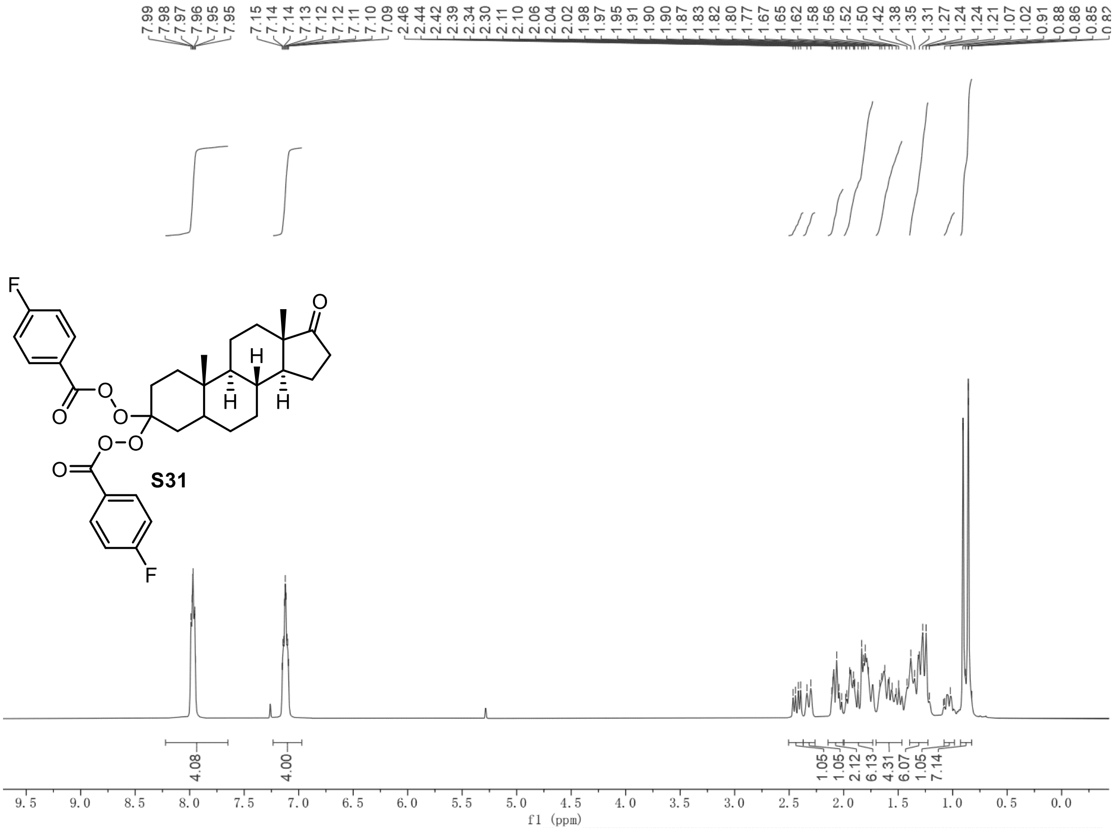

^{19}F NMR (376 MHz, $CDCl_3$) of **S31**

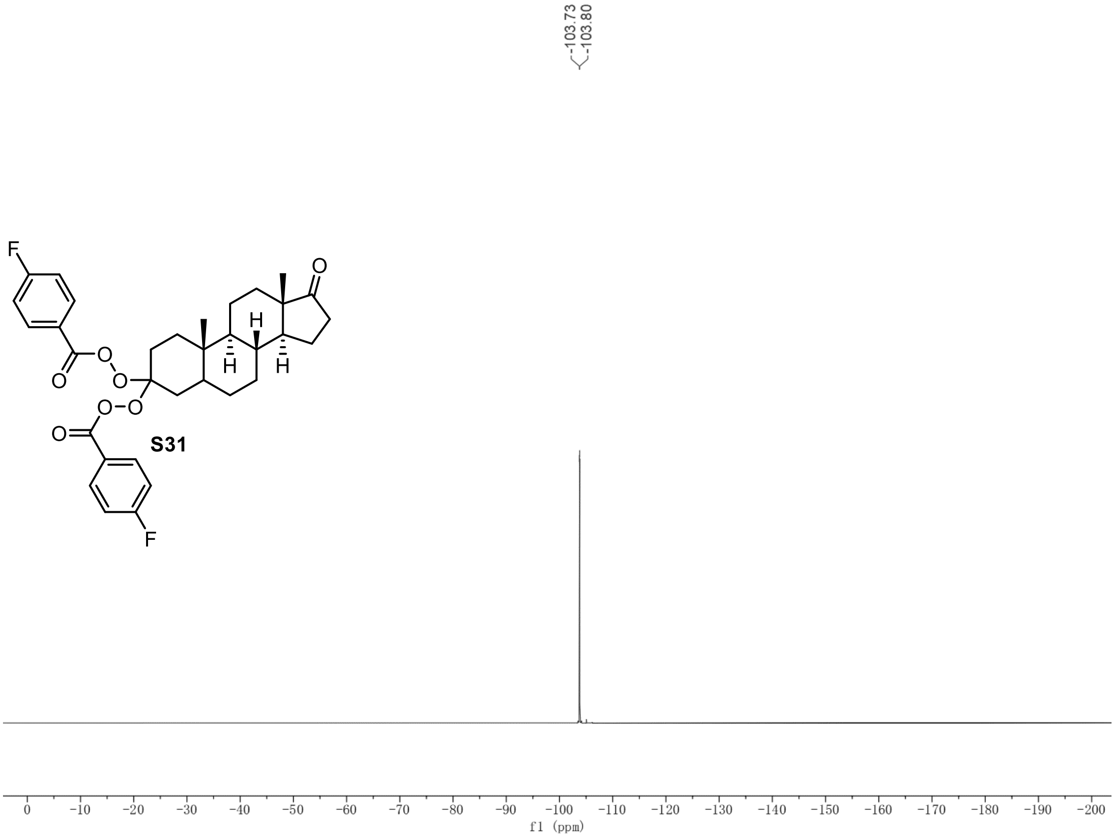

^{13}C NMR (100 MHz, $CDCl_3$) of **S31**

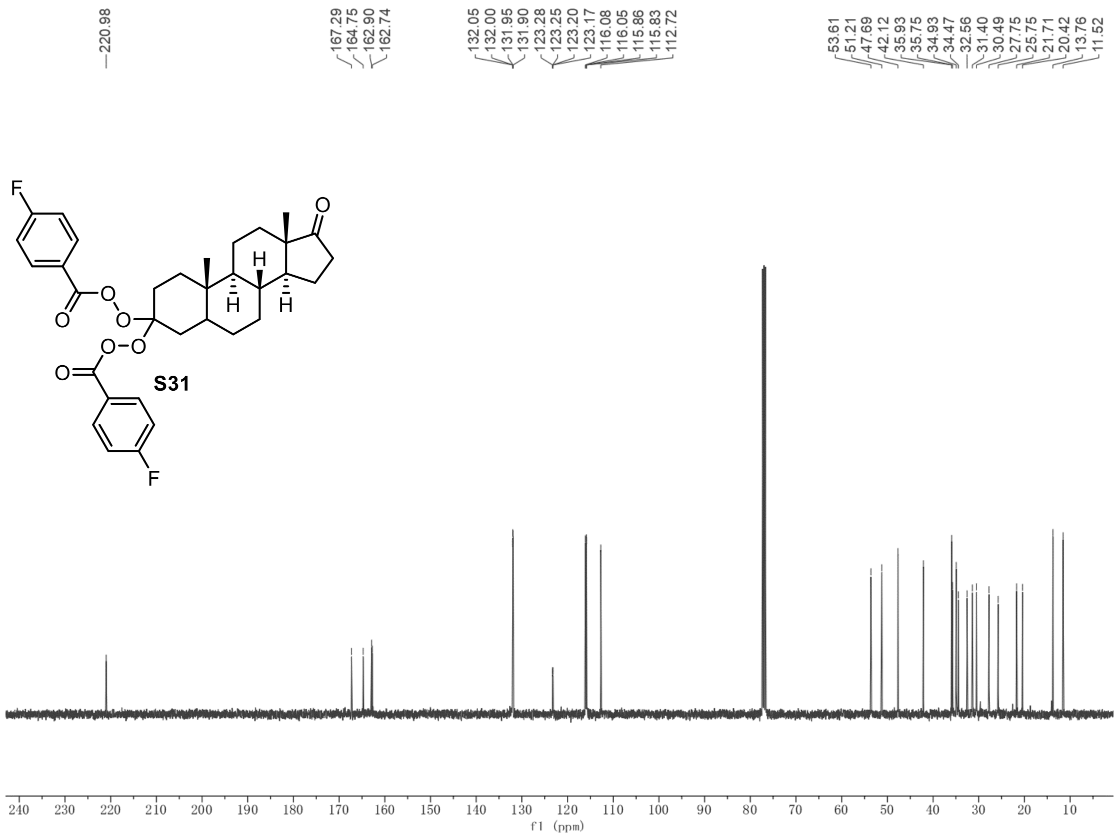

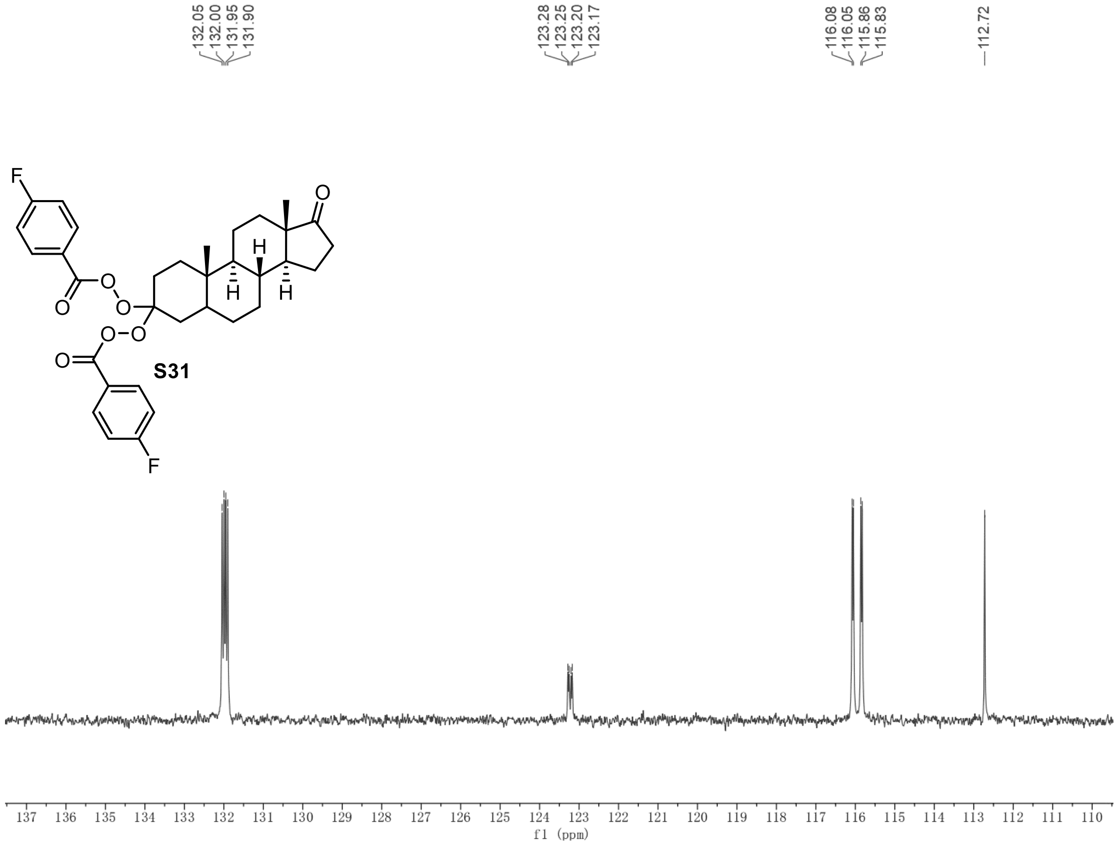

^{1}H NMR (400 MHz, $CDCl_3$) of **S32**

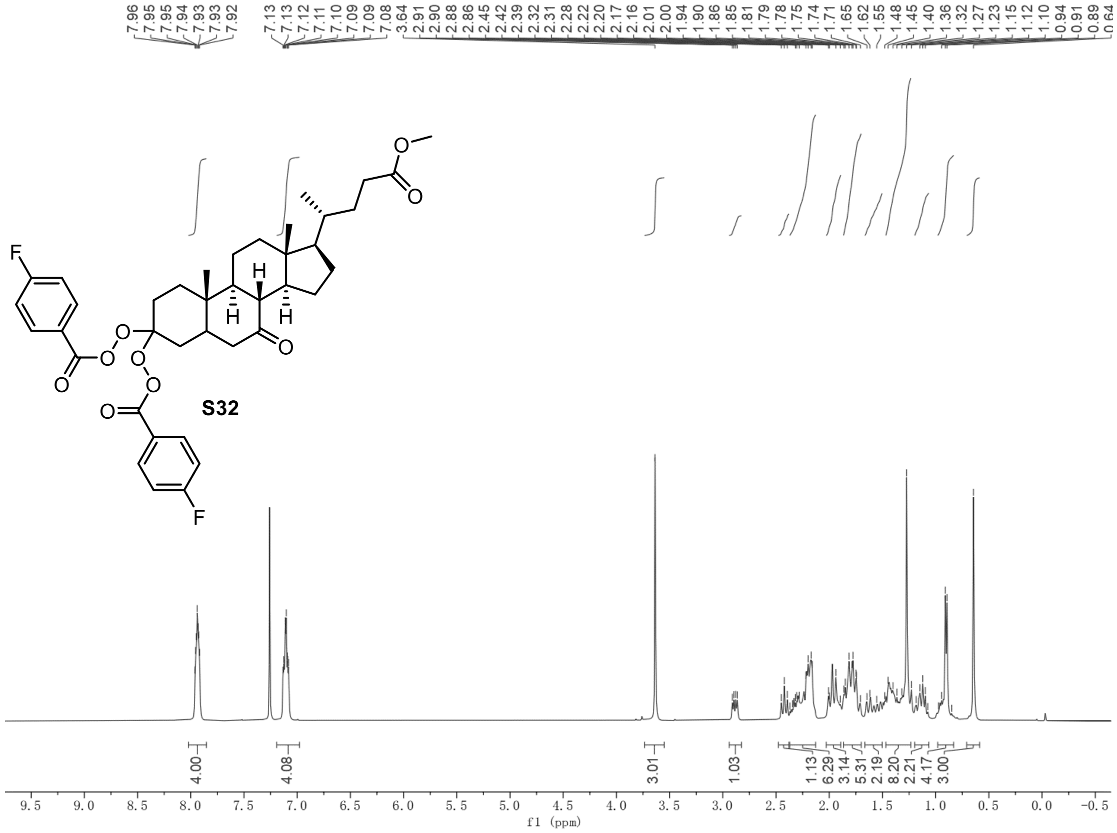

^{19}F NMR (376 MHz, $CDCl_3$) of **S32**

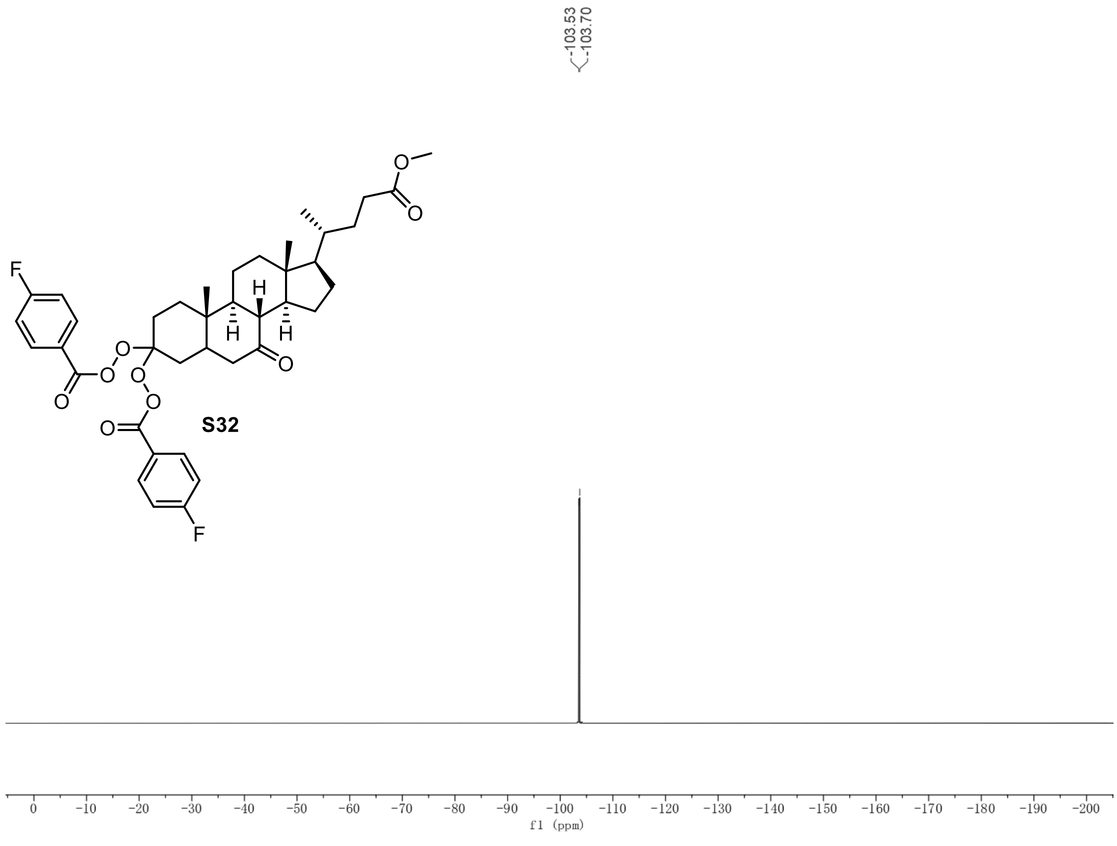

^{13}C NMR (100 MHz, $CDCl_3$) of **S32**

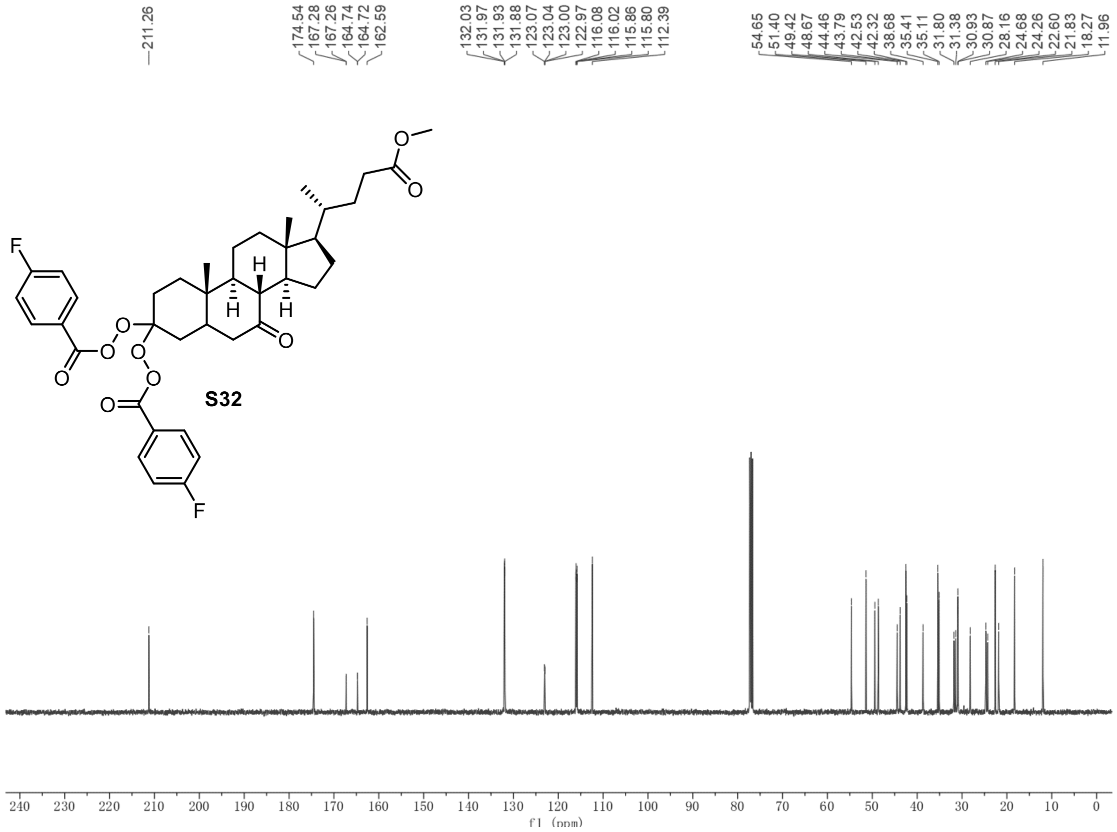

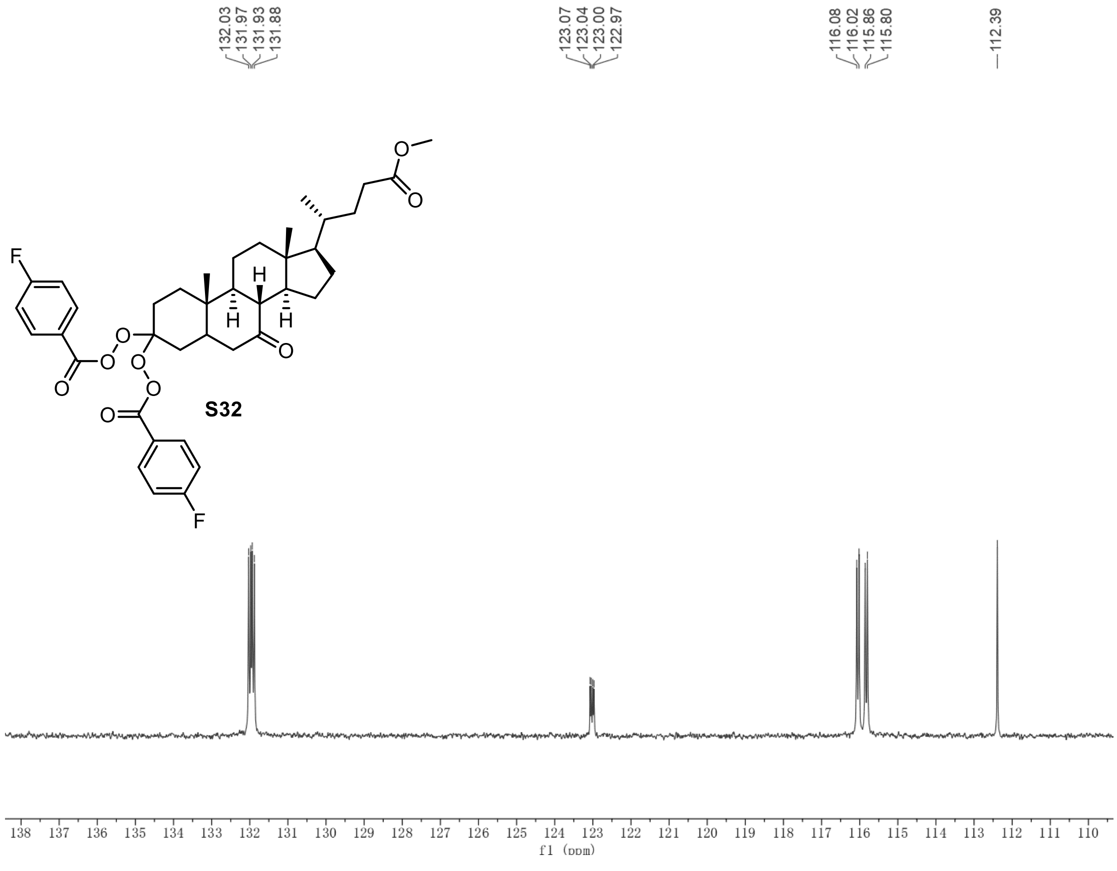

^{1}H NMR (400 MHz, $CDCl_3$) of **S33**

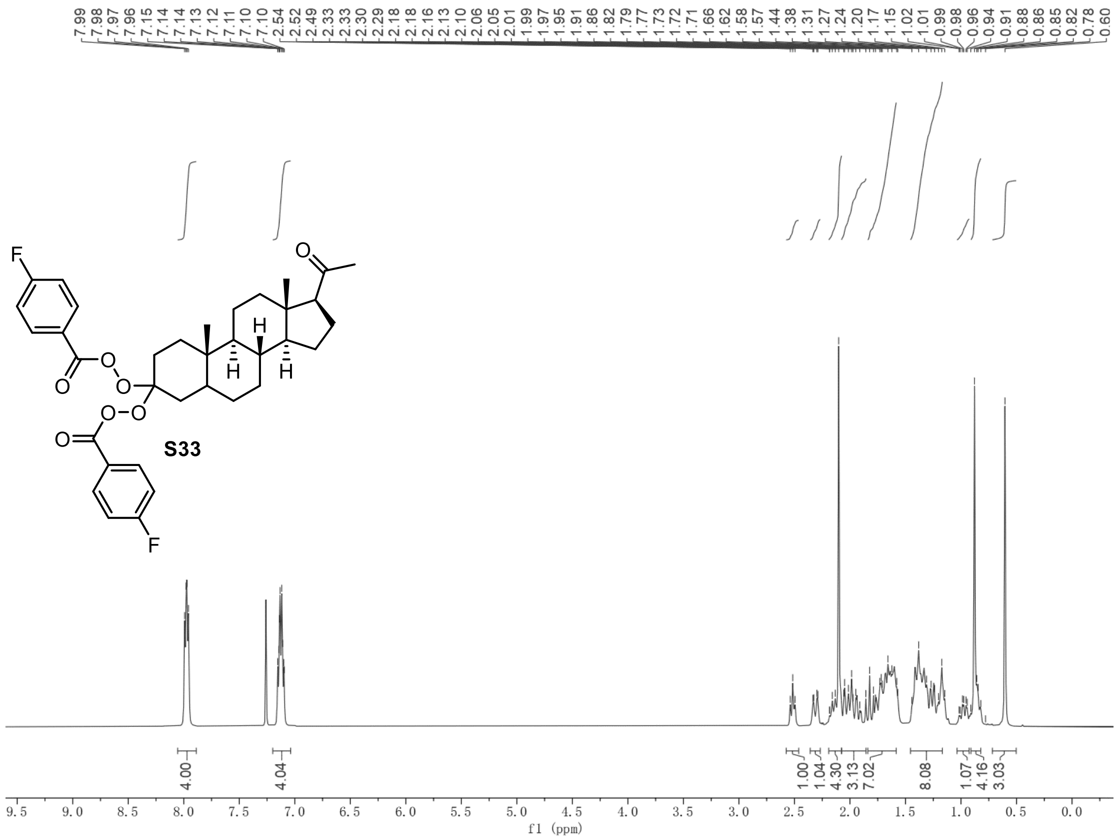

^{19}F NMR (376 MHz, $CDCl_3$) of **S33**

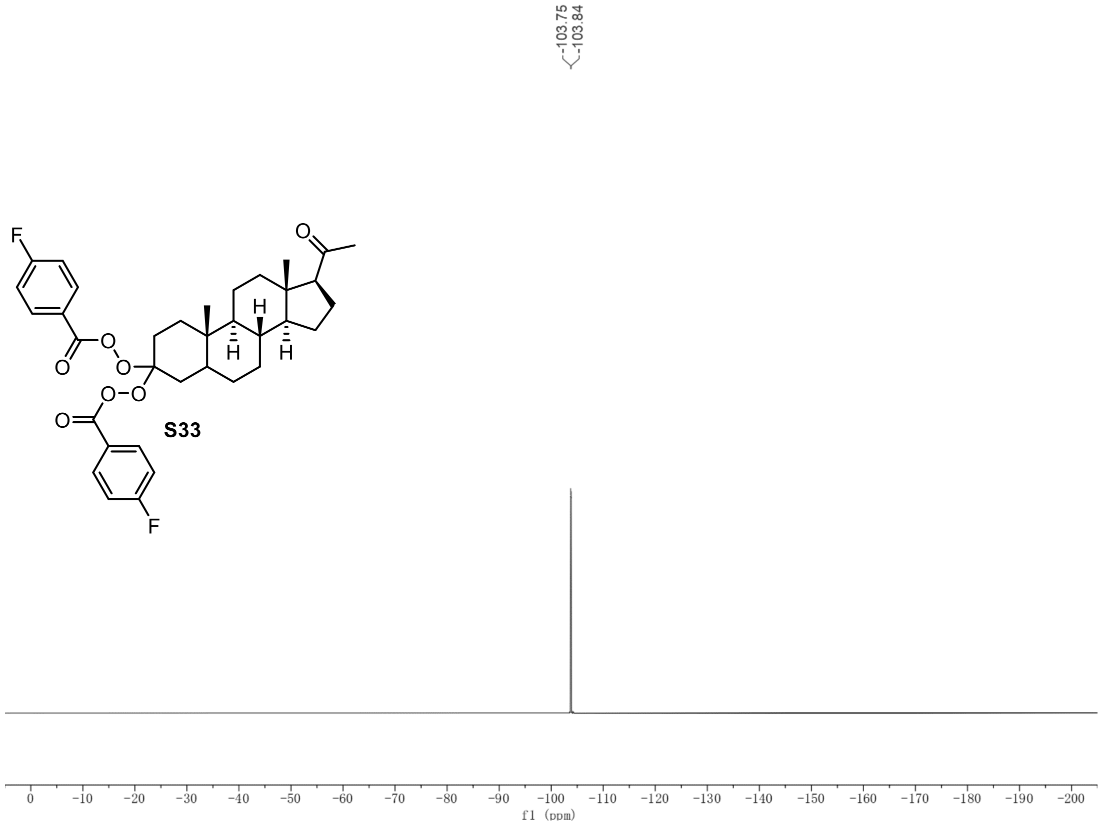

^{13}C NMR (100 MHz, $CDCl_3$) of **S33**

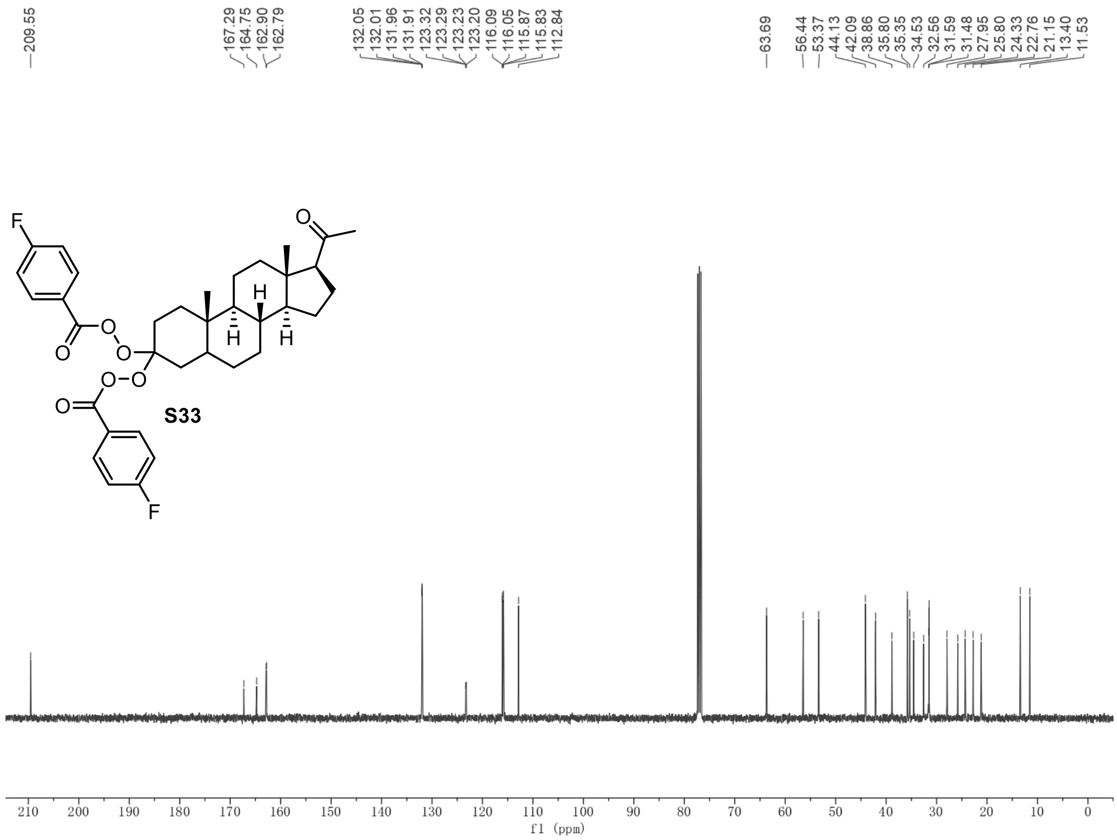

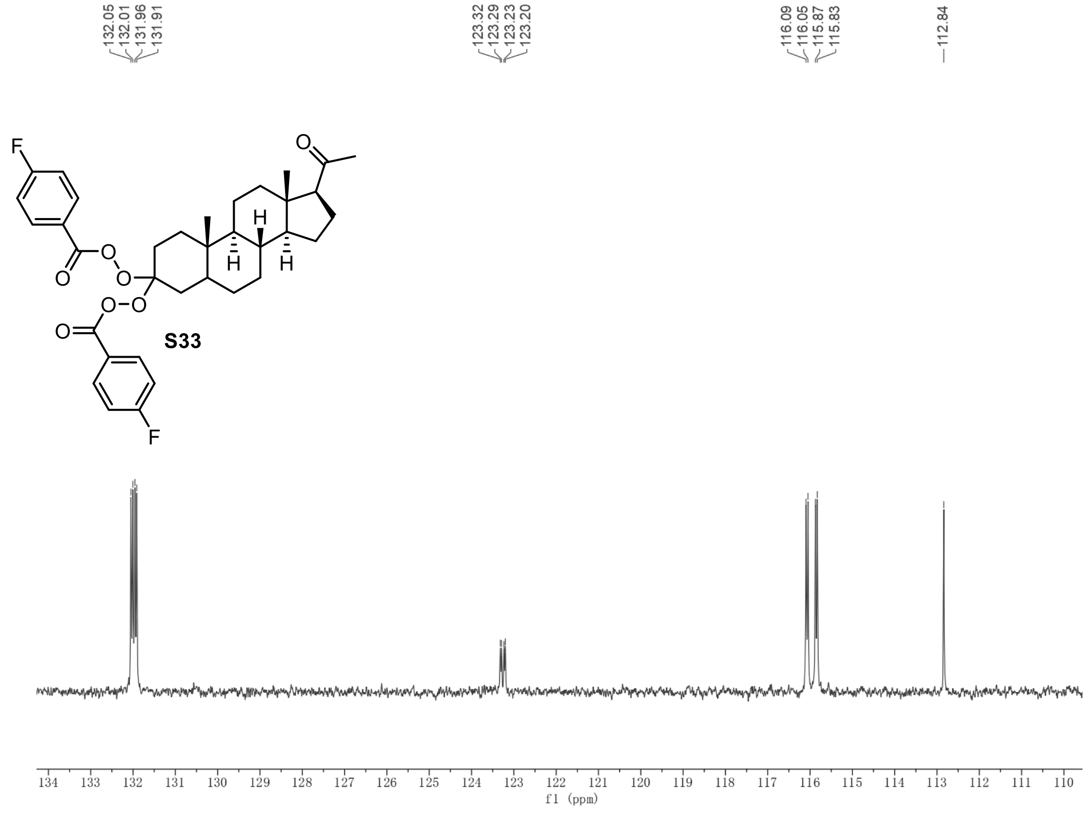

^{1}H NMR (400 MHz, $CDCl_3$) of **S34**

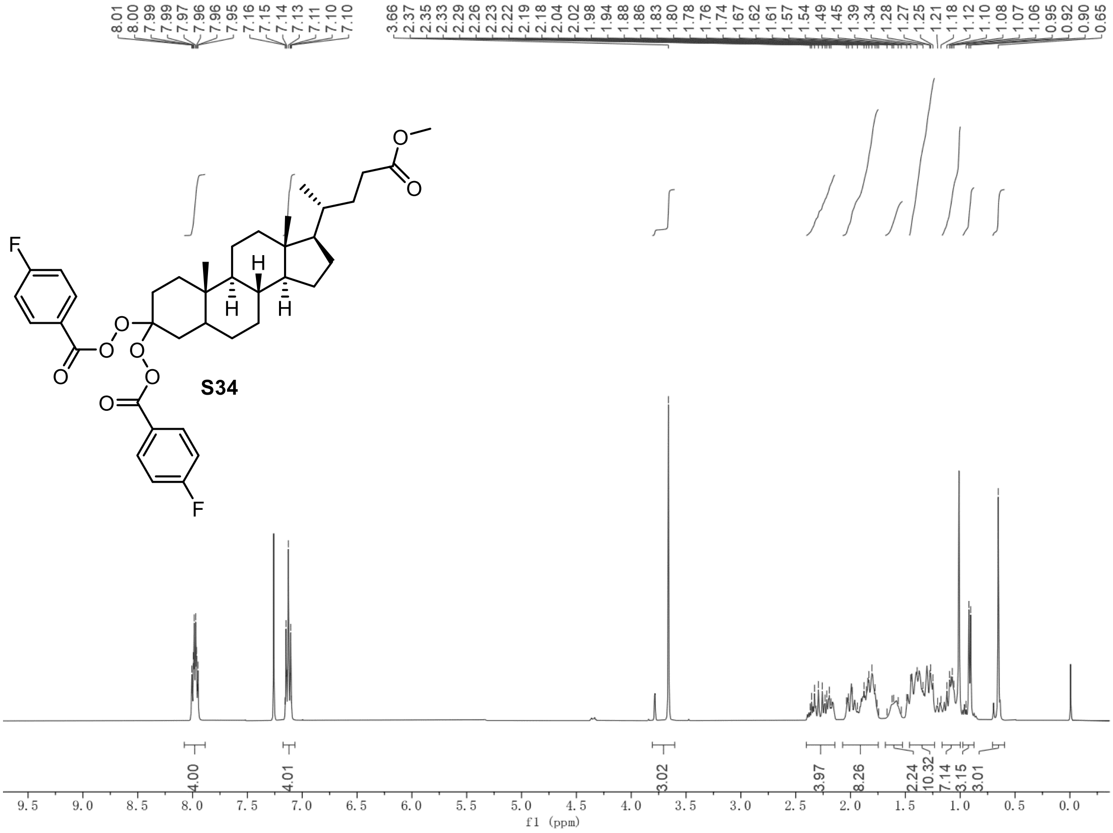

^{19}F NMR (376 MHz, $CDCl_3$) of **S34**

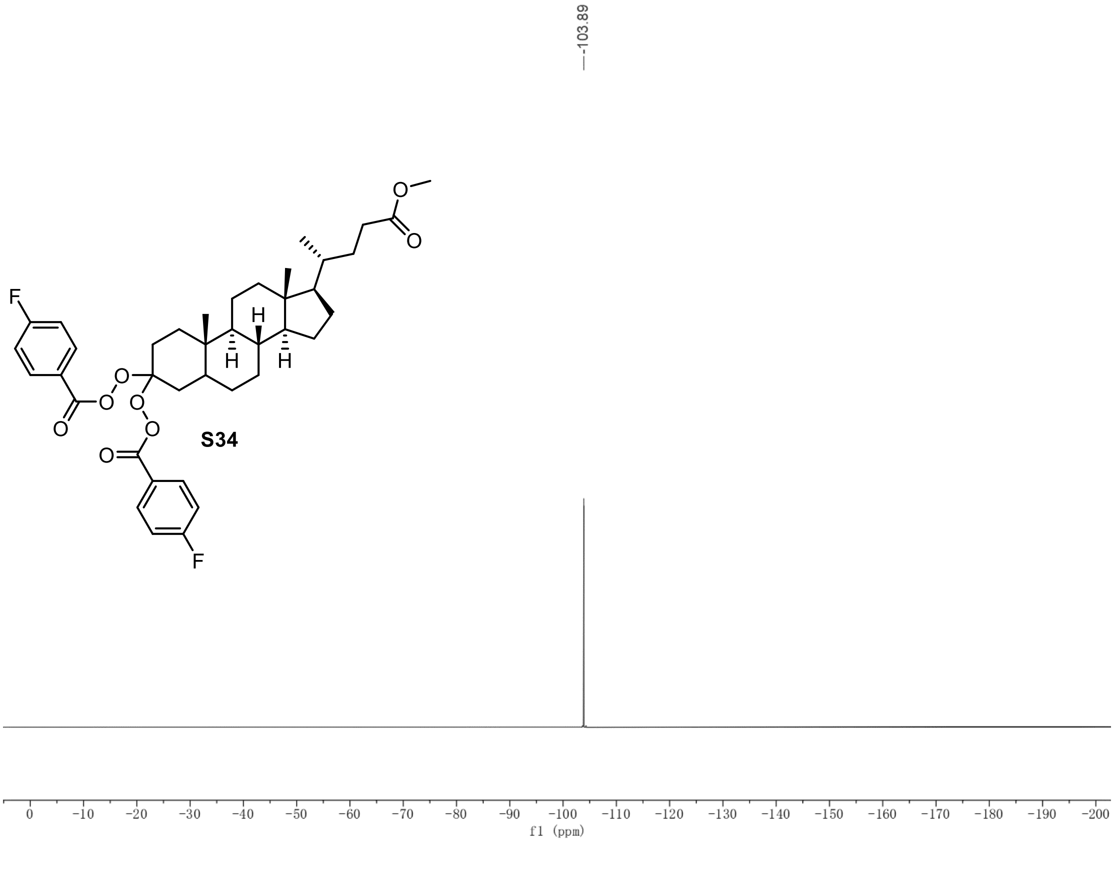

^{13}C NMR (100 MHz, $CDCl_3$) of **S34**

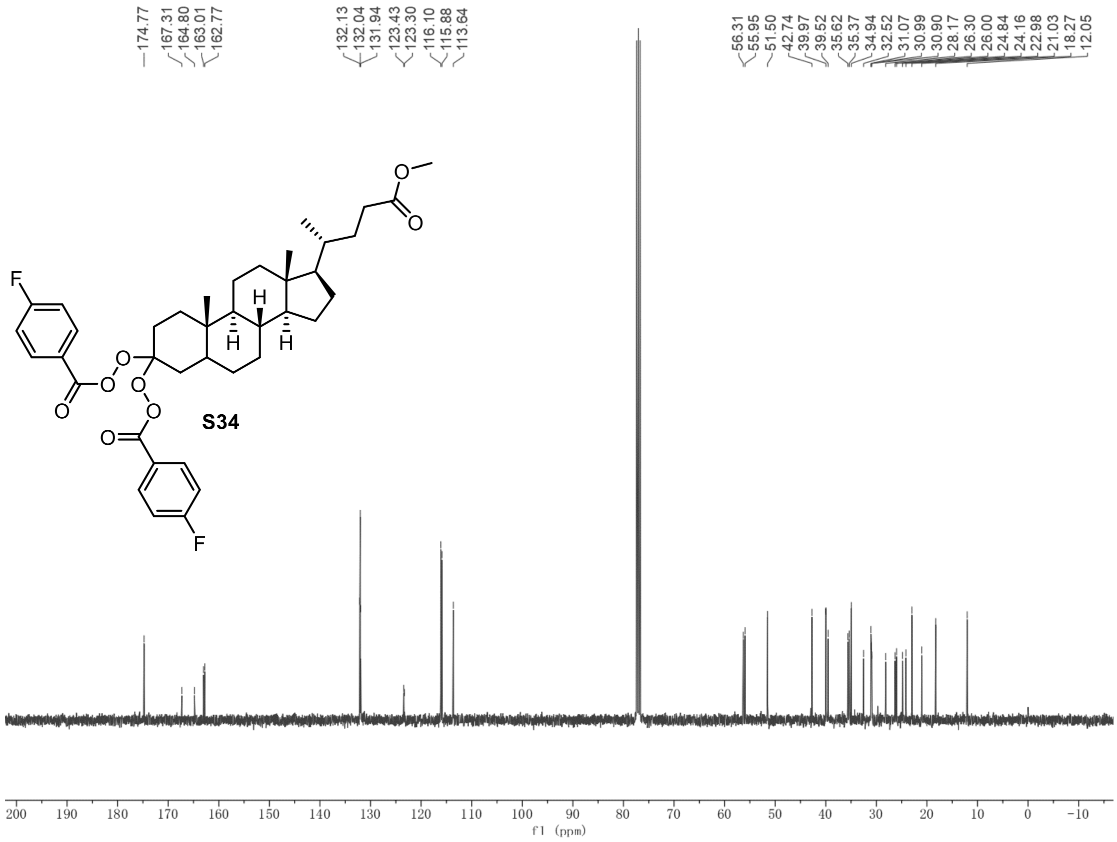

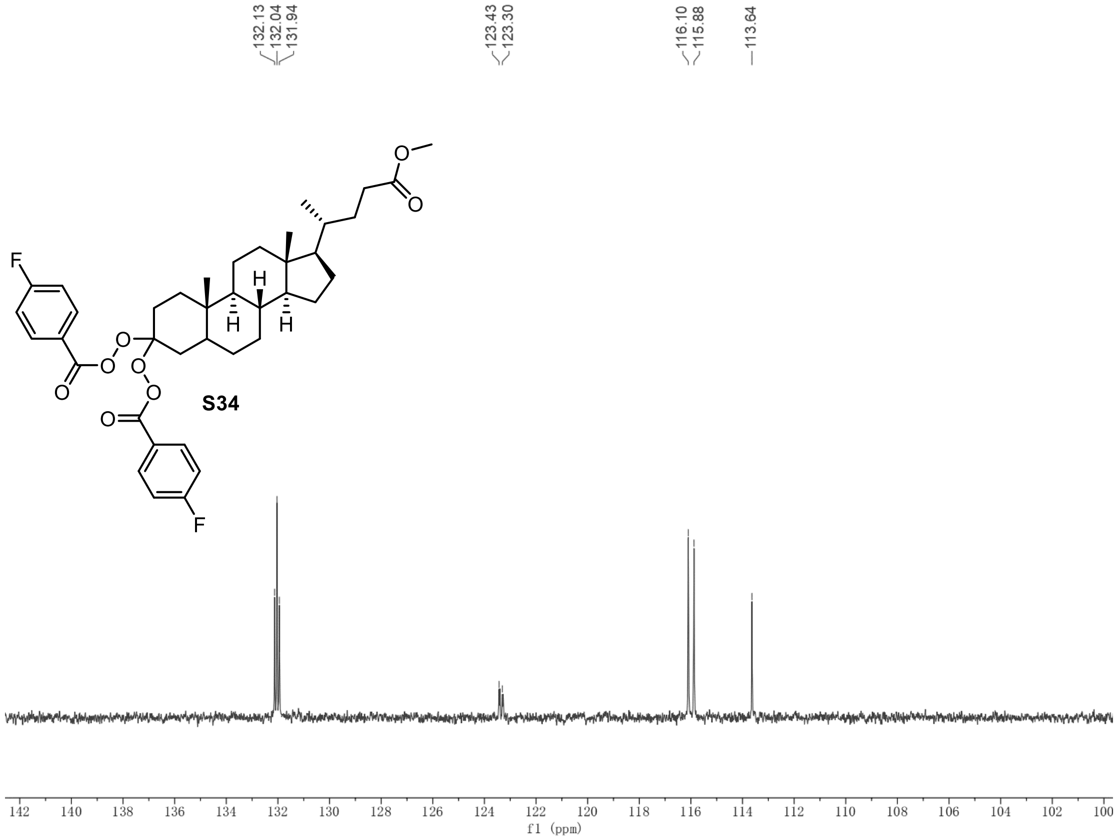

¹H NMR (400 MHz, CDCl$_3$) of **S35**

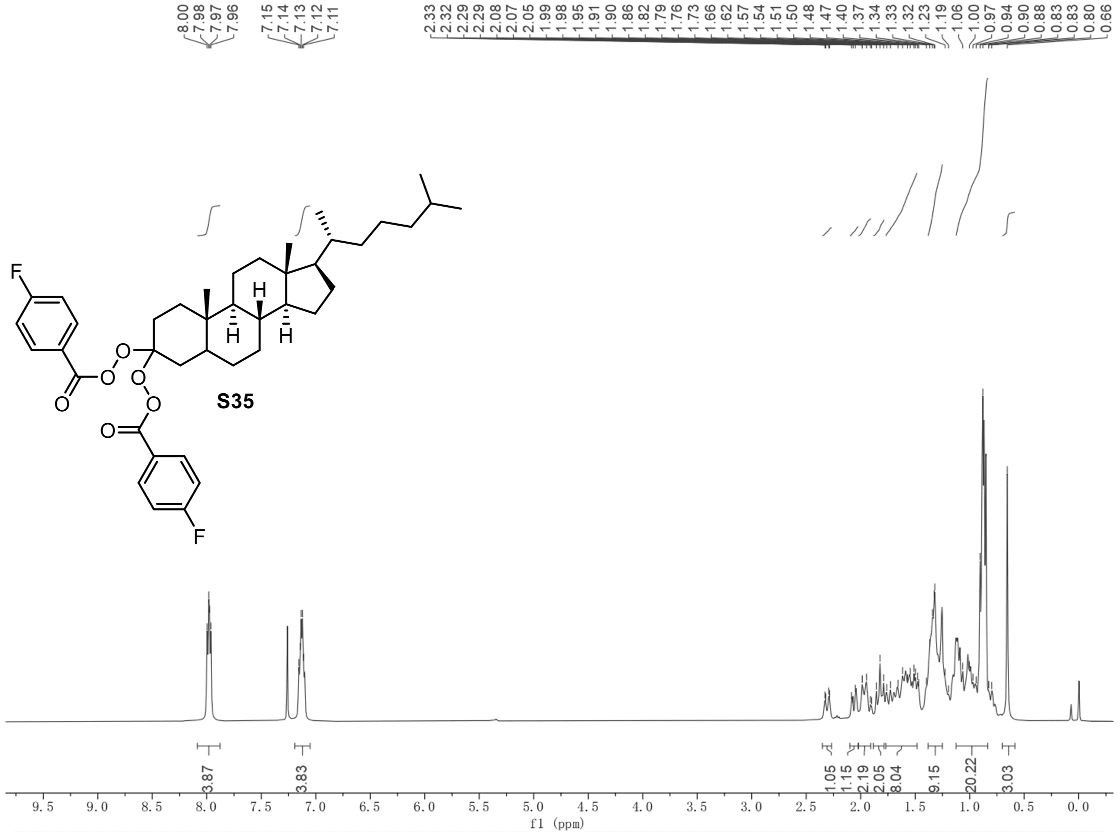

¹⁹F NMR (376 MHz, CDCl$_3$) of **S35**

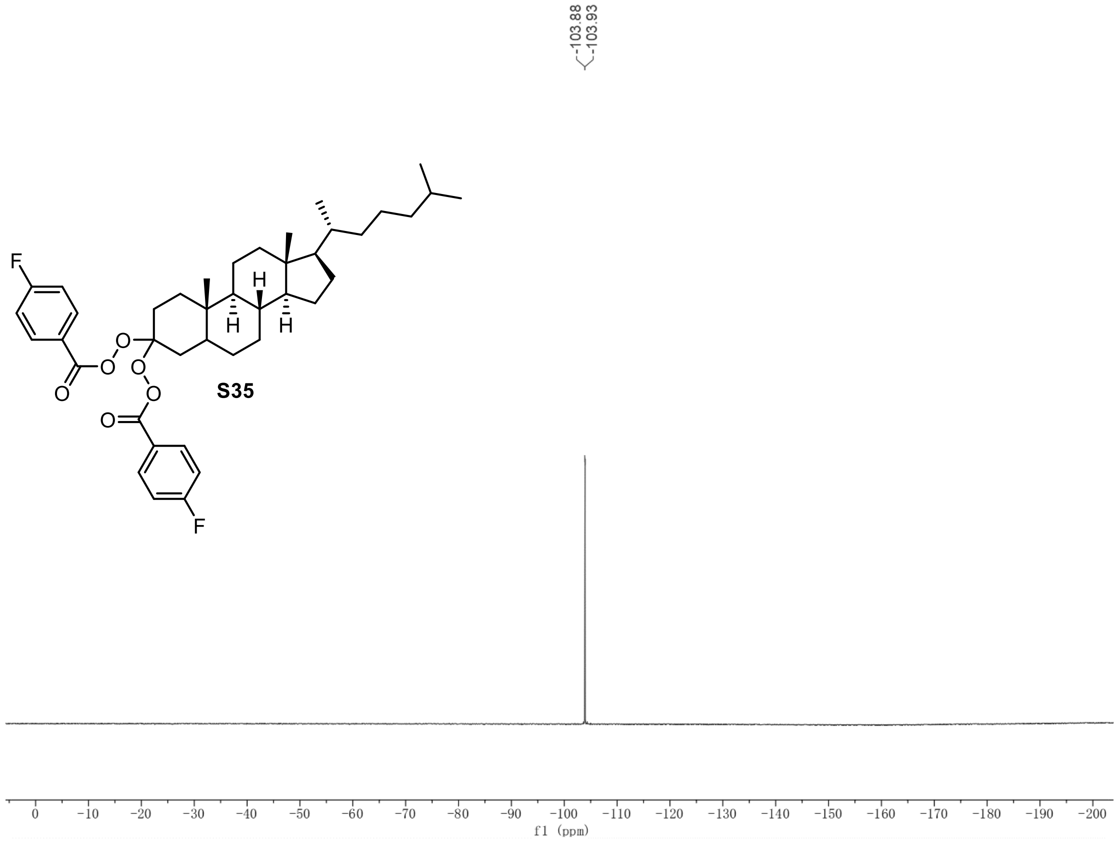

^{13}C NMR (100 MHz, $CDCl_3$) of **S35**

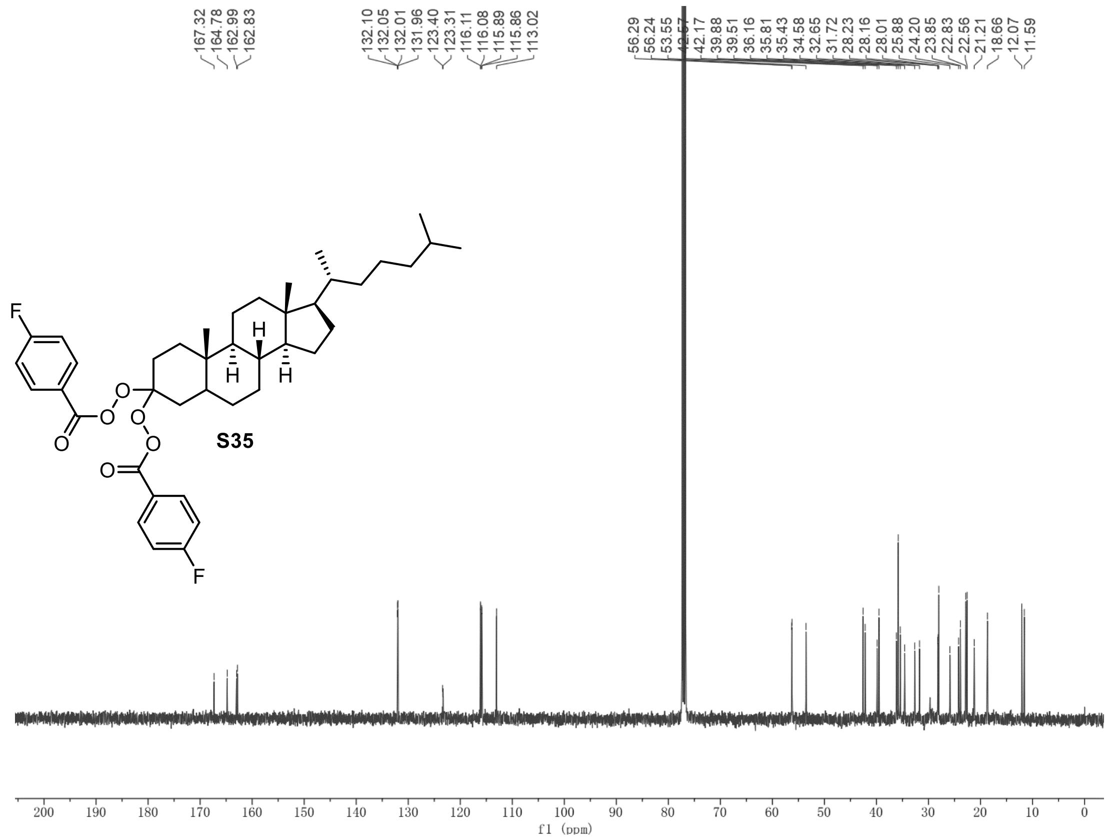

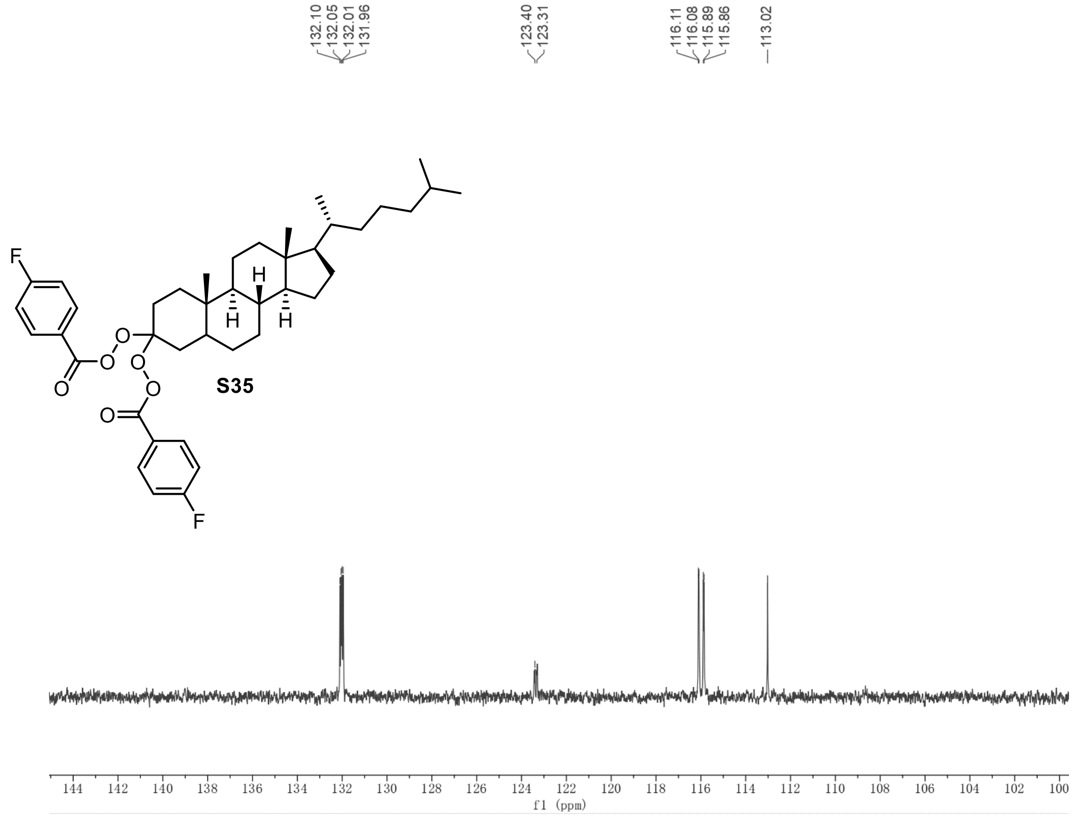

^{1}H NMR (400 MHz, $CDCl_3$) of **S36**

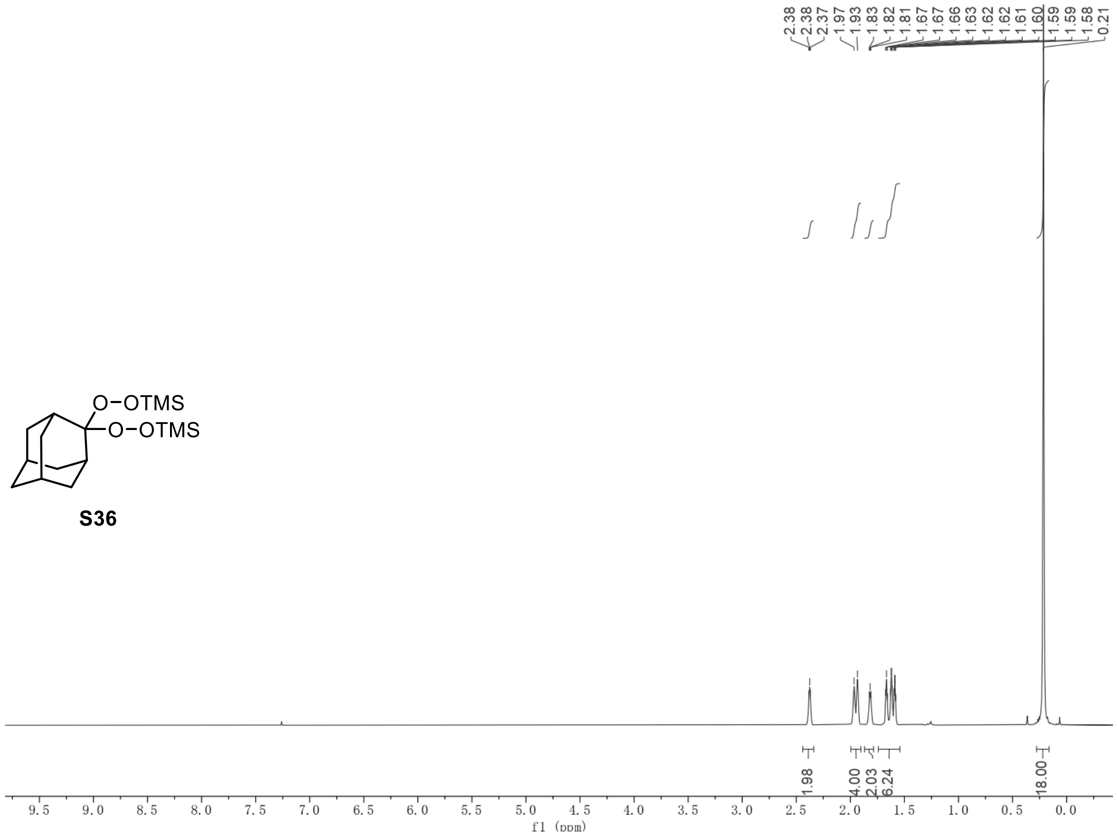

^{13}C NMR (100 MHz, $CDCl_3$) of **S36**

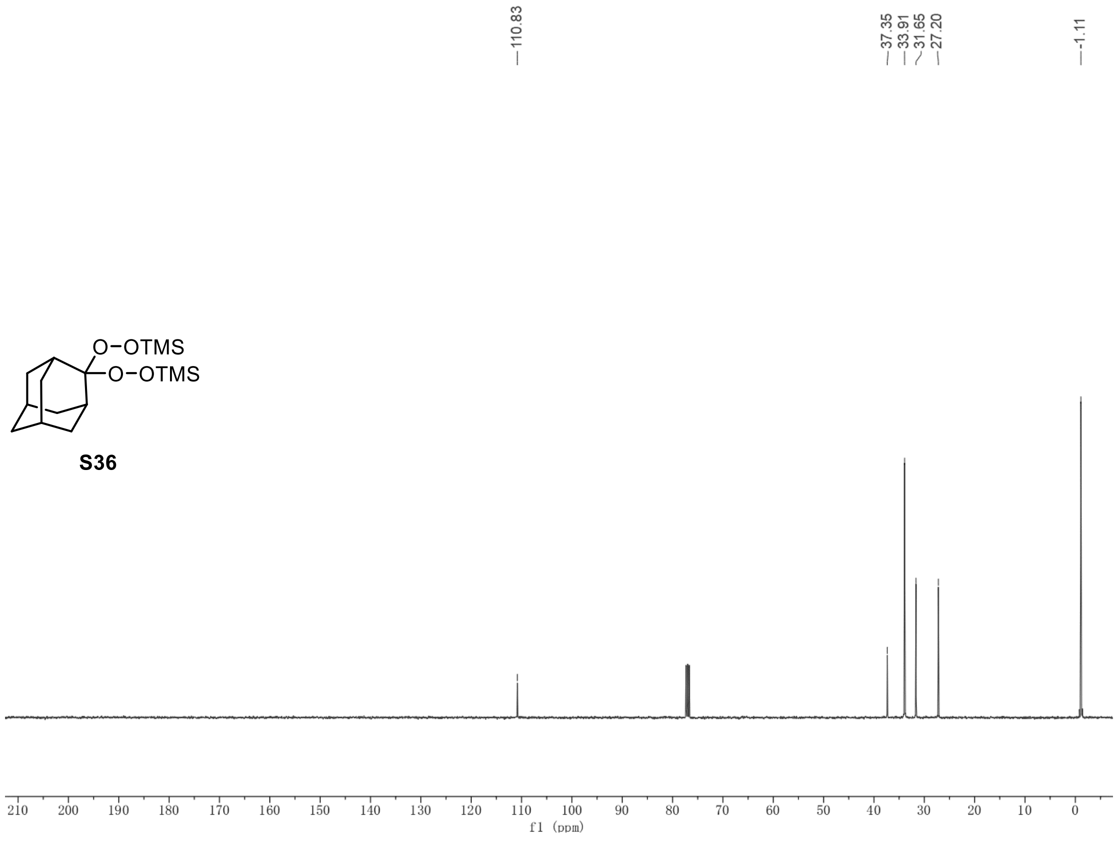

^{1}H NMR (400 MHz, $CDCl_3$) of **S37**

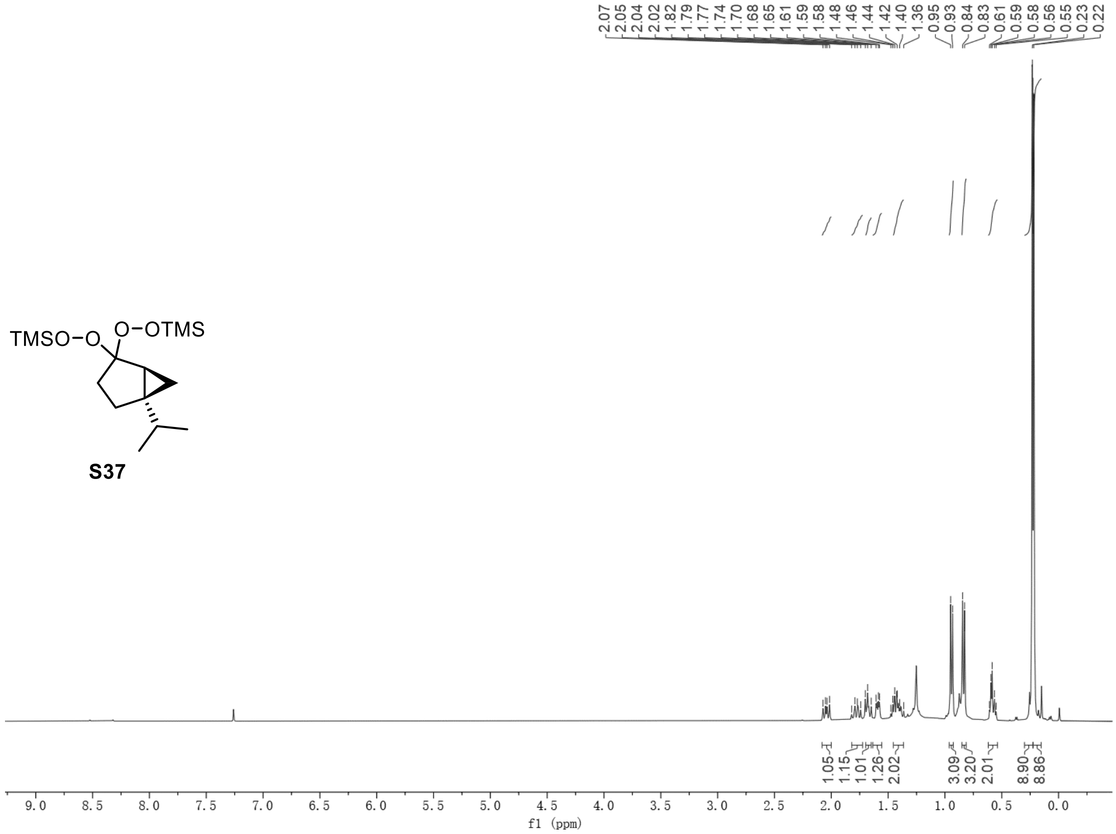

^{13}C NMR (100 MHz, $CDCl_3$) of **S37**

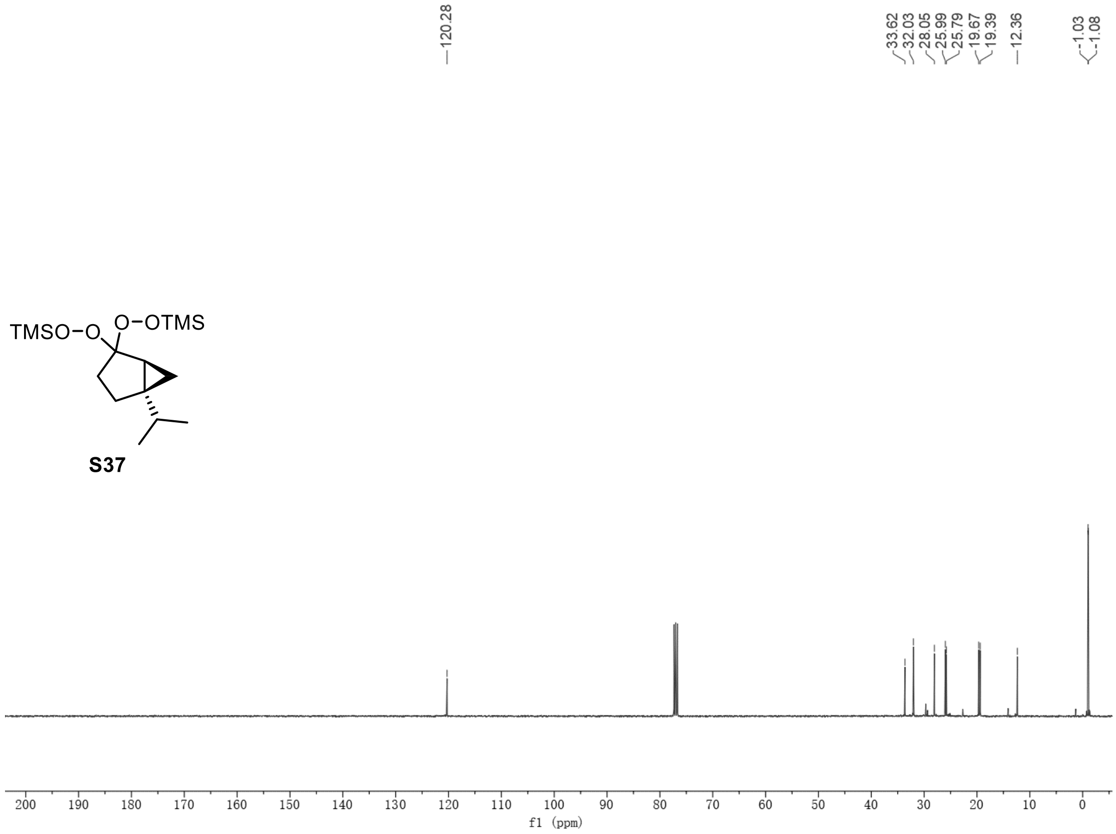

^{1}H NMR (400 MHz, $CDCl_3$) of **S38**

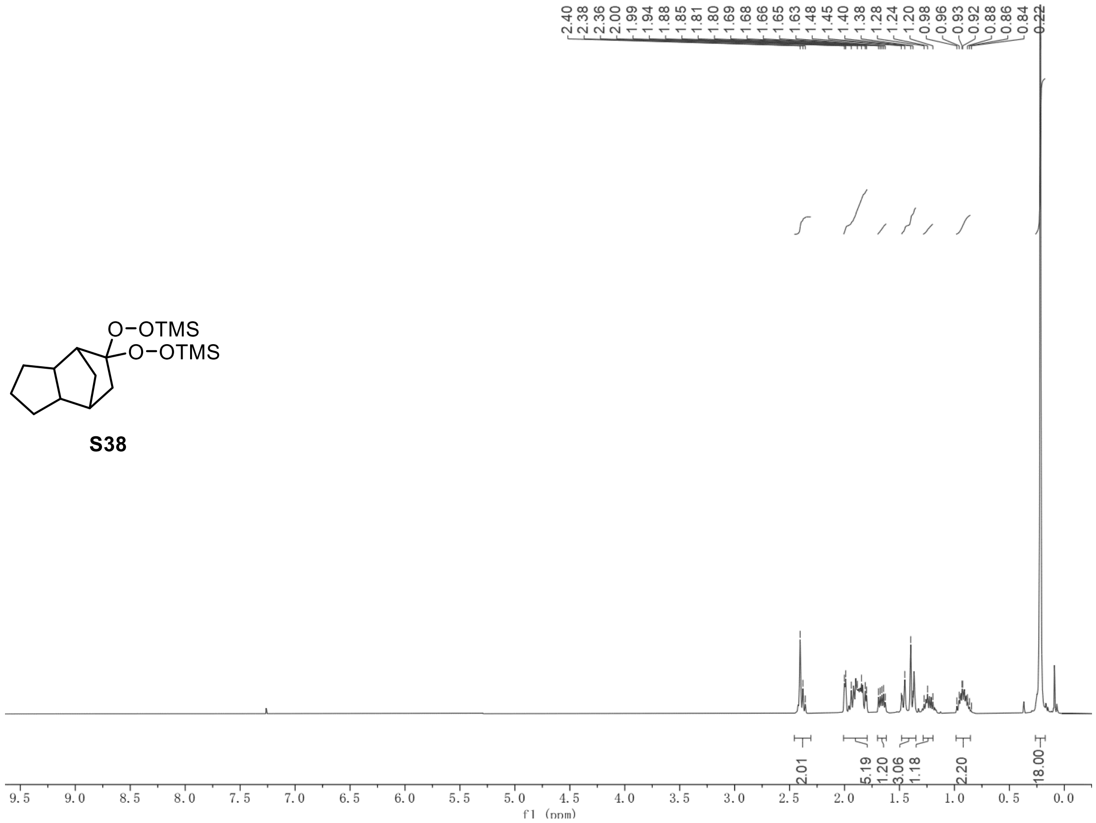

^{13}C NMR (100 MHz, $CDCl_3$) of **S38**

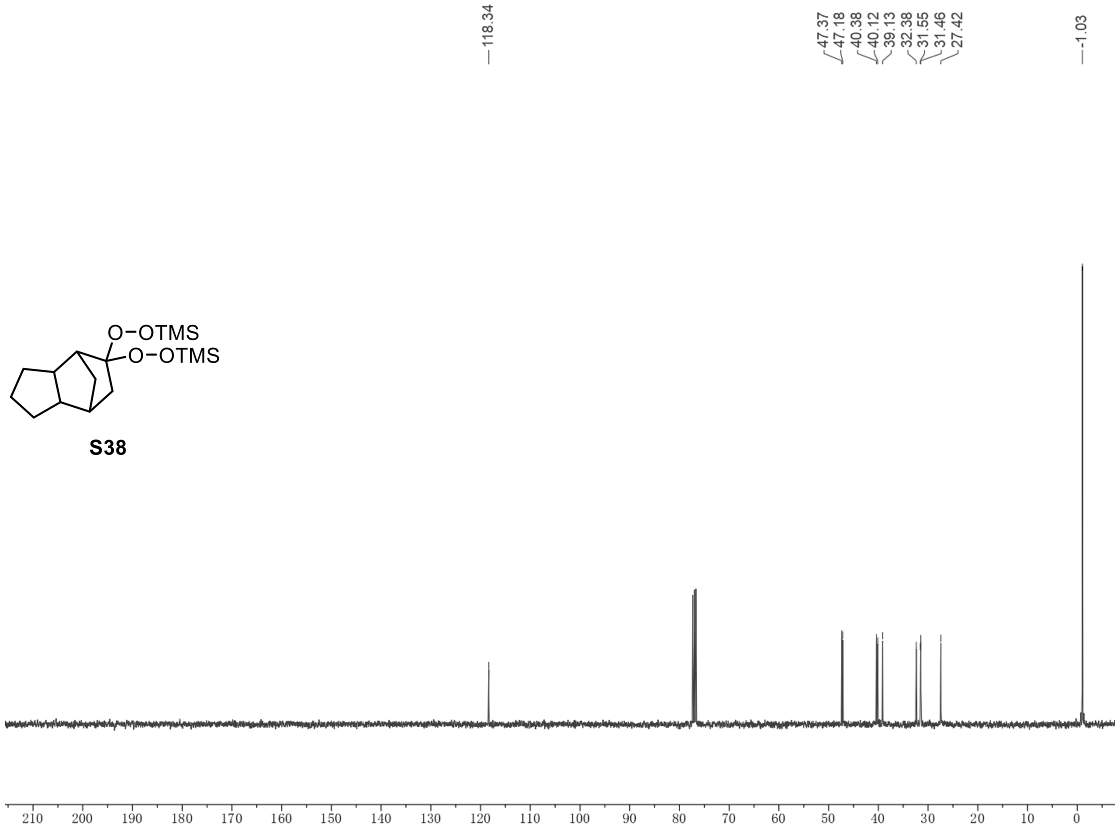

^{1}H NMR (400 MHz, $CDCl_3$) of **S39**

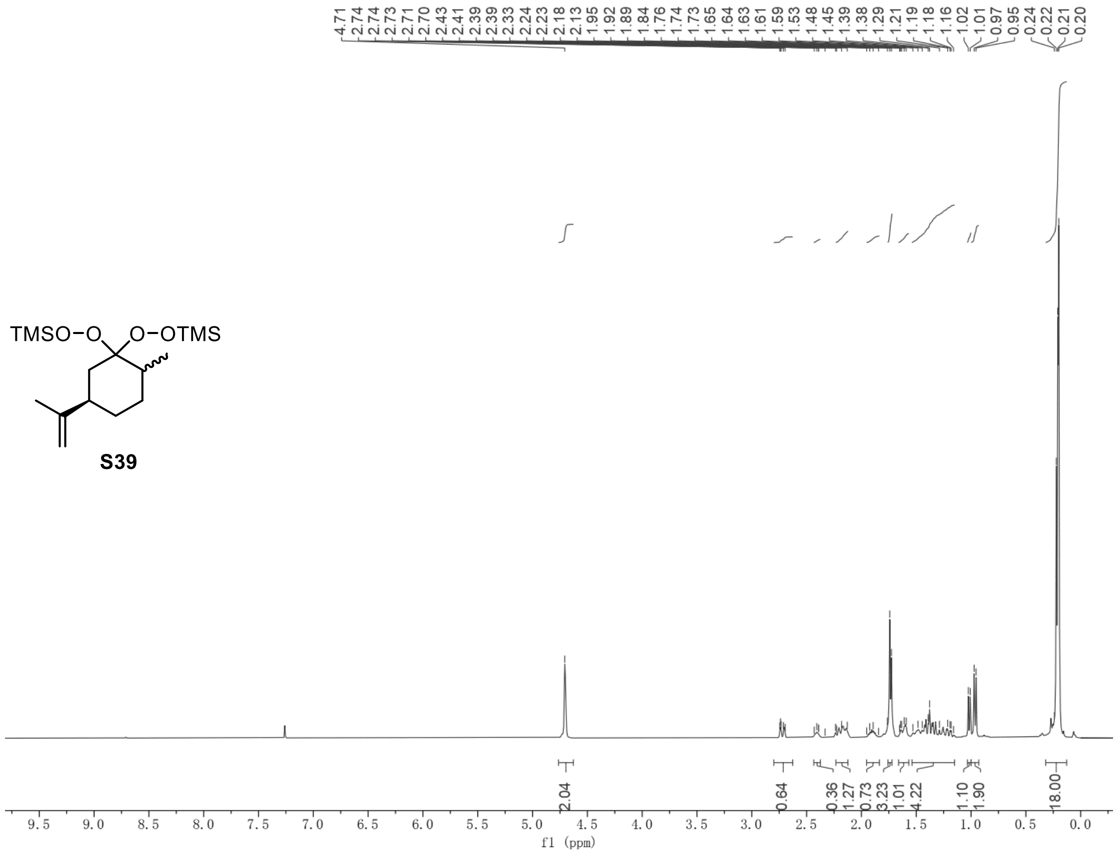

^{13}C NMR (100 MHz, $CDCl_3$) of **S39**

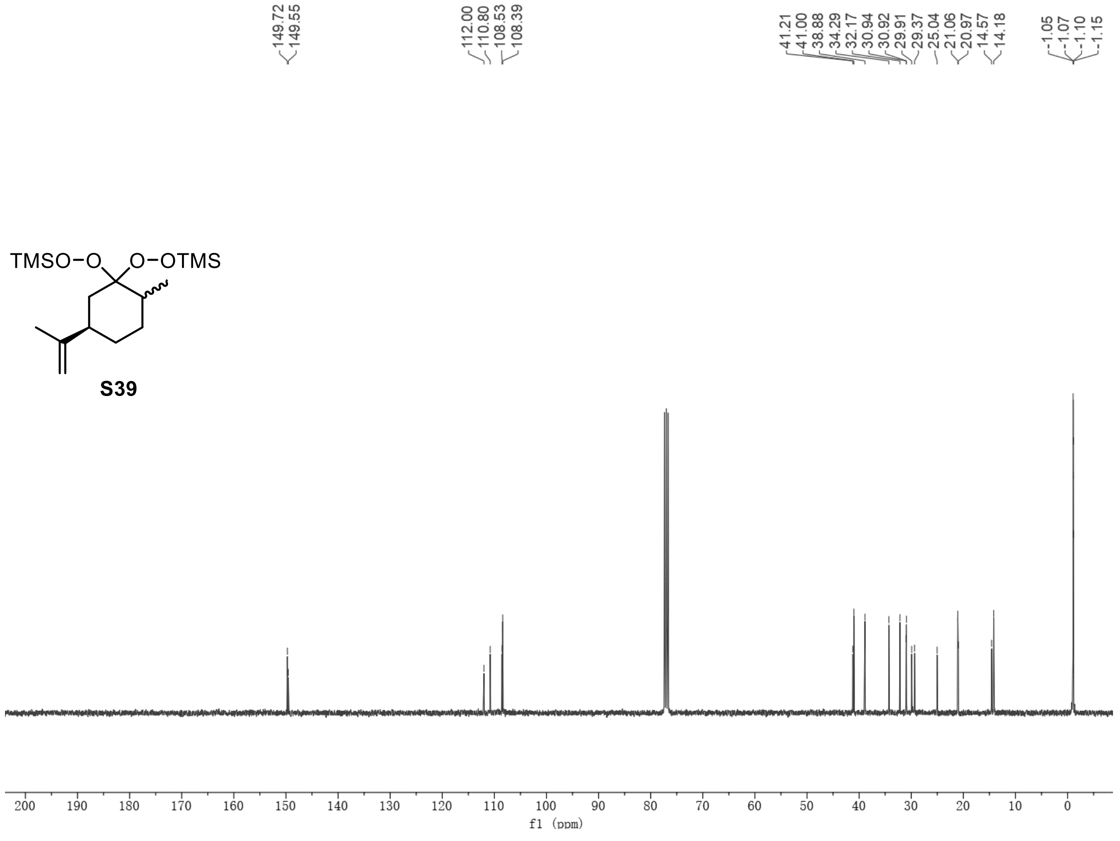

^{1}H NMR (400 MHz, $CDCl_3$) of **S40**

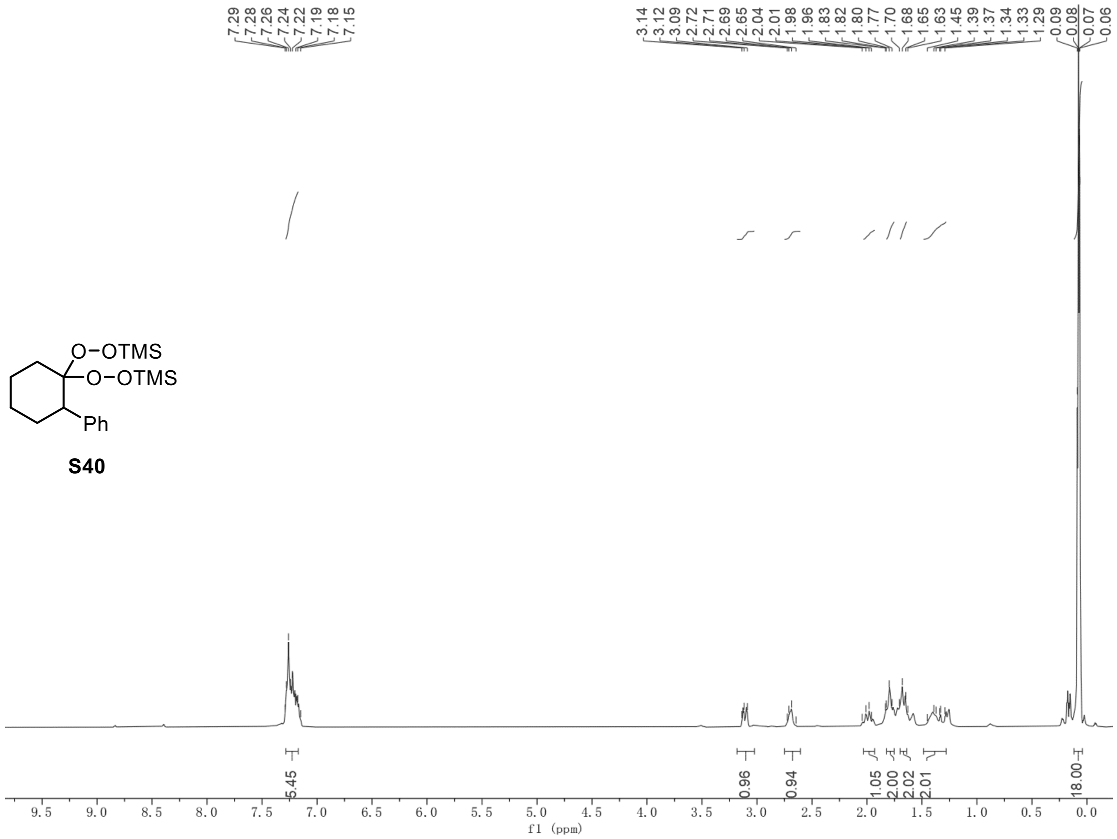

^{13}C NMR (100 MHz, $CDCl_3$) of **S40**

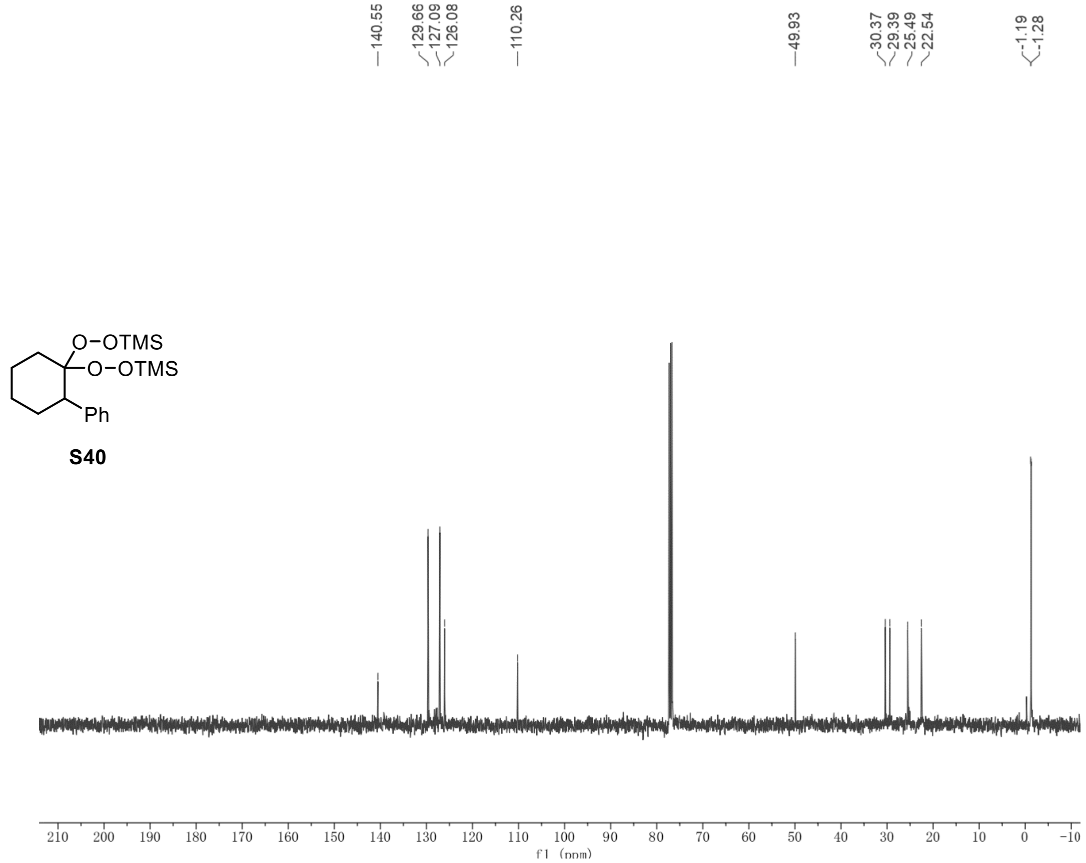

^{1}H NMR (400 MHz, $CDCl_3$) of **S41**

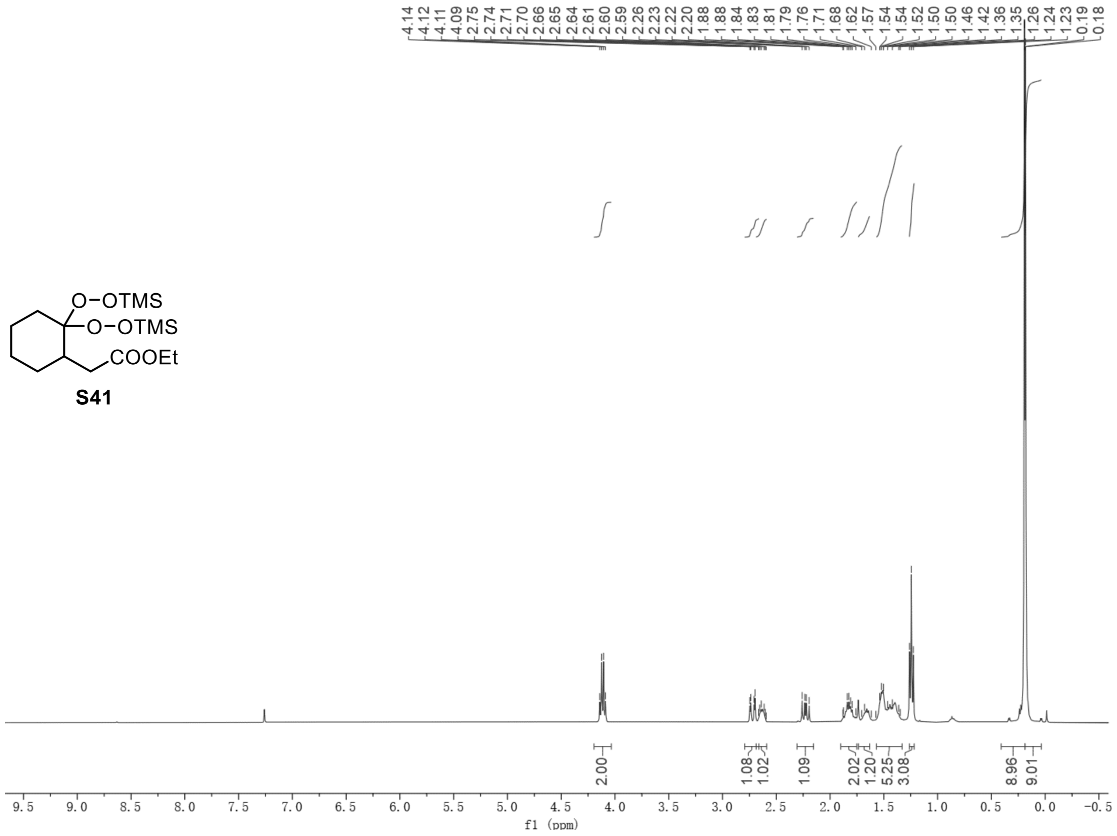

^{13}C NMR (100 MHz, $CDCl_3$) of **S41**

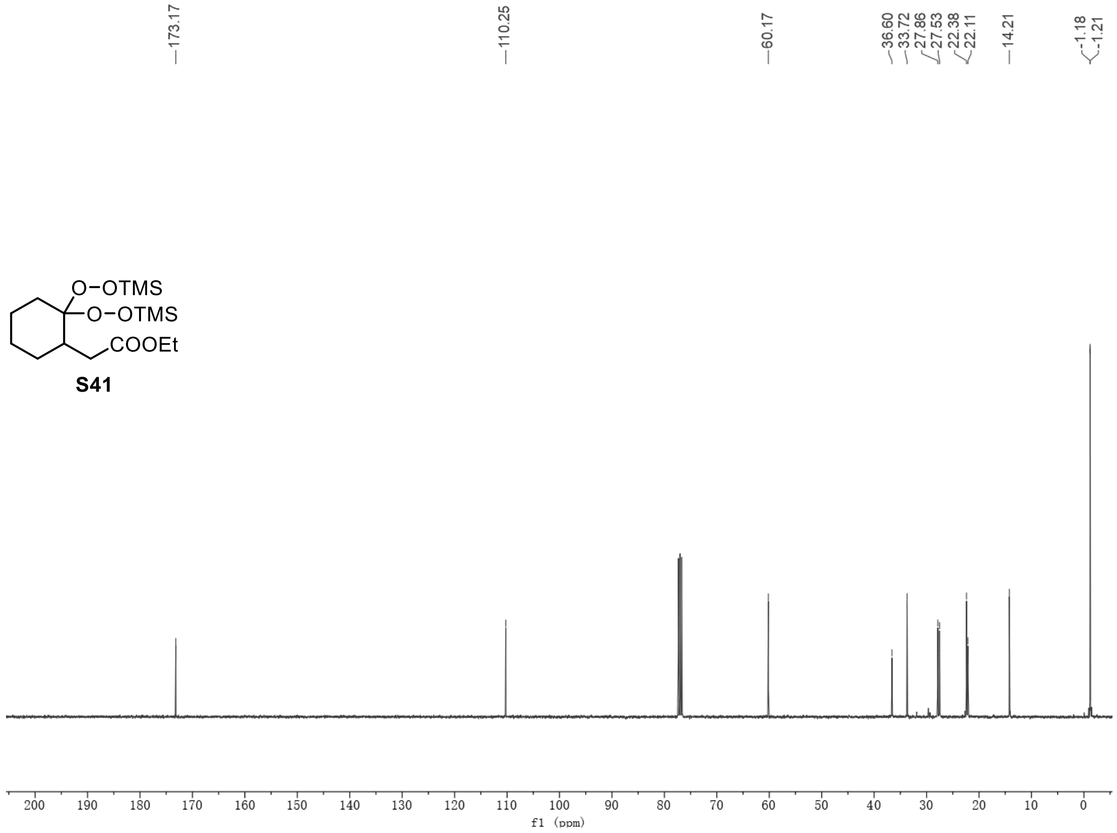

^{1}H NMR (400 MHz, $CDCl_3$) of **S42**

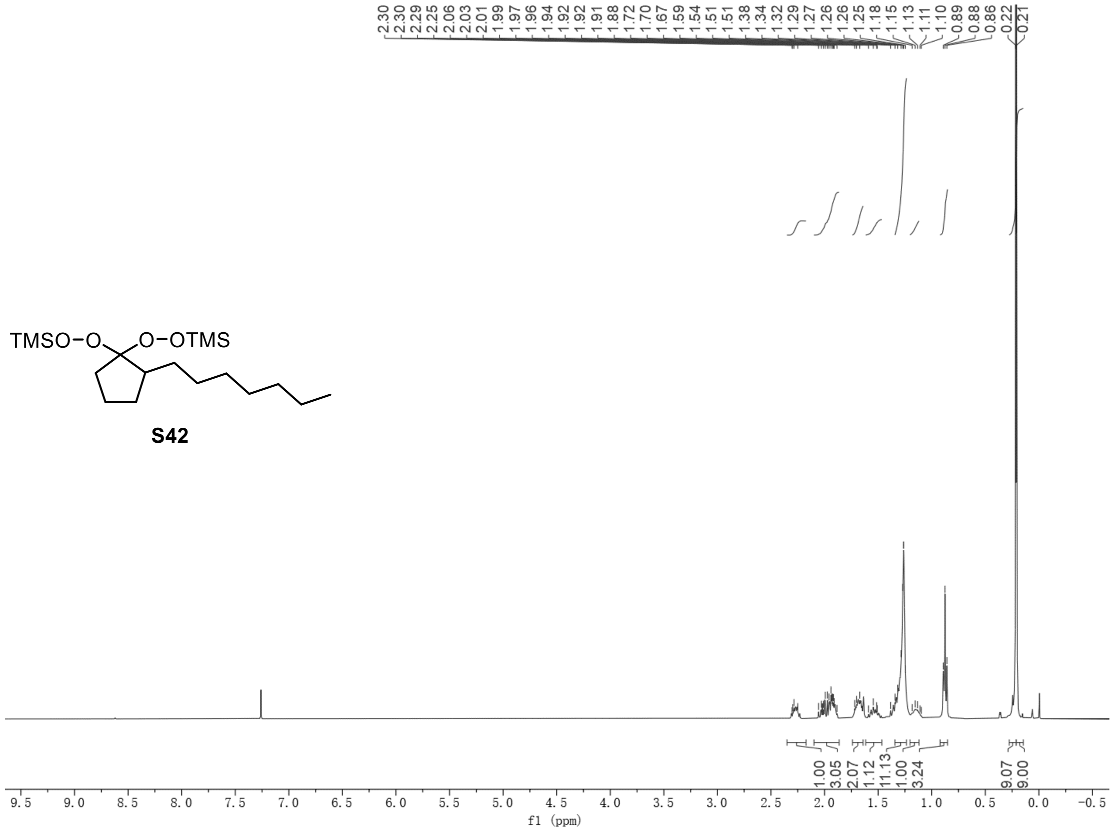

^{13}C NMR (100 MHz, $CDCl_3$) of **S42**

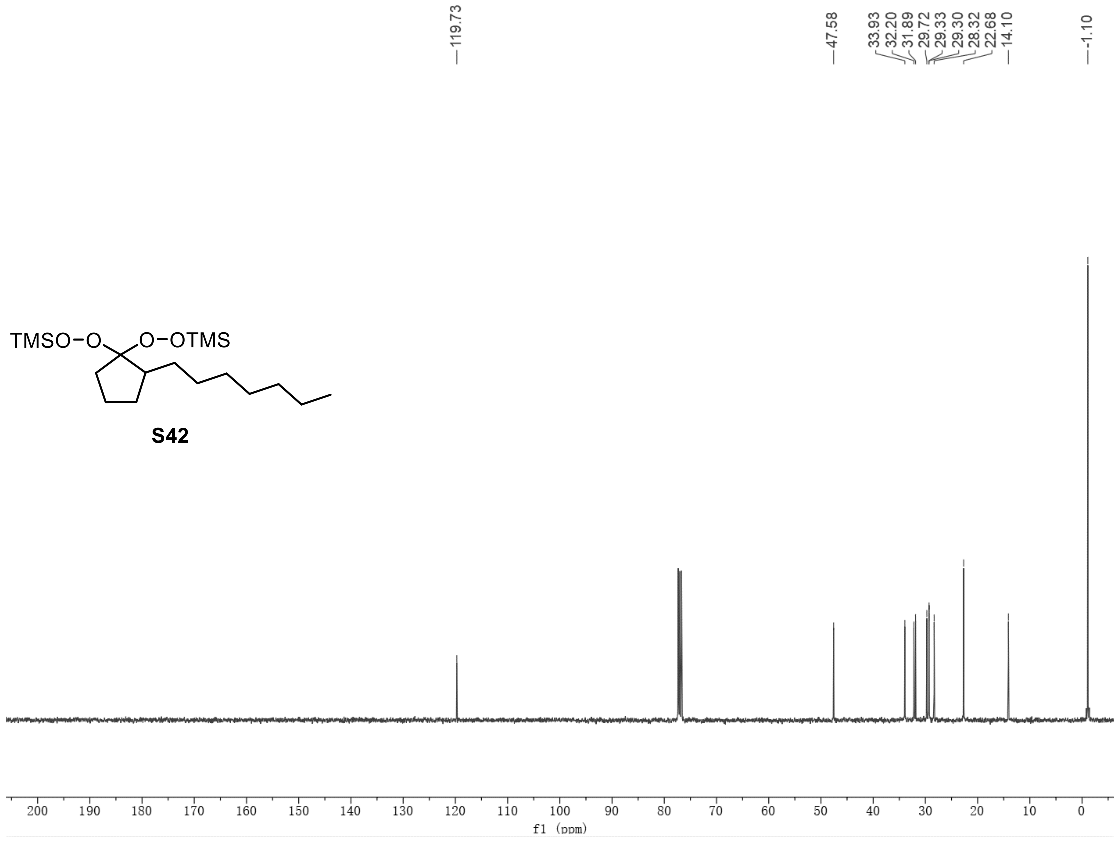

^{1}H NMR (400 MHz, $CDCl_3$) of **S43**

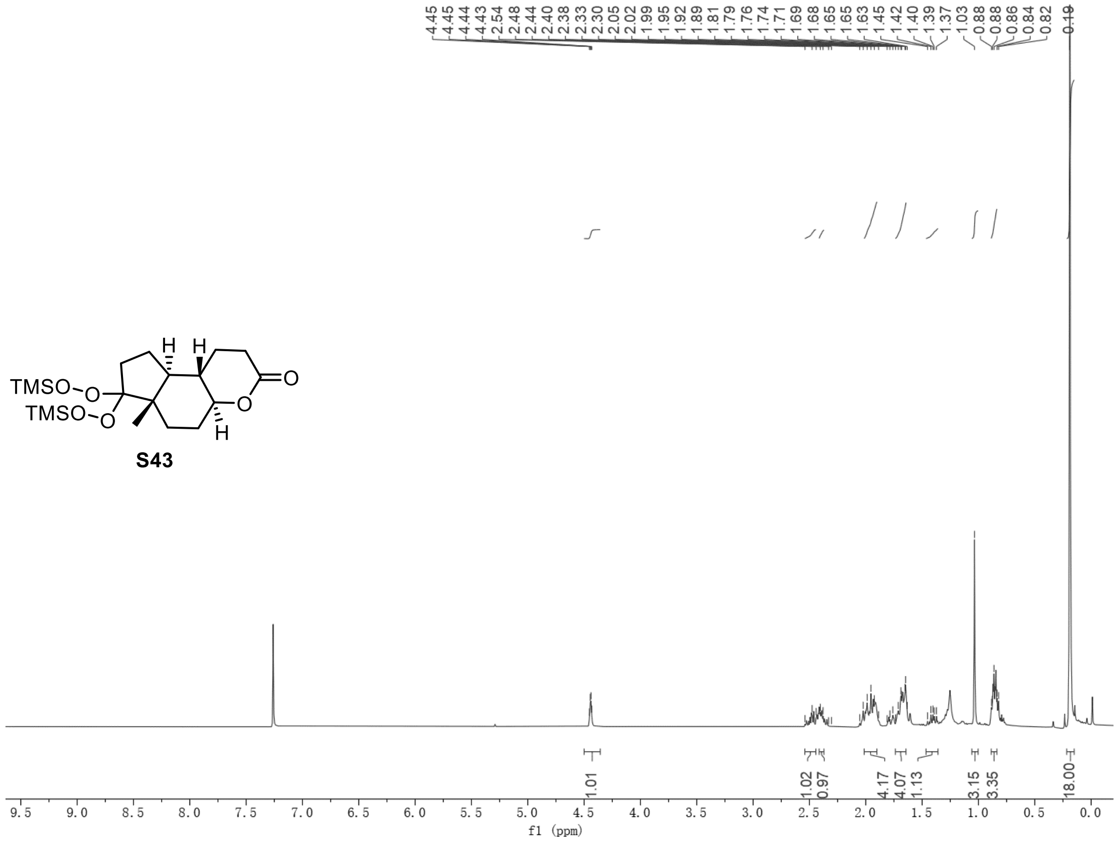

^{13}C NMR (100 MHz, $CDCl_3$) of **S43**

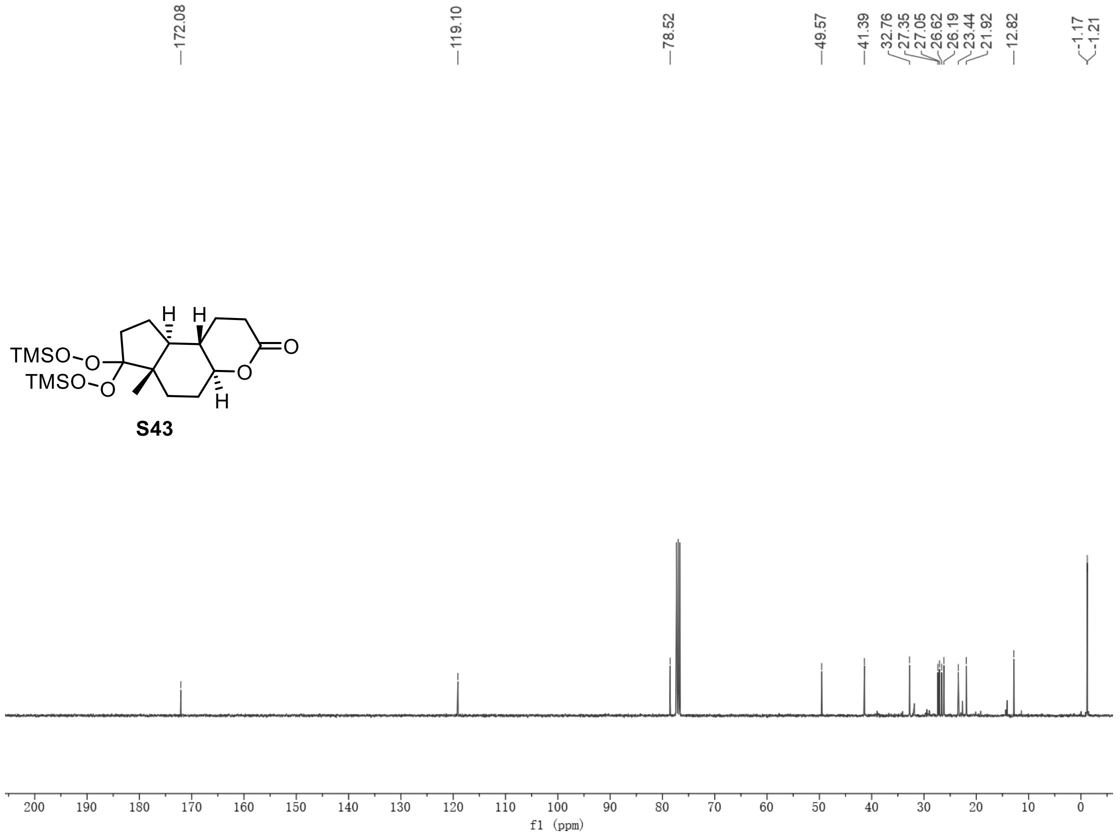

^{1}H NMR (400 MHz, $CDCl_3$) of **S44**

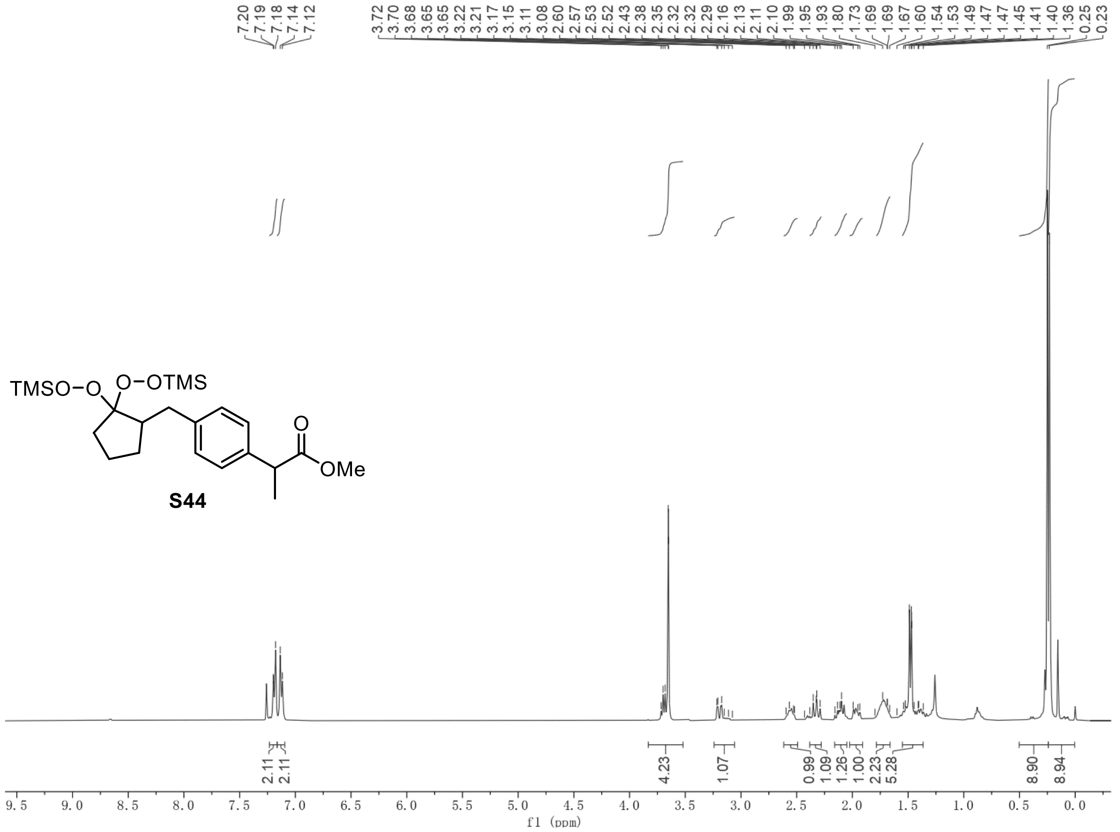

^{13}C NMR (100 MHz, $CDCl_3$) of **S44**

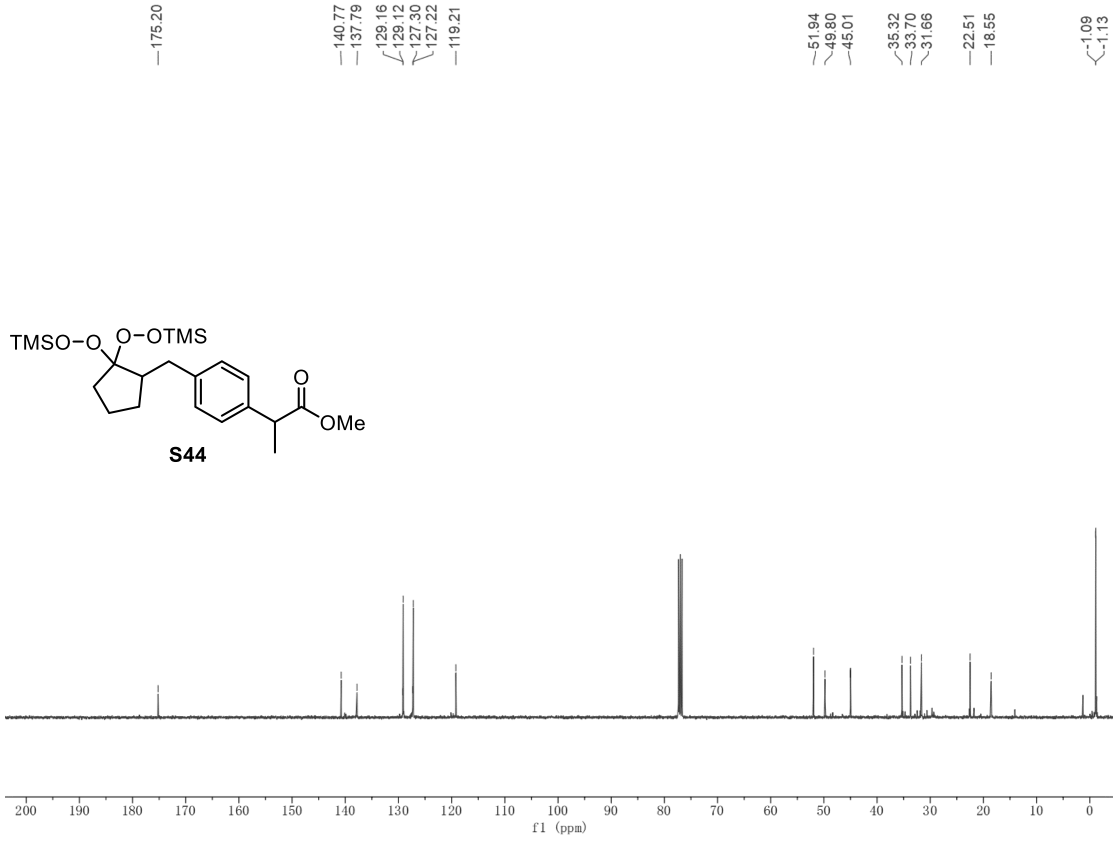

^{1}H NMR (400 MHz, $CDCl_3$) of **S45**

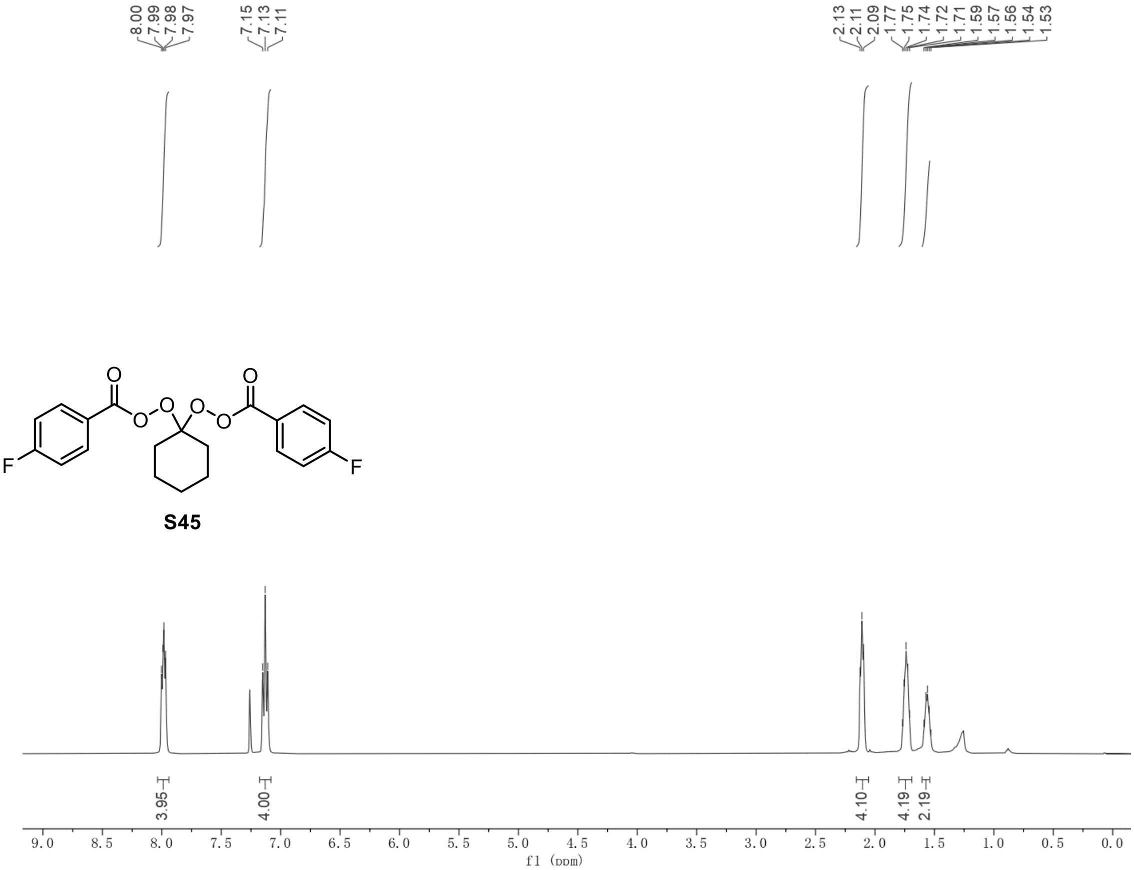

^{19}F NMR (376 MHz, $CDCl_3$) of **S45**

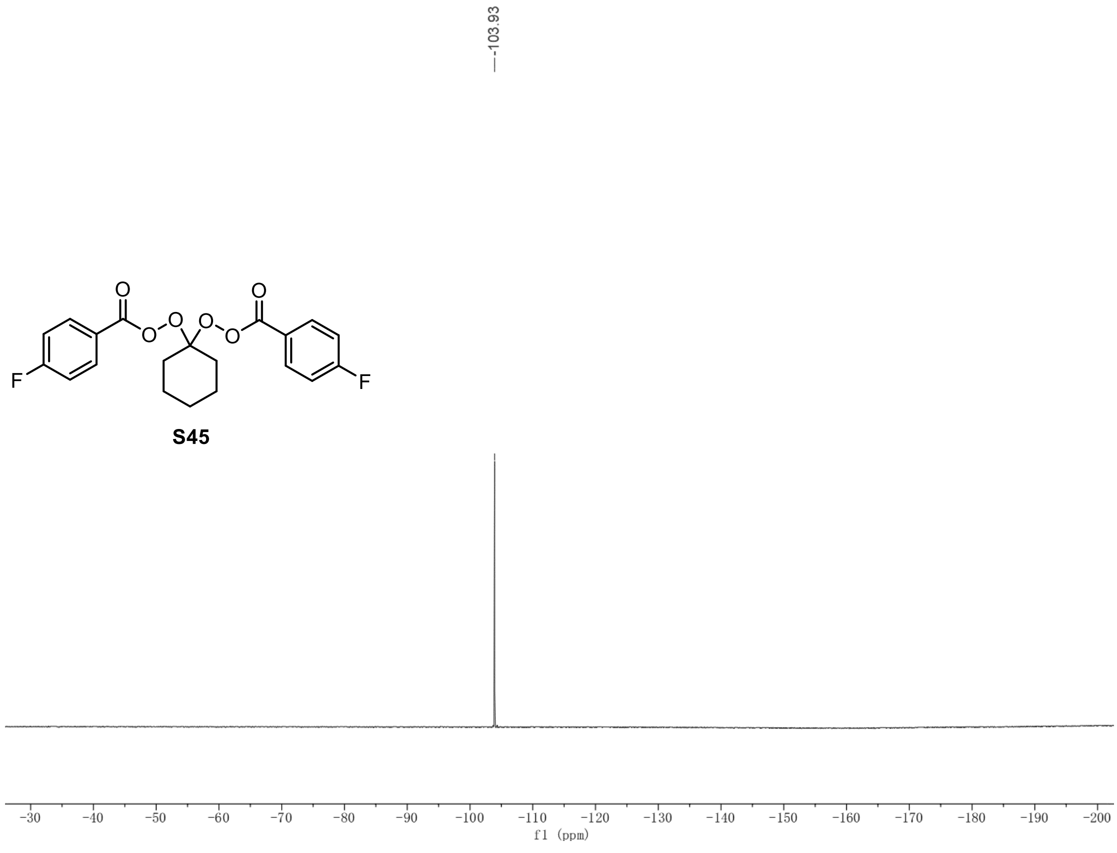

^{13}C NMR (100 MHz, $CDCl_3$) of **S45**

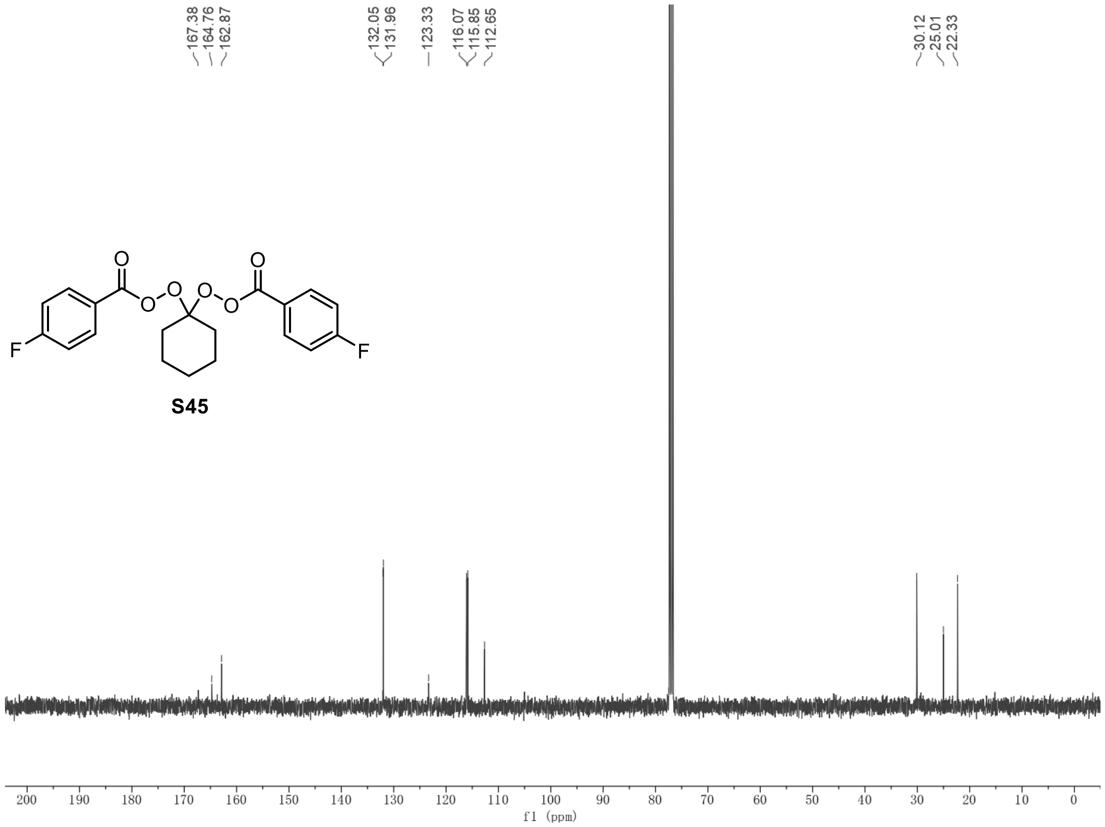

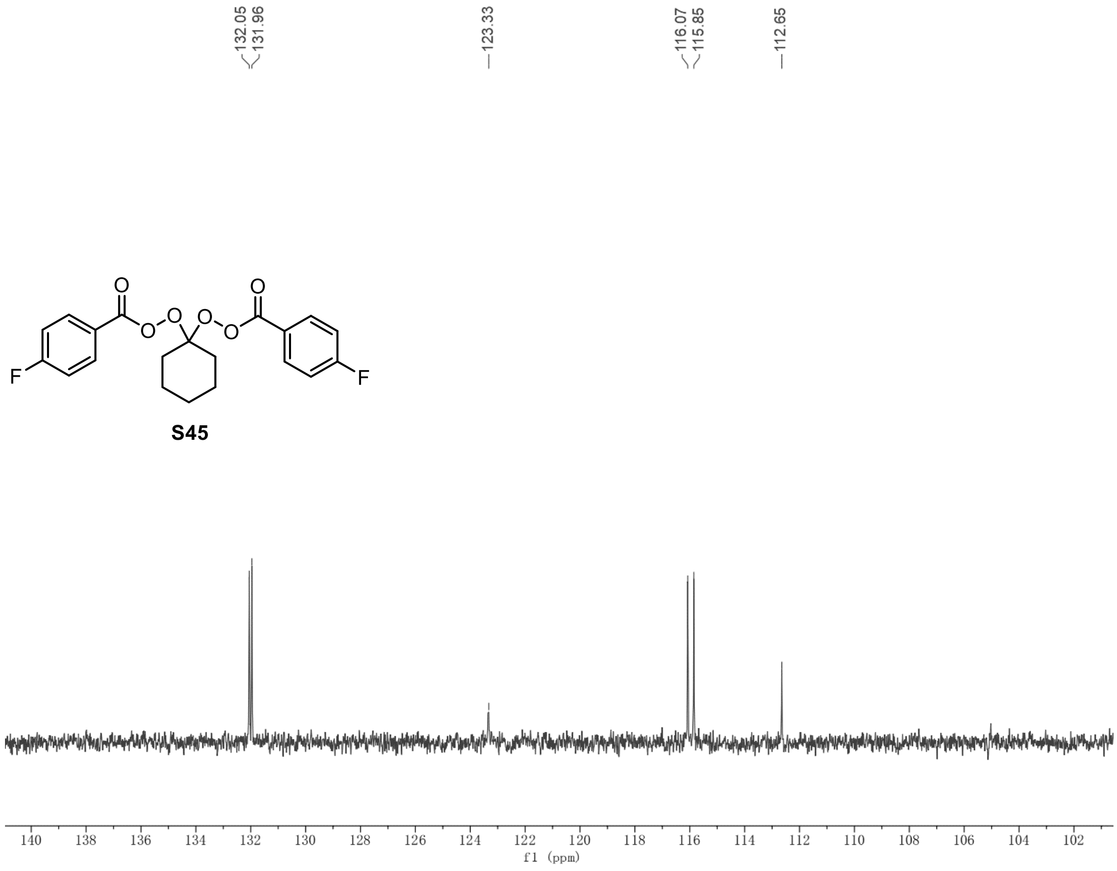

^{1}H NMR (400 MHz, $CDCl_3$) of **S46**

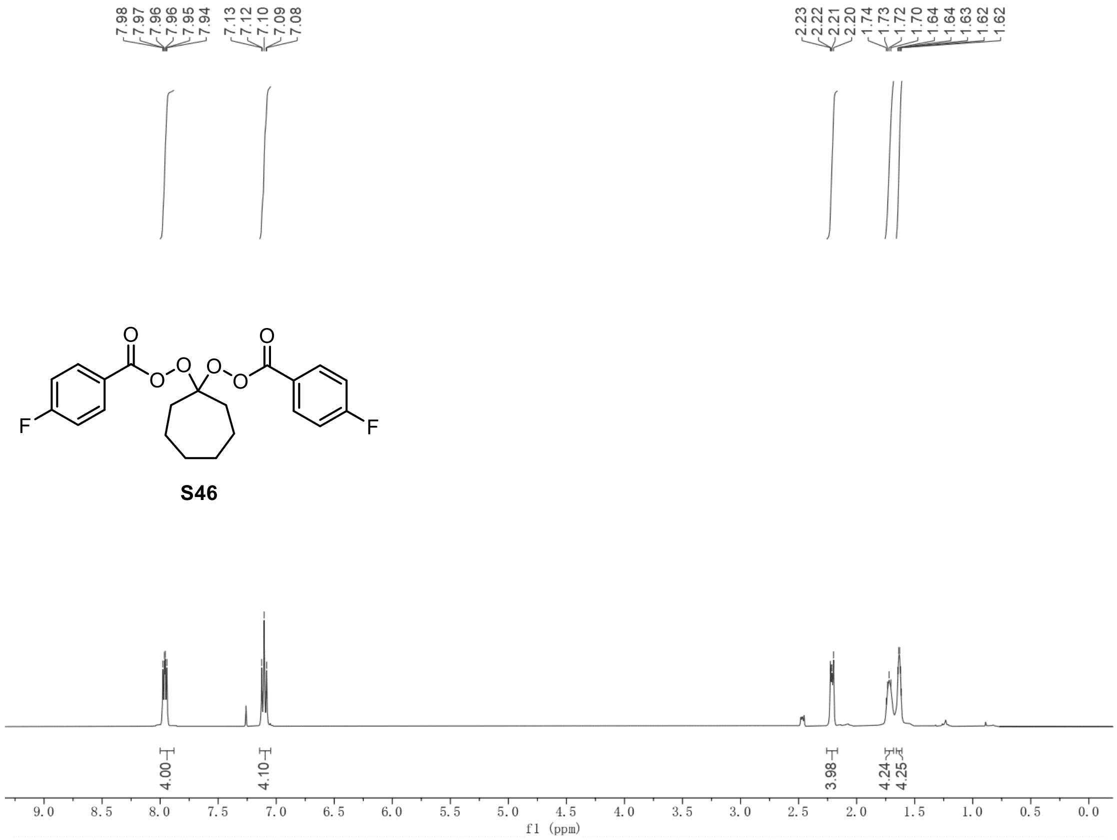

^{19}F NMR (376 MHz, $CDCl_3$) of **S46**

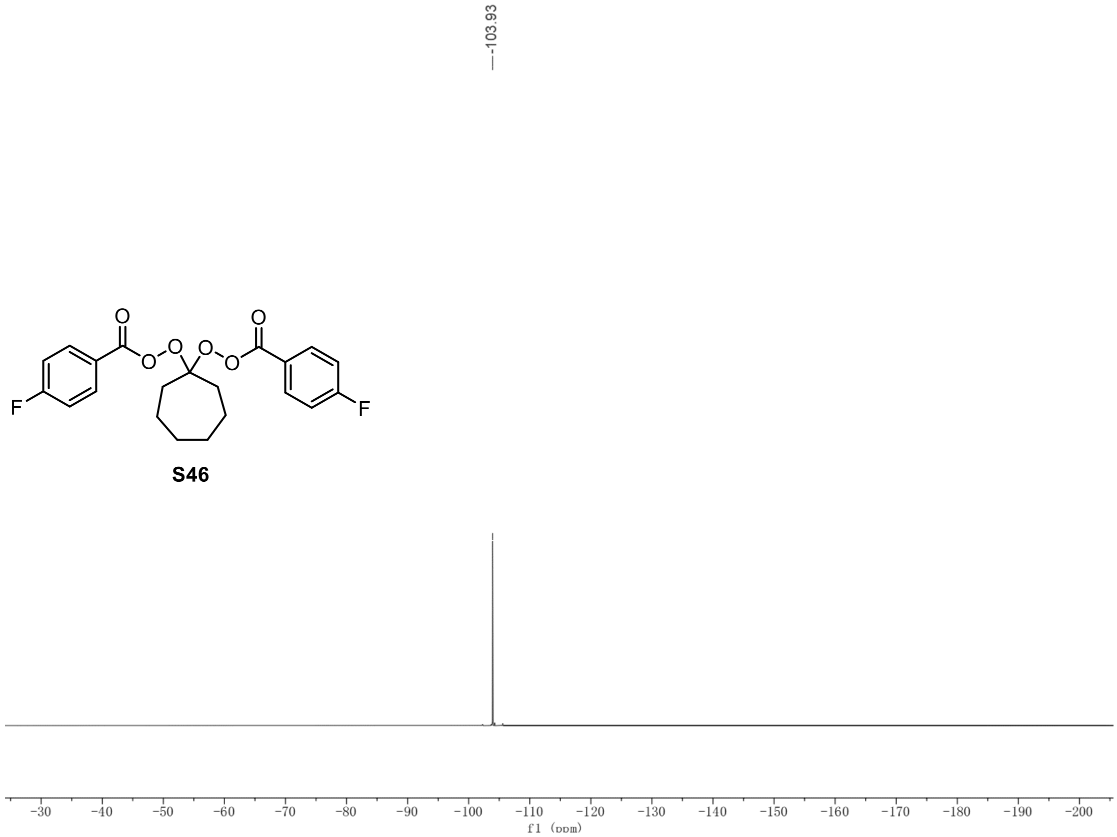

^{13}C NMR (100 MHz, $CDCl_3$) of **S46**

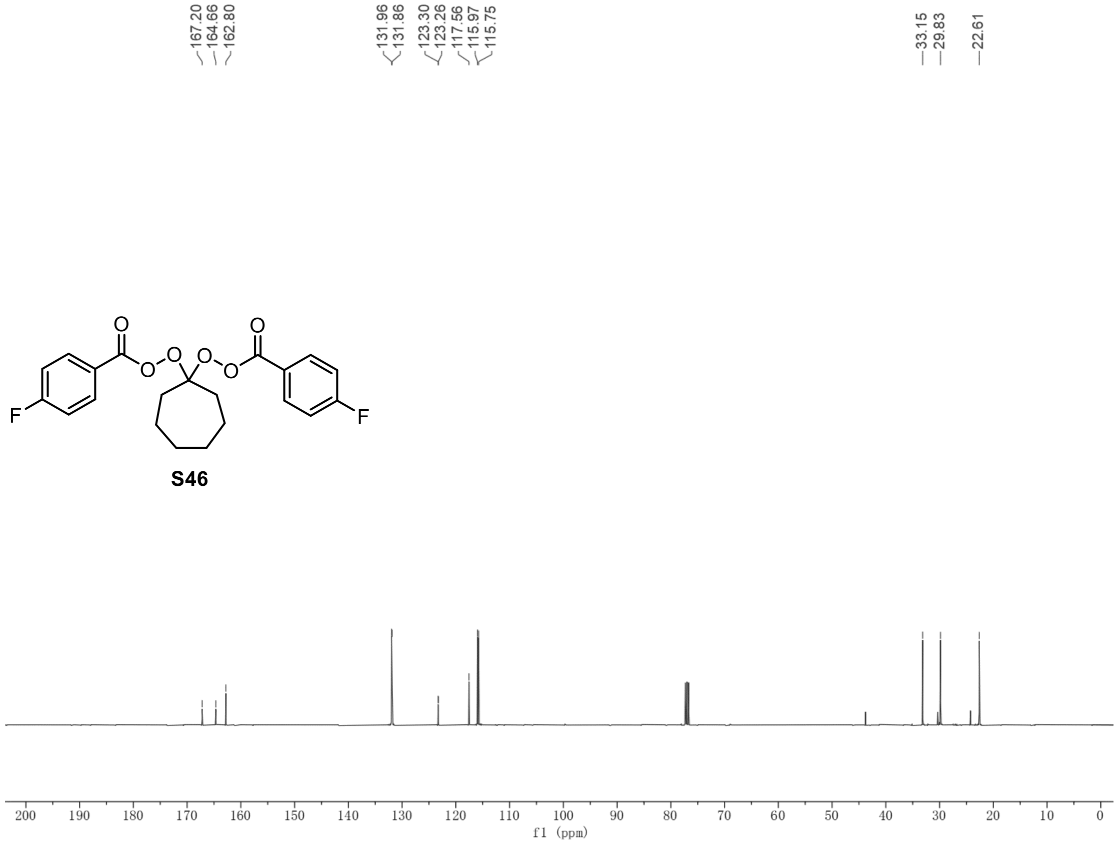

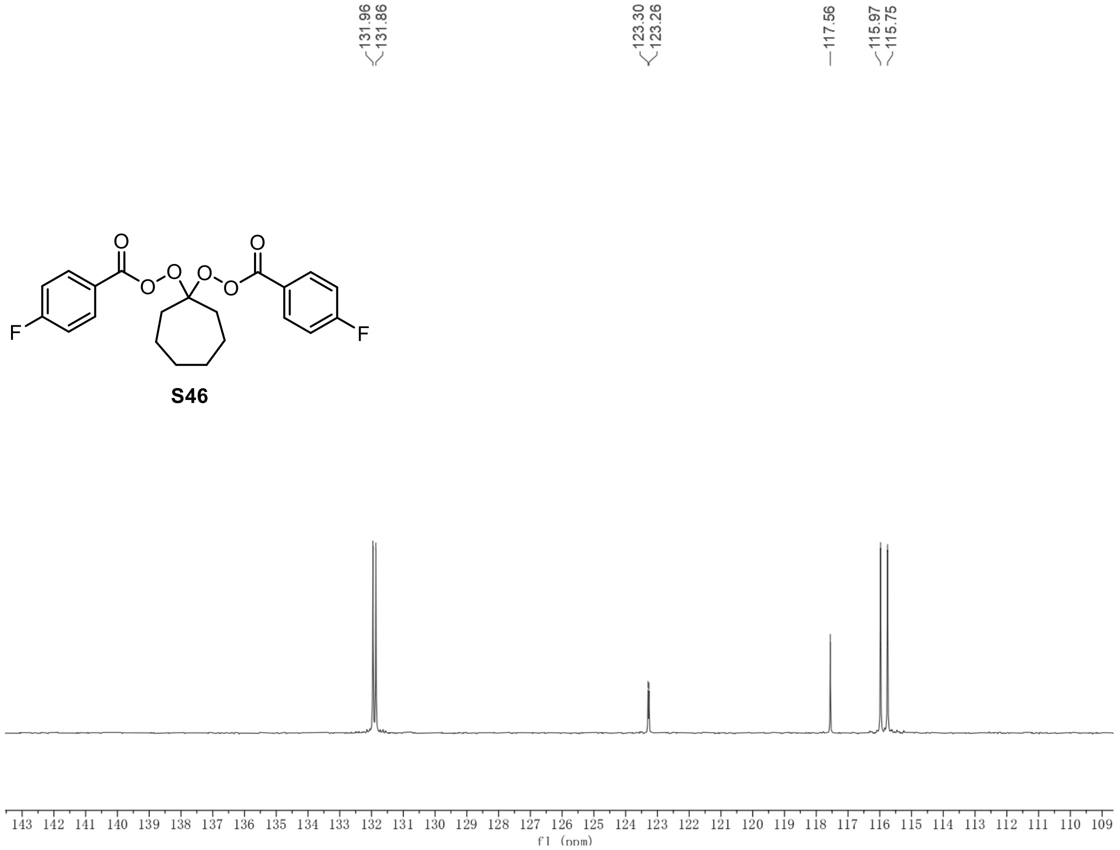

^{1}H NMR (400 MHz, $CDCl_3$) of **S47**

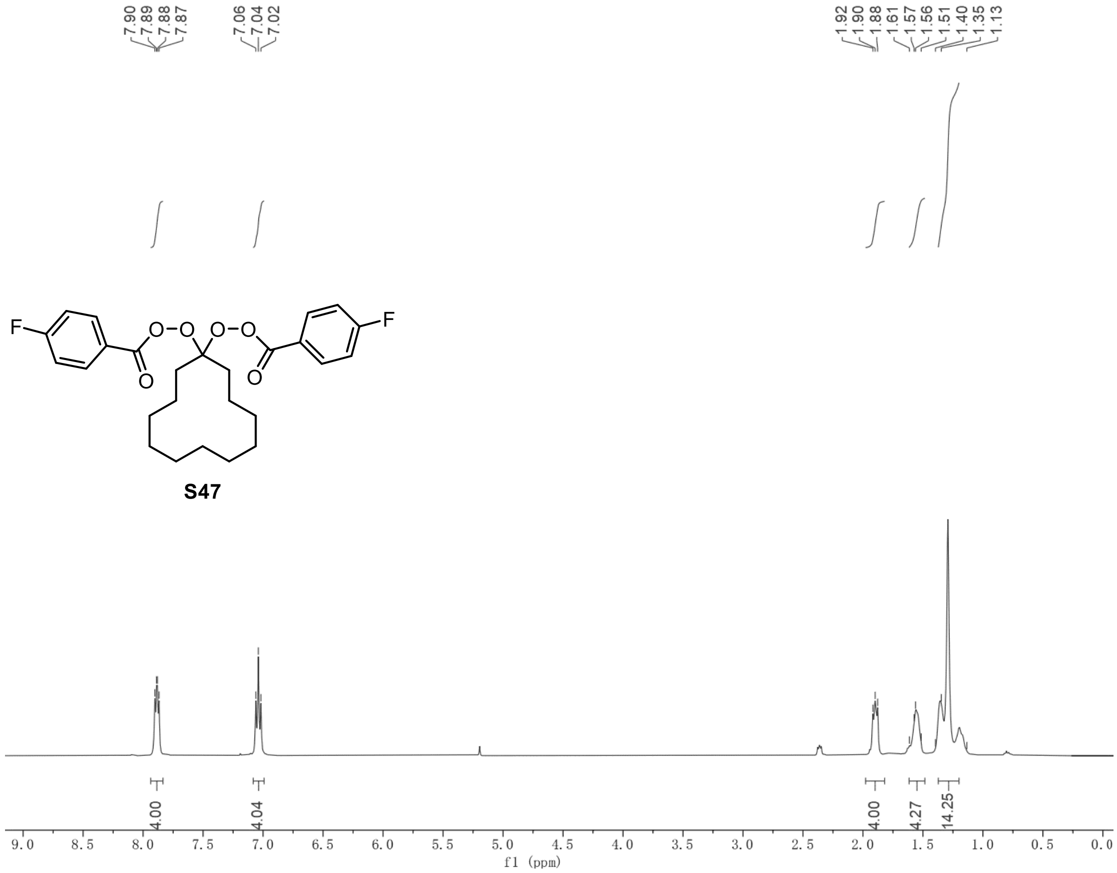

^{19}F NMR (376 MHz, $CDCl_3$) of **S47**

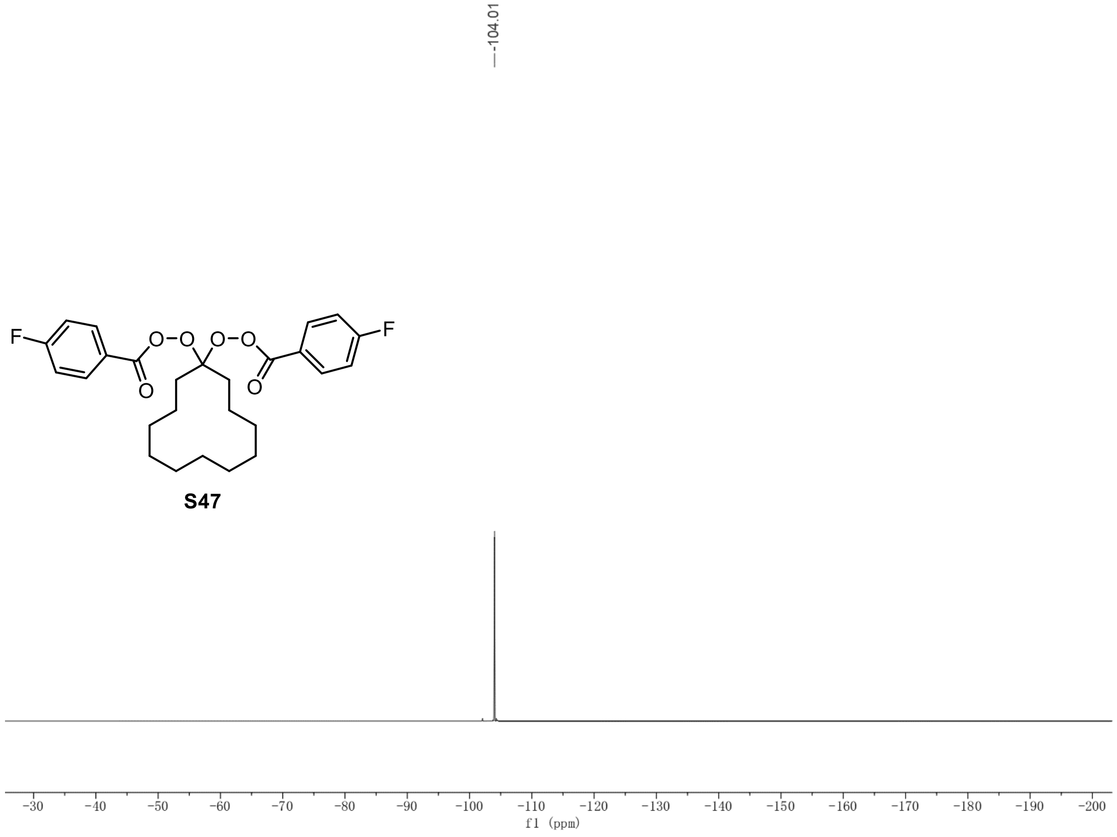

^{13}C NMR (100 MHz, $CDCl_3$) of **S47**

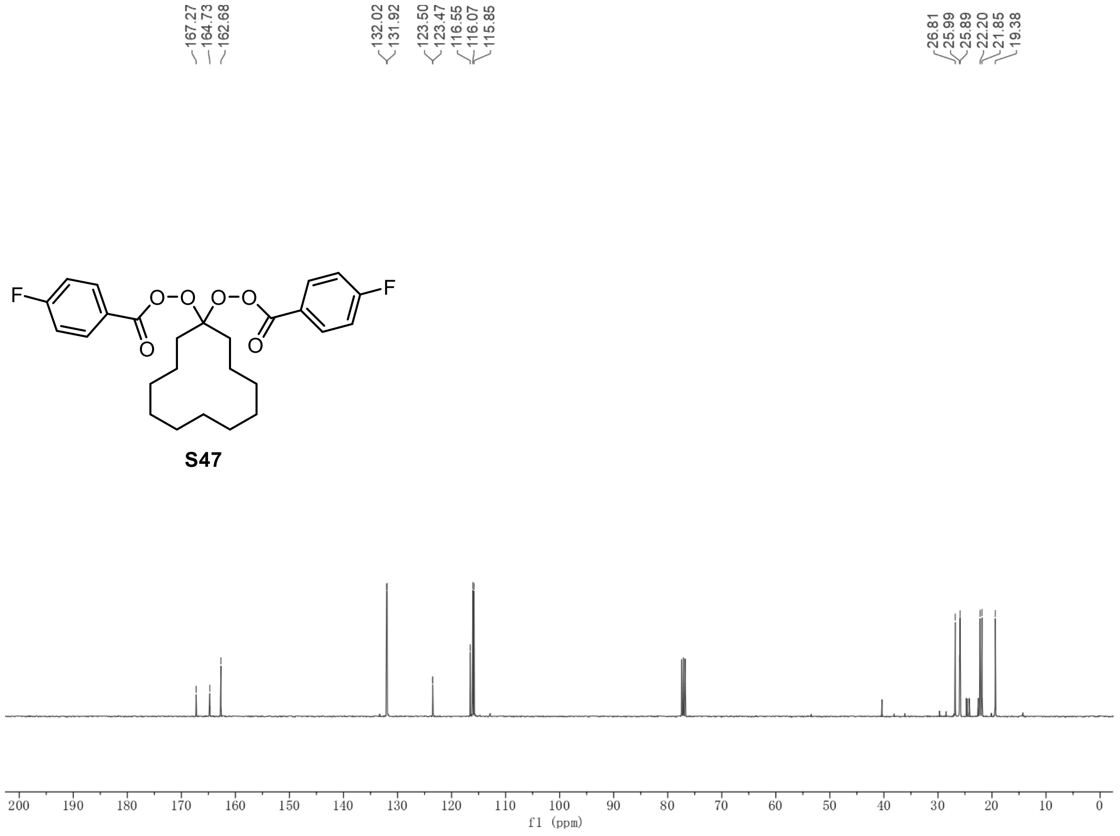

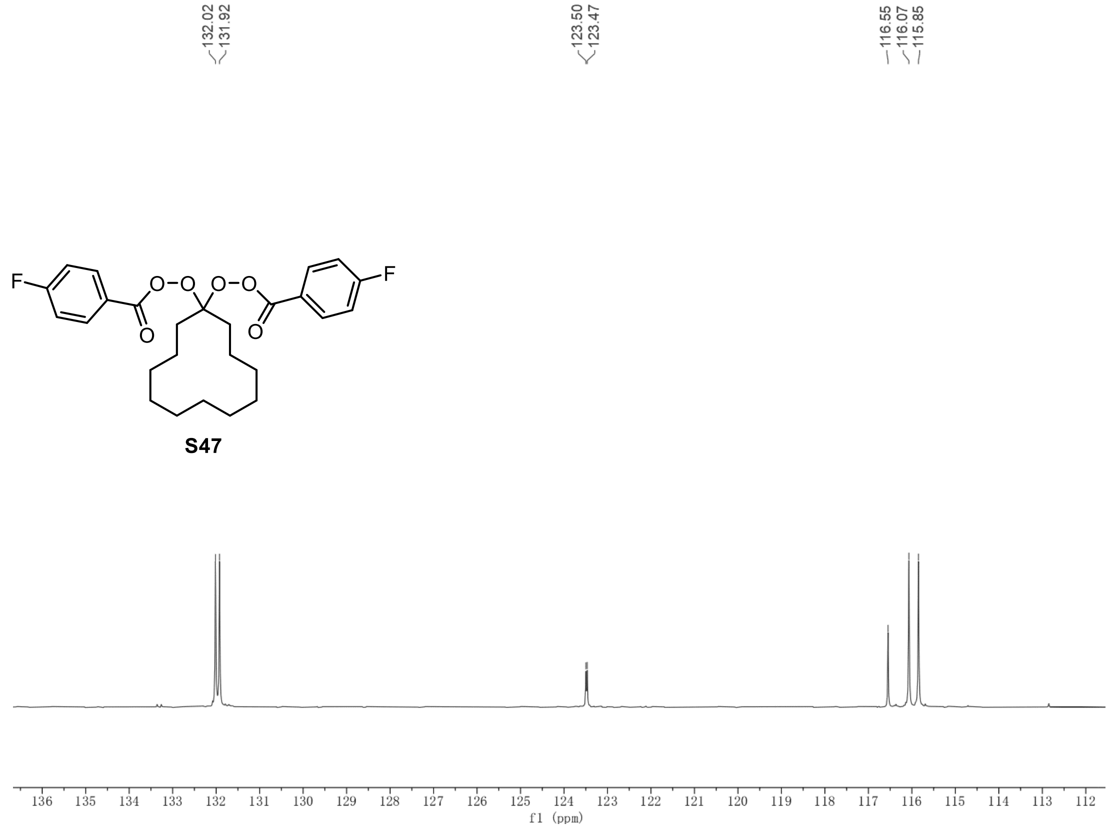

^{1}H NMR (400 MHz, CDCl$_3$) of **S48**

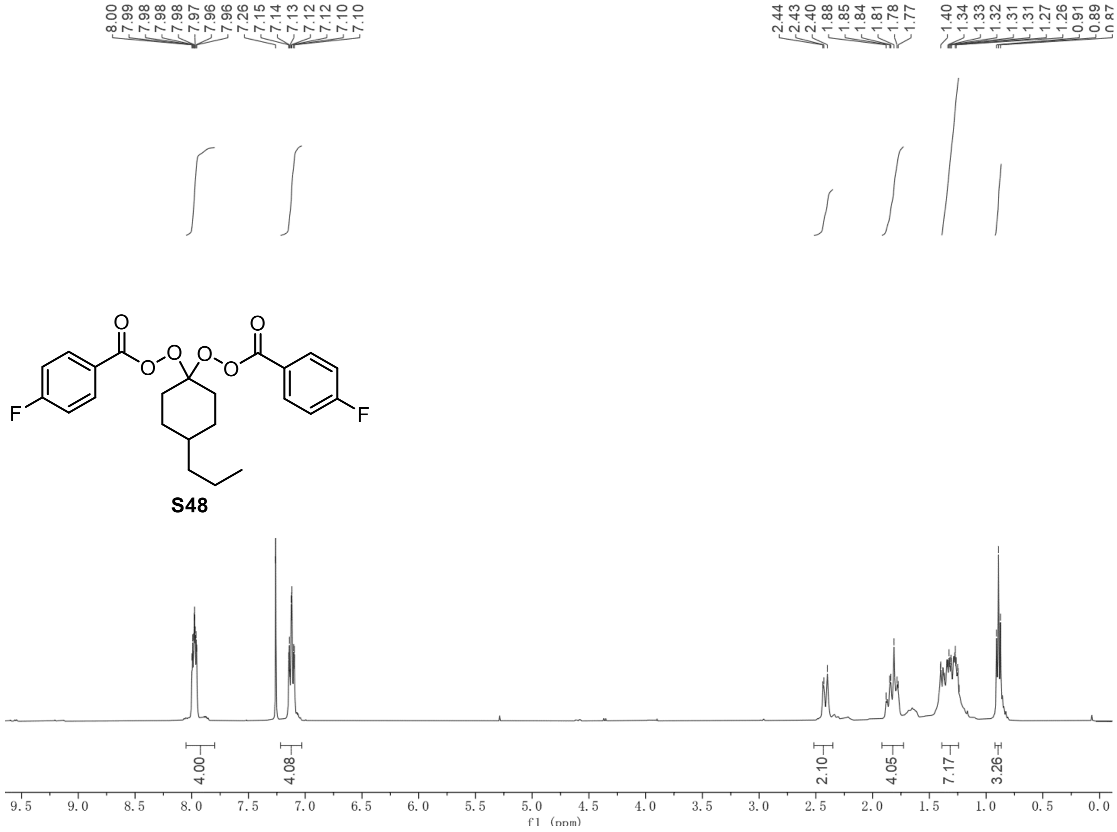

^{19}F NMR (376 MHz, CDCl$_3$) of **S48**

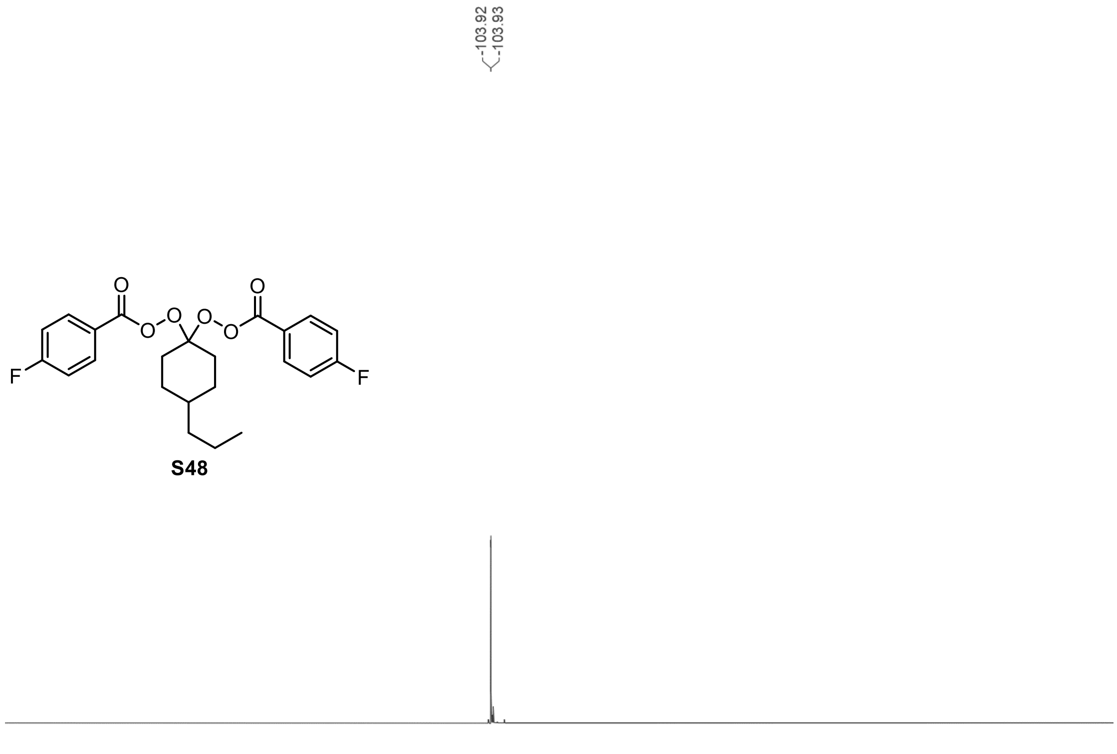

^{13}**C NMR** (100 MHz, $CDCl_3$) of **S48**

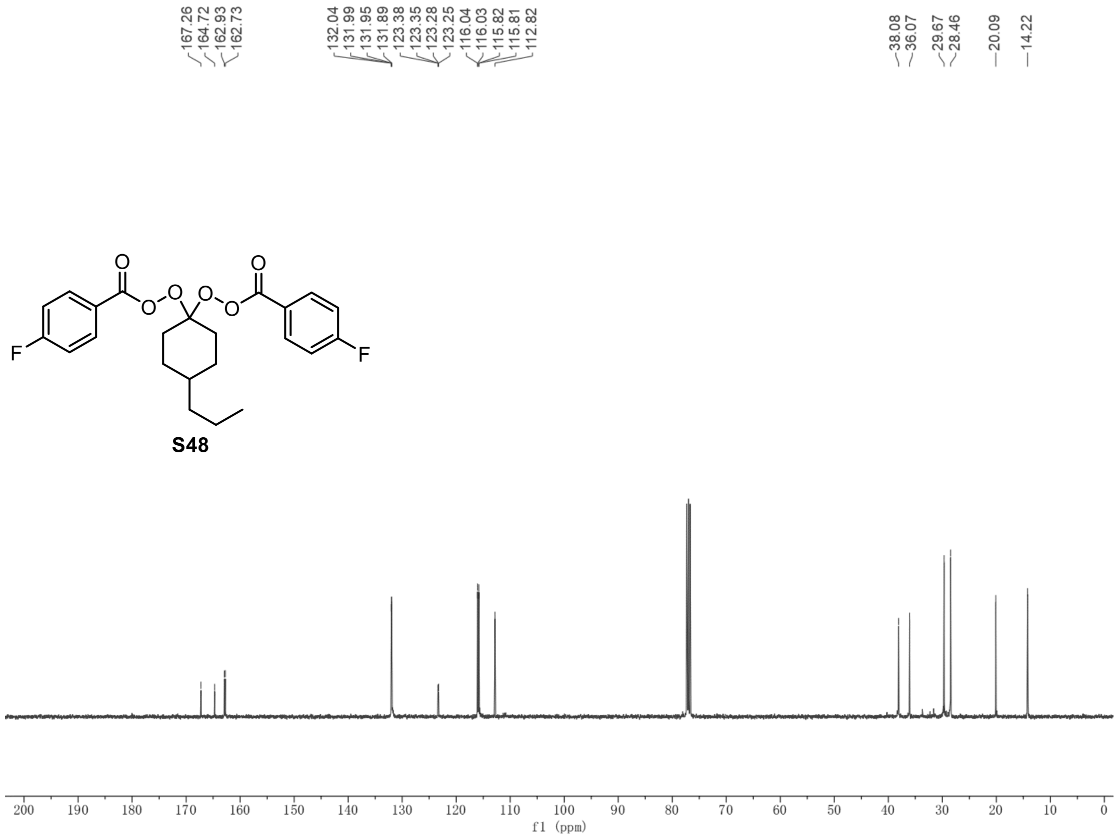

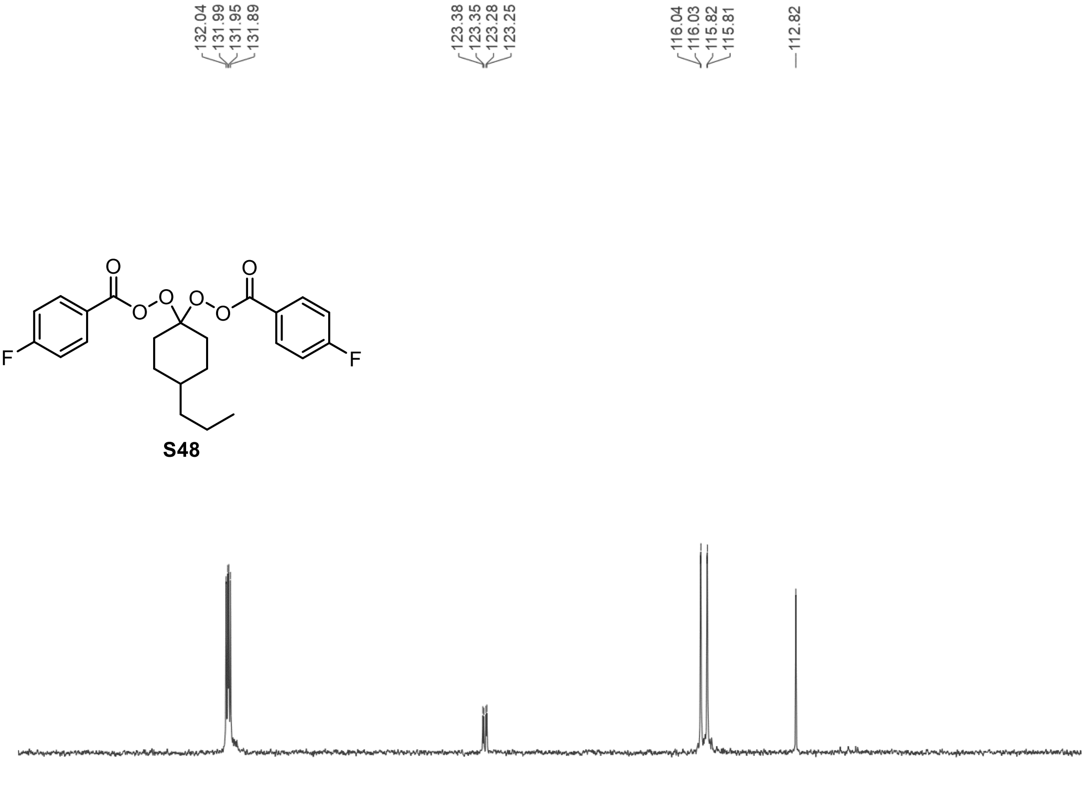

^{1}H NMR (400 MHz, $CDCl_3$) of **S49**

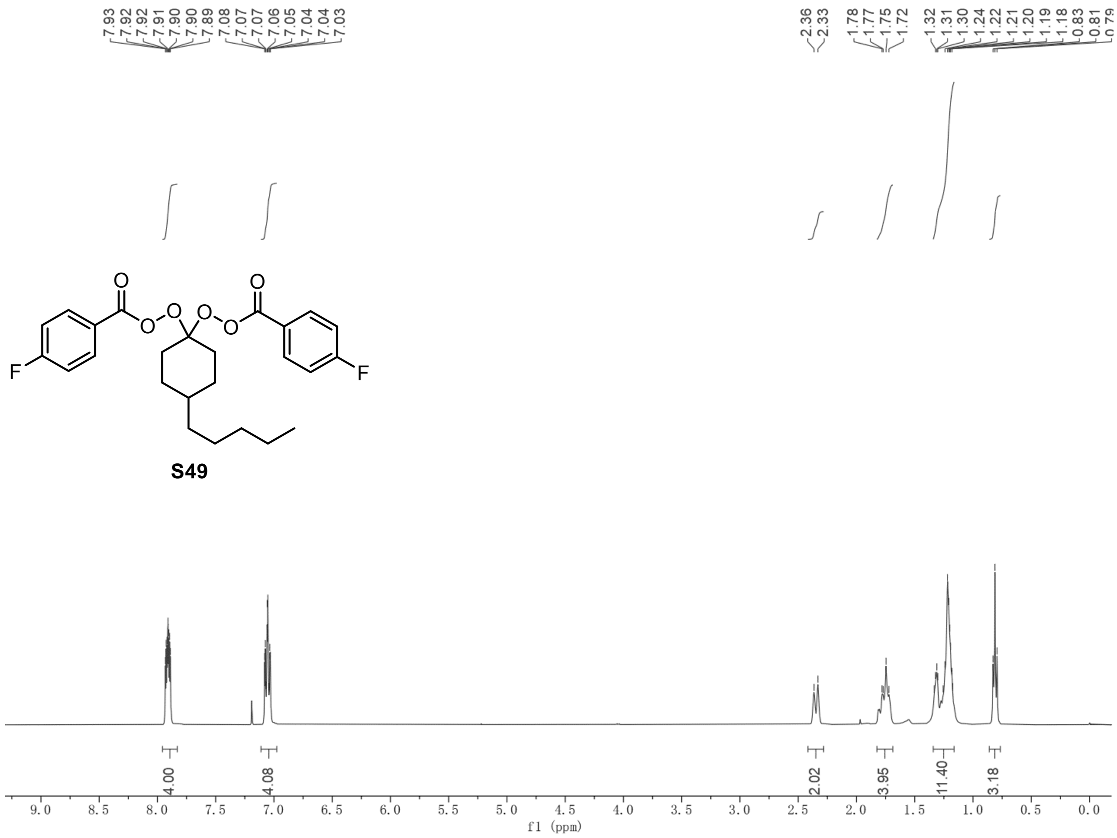

^{19}F NMR (376 MHz, $CDCl_3$) of **S49**

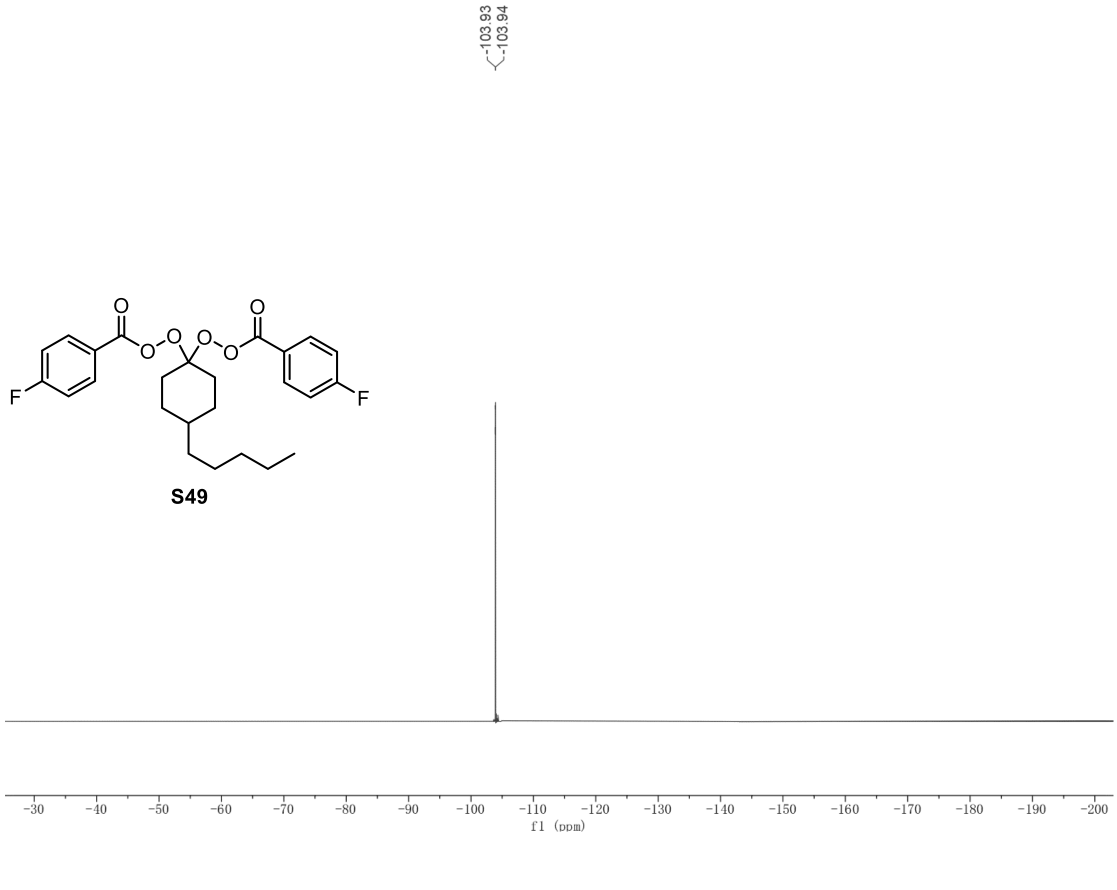

^{13}C NMR (100 MHz, CDCl$_3$) of **S49**

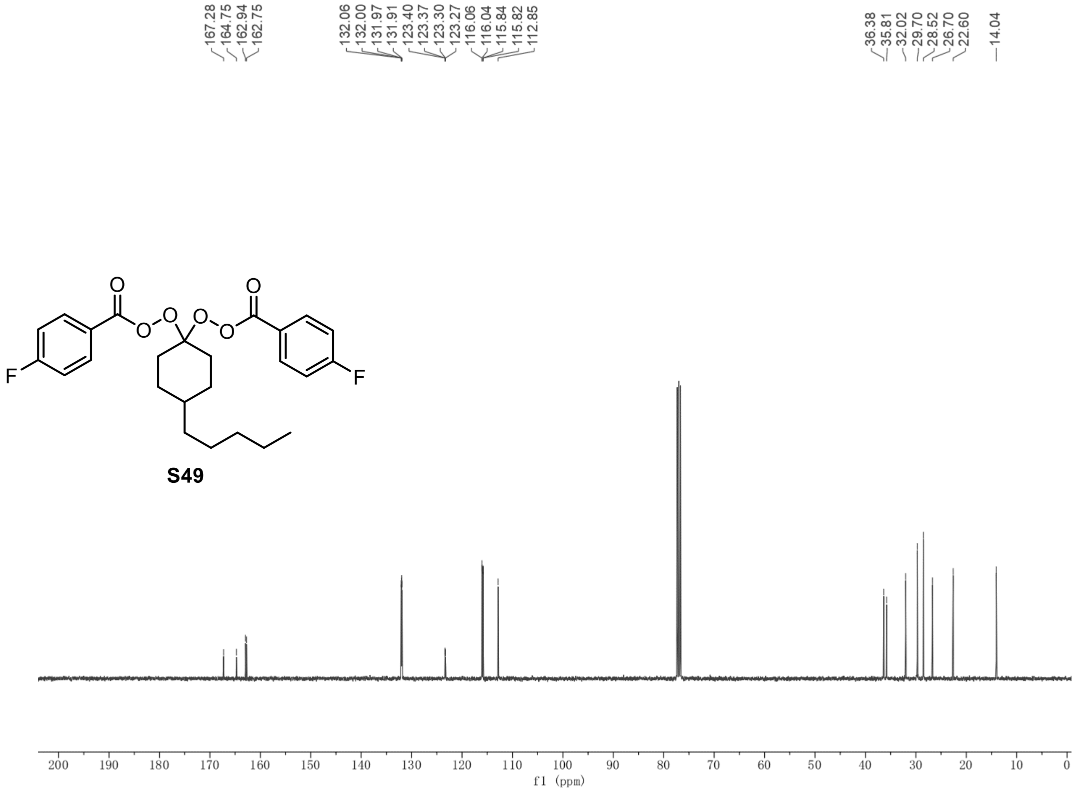

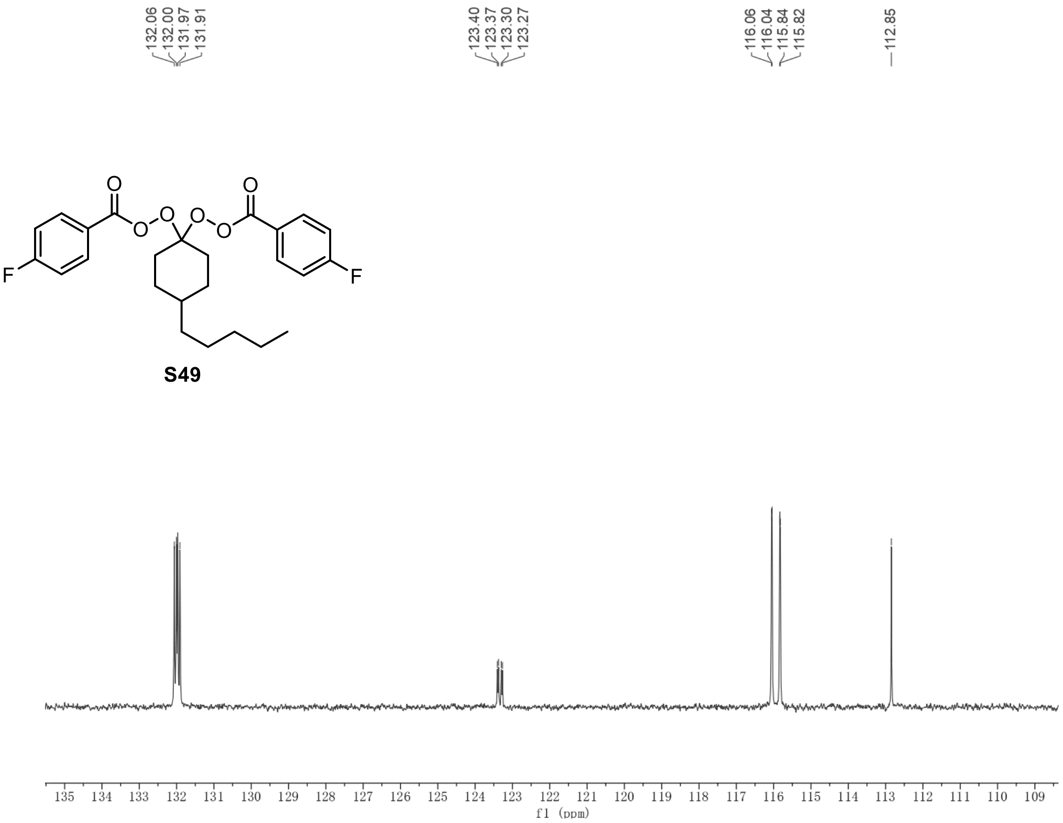

^{1}H NMR (400 MHz, $CDCl_3$) of **S50**

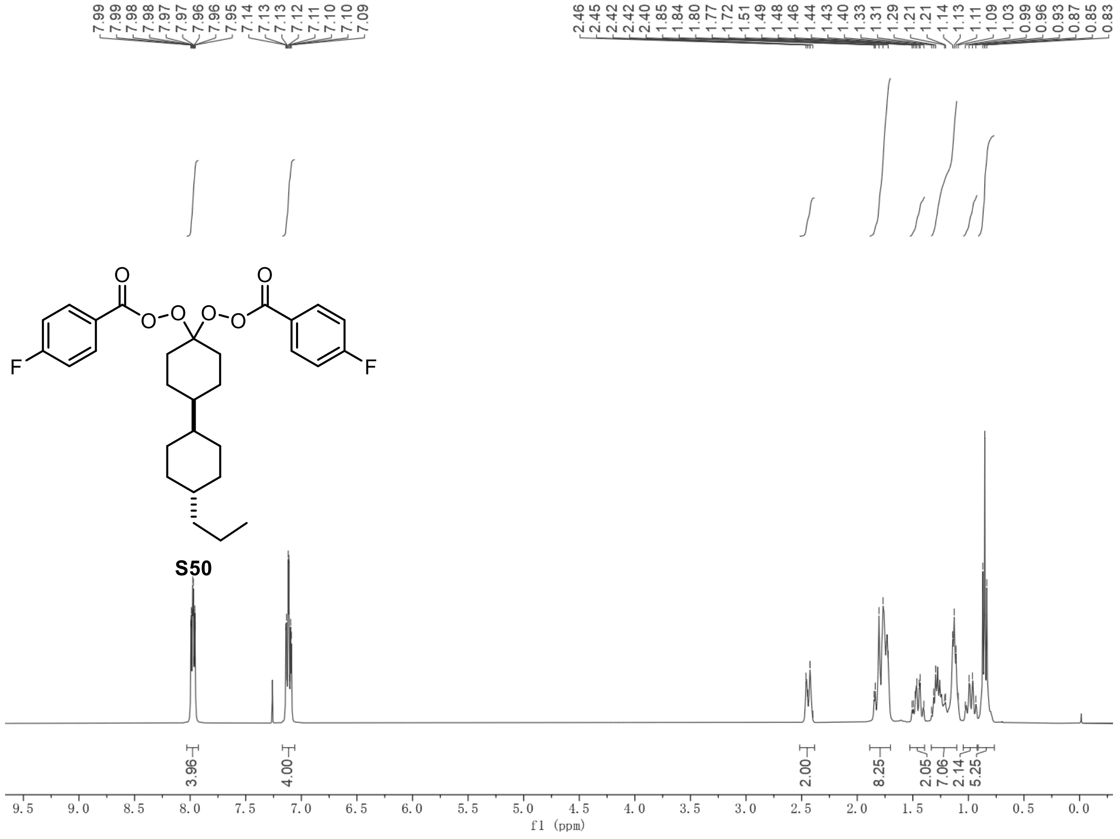

^{19}F NMR (376 MHz, $CDCl_3$) of **S50**

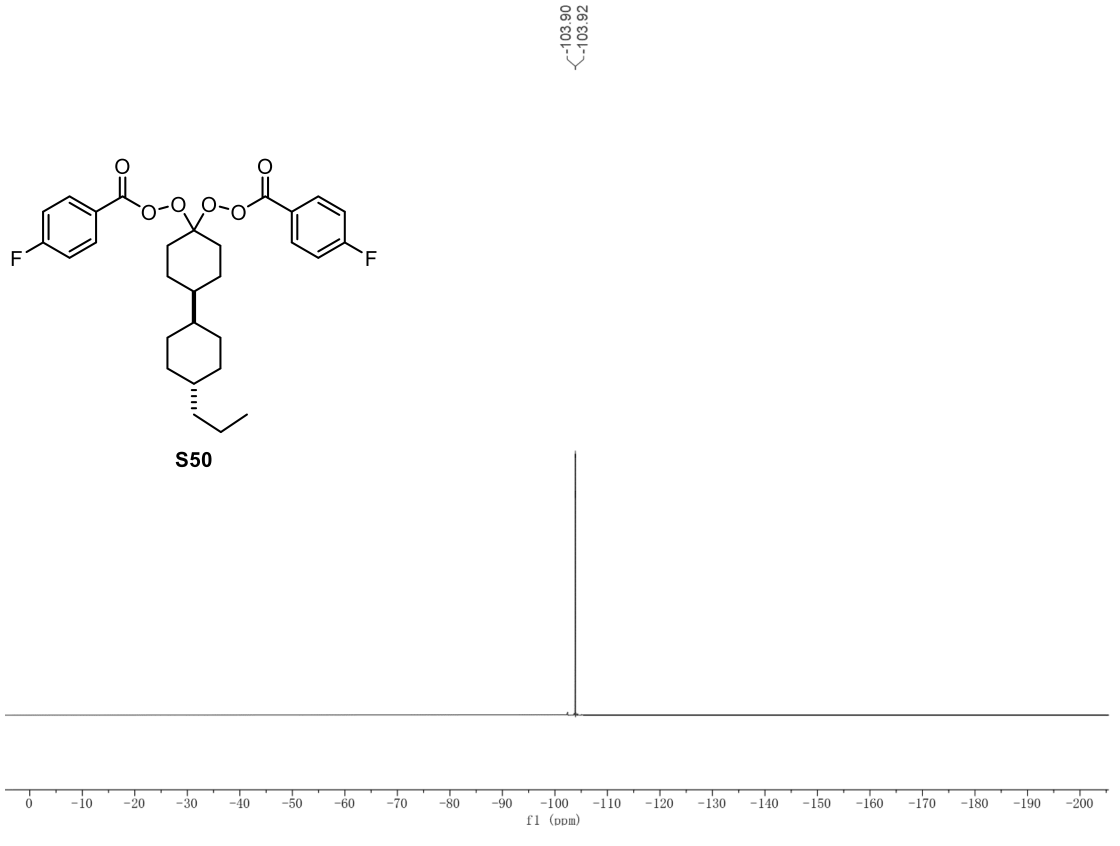

^{13}C NMR (100 MHz, $CDCl_3$) of **S50**

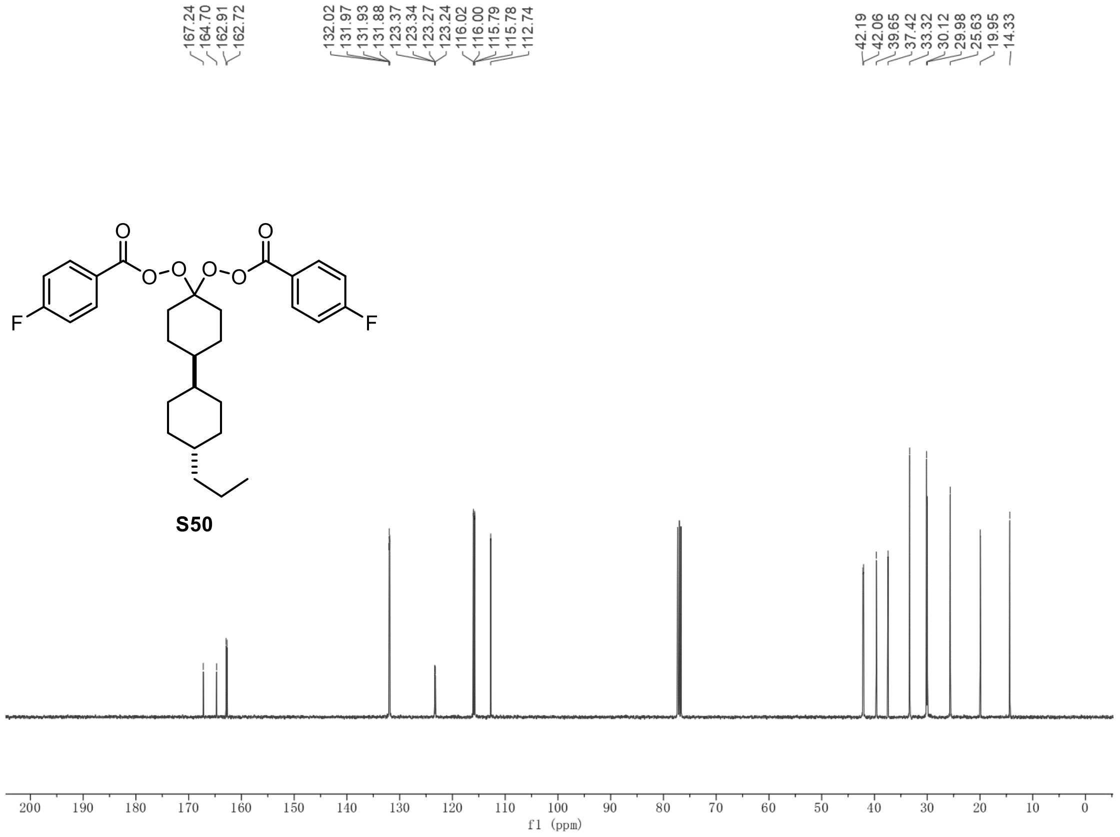

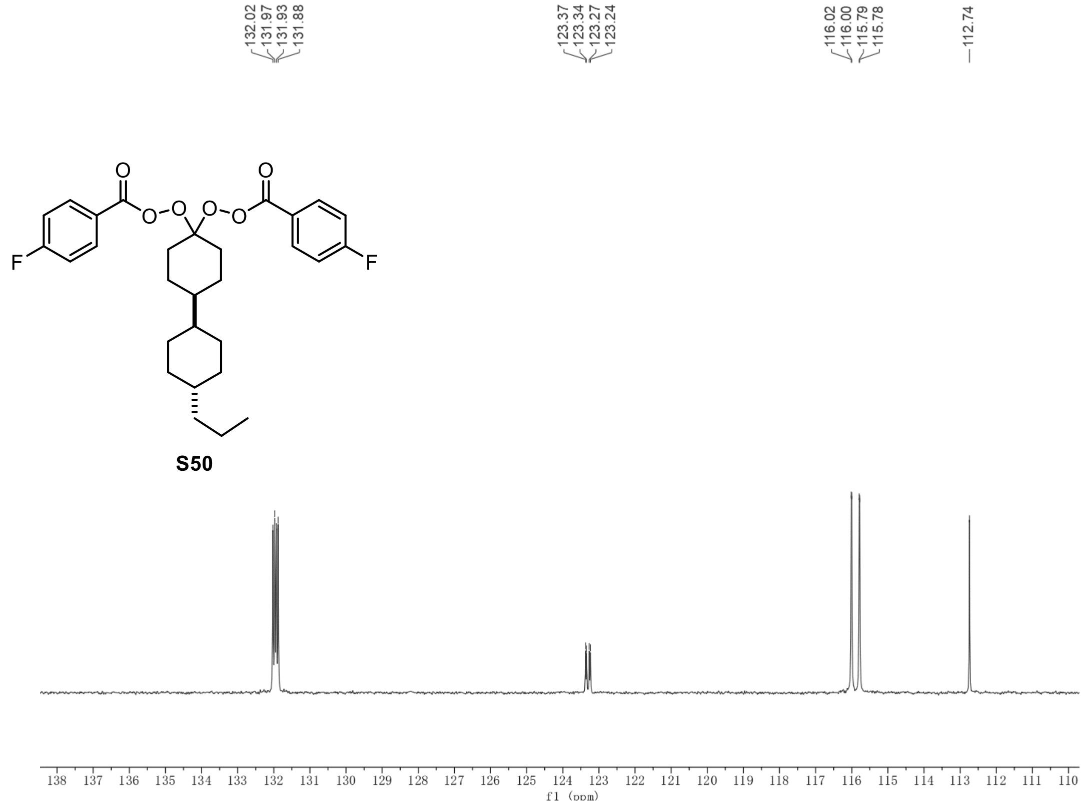

^{1}H NMR (400 MHz, $CDCl_3$) of **S51**

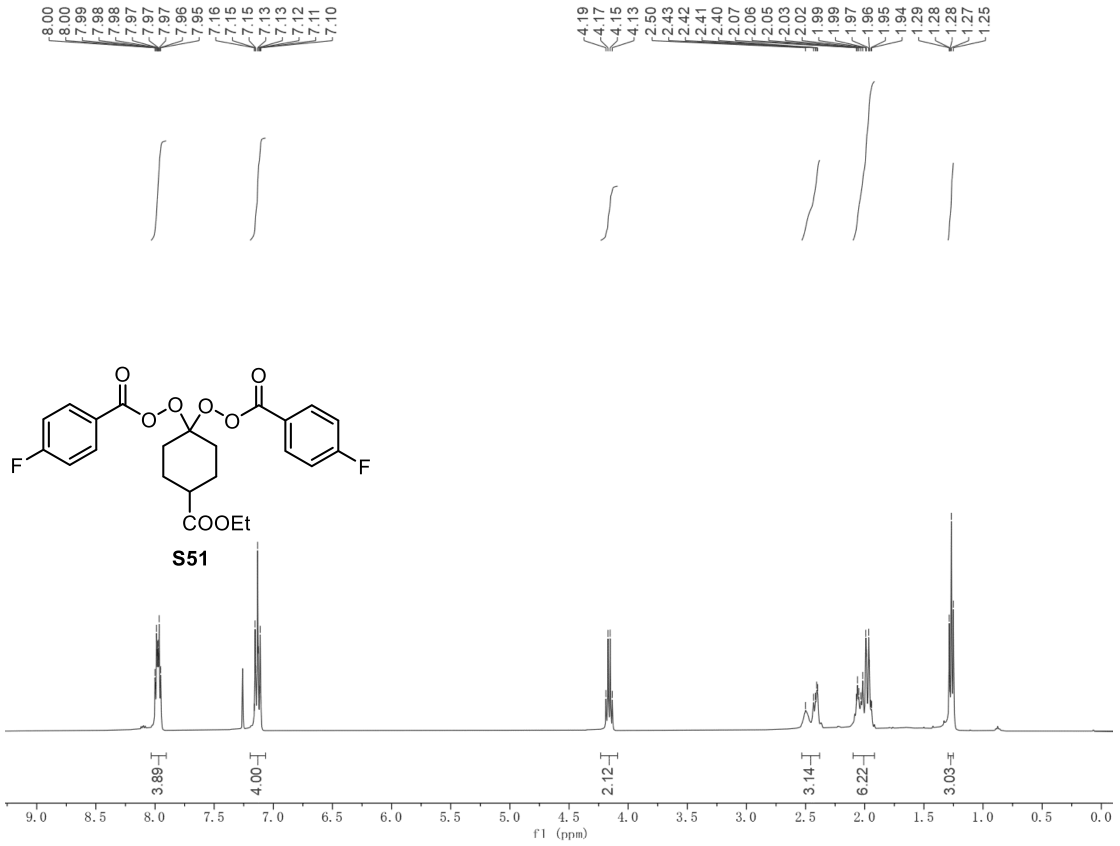

S51

^{19}F NMR (376 MHz, $CDCl_3$) of **S51**

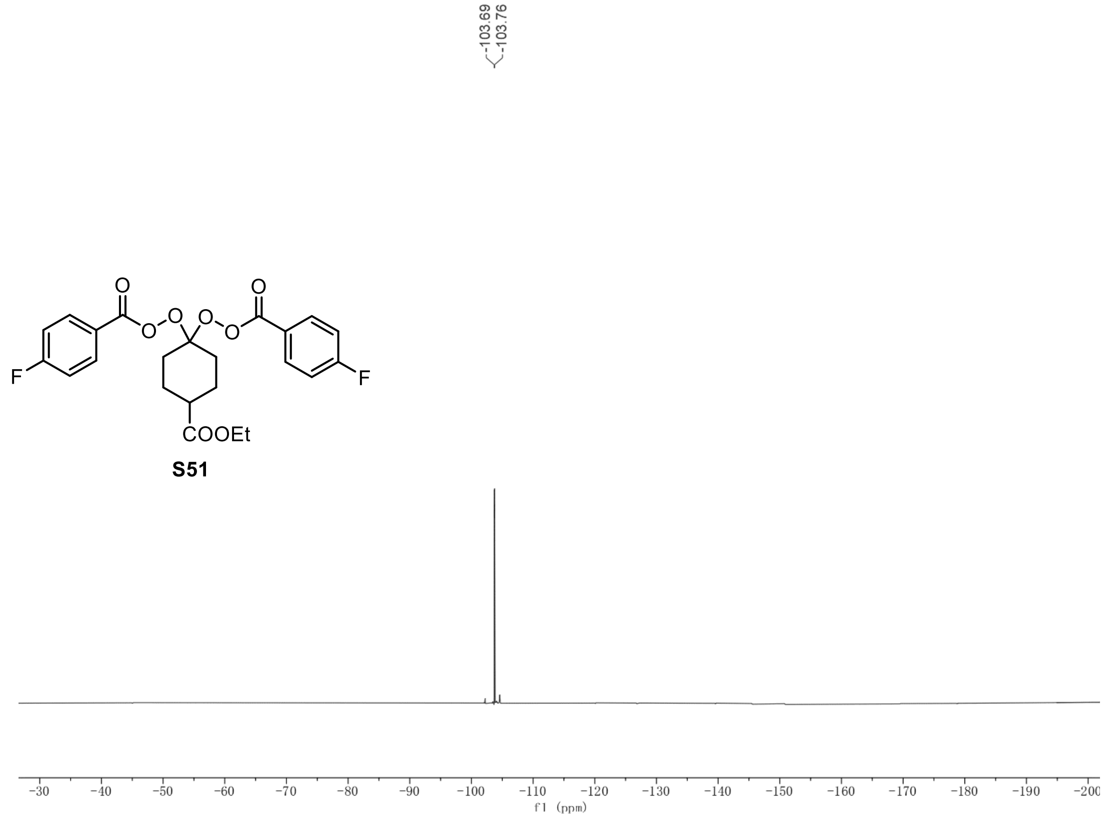

S51

^{13}C NMR (100 MHz, $CDCl_3$) of **S51**

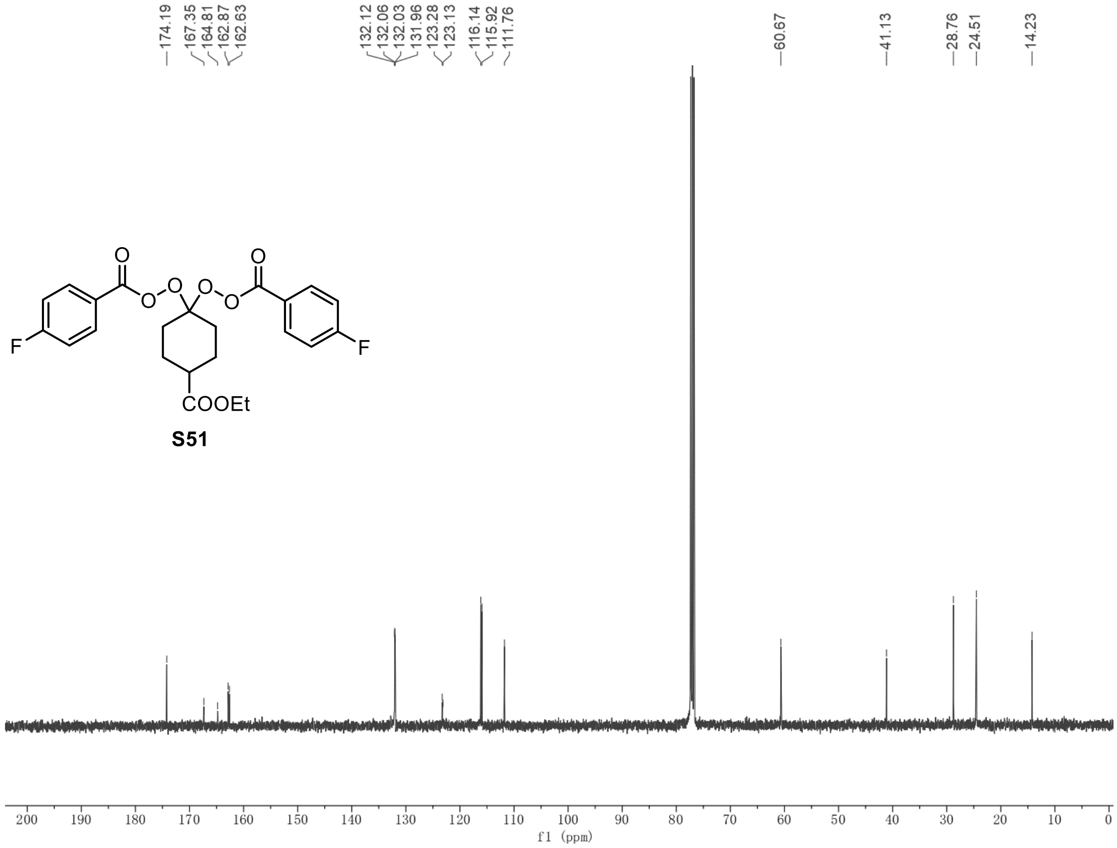

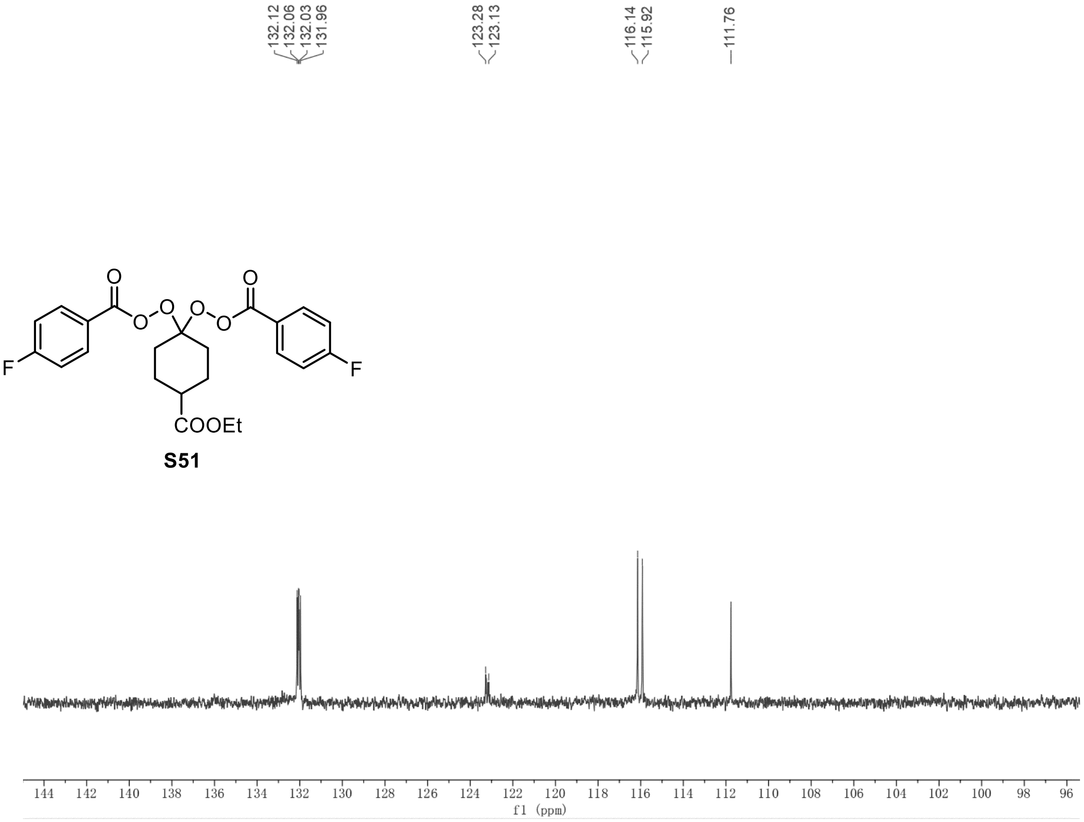

^{1}H NMR (400 MHz, $CDCl_3$) of **S52**

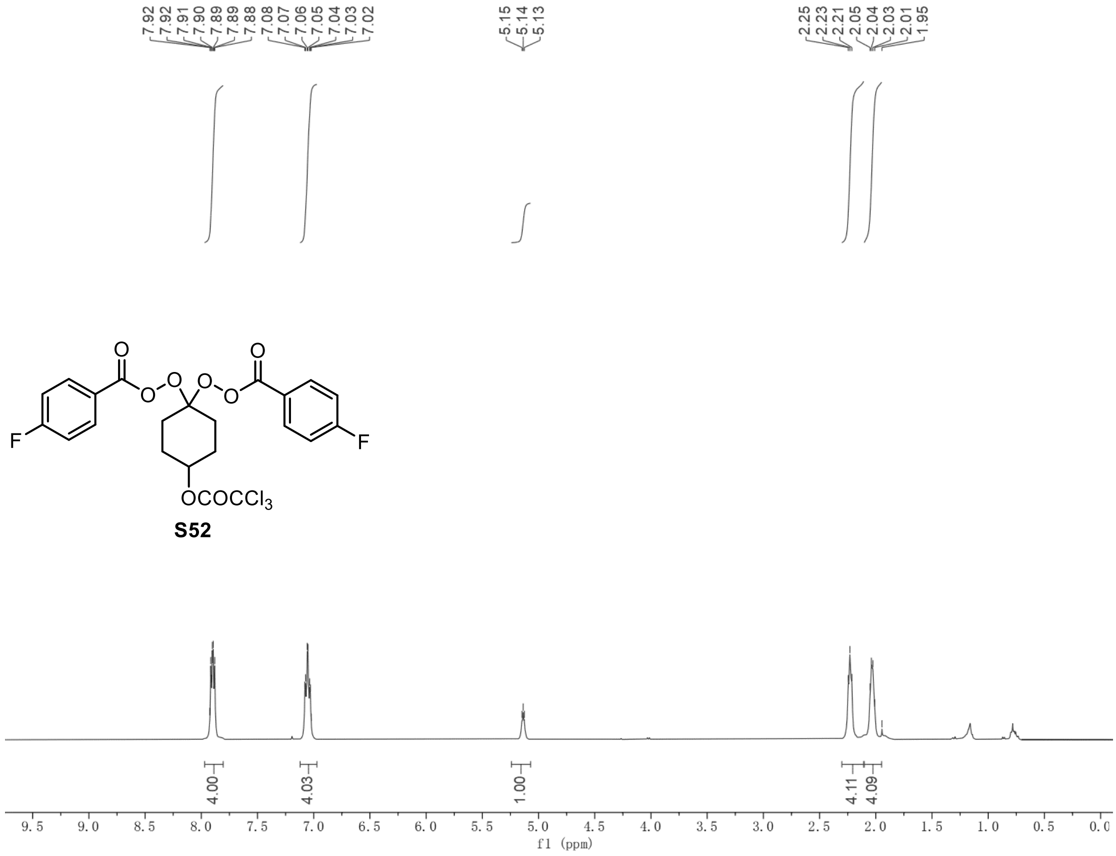

^{19}F NMR (376 MHz, $CDCl_3$) of **S52**

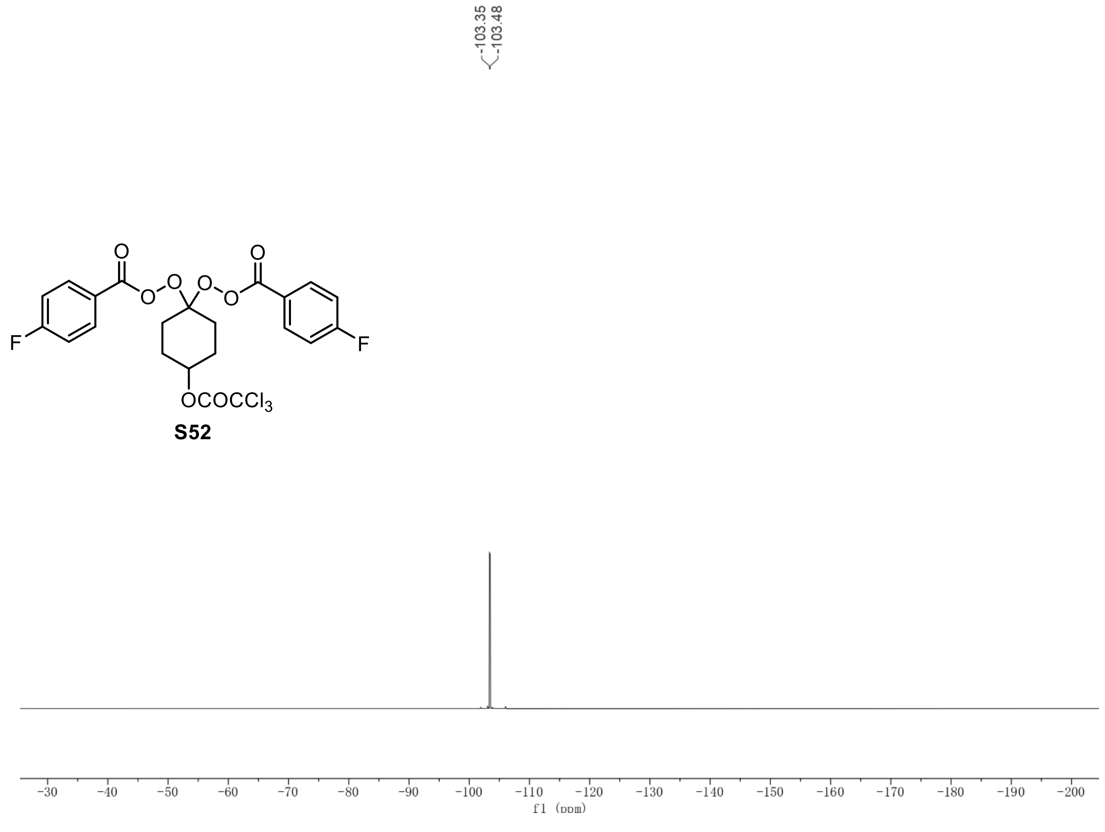

^{13}**C NMR** (100 MHz, $CDCl_3$) of **S52**

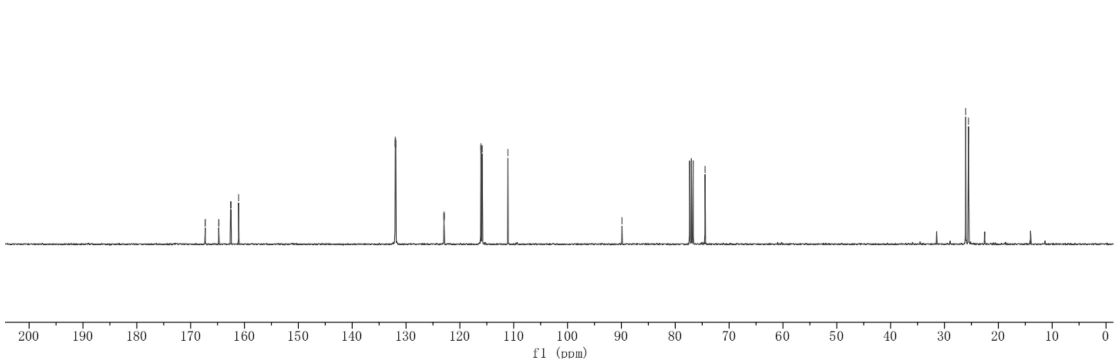

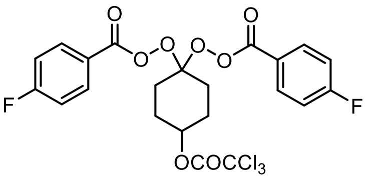

S52

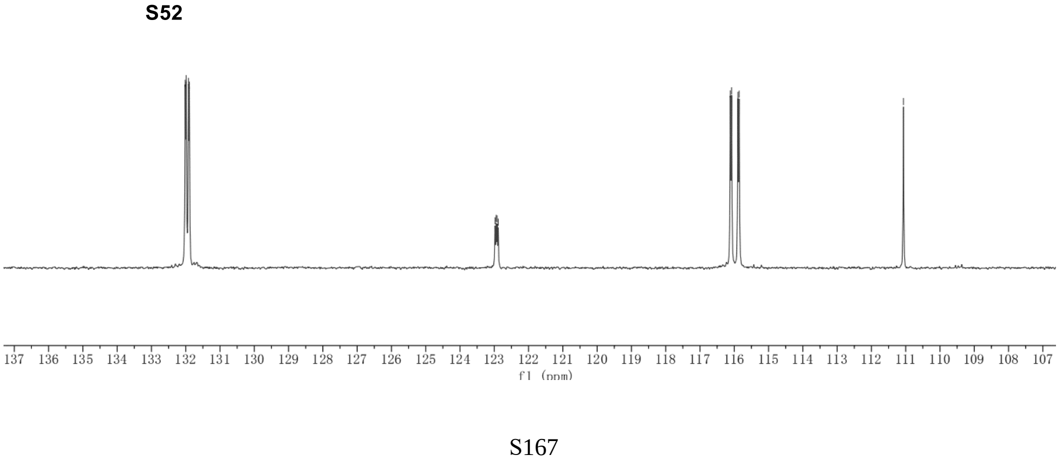

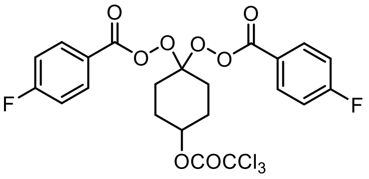

S52

^{1}H NMR (400 MHz, $CDCl_3$) of **S53**

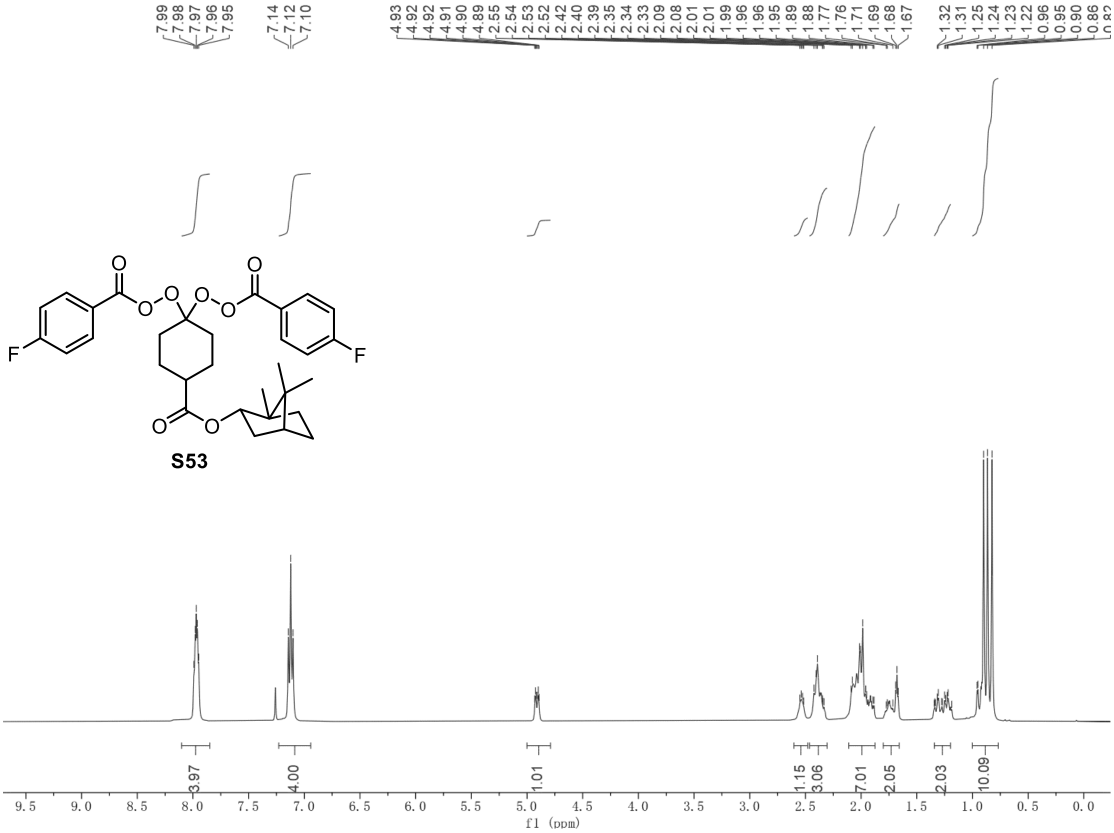

^{19}F NMR (376 MHz, $CDCl_3$) of **S53**

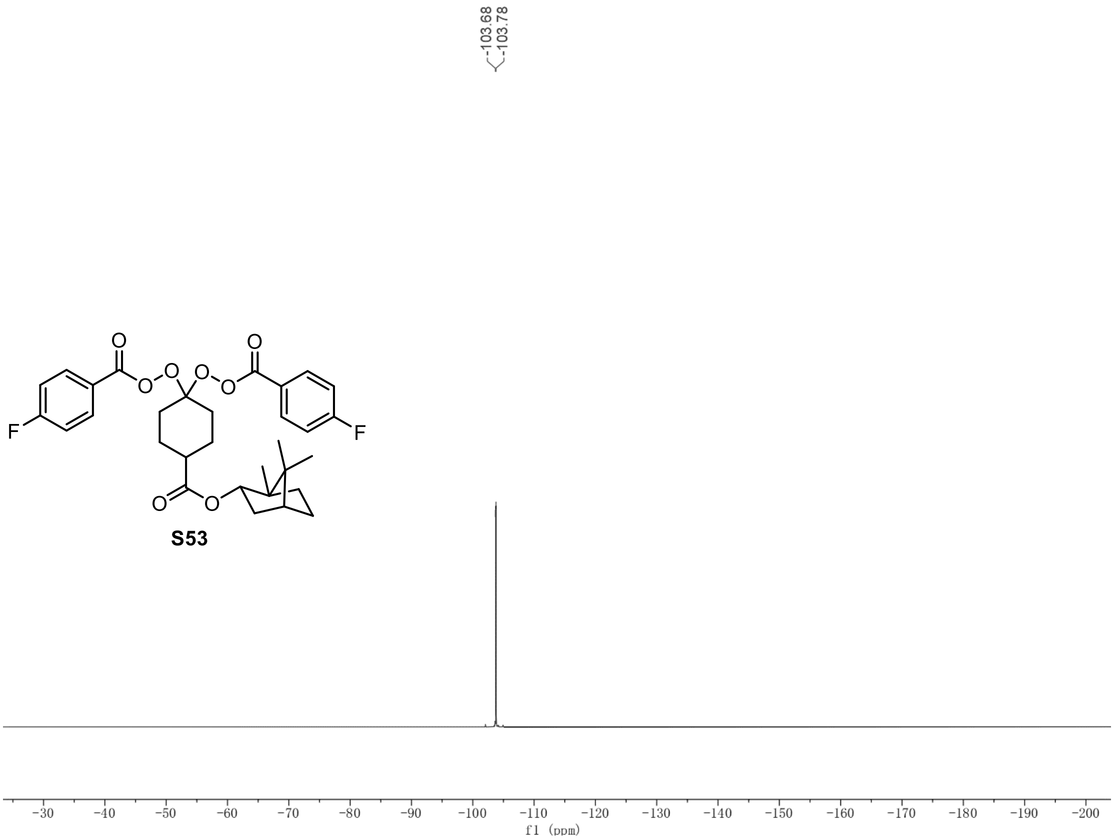

^{13}C NMR (100 MHz, $CDCl_3$) of **S53**

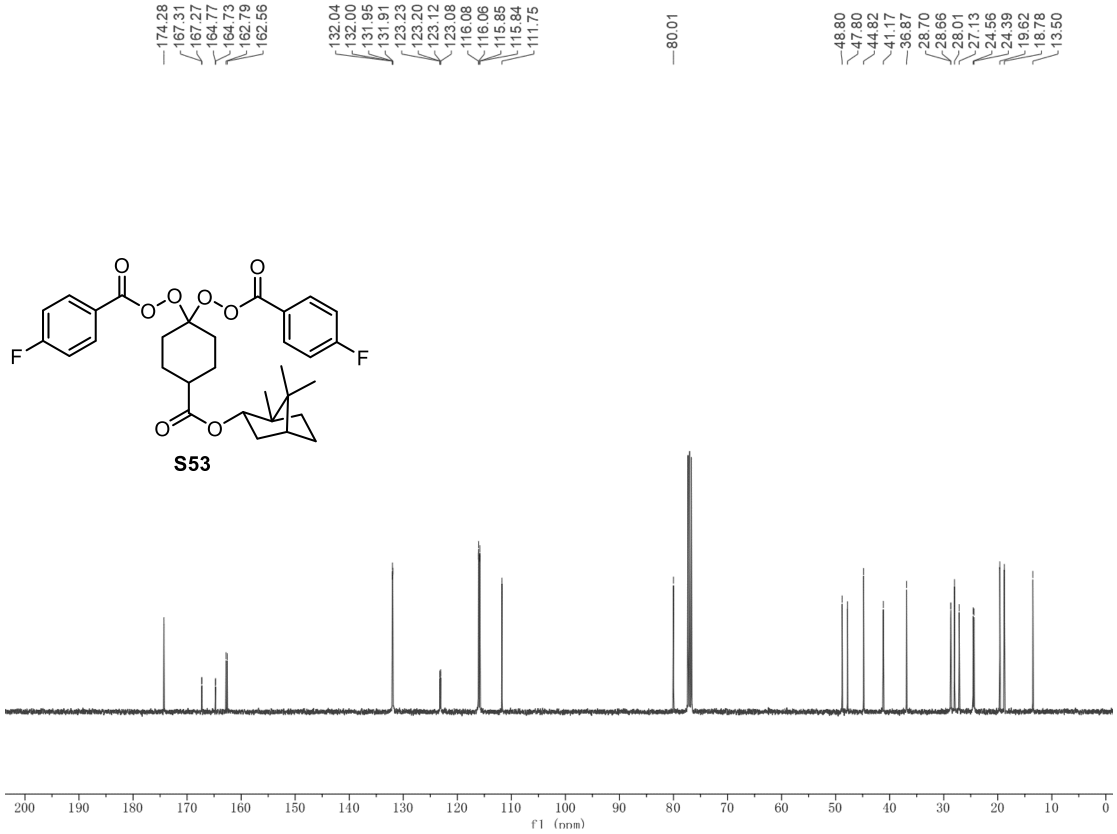

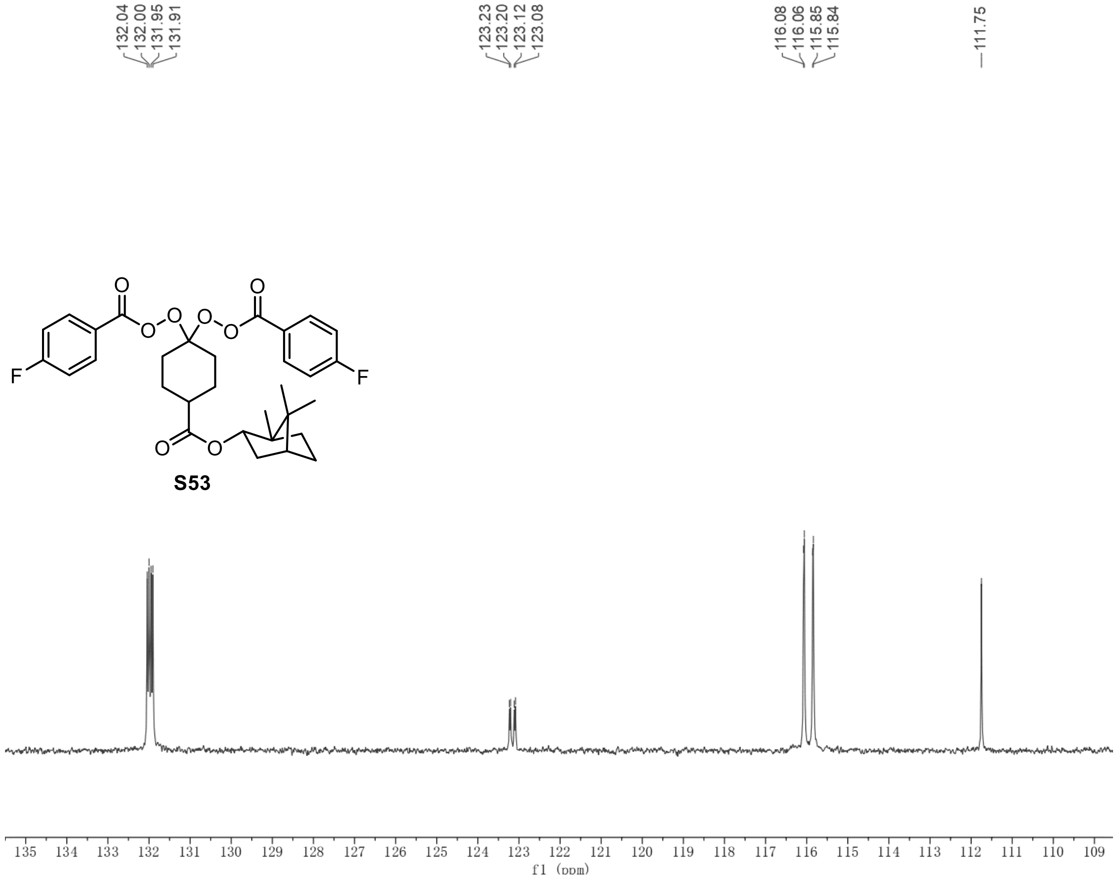

^{1}H NMR (400 MHz, CDCl$_3$) of **S54**

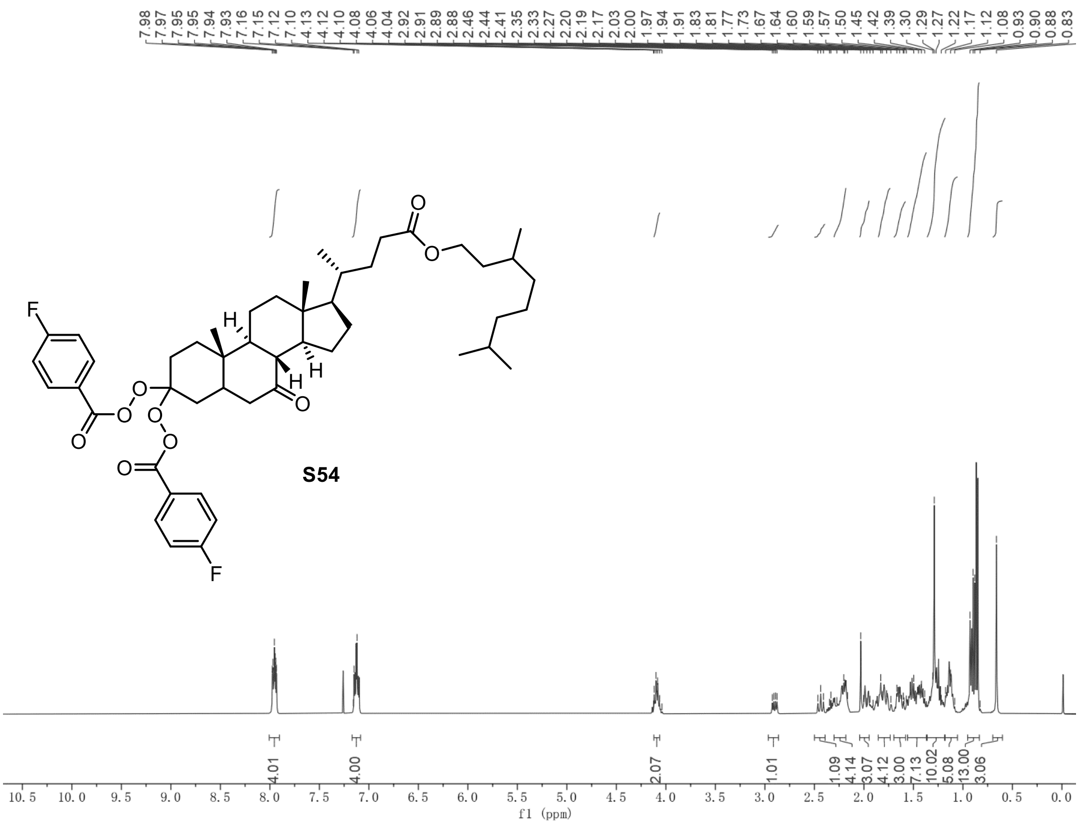

^{19}F NMR (376 MHz, CDCl$_3$) of **S54**

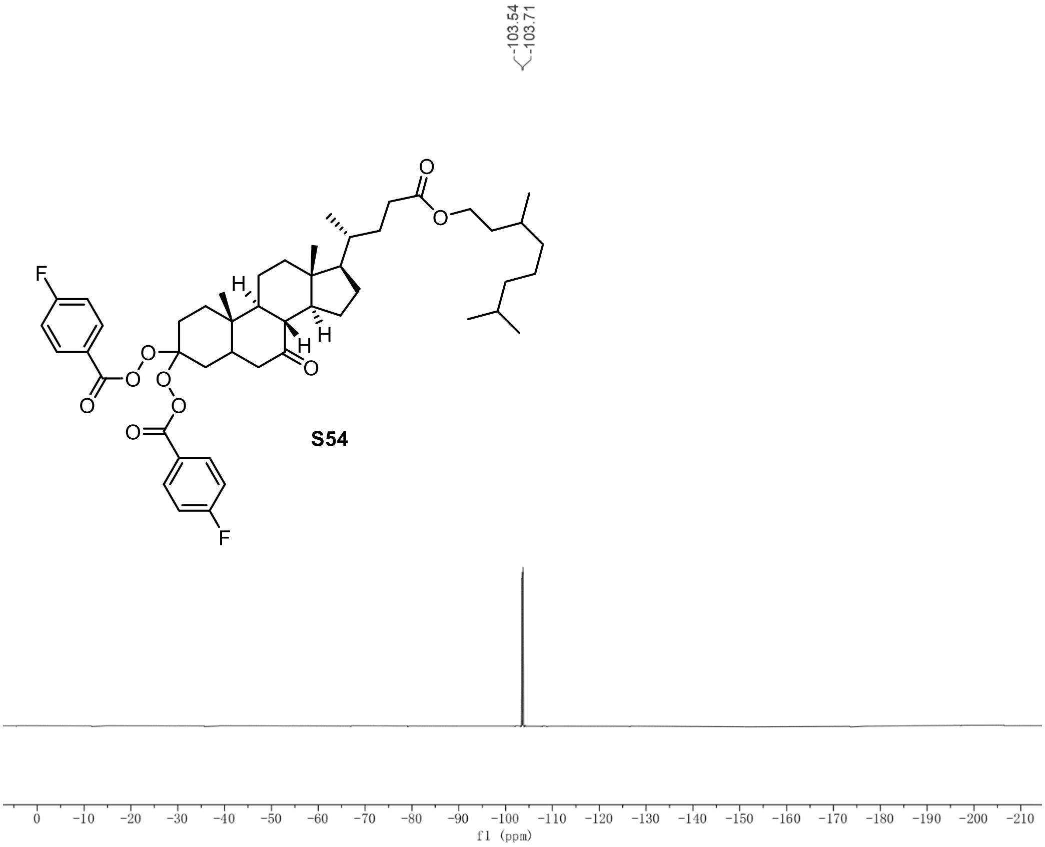

^{13}**C NMR** (100 MHz, $CDCl_3$) of **S54**

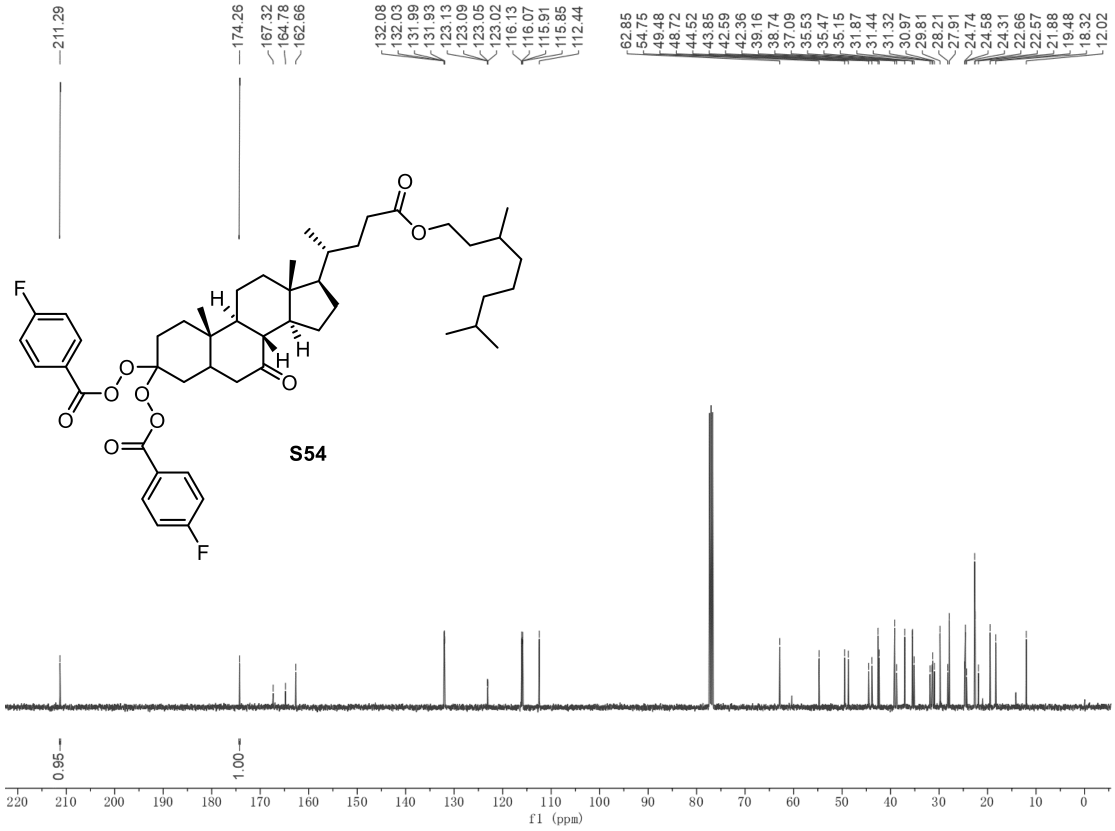

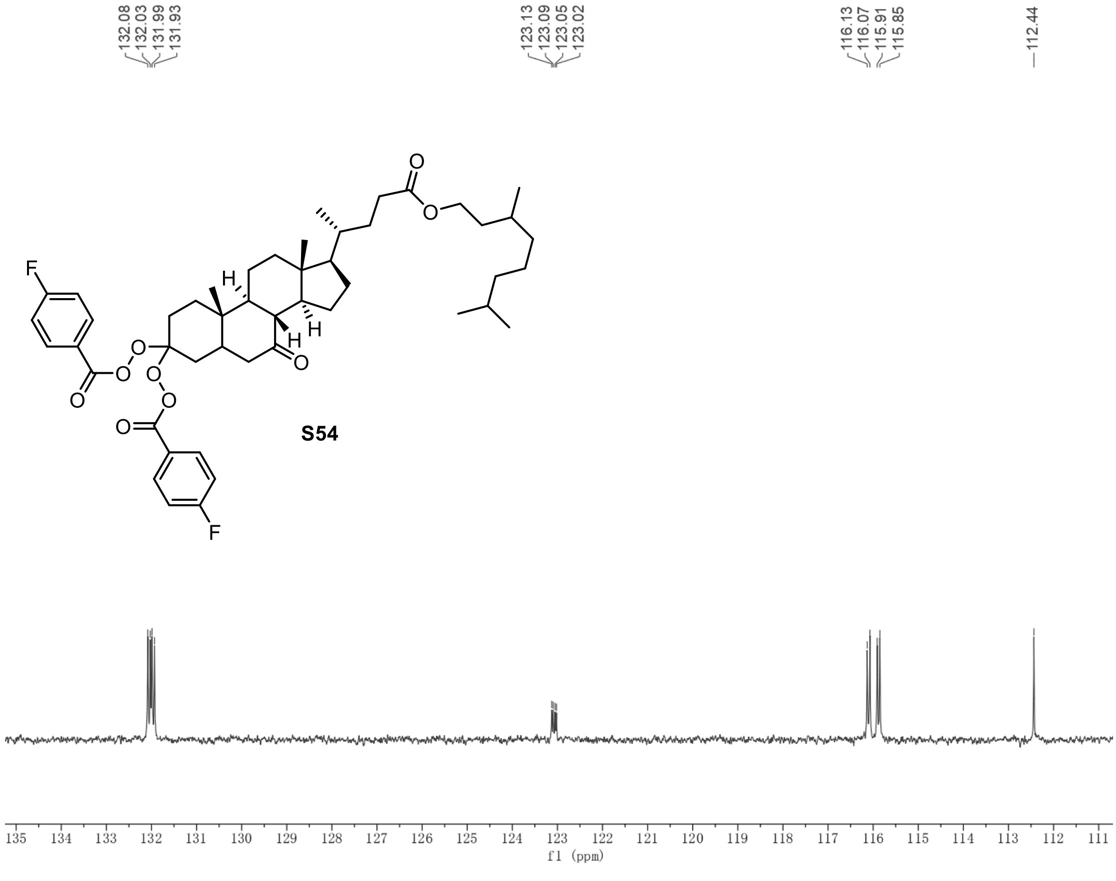

¹H NMR (400 MHz, DMSO-d$_6$) of **S55**

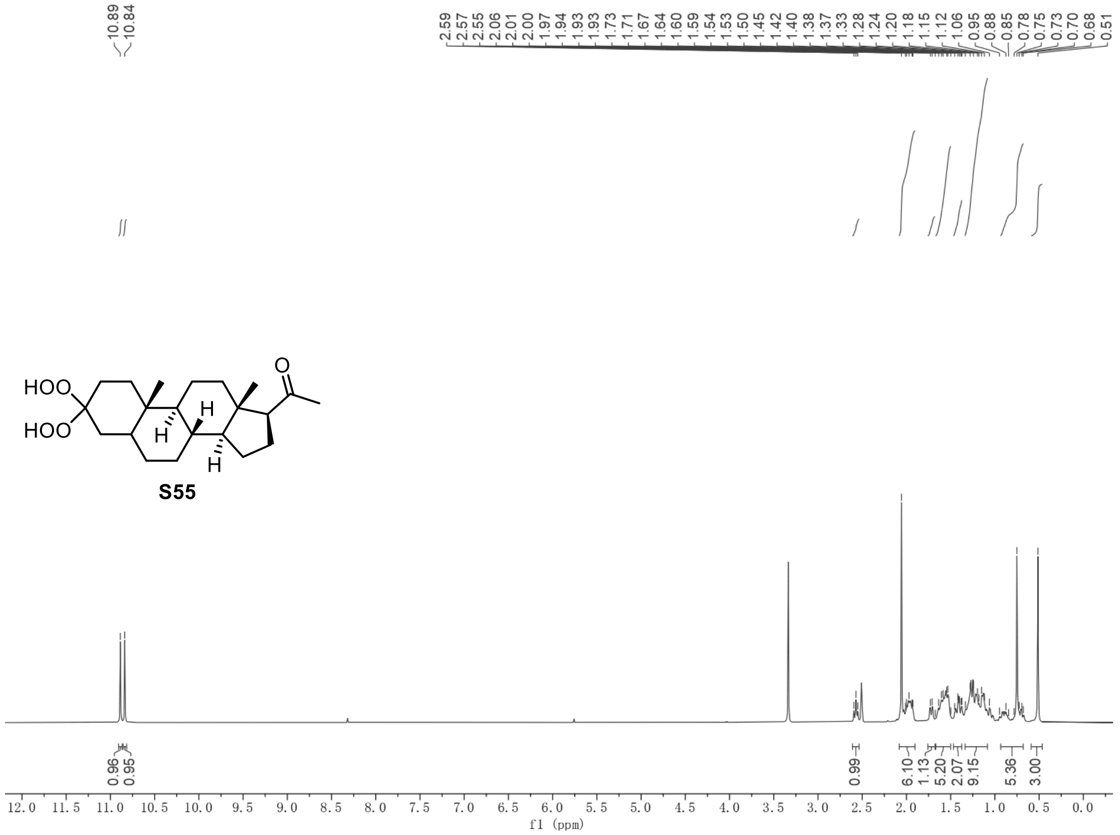

¹³C NMR (100 MHz, DMSO-d$_6$) of **S55**

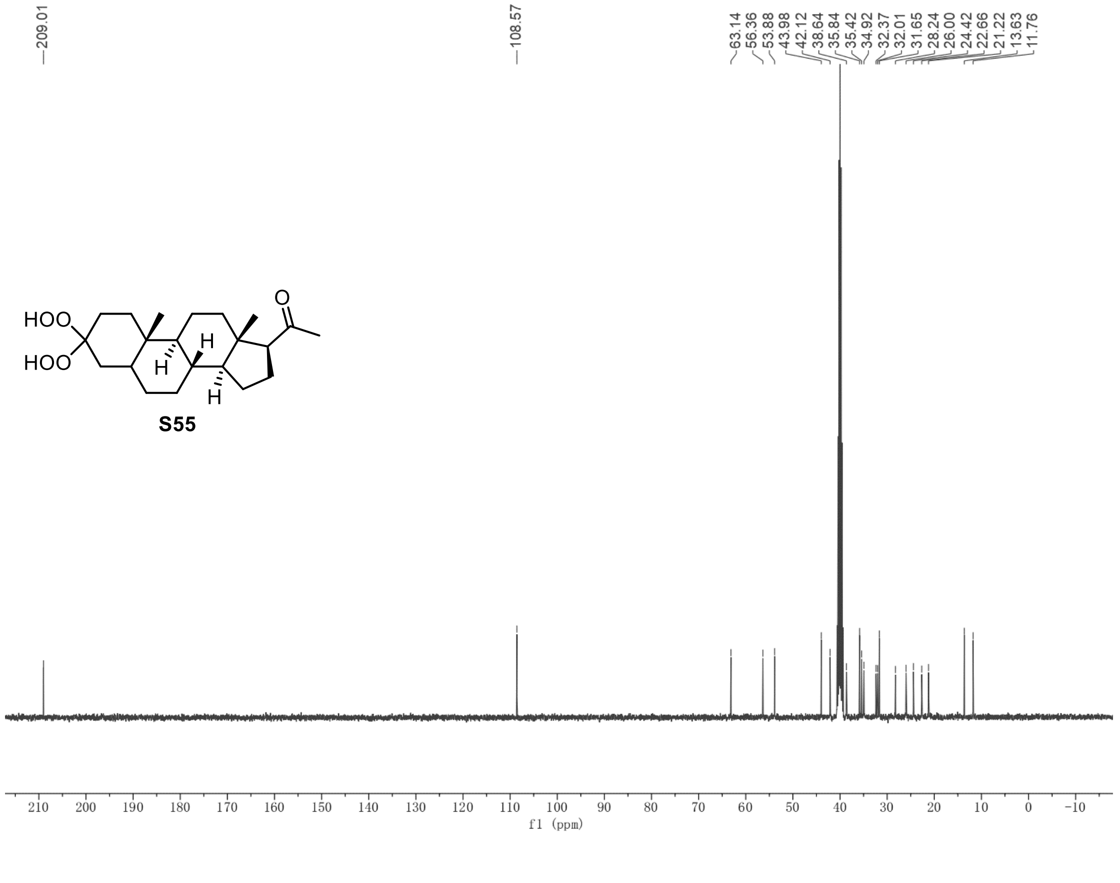

^{1}H NMR (400 MHz, $CDCl_3$) of **S56**

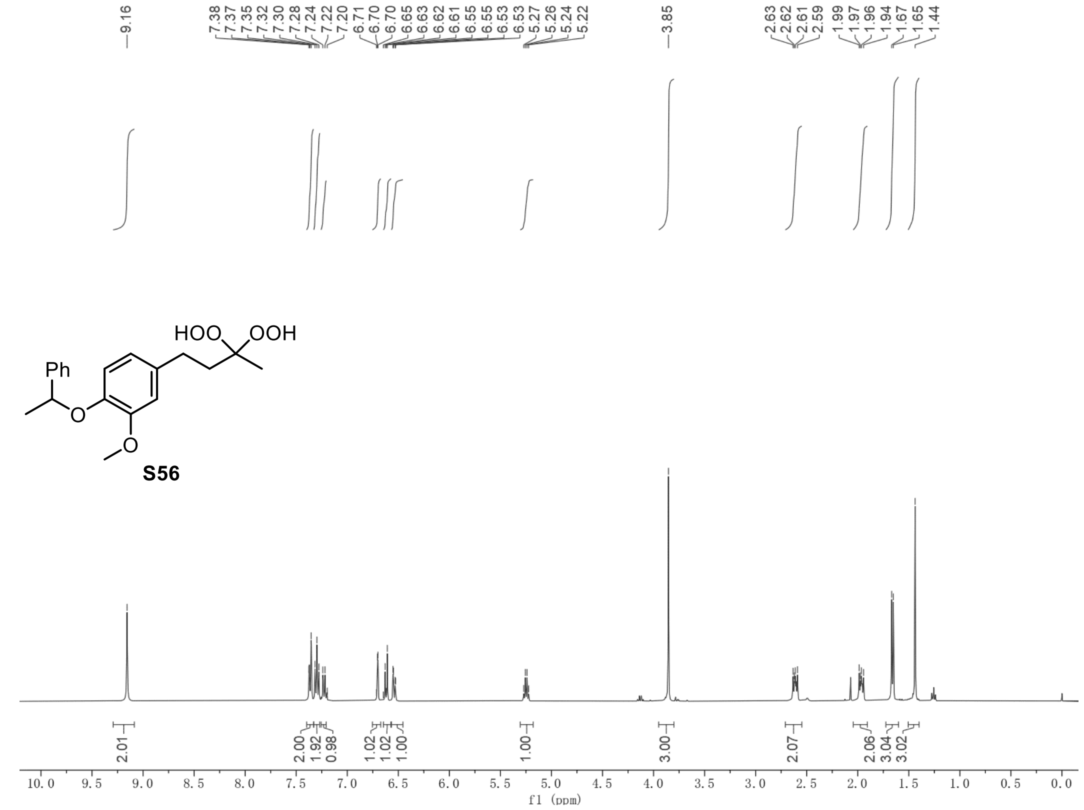

^{13}C NMR (100 MHz, $CDCl_3$) of **S56**

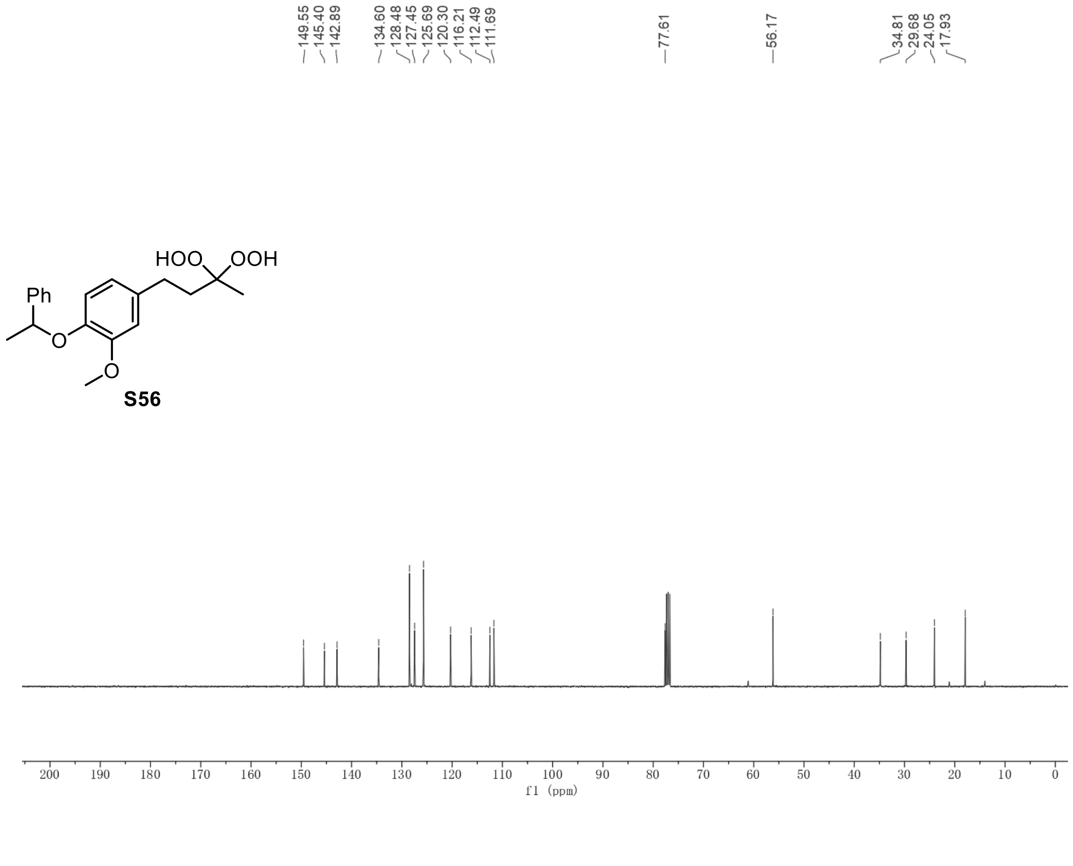

^{1}H NMR (400 MHz, $CDCl_3$) of **S57**

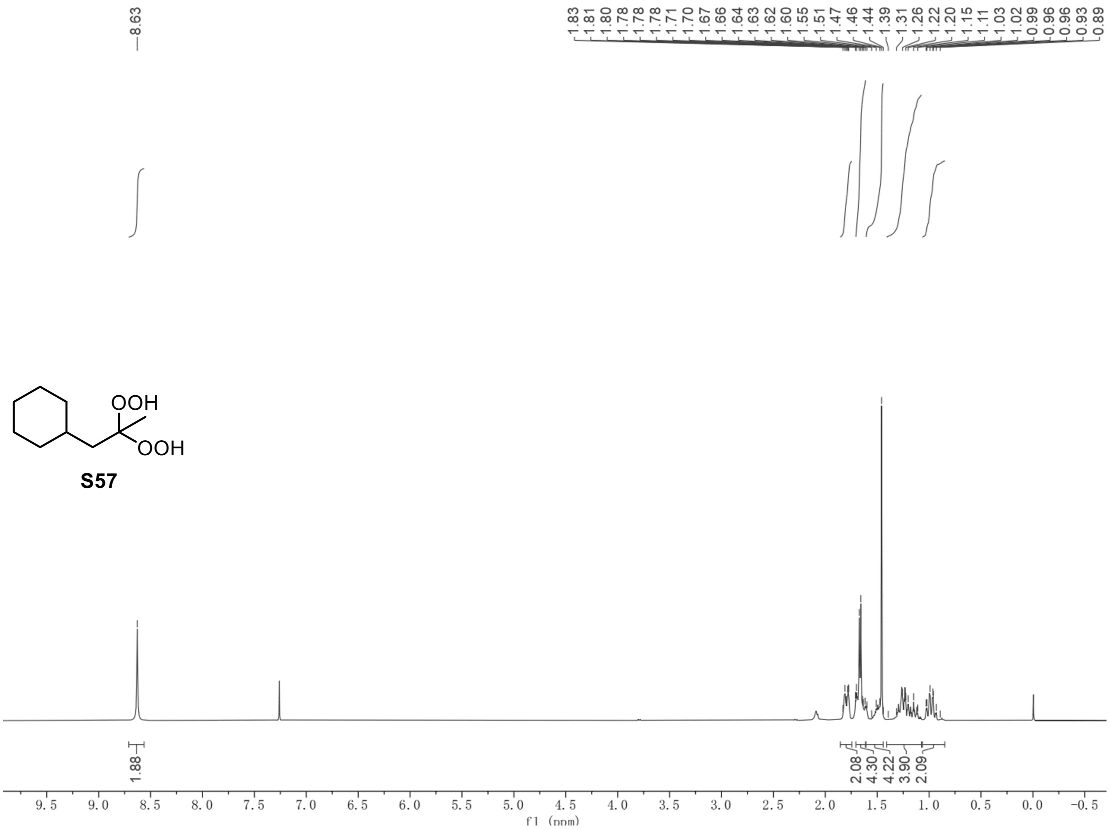

^{13}C NMR (100 MHz, $CDCl_3$) of **S57**

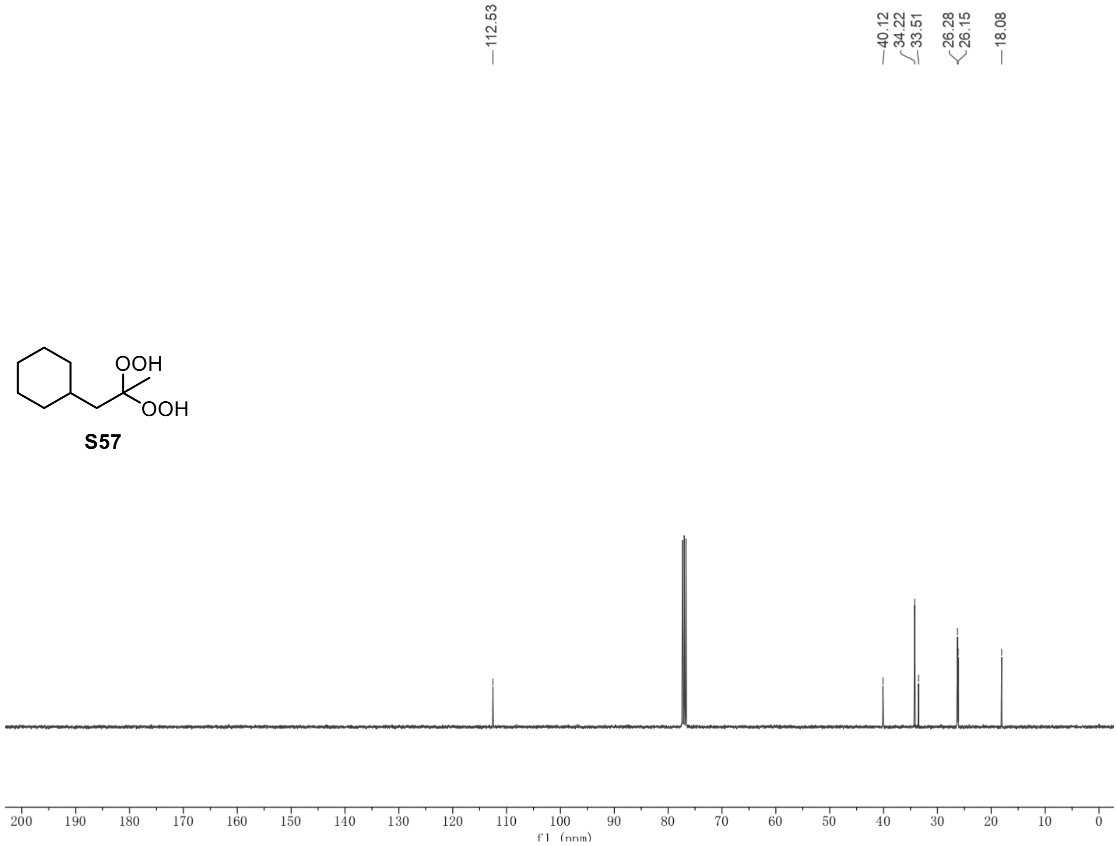

^{1}H NMR (400 MHz, $CDCl_3$) of **S58**

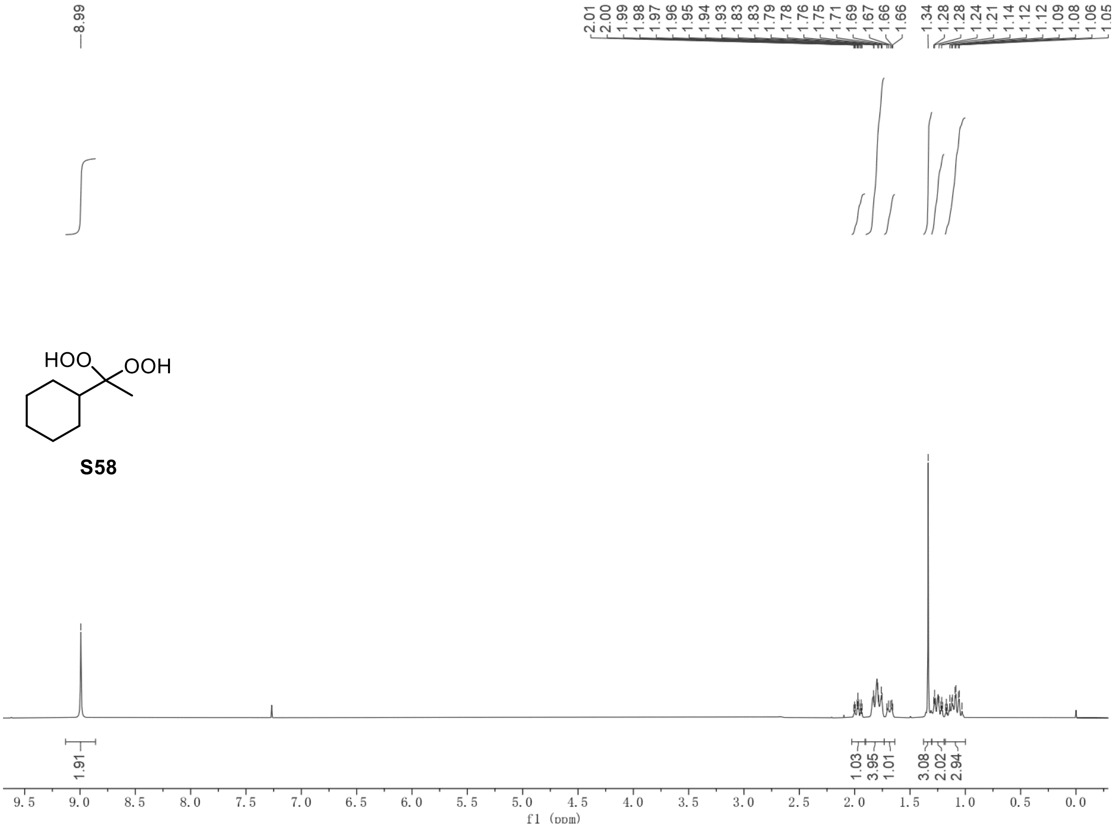

^{13}C NMR (100 MHz, $CDCl_3$) of **S58**

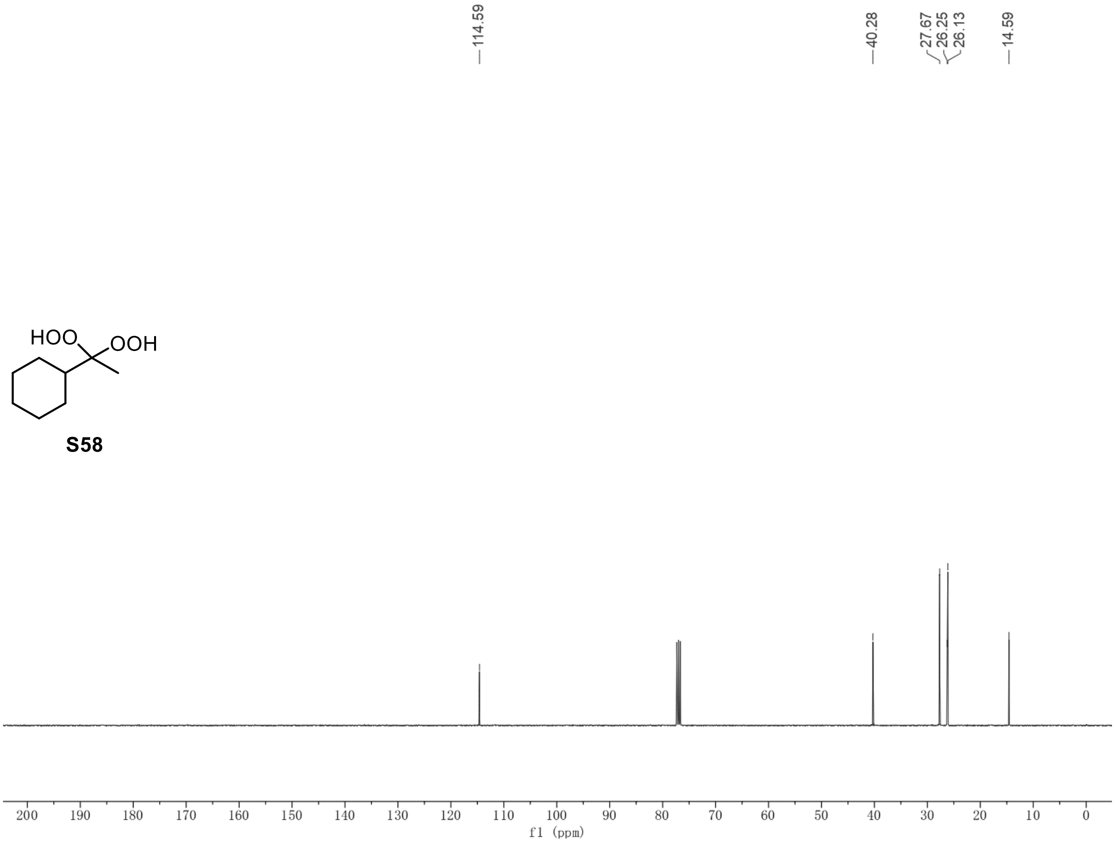

11. NMR spectra of products

^{1}H NMR (400 MHz, $CDCl_3$) of **1**

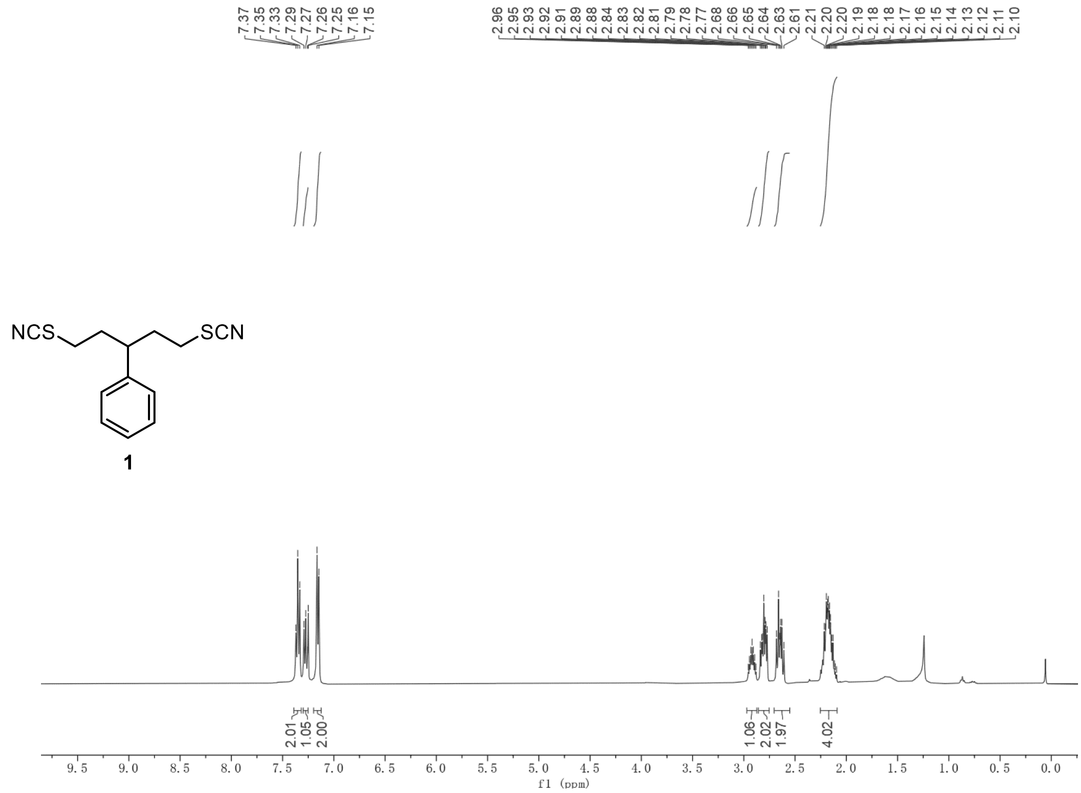

^{13}C NMR (100 MHz, $CDCl_3$) of **1**

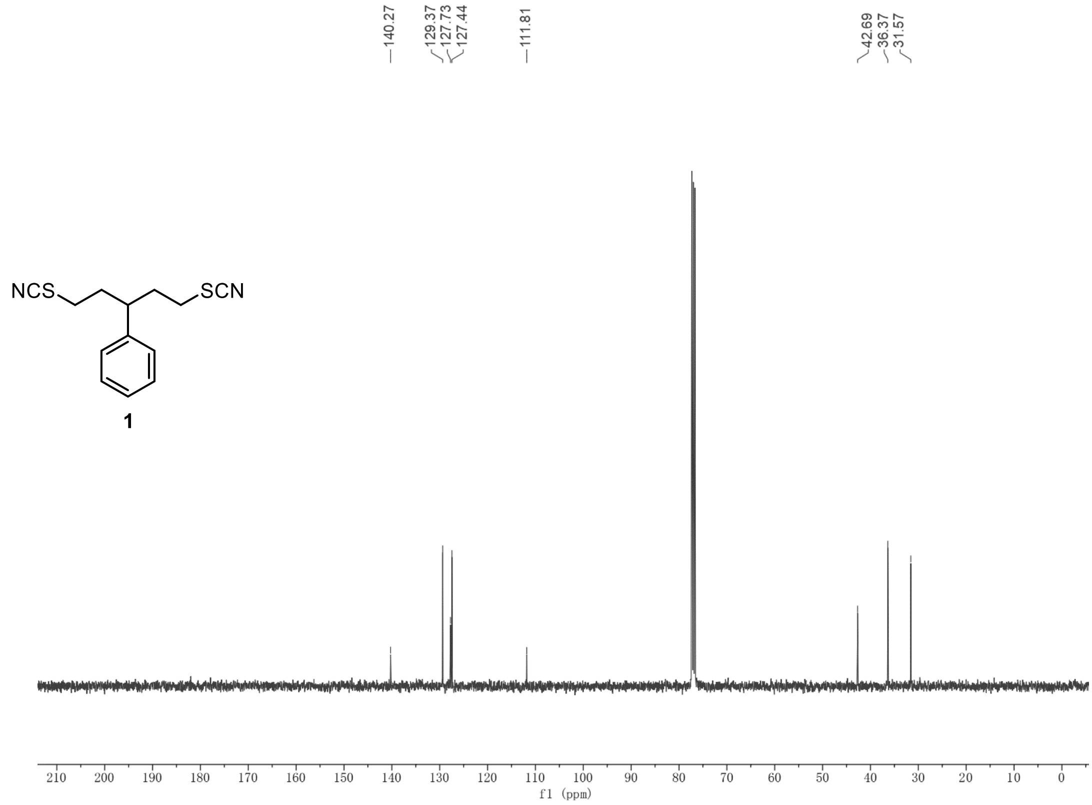

^{1}H NMR (400 MHz, $CDCl_3$) of **2**

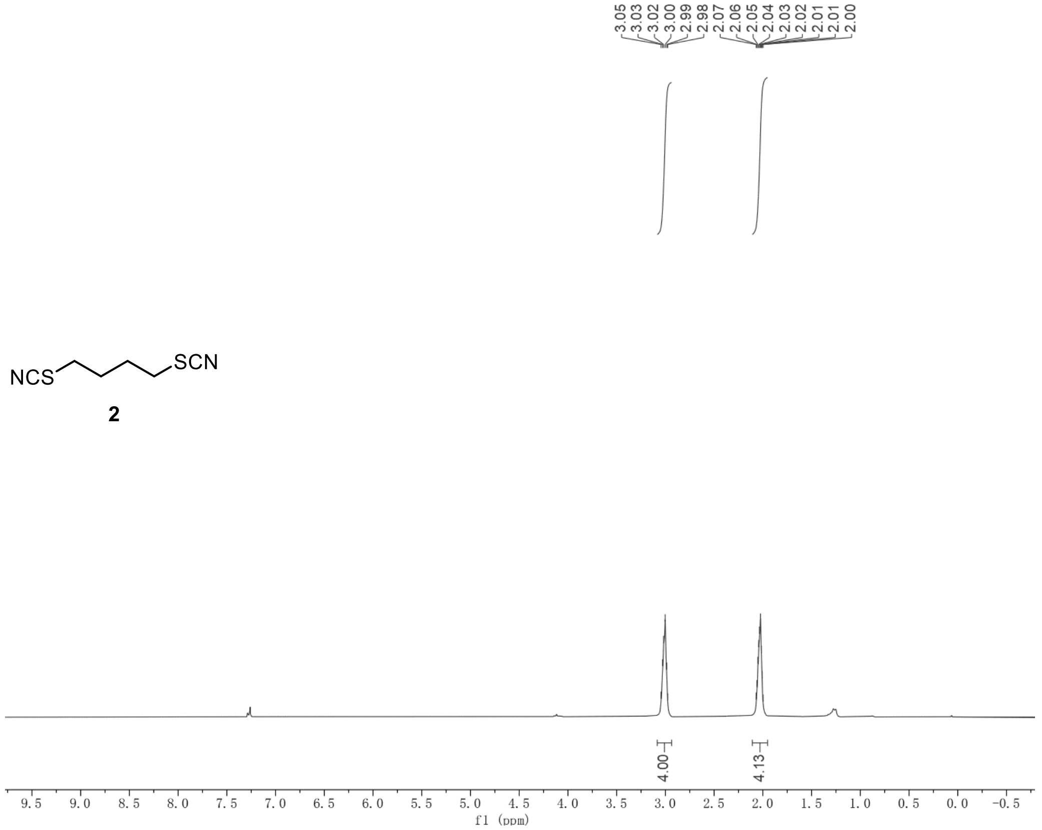

^{13}C NMR (100 MHz, $CDCl_3$) of **2**

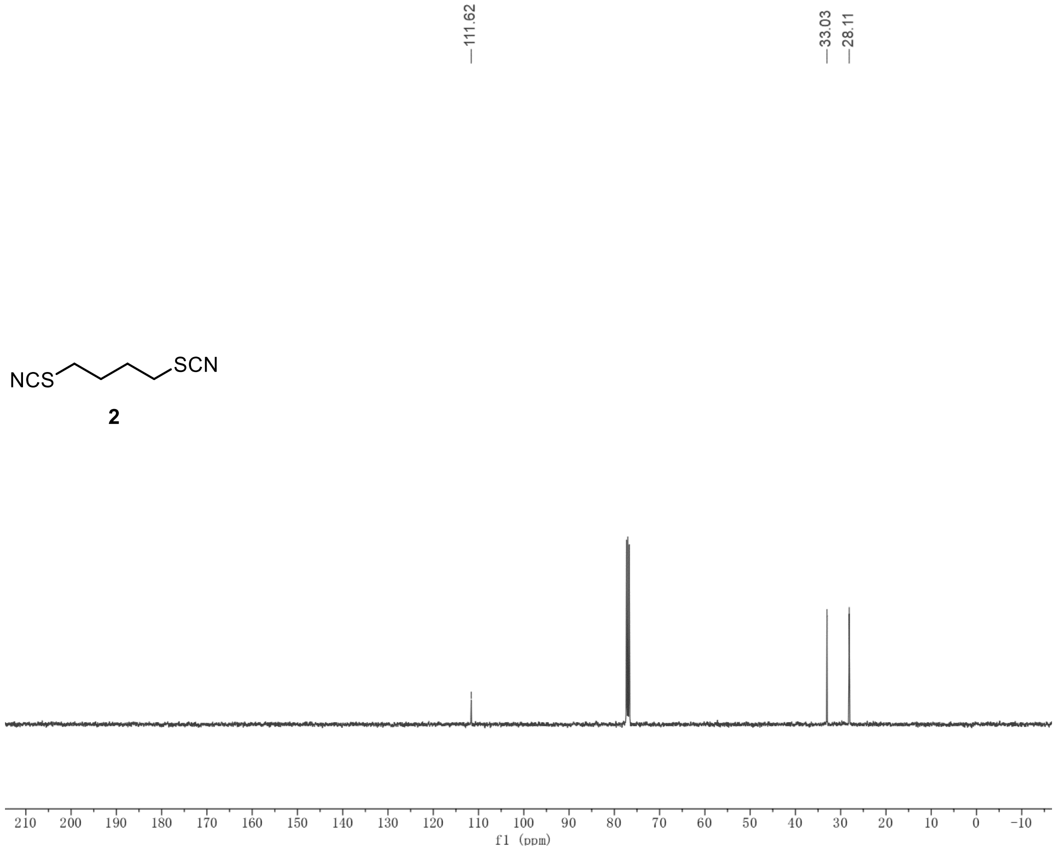

^{1}H NMR (400 MHz, $CDCl_3$) of **3**

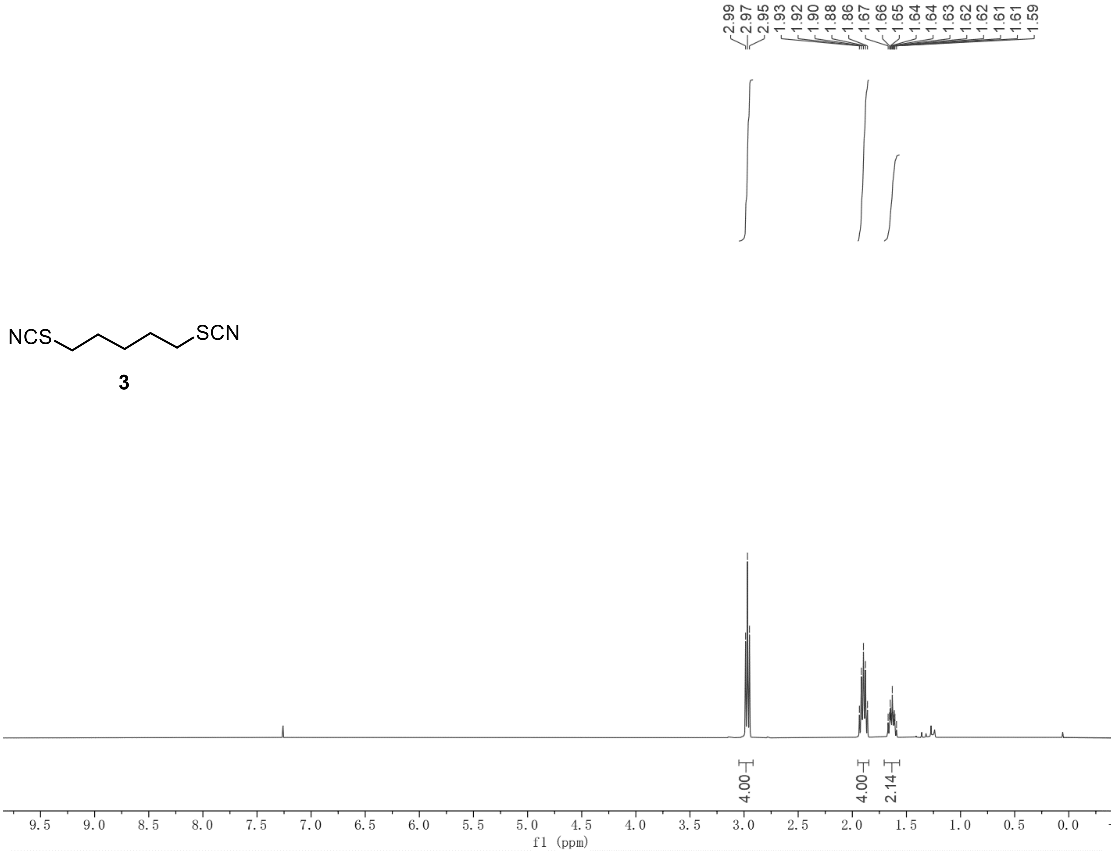

3

^{13}C NMR (100 MHz, $CDCl_3$) of **3**

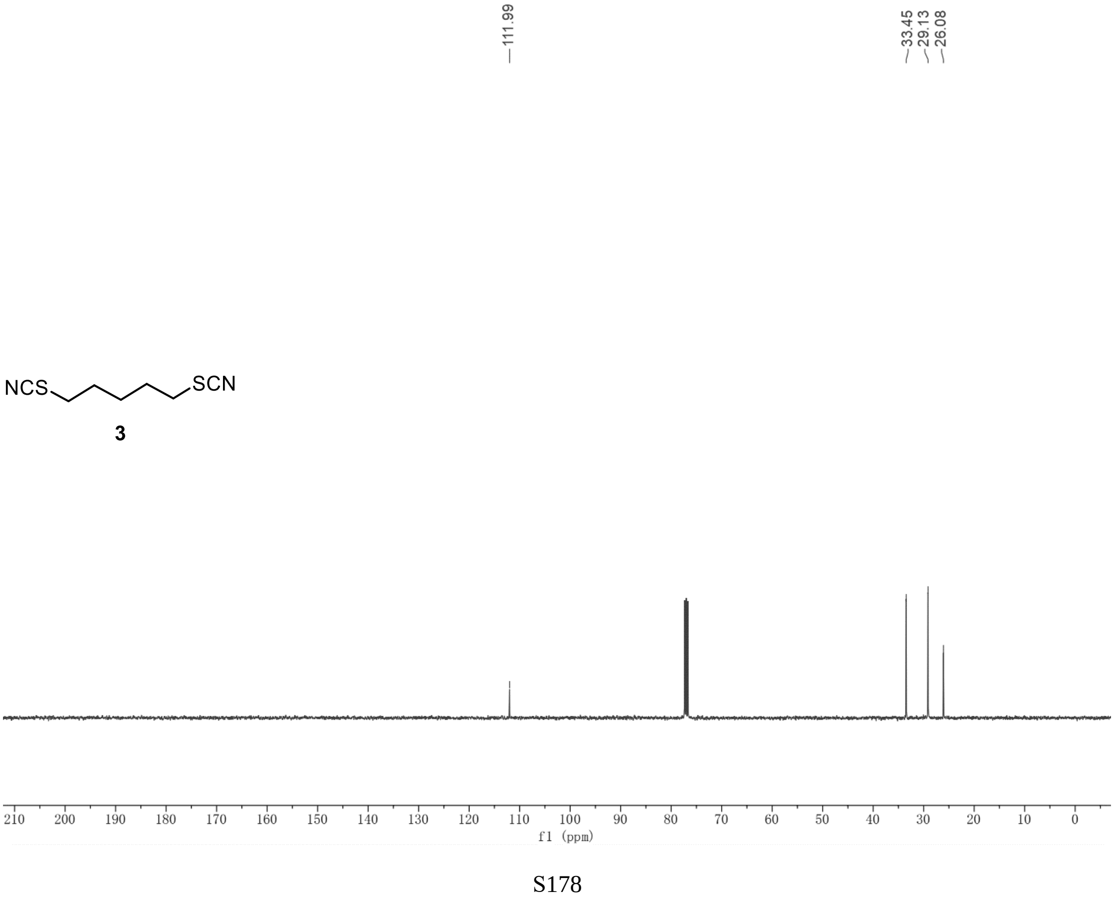

3

^{1}H NMR (400 MHz, $CDCl_3$) of **4**

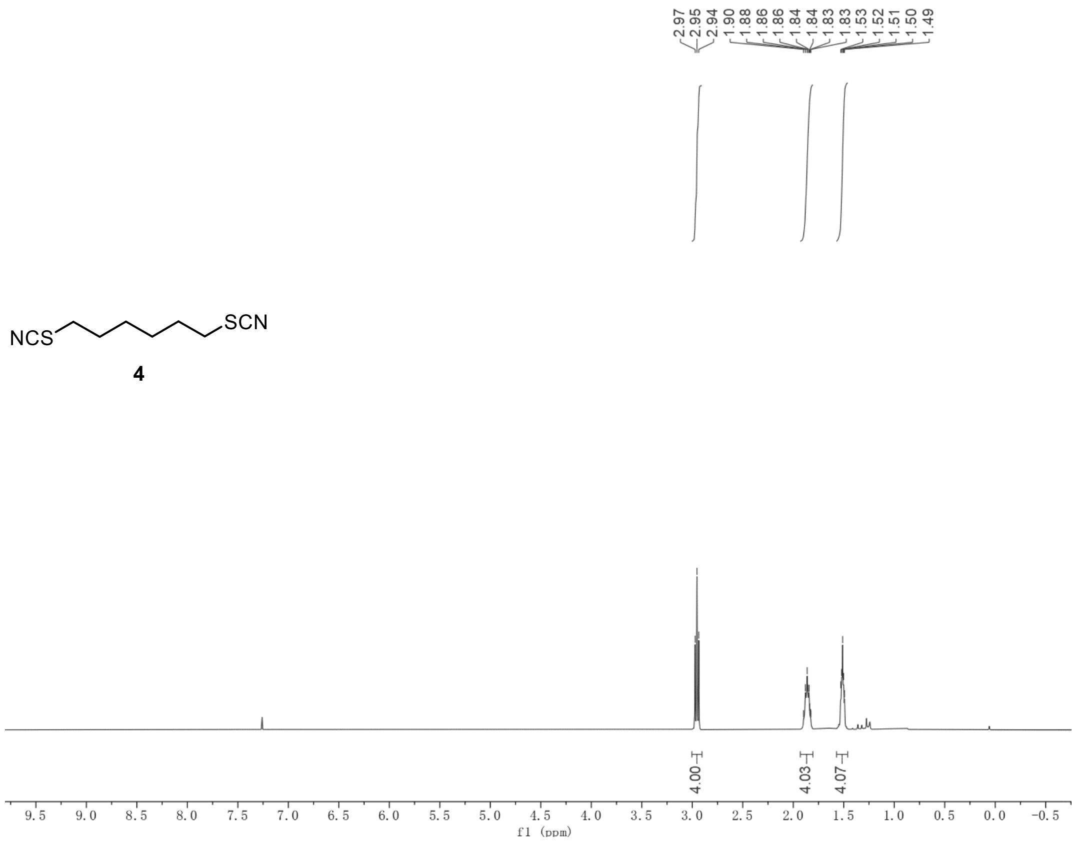

^{13}C NMR (100 MHz, $CDCl_3$) of **4**

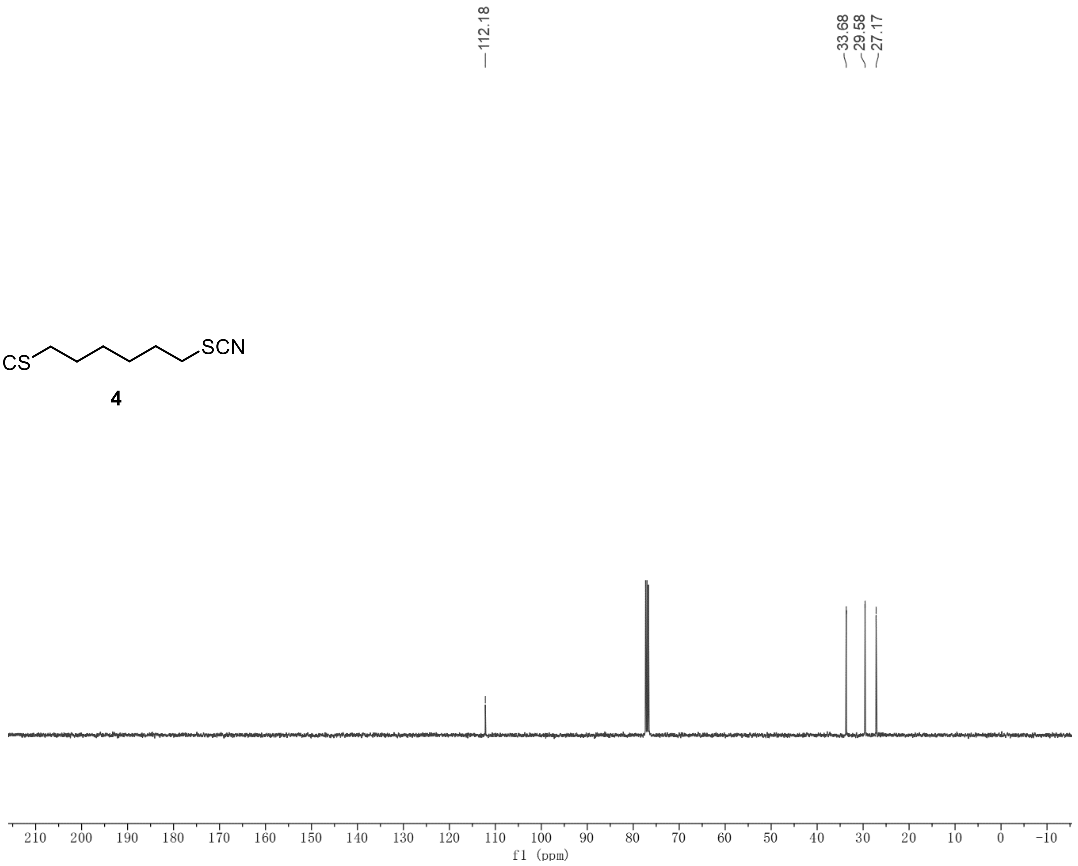

^{1}H NMR (400 MHz, $CDCl_3$) of **5**

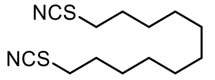

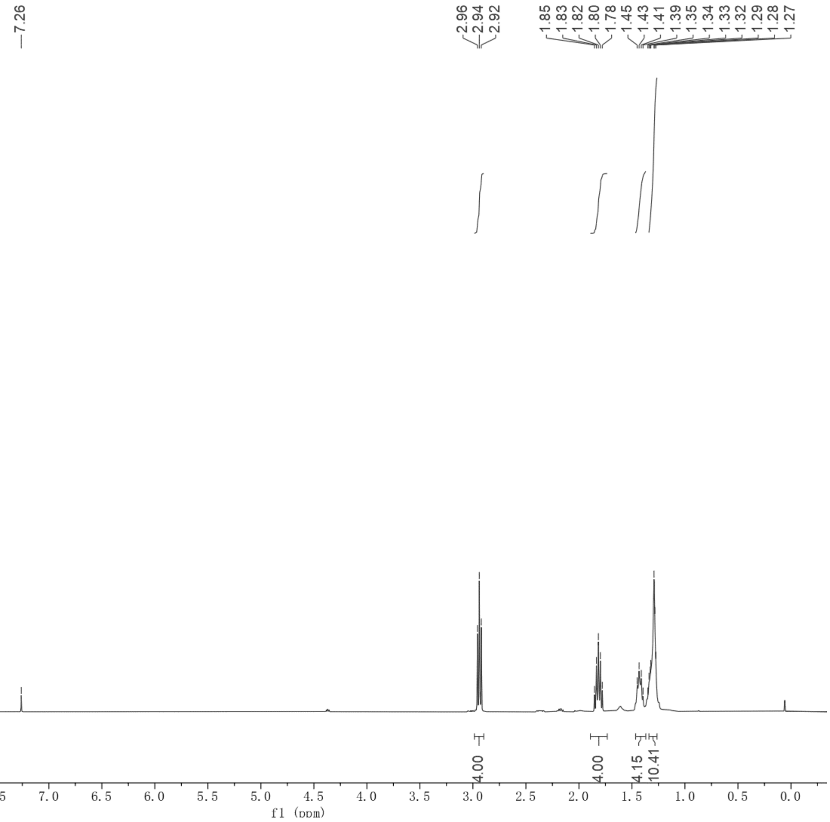

5

^{13}C NMR (100 MHz, $CDCl_3$) of **5**

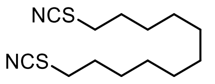

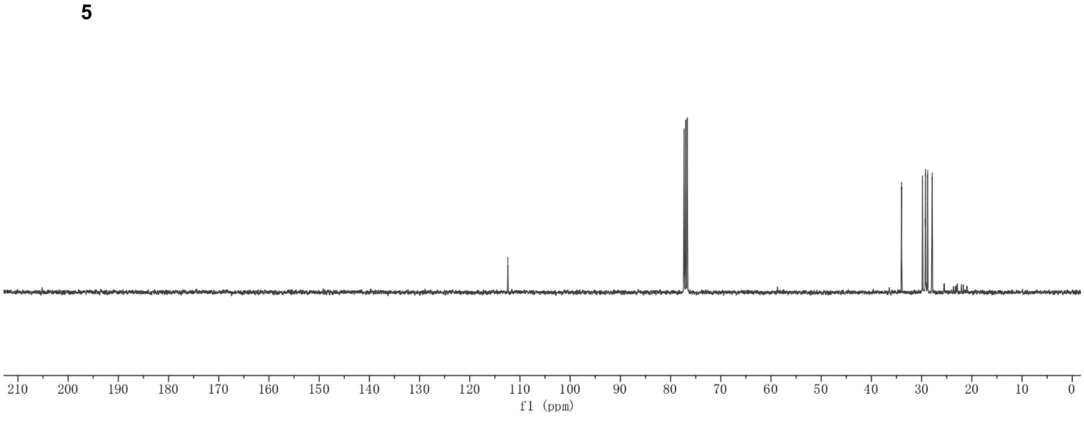

5

^{1}H NMR (400 MHz, $CDCl_3$) of **6**

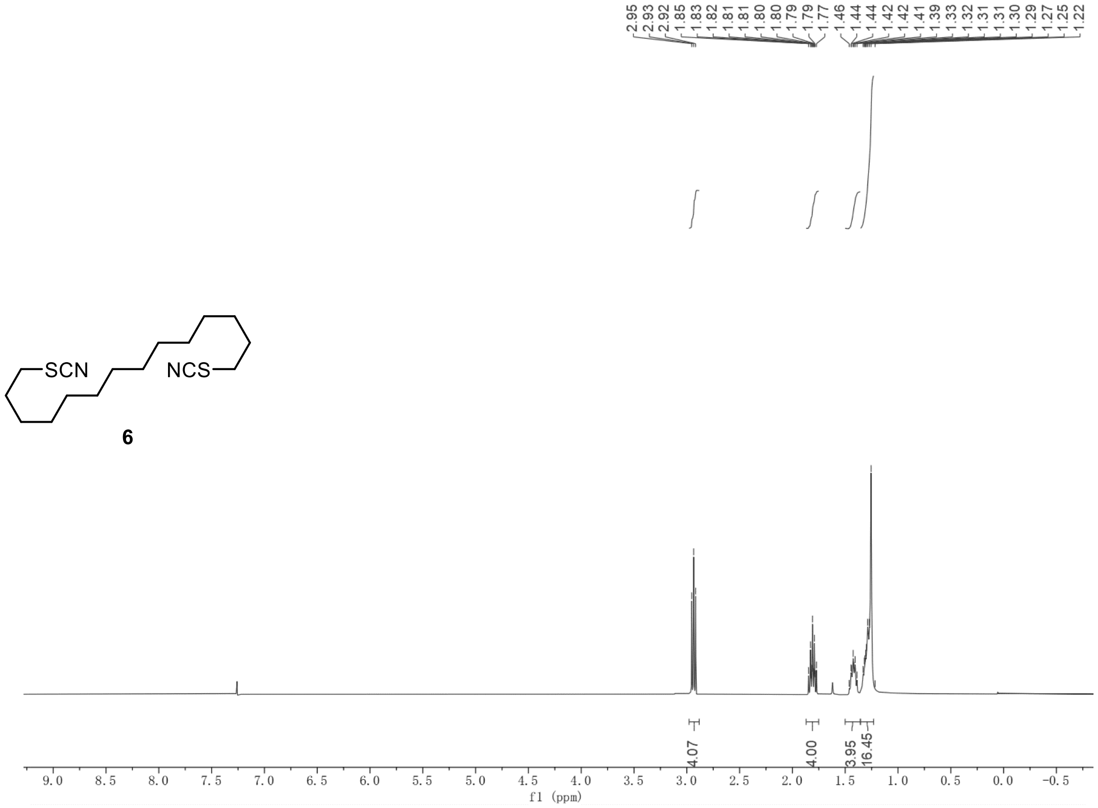

^{13}C NMR (100 MHz, $CDCl_3$) of **6**

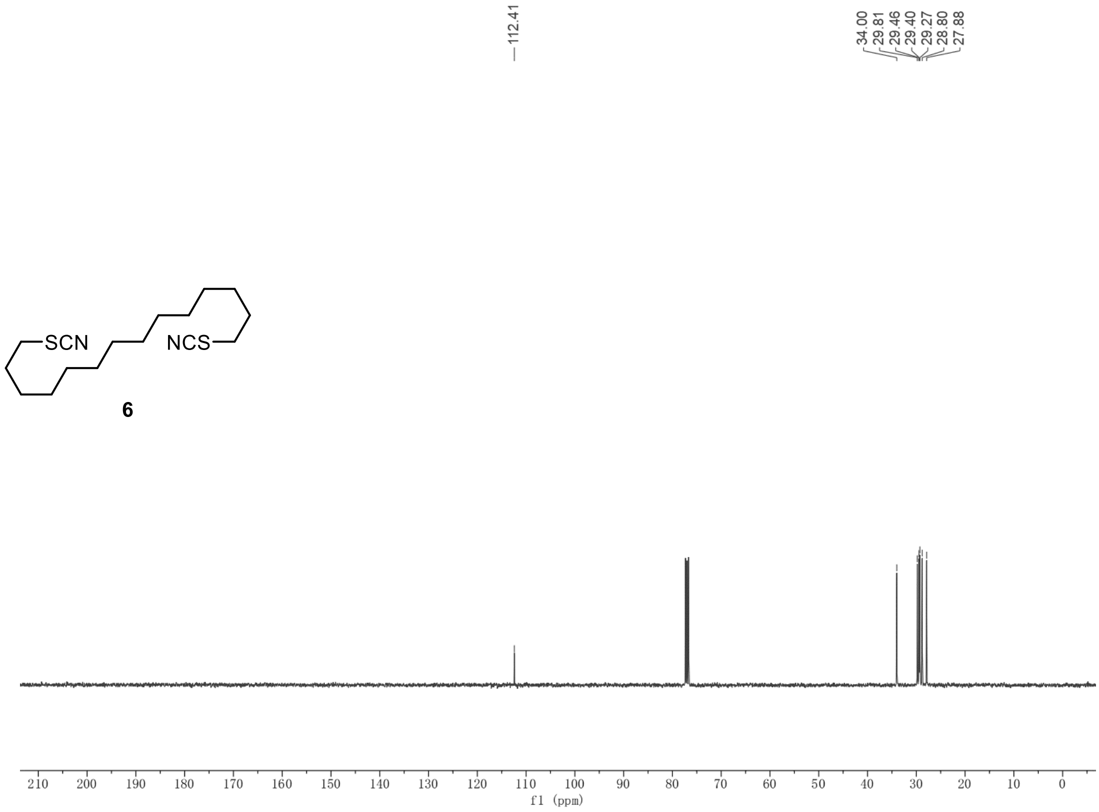

^{1}H NMR (400 MHz, $CDCl_3$) of **7**

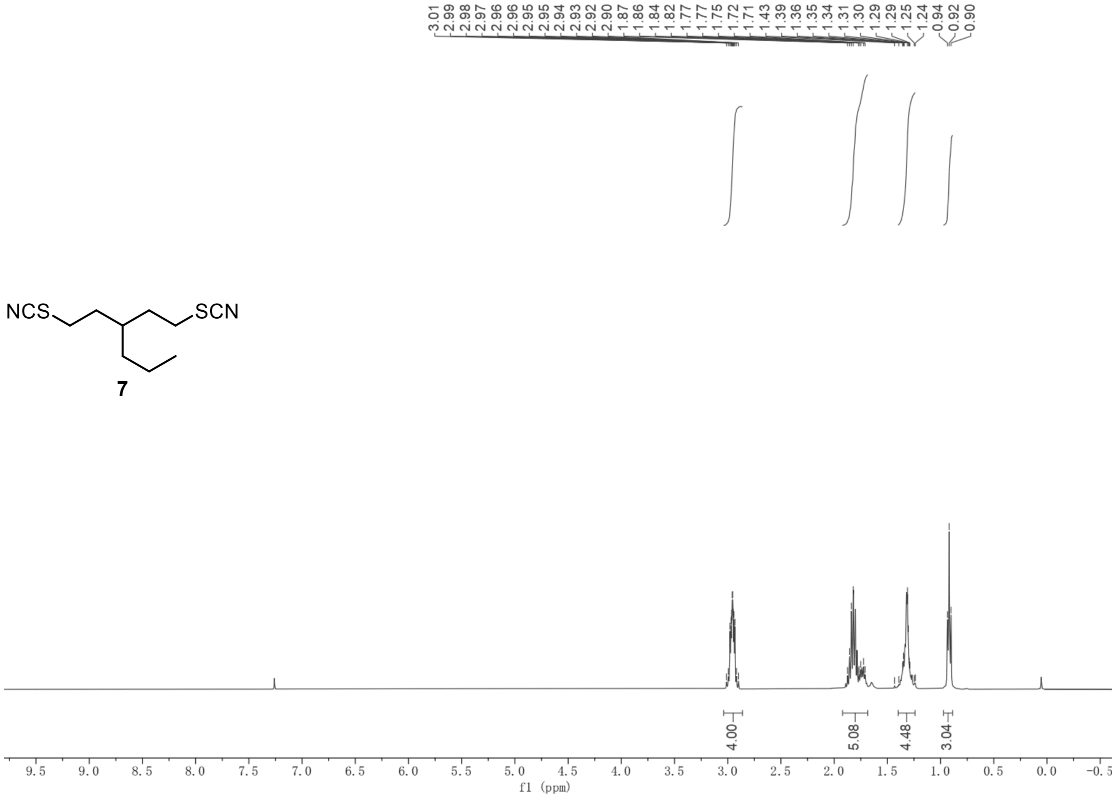

^{13}C NMR (100 MHz, $CDCl_3$) of **7**

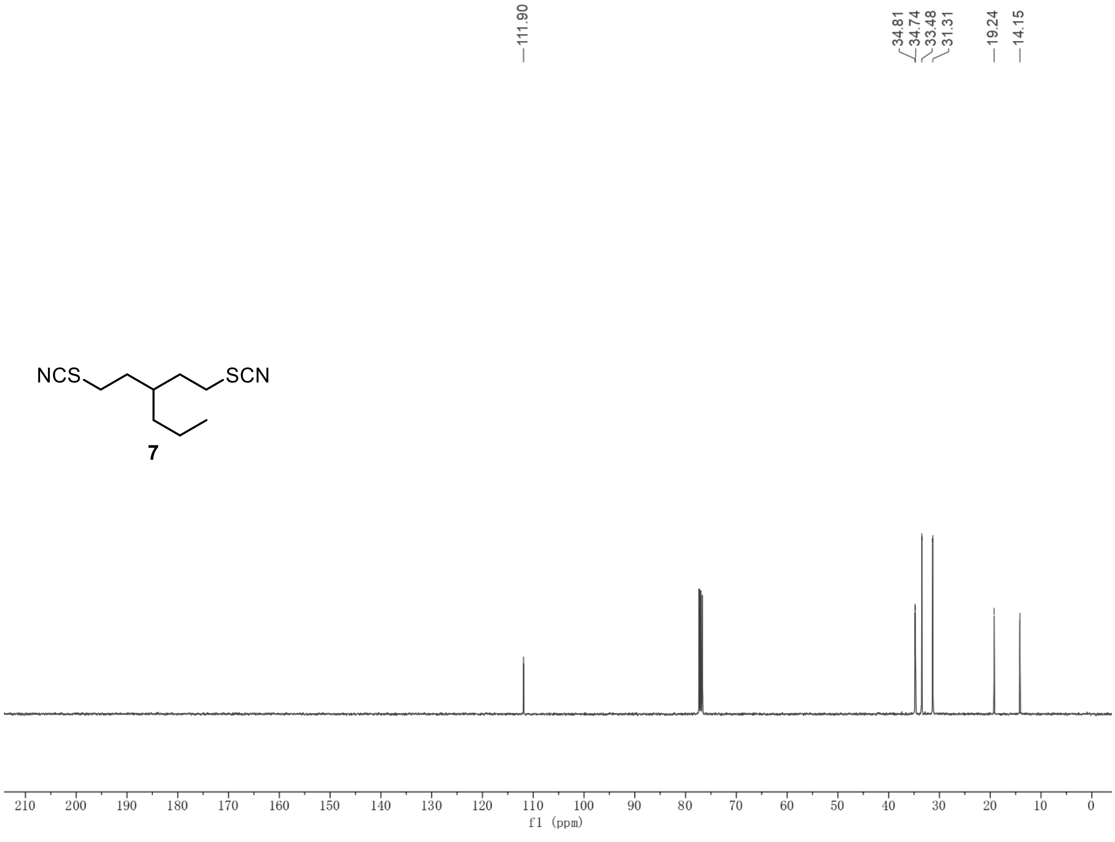

^{1}H NMR (400 MHz, $CDCl_3$) of **8**

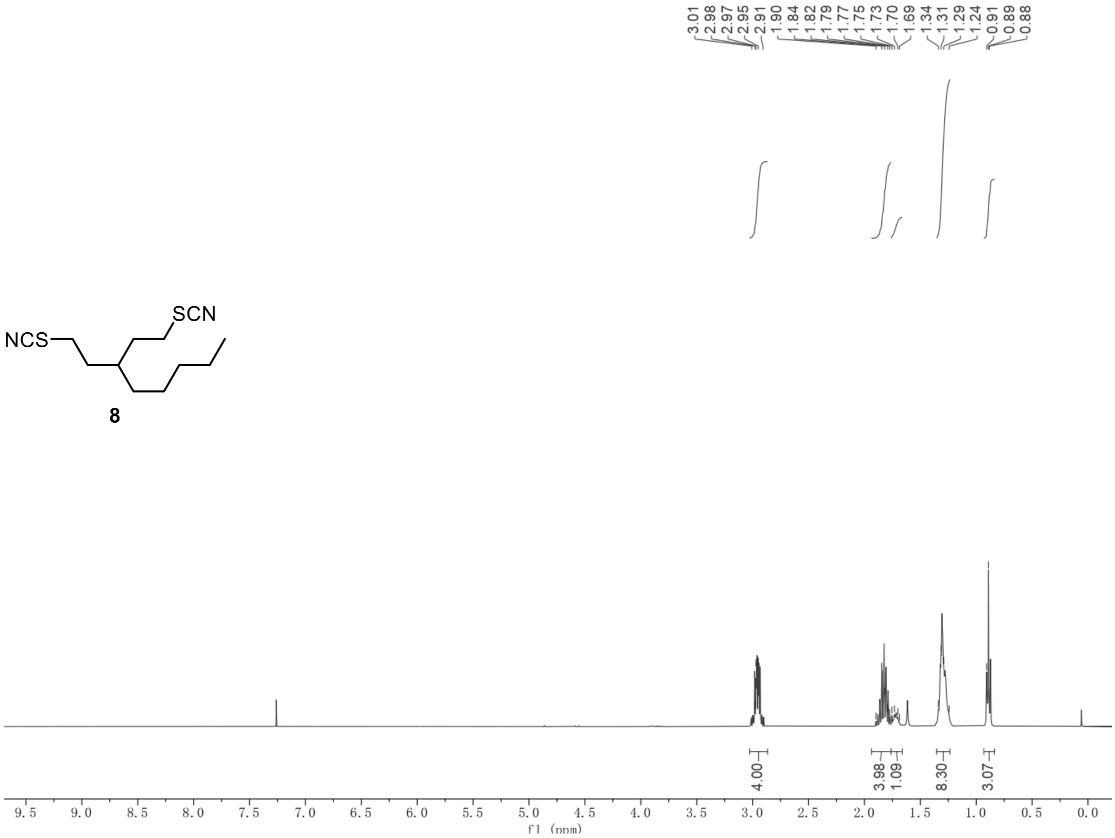

^{13}C NMR (100 MHz, $CDCl_3$) of **8**

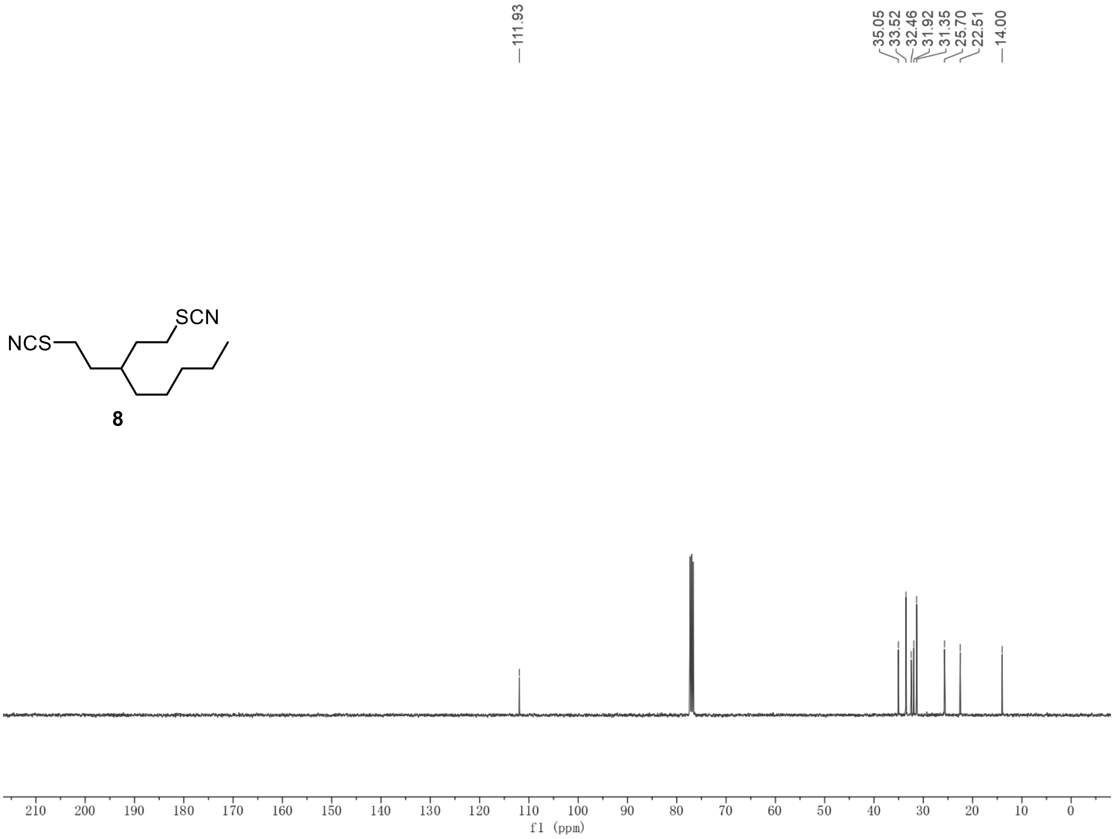

¹H NMR (400 MHz, $CDCl_3$) of **9**

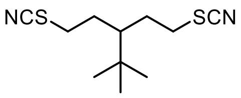

9

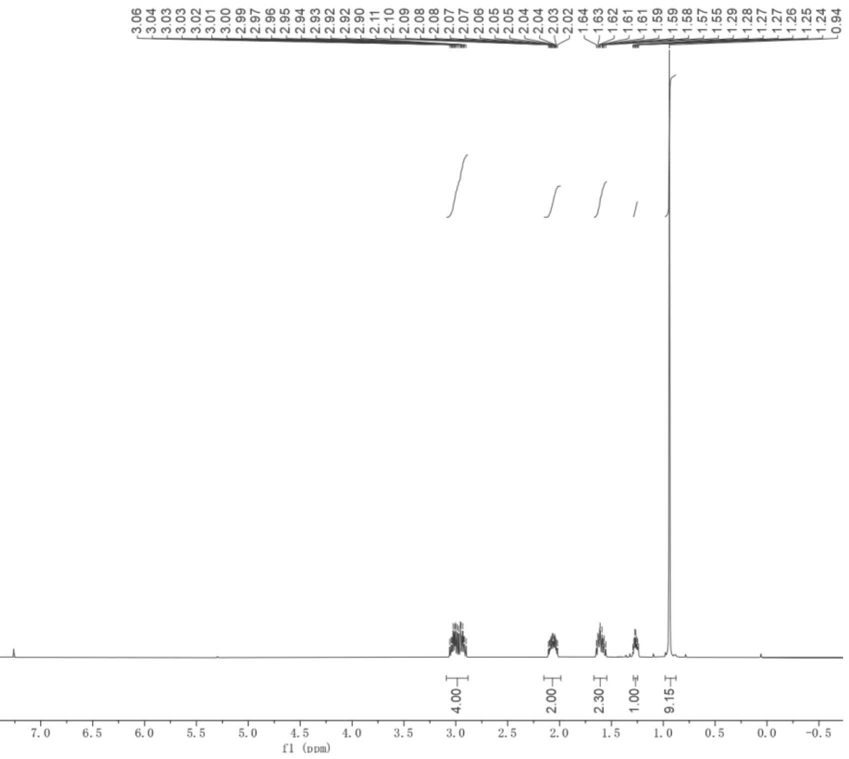

¹³C NMR (100 MHz, $CDCl_3$) of **9**

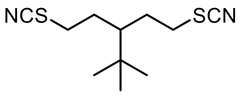

9

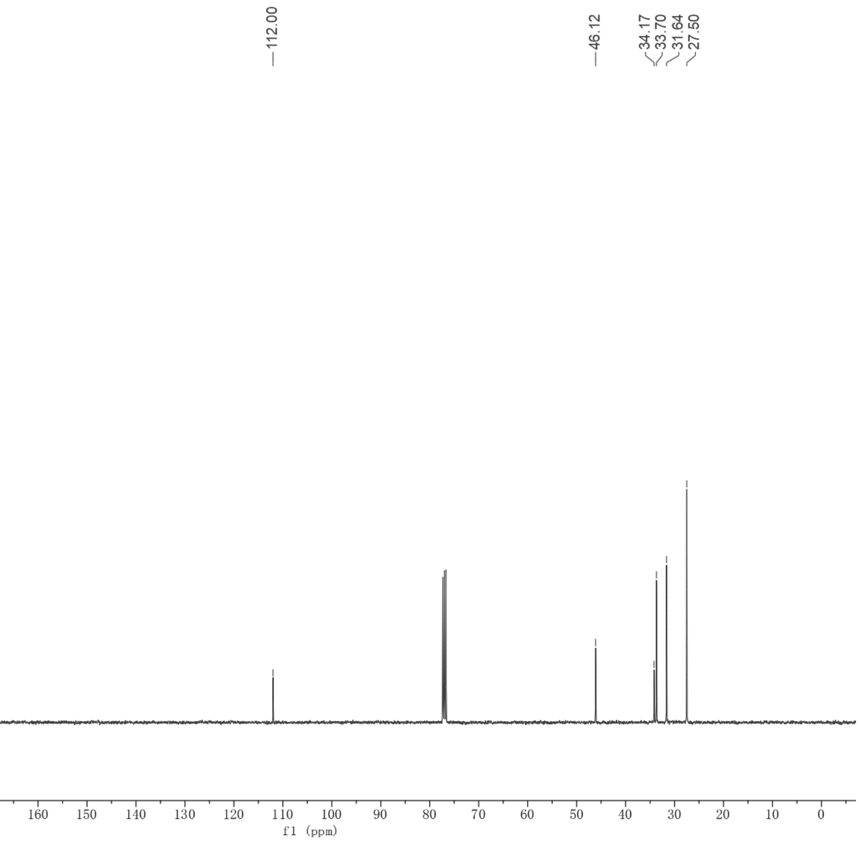

^{1}H NMR (400 MHz, $CDCl_3$) of **10**

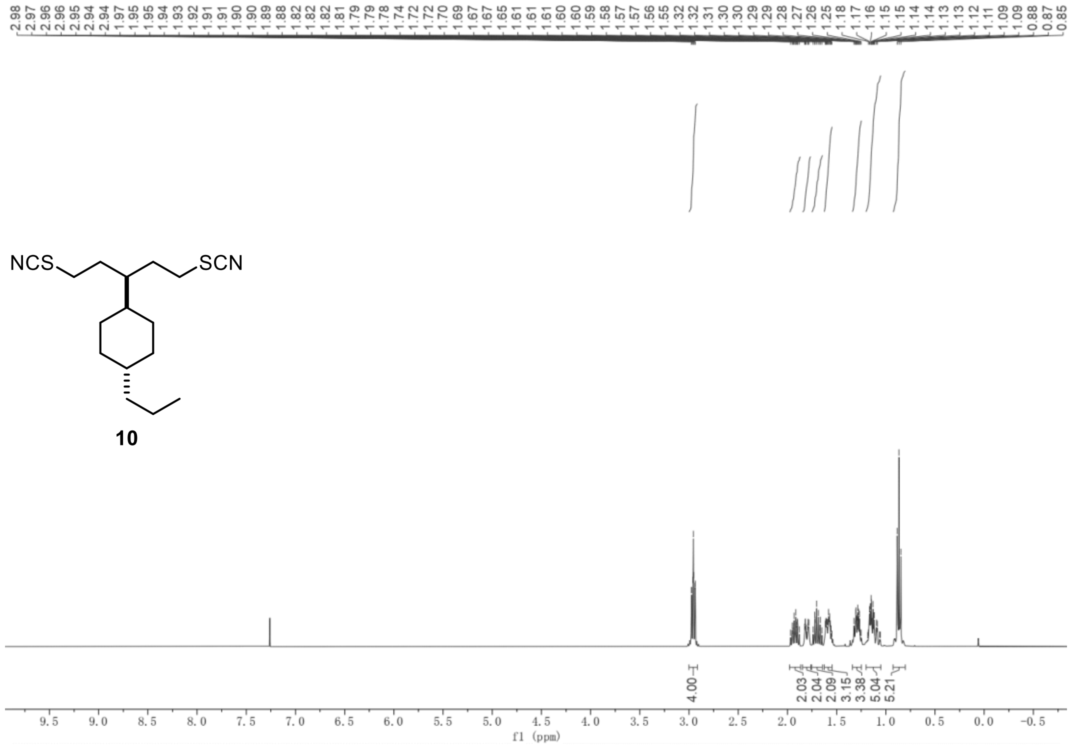

^{13}C NMR (100 MHz, $CDCl_3$) of **10**

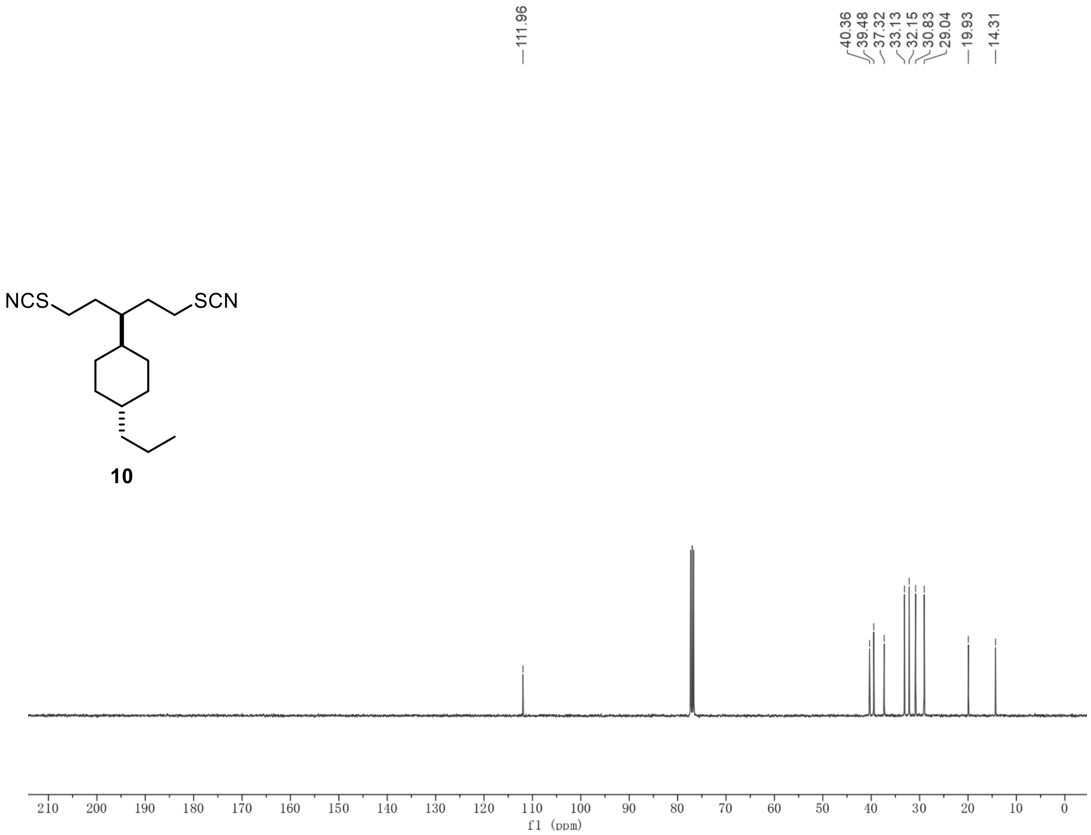

^{1}H NMR (400 MHz, $CDCl_3$) of **11**

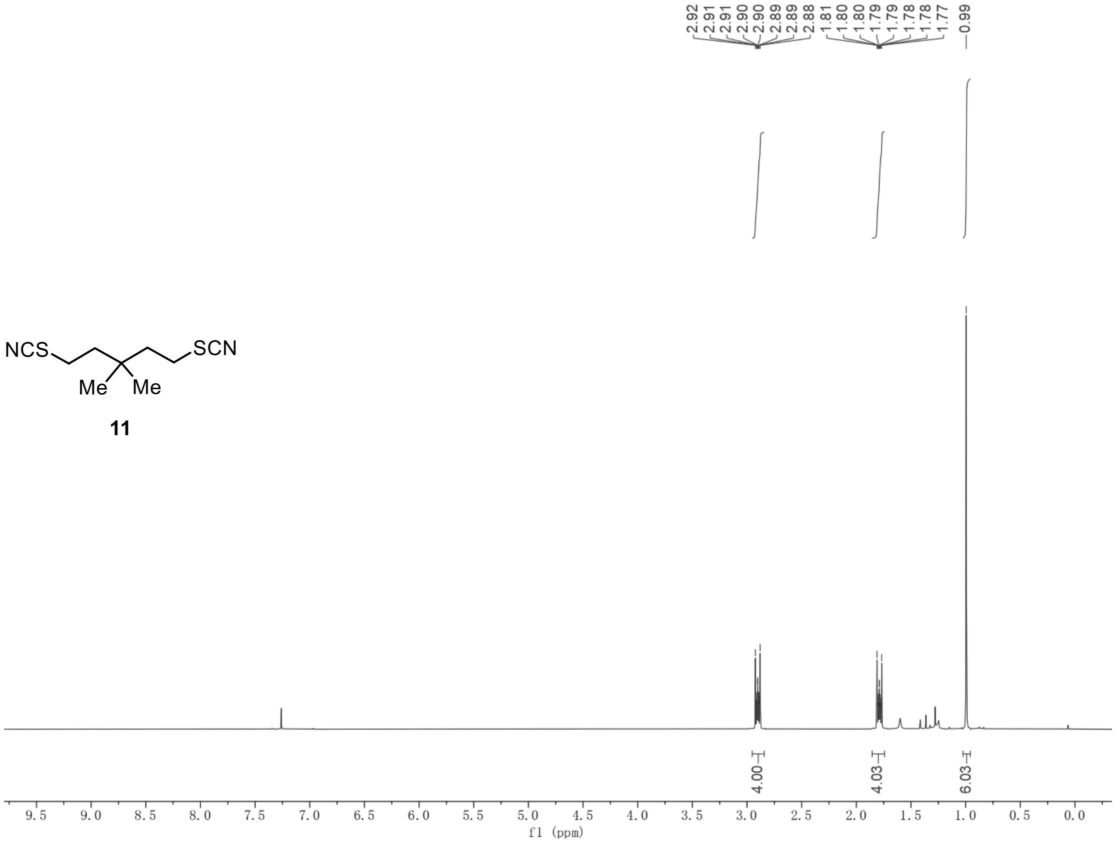

^{13}C NMR (100 MHz, $CDCl_3$) of **11**

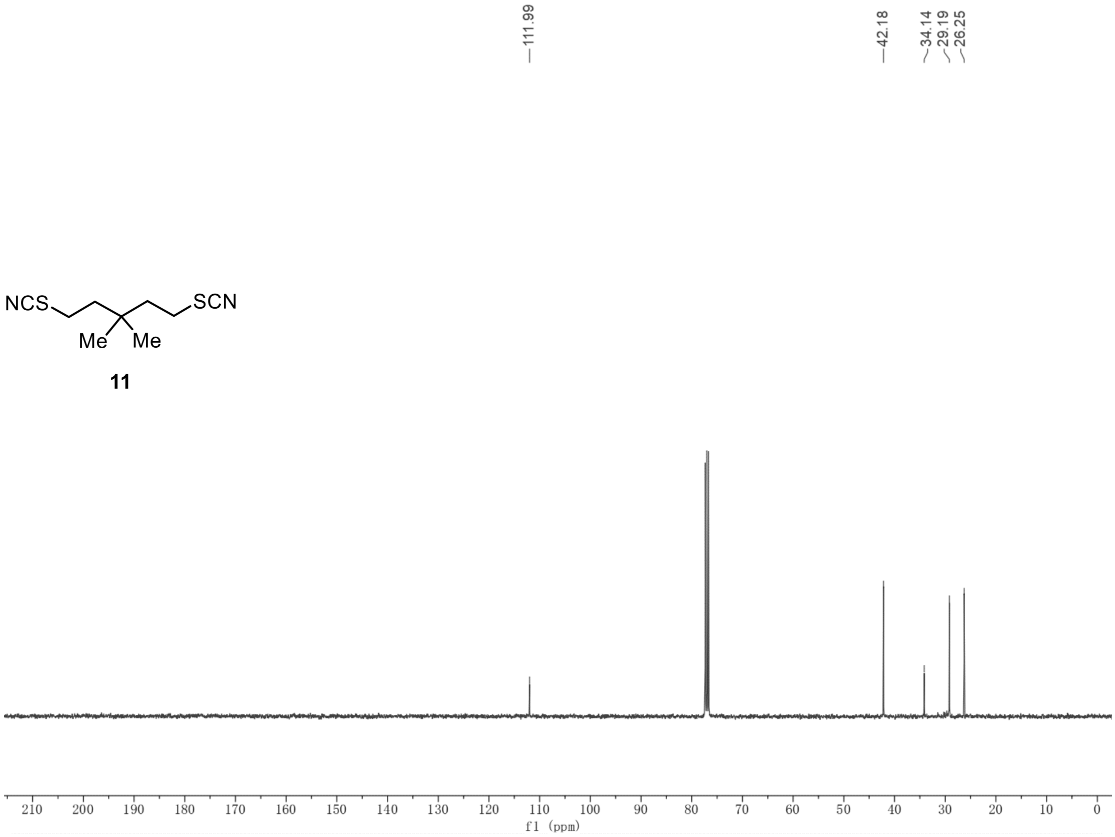

^{1}H NMR (400 MHz, $CDCl_3$) of **12**

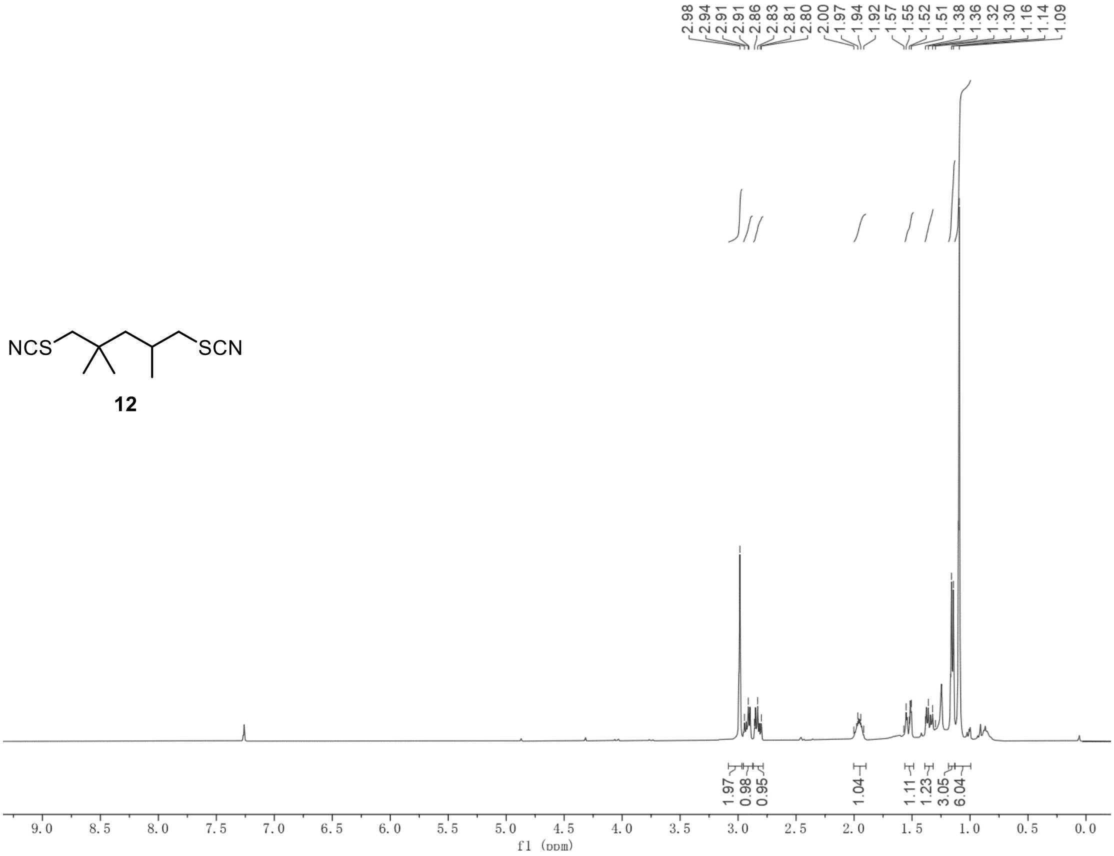

^{13}C NMR (100 MHz, $CDCl_3$) of **12**

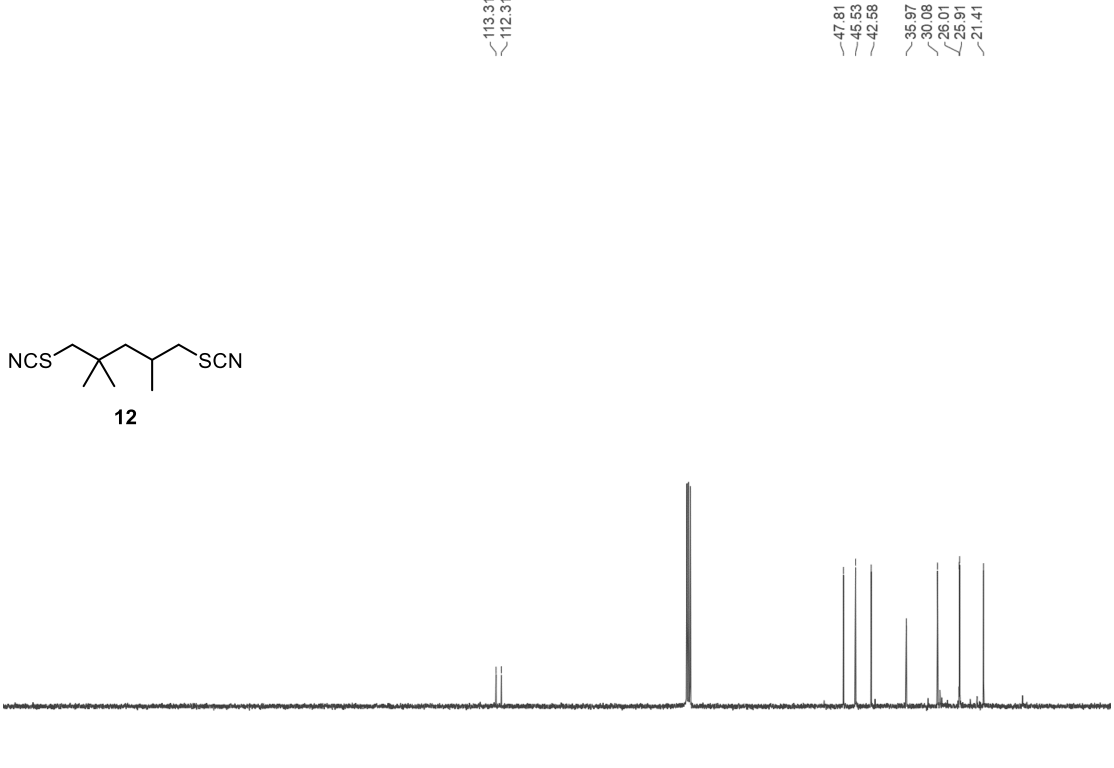

^{1}H NMR (400 MHz, $CDCl_3$) of **13**

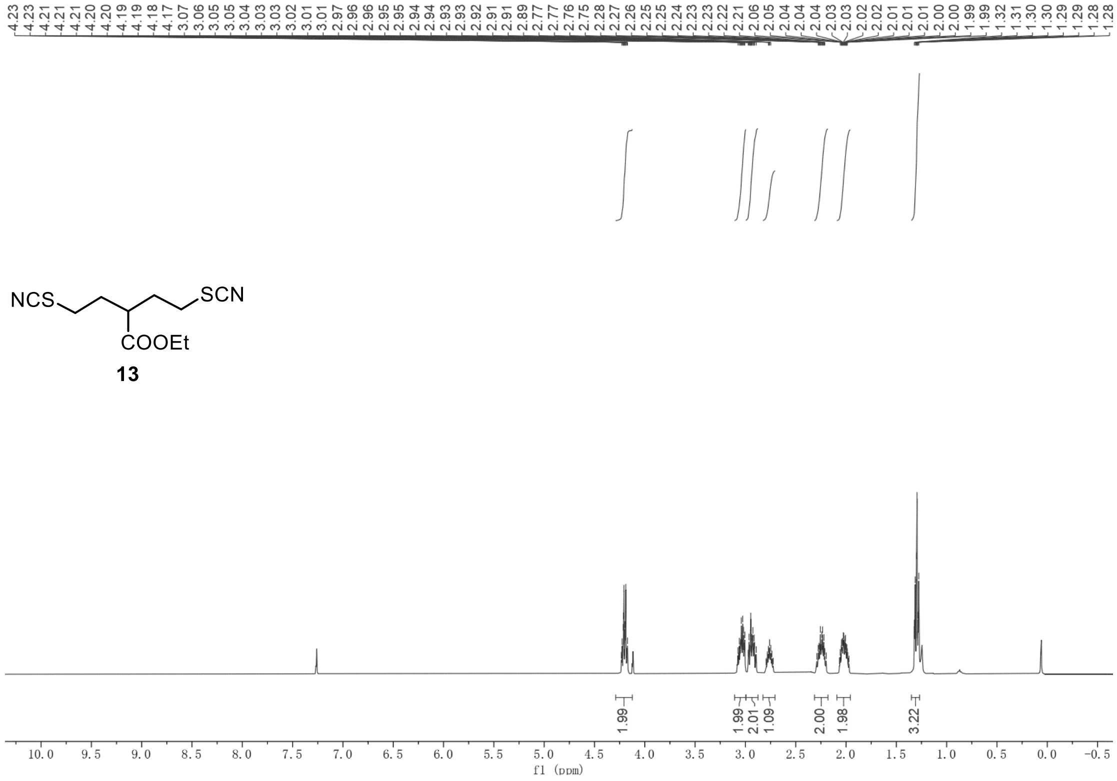

^{13}C NMR (100 MHz, $CDCl_3$) of **13**

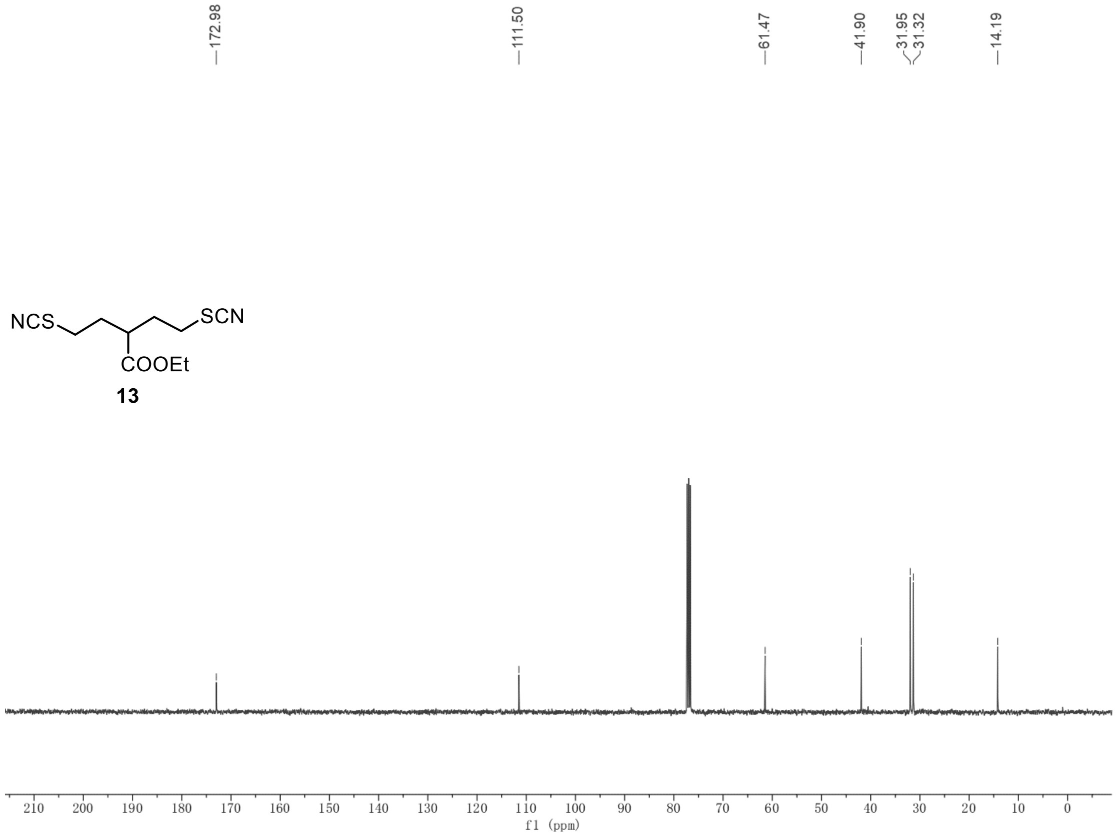

^{1}H NMR (400 MHz, $CDCl_3$) of **14**

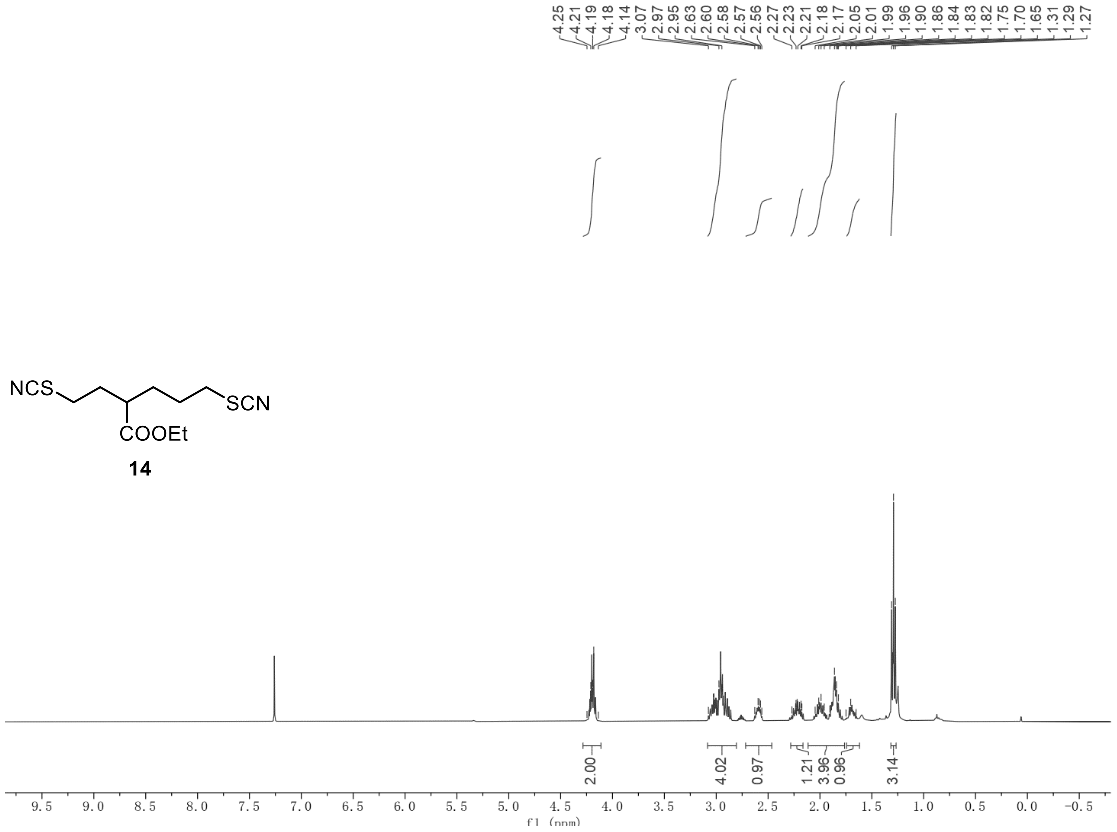

^{13}C NMR (100 MHz, $CDCl_3$) of **14**

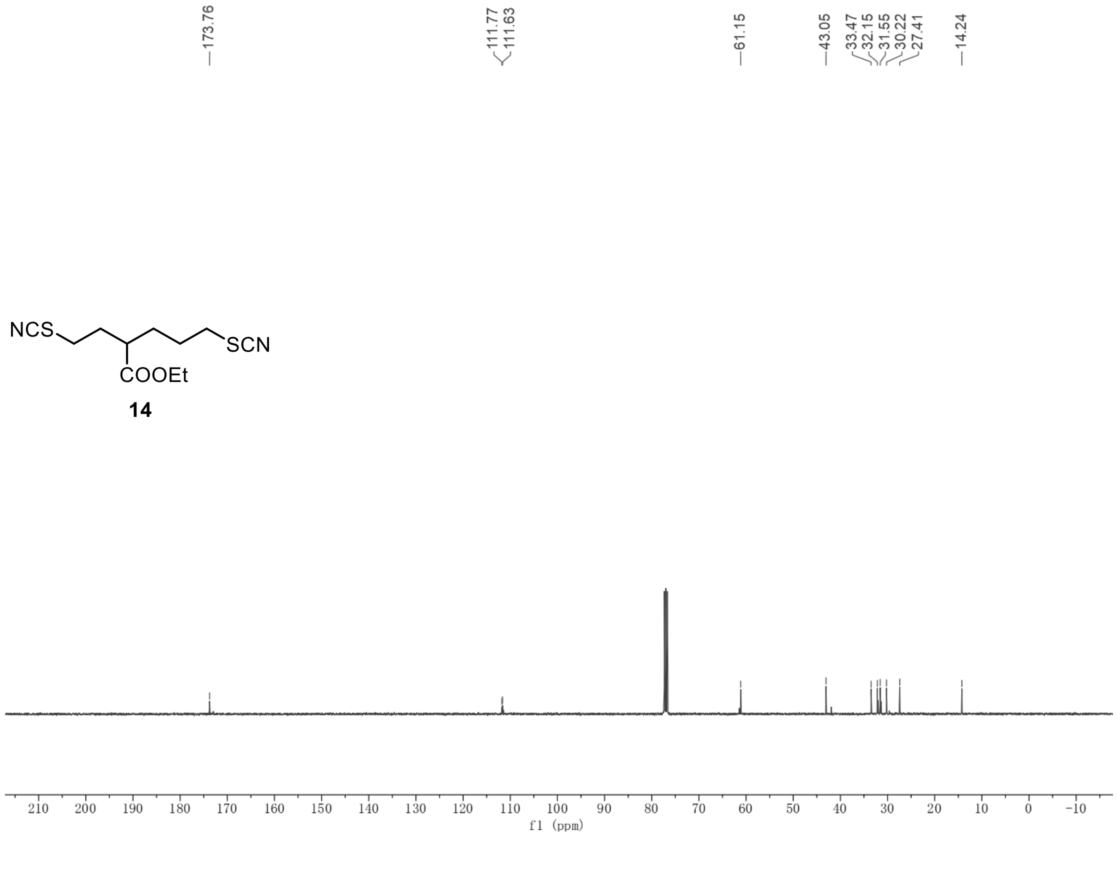

^{1}H NMR (400 MHz, $CDCl_3$) of **15**

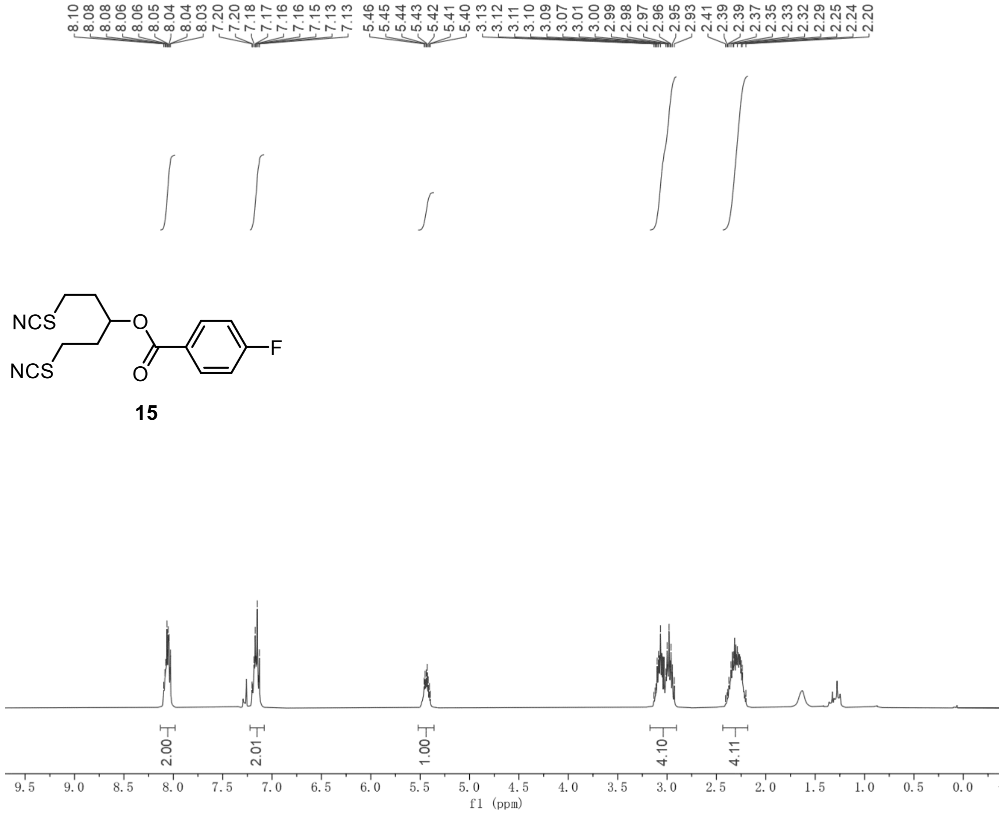

^{13}C NMR (100 MHz, $CDCl_3$) of **15**

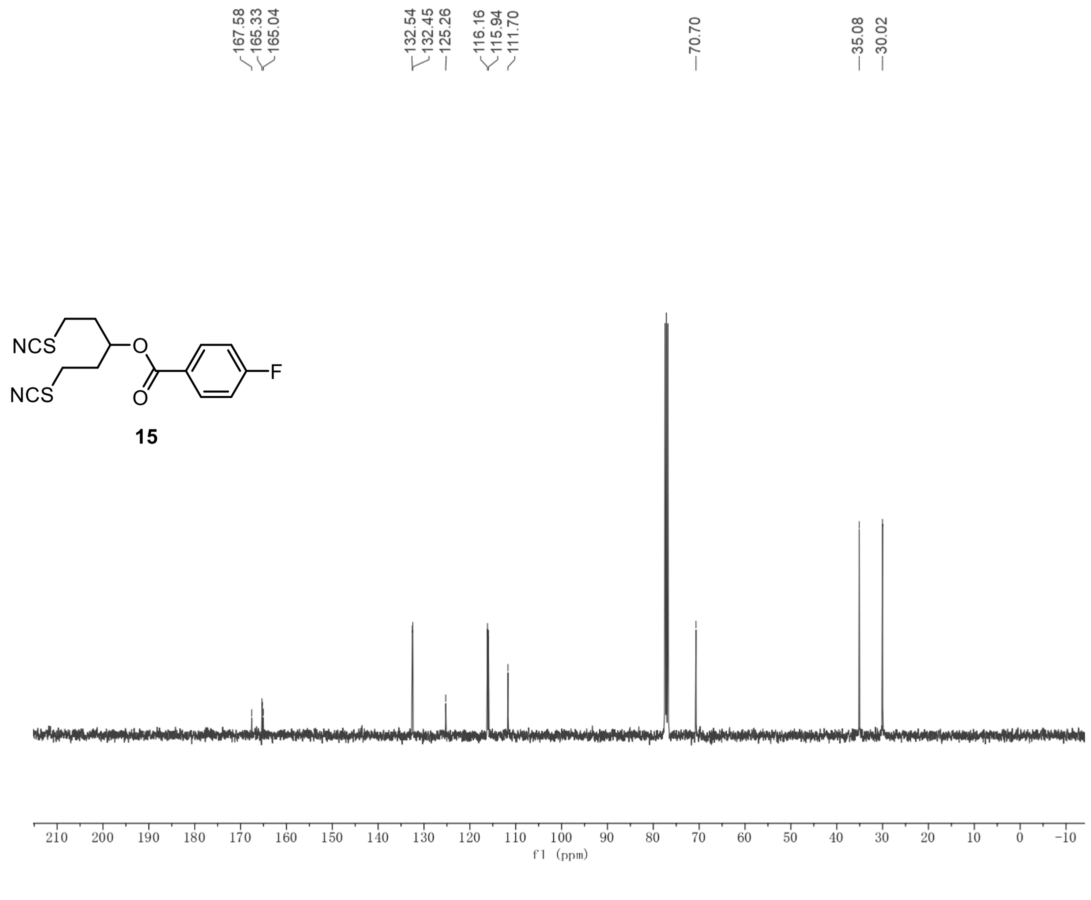

^{19}F NMR (376 MHz, $CDCl_3$) of **15**

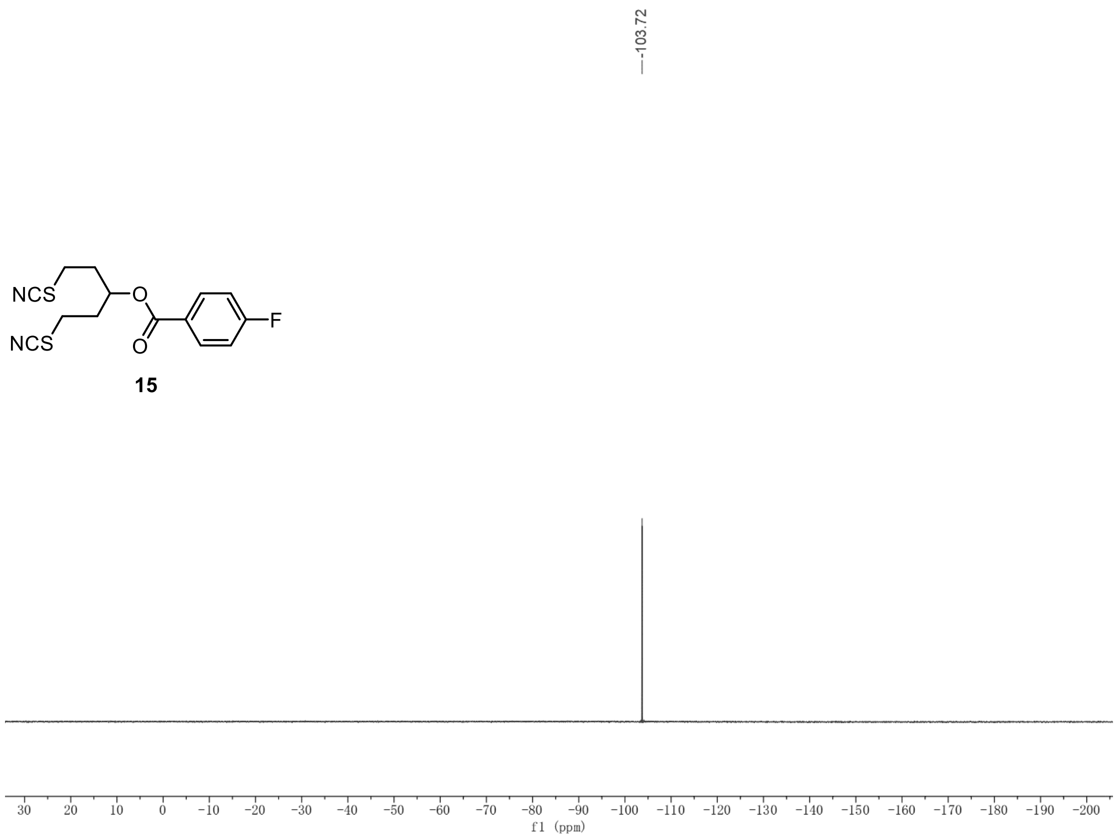

15

^{1}H NMR (400 MHz, $CDCl_3$) of **16**

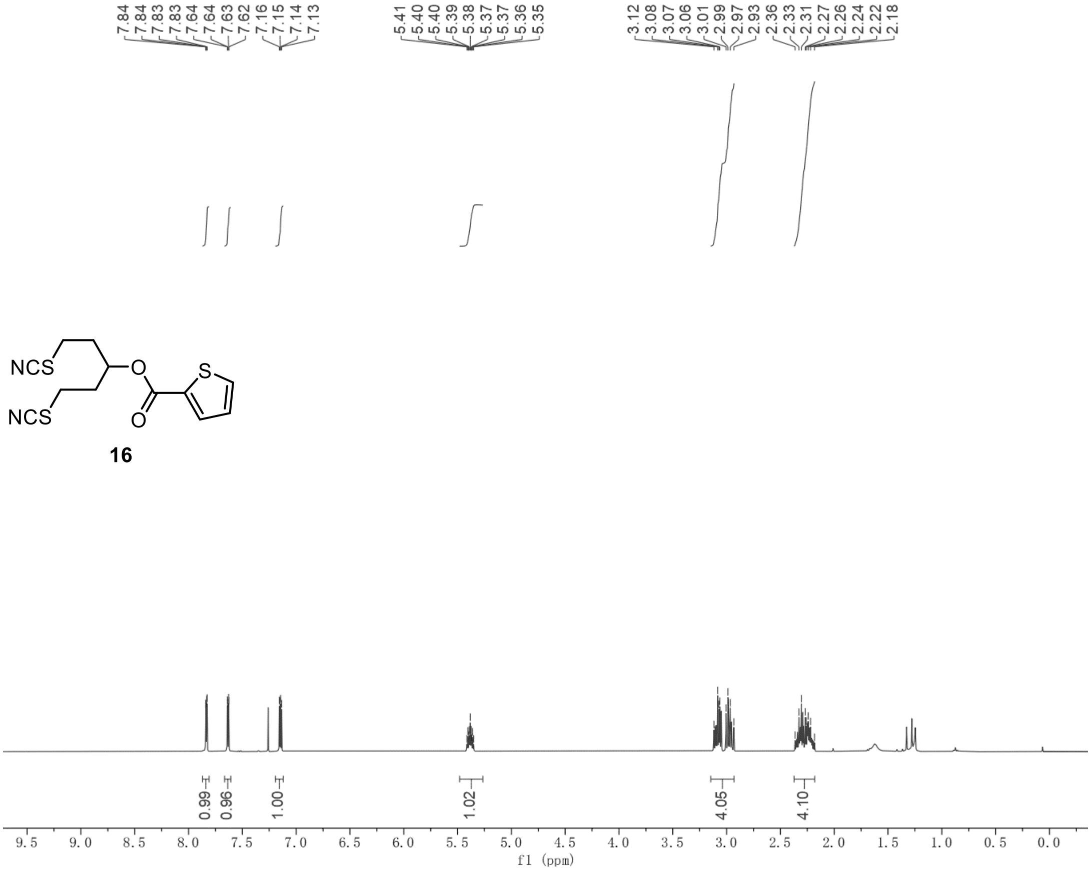

16

^{13}C NMR (100 MHz, $CDCl_3$) of **16**

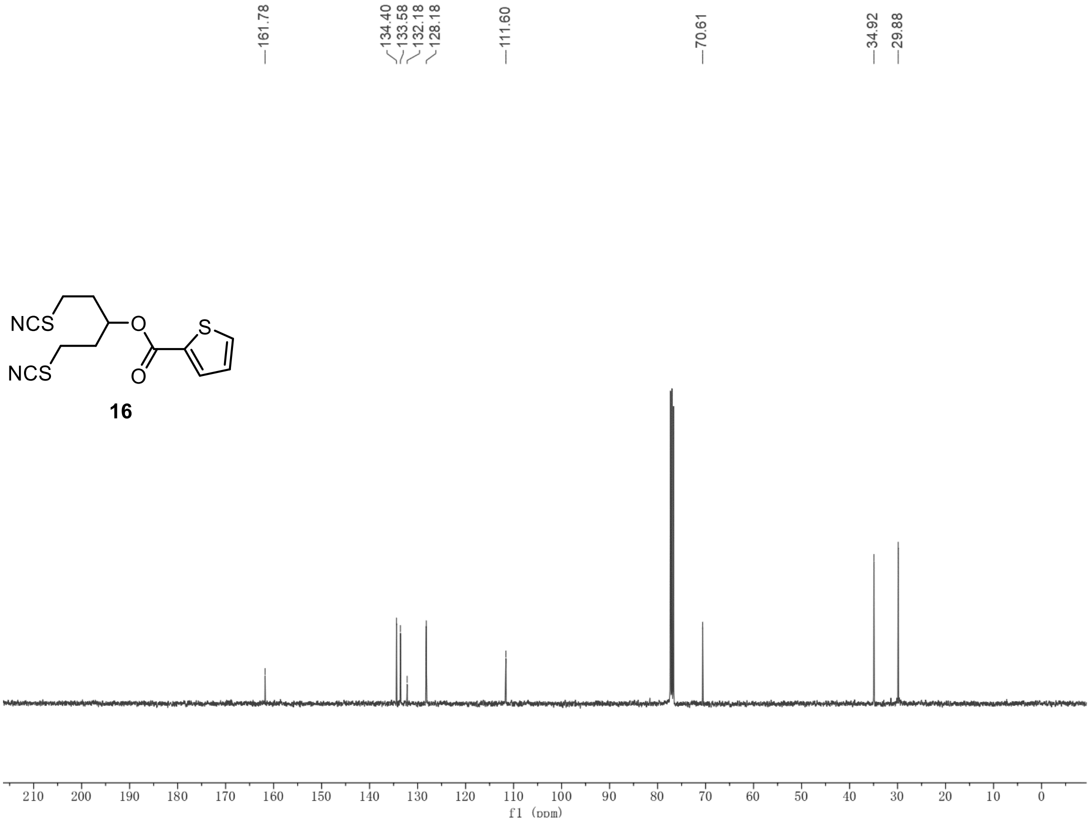

16

^{1}H NMR (400 MHz, $CDCl_3$) of **17**

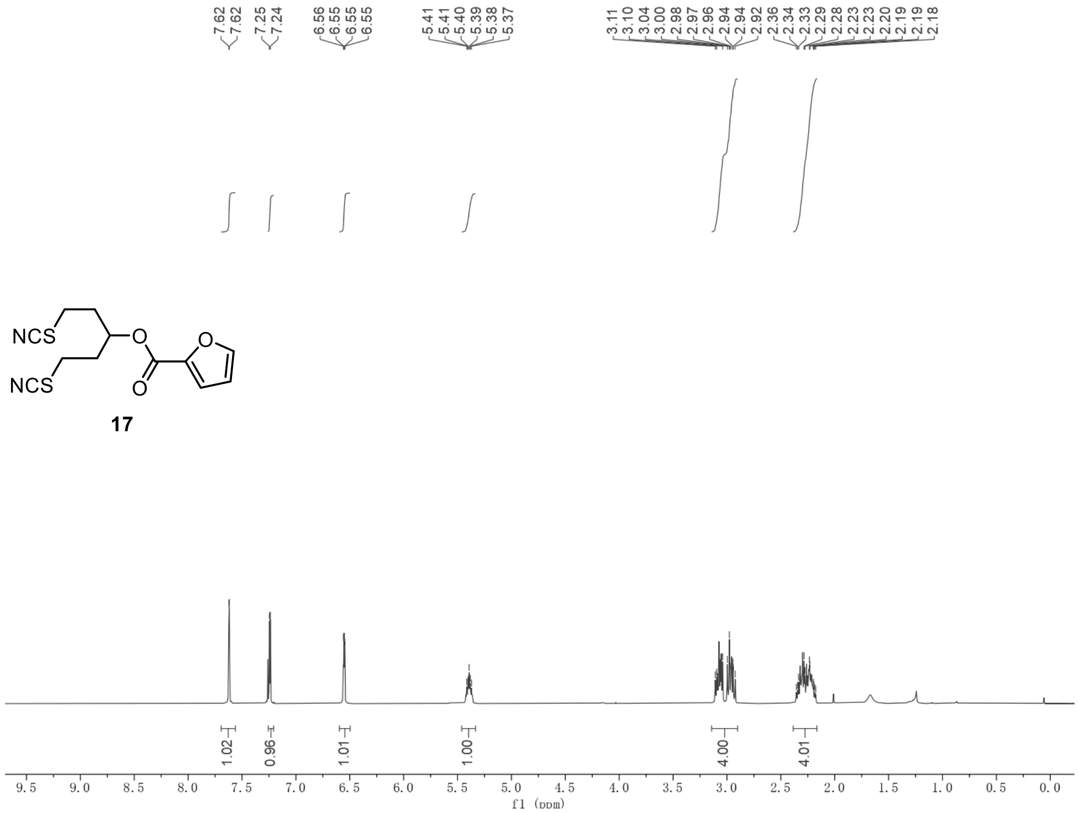

^{13}C NMR (100 MHz, $CDCl_3$) of **17**

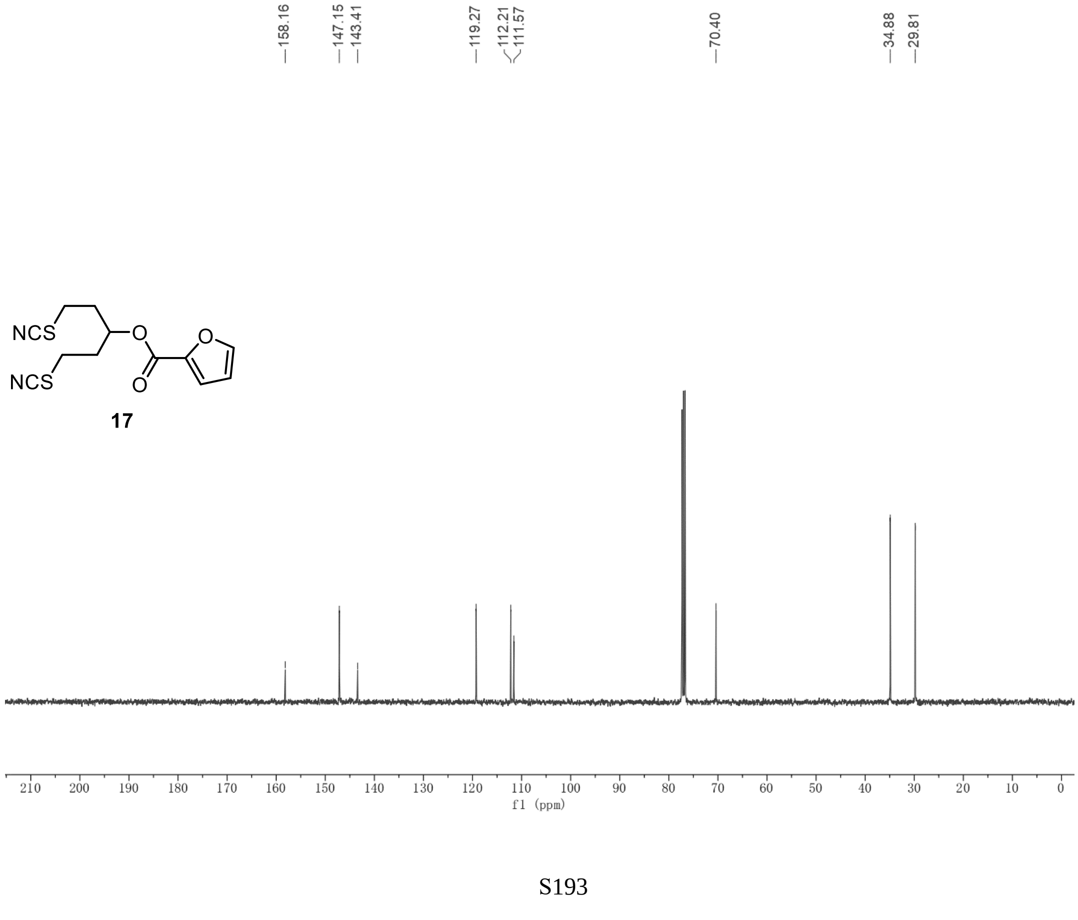

^{1}H NMR (400 MHz, $CDCl_3$) of **18**

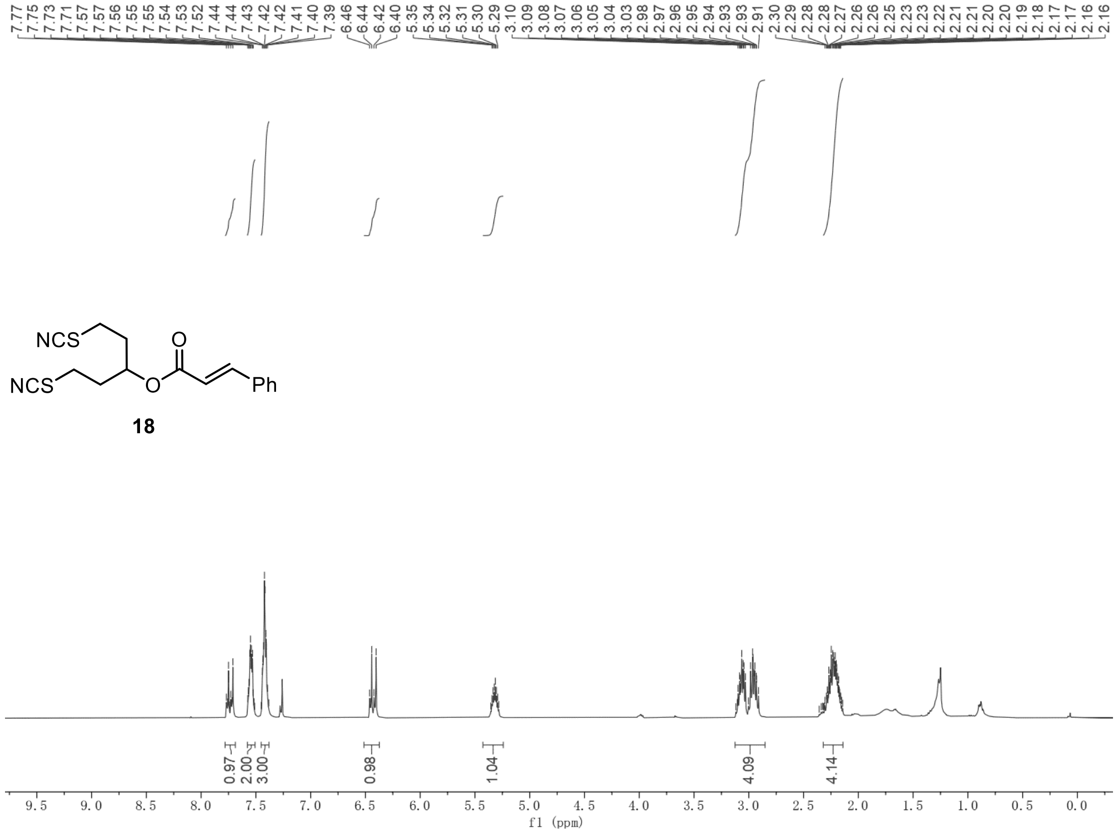

^{13}C NMR (100 MHz, $CDCl_3$) of **18**

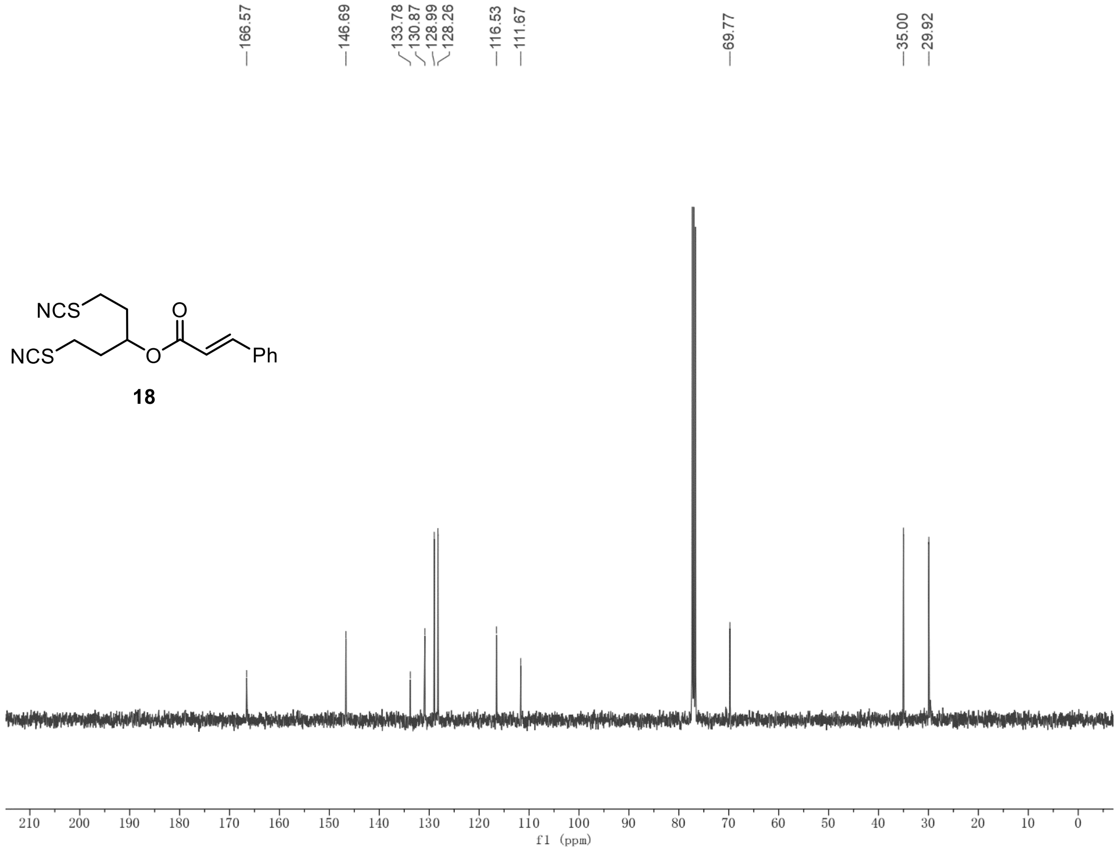

^{1}H NMR (400 MHz, $CDCl_3$) of **19**

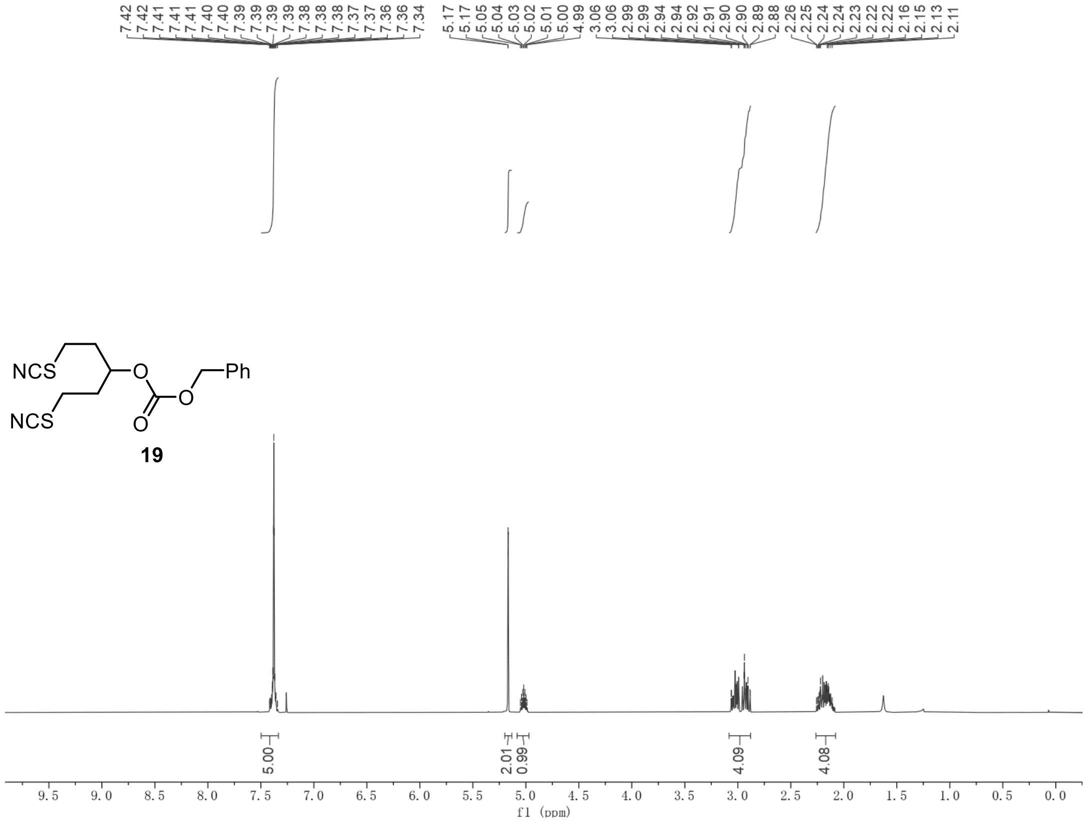

^{13}C NMR (100 MHz, $CDCl_3$) of **19**

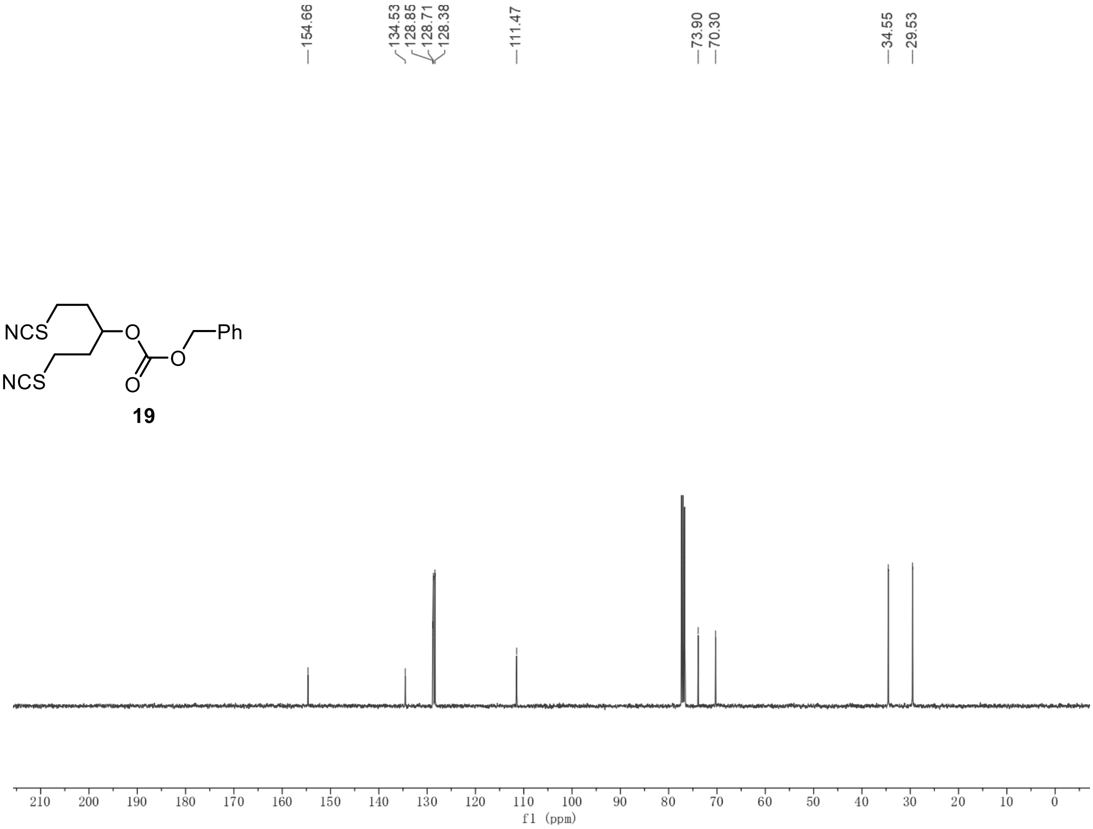

^{1}H NMR (400 MHz, $CDCl_3$) of **20**

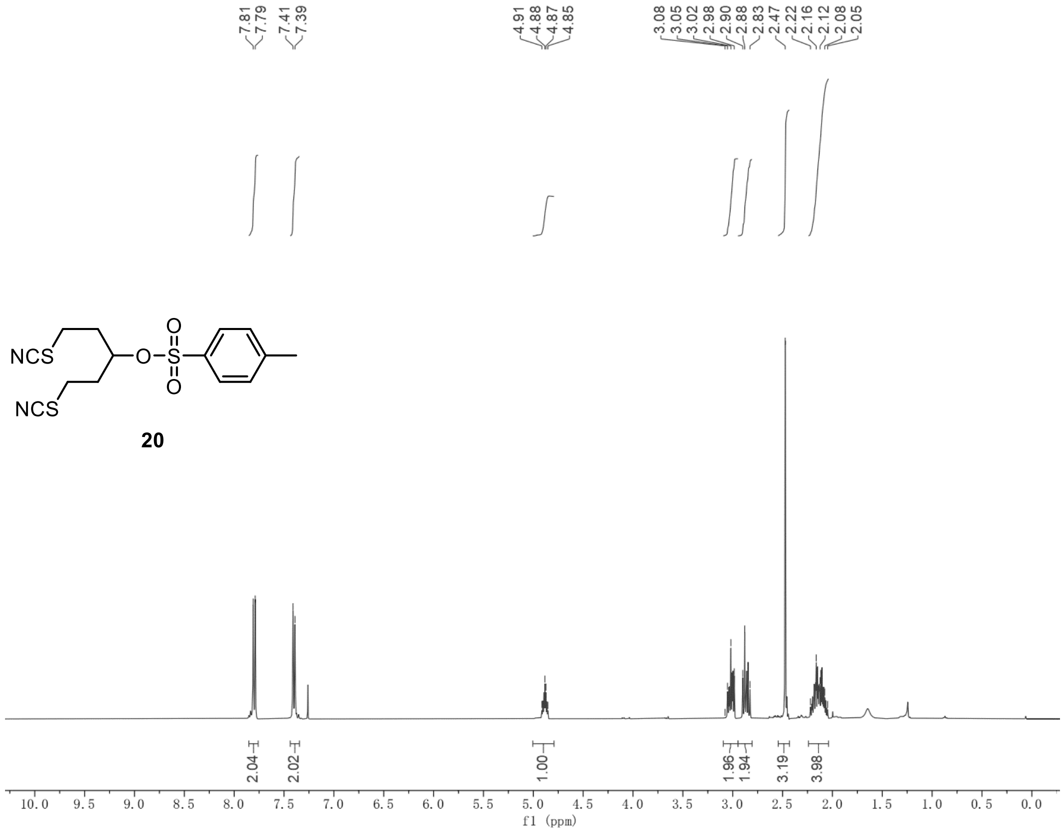

^{13}C NMR (100 MHz, $CDCl_3$) of **20**

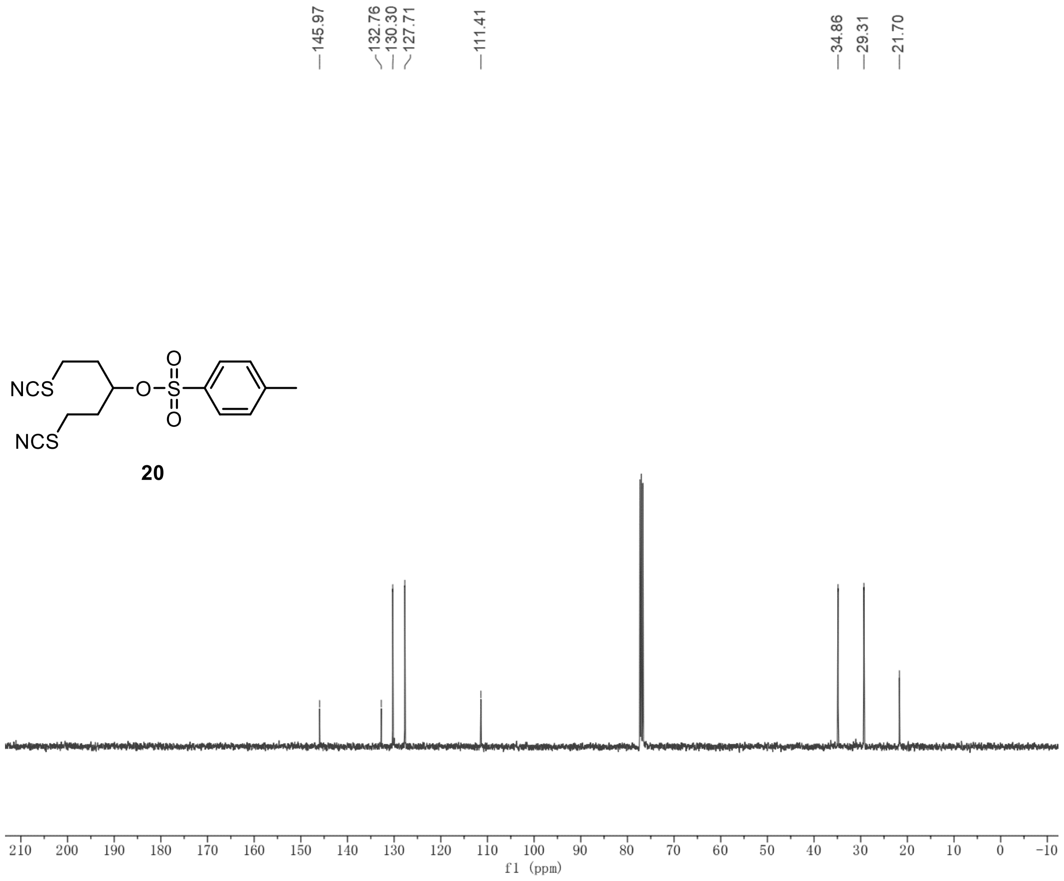

^{1}H NMR (400 MHz, $CDCl_3$) of **21**

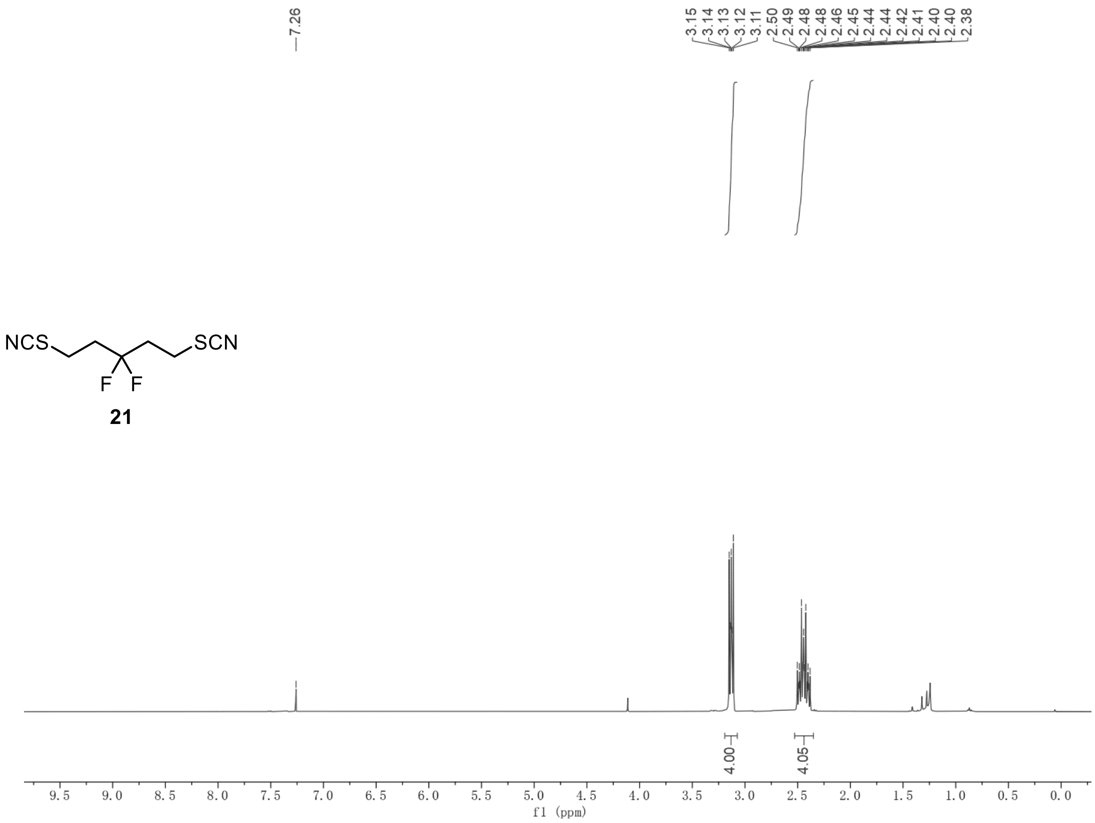

^{13}C NMR (100 MHz, $CDCl_3$) of **21**

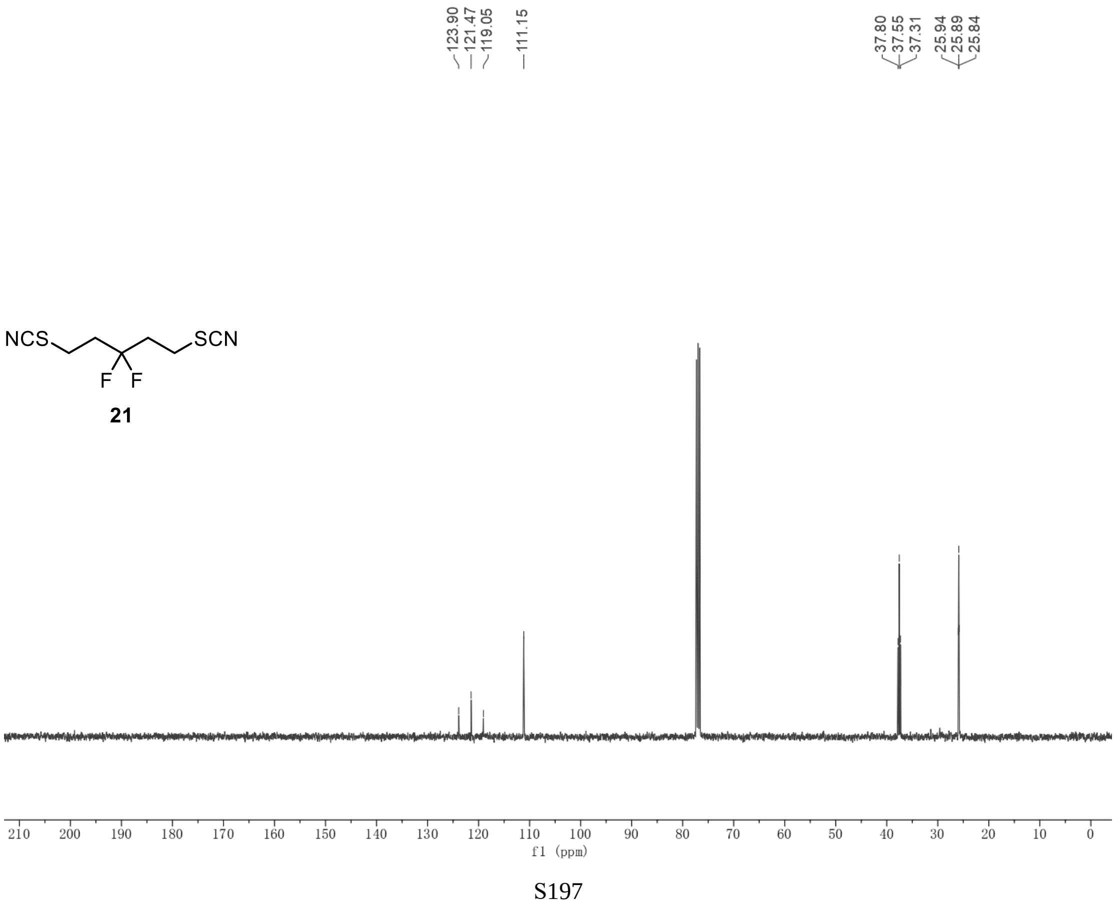

^{19}F NMR (376 MHz, $CDCl_3$) of **21**

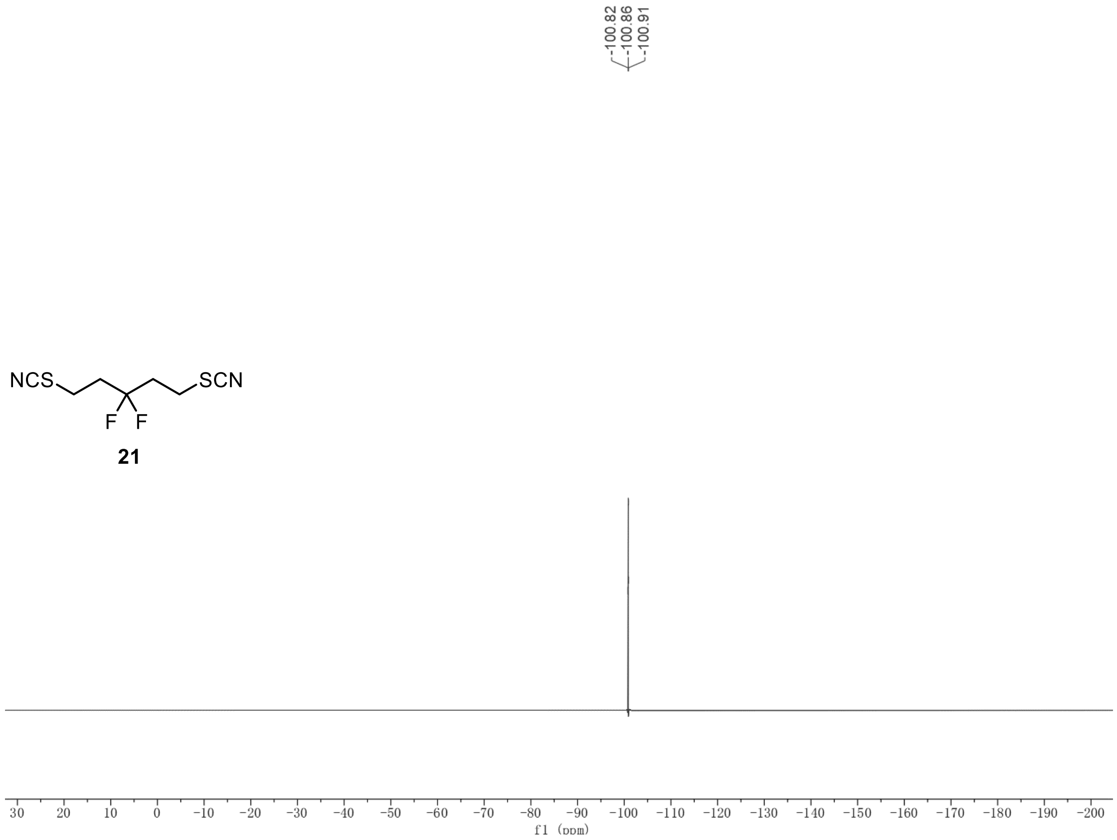

^{1}H NMR (400 MHz, $CDCl_3$) of **22**

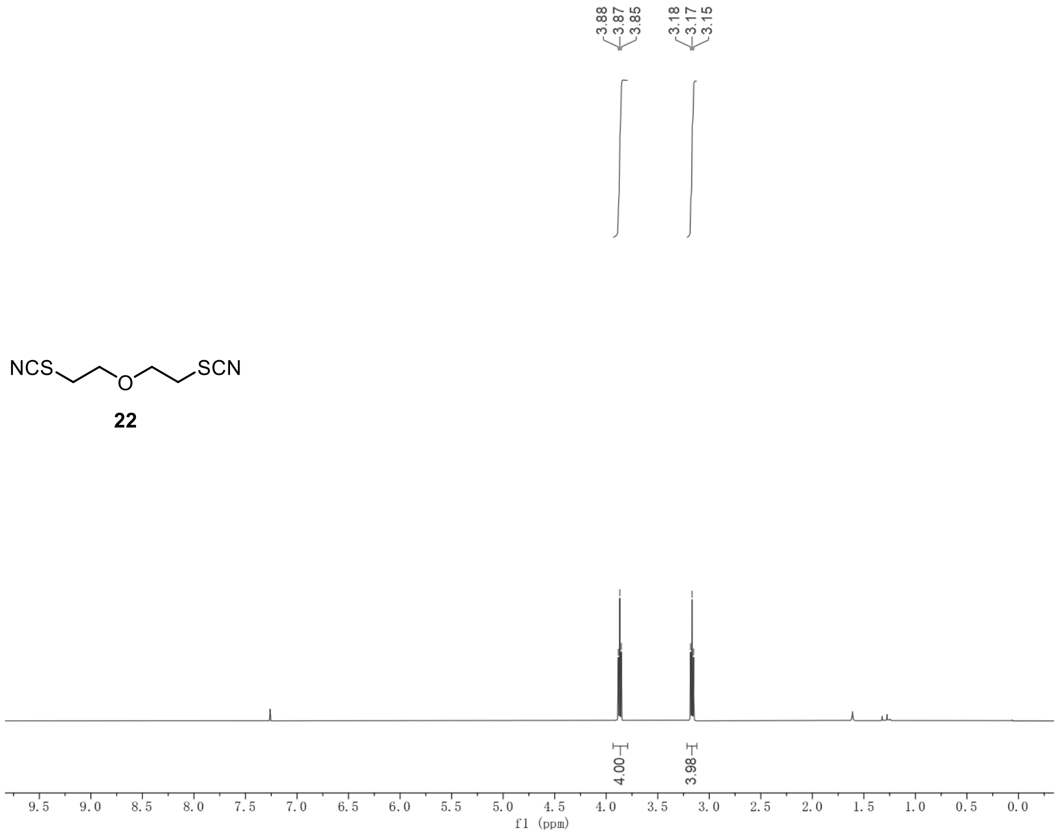

22

^{13}C NMR (100 MHz, $CDCl_3$) of **22**

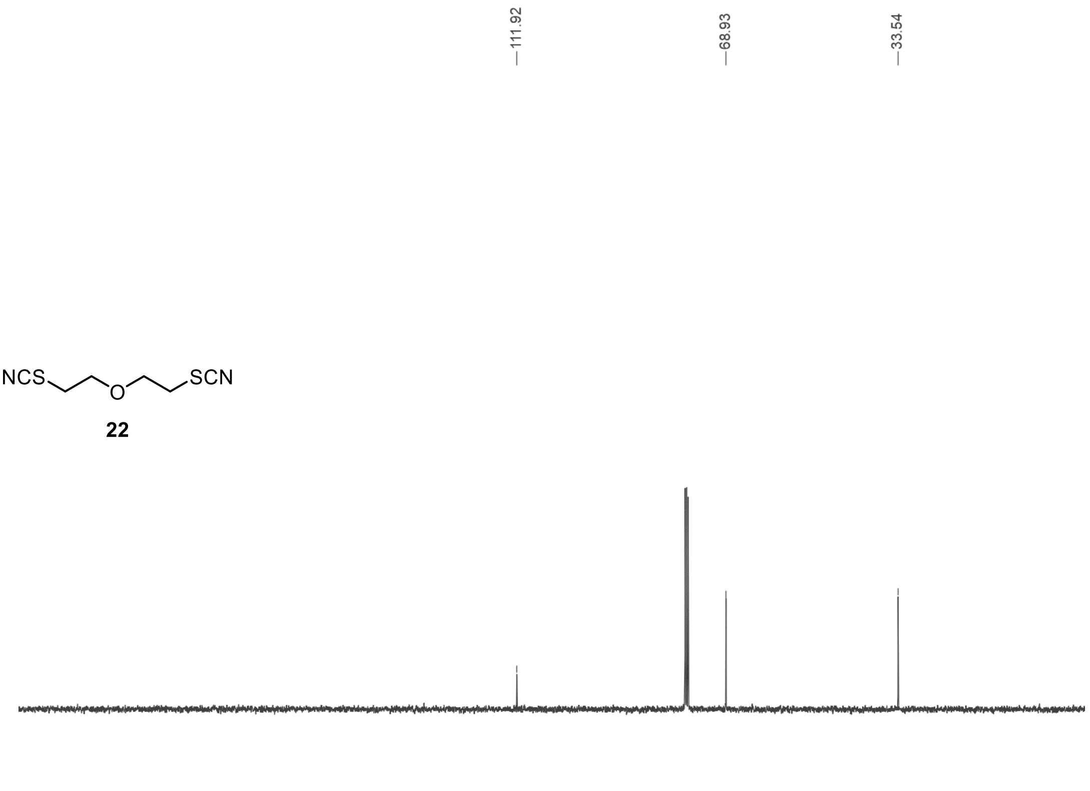

22

^{1}H NMR (400 MHz, $CDCl_3$) of **23**

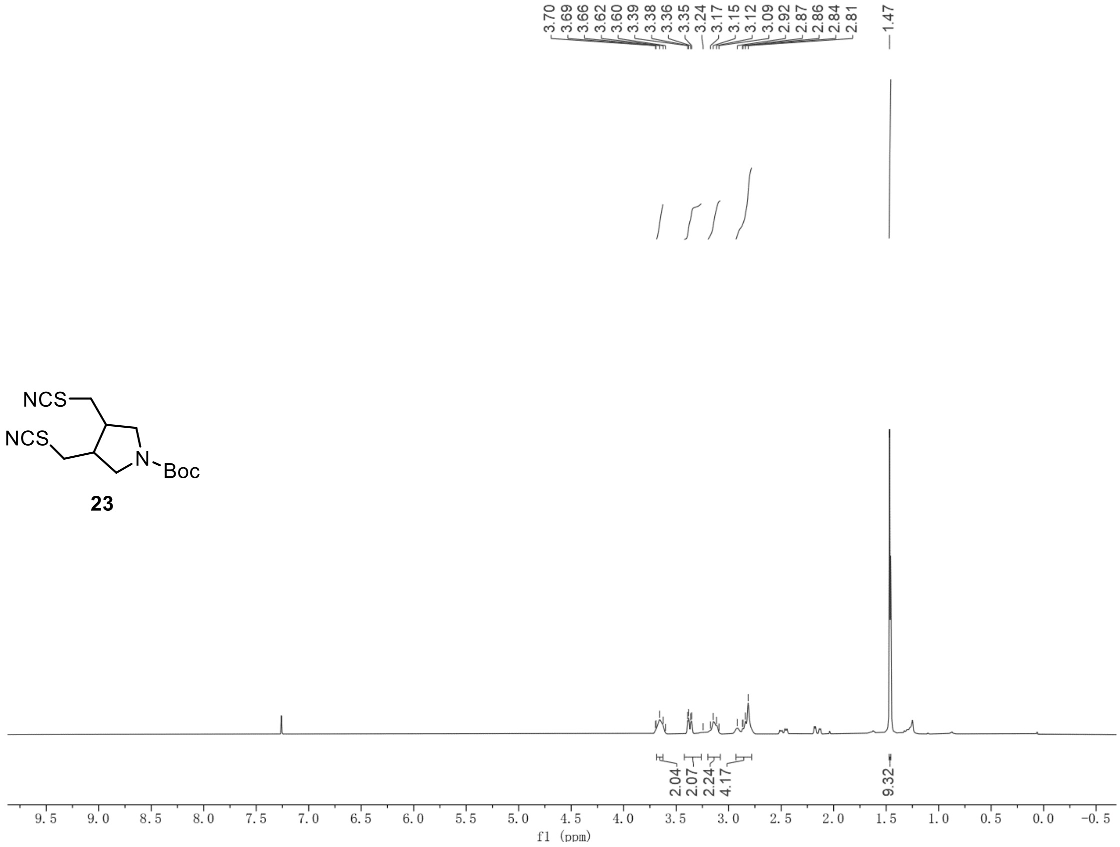

^{13}C NMR (100 MHz, $CDCl_3$) of **23**

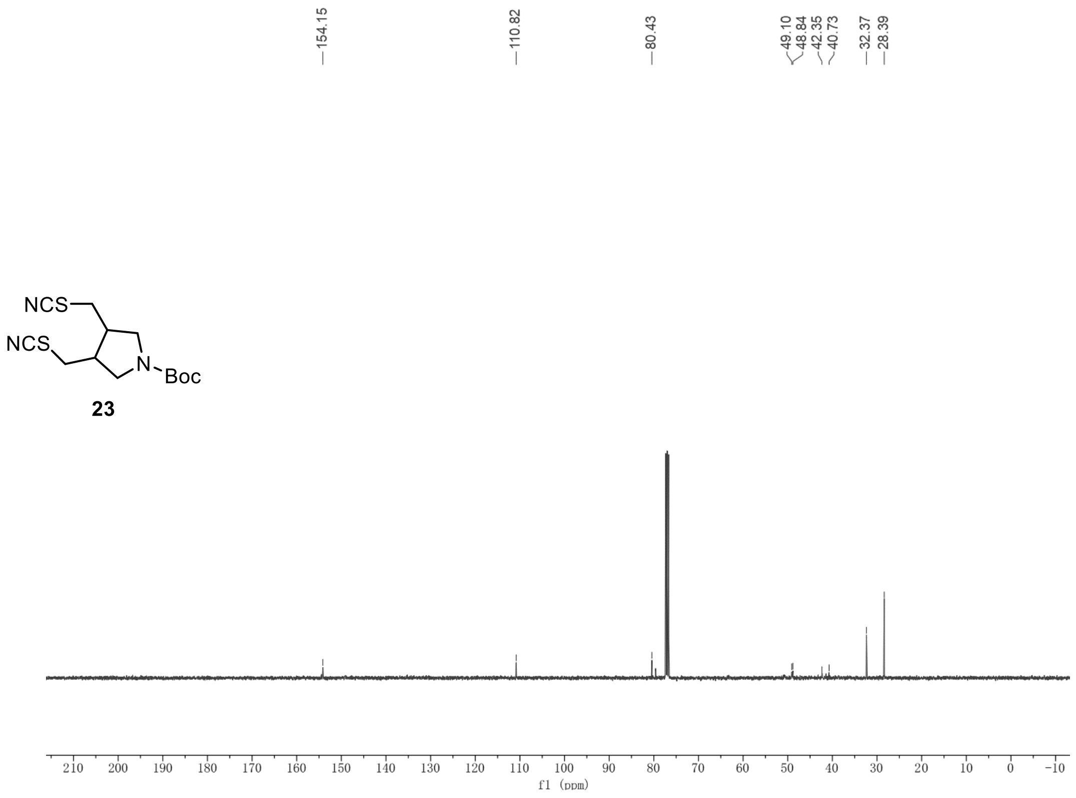

^{1}H NMR (400 MHz, $CDCl_3$) of **24**

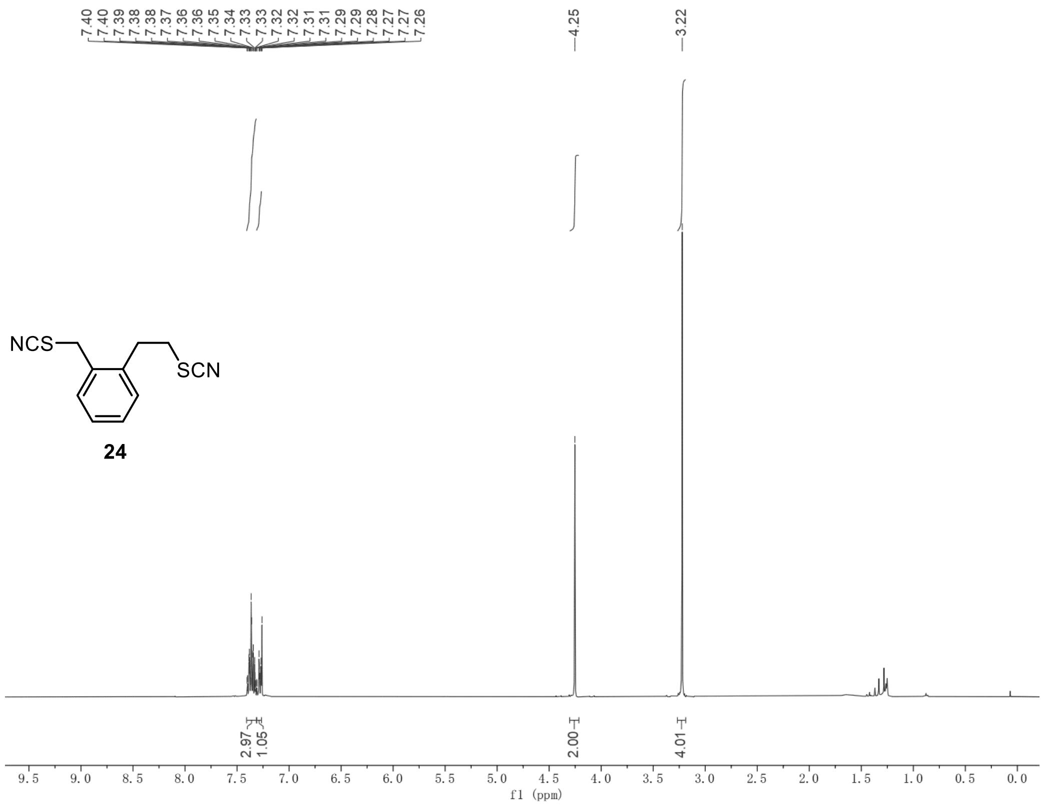

^{13}C NMR (100 MHz, $CDCl_3$) of **24**

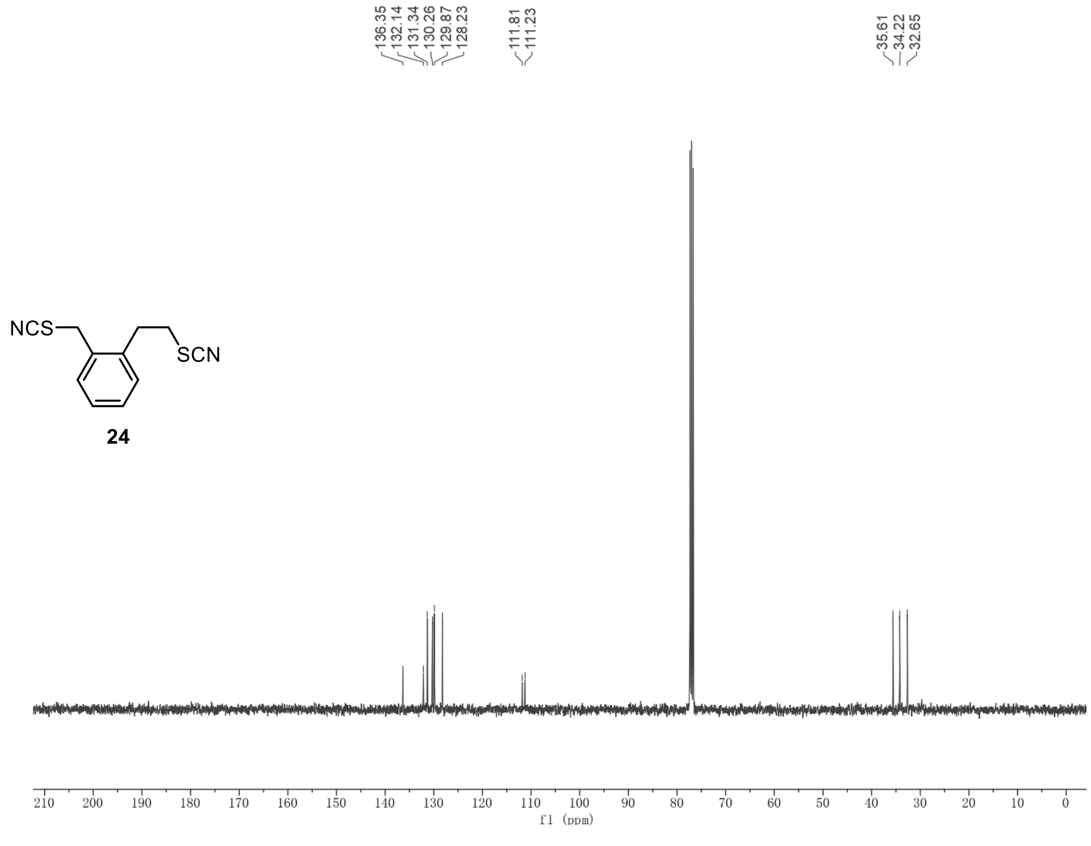

^{1}**H NMR** (400 MHz, $CDCl_3$) of **25**

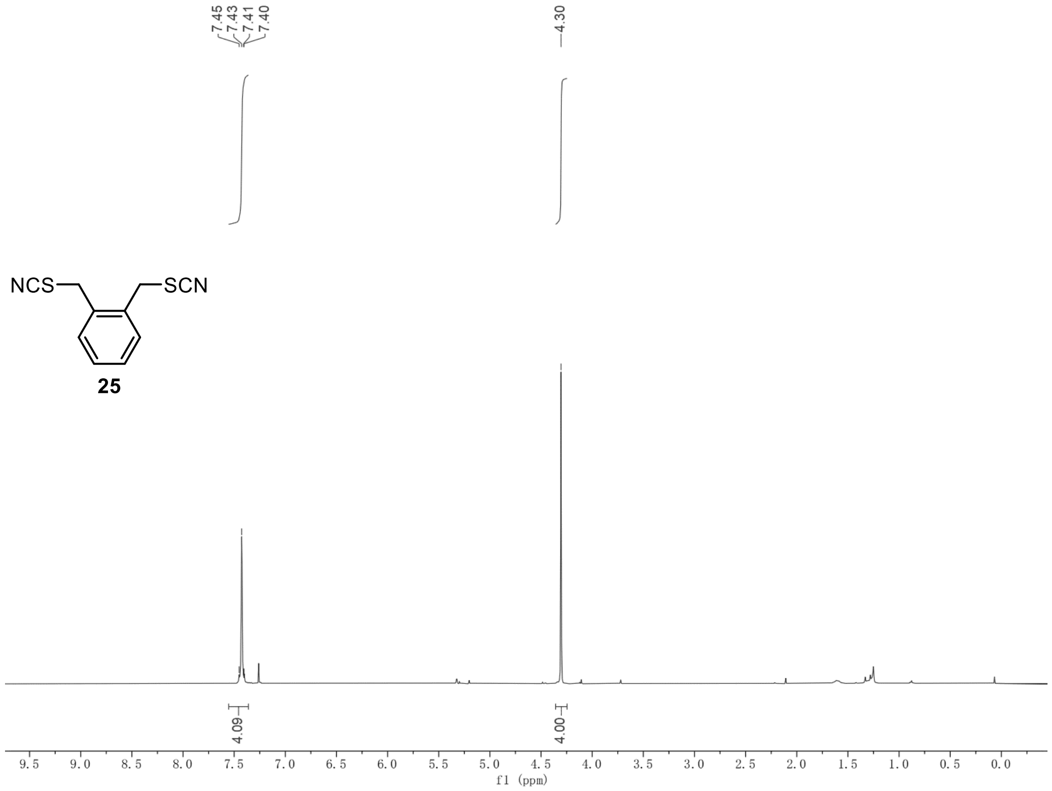

^{13}**C NMR** (100 MHz, $CDCl_3$) of **25**

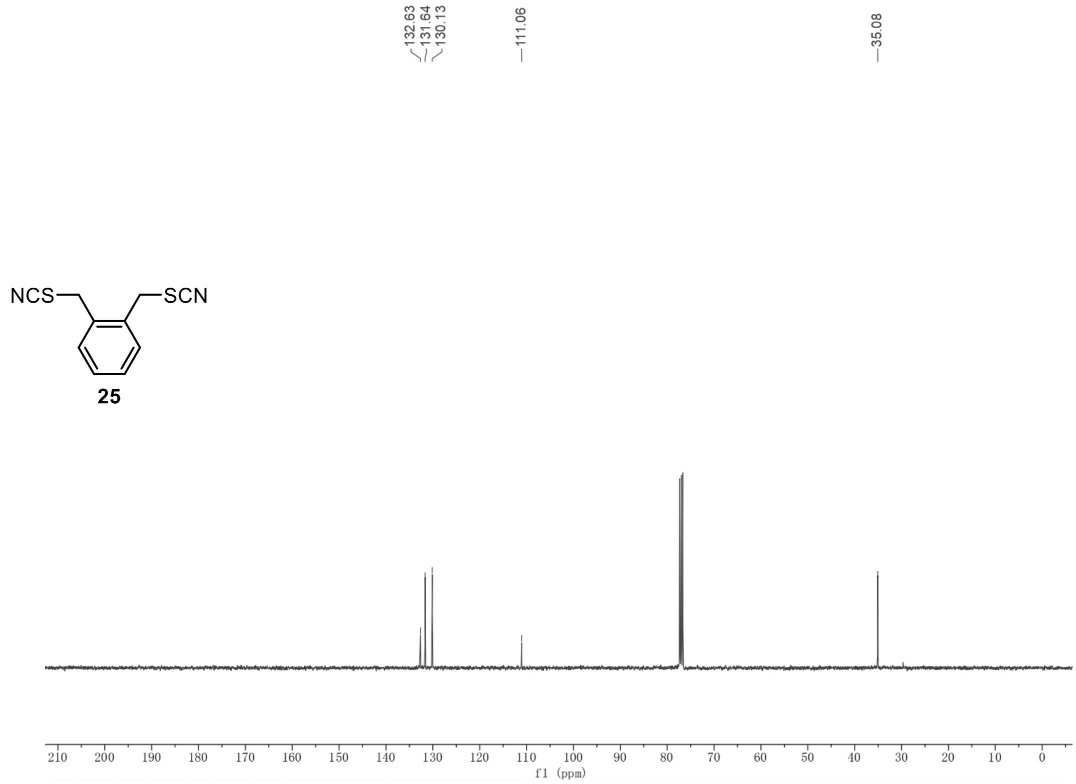

^{1}H NMR (400 MHz, $CDCl_3$) of **26**

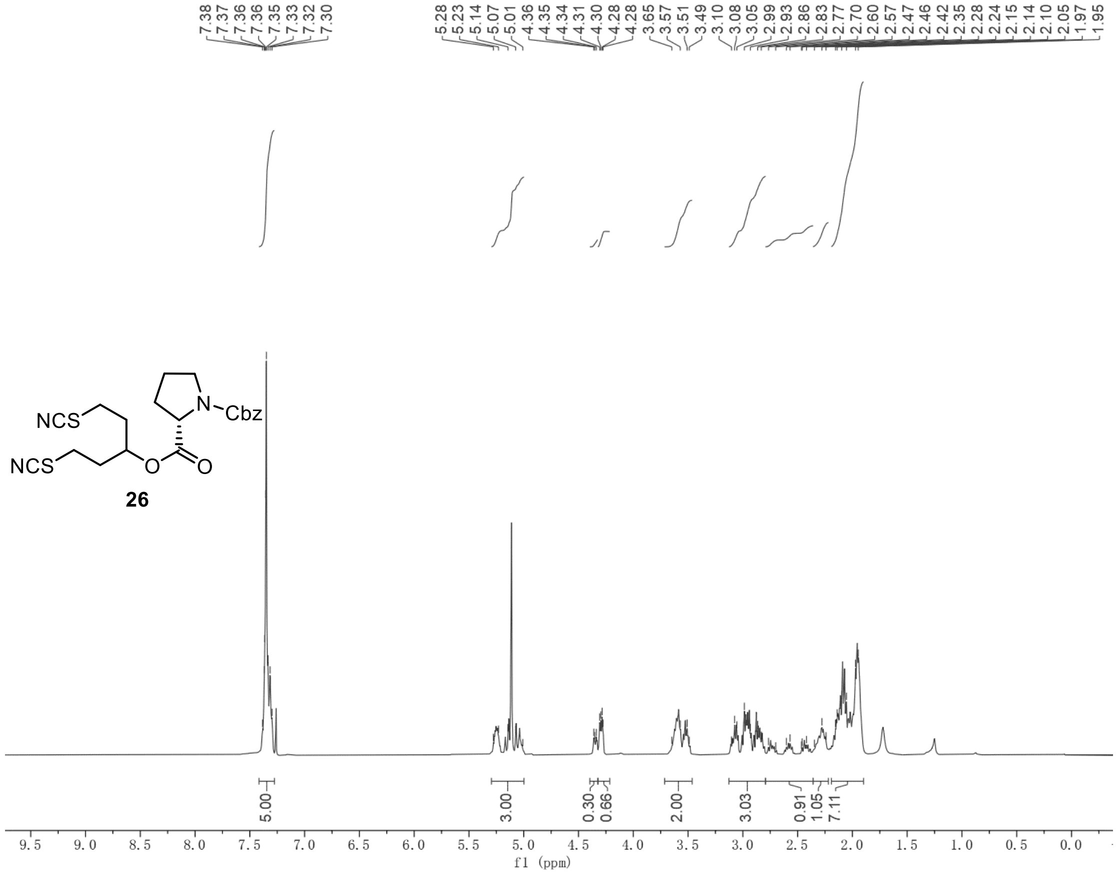

^{13}C NMR (100 MHz, $CDCl_3$) of **26**

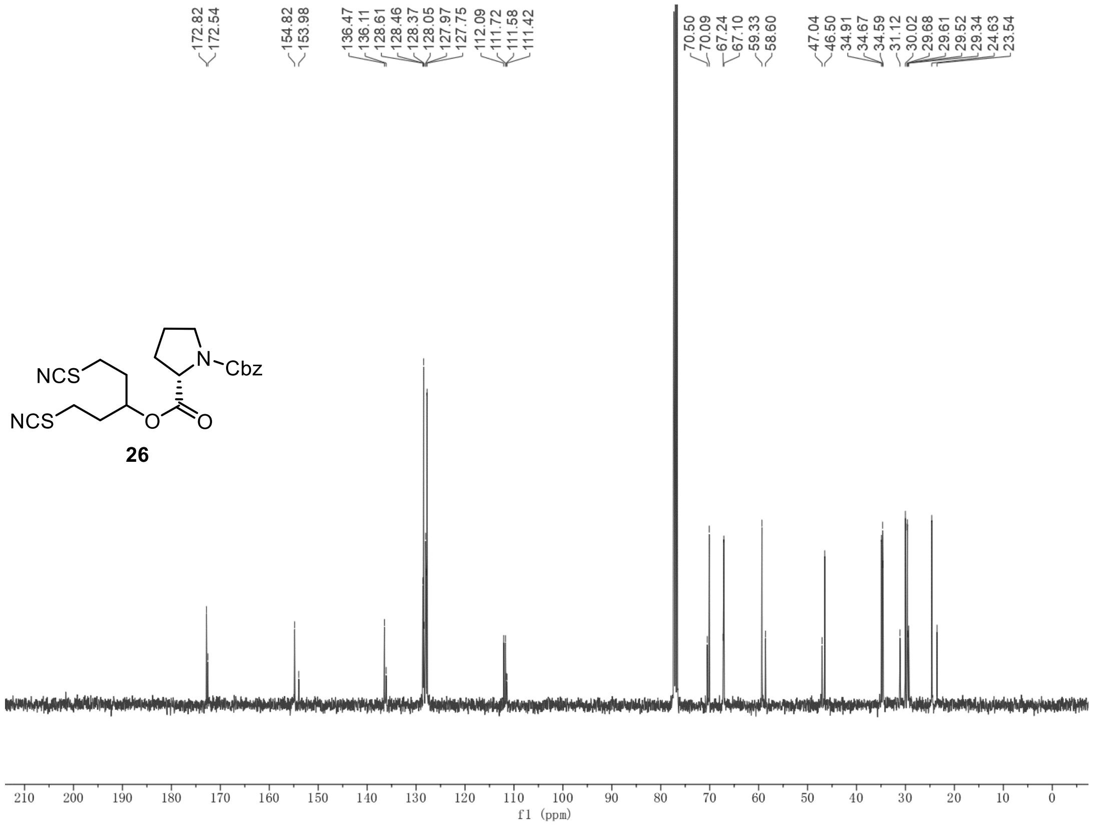

^{1}H NMR (400 MHz, $CDCl_3$) of **27**

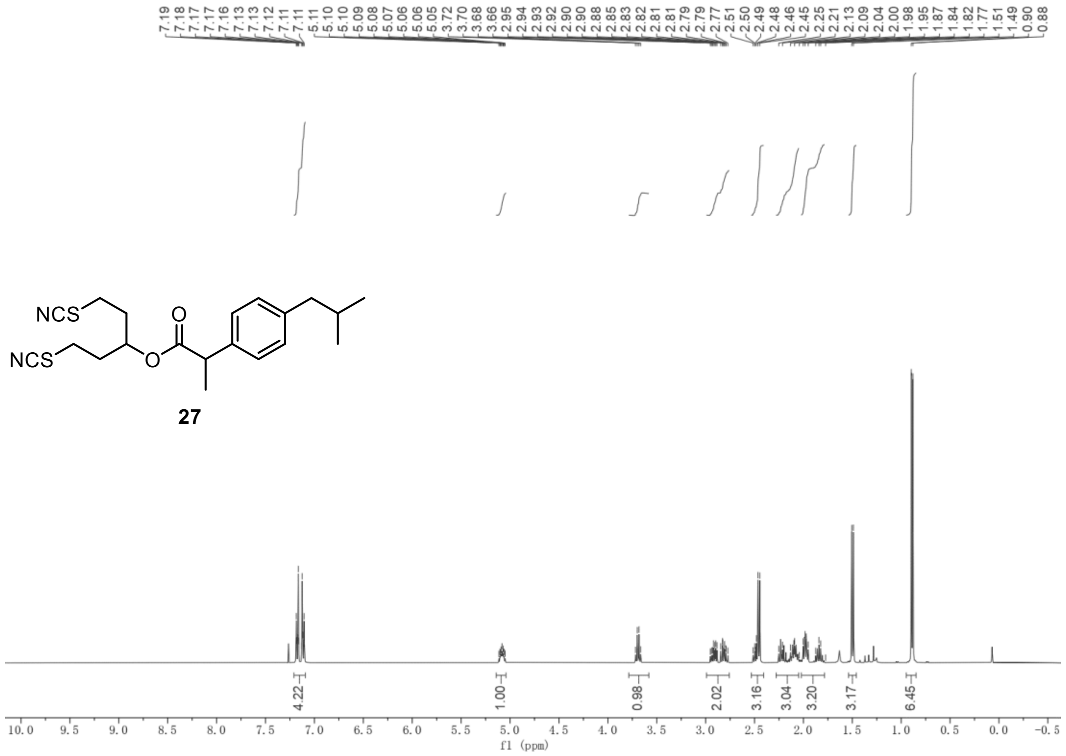

^{13}C NMR (100 MHz, $CDCl_3$) of **27**

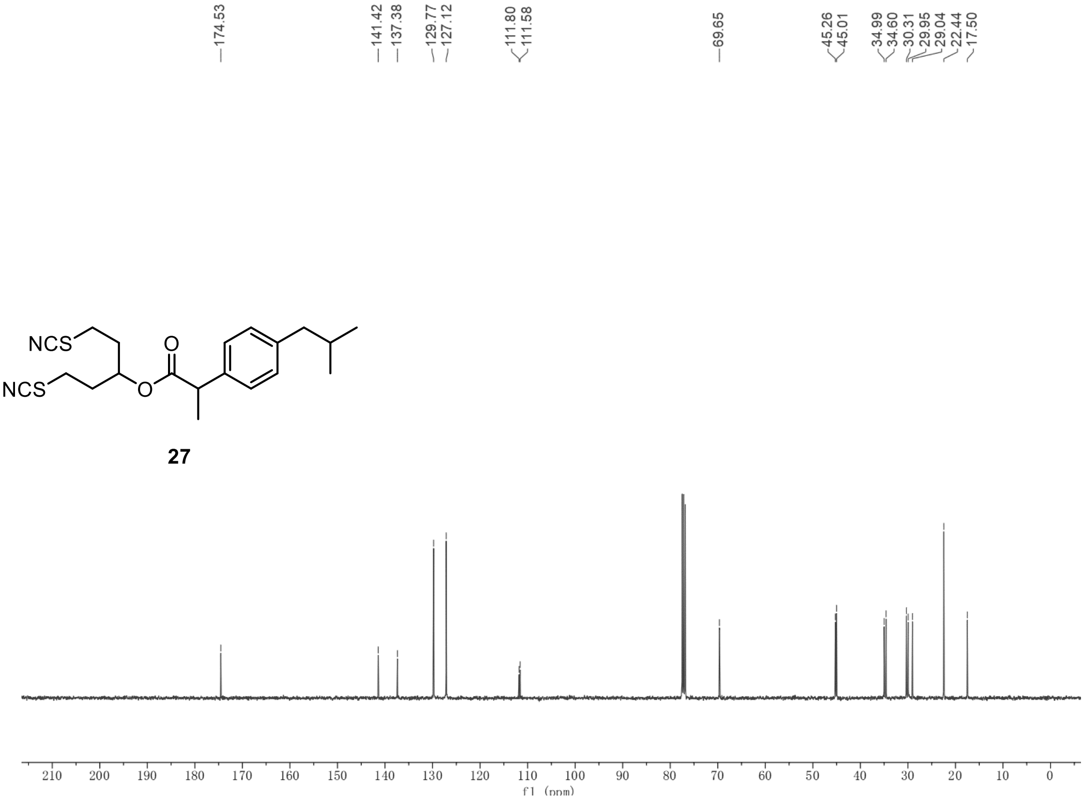

^{1}H NMR (400 MHz, $CDCl_3$) of **28**

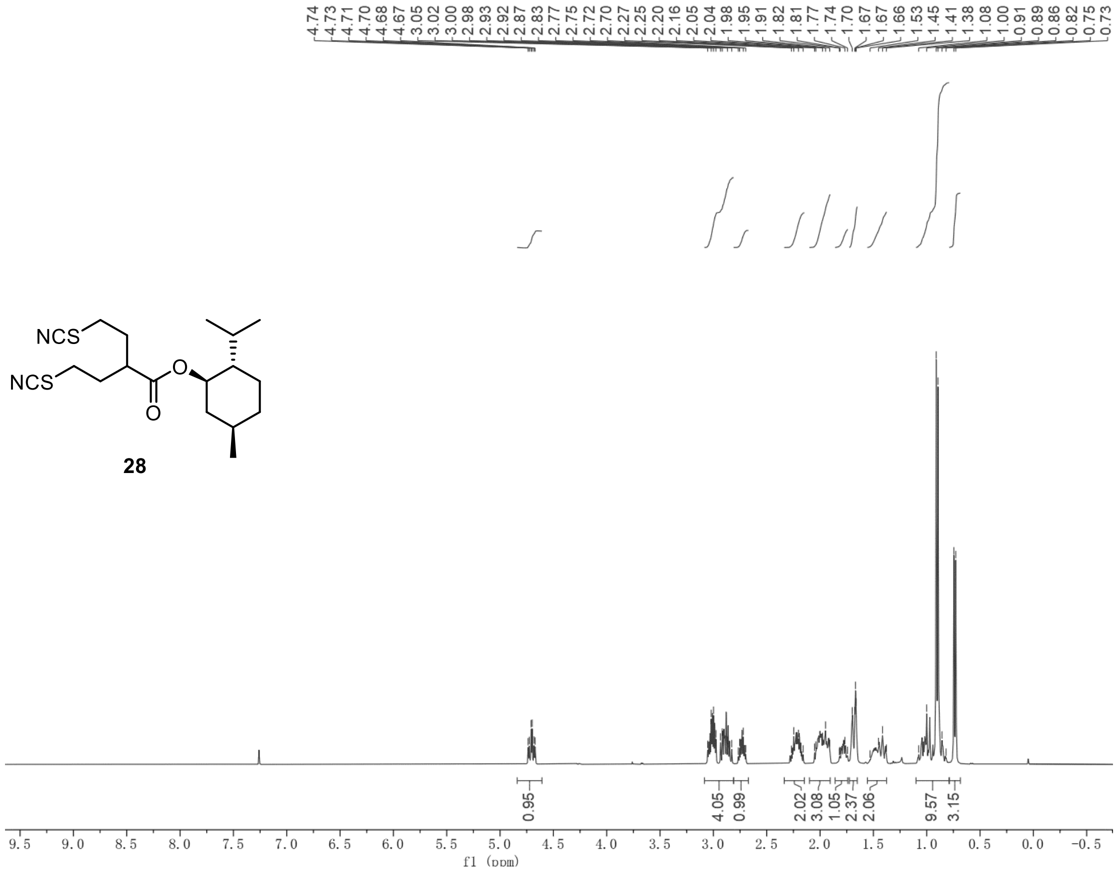

28

^{13}C NMR (100 MHz, $CDCl_3$) of **28**

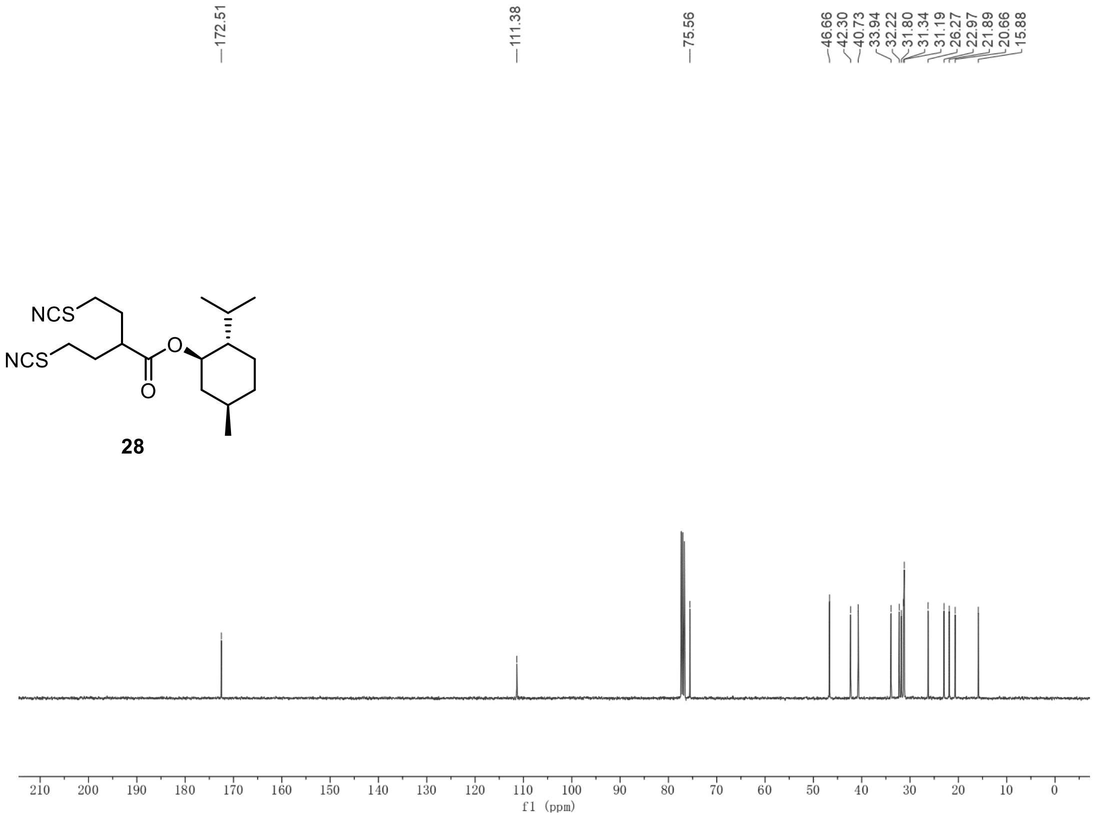

28

^{1}H NMR (400 MHz, $CDCl_3$) of **29**

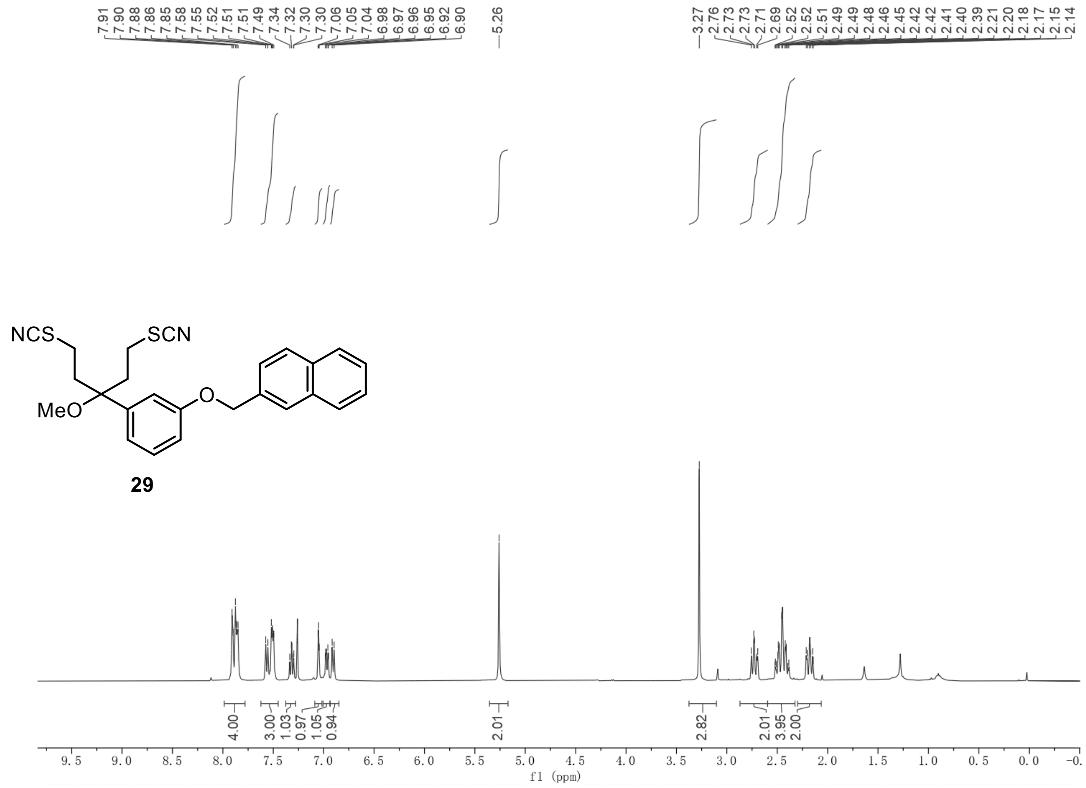

^{13}C NMR (100 MHz, $CDCl_3$) of **29**

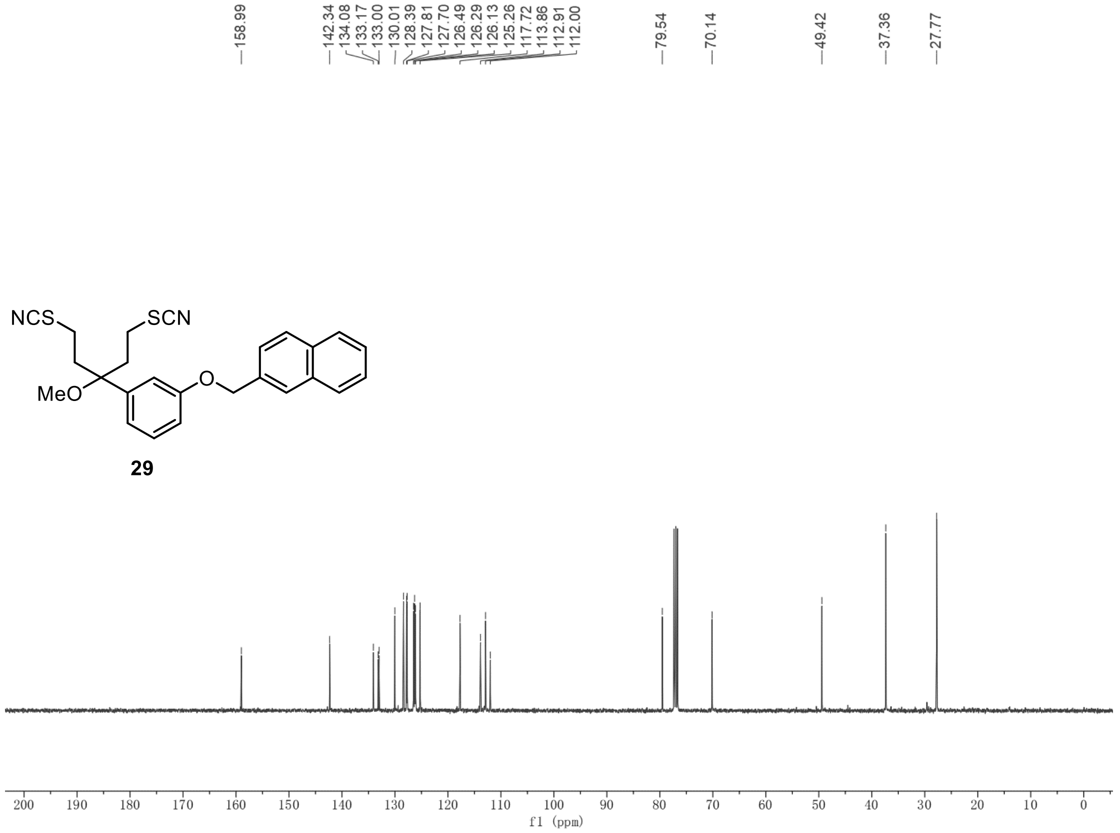

^{1}H NMR (400 MHz, CDCl$_3$) of **30**

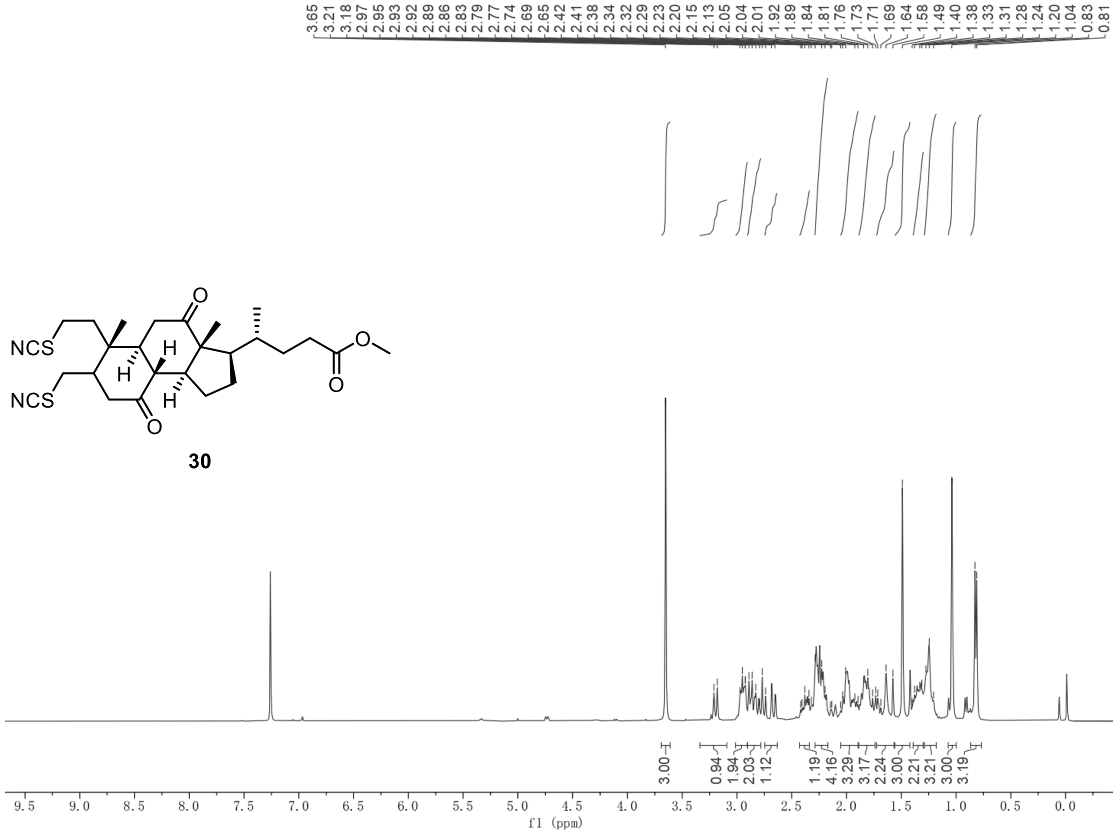

30

^{13}C NMR (100 MHz, CDCl$_3$) of **30**

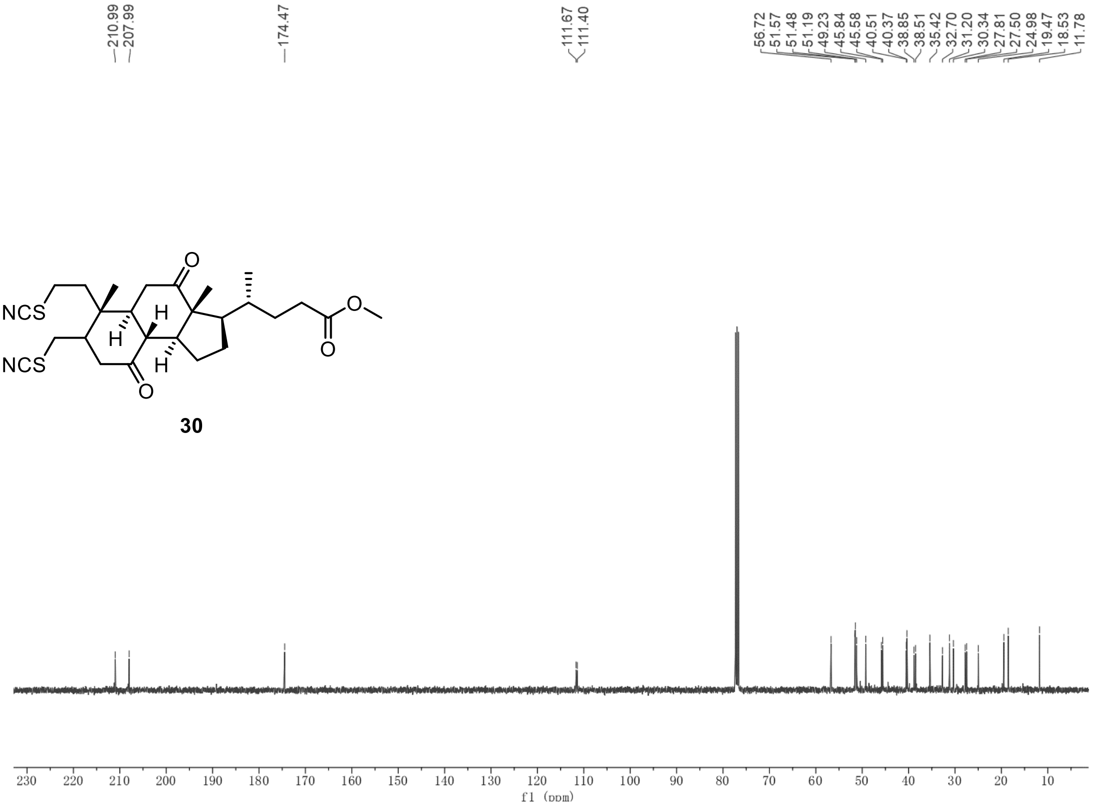

30

^{1}H NMR (400 MHz, $CDCl_3$) of **31**

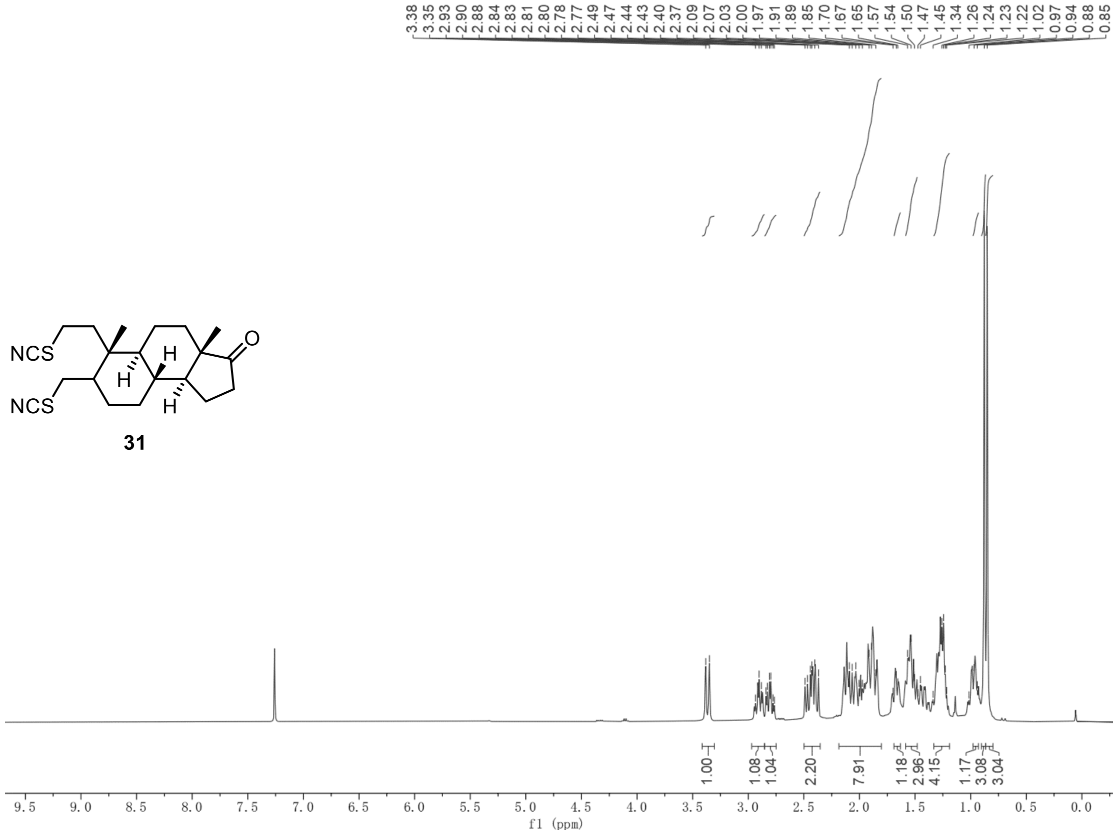

^{13}C NMR (100 MHz, $CDCl_3$) of **31**

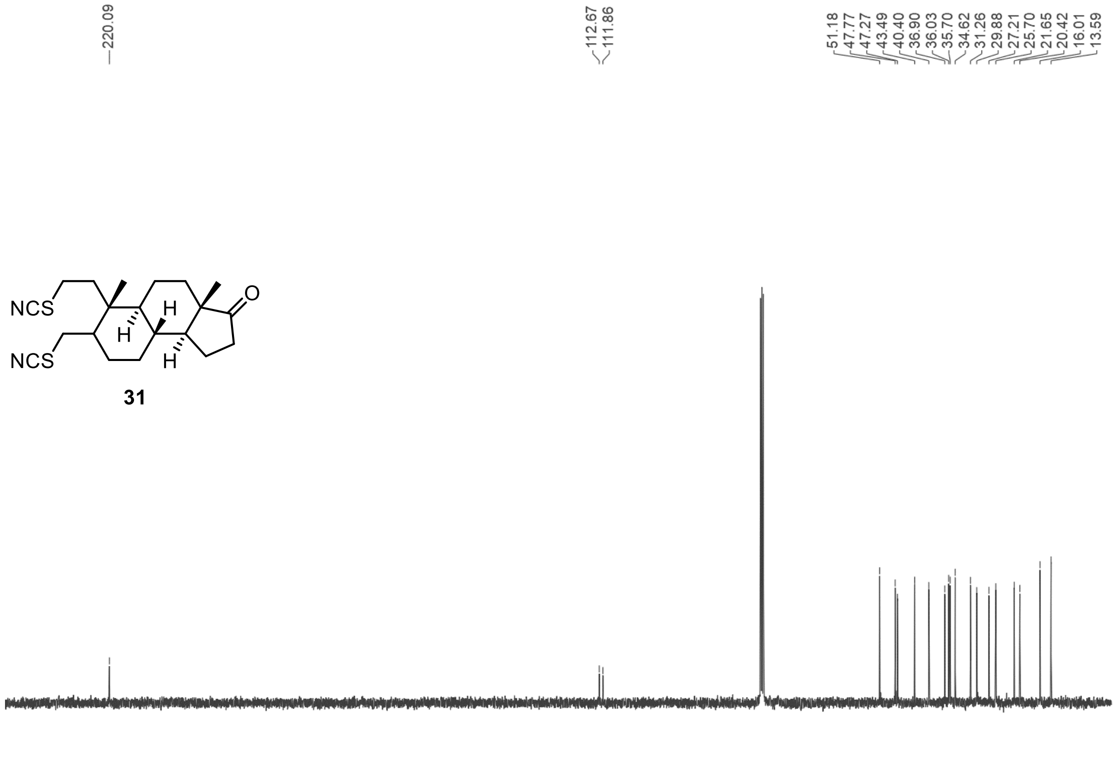

^{1}H NMR (400 MHz, CDCl$_3$) of **32**

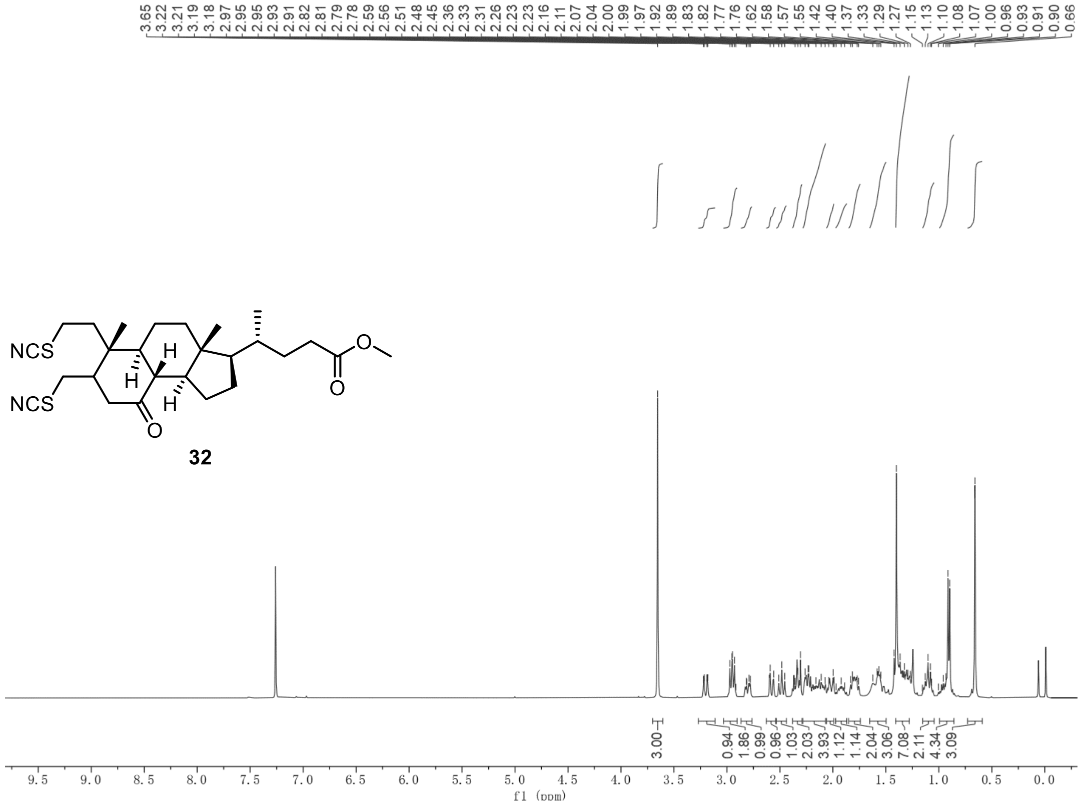

^{13}C NMR (100 MHz, CDCl$_3$) of **32**

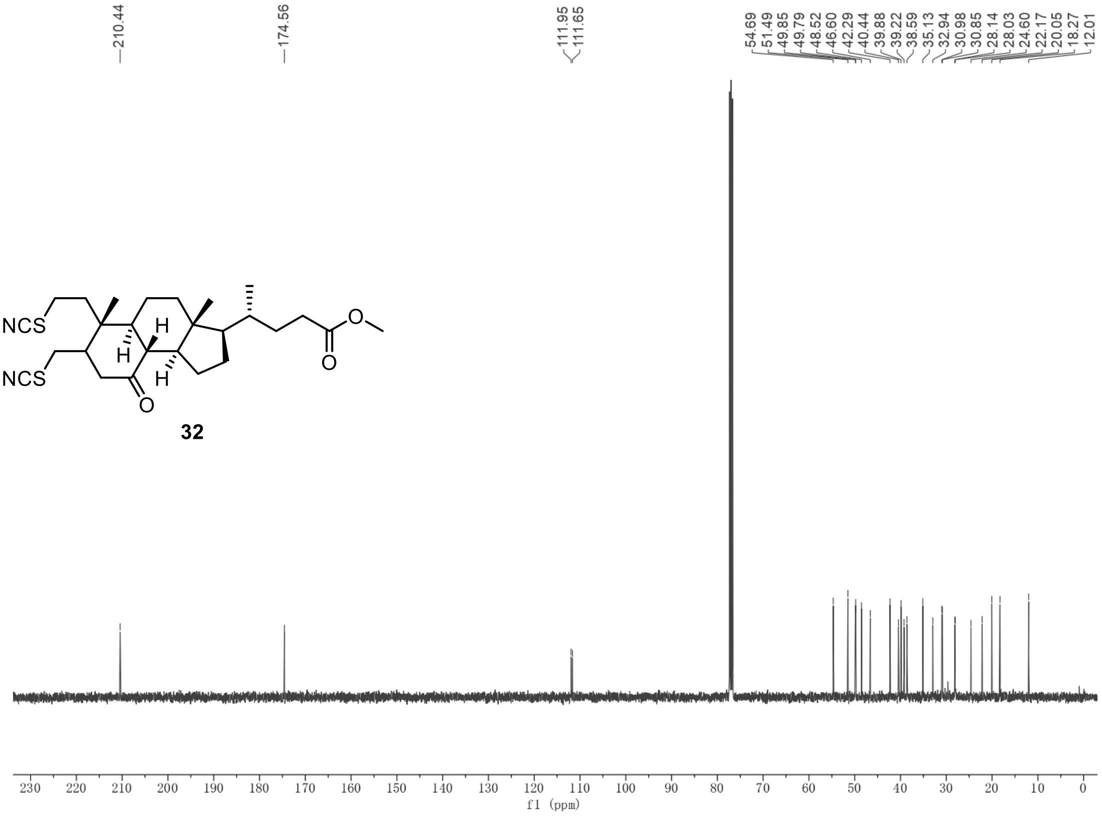

^{1}H NMR (400 MHz, $CDCl_3$) of **33**

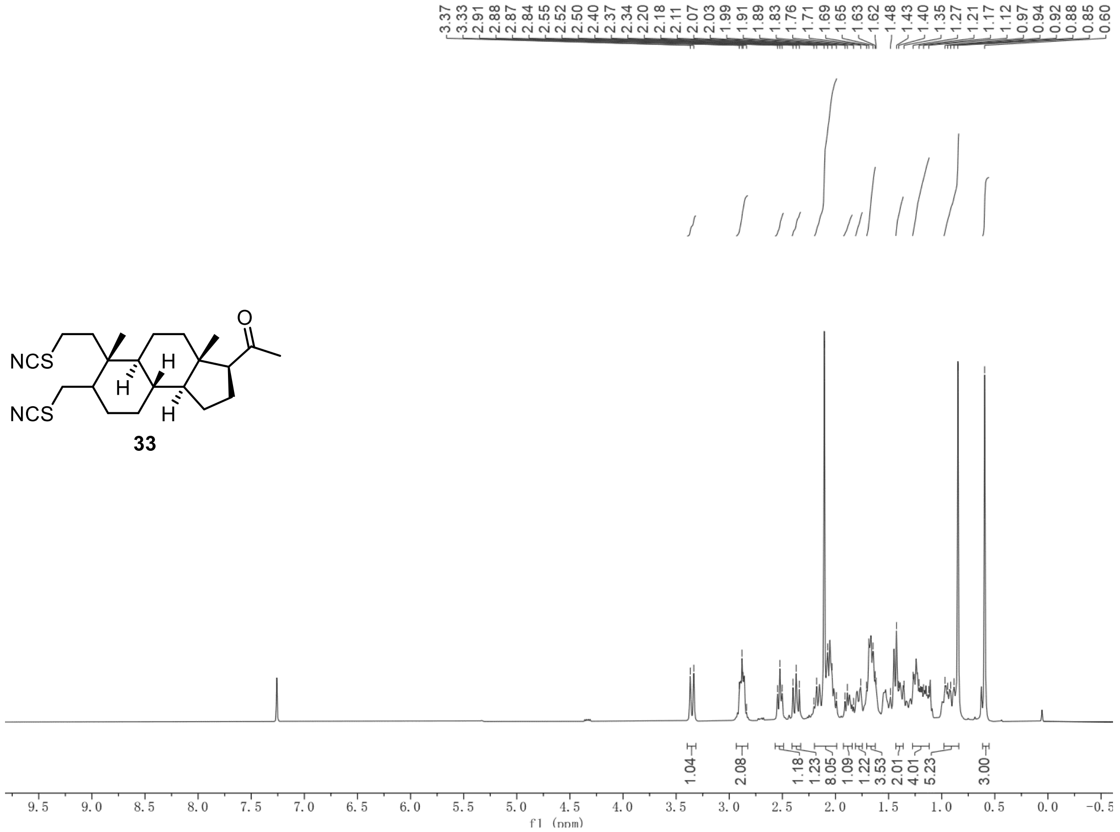

^{13}C NMR (100 MHz, $CDCl_3$) of **33**

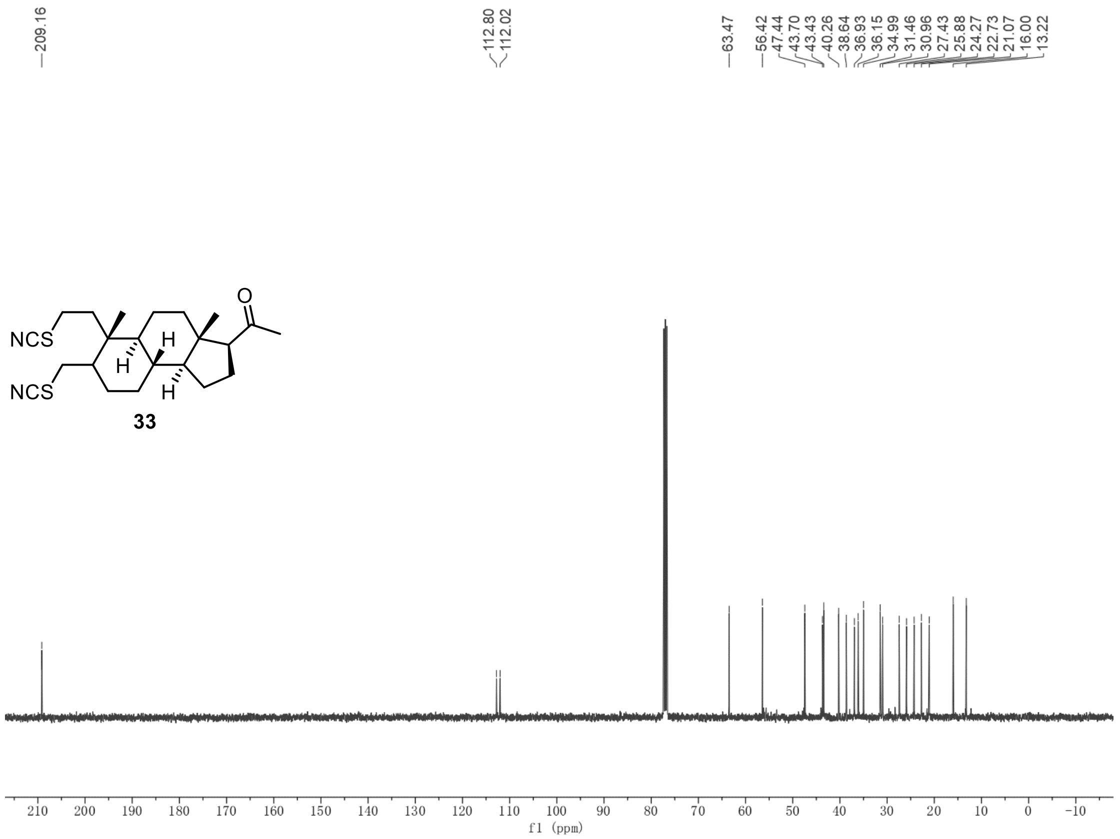

^{1}H NMR (400 MHz, $CDCl_3$) of **34**

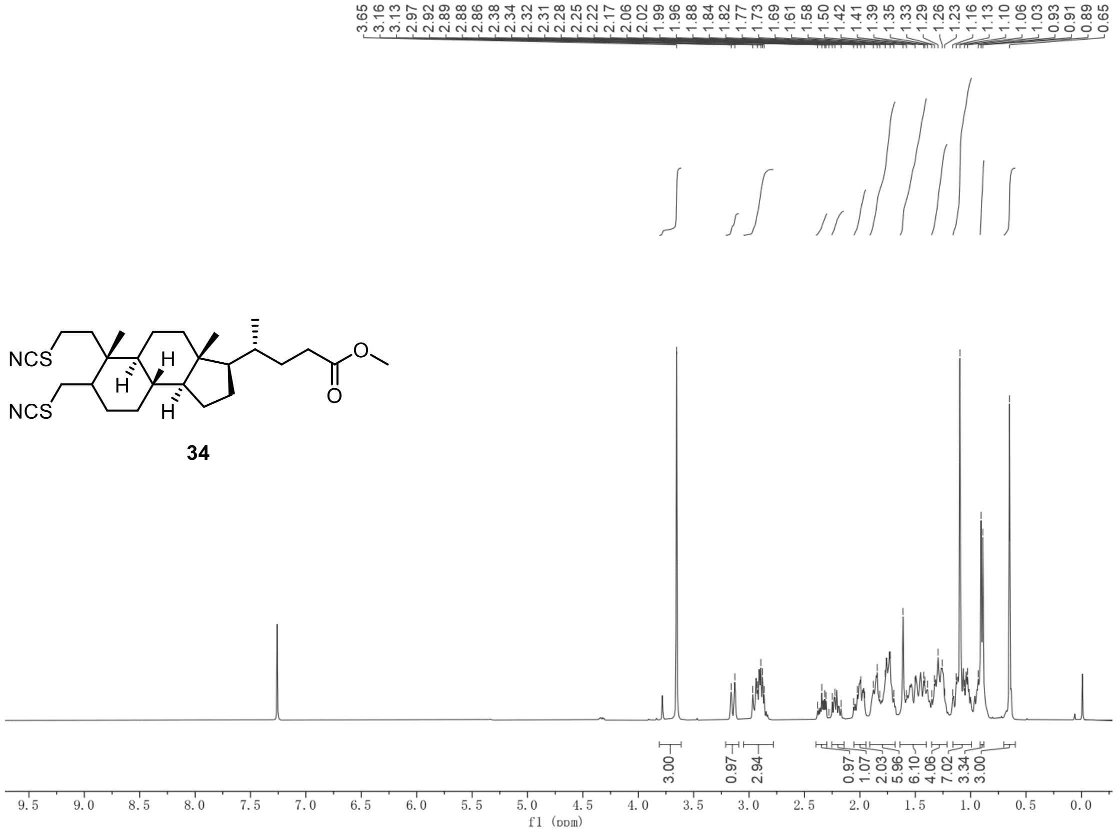

^{13}C NMR (100 MHz, $CDCl_3$) of **34**

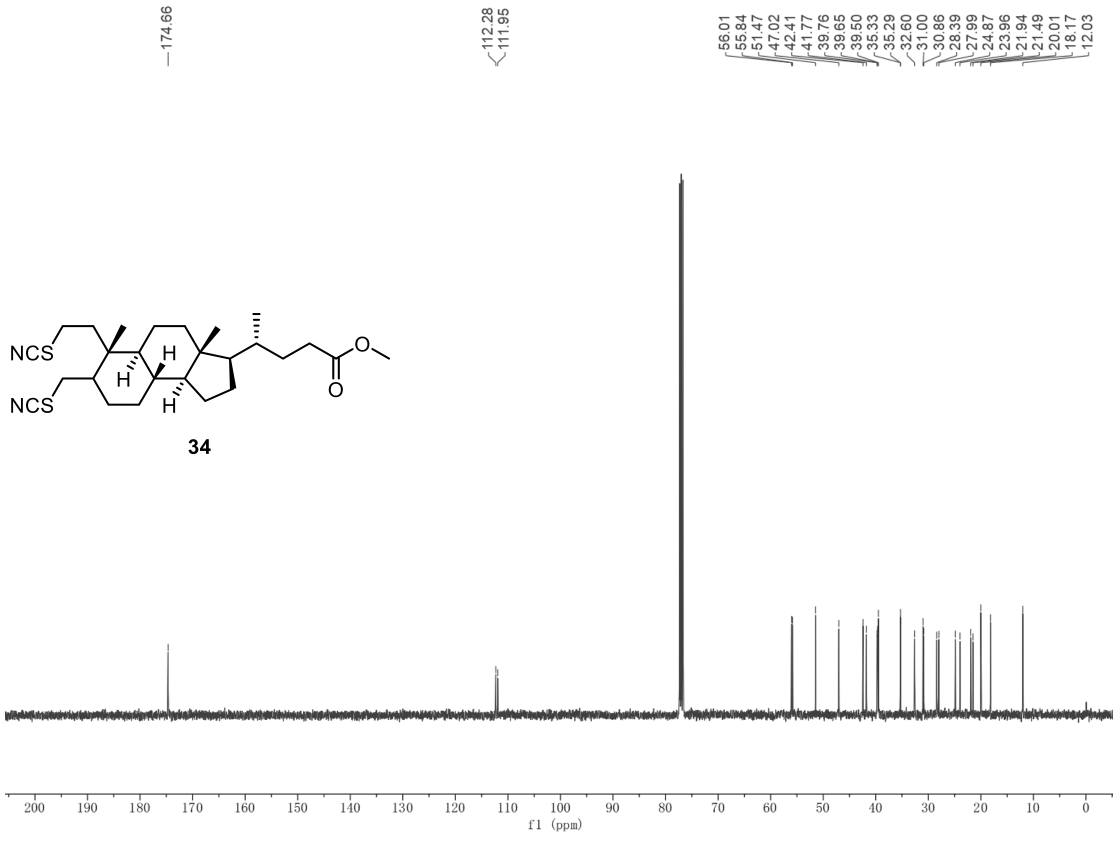

^{1}H NMR (400 MHz, $CDCl_3$) of **35**

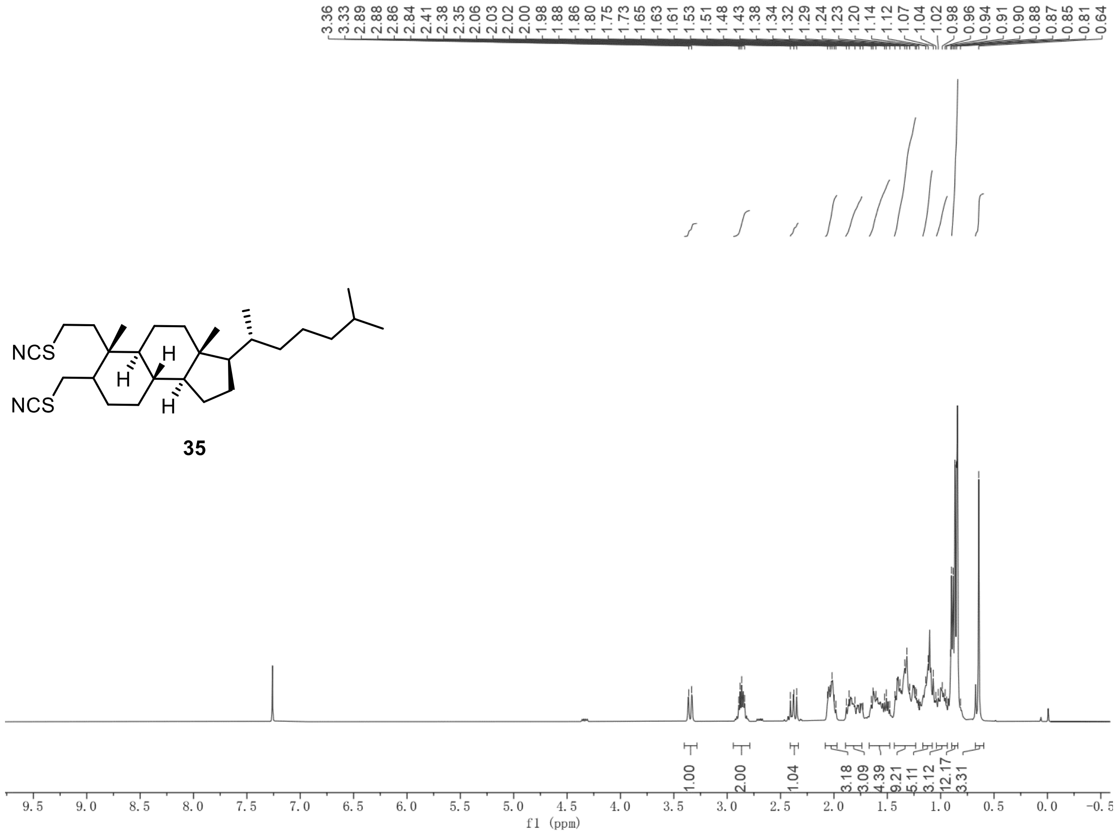

^{13}C NMR (100 MHz, $CDCl_3$) of **35**

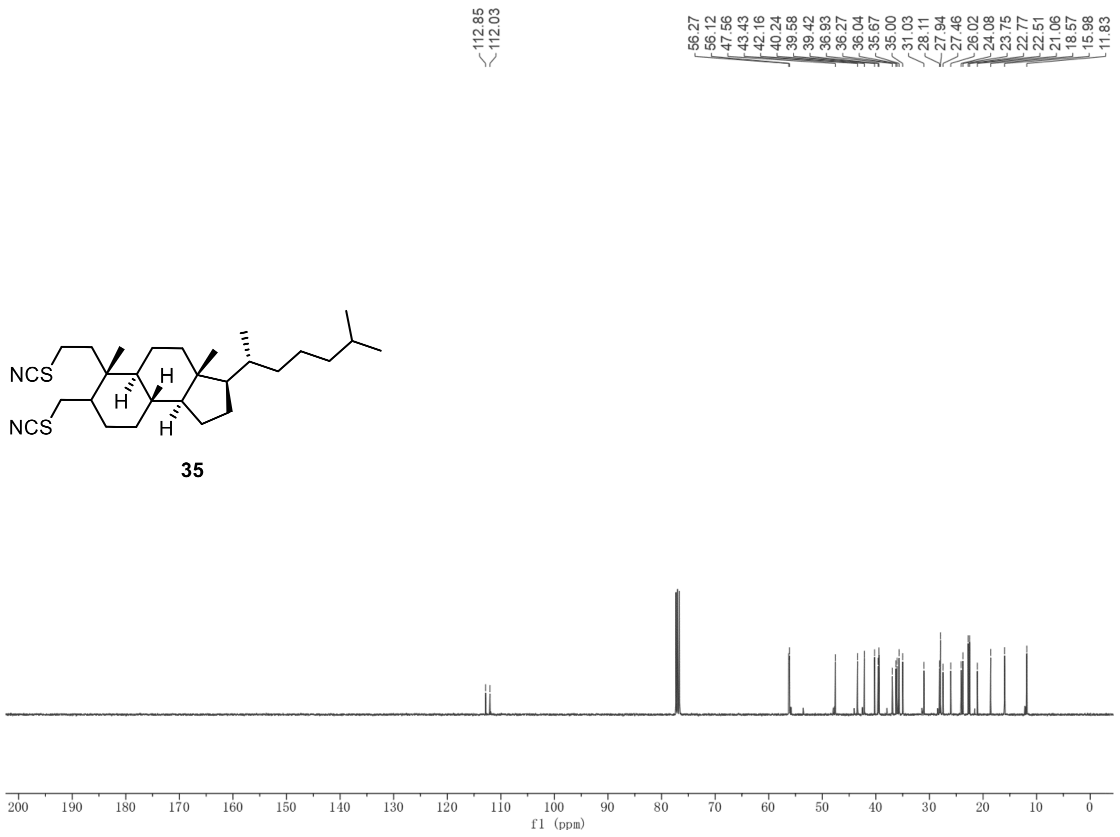

^{1}H NMR (400 MHz, $CDCl_3$) of **36**

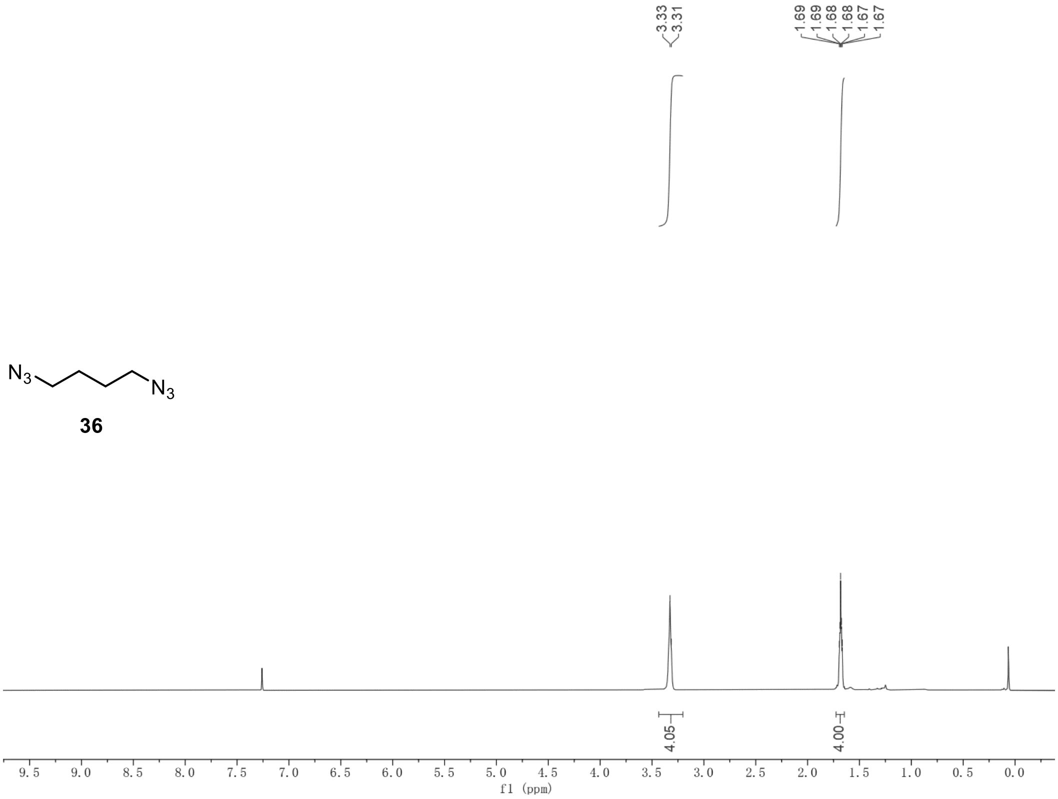

36

^{13}C NMR (100 MHz, $CDCl_3$) of **36**

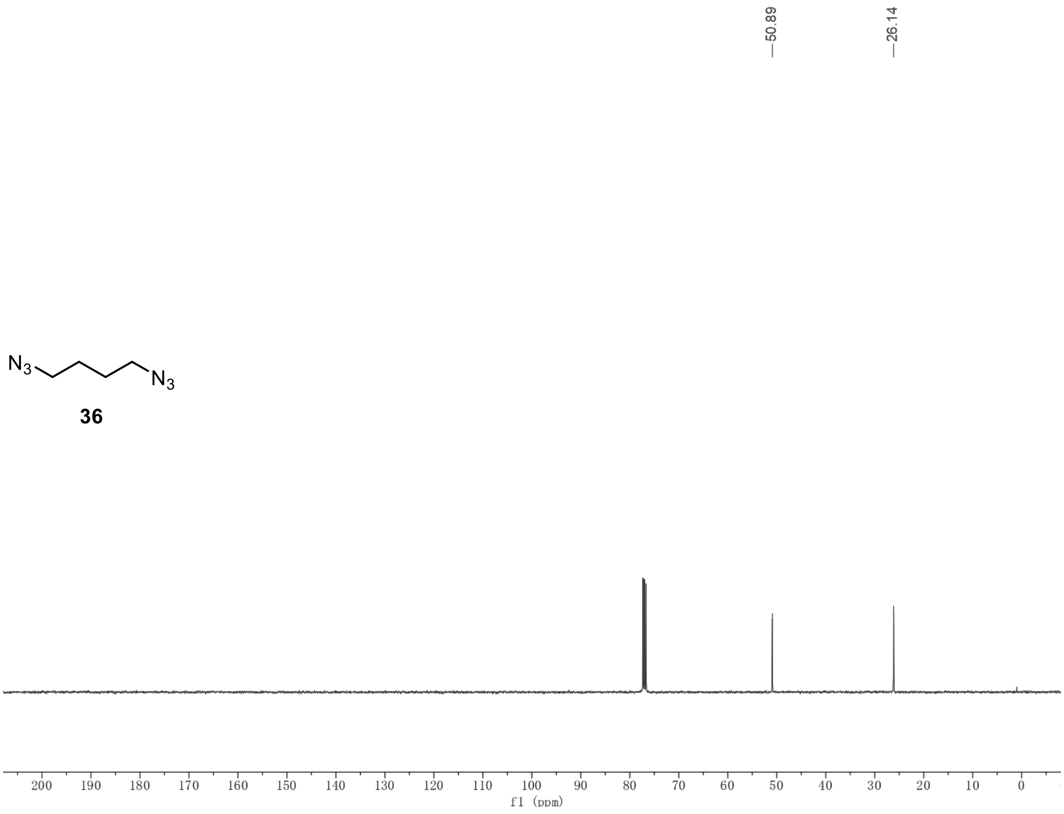

36

^{1}H NMR (400 MHz, $CDCl_3$) of **37**

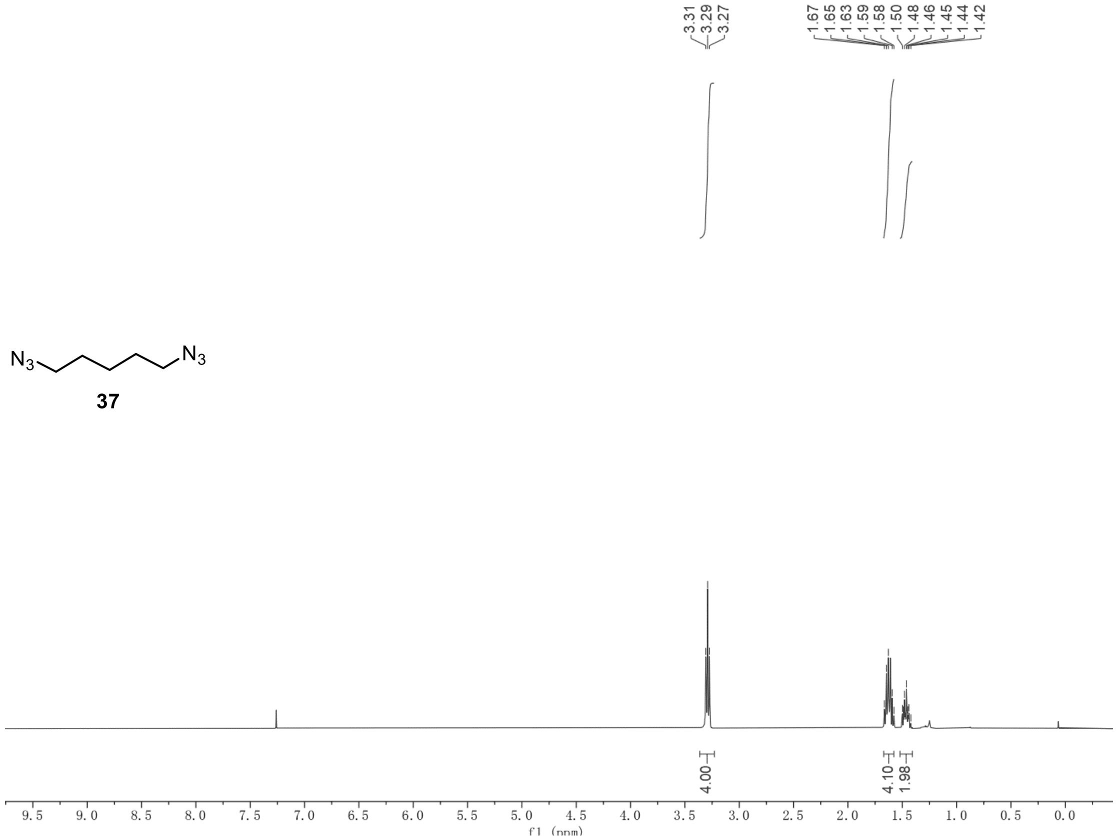

37

^{13}C NMR (100 MHz, $CDCl_3$) of **37**

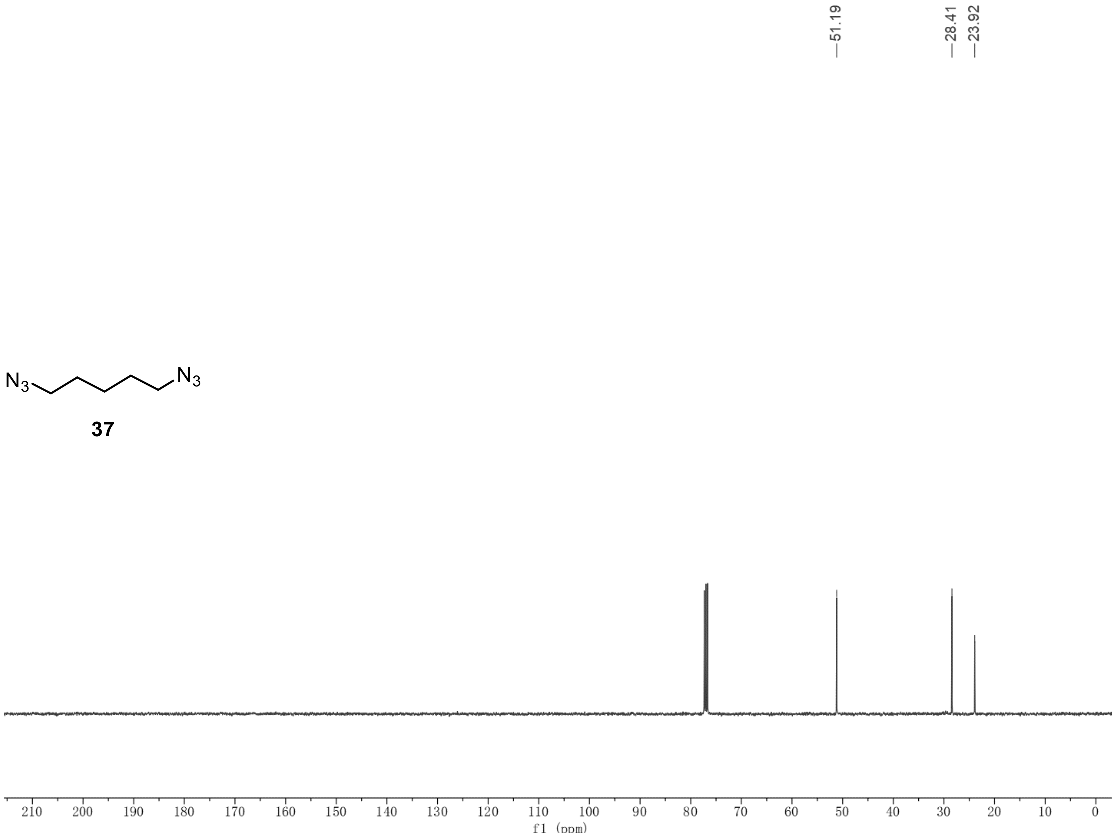

37

^{1}**H NMR** (400 MHz, $CDCl_3$) of **38**

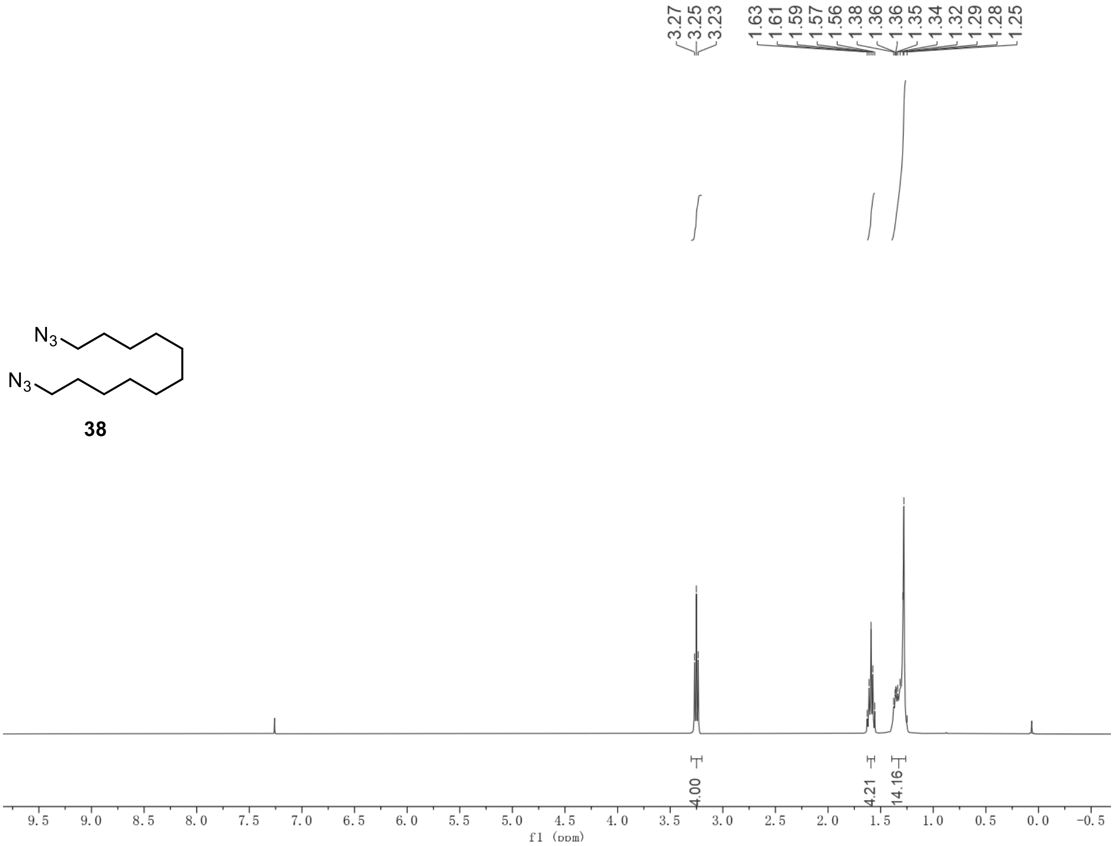

^{13}**C NMR** (100 MHz, $CDCl_3$) of **38**

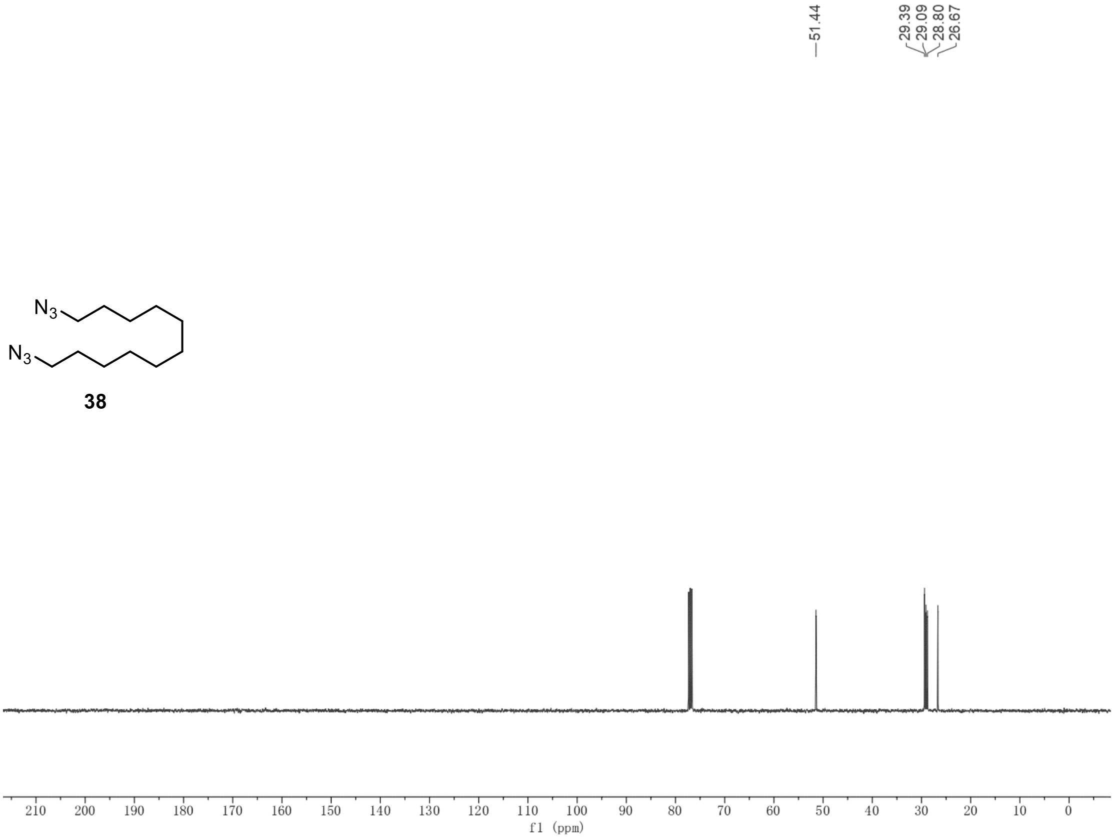

^{1}H NMR (400 MHz, $CDCl_3$) of **39**

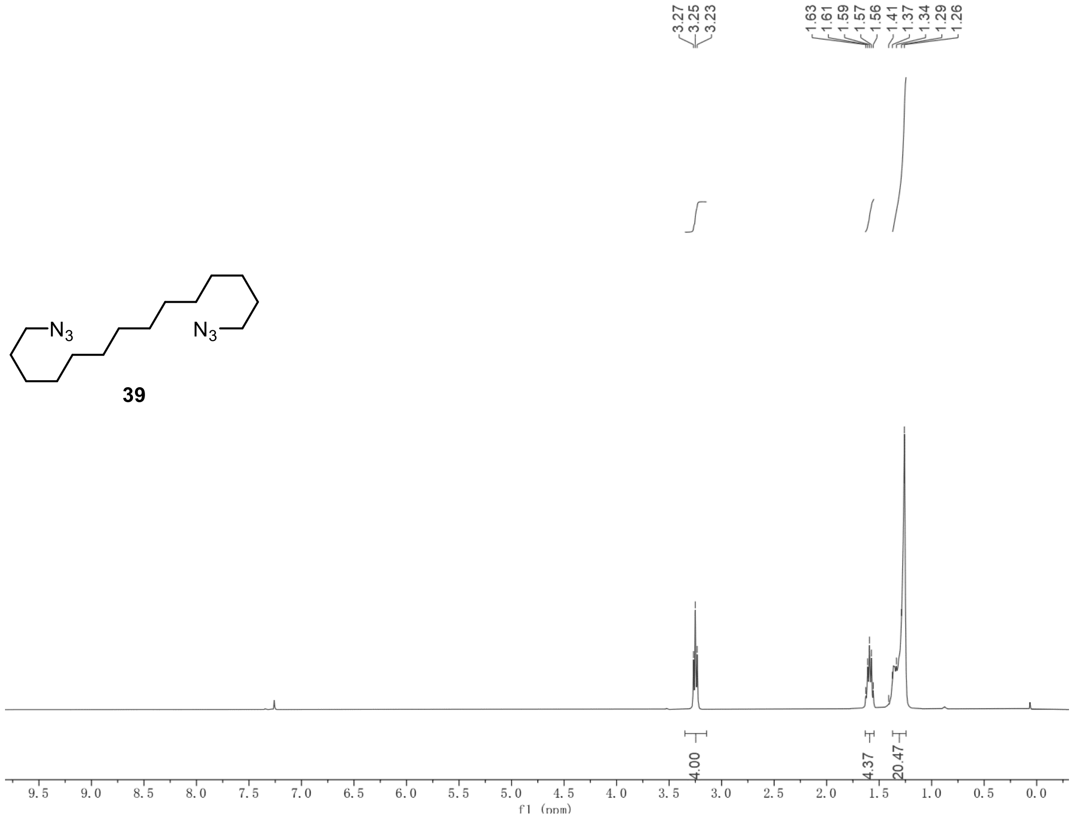

^{13}C NMR (100 MHz, $CDCl_3$) of **39**

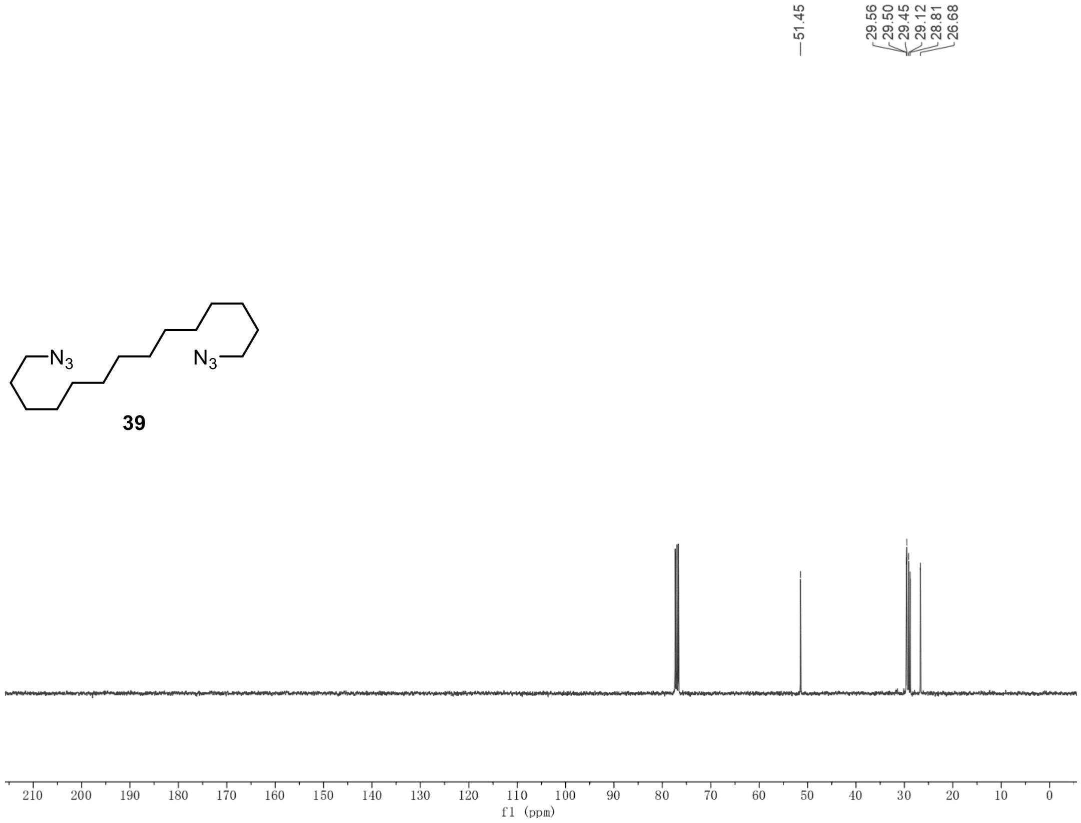

^{1}H NMR (400 MHz, $CDCl_3$) of **40**

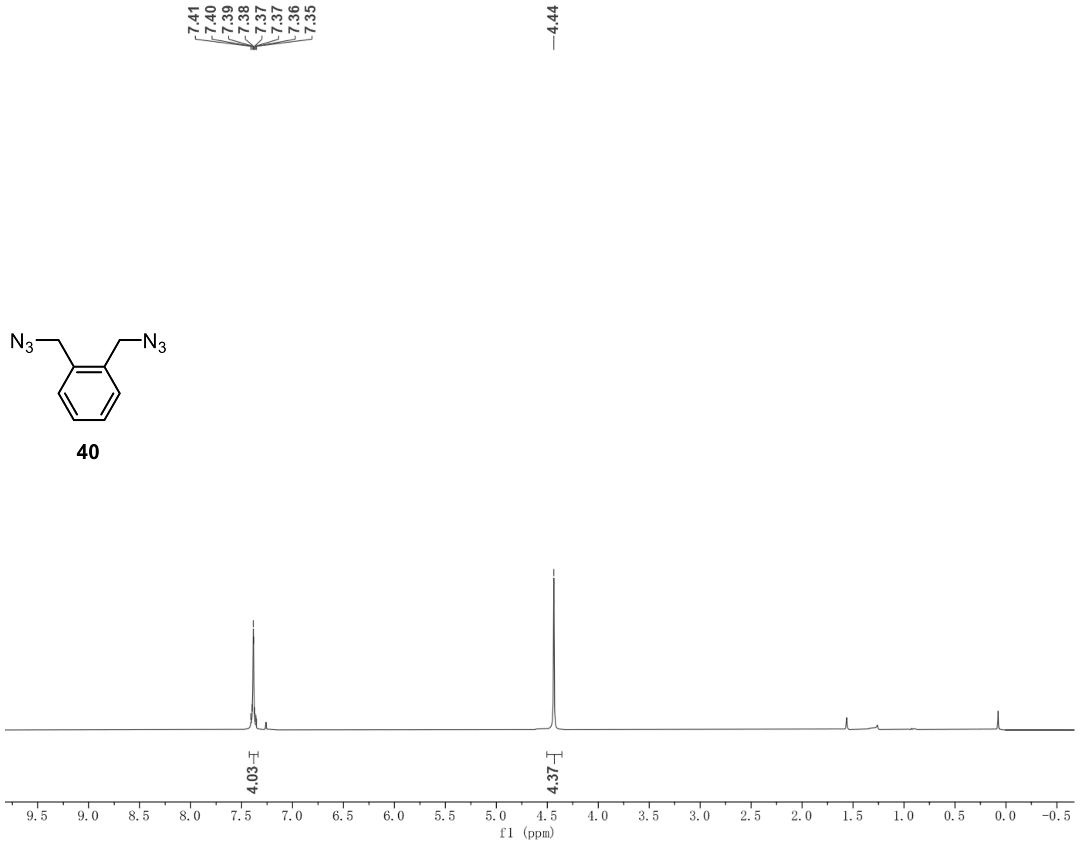

^{13}C NMR (100 MHz, $CDCl_3$) of **40**

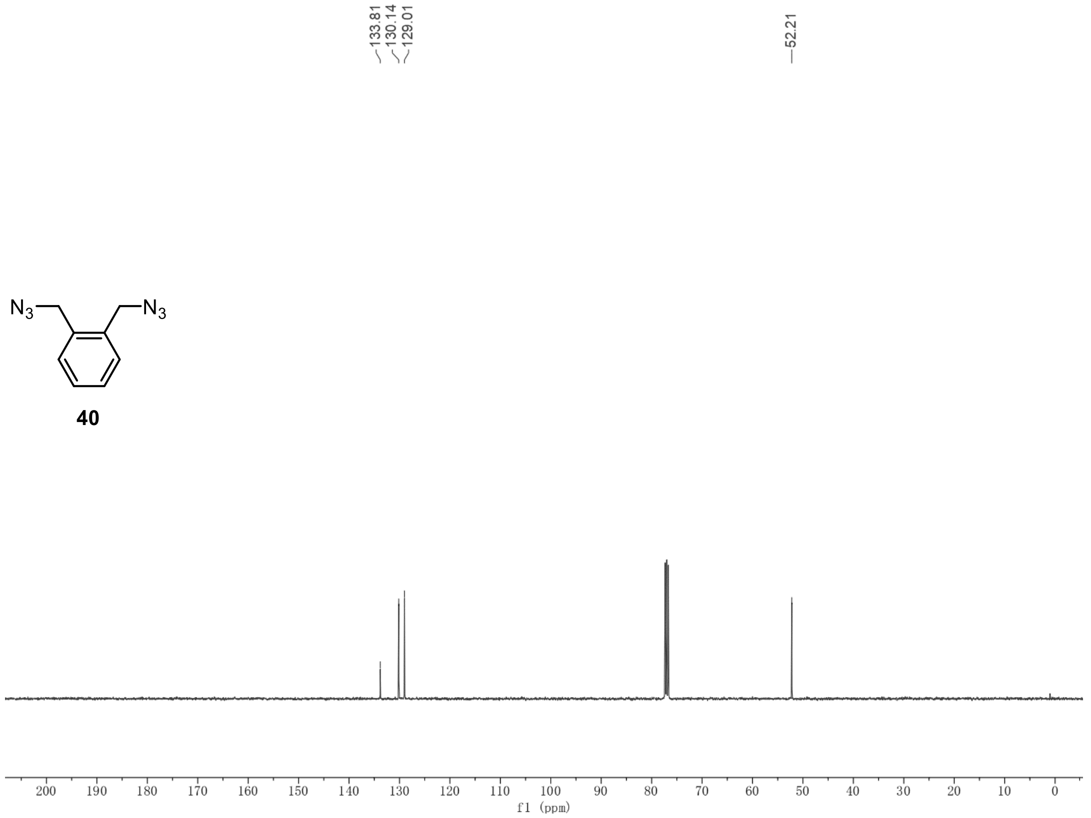

^{1}H NMR (400 MHz, $CDCl_3$) of **41**

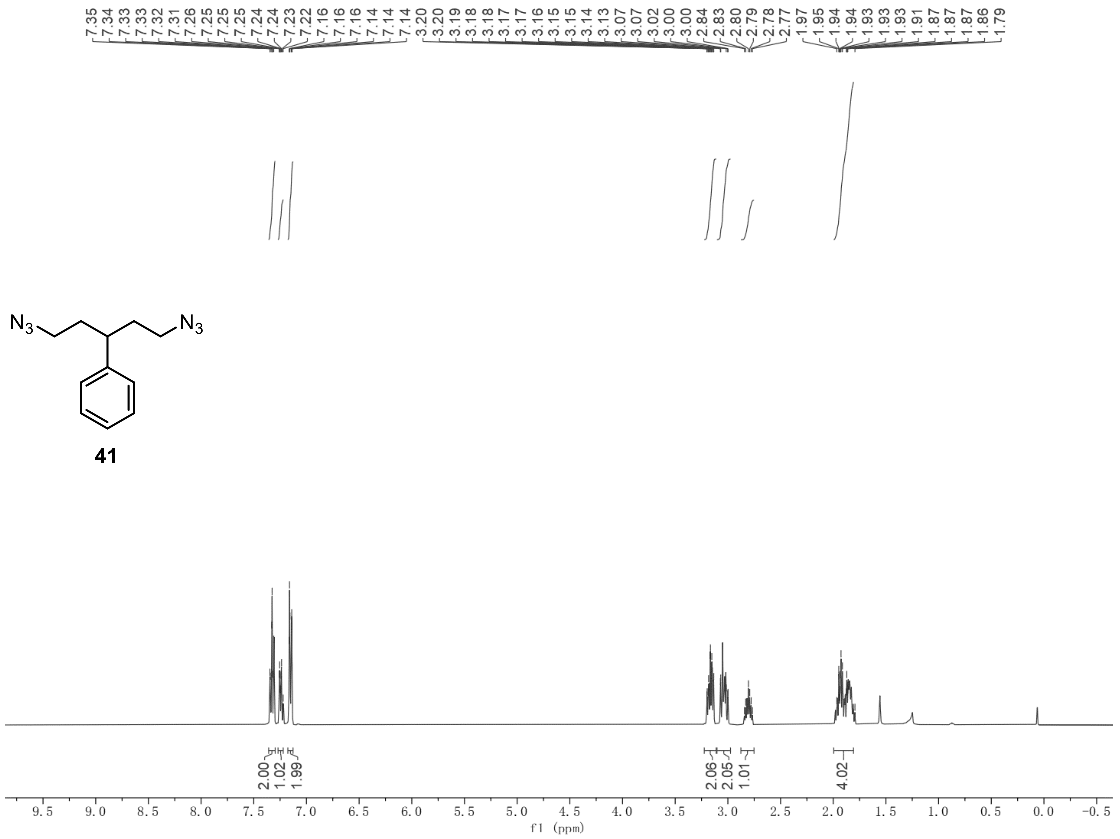

^{13}C NMR (100 MHz, $CDCl_3$) of **41**

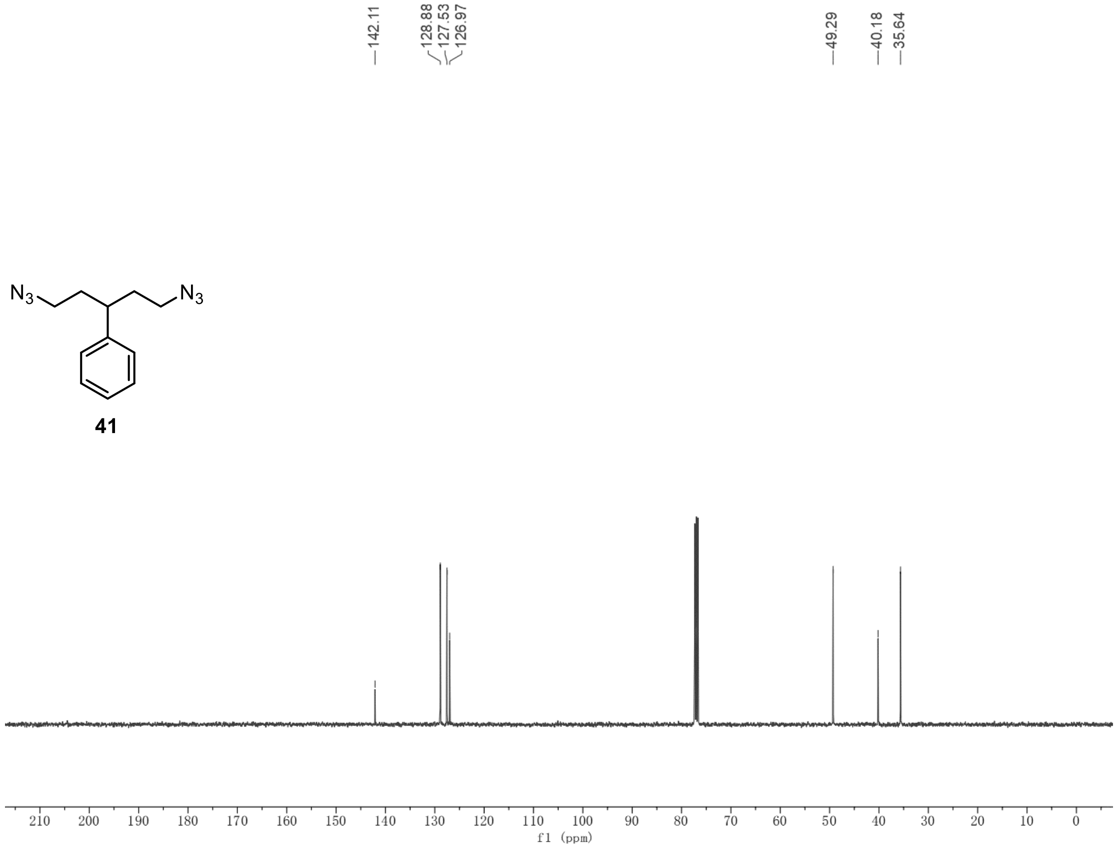

^{1}H NMR (400 MHz, $CDCl_3$) of **42**

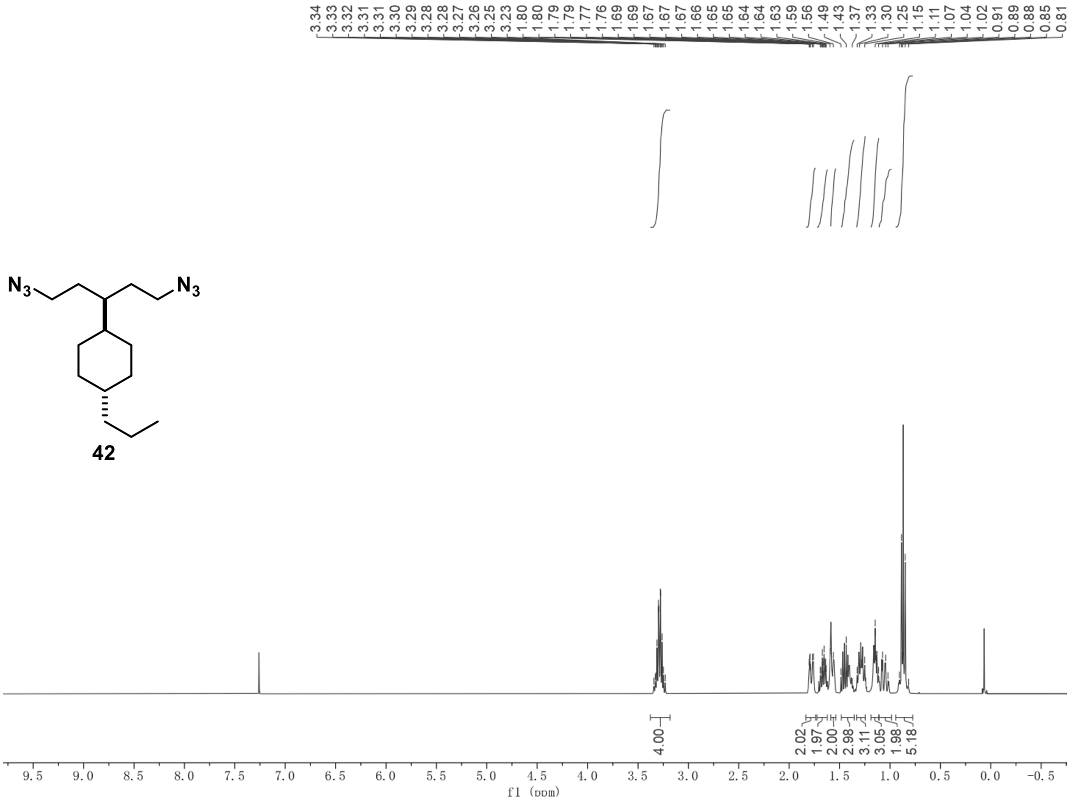

^{13}C NMR (100 MHz, $CDCl_3$) of **42**

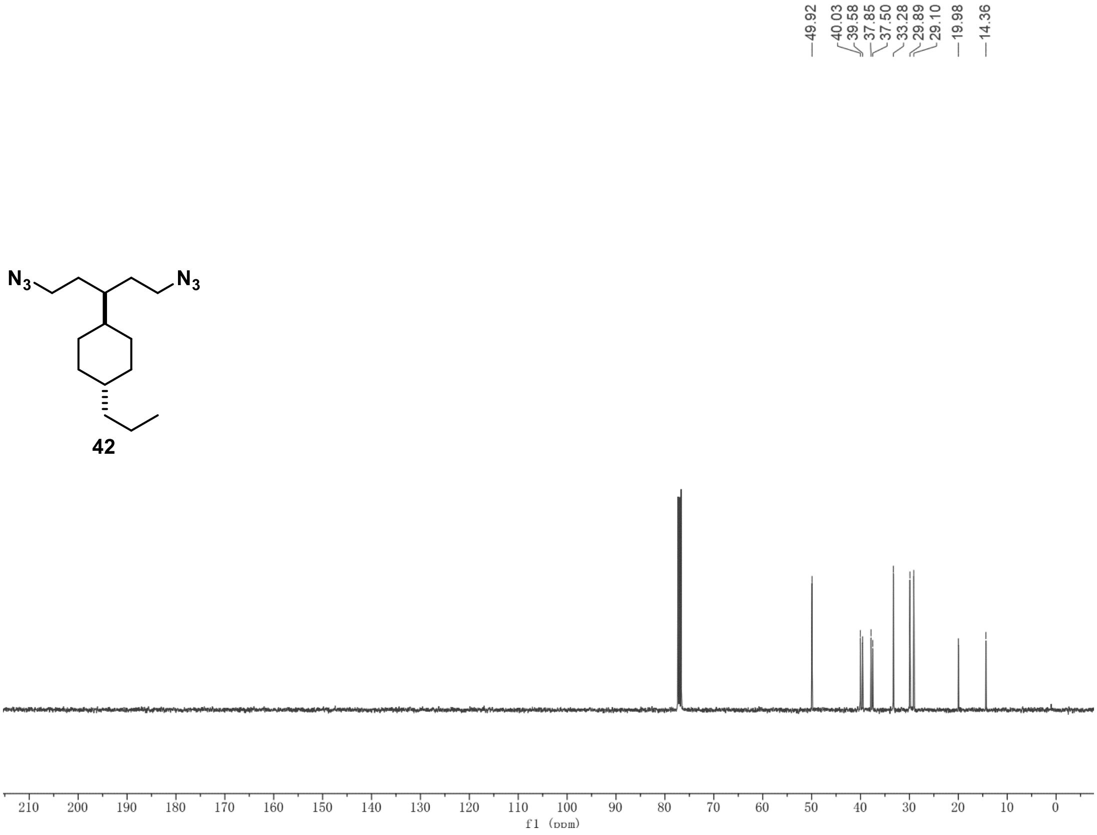

^{1}H NMR (400 MHz, $CDCl_3$) of **43**

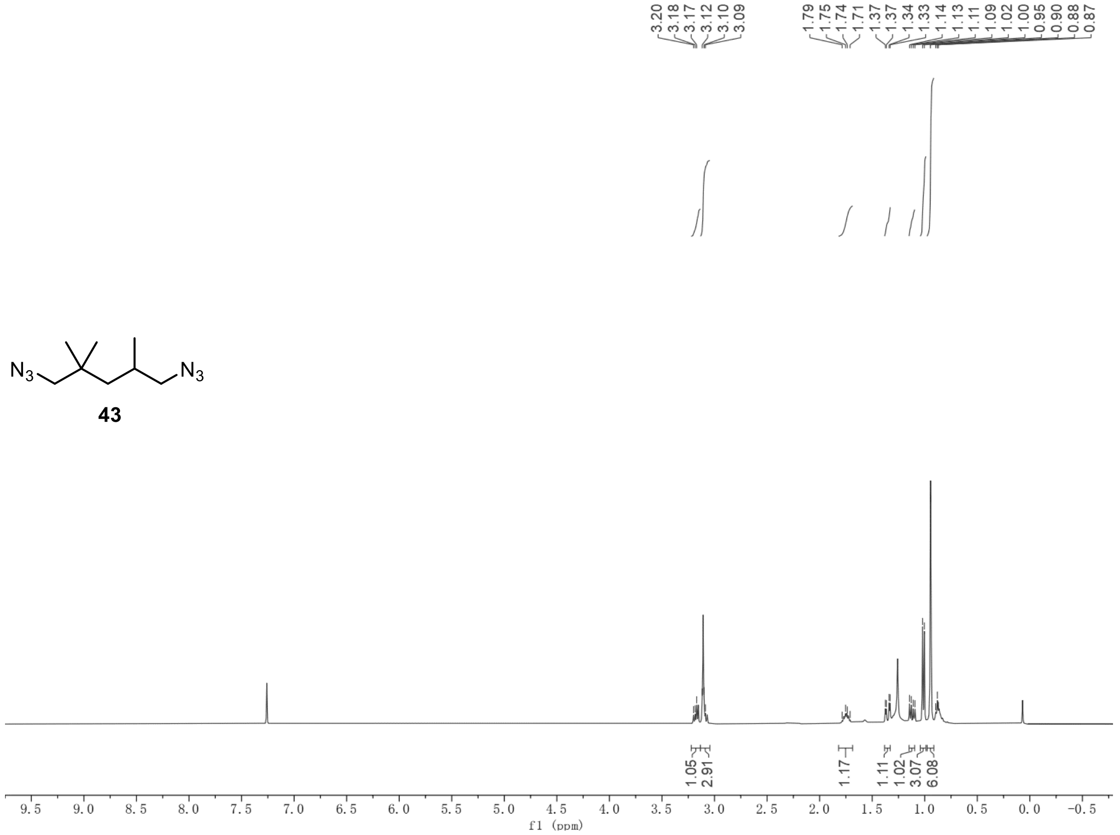

43

^{13}C NMR (100 MHz, $CDCl_3$) of **43**

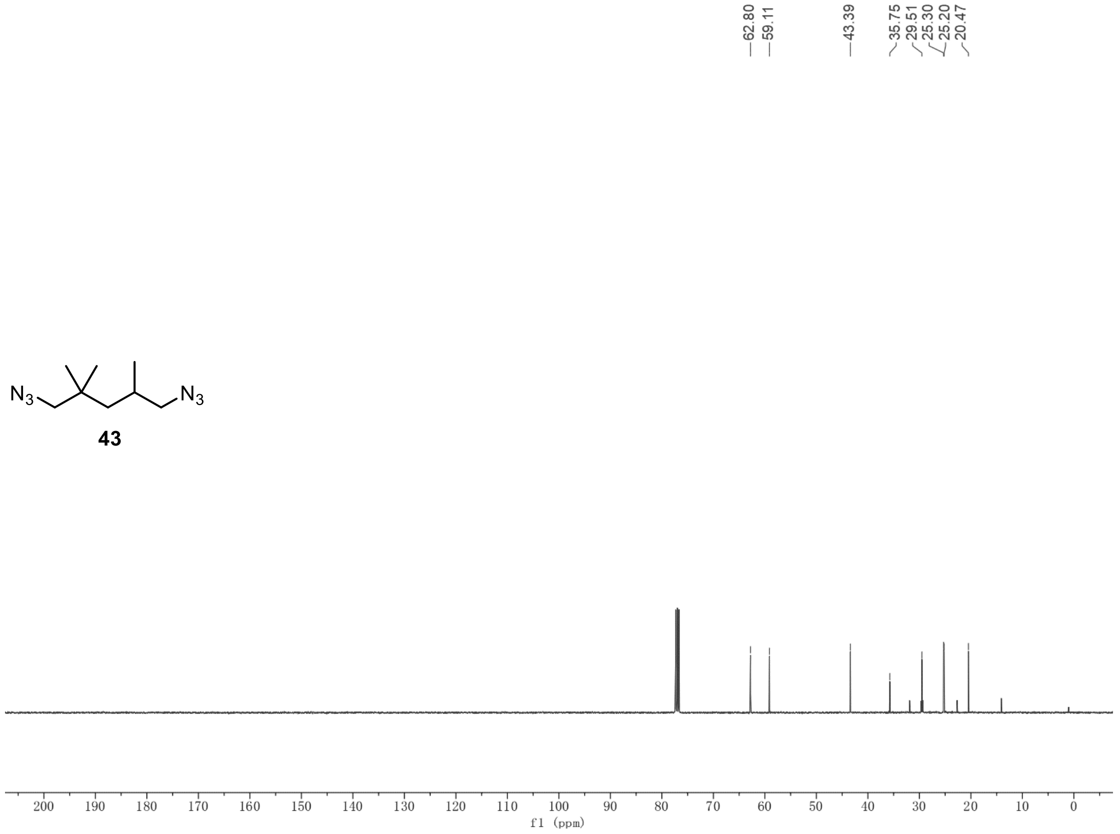

43

^{1}H NMR (400 MHz, $CDCl_3$) of **44**

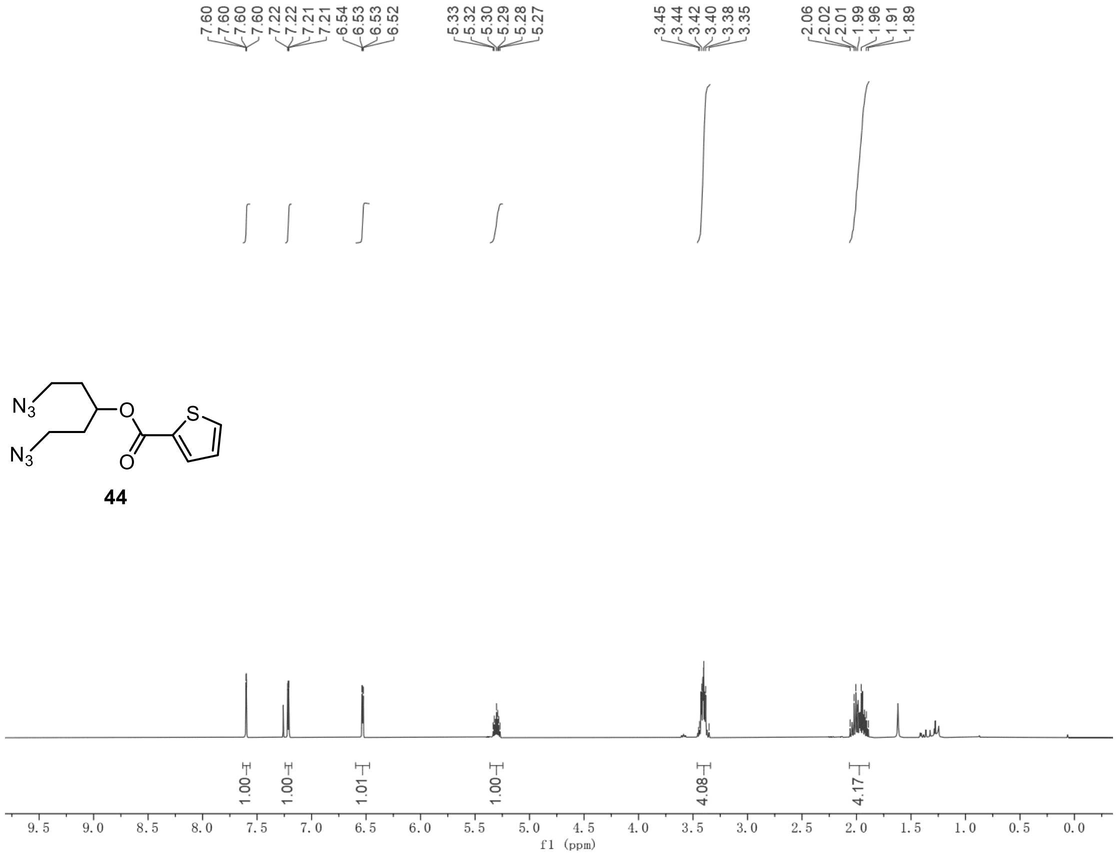

^{13}C NMR (100 MHz, $CDCl_3$) of **44**

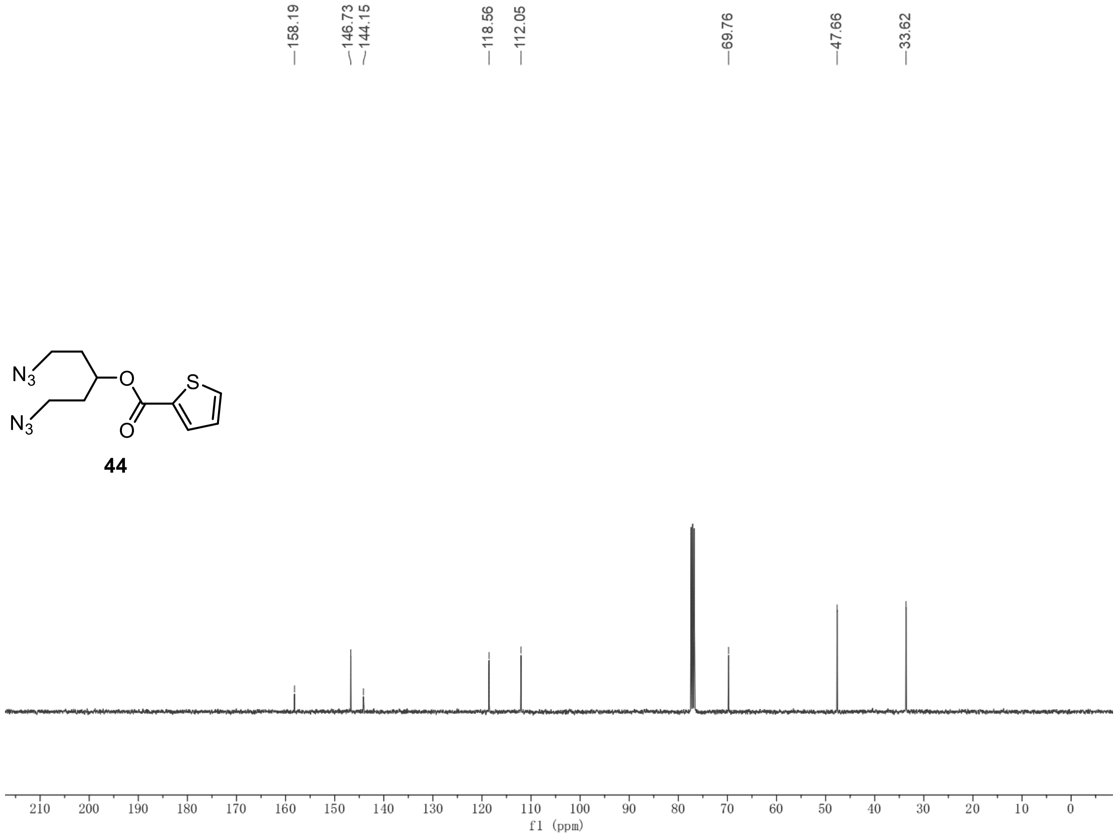

^{1}H NMR (400 MHz, $CDCl_3$) of **45**

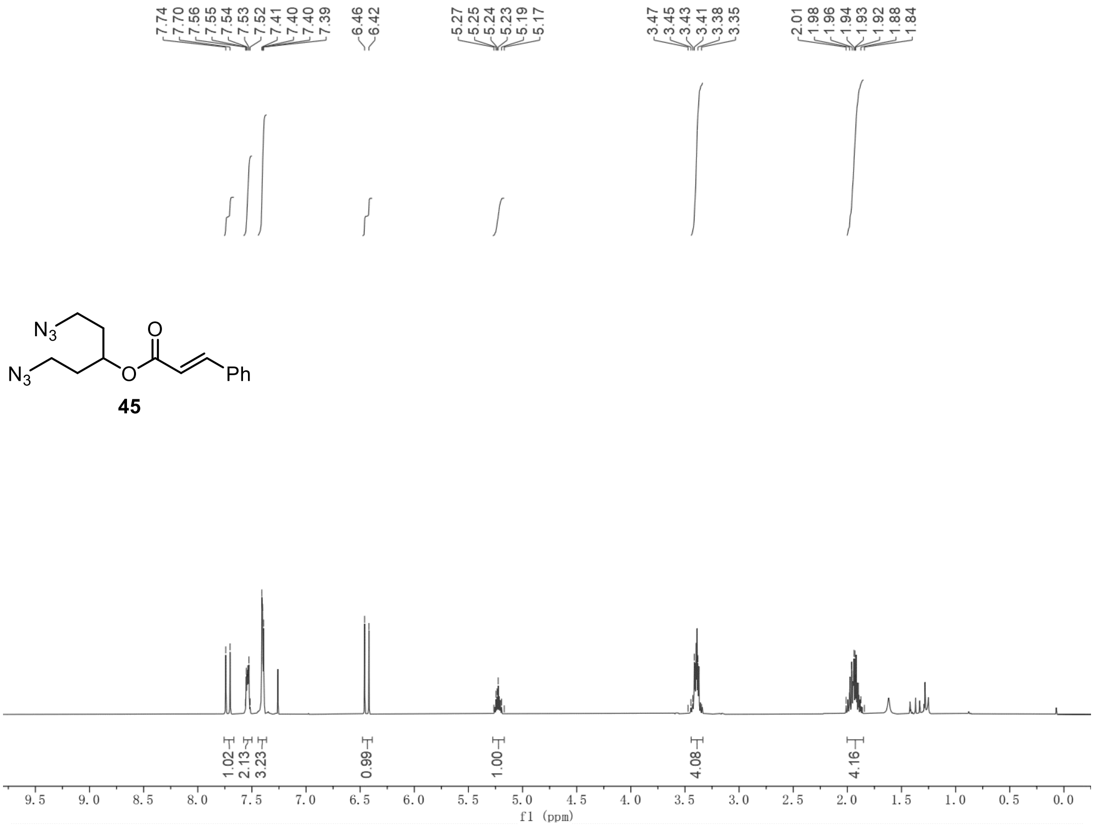

^{13}C NMR (100 MHz, $CDCl_3$) of **45**

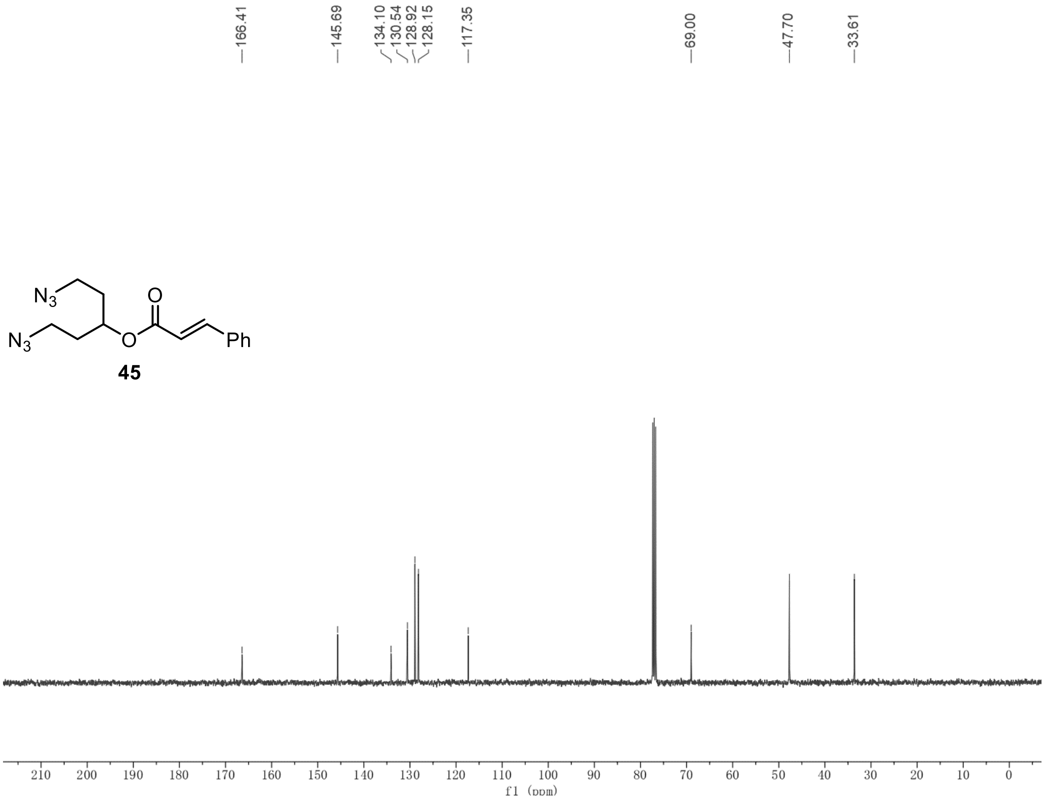

^{1}H NMR (400 MHz, $CDCl_3$) of **46**

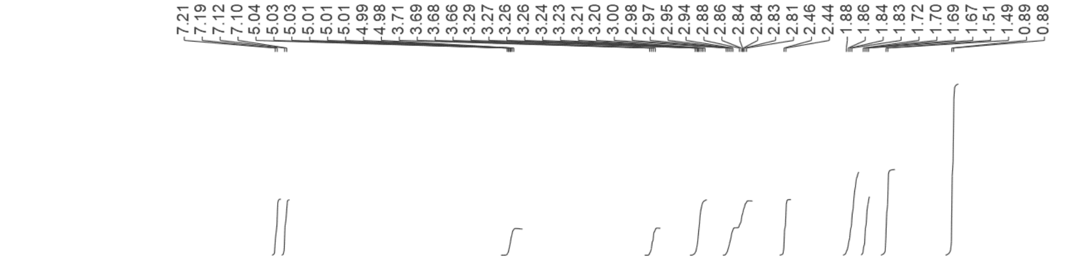

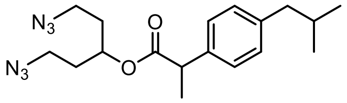

46

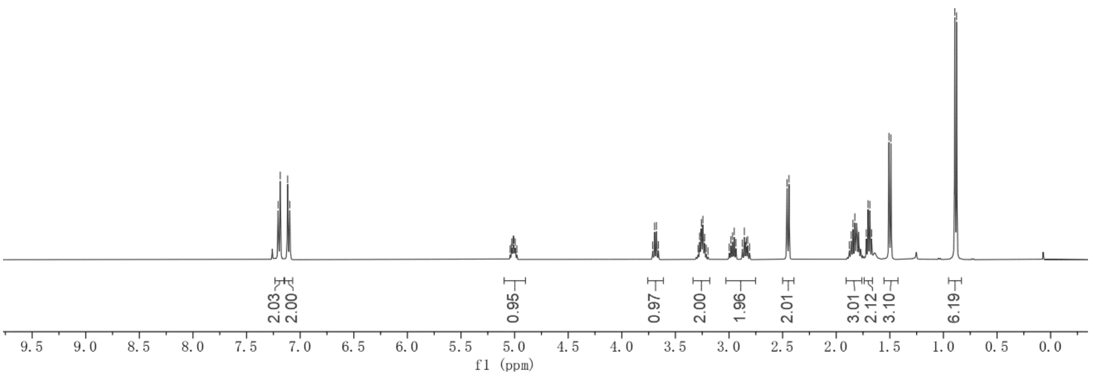

^{13}C NMR (100 MHz, $CDCl_3$) of **46**

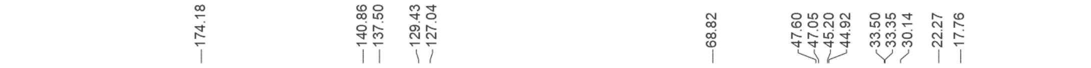

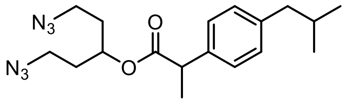

46

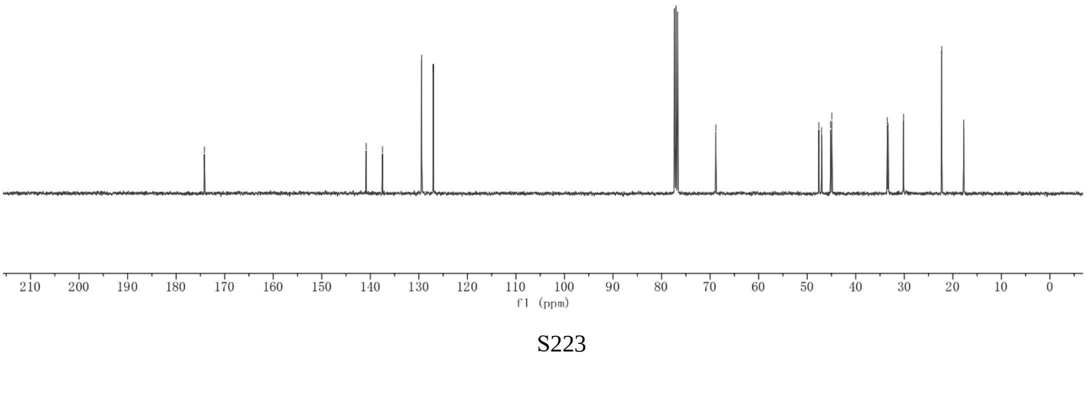

^{1}H NMR (400 MHz, $CDCl_3$) of **47**

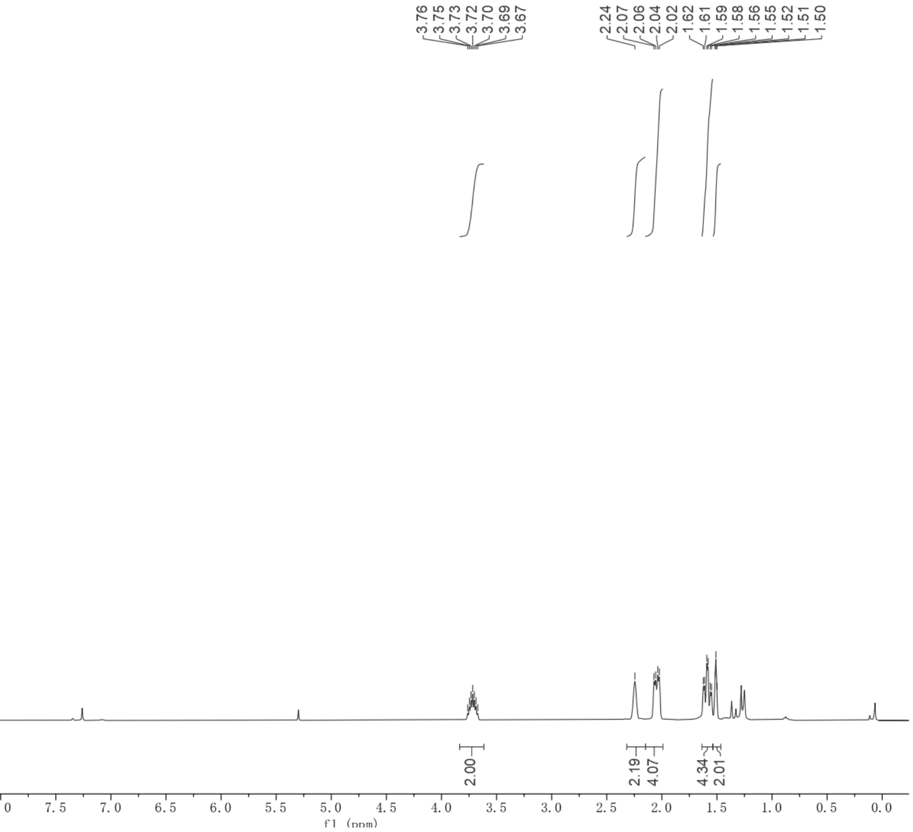

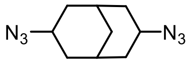

47

^{13}C NMR (100 MHz, $CDCl_3$) of **47**

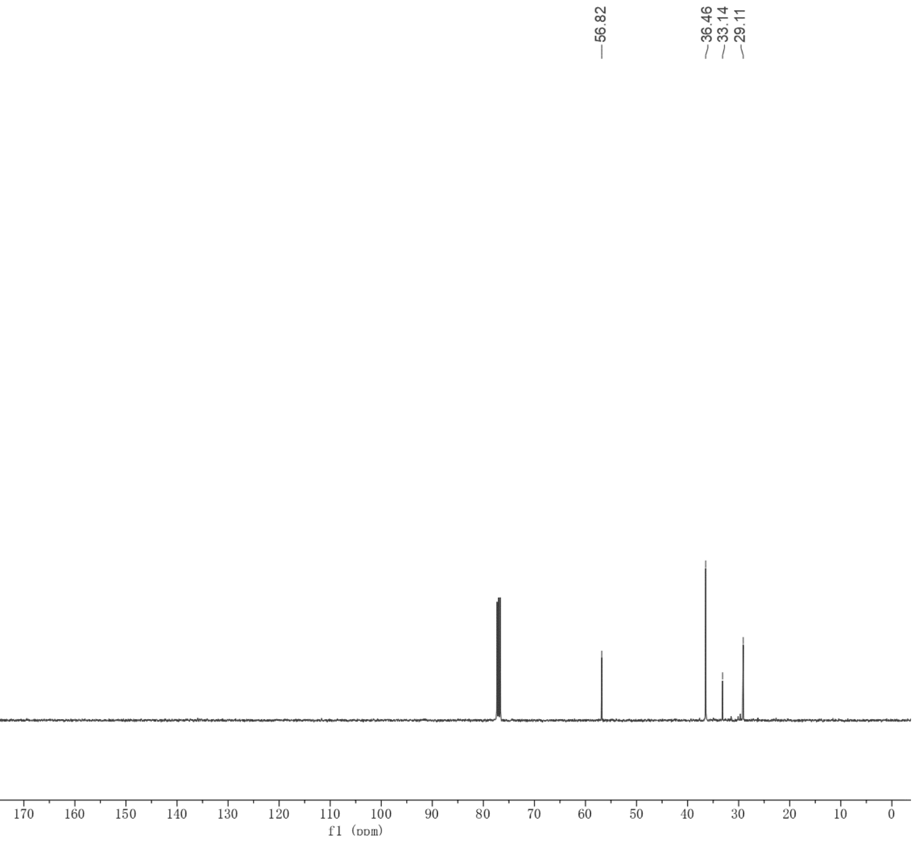

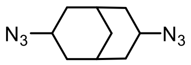

47

^{1}H NMR (400 MHz, $CDCl_3$) of **48**

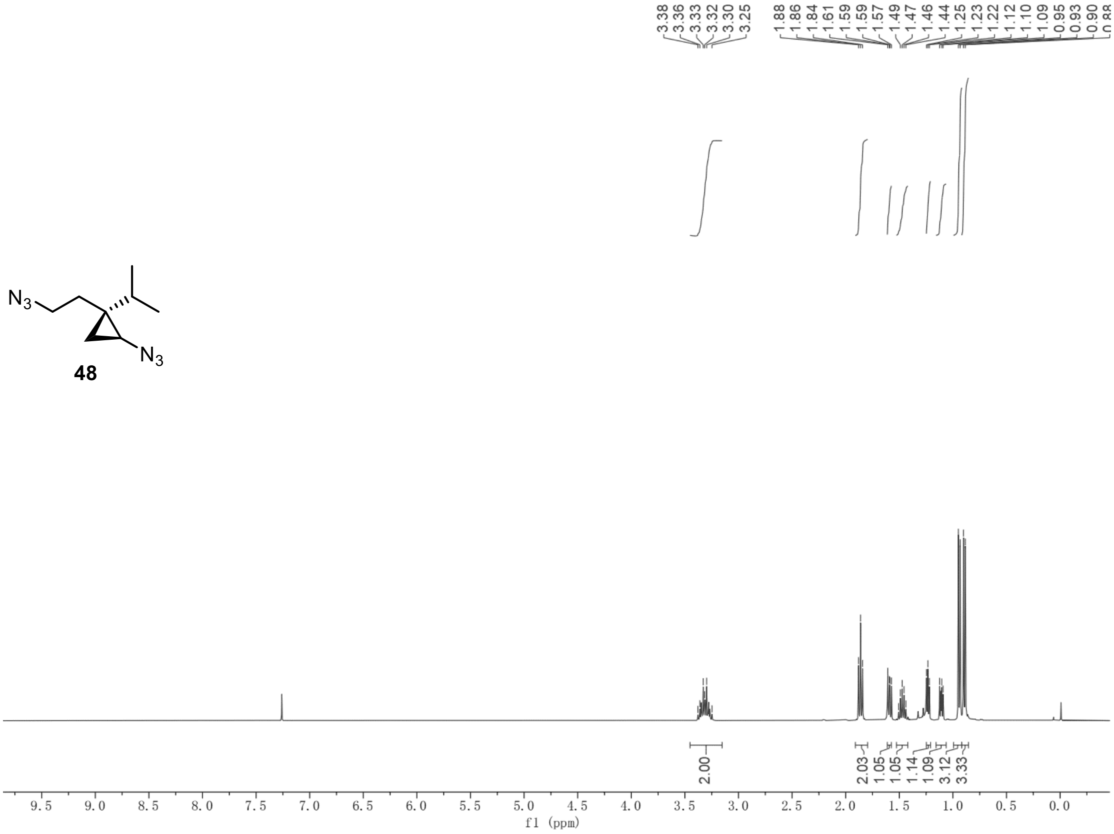

^{13}C NMR (100 MHz, $CDCl_3$) of **48**

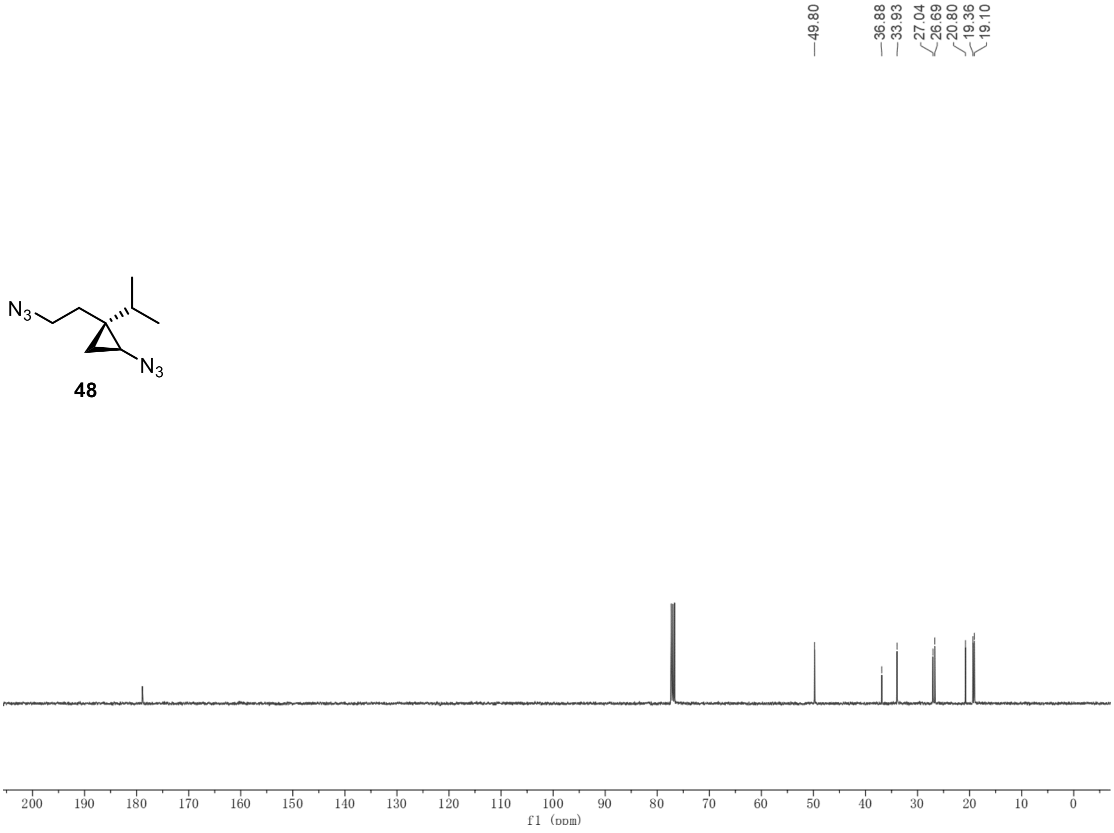

^{1}H NMR (400 MHz, $CDCl_3$) of **49**

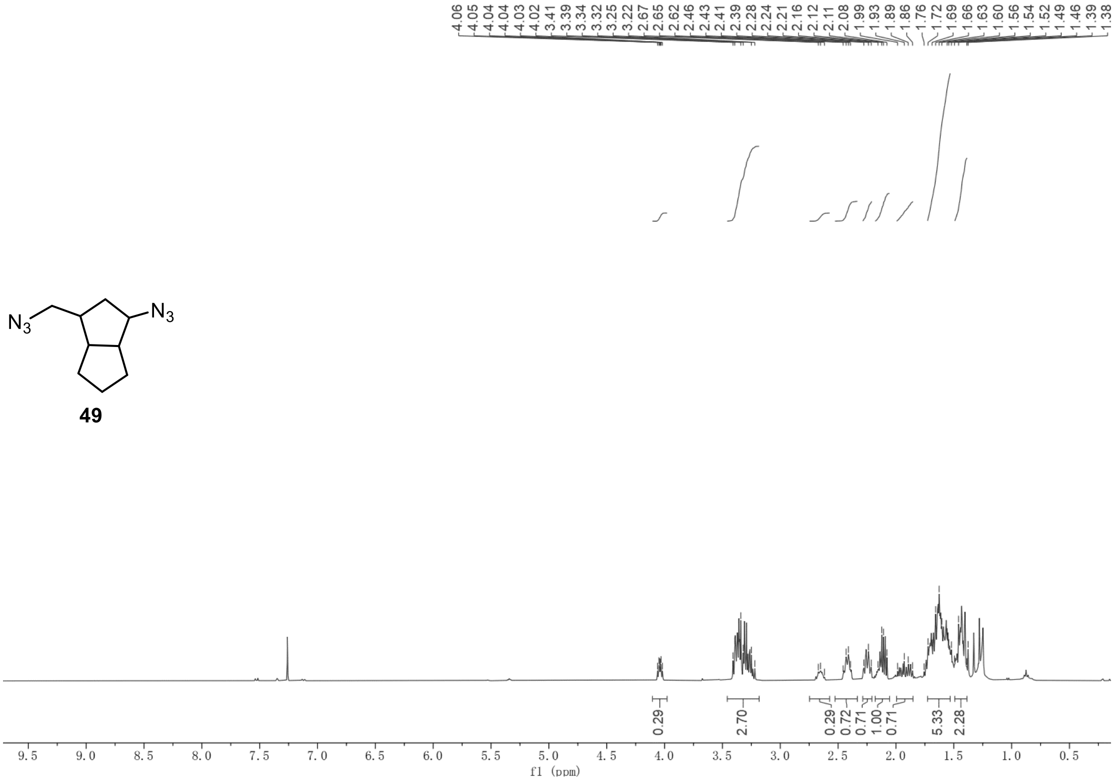

^{13}C NMR (100 MHz, $CDCl_3$) of **49**

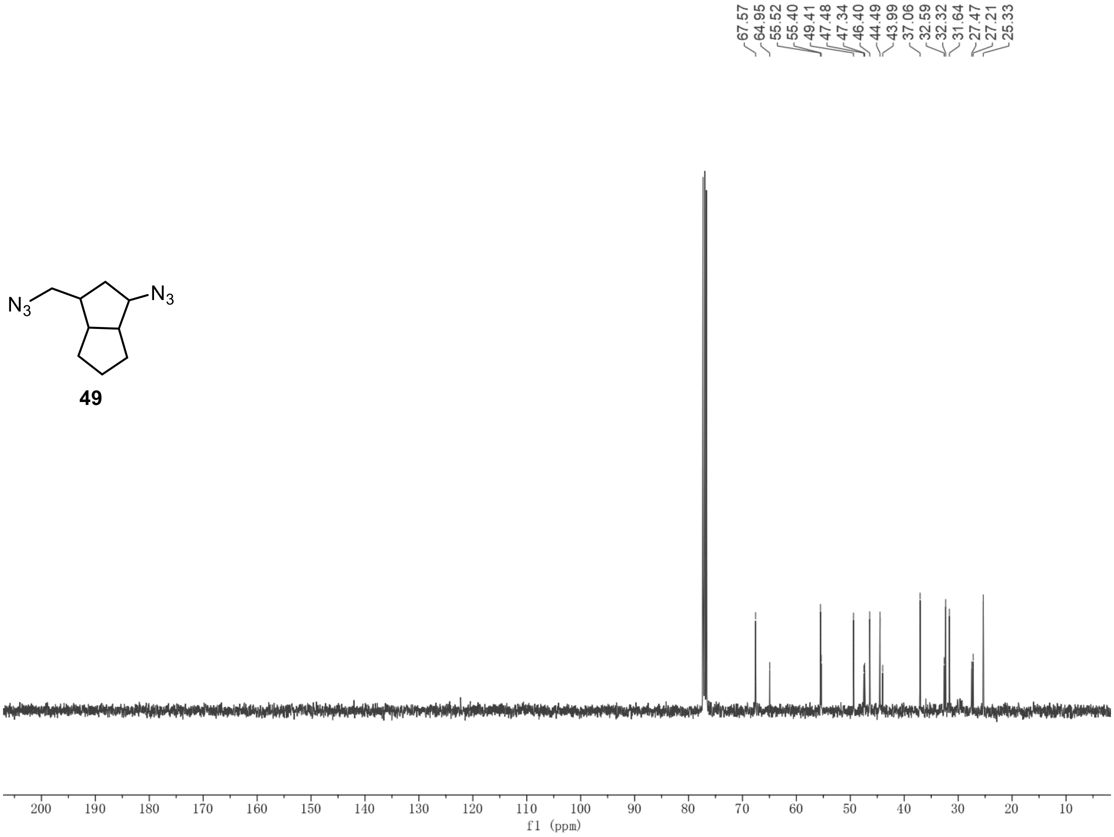

^{1}H NMR (400 MHz, $CDCl_3$) of **50**

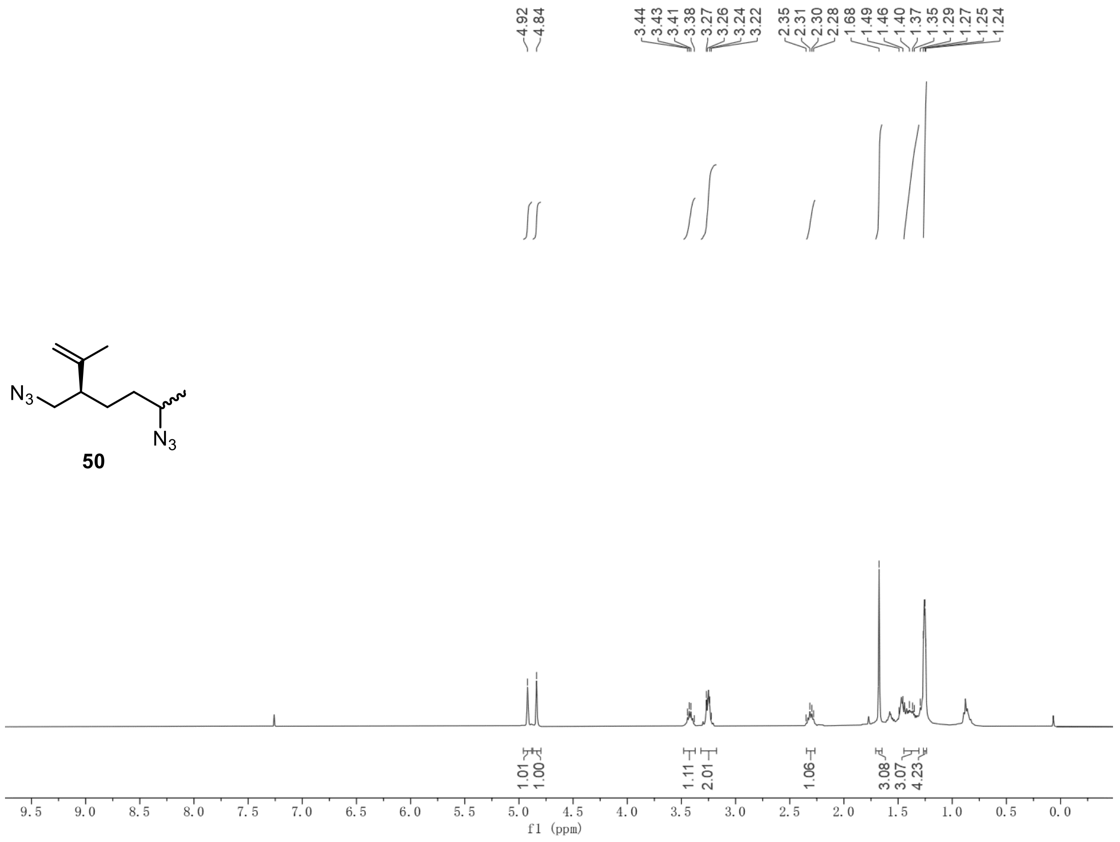

^{13}C NMR (100 MHz, $CDCl_3$) of **50**

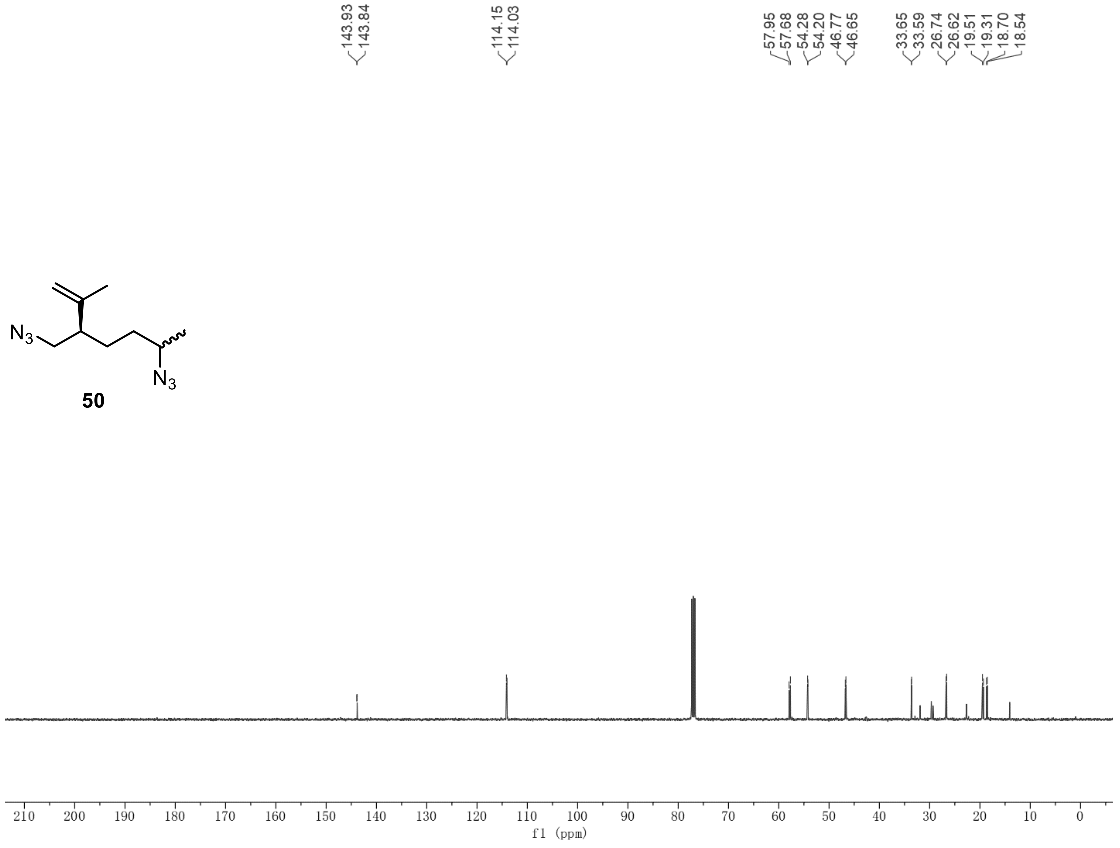

¹H NMR (400 MHz, $CDCl_3$) of **51**

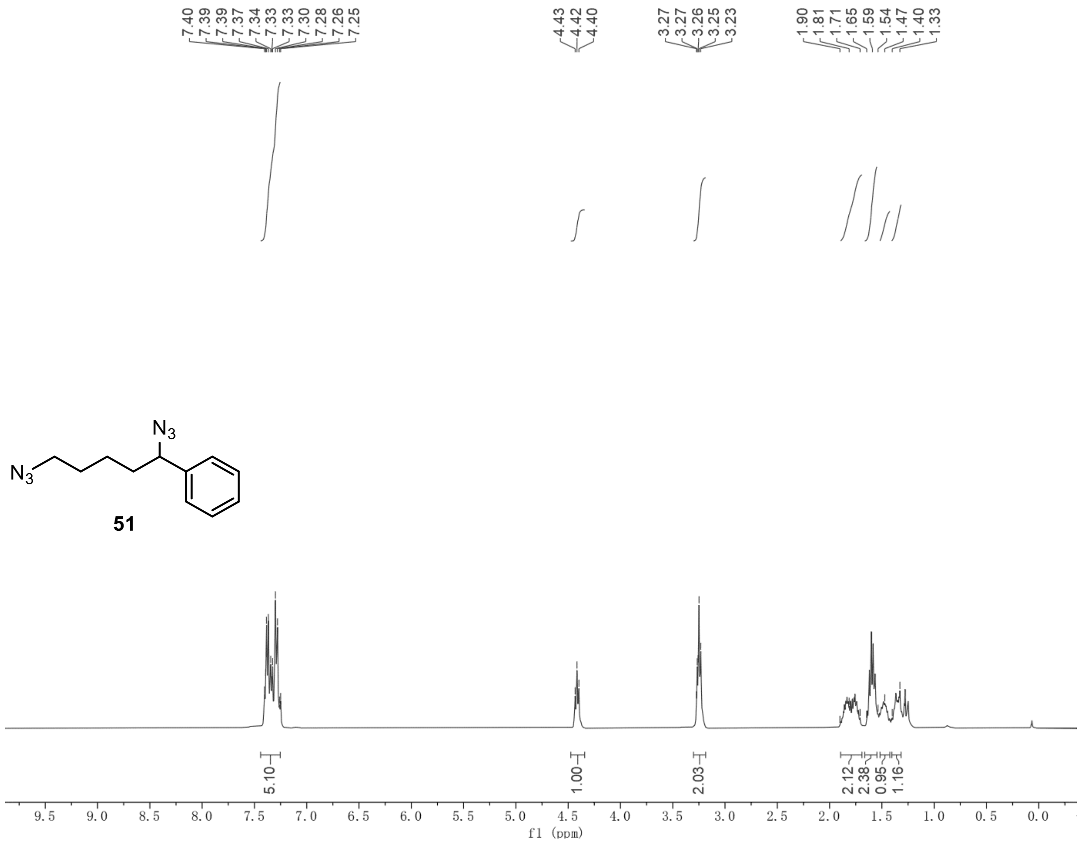

¹³C NMR (100 MHz, $CDCl_3$) of **51**

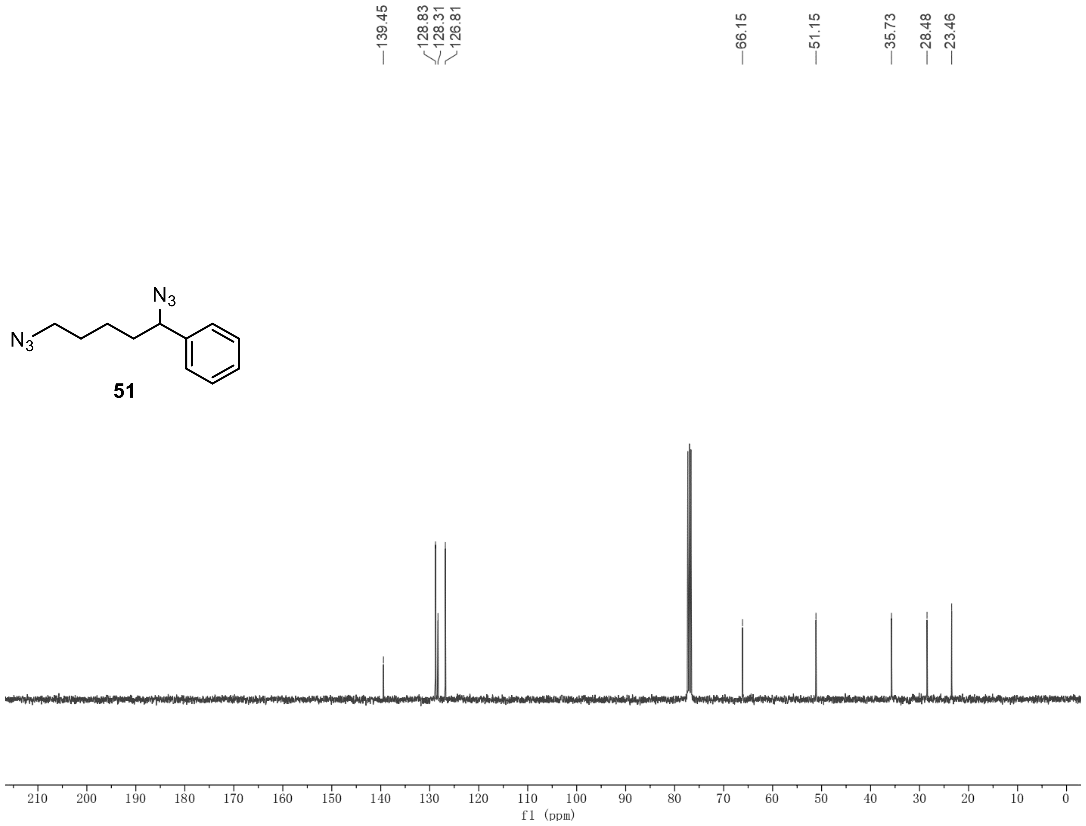

^{1}H NMR (400 MHz, $CDCl_3$) of **52**

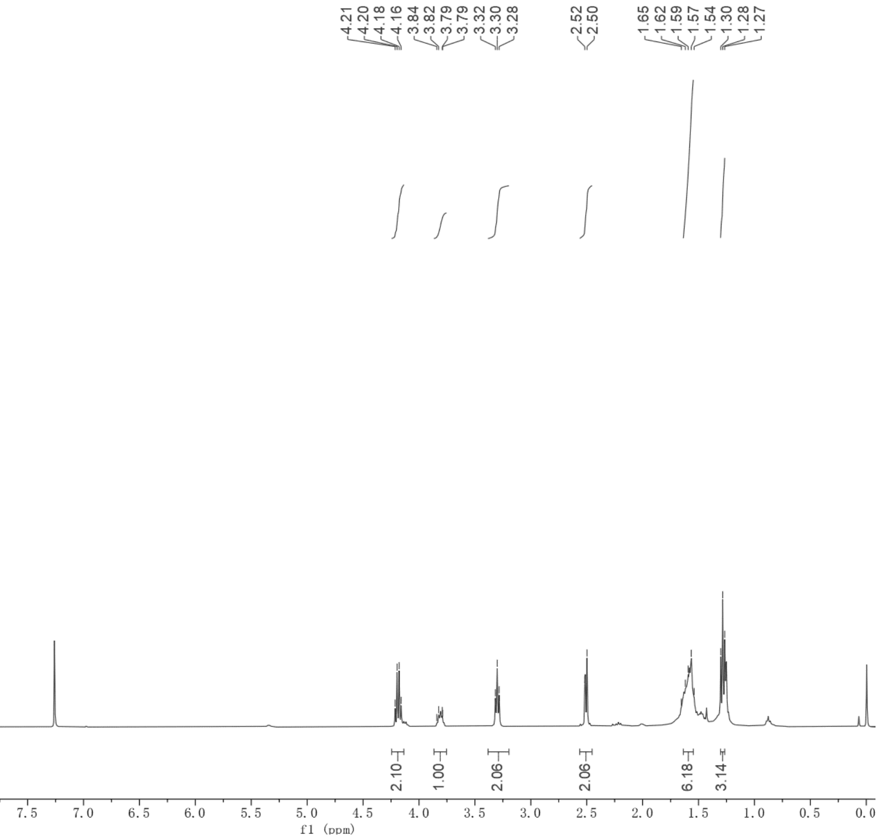

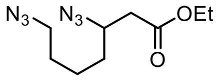

52

^{13}C NMR (100 MHz, $CDCl_3$) of **52**

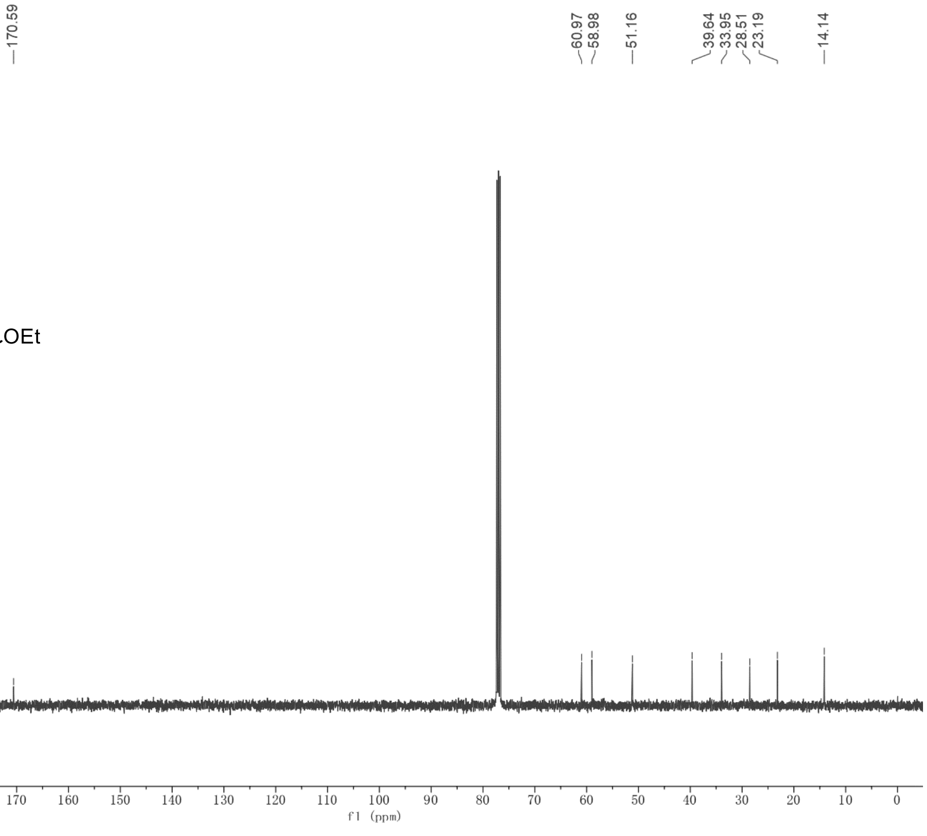

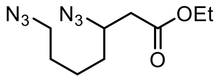

52

^{1}H NMR (400 MHz, $CDCl_3$) of **53**

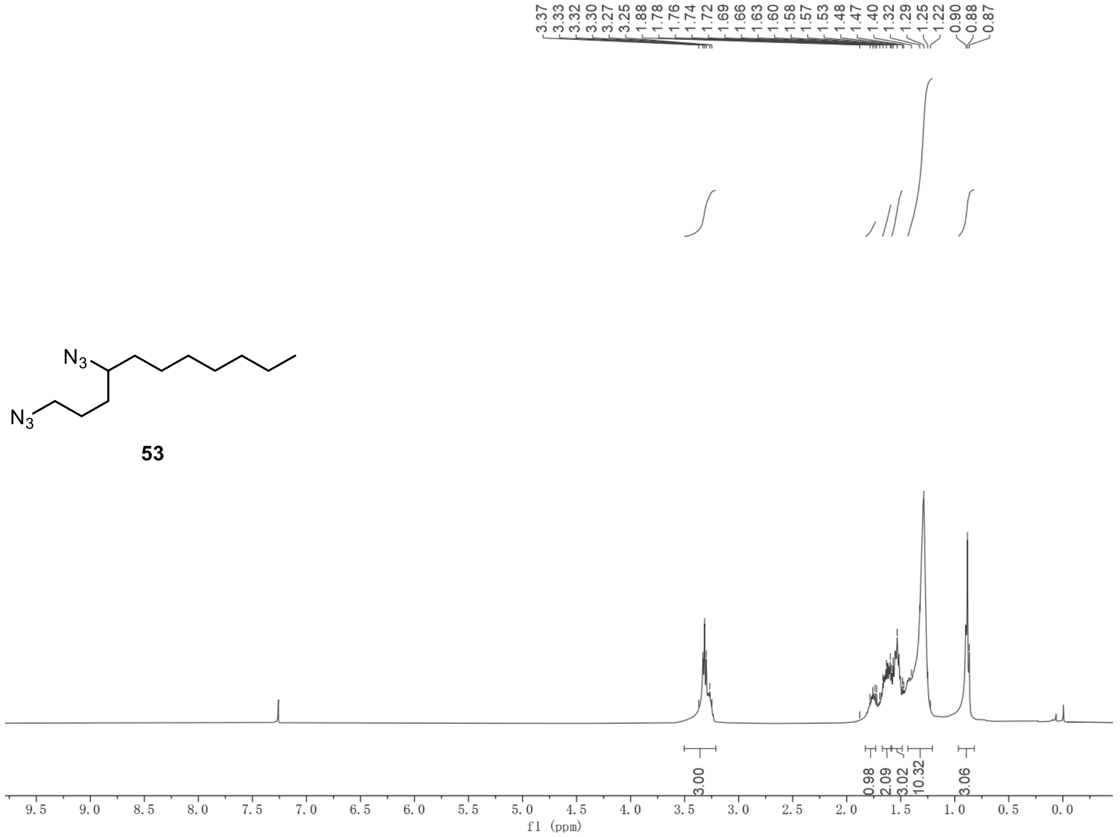

^{13}C NMR (100 MHz, $CDCl_3$) of **53**

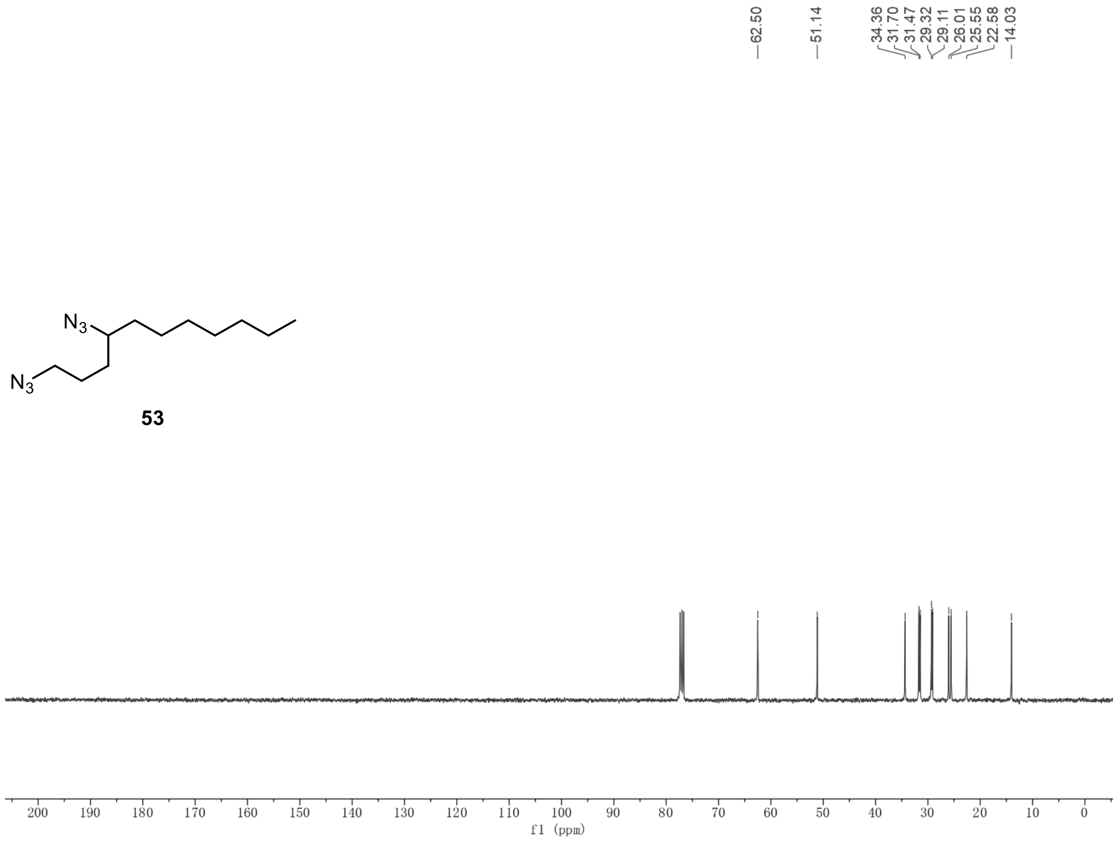

^{1}H NMR (400 MHz, $CDCl_3$) of **54**

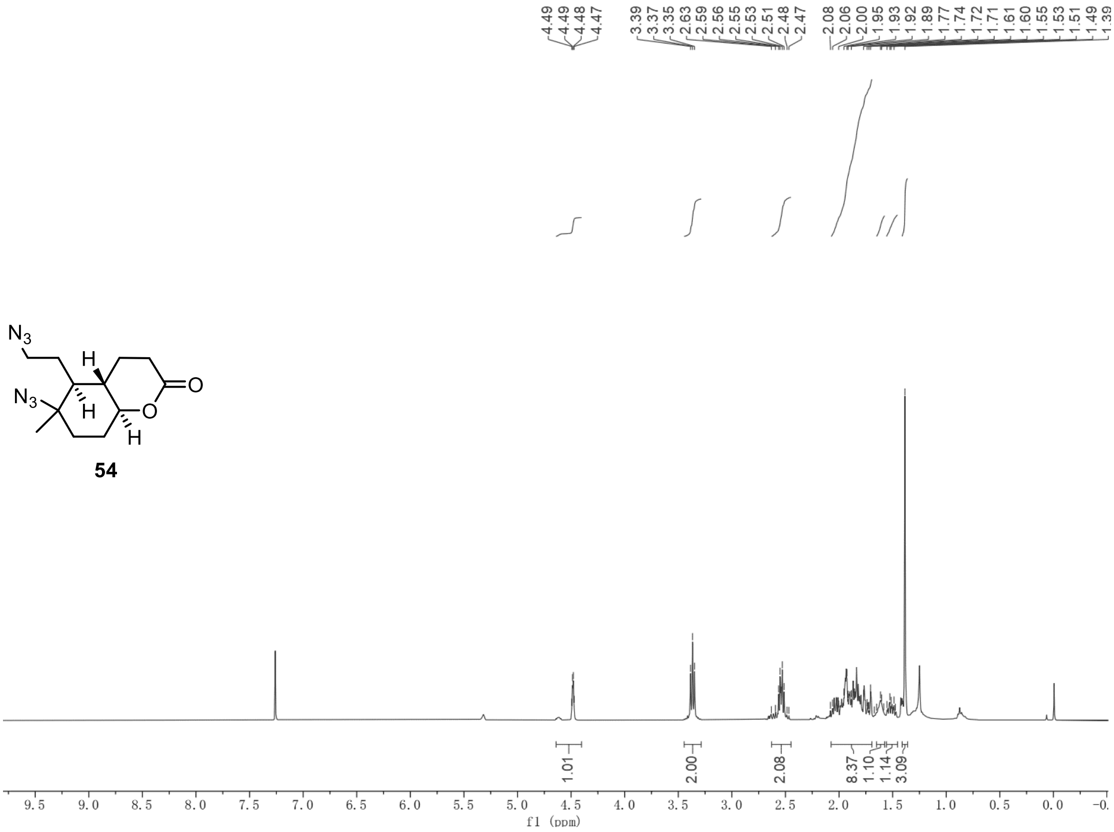

54

^{13}C NMR (100 MHz, $CDCl_3$) of **54**

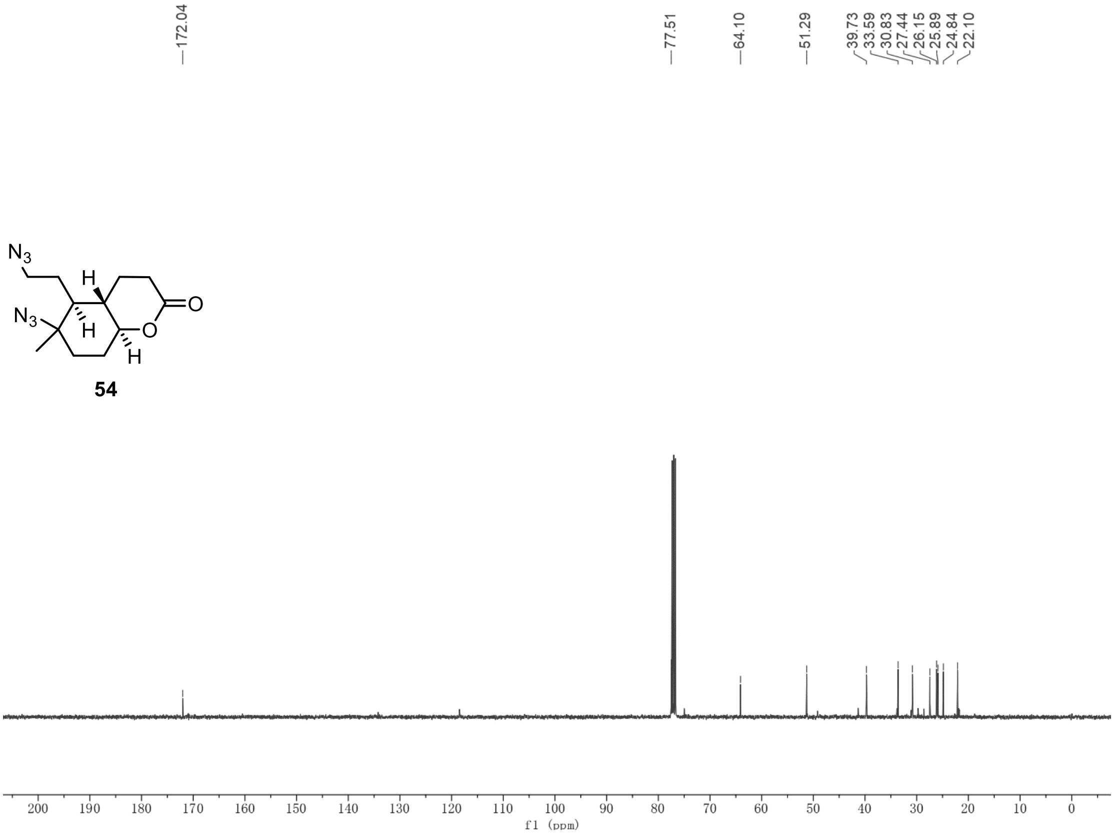

54

^{1}H NMR (400 MHz, $CDCl_3$) of **54’**

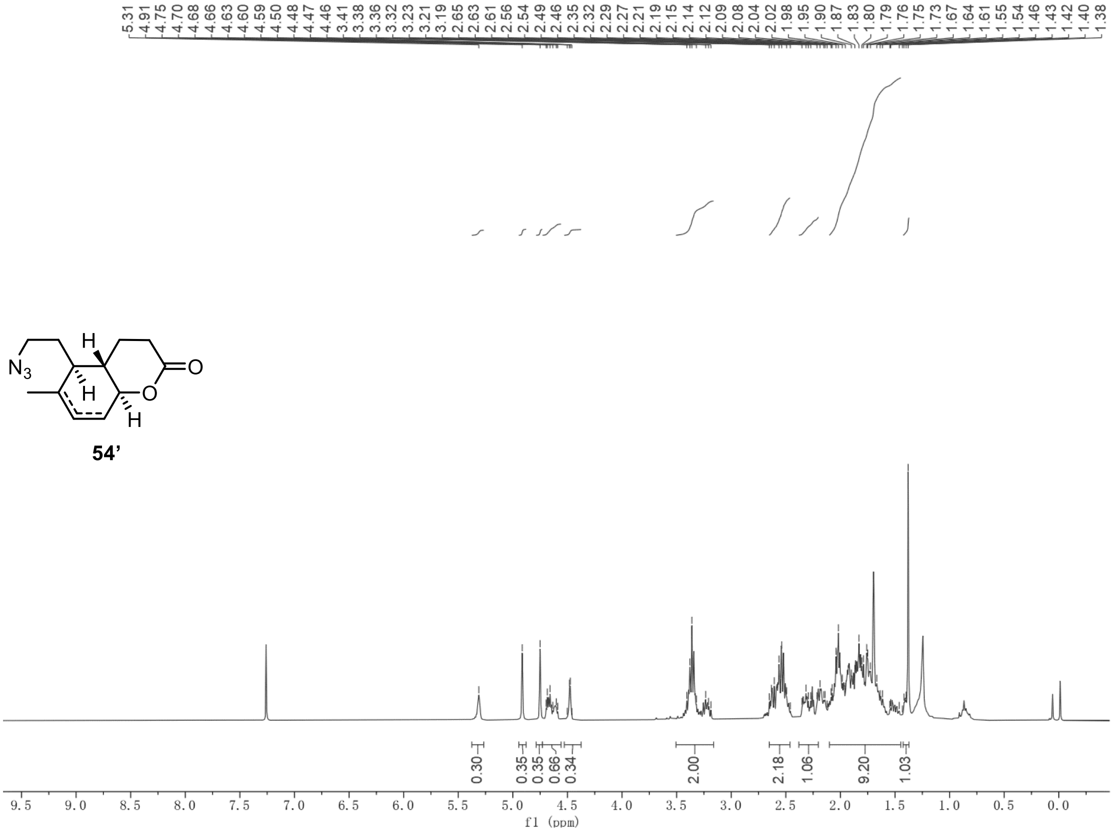

54’

^{13}C NMR (100 MHz, $CDCl_3$) of **54’**

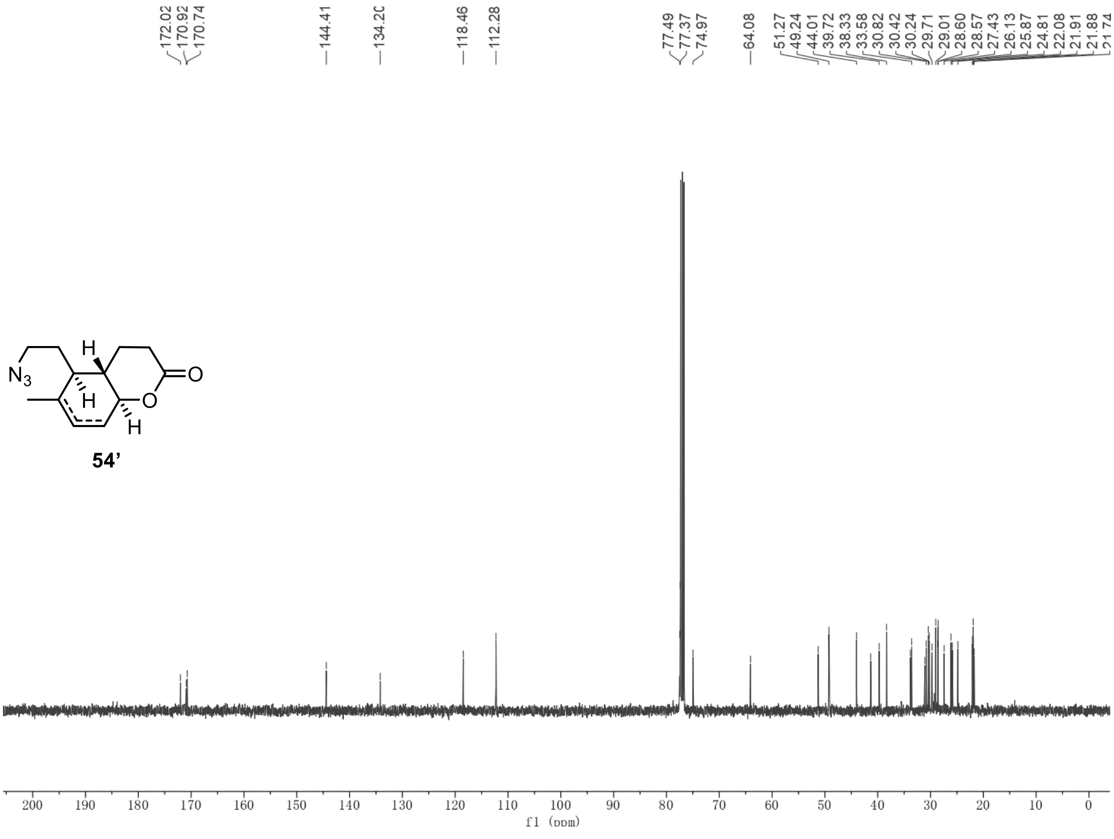

54’

^{1}H NMR (400 MHz, CDCl$_3$) of **55**

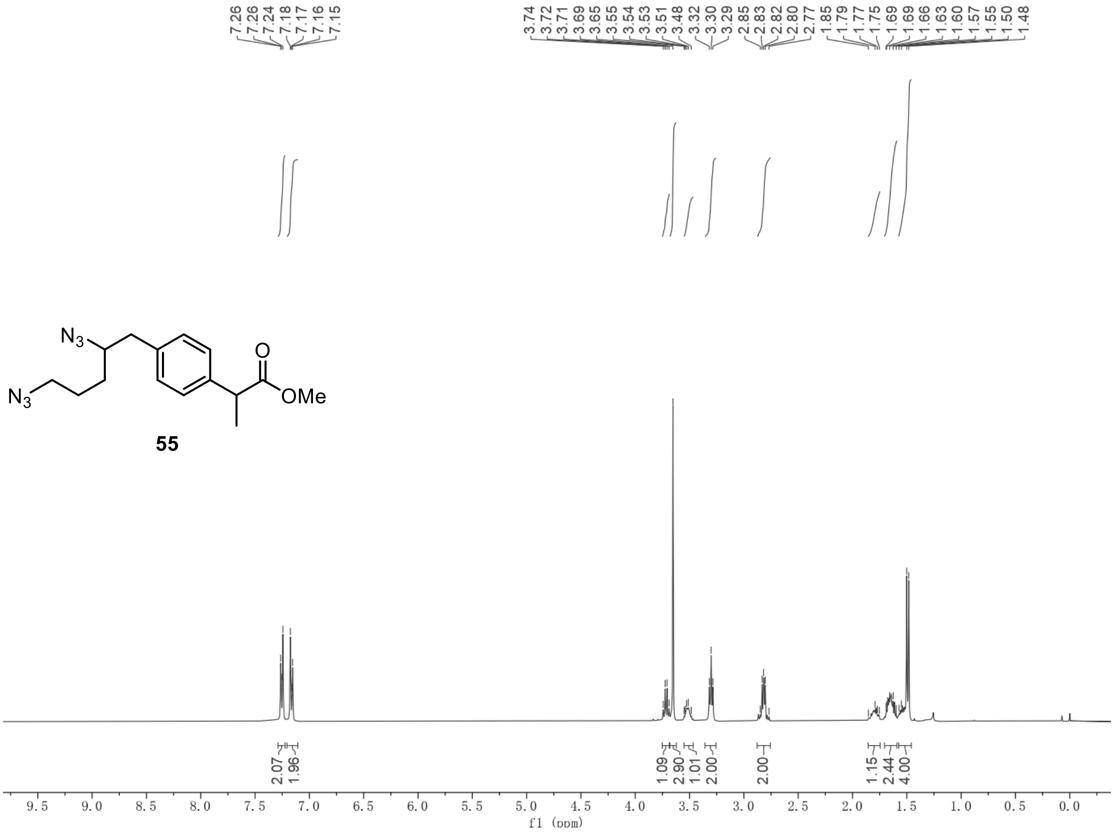

^{13}C NMR (100 MHz, CDCl$_3$) of **55**

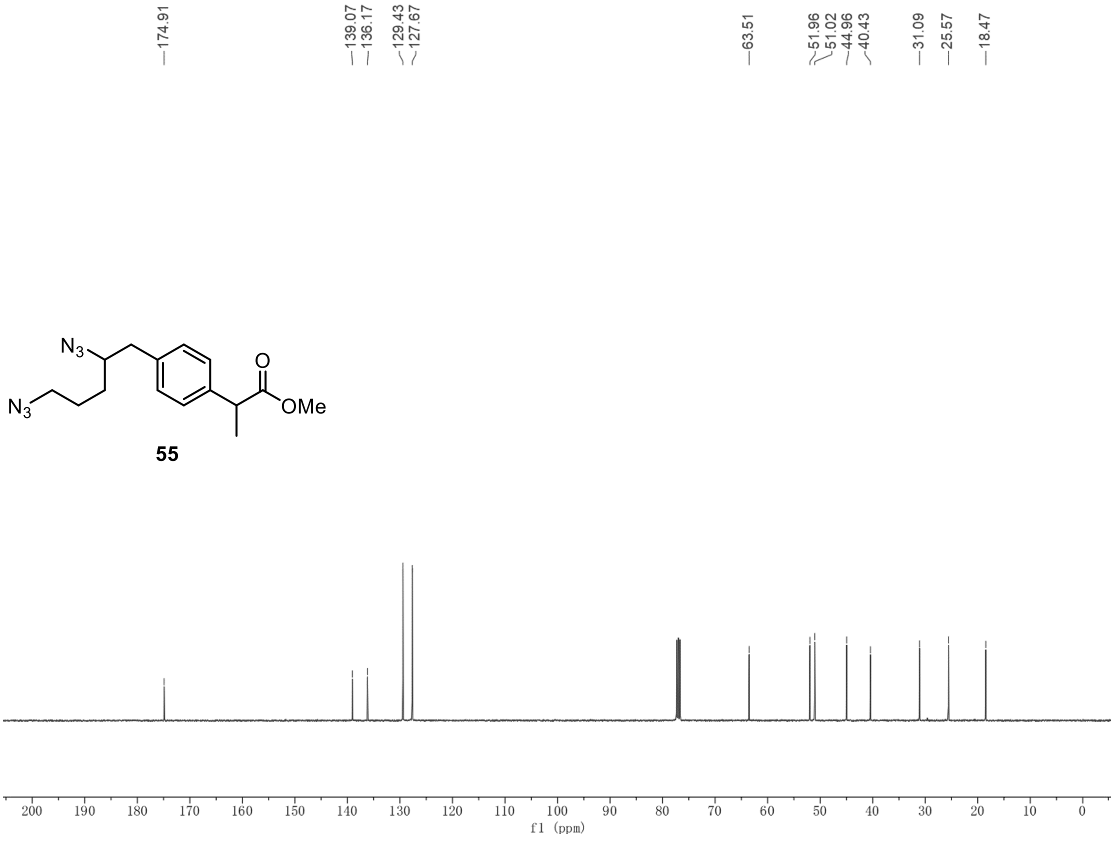

^{1}H NMR (400 MHz, $CDCl_3$) of **56**

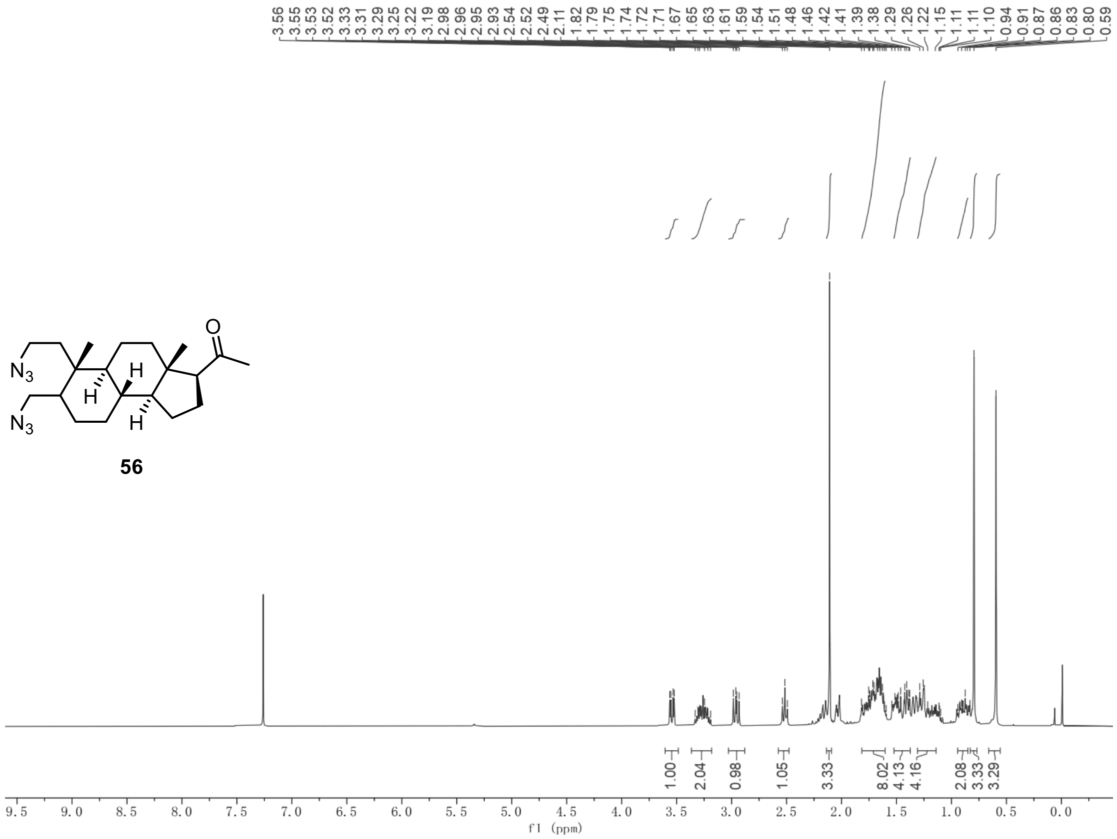

^{13}C NMR (100 MHz, $CDCl_3$) of **56**

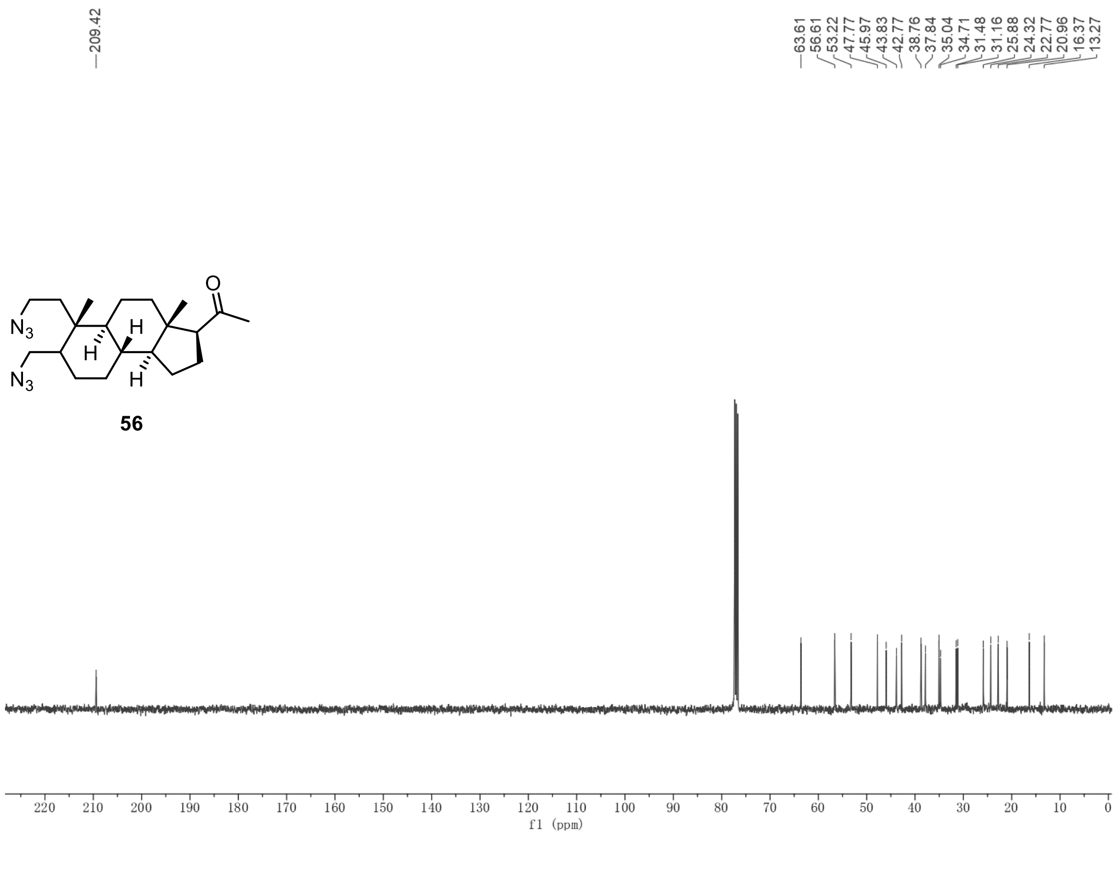

^{1}H NMR (400 MHz, $CDCl_3$) of **57**

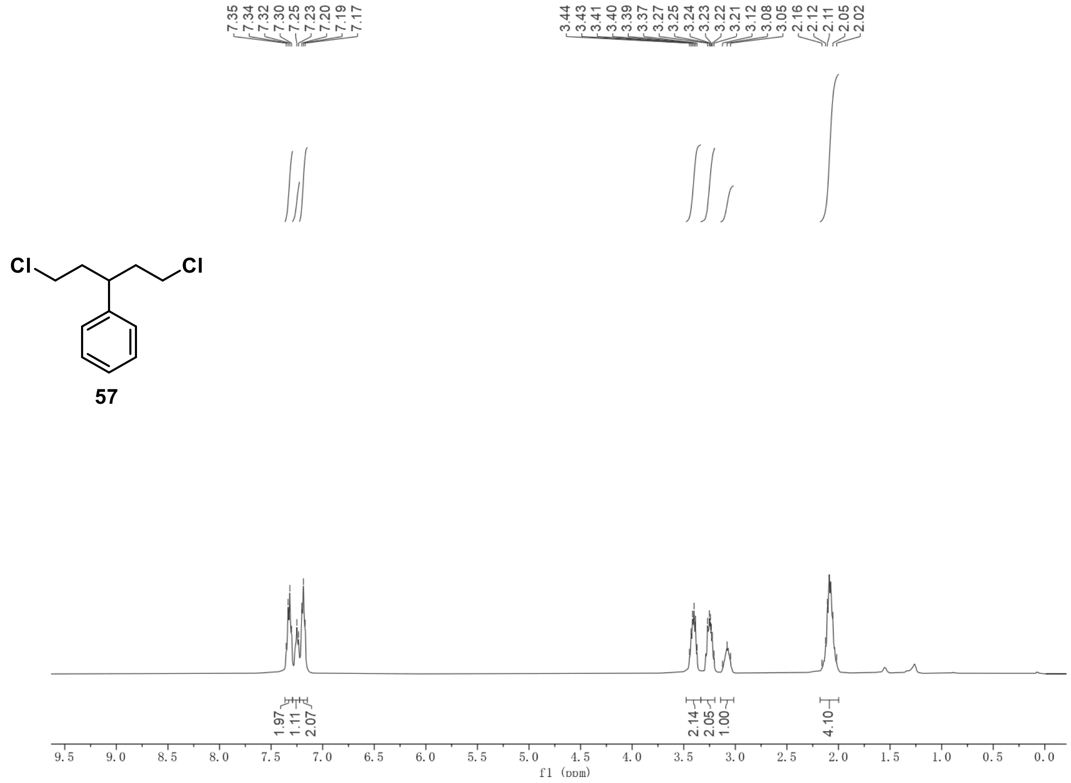

^{13}C NMR (100 MHz, $CDCl_3$) of **57**

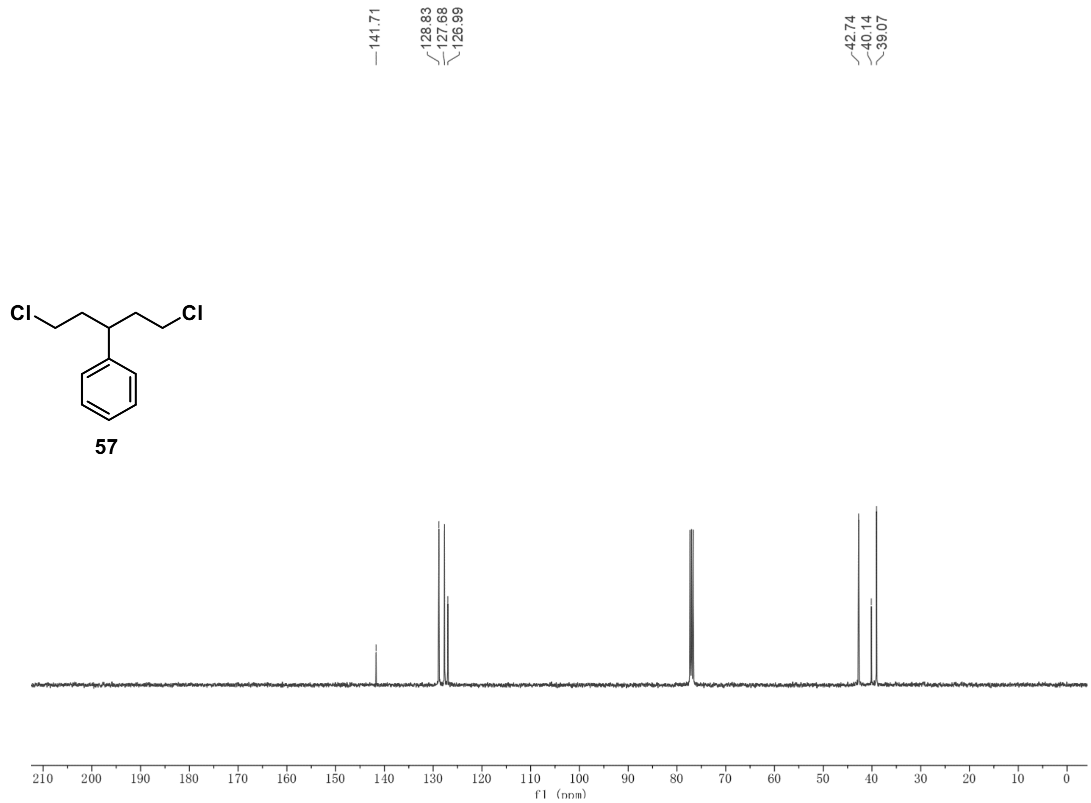

^{1}**H NMR** (400 MHz, $CDCl_3$) of **58**

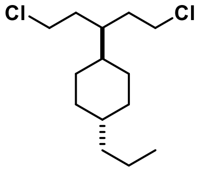

58

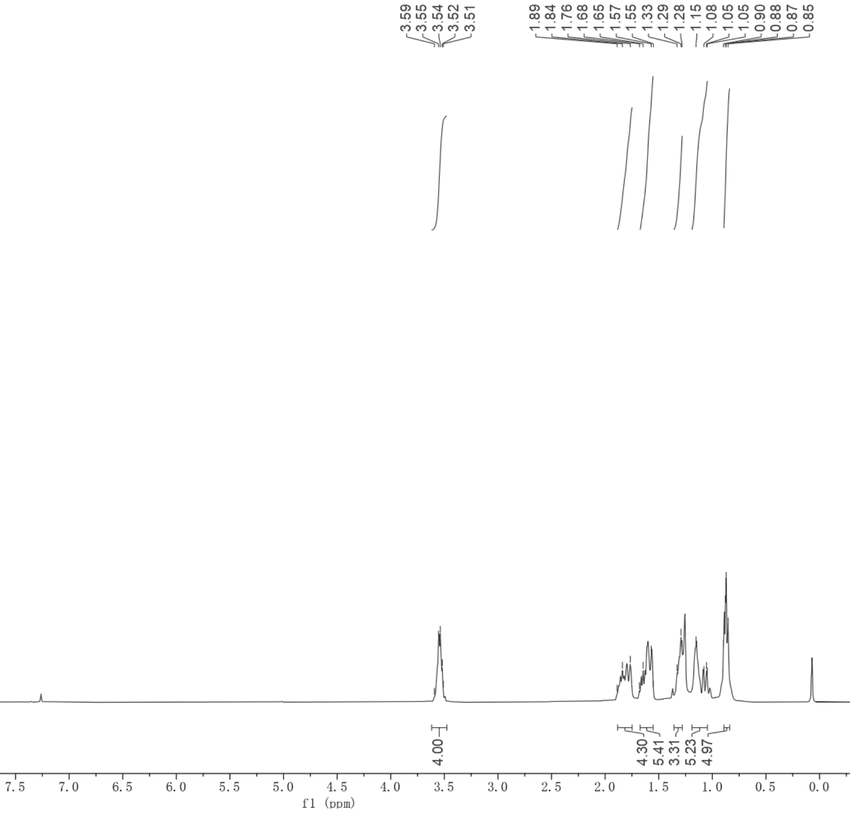

^{13}**C NMR** (100 MHz, $CDCl_3$) of **58**

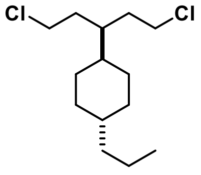

58

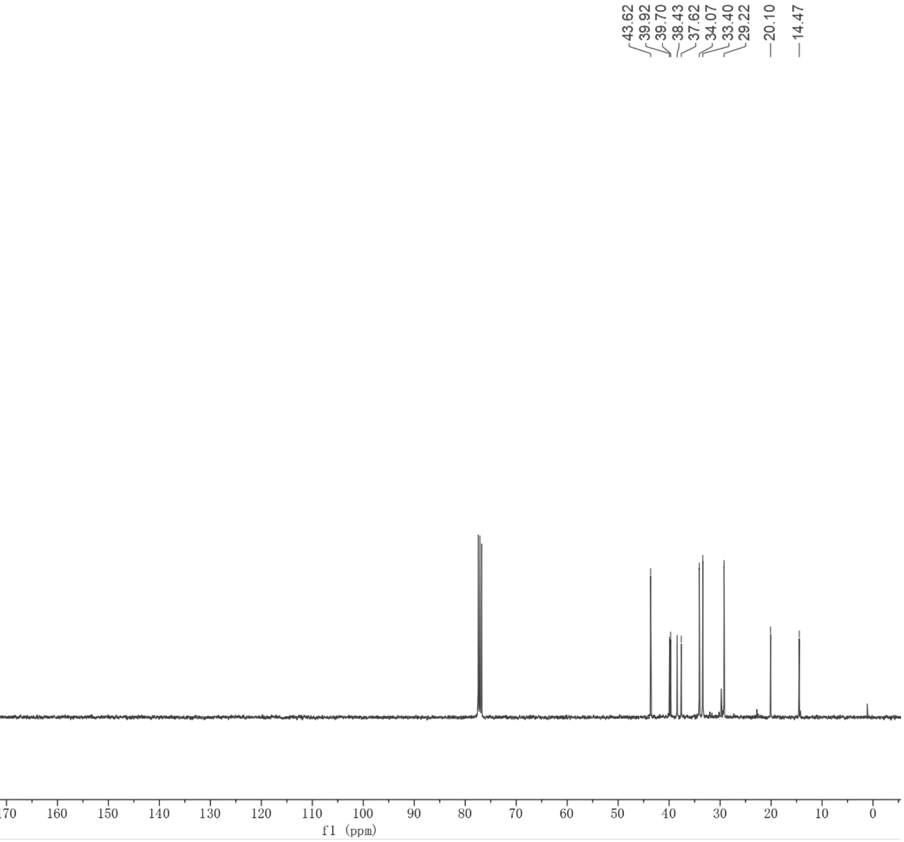

^{1}H NMR (400 MHz, $CDCl_3$) of **59**

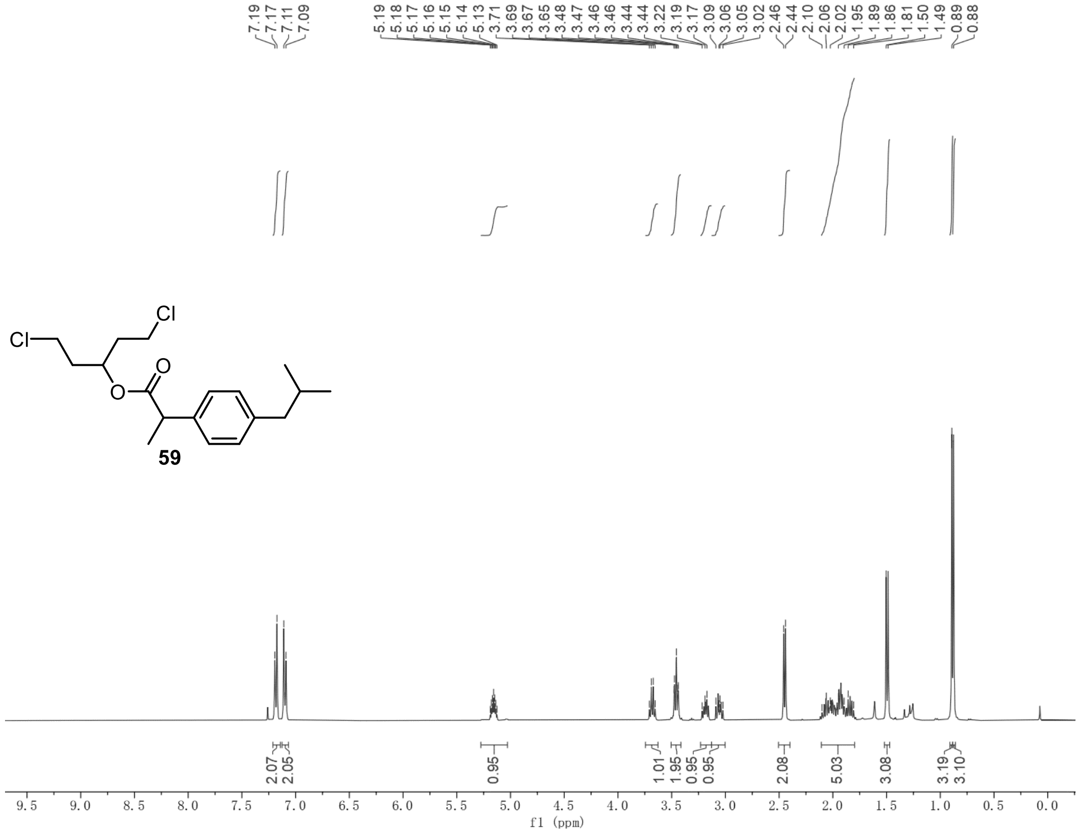

^{13}C NMR (100 MHz, $CDCl_3$) of **59**

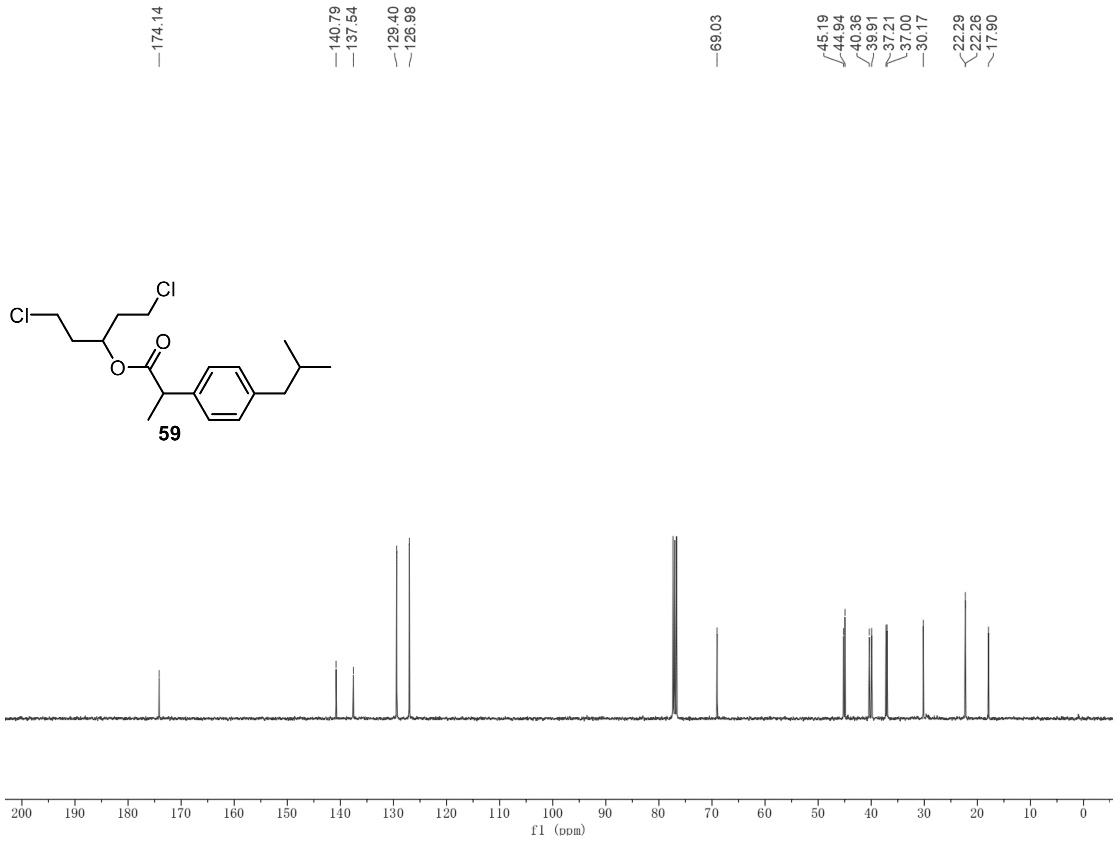

¹H NMR (400 MHz, $CDCl_3$) of **60**

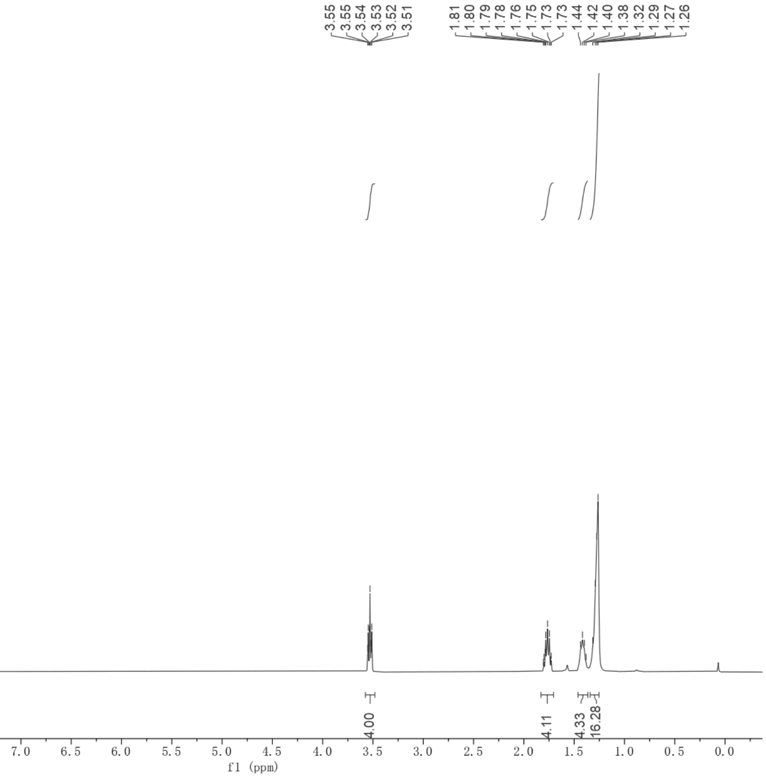

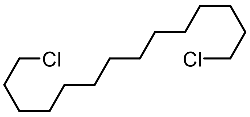

60

¹³C NMR (100 MHz, $CDCl_3$) of **60**

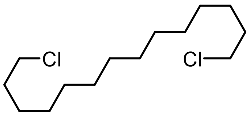

60

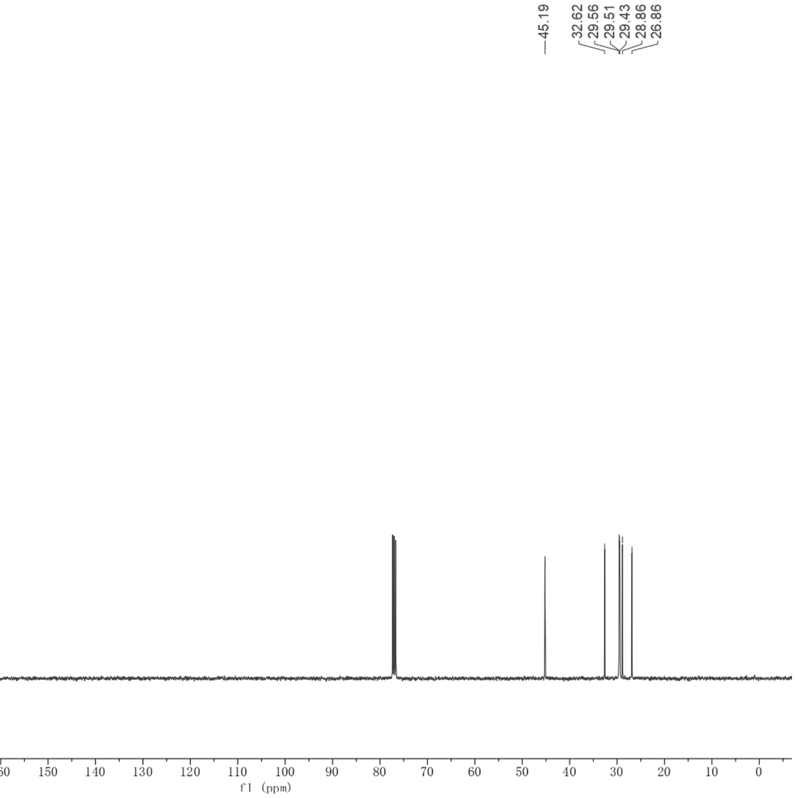

^{1}H NMR (400 MHz, $CDCl_3$) of **61**

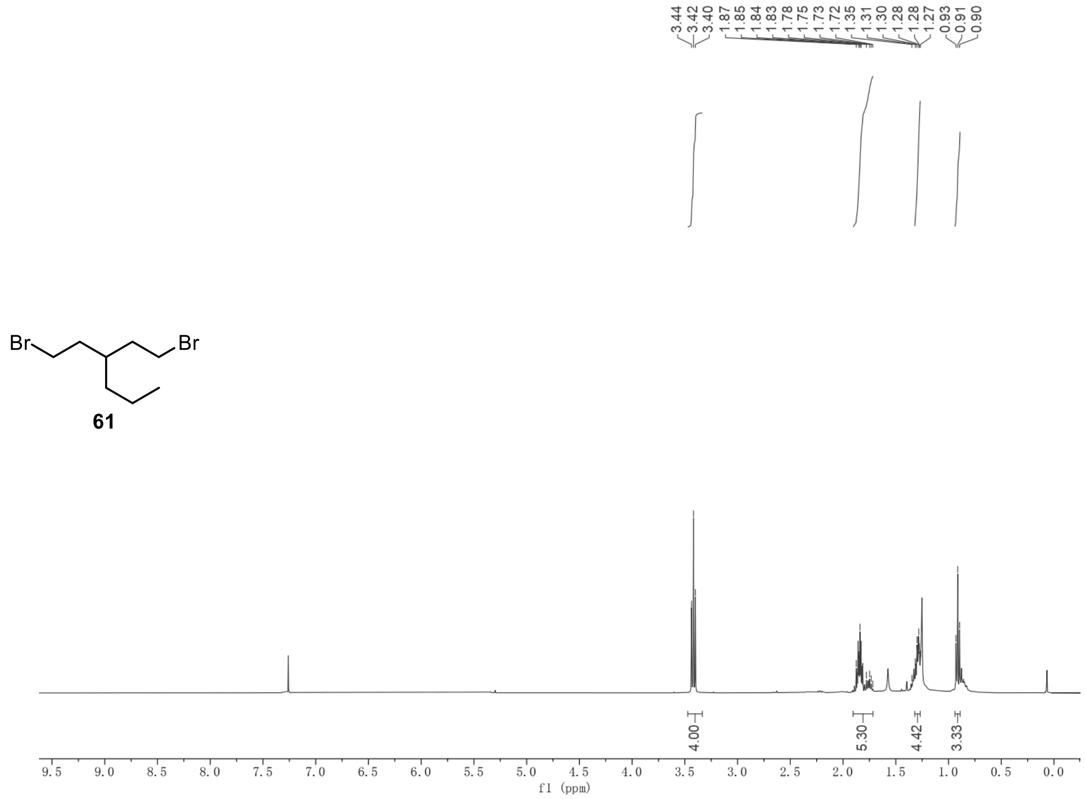

^{13}C NMR (100 MHz, $CDCl_3$) of **61**

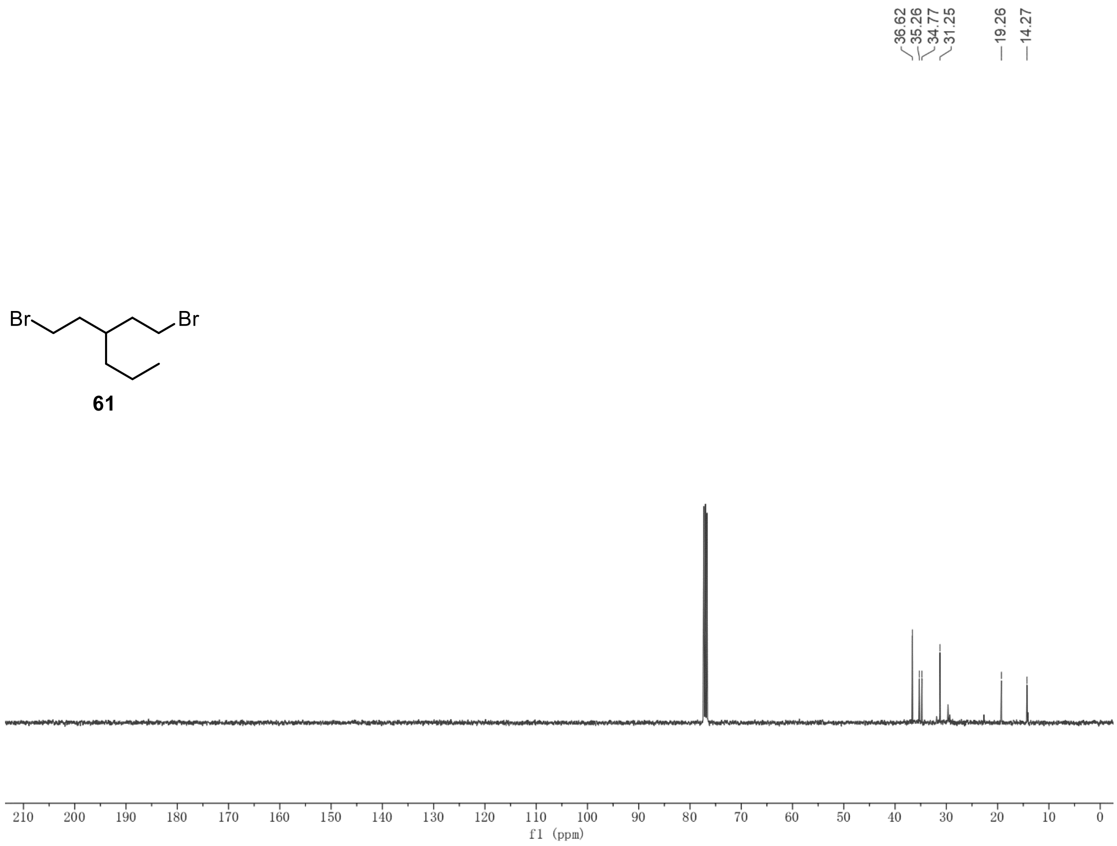

^{1}H NMR (400 MHz, $CDCl_3$) of **62**

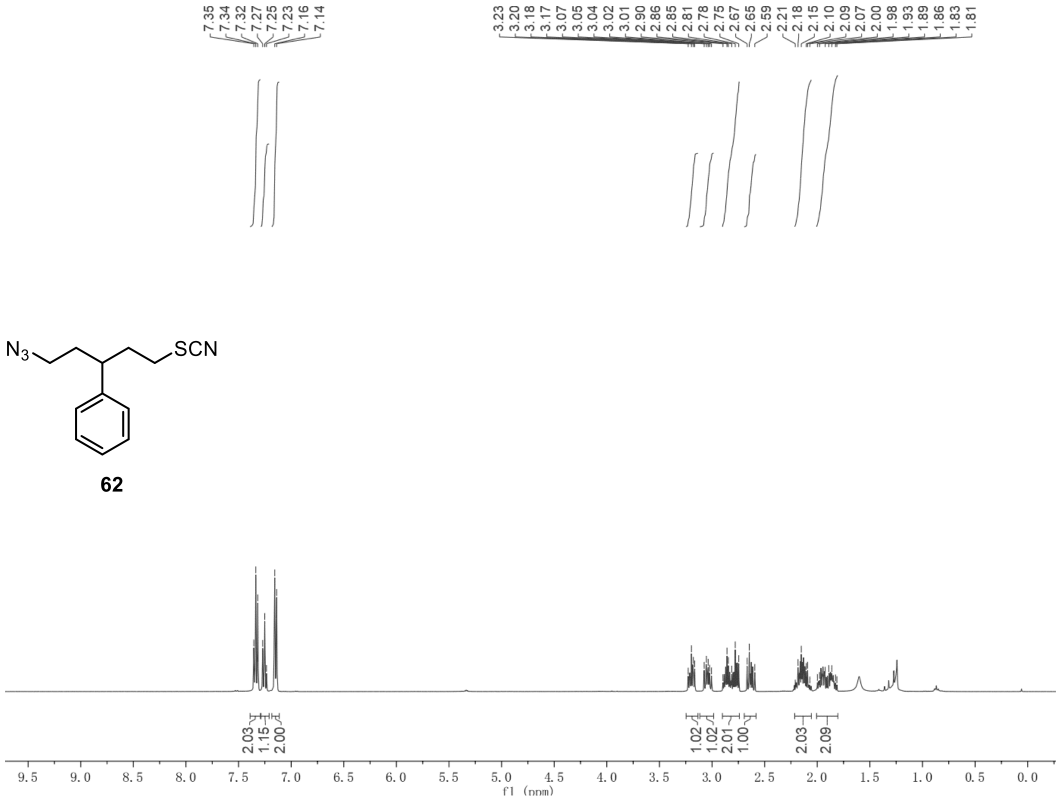

62

^{13}C NMR (100 MHz, $CDCl_3$) of **62**

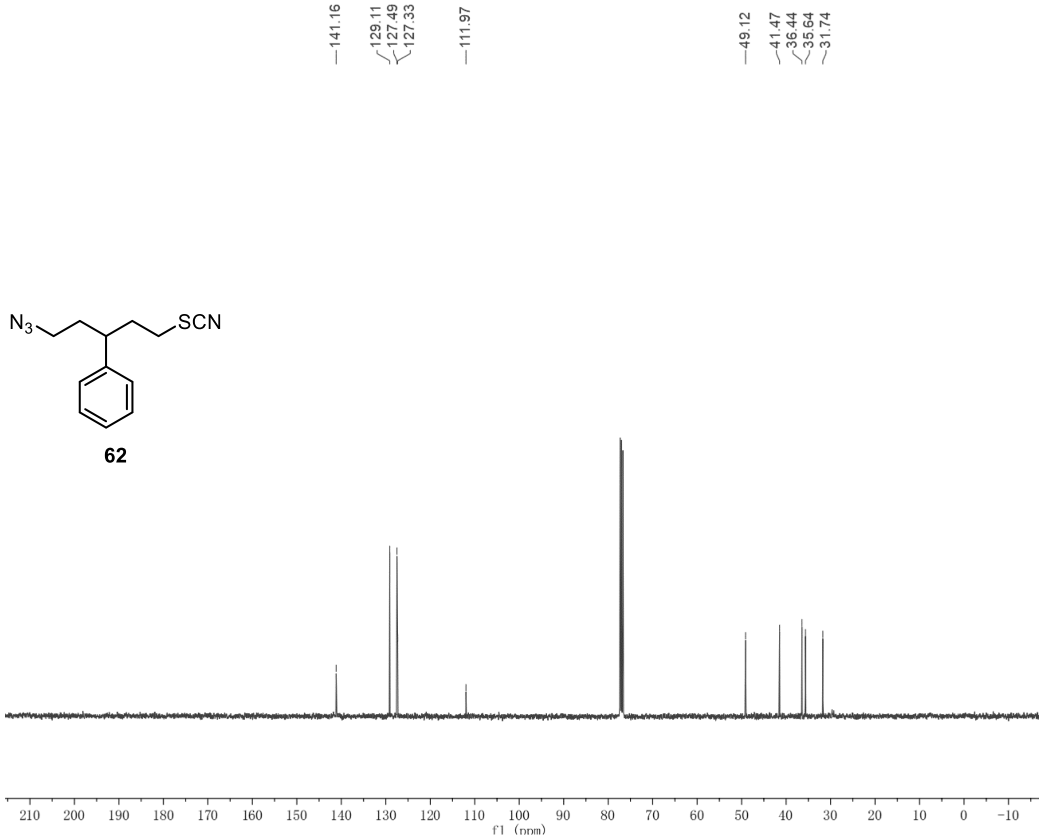

62

^{1}H NMR (400 MHz, $CDCl_3$) of **63**

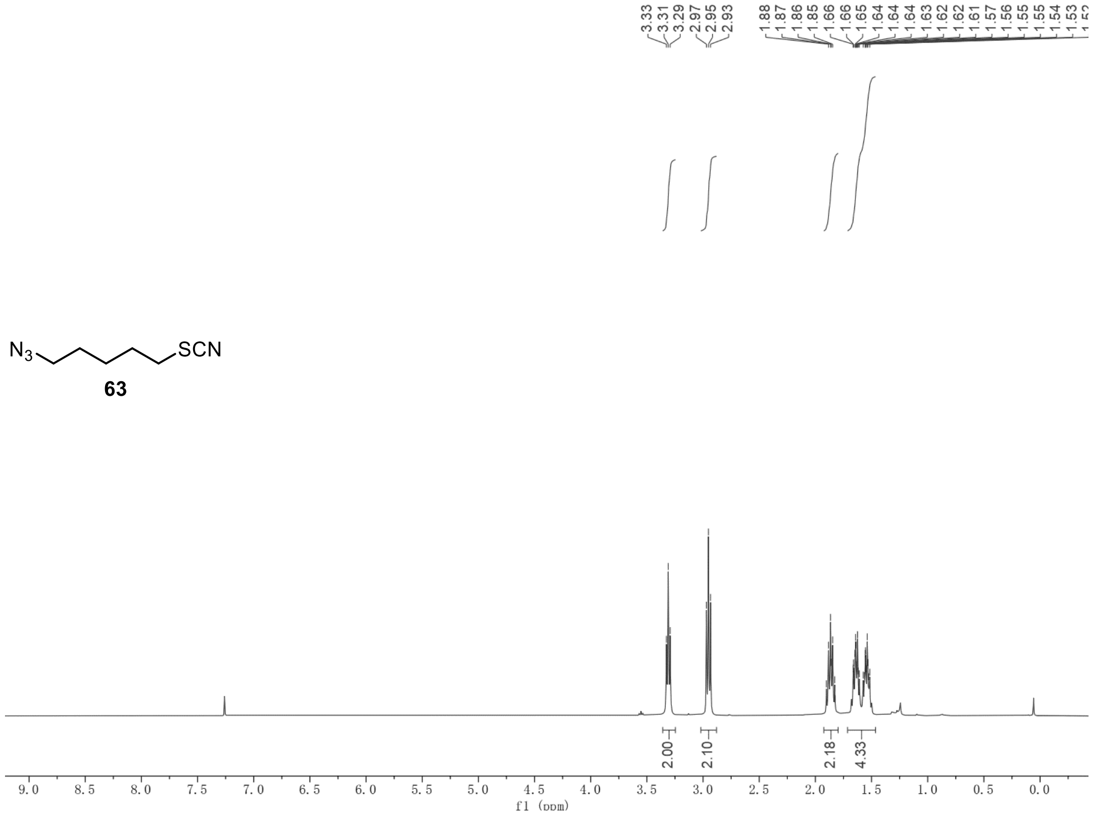

^{13}C NMR (100 MHz, $CDCl_3$) of **63**

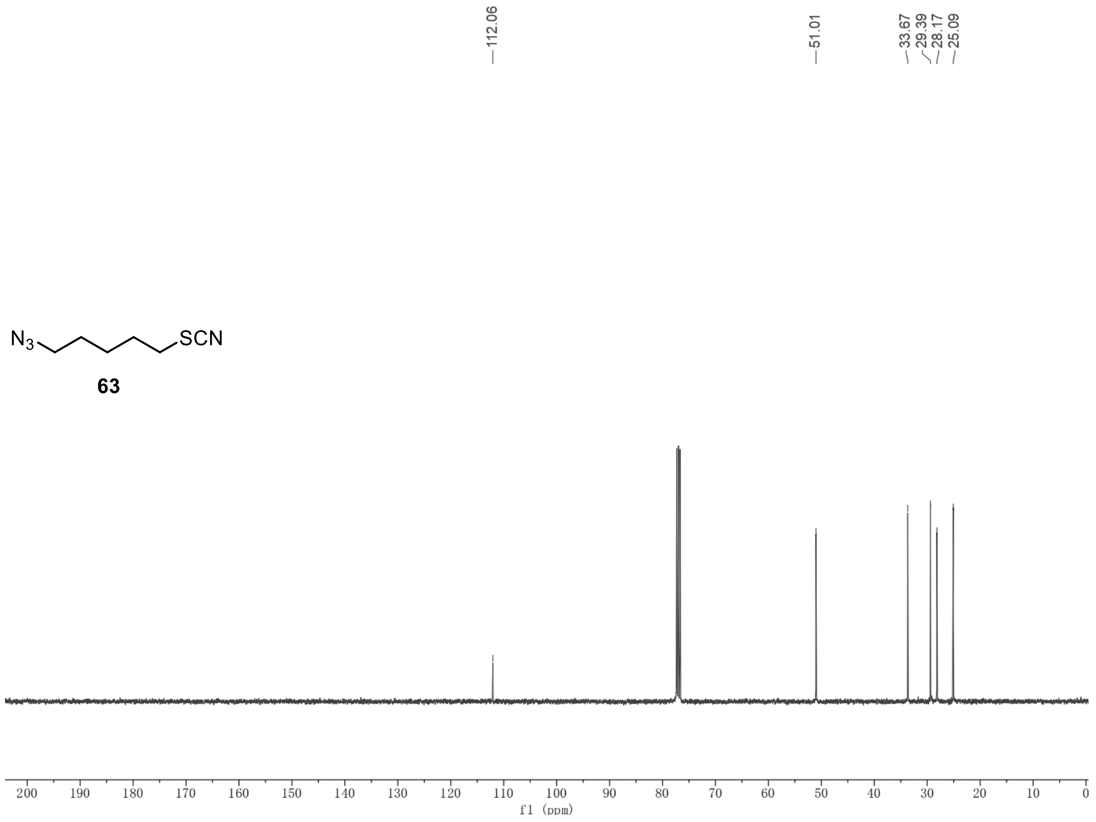

^{1}H NMR (400 MHz, $CDCl_3$) of **64**

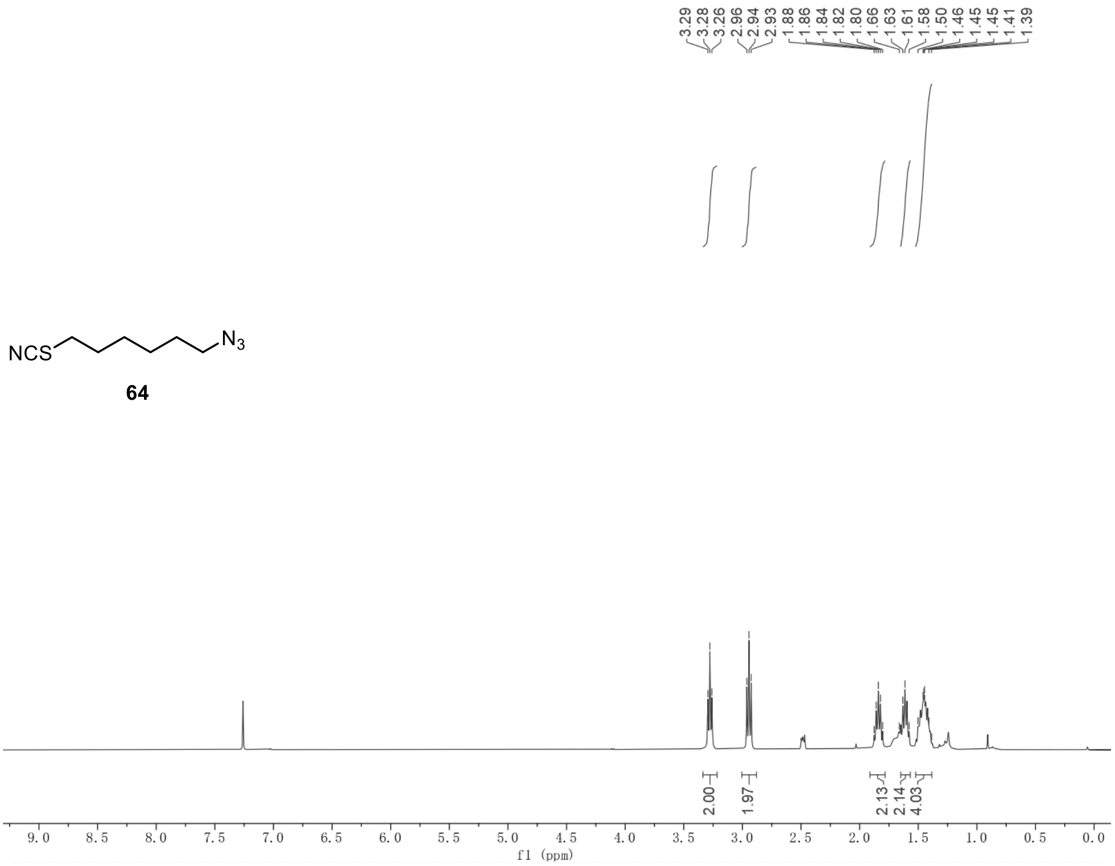

^{13}C NMR (100 MHz, $CDCl_3$) of **64**

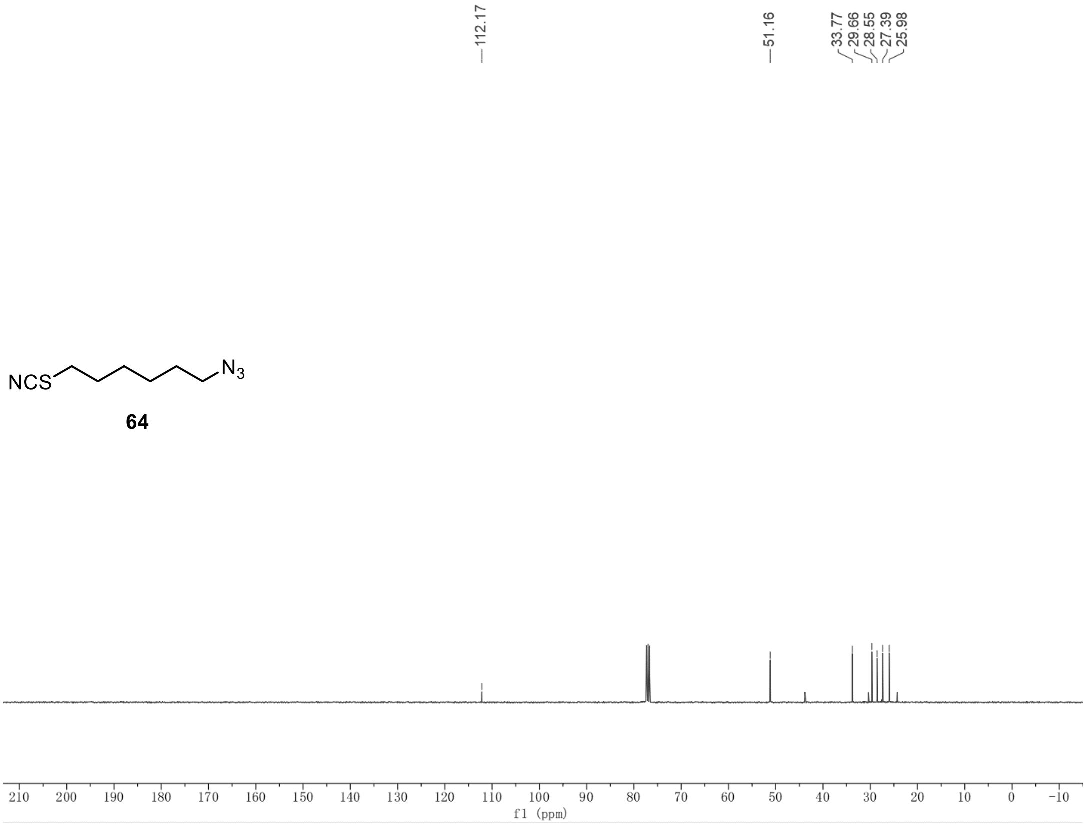

^{1}H NMR (400 MHz, $CDCl_3$) of **65**

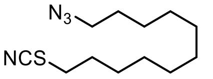

65

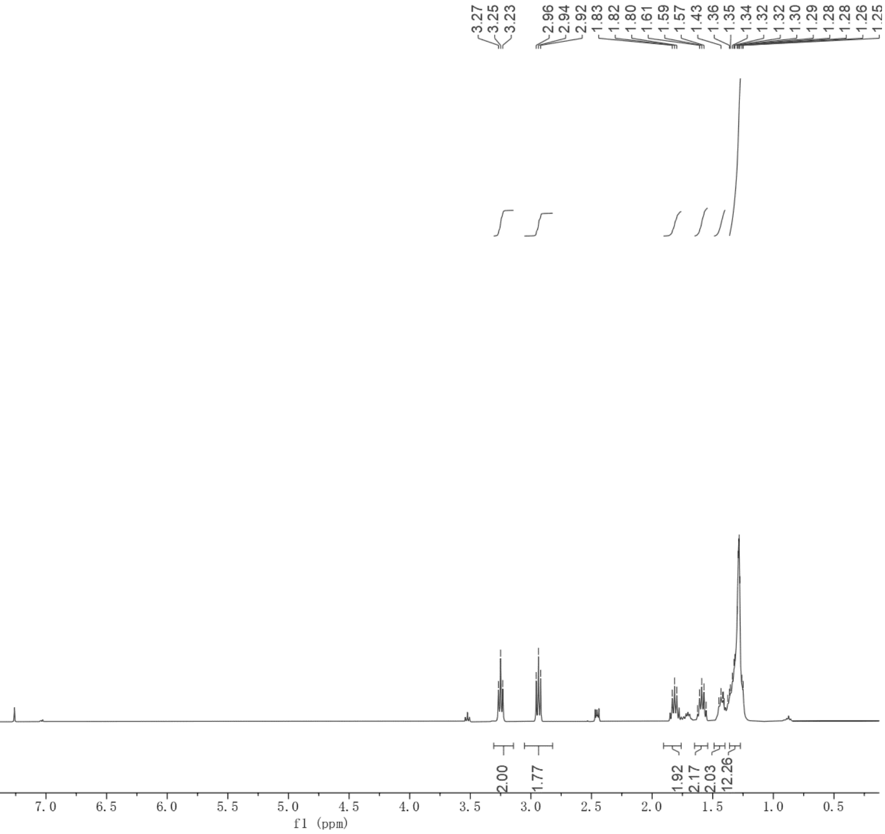

^{13}C NMR (100 MHz, $CDCl_3$) of **65**

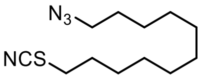

65

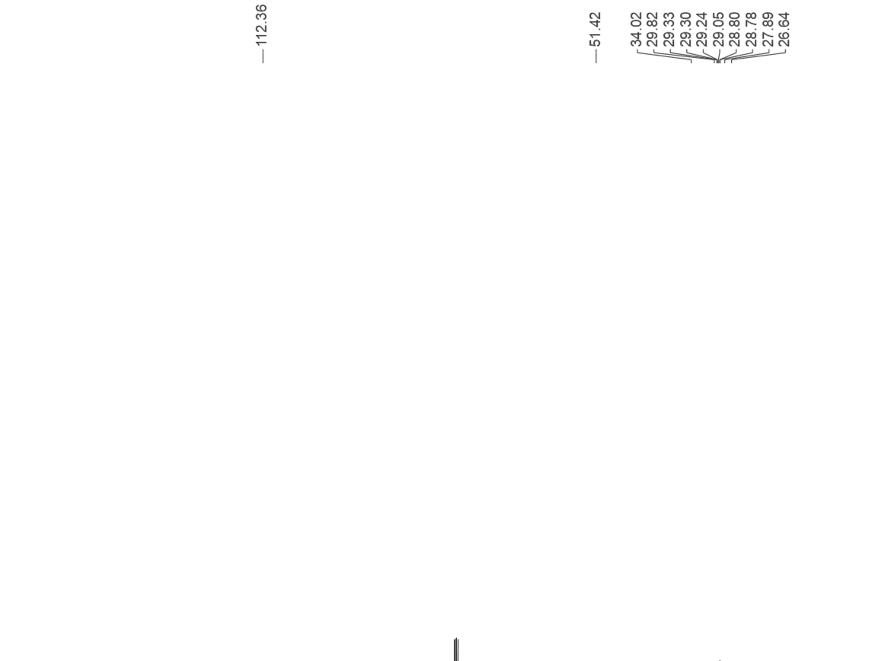

^{1}H NMR (400 MHz, $CDCl_3$) of **66**

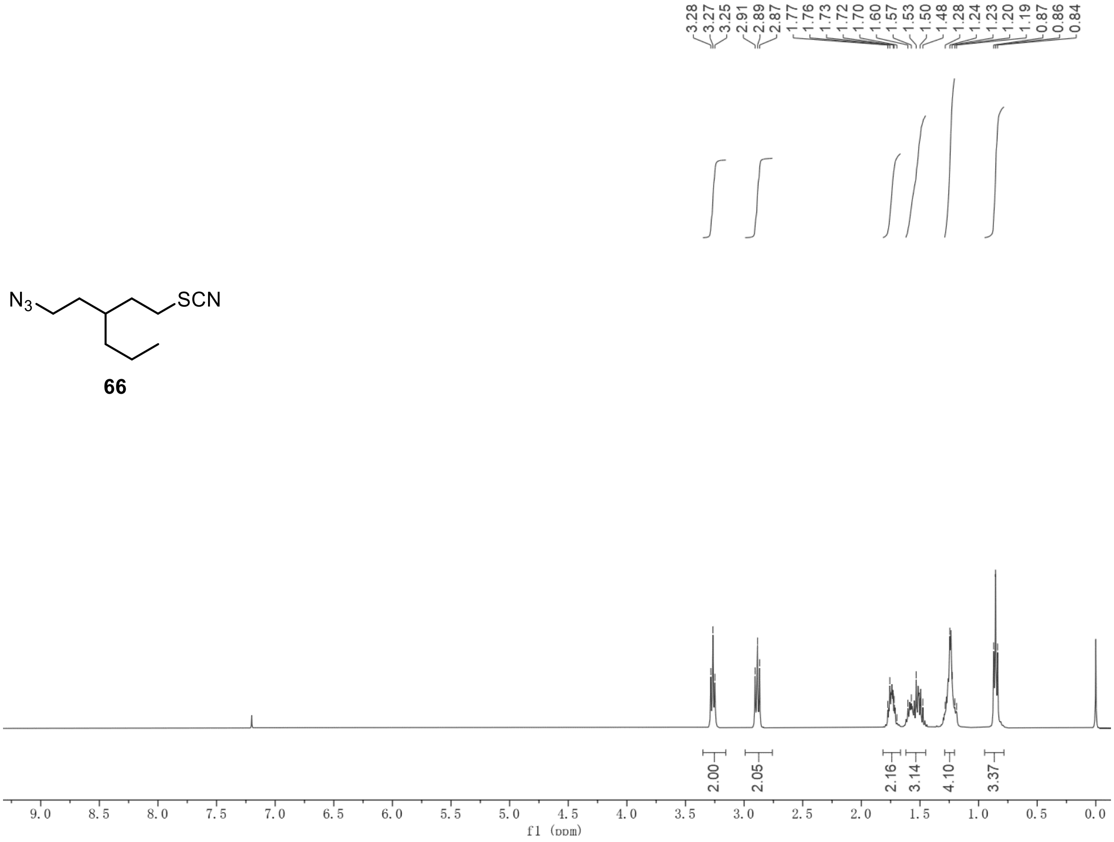

^{13}C NMR (100 MHz, $CDCl_3$) of **66**

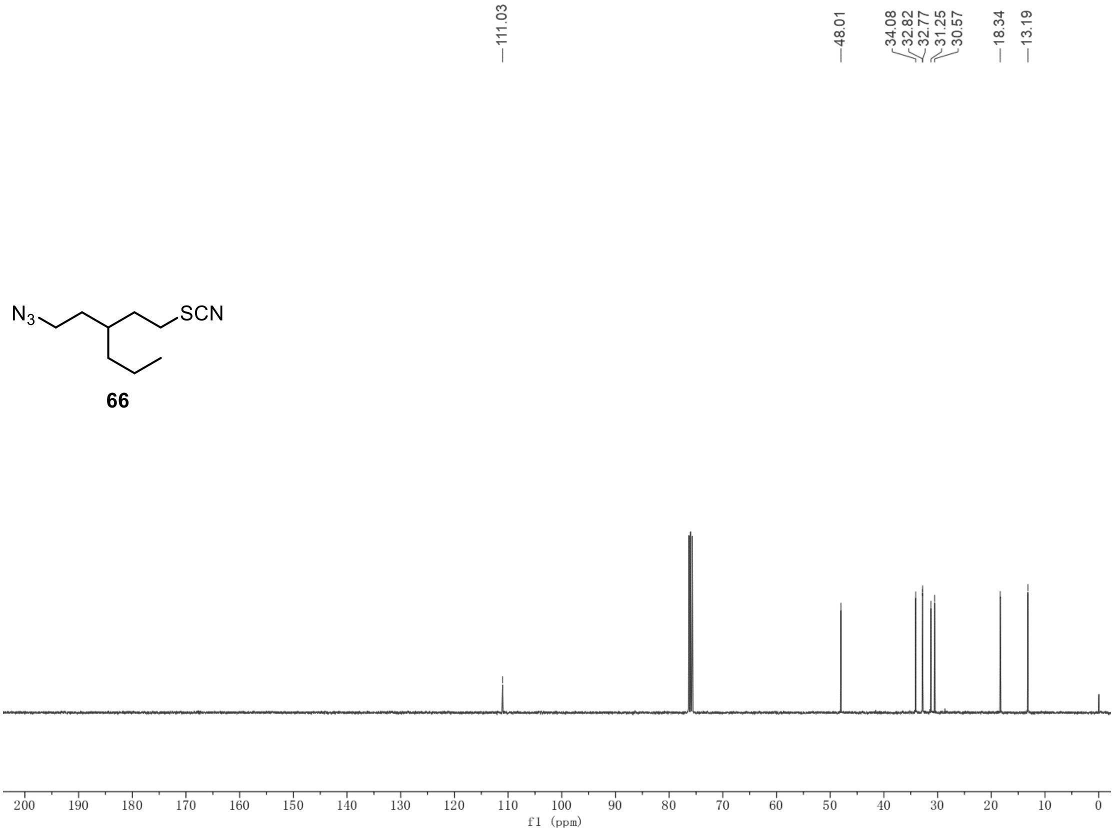

^{1}H NMR (400 MHz, $CDCl_3$) of **67**

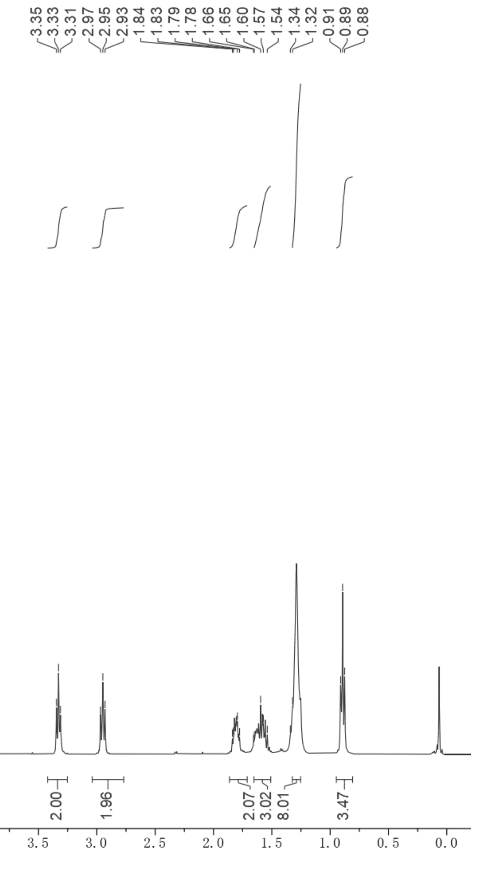

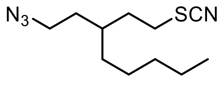

67

^{13}C NMR (100 MHz, $CDCl_3$) of **67**

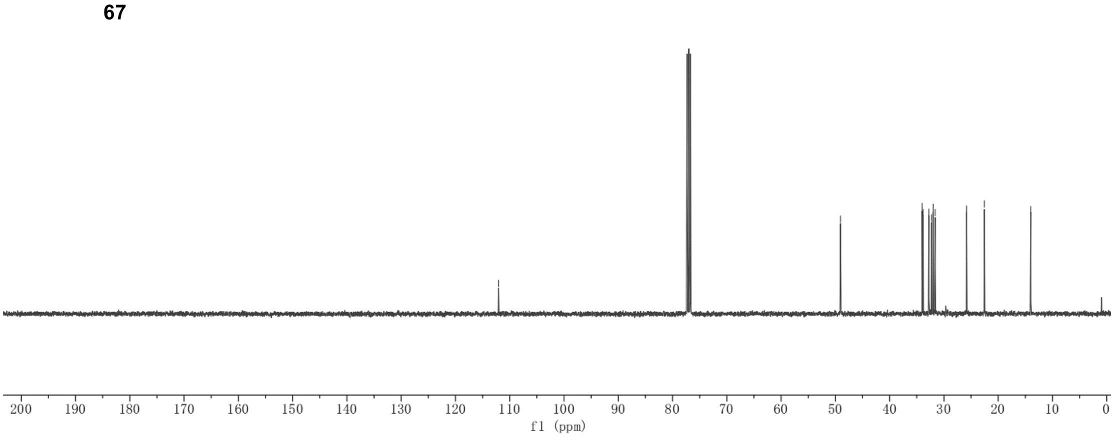

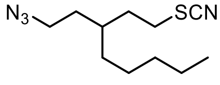

67

^{1}H NMR (400 MHz, $CDCl_3$) of **68**

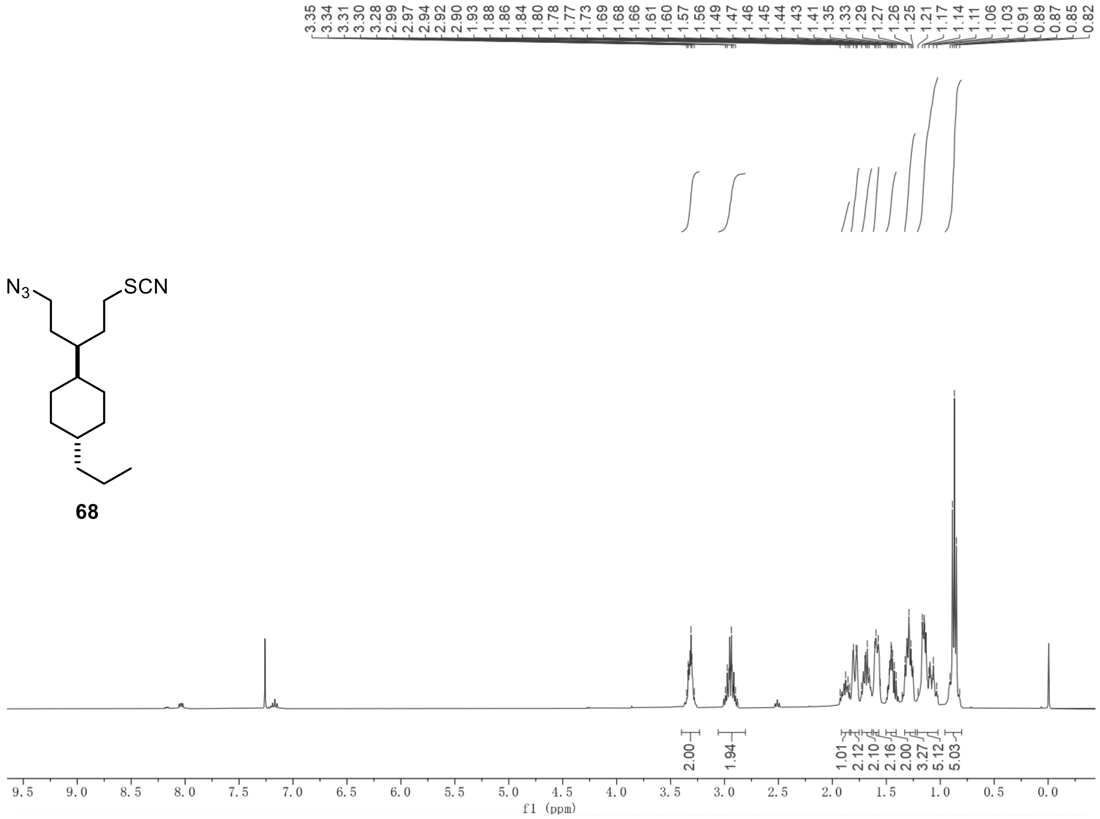

^{13}C NMR (100 MHz, $CDCl_3$) of **68**

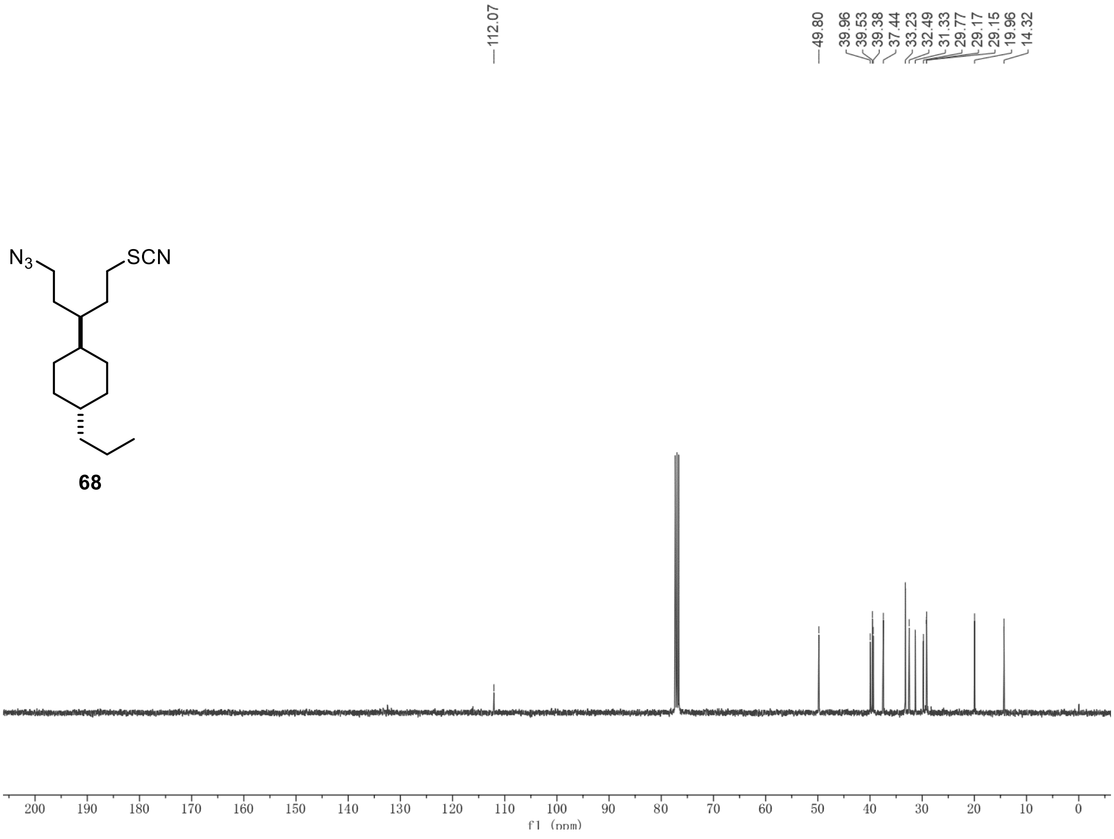

^{1}H NMR (400 MHz, $CDCl_3$) of **69**

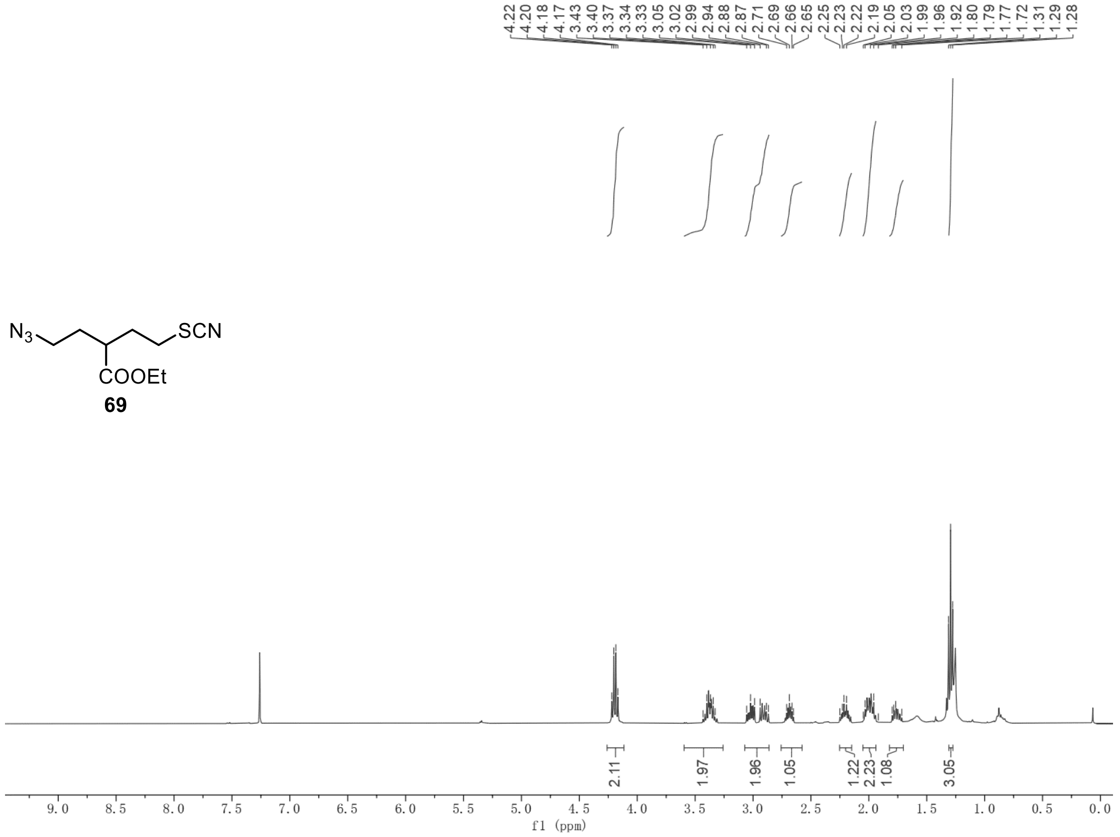

69

^{13}C NMR (100 MHz, $CDCl_3$) of **69**

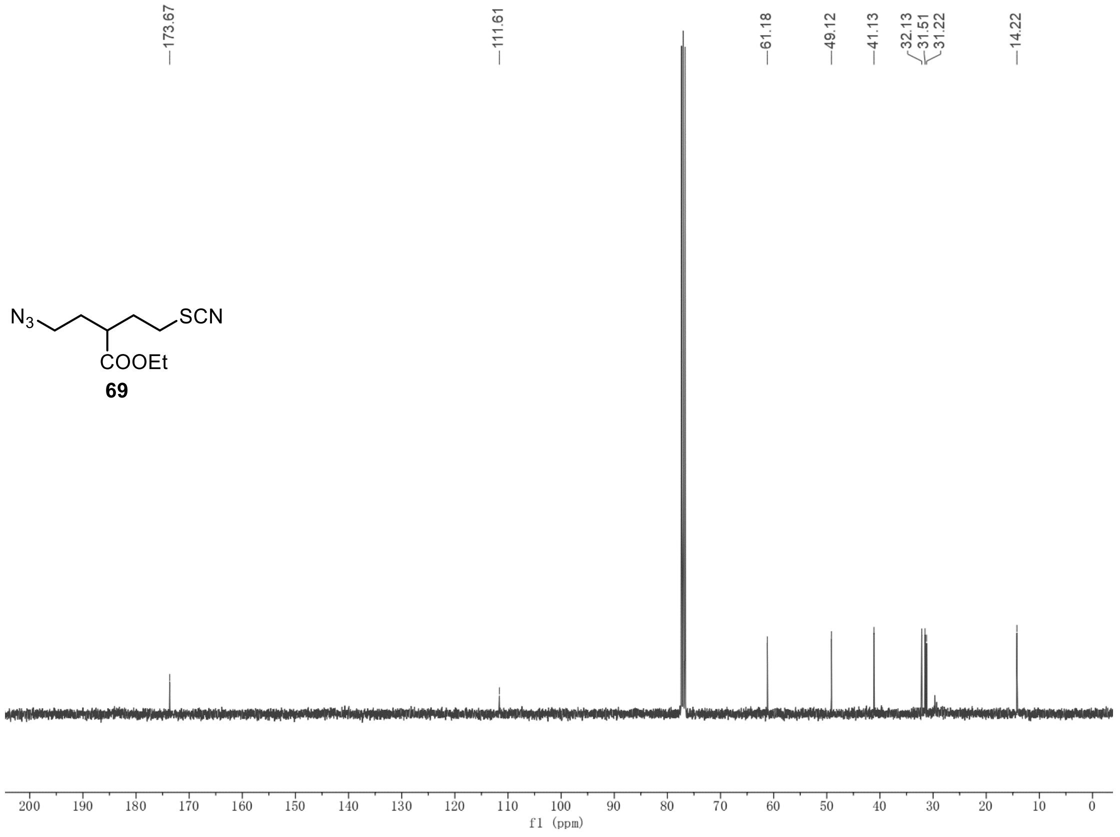

69

^{1}H NMR (400 MHz, $CDCl_3$) of **70**

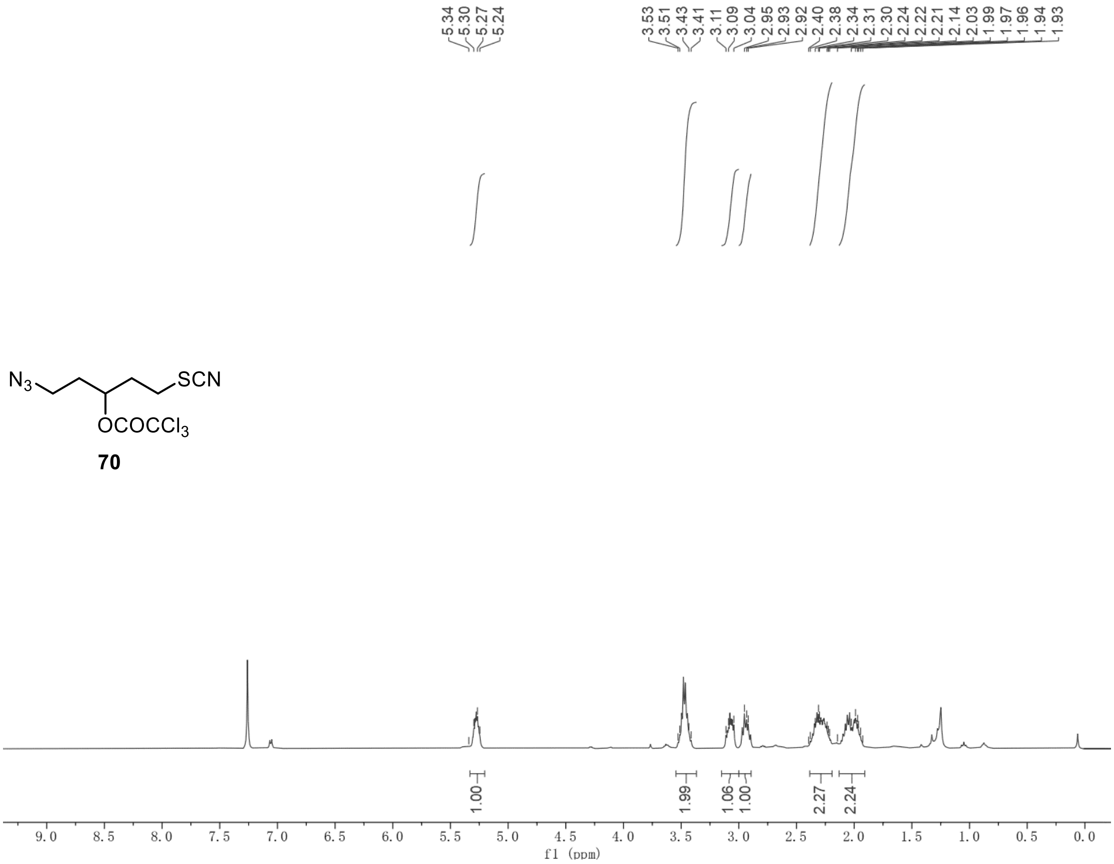

^{13}C NMR (100 MHz, $CDCl_3$) of **70**

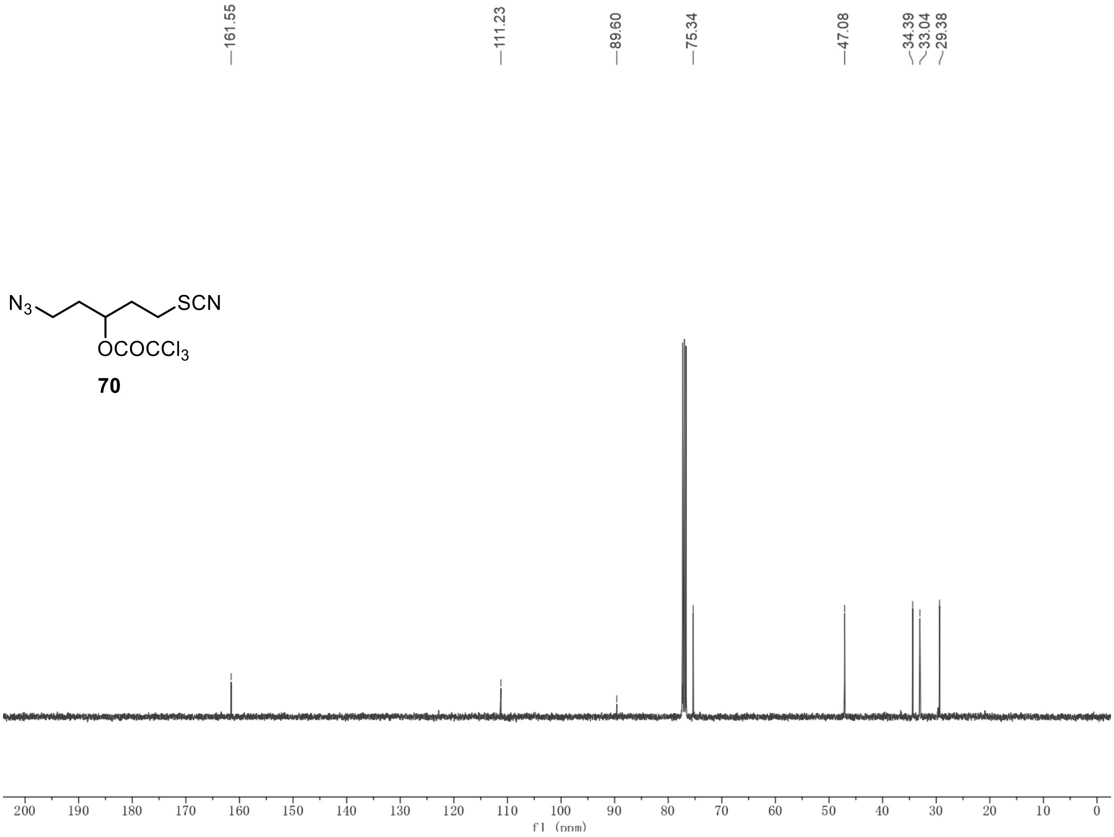

^{1}H NMR (400 MHz, $CDCl_3$) of **71**

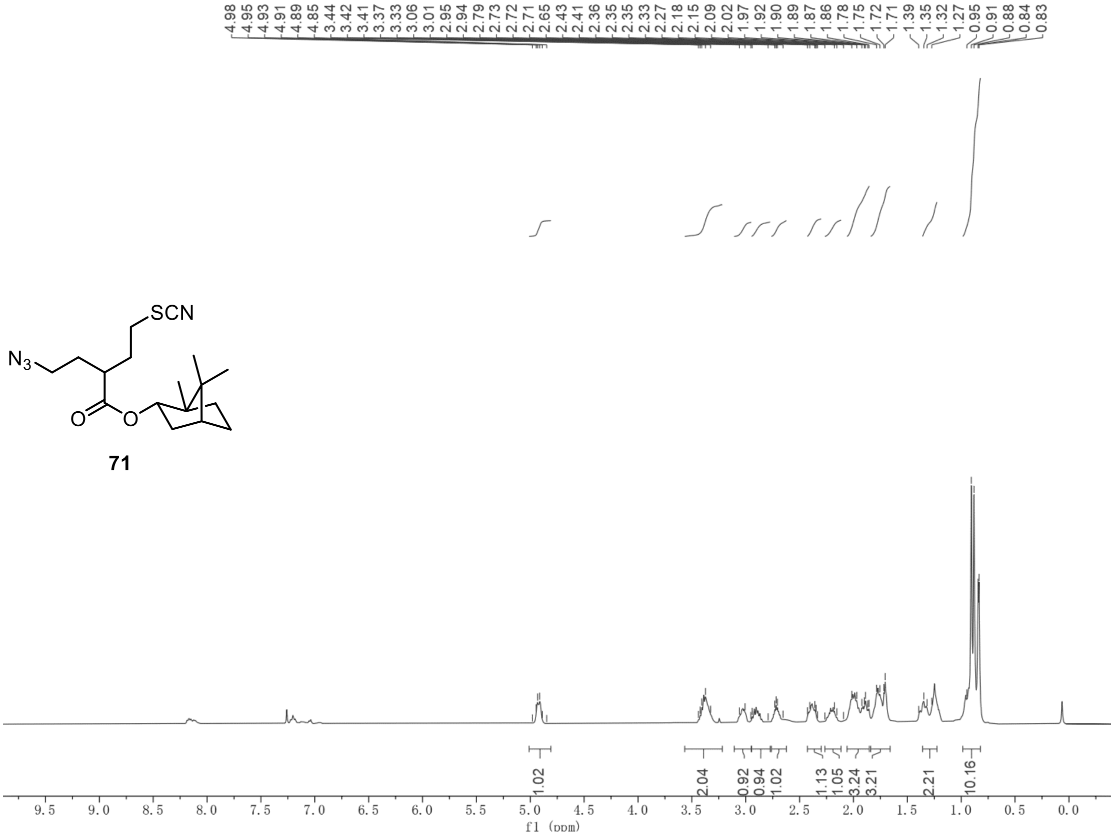

71

^{13}C NMR (100 MHz, $CDCl_3$) of **71**

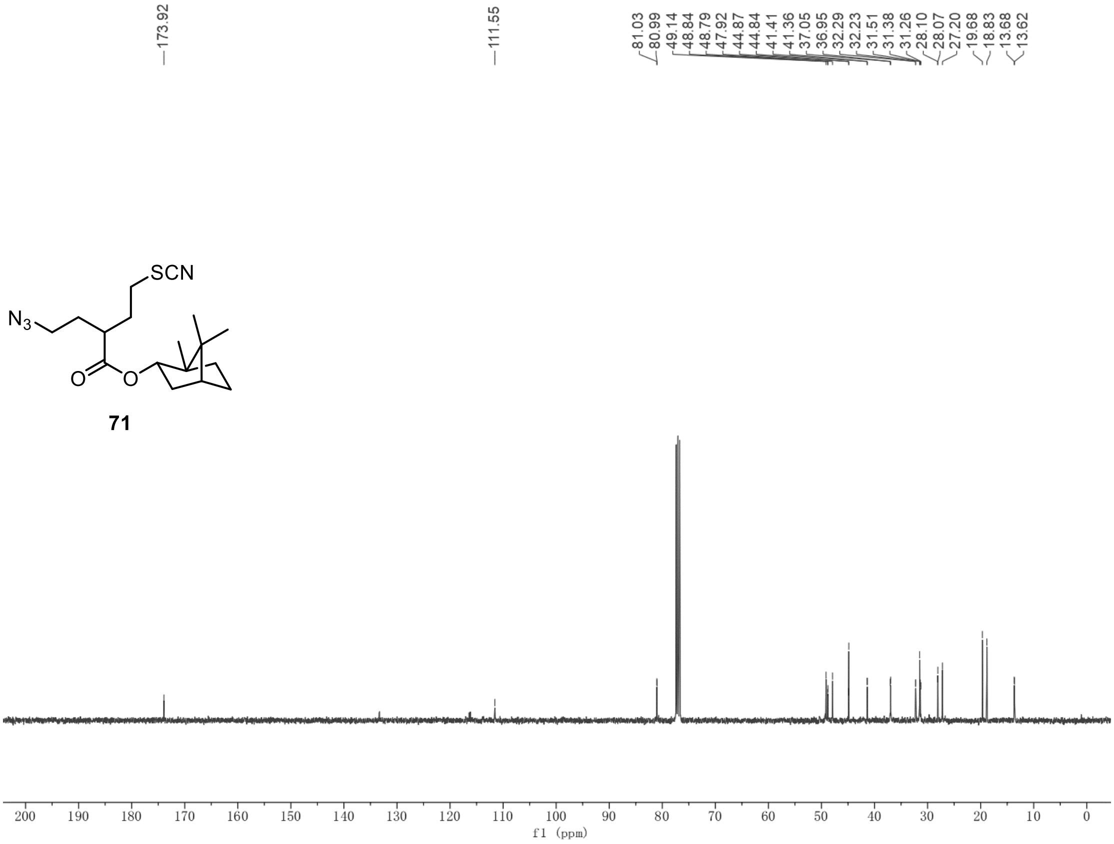

71

^{1}H NMR (400 MHz, $CDCl_3$) of **72**

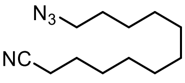

72

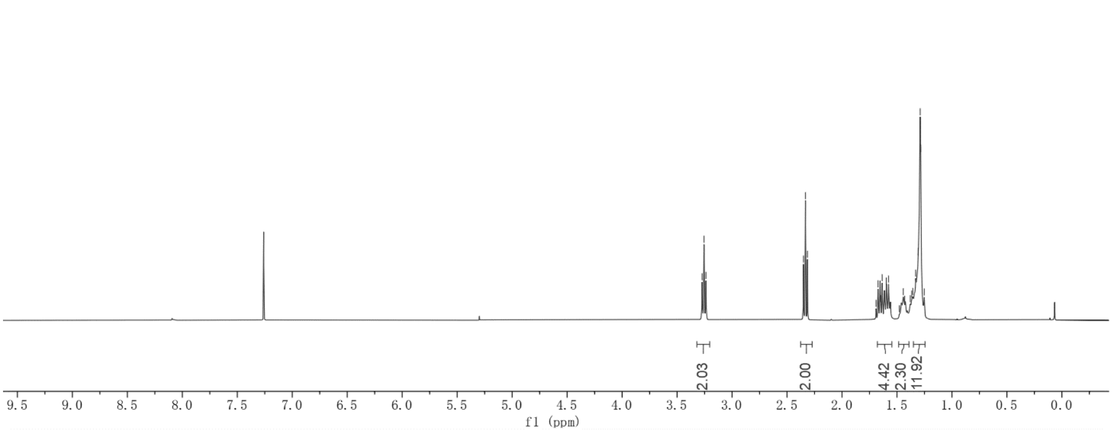

^{13}C NMR (100 MHz, $CDCl_3$) of **72**

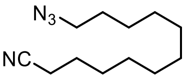

72

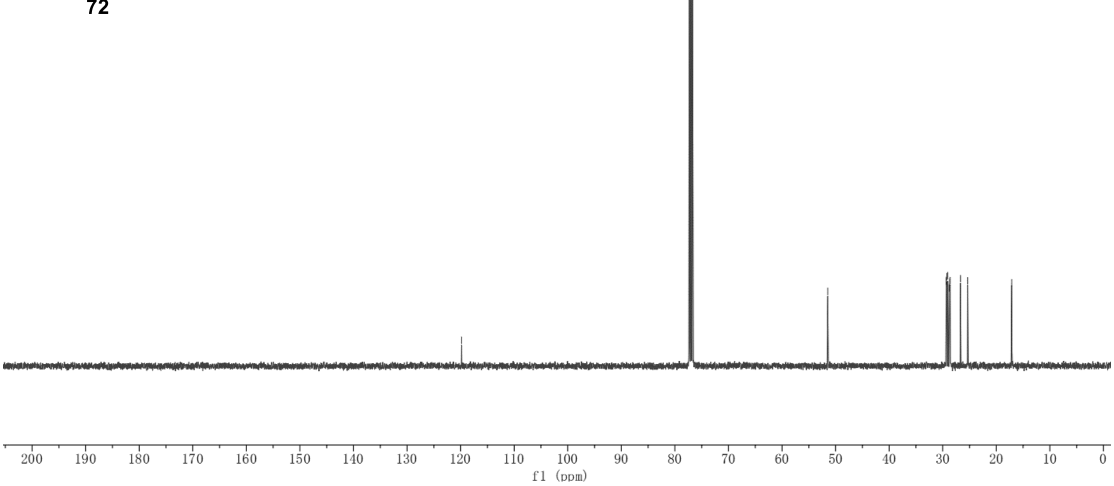

^{1}H NMR (400 MHz, $CDCl_3$) of **73**

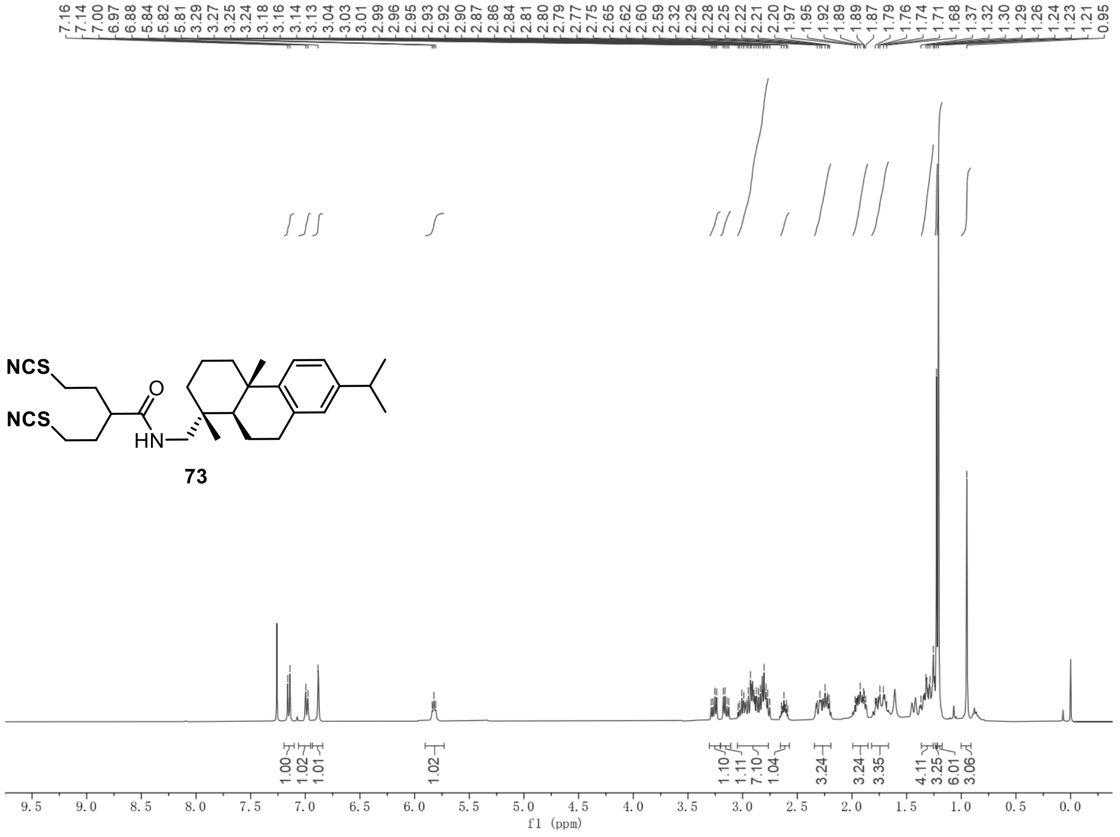

^{13}C NMR (100 MHz, $CDCl_3$) of **73**

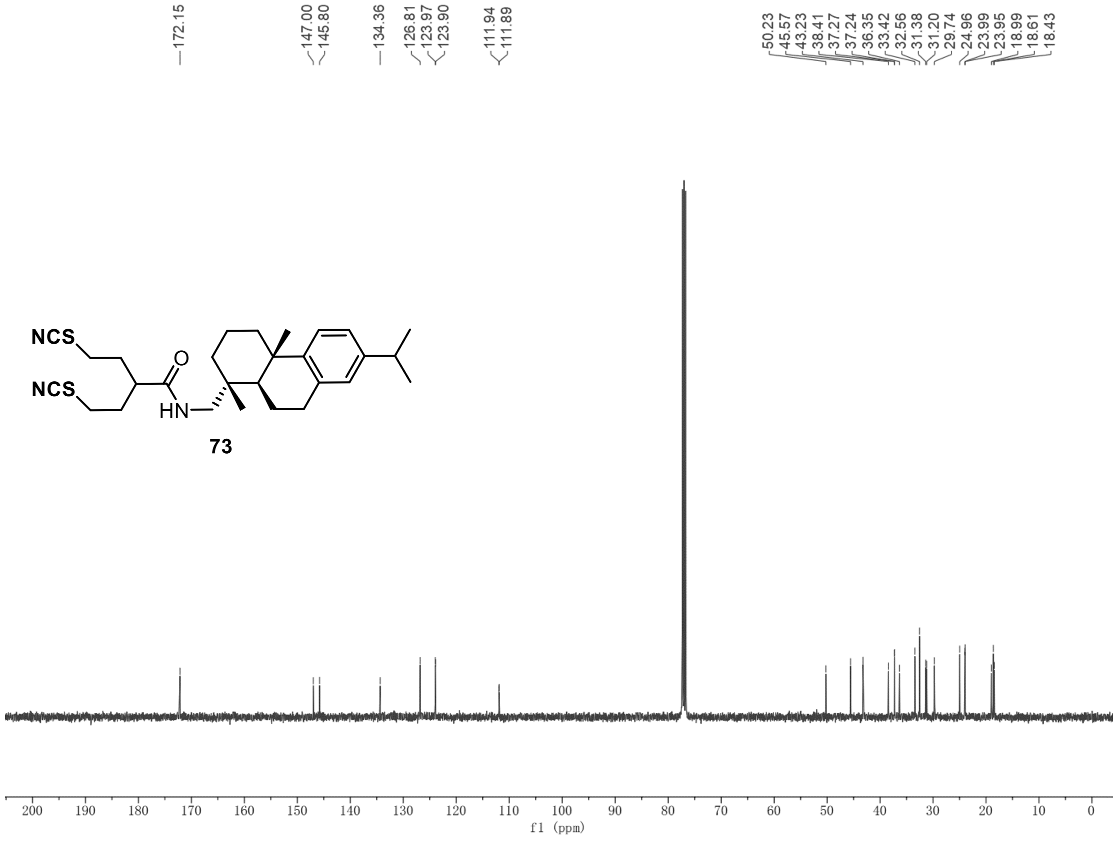

^{1}H NMR (400 MHz, $CDCl_3$) of **74**

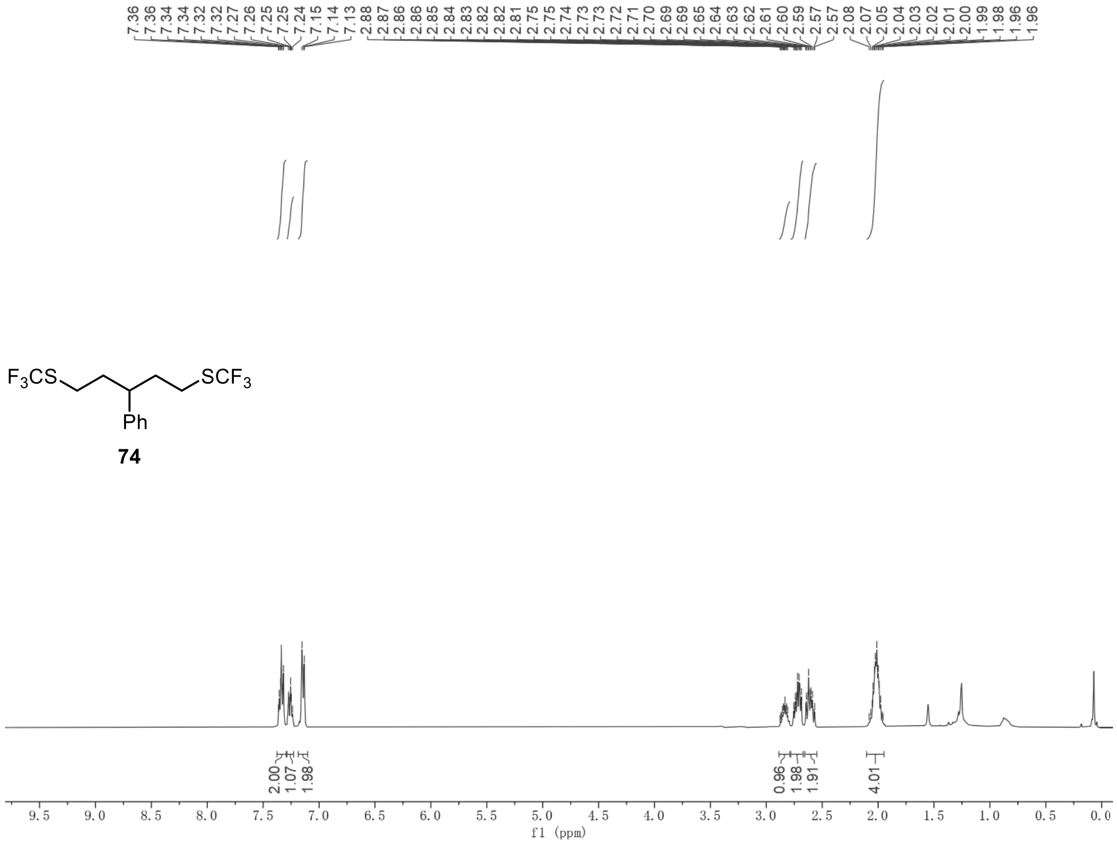

^{19}F NMR (376 MHz, $CDCl_3$) of **74**

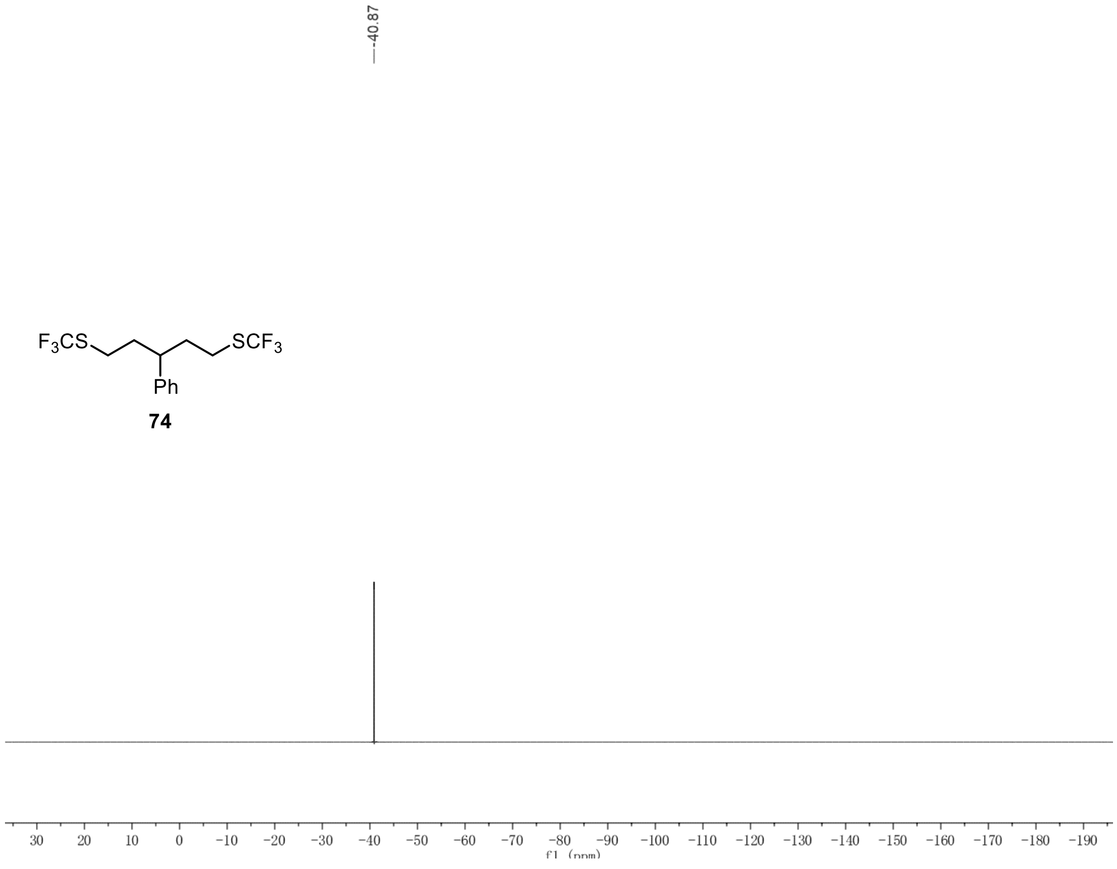

^{13}C NMR (100 MHz, $CDCl_3$) of **74**

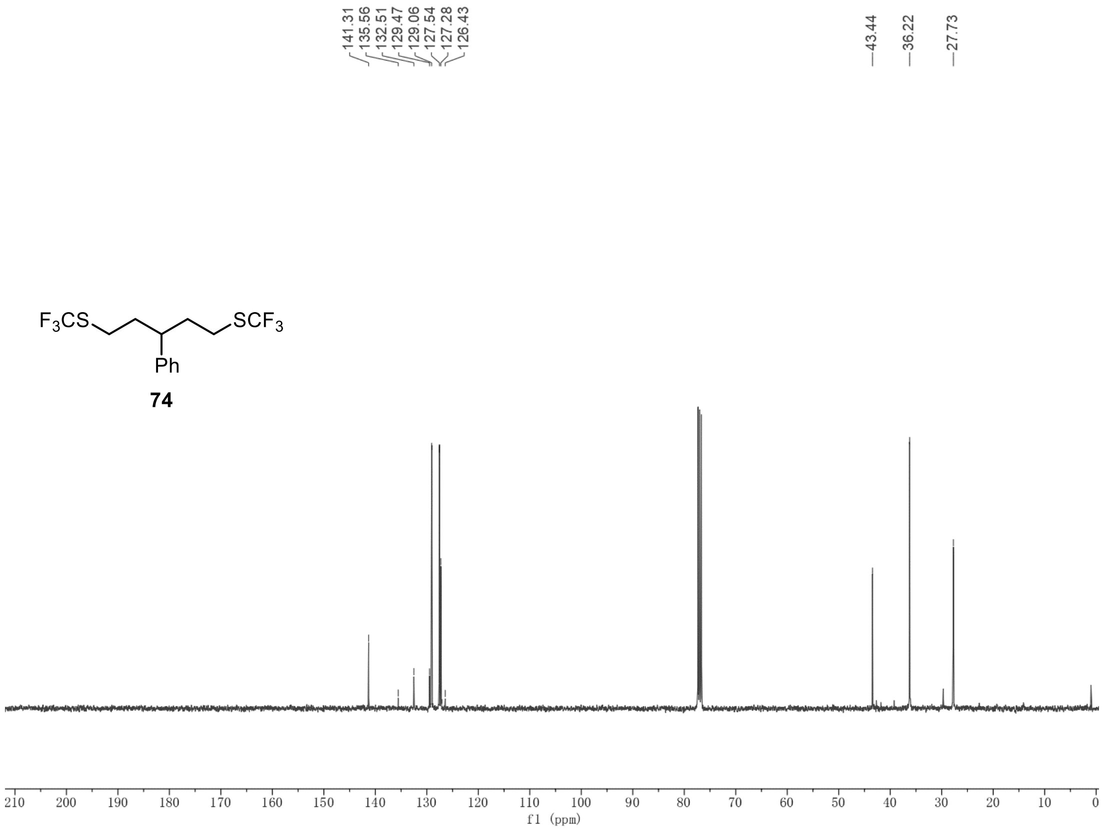

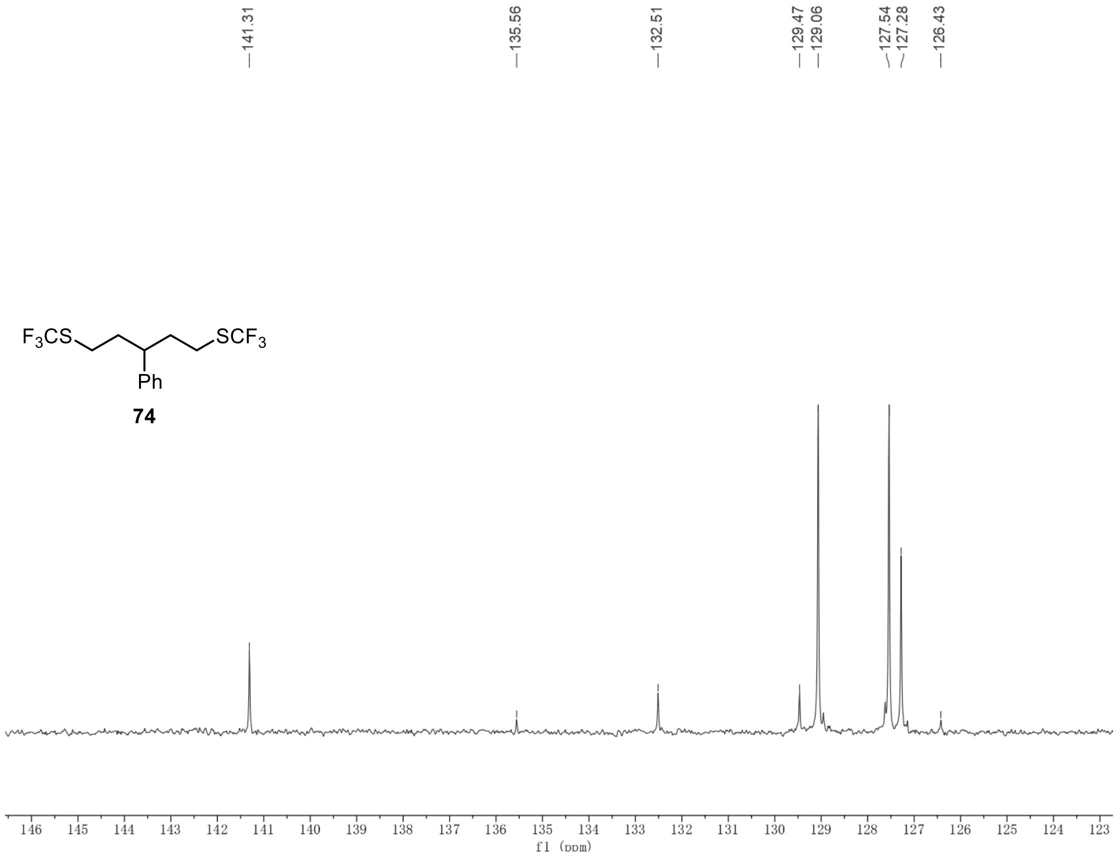

^{1}H NMR (400 MHz, $CDCl_3$) of **75**

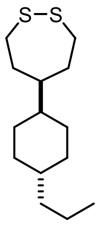

75

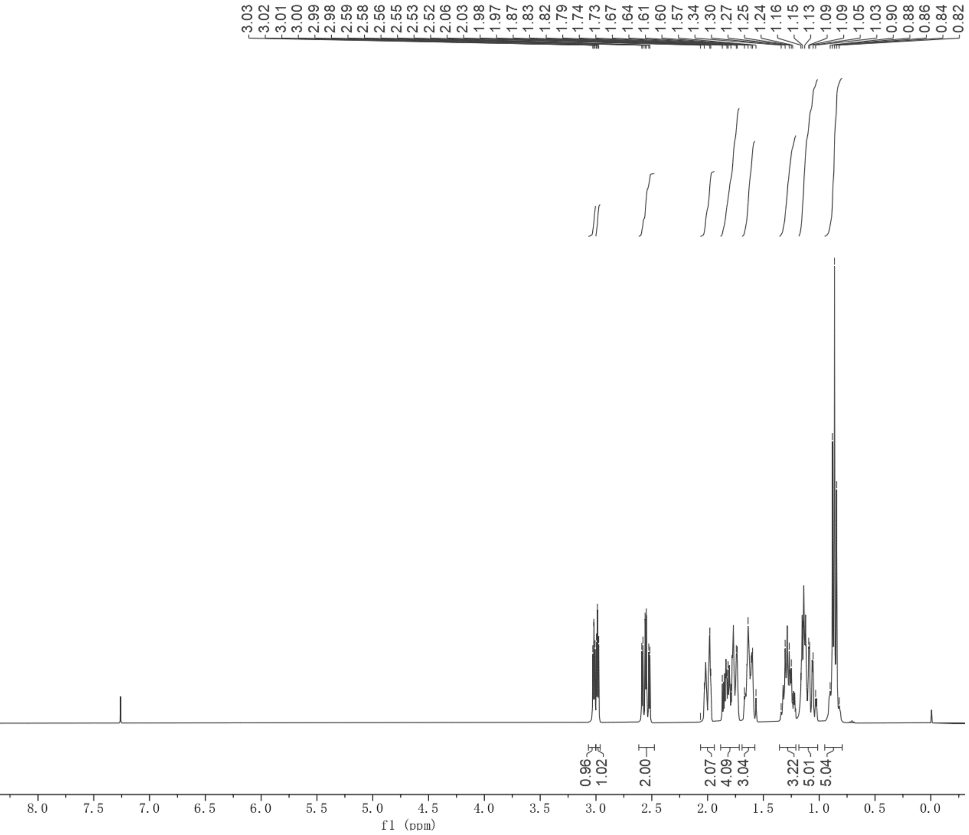

^{13}C NMR (100 MHz, $CDCl_3$) of **75**

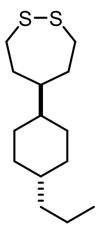

75

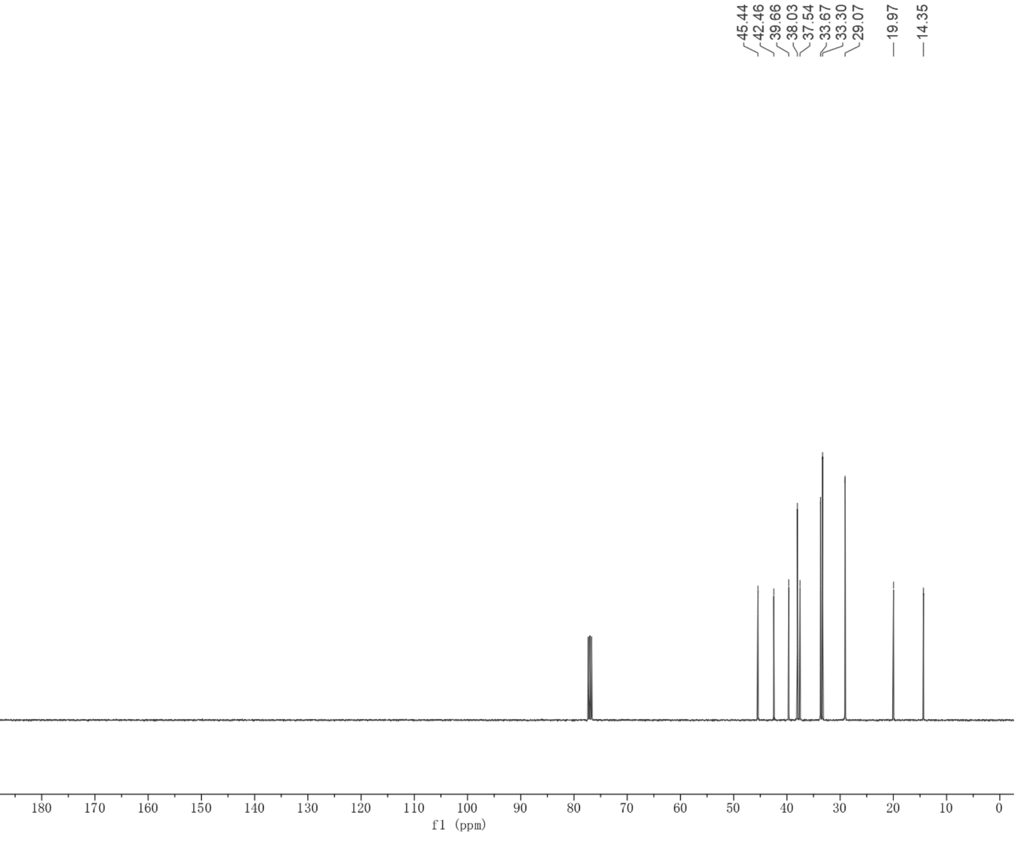

^{1}H NMR (400 MHz, $CDCl_3$) of **76**

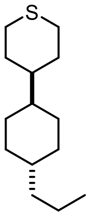

76

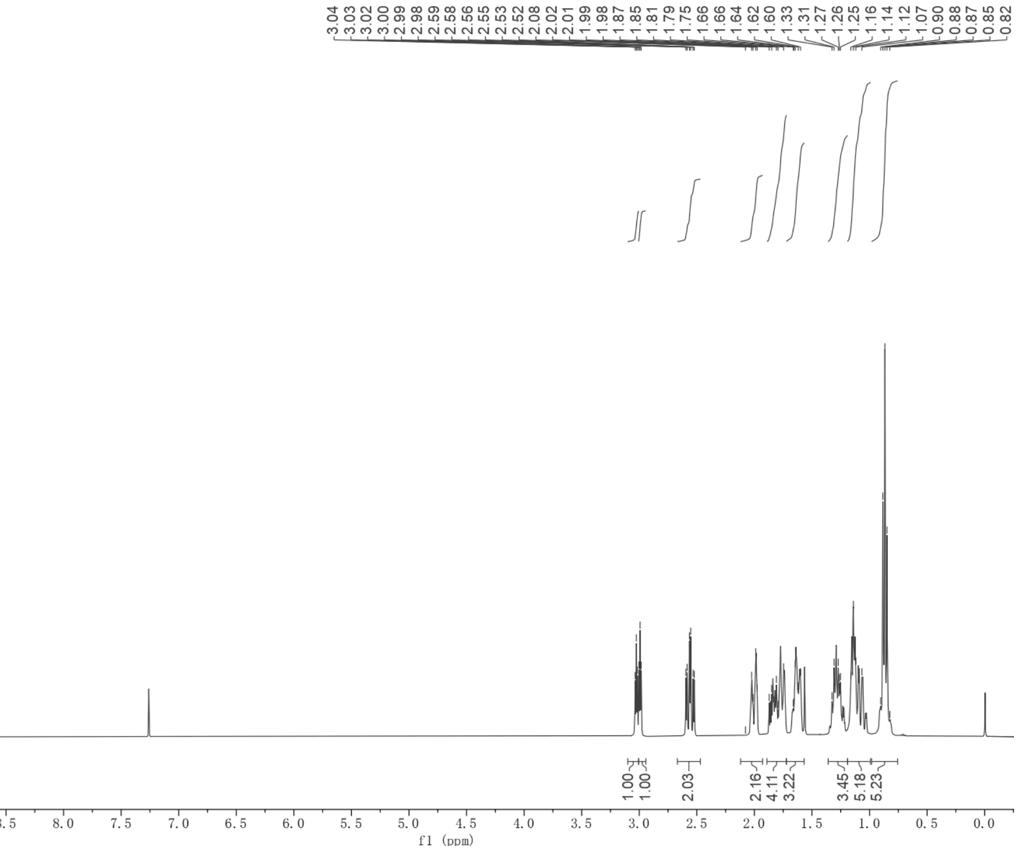

^{13}C NMR (100 MHz, $CDCl_3$) of **76**

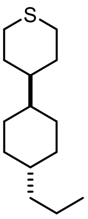

76

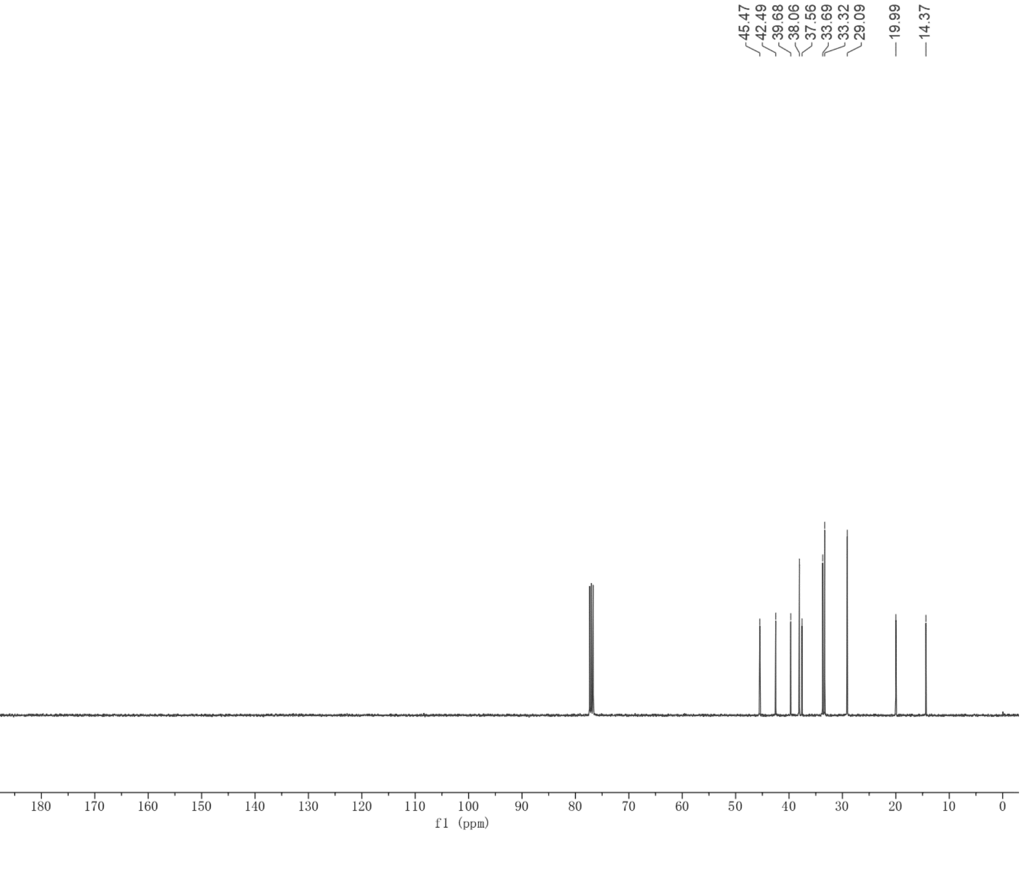

^{1}H NMR (400 MHz, $CDCl_3$) of **77**

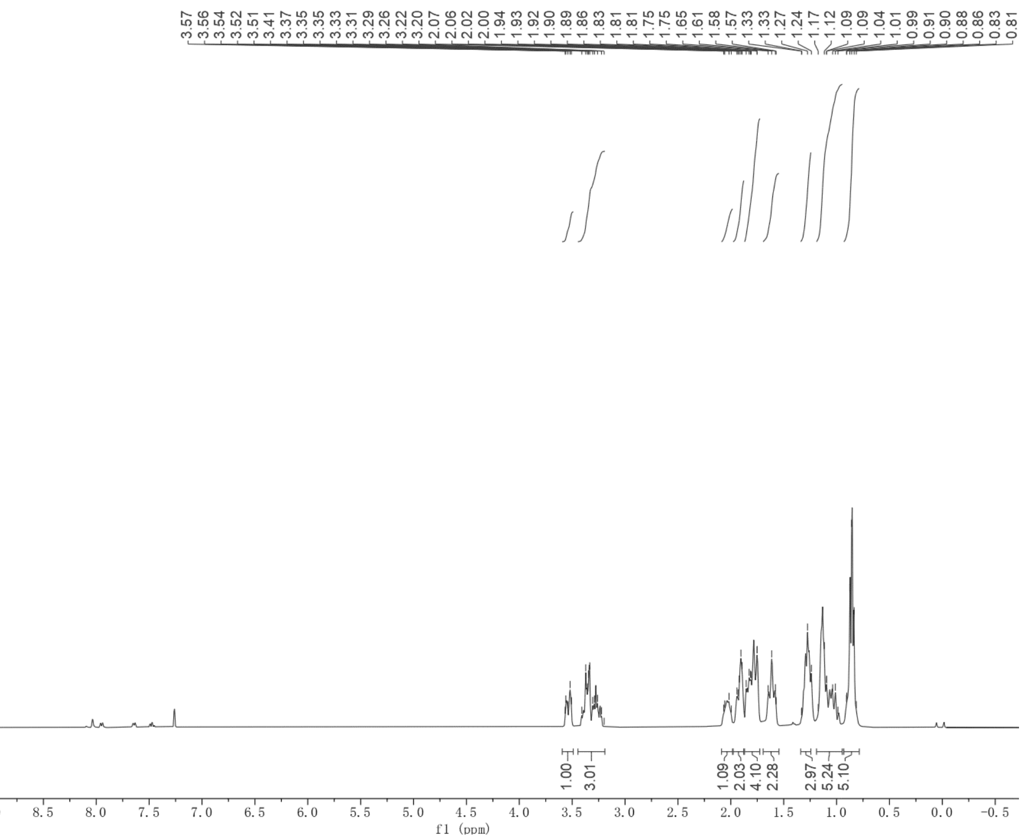

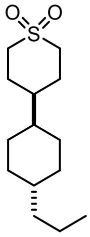

77

^{13}C NMR (100 MHz, $CDCl_3$) of **77**

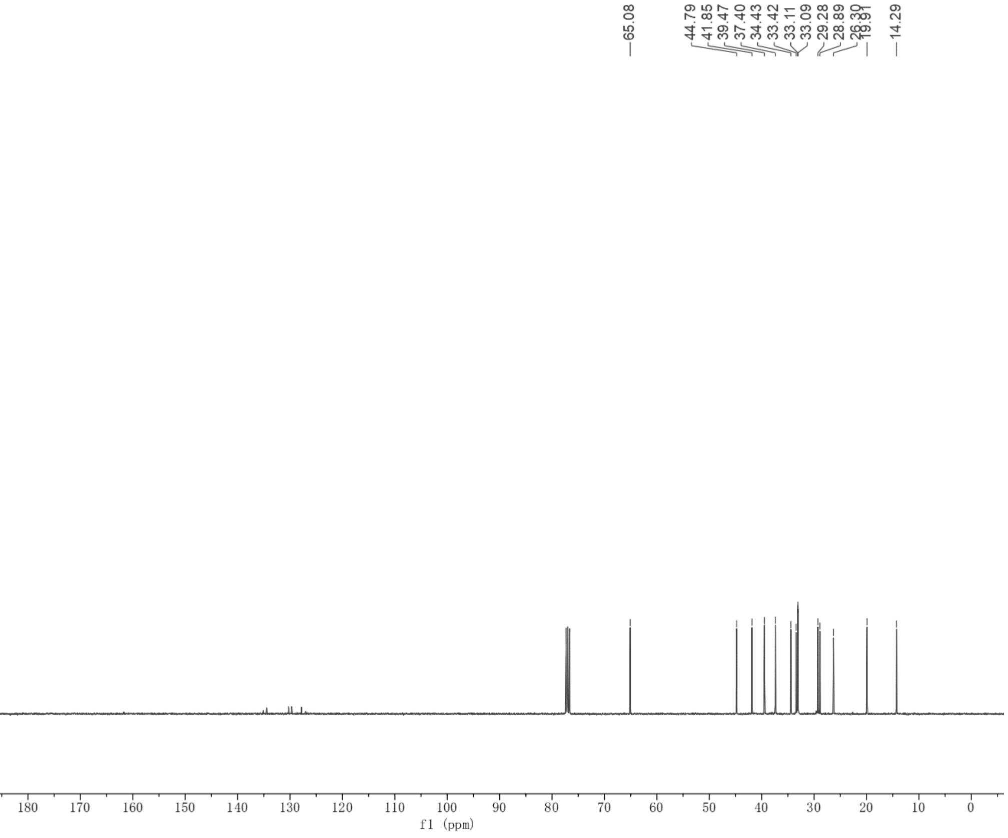

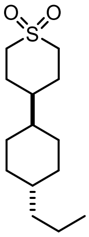

77

^{1}H NMR (400 MHz, CDCl$_3$) of **78**

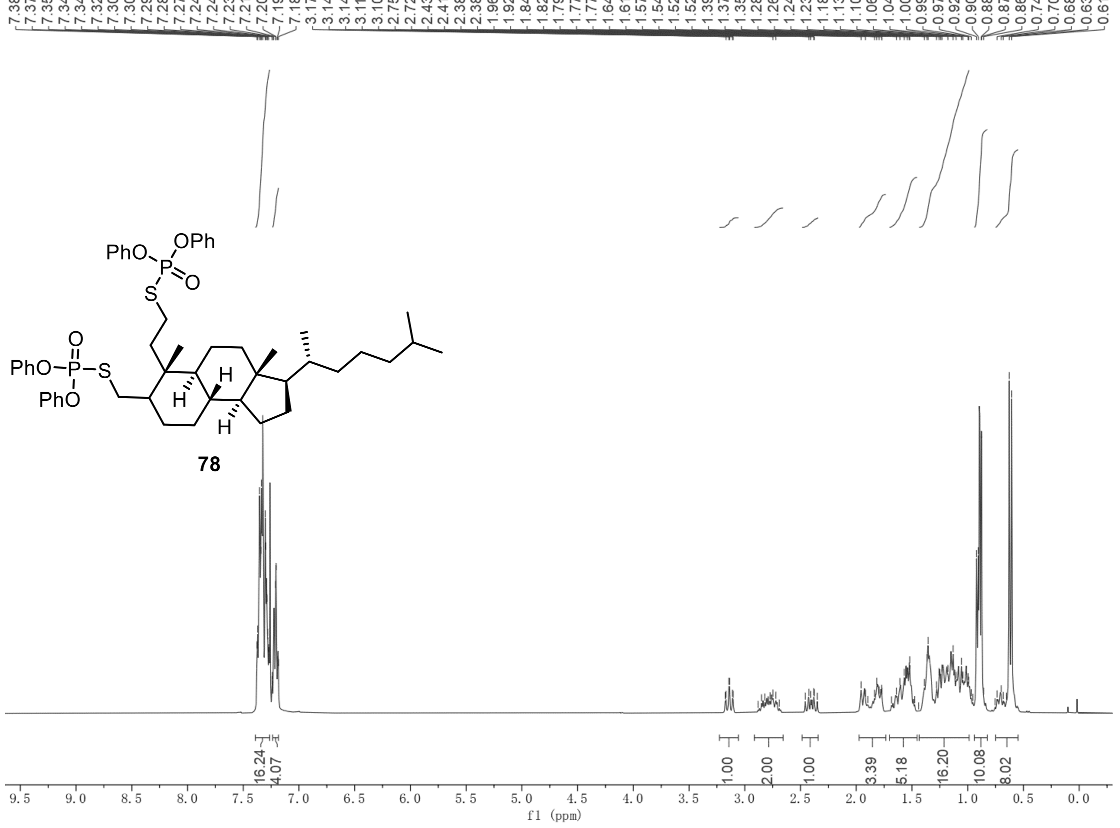

^{31}P NMR (161 MHz, CDCl$_3$) of **78**

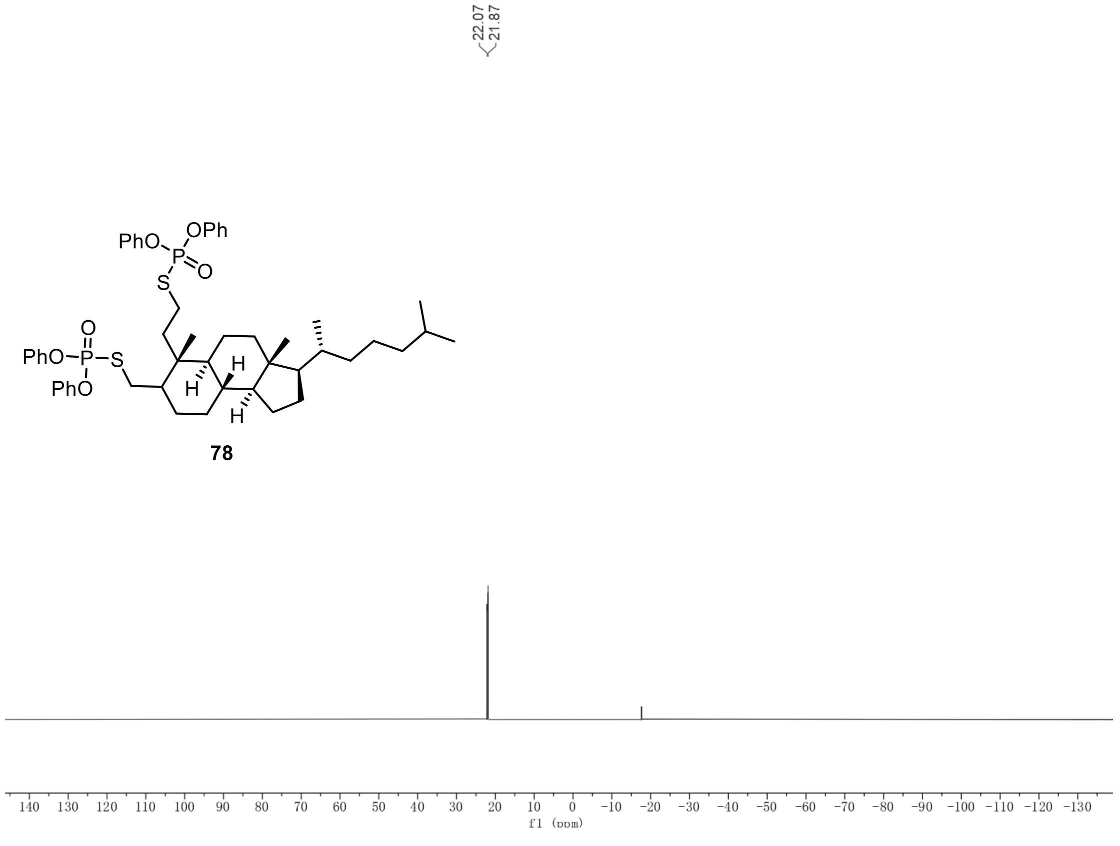

^{13}**C NMR** (100 MHz, $CDCl_3$) of **78**

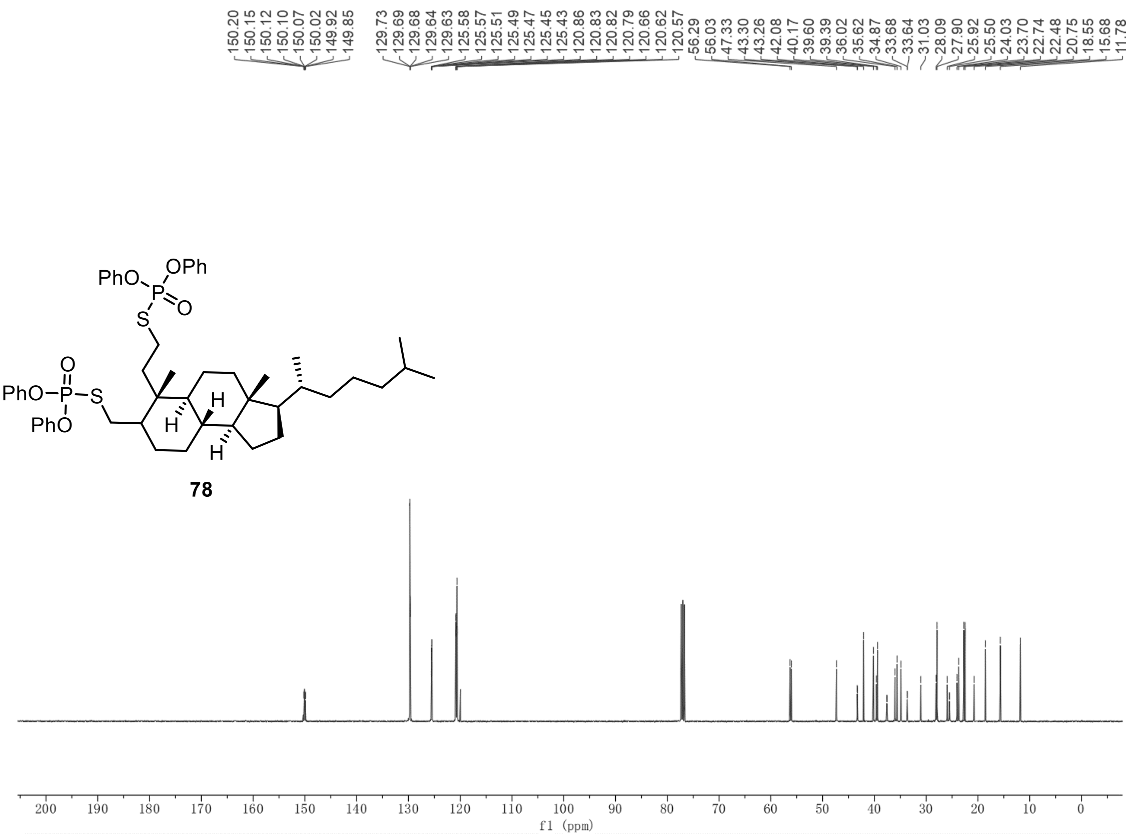

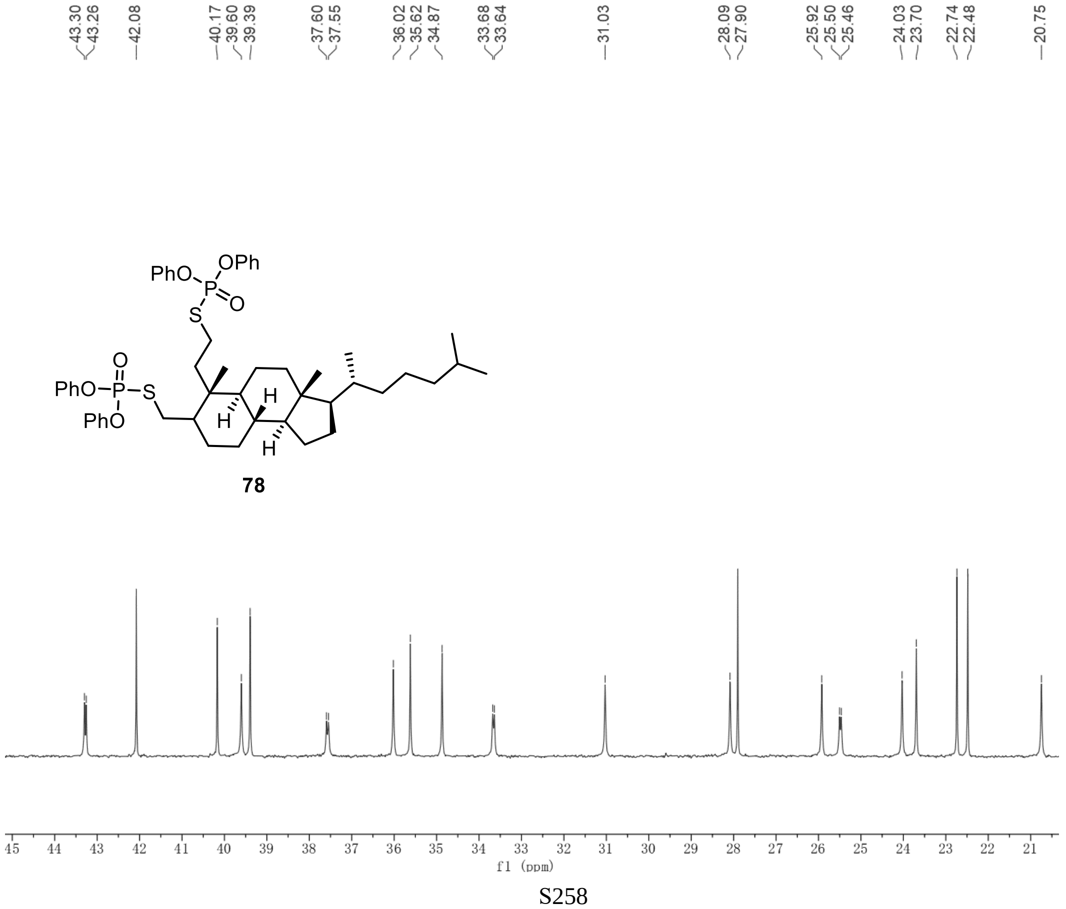

^{1}H NMR (400 MHz, $CDCl_3$) of **79**

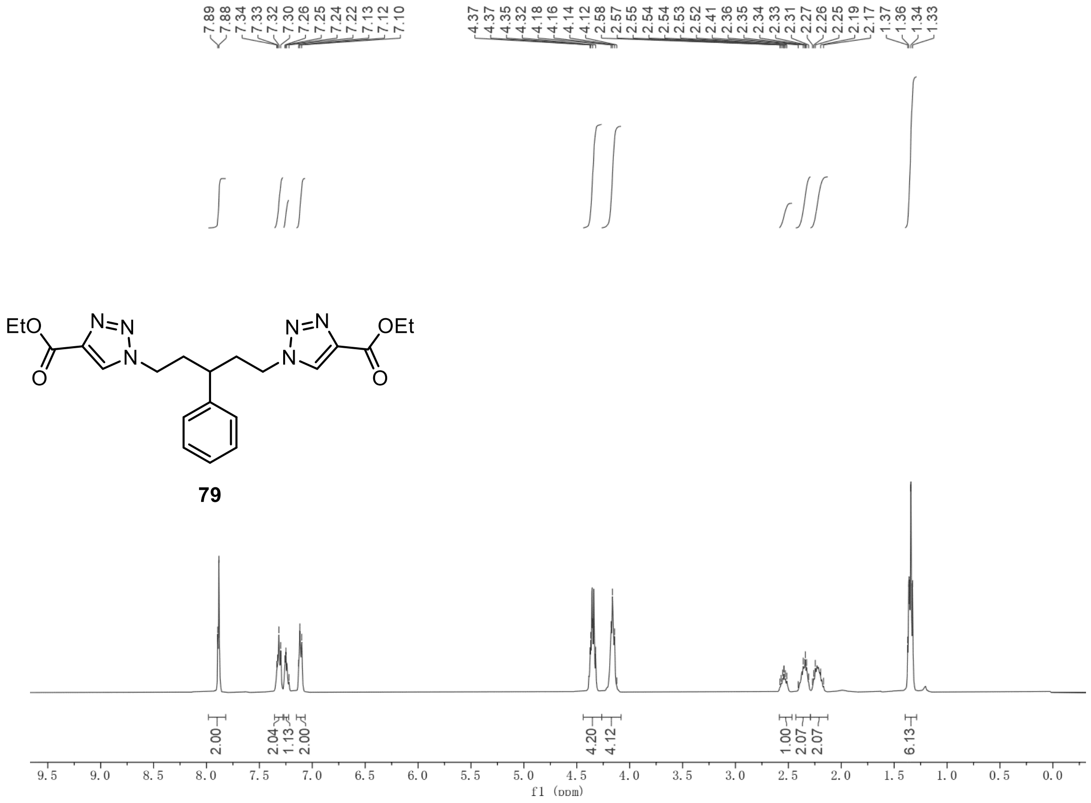

^{13}C NMR (100 MHz, $CDCl_3$) of **79**

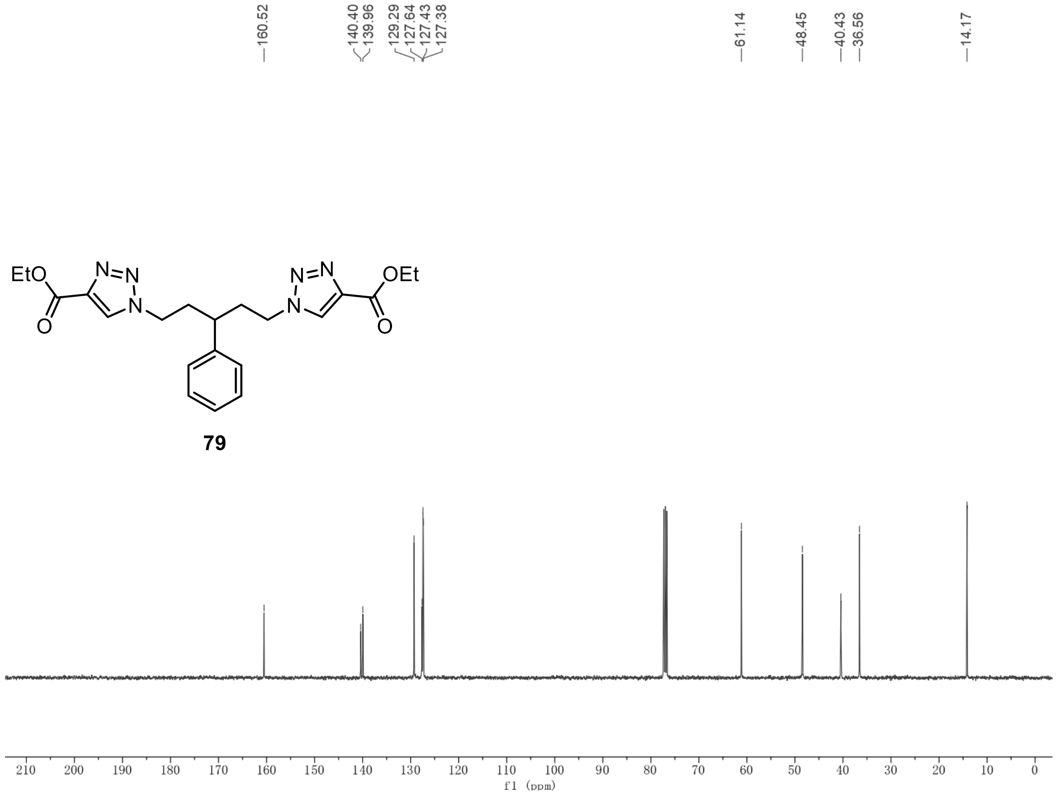

^{1}H NMR (400 MHz, $CDCl_3$) of **80**

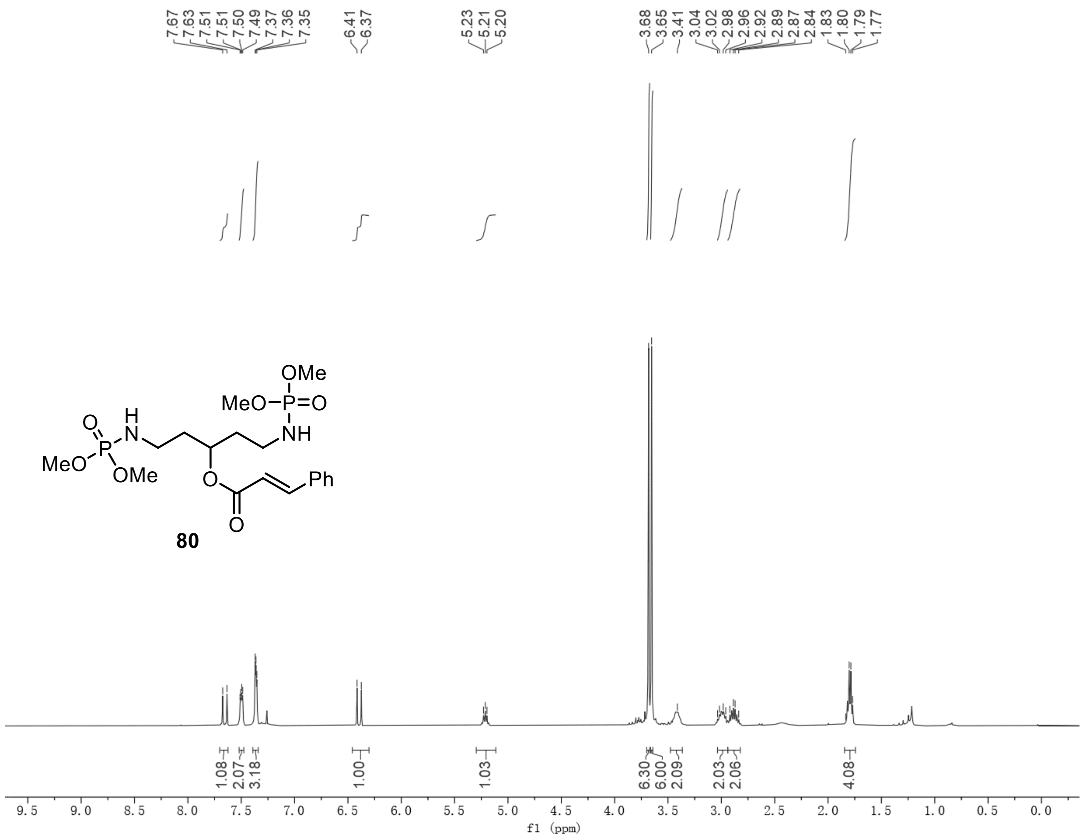

^{13}C NMR (100 MHz, $CDCl_3$) of **80**

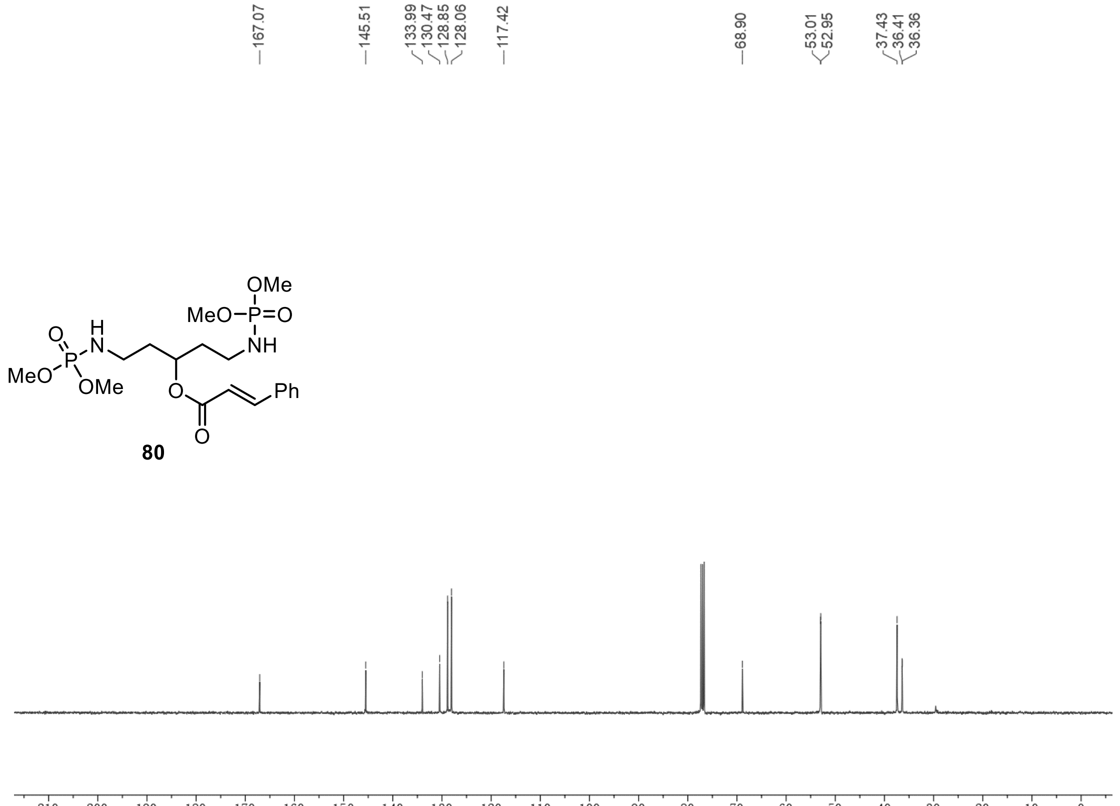

^{31}P NMR (161 MHz, CDCl$_3$) of **80**

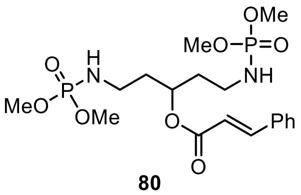

80

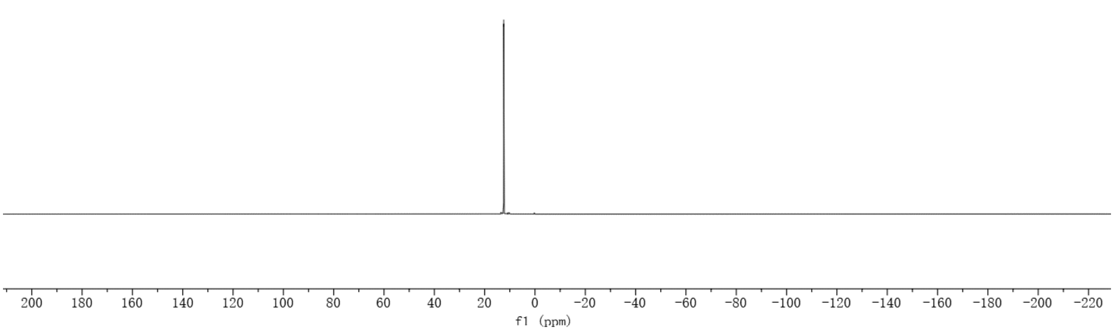

^{1}H NMR (400 MHz, $CDCl_3$)

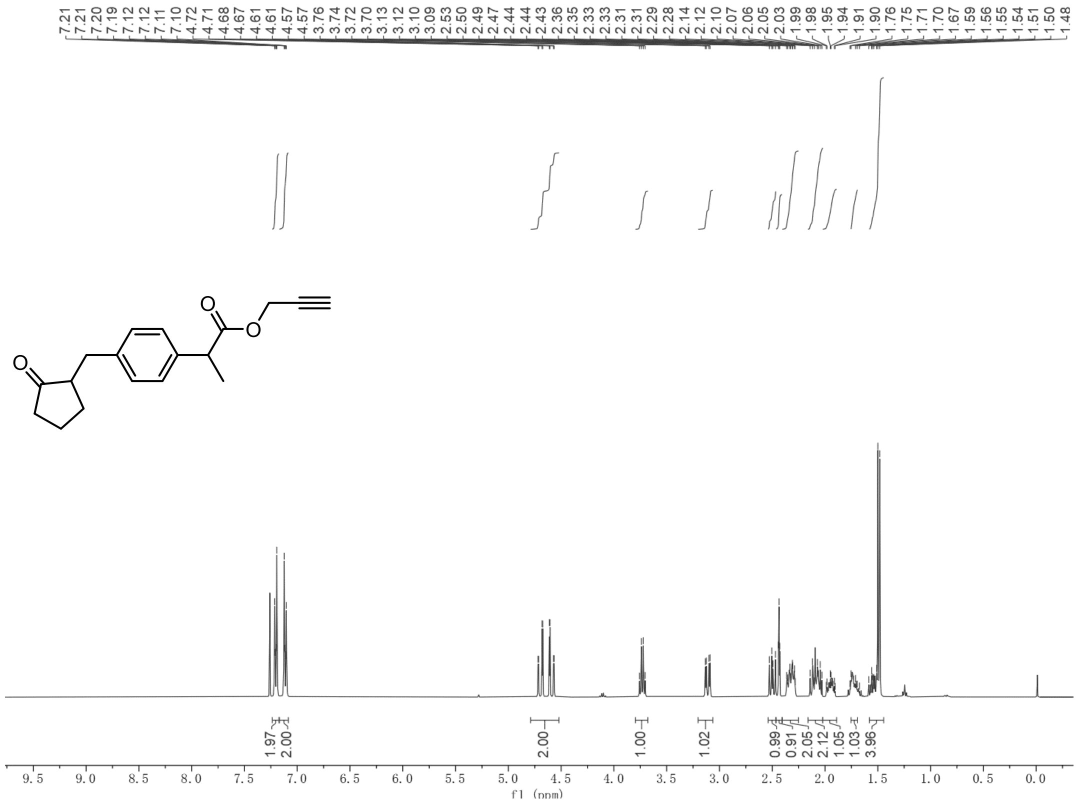

^{13}C NMR (100 MHz, $CDCl_3$)

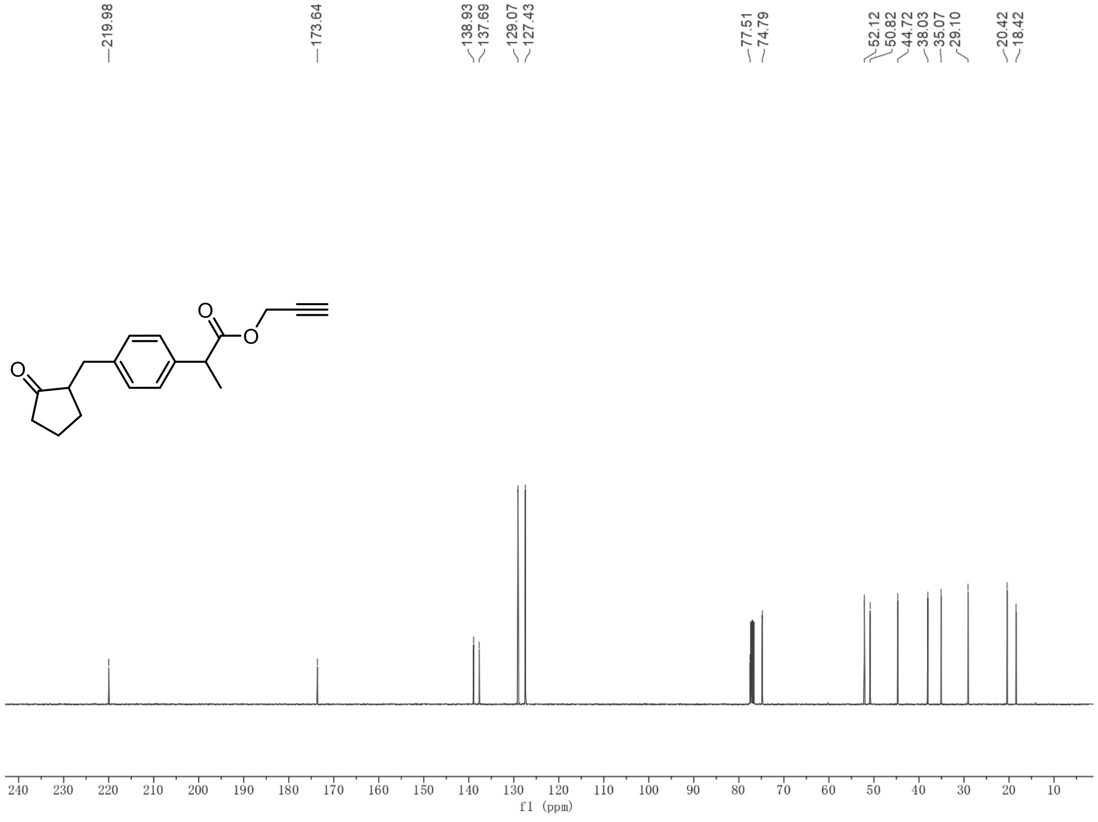

^{1}H NMR (400 MHz, $CDCl_3$) of **81**

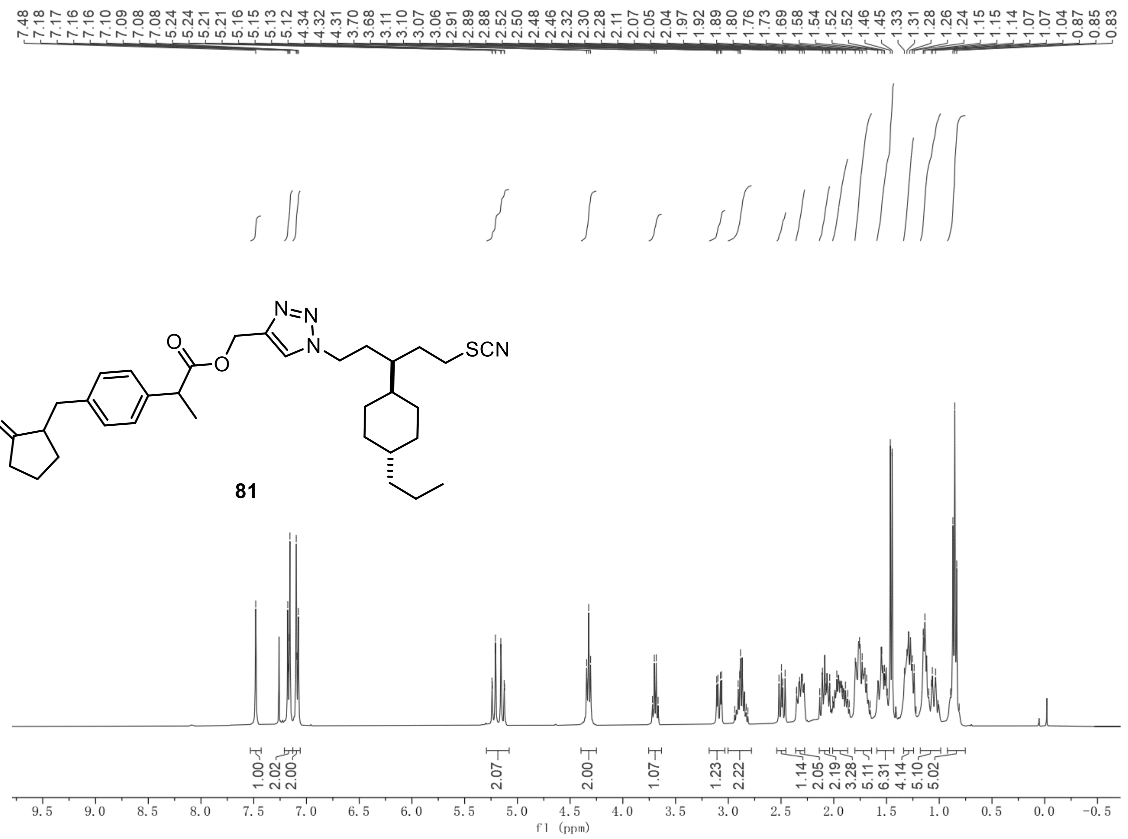

^{13}C NMR (100 MHz, $CDCl_3$) of **81**

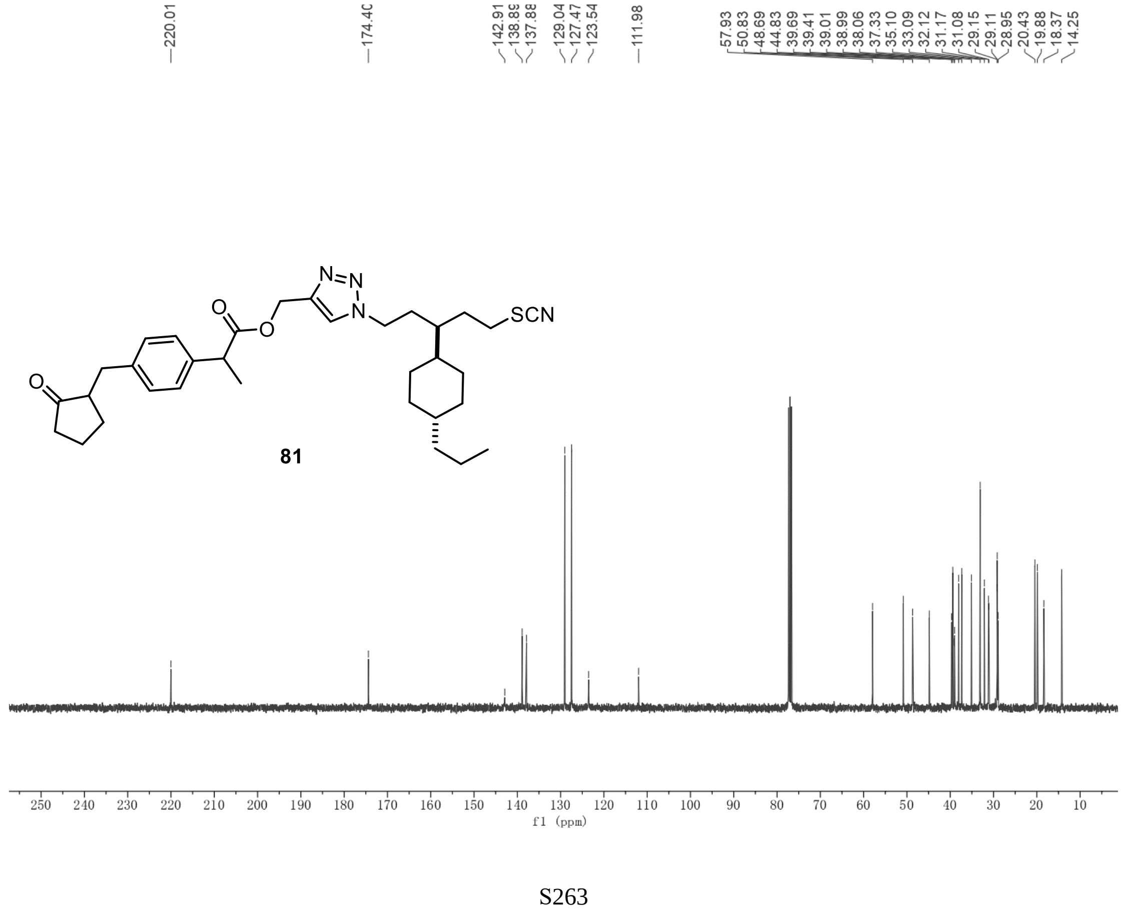

^{1}H NMR (400 MHz, $CDCl_3$) of **82**

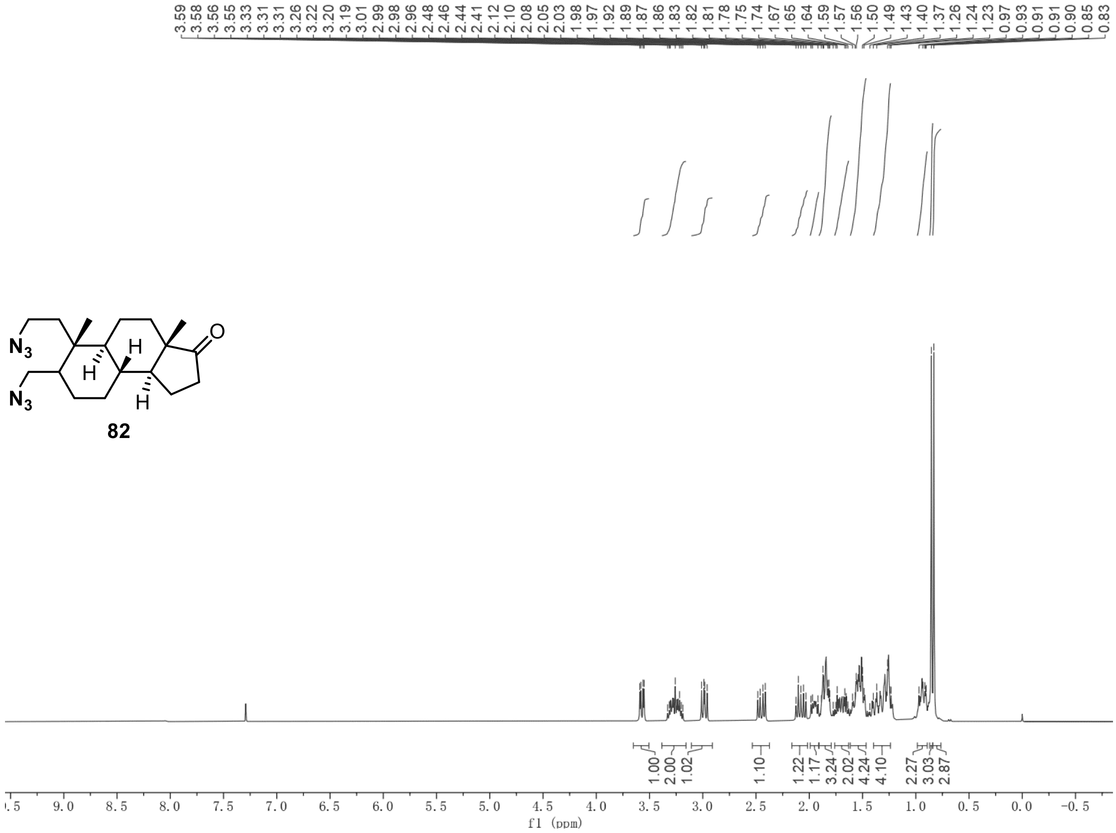

^{13}C NMR (100 MHz, $CDCl_3$) of **82**

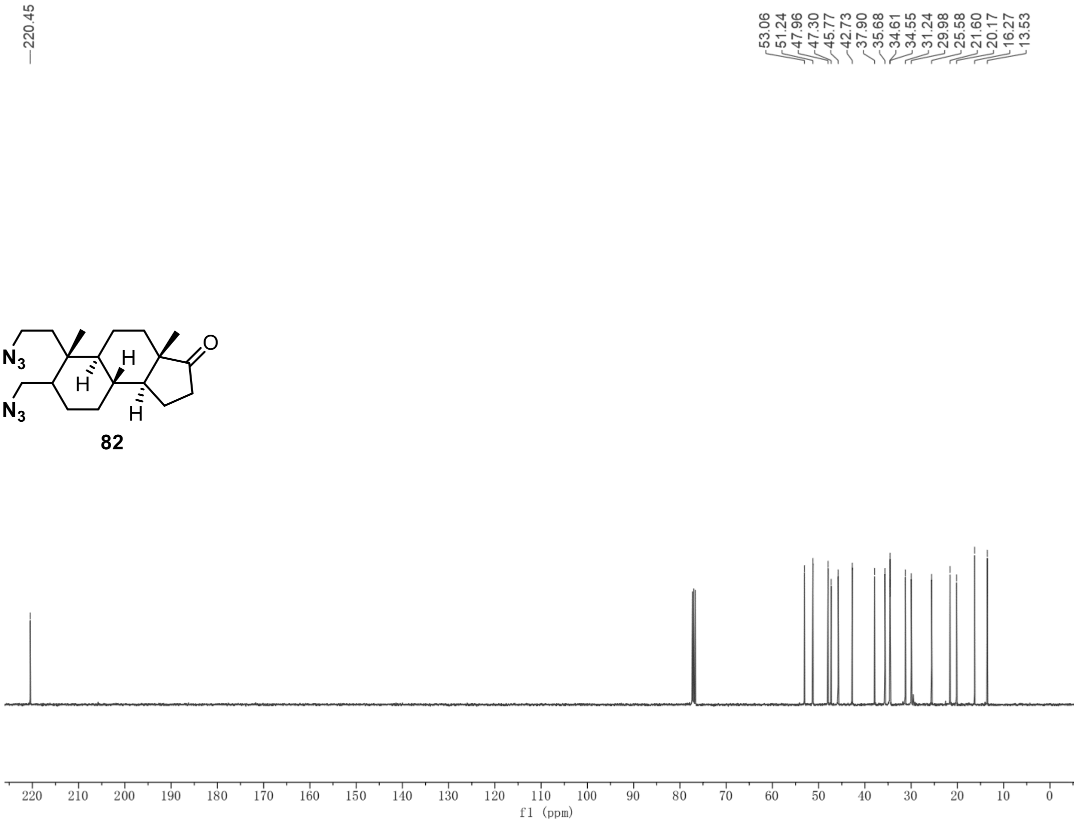

^{1}H NMR (400 MHz, $CDCl_3$) of **83**

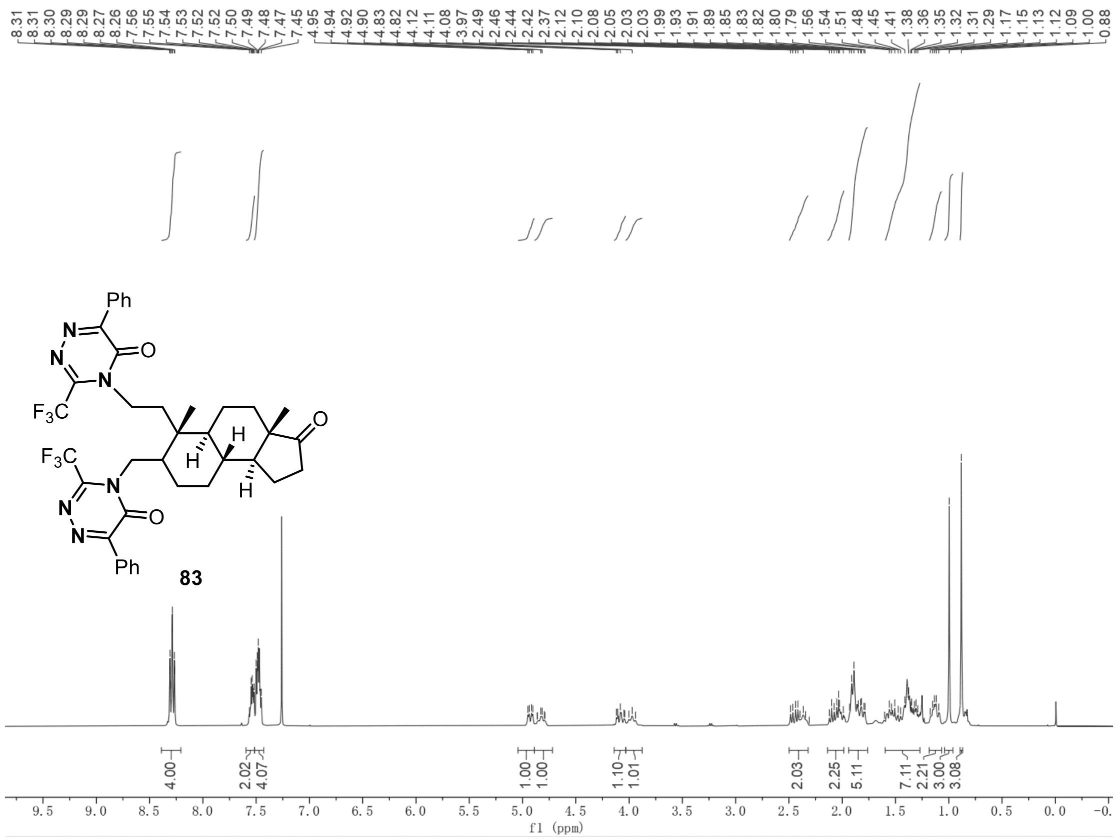

^{19}F NMR (376 MHz, $CDCl_3$) of **83**

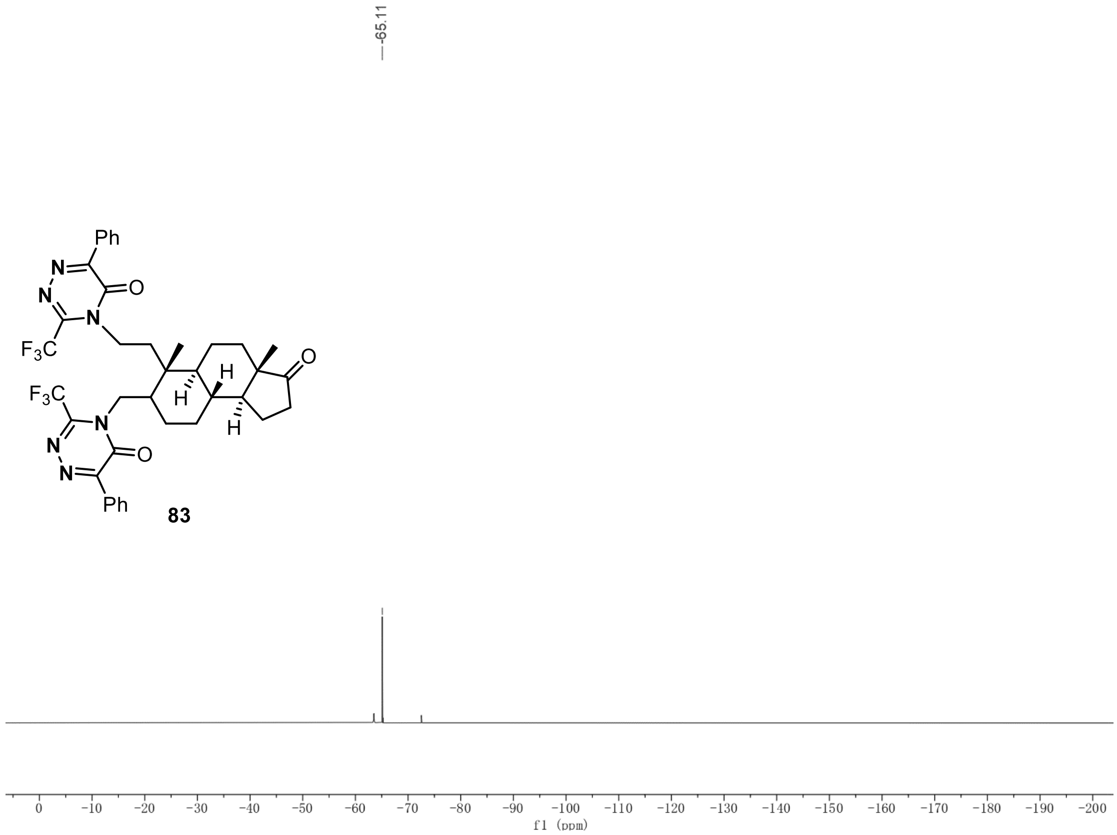

^{13}C NMR (100 MHz, $CDCl_3$) of **83**

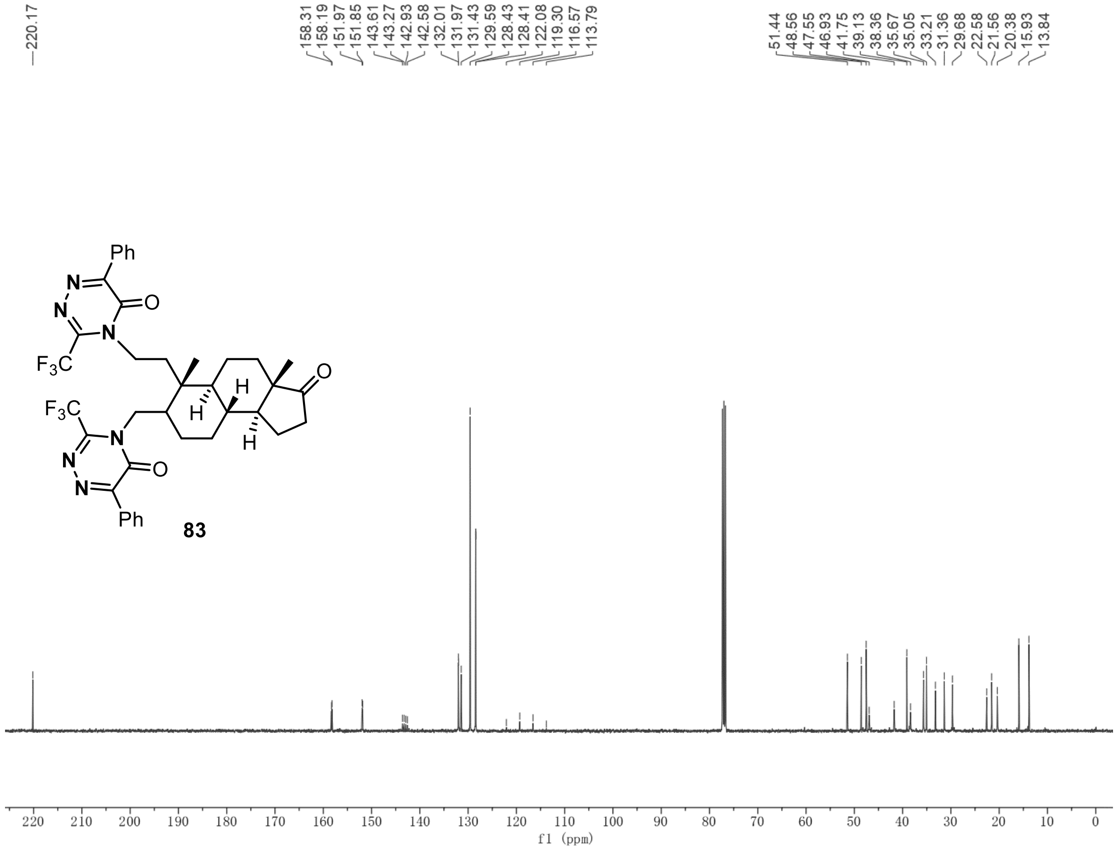

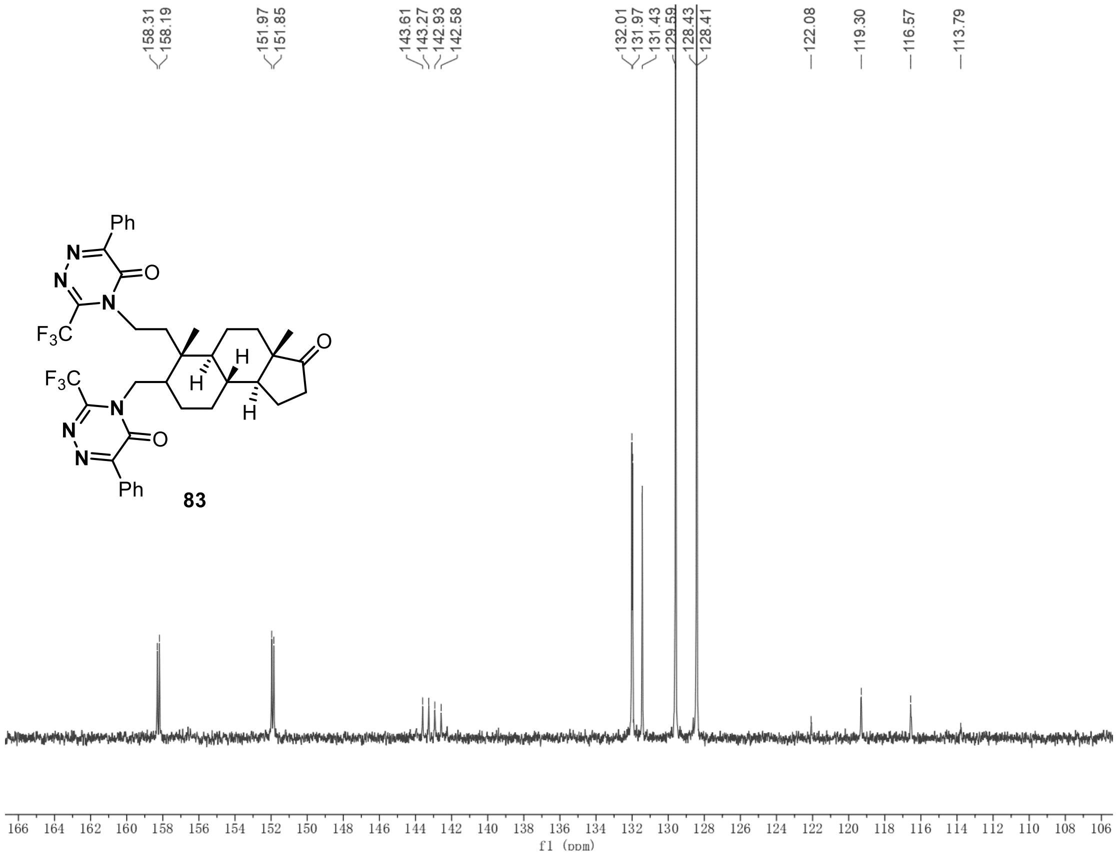

^{1}H NMR (400 MHz, $CDCl_3$) of **84**

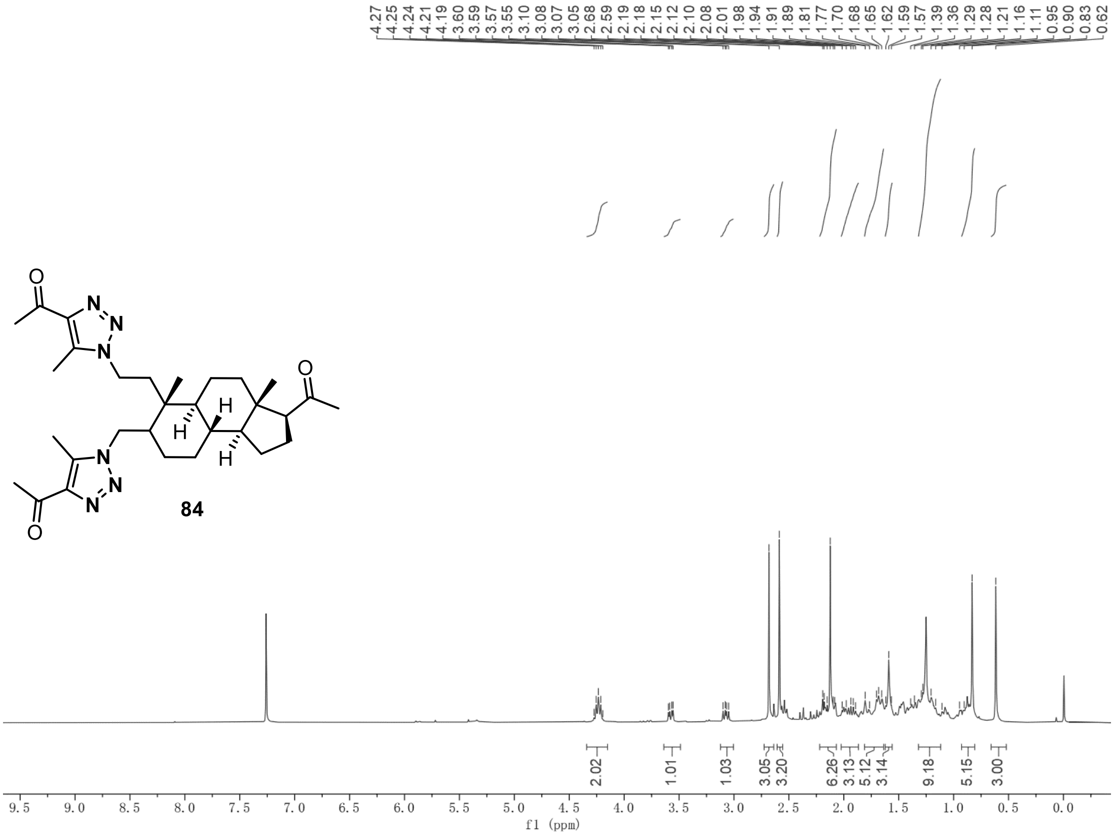

^{13}C NMR (100 MHz, $CDCl_3$) of **84**

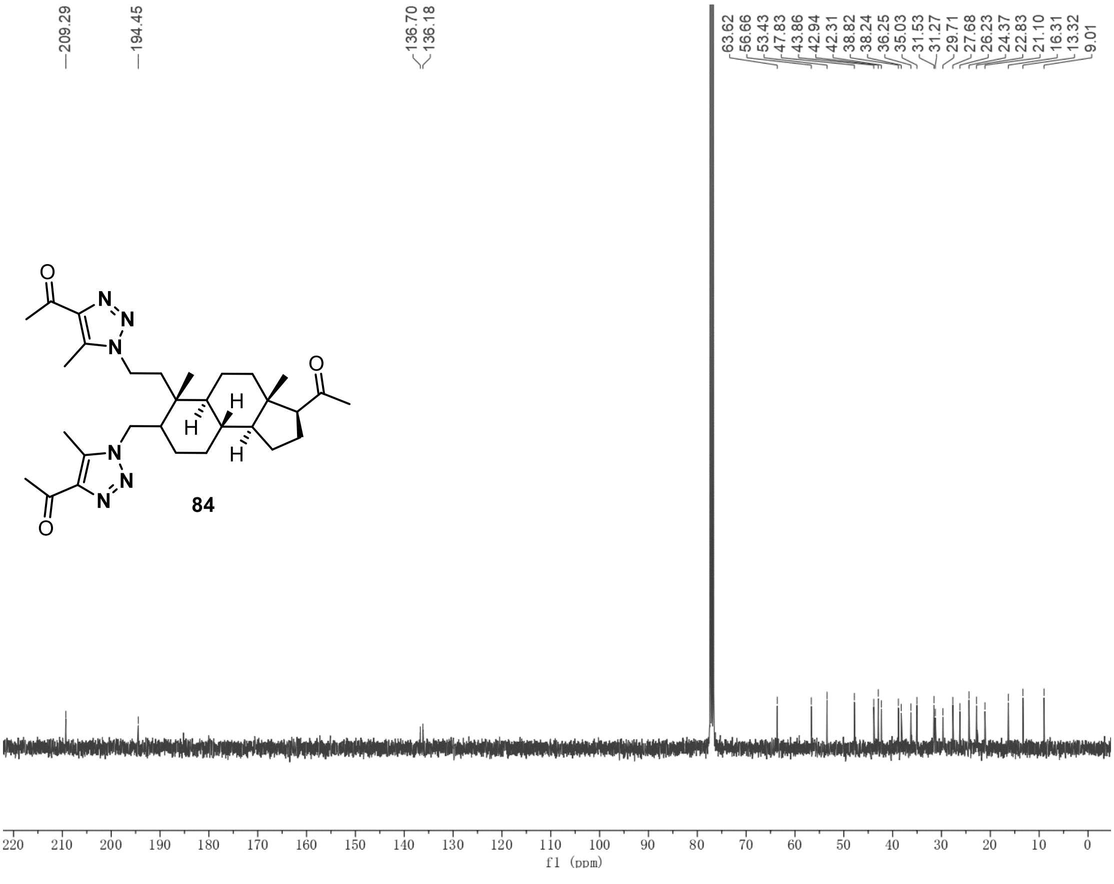

^{1}H NMR (400 MHz, $CDCl_3$) of **85**

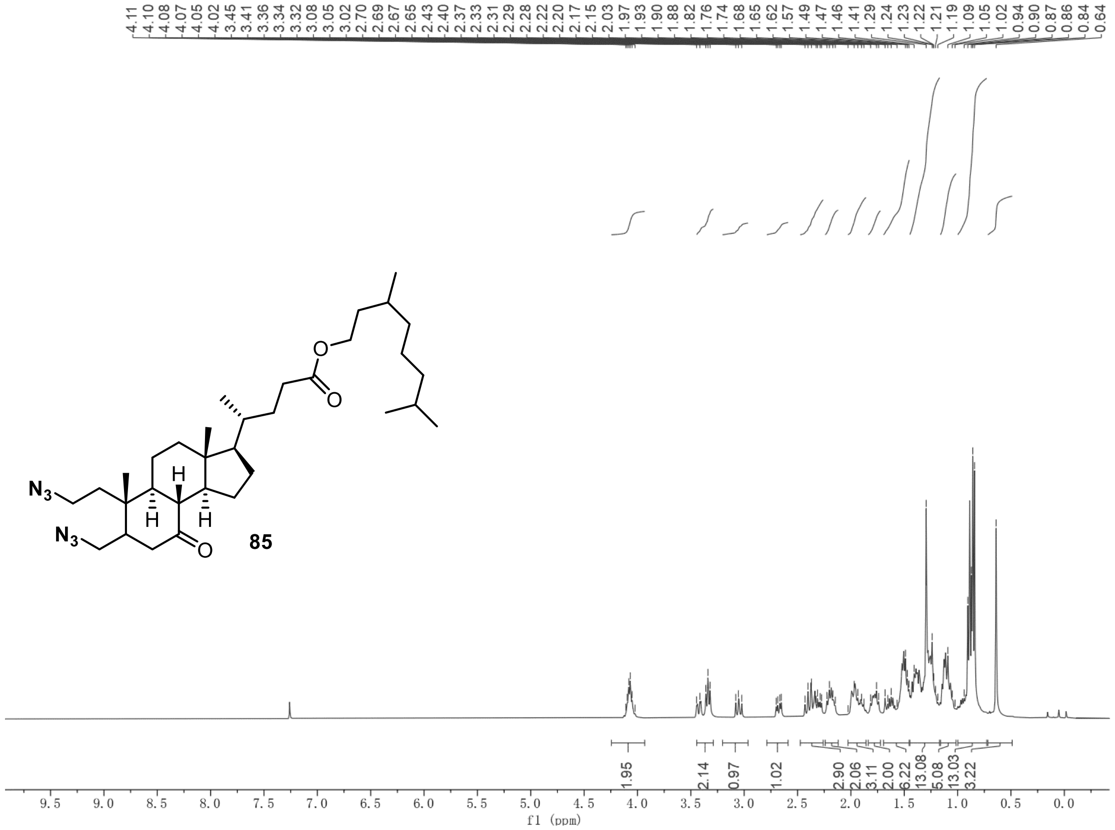

^{13}C NMR (100 MHz, $CDCl_3$) of **85**

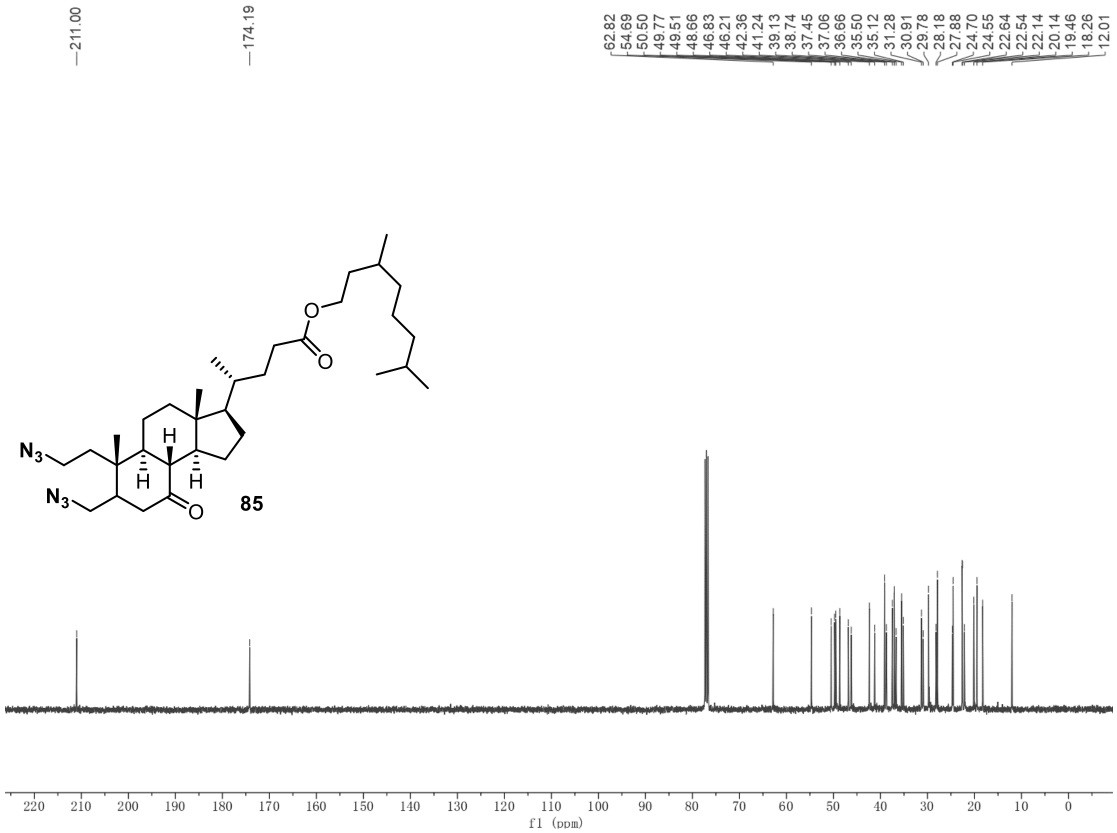

^{1}H NMR (400 MHz, $CDCl_3$) of **86**

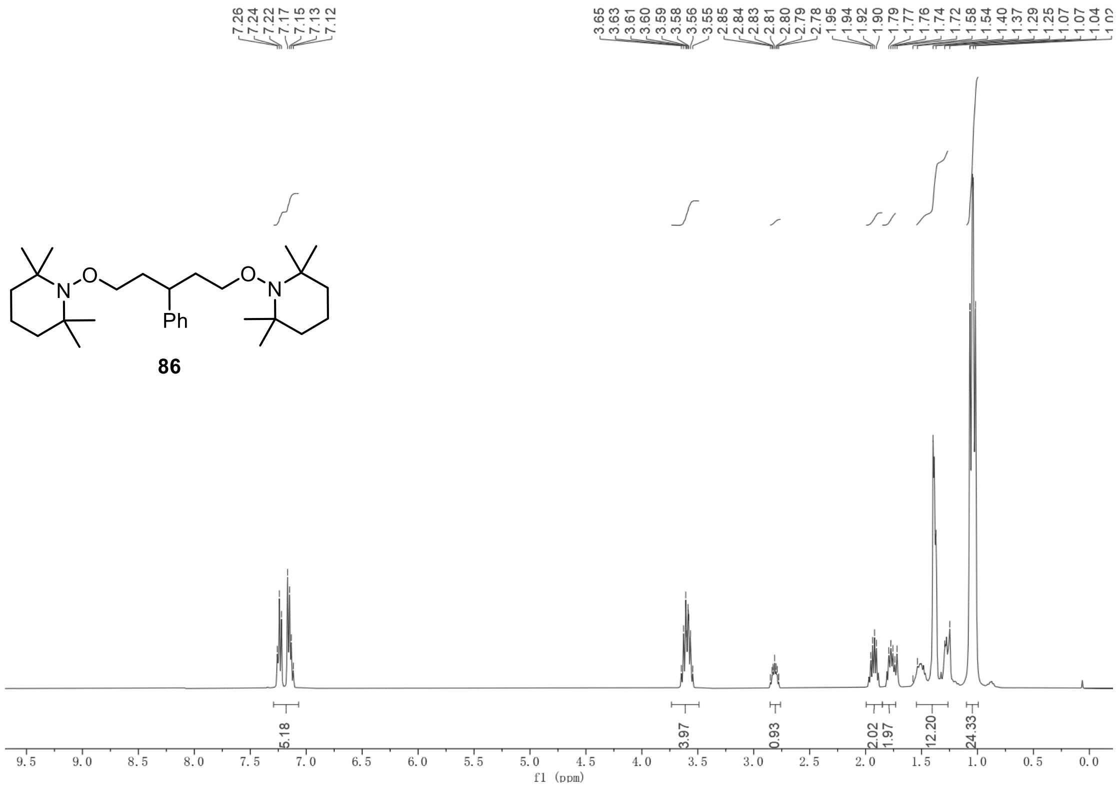

^{13}C NMR (100 MHz, $CDCl_3$) of **86**

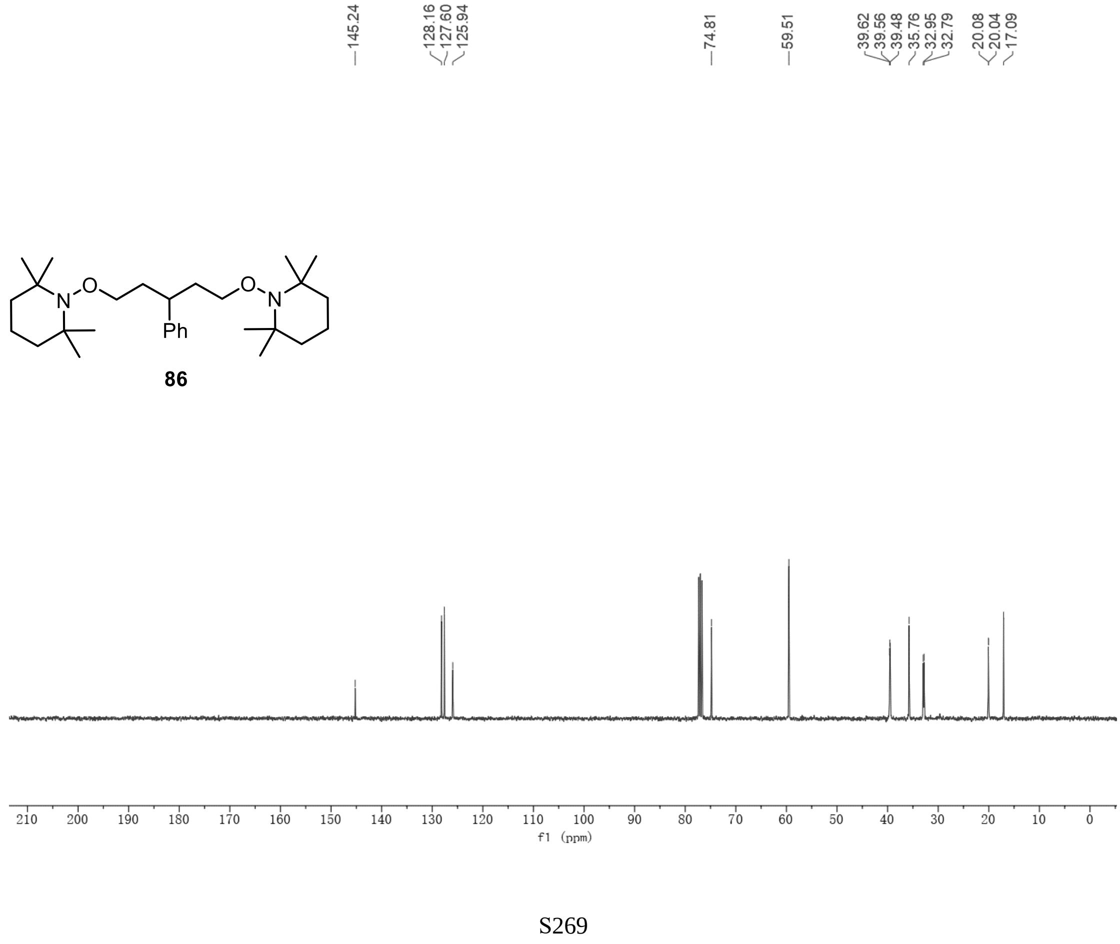

^{1}H NMR (400 MHz, $CDCl_3$) of **1'**

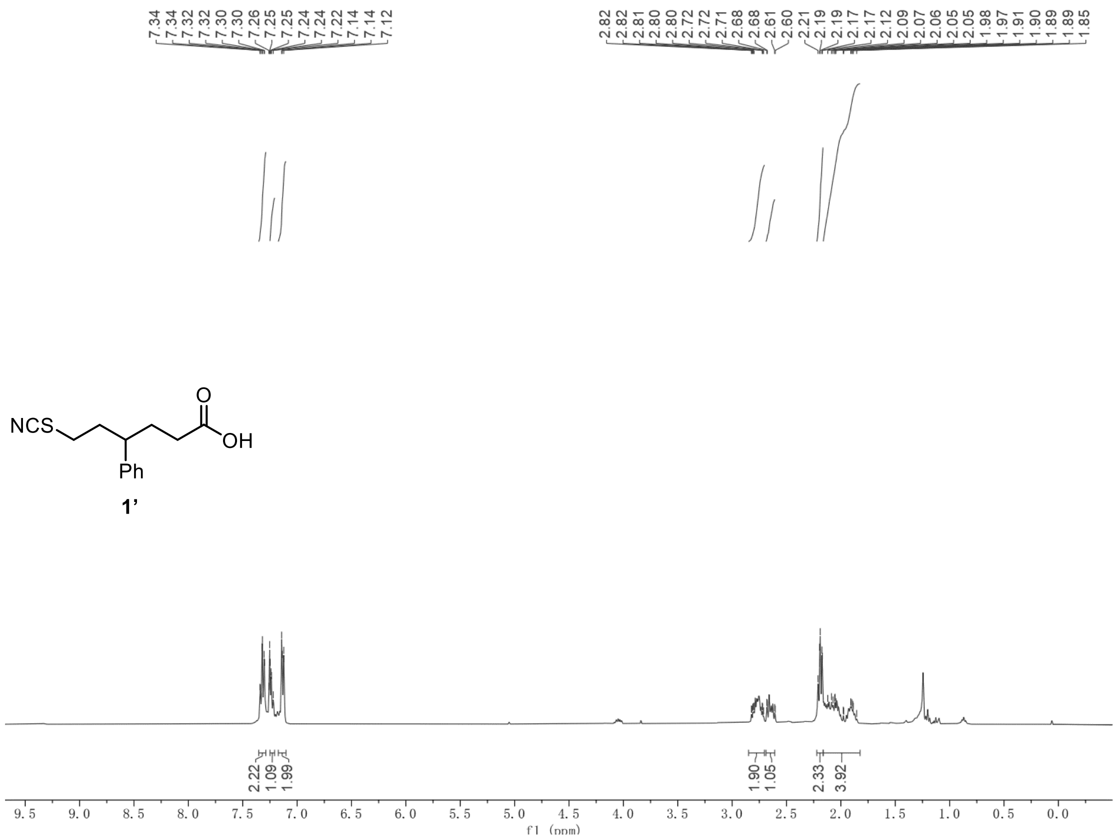

^{13}C NMR (100 MHz, $CDCl_3$) of **1'**

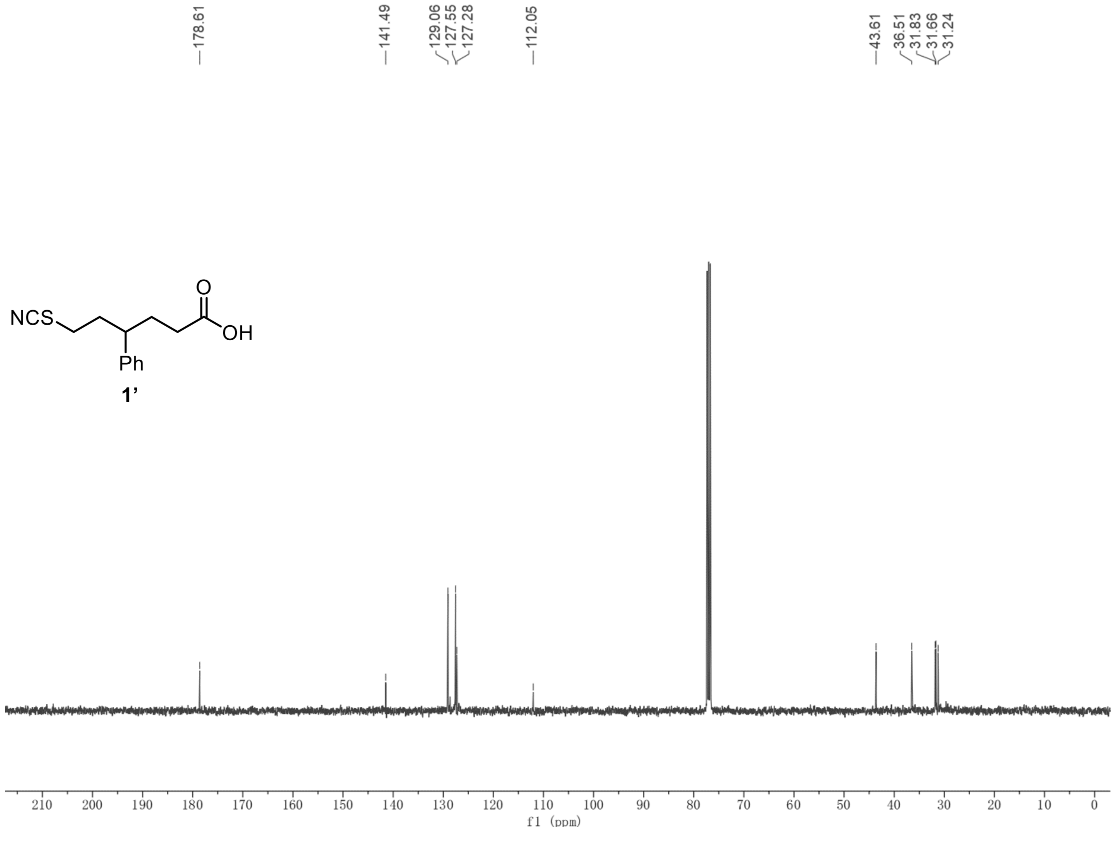

^{1}H NMR (400 MHz, $CDCl_3$) of **87**

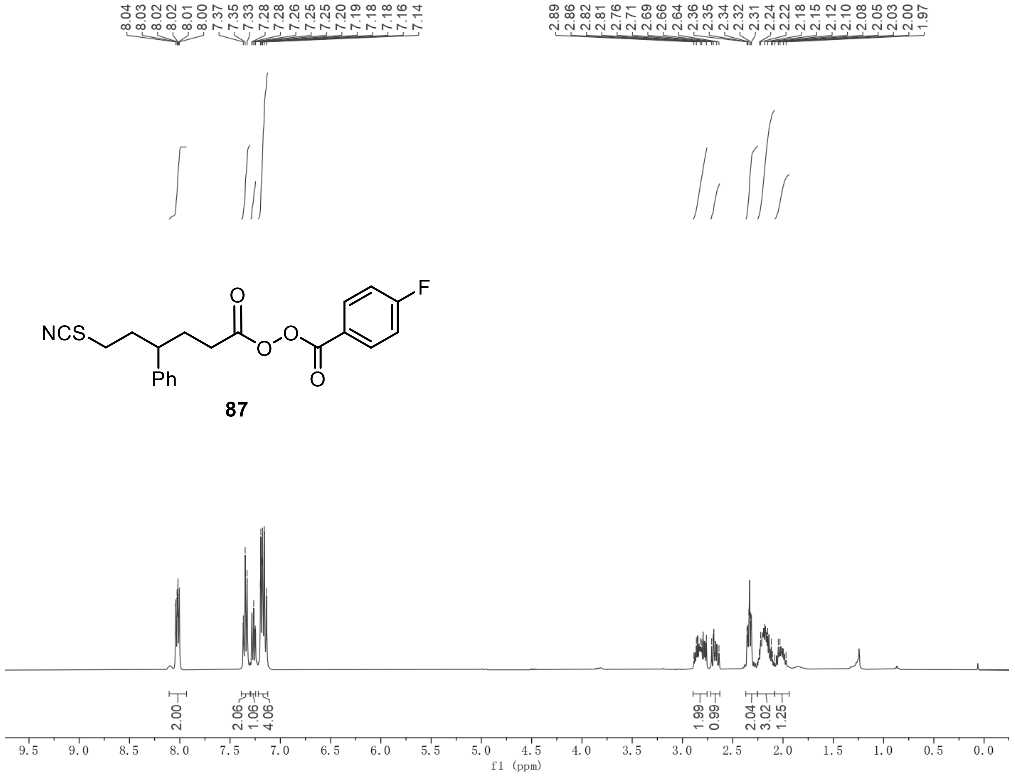

^{19}F NMR (376 MHz, $CDCl_3$) of **87**

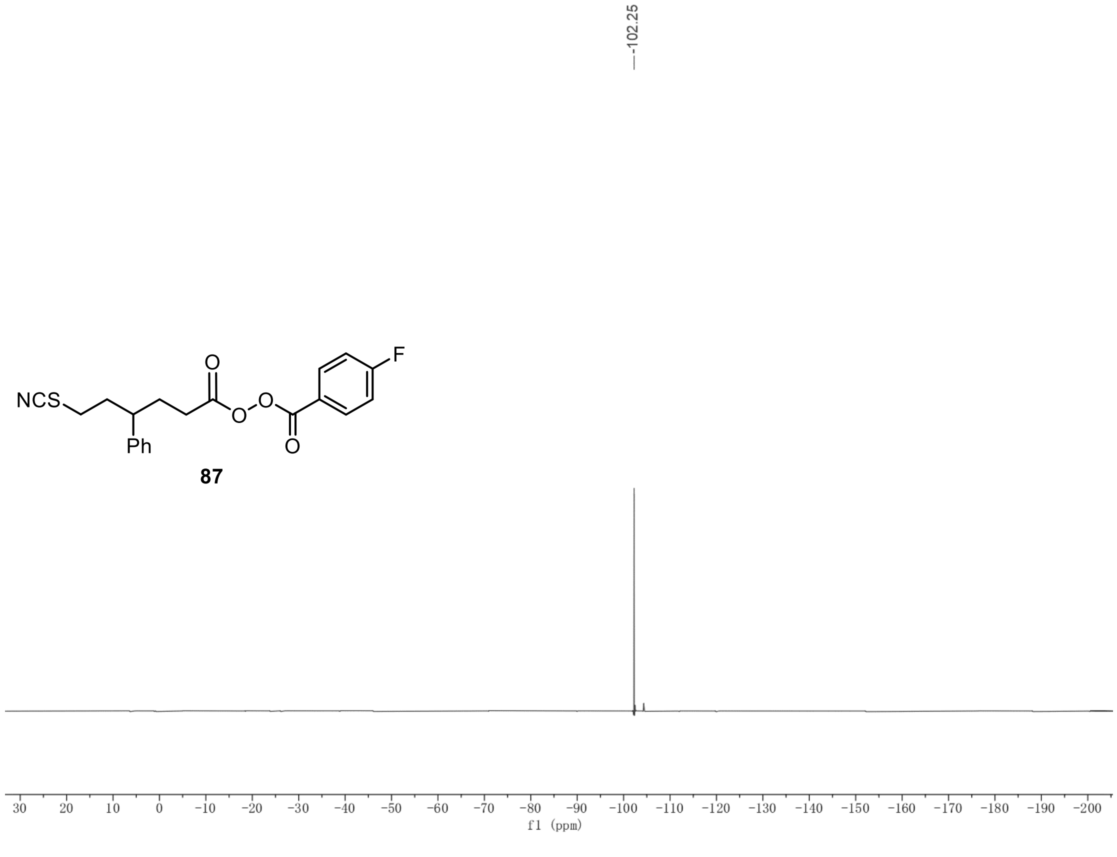

^{13}C NMR (100 MHz, $CDCl_3$) of **87**

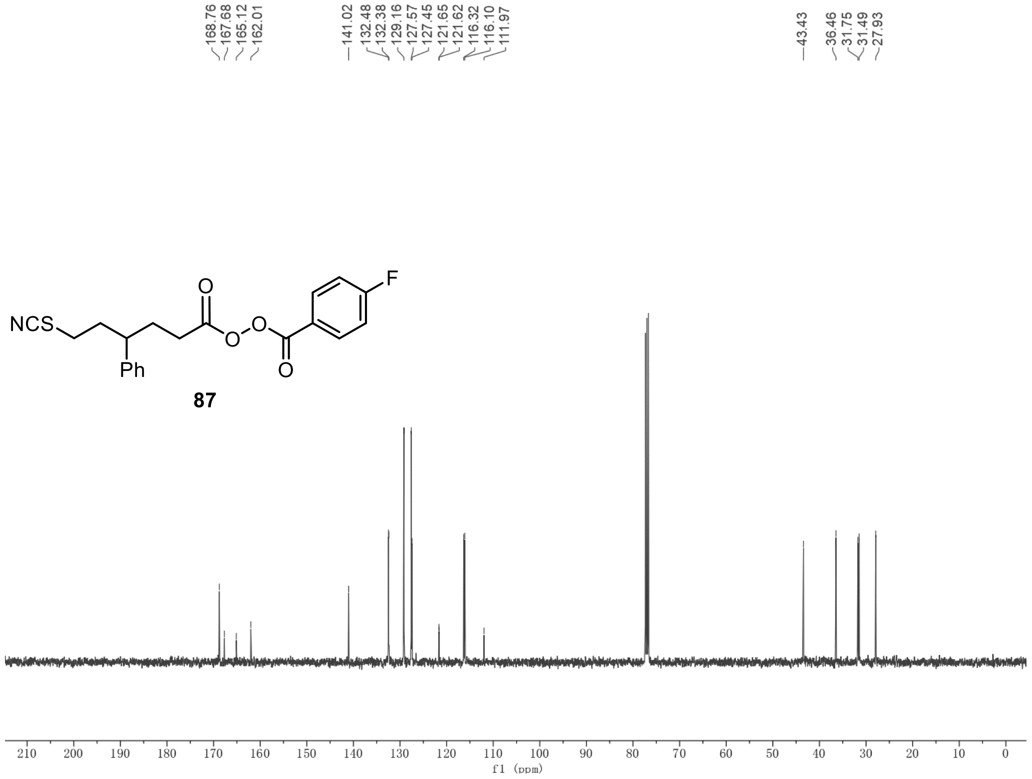

^{1}H NMR (400 MHz, $CDCl_3$) of **C1**

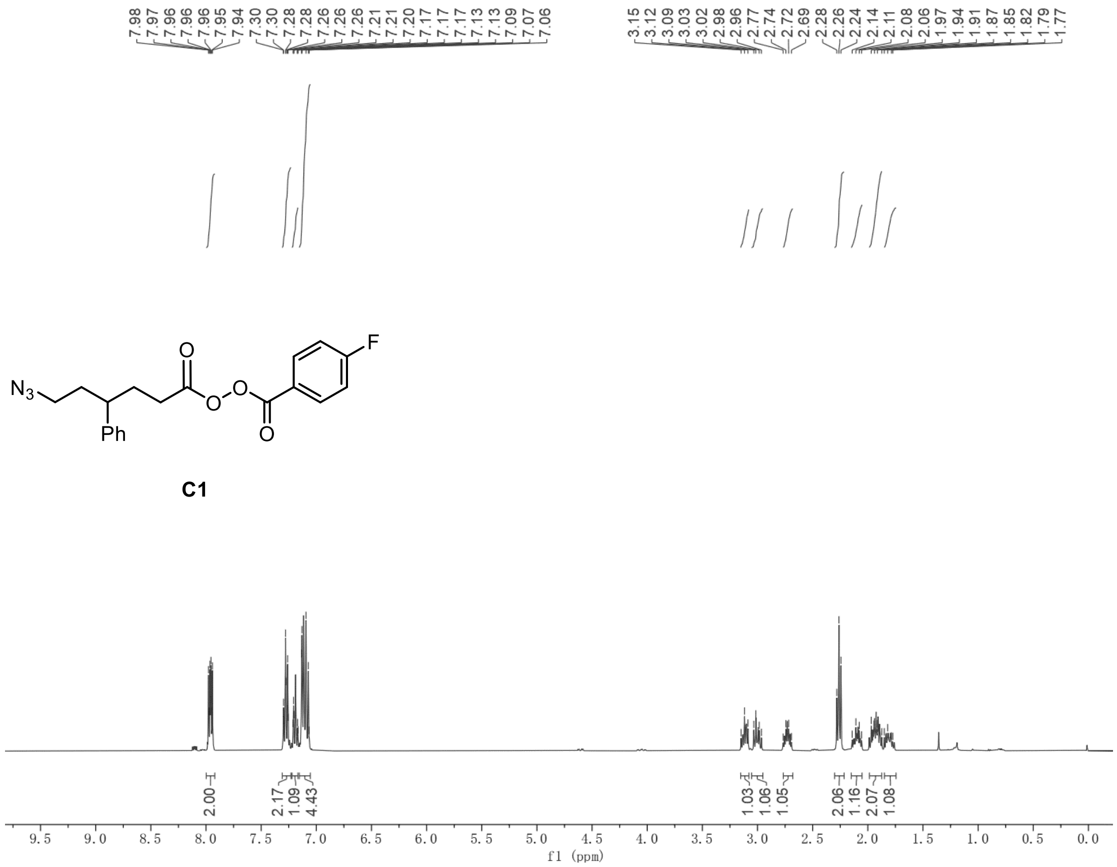

^{19}F NMR (376 MHz, $CDCl_3$) of **C1**

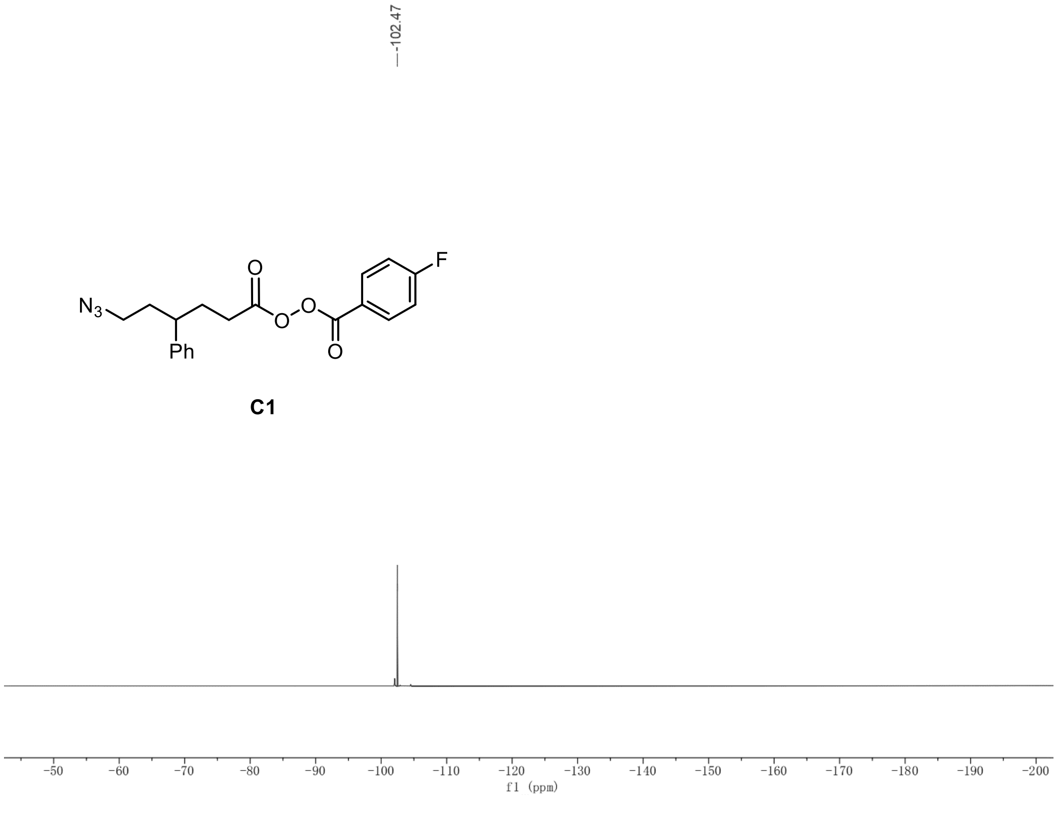

^{13}C NMR (100 MHz, $CDCl_3$) of **C1**

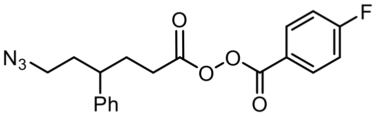

C1

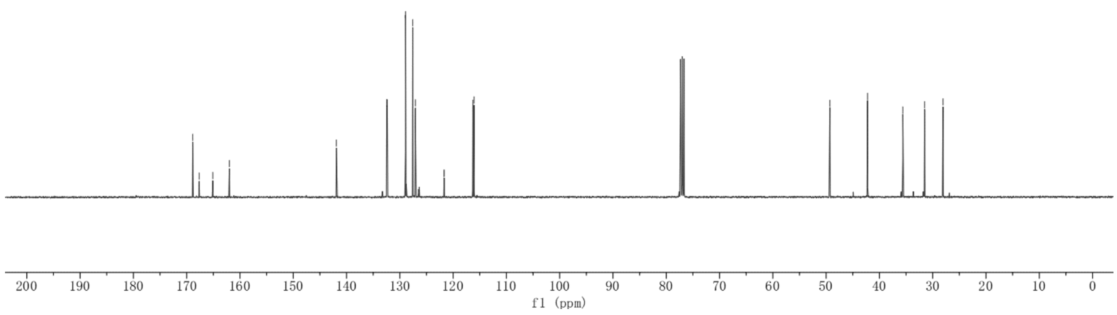

Supplementary References

1. Terent'ev, A. O., Platonov, M. M., Kashin, A. S. & Nikishin, G. I. Oxidation of cycloalkanones with hydrogen peroxide: an alternative route to the Baeyer–Villiger reaction. Synthesis of dicarboxylic acid esters. *Tetrahedron* **64**, 7944–7948 (2008).
2. Li, Y., Hao, H.-D., Zhang, Q. & Wu, Y. A broadly applicable mild method for the synthesis of *gem*-diperoxides from corresponding ketones or 1,3-dioxolanes. *Org. Lett.* **11**, 1615–1618 (2009).
3. Feng, Q., Wang, Q. & Zhu, J. Oxidative rearrangement of 1,1-disubstituted alkenes to ketones. *Science* **379**, 1363–1368 (2023).
4. Zhang, Z. & Dong, G. Carbonyl-to-sulfur swap enabled by sequential double carbon-carbon bond activation. *Science* **388**, 1436–1440 (2025).
5. Ganji, S., Svensson, F. G. & Unelius, C. R. Asymmetric synthesis of oxygenated monoterpenoids of importance for bark beetle ecology. *J. Nat. Prod.* **83**, 3332–3337 (2020).
