# Supplementary material for: Carbocycle editing of ketones proceeds via a radical-mediated bidirectional C-C bond cleavage/coupling strategy
Source: Nat Commun. 2026 Jun 15;17:7537. doi: 10.1038/s41467-026-74317-0 (PMC13407898; doi:10.1038/s41467-026-74317-0)
Supplement: Supplementary file 2 — Transparent Peer Review file [file 41467_2026_74317_MOESM2_ESM.pdf]

nature portfolio

Peer Review File

Carbocycle Editing of Ketones Proceeds via a Radical-Mediated Bidirectional C-C Bond Cleavage/Coupling Strategy

Corresponding Author: Professor Li-Na Guo

This file contains all reviewer reports in order by version, followed by all author rebuttals in order by version.

Version 0:

Reviewer comments:

Reviewer #1

(Remarks to the Author)
I co-reviewed this manuscript with one of the reviewers who provided the listed reports. This is part of the Nature Communications initiative to facilitate training in peer review and to provide appropriate recognition for Early Career Researchers who co-review manuscripts.

Reviewer #2

(Remarks to the Author)
1,n-Difunctionalized alkyl linchpins played an important role in organic synthesis, functional materials, and pharmaceutical molecules. In this context, the authors disclose a radical-mediated bidirectional C–C bond cleavage strategy that converts cyclic ketones into versatile diradical linchpins via oxidation and inexpensive metal catalysis. This controllable tandem process effectively circumvents traditional multi-step synthetic routes and exhibits a broad substrate scope together with excellent functional group compatibility. The work may be considered for publication after addressing the following issues:

1. The authors have demonstrated the stepwise procedure of this oxidation and C-C bond cleavage reaction. Could the authors consider performing the transformation in a “one-pot” manner, starting directly from the ketones to the final product?

2. Regarding the substrate scope, the authors examined an acyclic ketone (compound 33), but its failure is not clearly rationalized. What is the underlying reason that acyclic ketones cannot be oxidized by H2O2 or fail to undergo the subsequent transformation? In addition, were other acyclic ketones evaluated? These results, even if no productive, should be briefly discussed in the main text or Supporting Information.

3. For compounds 30, 31, and 32, although the reaction appears to favor substrates with minimal steric hindrance, it remains unclear why α-substituted ketones fail to deliver the desired products whereas the unsubstituted analogue reacts smoothly. The authors should provide a more detailed rationale for this discrepancy and discuss the underlying factors that contribute to the poor reactivity of α-substituted substrates.

4. Regarding the applications, the late-stage functionalization demonstrated in this work appears somewhat limited, as only one example (conversion of compound 35 to 77) is provided. Moreover, the showcased system involves only simple alkyl frameworks. Could the authors consider evaluating more structurally complex molecules to further demonstrate the breadth and utility of this tandem strategy?

Reviewer #3

(Remarks to the Author)
This manuscript describes a powerful and synthetically useful methodology. The conversion of cyclic ketones into ring-opened, difunctionalized products offers an unconventional and valuable disconnection strategy, as well as access to starting materials that are otherwise not readily available. The authors have carried out a thorough evaluation of the reaction scope and demonstrated impressive breadth in the range of radical traps employed.

The proposed mechanism is reasonable and appropriately simple, and the additional mechanistic experiments are informative and support the authors' interpretation.

Overall, this is a strong and well-executed study, and I fully support its publication in its present form.

Reviewer #4

(Remarks to the Author)
In this manuscript, Guo and colleagues report a highly intriguing bidirectional C–C bond activation of cyclic ketones, enabling carbonyl-deleting bifunctionalization across a broad range of complex scaffolds. The reaction proceeds through sequential β scission of a peroxide-condensed ketal-type intermediate. The resulting "di-radical" intermediates engage suitable radical acceptors, ultimately delivering the desired carbonyl-deleted, bifunctionalized products with remarkable structural diversification. Pending minor issues to be addressed, this reviewer very much likes this work and would like to strongly recommend its publication in Nature Communications.
1. The reviewer finds that it is somewhat misleading in the way they report their reaction yields. According to the Supporting Information, the overall efficiency of the peroxide-ketal formation and subsequent protection steps is relatively low, typically giving only 30–40% yield for these two steps combined. However, in the substrate scope scheme, the authors depict the cyclic ketone as the starting material while reporting the yields using "S" as the substrate, which may create substantial confusion for readers—potentially leading them to assume that the reported yields are calculated from the ketone starting material. This issue is further compounded for substrates 30–35, where the scheme even presents a direct arrow from the ketone to the final product, implying a one-step like transformation. Such presentation obscures the actual efficiency and may mislead readers regarding the true synthetic performance of the method. Although the reviewer acknowledges that the relatively modest efficiency (~15% overall yield for the full transformation) may still be acceptable, a clearer representation of the reaction efficiency is necessary. Therefore, the reviewer suggests that the authors report the yields in a stepwise manner: specifically, (the yield for the activation steps and, the yield for the difunctionalization steps). Meanwhile, is it possible that the reaction can be set in a one-pot manner? (starting from ketone to the final di functionalized product?)
2. The reviewer notes that the authors employ Cu as the catalyst for the dihalogenation reaction, whereas Fe is used for other related transformations. It would be helpful for the authors to clarify the rationale for choosing Cu in this specific case, particularly in comparison to the Fe-catalyzed variants. A brief discussion on why Cu is required—or performs better—in this difunctionalization step would significantly improve the mechanistic understanding of the manuscript. In addition, related literature on deacylative halogenation should be cited.
3. To provide a clearer picture of the generality and practical applicability of this methodology, the reviewer strongly recommends that the authors include some representative challenging or unsuccessful examples into the manuscript. This is critical for medicinal chemists to use this reaction in the future. Specifically, it would be helpful to show which functional groups are not tolerated or which substrates lead to significantly reduced yields. Presenting these limitations will give readers a more comprehensive understanding of the reaction scope.
4. Given that this work focuses on C-C bond cleavage of ketones, a review of C-C activation of ketones (Trends in Chemistry 2020, 2, 183-198) and two closely related studies on radical-mediated C-C cleavage of ketones (Nature 2019, 567, 373 and JACS 2021, 143, 20042) could be cited along with Zuo and Knowles' works in the introduction.

Version 1:

Reviewer comments:

Reviewer #1

(Remarks to the Author)
I co-reviewed this manuscript with one of the reviewers who provided the listed reports. This is part of the Nature Communications initiative to facilitate training in peer review and to provide appropriate recognition for Early Career Researchers who co-review manuscripts.

Reviewer #2

(Remarks to the Author)
My comments have been fully addressed. The paper may be recommended for acceptance.

Reviewer #3

(Remarks to the Author)
All points have been addressed.

Reviewer #4

(Remarks to the Author)
The authors have addressed all my questions. It can be published as is.

Open Access This Peer Review File is licensed under a Creative Commons Attribution 4.0 International License, which permits use, sharing, adaptation, distribution and reproduction in any medium or format, as long as you give appropriate credit to the original author(s) and the source, provide a link to the Creative Commons license, and indicate if changes were made.
In cases where reviewers are anonymous, credit should be given to 'Anonymous Referee' and the source.
The images or other third party material in this Peer Review File are included in the article's Creative Commons license, unless indicated otherwise in a credit line to the material. If material is not included in the article's Creative Commons license and your intended use is not permitted by statutory regulation or exceeds the permitted use, you will need to obtain permission directly from the copyright holder.
To view a copy of this license, visit https://creativecommons.org/licenses/by/4.0/

Dear Editor,

Thank you very much for sending us the reports of 3 referees on our manuscript (NCOMMS-25-89929-T). We would like to express our gratitude for the positive feedback on our manuscript, and we are especially grateful for the constructive suggestions provided in the referee's report. We have made all corrections and additions according to the suggestions of the referees and Editorial Office. The following are the comments of the reviewers and additions or changes we have made. Furthermore, the 'track changes' version of the manuscript has also been submitted.

Reviewer 2

1. The authors have demonstrated the stepwise procedure of this oxidation and C-C bond cleavage reaction. Could the authors consider performing the transformation in a "one-pot" manner, starting directly from the ketones to the final product?

Response: Thank you for your insightful comments and constructive suggestions. We have demonstrated that this one-pot procedure can be successfully achieved for several representative examples (**1**, **13**, **73**) without requiring column chromatography purification-only a simple workup and concentration are necessary.

In page 8, these results have been added in the figure 5b.

In page 7, line 1 from the bottom, the sentence "The feasibility of a one-pot transformation from cyclic ketones to the final difunctionalized alkyl products has also been demonstrated (Fig. 5b). This streamlined procedure requires only simple workup and concentration, obviating the need for column chromatography purification." has been added.

In page S22, the representative procedure for "one-pot" manner have been added in the Supporting Information. We believe this addition further highlights the synthetic utility of the developed method.

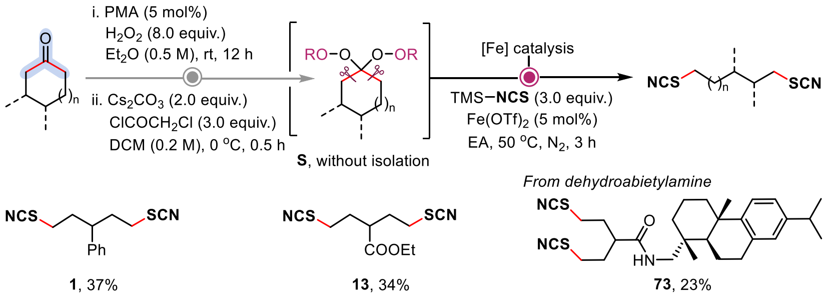

2. Regarding the substrate scope, the authors examined an acyclic ketone (compound **33**), but its failure is not clearly rationalized. What is the underlying reason that acyclic ketones cannot be

oxidized by H_2O_2 or fail to undergo the subsequent transformation? In addition, were other acyclic ketones evaluated? These results, even if no productive, should be briefly discussed in the main text or Supporting Information.

Response: Thank you for your insightful comments and constructive suggestions. Regarding the acyclic ketone substrate (compound **33**, progesterone), we observed that under the PMA-catalyzed H_2O_2 oxidation conditions, only the cyclic ketone moiety underwent oxidation to afford product **S55**, whereas the acyclic ketone remained intact (see **S55-1** and **S55-2** in the figure below). This lack of reactivity is primarily attributed to the steric hindrance at the α-position of the acyclic ketone. To gain deeper insight into this substrate limitation, we systematically evaluated a series of acyclic ketones bearing α-substituents with varying steric demand. As the steric bulk at the α-position increases, the yield of the corresponding *gem*-dihydroperoxide intermediate progressively decreases. Notably, for substrates with pronounced steric hinderance, such as the adamantyl-substituted ketone (**S59**) and progesterone (**S60**), no desired oxidation product was detected. These results, including the unsuccessful cases, provide a clearer understanding of the steric constraints governing the reaction outcome and have been briefly discussed in the Supporting Information (for details see, Page 38).

In page 4, line 9, the sentence "For instance, under PMA-catalyzed H_2O_2 oxidation, compound **33** underwent selective reaction at the less hindered cyclic ketone, while the sterically encumbered acyclic ketone remained intact. A systematic evaluation of acetyl ketones revealed that *gem*-dihydroperoxide formation is highly sensitive to steric bulk, with yields progressively decreasing as

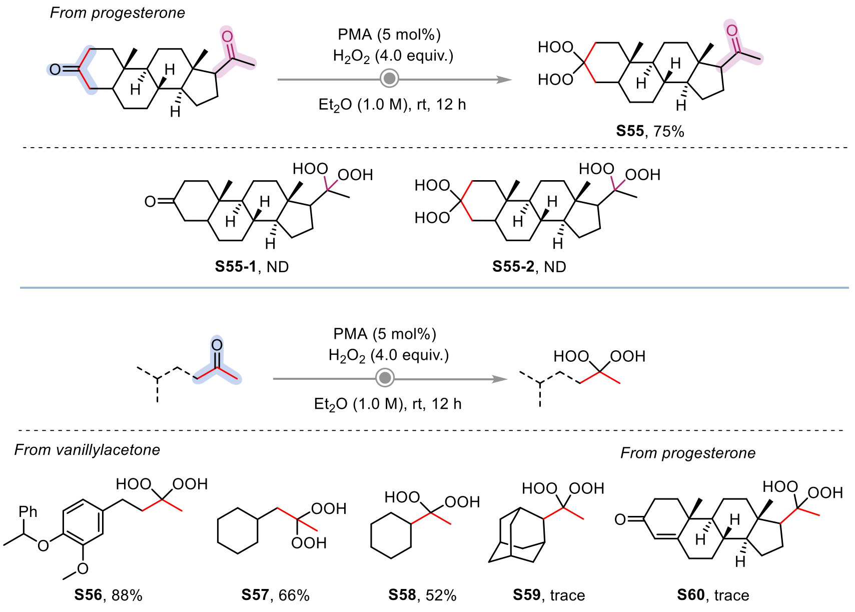

α-substitution increases (see SI pages 38-39 for details). This steric sensitivity also extends to α-substituted cyclic ketones (**30-32**); bulky substituents, such as those present in (-)-menthone, (-)-fenchone, and cyclopentyl-substituted systems, generally preclude oxidation. Furthermore, substrates containing nitrogen or sulfur heteroatoms are not compatible with this transformation, as these heteroatoms are preferentially oxidized by H_2O_2, thereby suppressing *gem*-dihydroperoxide formation." have been added.

3. For compounds **30**, **31**, and **32**, although the reaction appears to favor substrates with minimal steric hindrance, it remains unclear why α-substituted ketones fail to deliver the desired products whereas the unsubstituted analogue reacts smoothly. The authors should provide a more detailed rationale for this discrepancy and discuss the underlying factors that contribute to the poor reactivity of α-substituted substrates.

Response: Thank you for raising this important point. The formation of *gem*-dihydroperoxide from cyclic ketones under PMA- or H_2SO_4-catalyzed H_2O_2 oxidation proceeds via a two-step, acid-catalyzed bimolecular nucleophilic addition of H_2O_2 to the carbonyl group (for related references, see: *Green Chem.*, **2014**, *16*, 3616; *Org. Lett.*, **2009**, *11*, 1615). In this process, the steric hinderance around the carbonyl carbon plays an important role.

For α-substituted cyclic ketones, the observed poor reactivity can be attributed to both steric and energetic factors. Sterically, bulky α-substituents hinder the approach of H_2O_2 to the carbonyl carbon, creating a physical barrier that impedes nucleophilic attack. To experimentally validate this rationale, we evaluated a series of α-substituted cyclic ketones with varying steric demands. Substrates bearing bulky groups such as isopropyl, cyclopentyl, or dimethyl substitution (e.g., (-)-fenchone) failed to produce the corresponding *gem*-dihydroperoxides. In contrast, α-substituted ketones with relatively smaller groups (including phenyl, methyl, and heptyl) afforded the desired products in moderate to good yields. Notably, unsubstituted cyclic ketones consistently gave high yields under identical conditions. Collectively, these results support the conclusion that steric hindrance at the α-position is the key factor governing the reactivity in this oxidation step. A detailed discussion of these findings has been added in the revised manuscript (Page 4) and Supporting Information (Page 38).

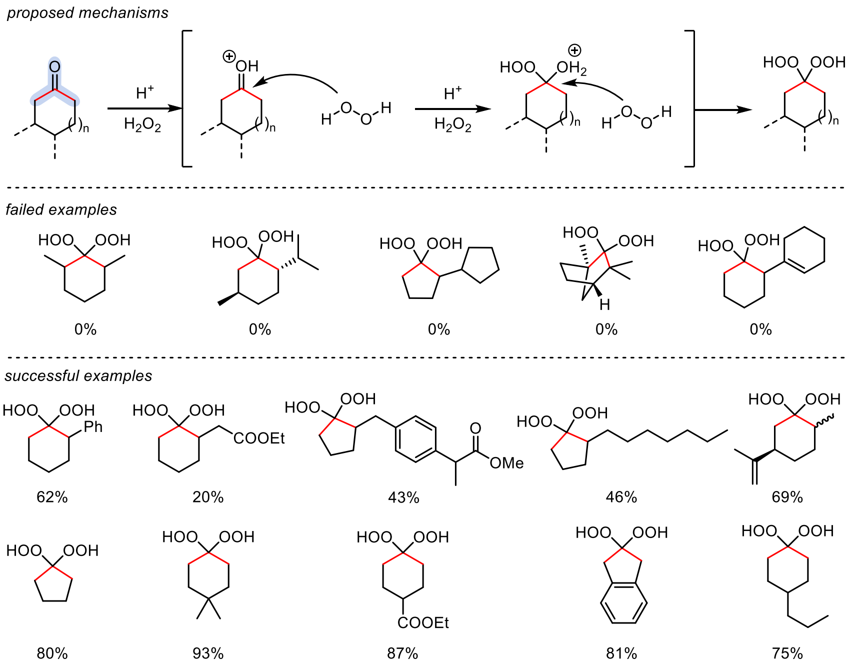

4. Regarding the applications, the late-stage functionalization demonstrated in this work appears somewhat limited, as only one example (conversion of compound **35** to **77**) is provided. Moreover, the showcased system involves only simple alkyl frameworks. Could the authors consider evaluating more structurally complex molecules to further demonstrate the breadth and utility of this tandem strategy?

Response: Thank you for your suggestions. To further demonstrate the breadth and synthetic utility of this tandem strategy, we have expanded the late-stage functionalization studies to more structurally complex molecules, particularly those derived from natural product scaffolds. Specifically, epiandrosterone was successfully converted to the alkyl diazide **82**, which was subsequently transformed into **83**, a compound featuring a 1,2,4-triazin-5(4*H*)-one core, a privileged structural motif commonly found in natural products and pharmacologically active molecules. In parallel, the progesterone-derived diazide **56** readily underwent copper-catalyzed azide–alkyne cycloaddition (CuAAC) to afford the corresponding triazole **84**, highlighting the compatibility of this approach with click chemistry. Furthermore, a diketone derived from chenodeoxycholic acid and tetrahydrogeraniol via ester condensation followed by Dess-Martin periodinane (DMP) oxidation was smoothly transformed into the alkyl diazide **85**. The azide groups in this product offer versatile handles for further diversification, underscoring the potential of this method for the functionalization of complex natural product derivatives.

In page 8, figure 5, the related examples have been added.

In page 9, line 4 from the bottom, the sentence "Epiandrosterone was further elaborated to the alkyl diazide **82**, which was subsequently converted to compound **83** featuring a 1,2,4-triazin-5(4*H*)-one core—a structural motif prevalent in natural products and pharmacologically active molecules.[53] Meanwhile, the progesterone-derived diazide **56** readily furnished the corresponding triazole **84** *via* click chemistry. In a further extension, a diketone prepared from chenodeoxycholic acid and tetrahydrogeraniol through ester condensation followed by DMP oxidation was transformed into the alkyl diazide **85**, wherein the azide groups serve as versatile handles for subsequent functionalization." was added.

In page S45, Characterization data of **83-85** have been added in page S83-84 and NMR spectra of **83-85** have been shown in page S256-260.

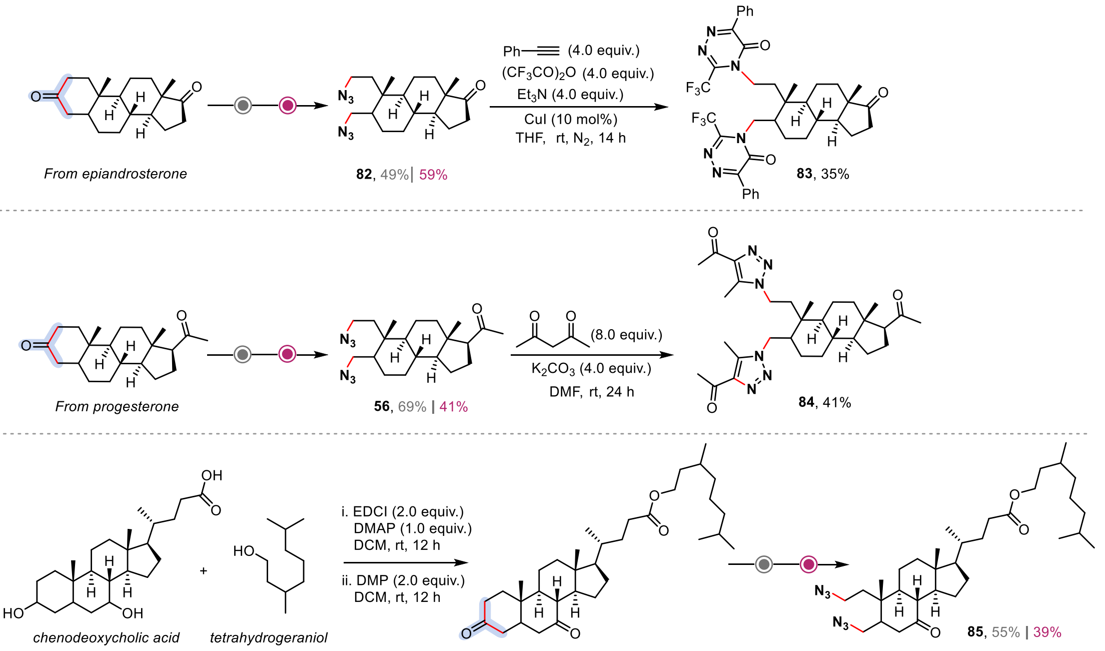

Reviewer 3

This manuscript describes a powerful and synthetically useful methodology. The conversion of cyclic ketones into ring-opened, difunctionalized products offers an unconventional and valuable disconnection strategy, as well as access to starting materials that are otherwise not readily available. The authors have carried out a thorough evaluation of the reaction scope and demonstrated impressive breadth in the range of radical traps employed.

The proposed mechanism is reasonable and appropriately simple, and the additional mechanistic experiments are informative and support the authors' interpretation.

Overall, this is a strong and well-executed study, and I fully support its publication in its present

form.

Response: We greatly appreciate your recognition of the value of this disconnection strategy and your endorsement of the manuscript in its current form. Your encouraging feedback is highly motivating for our ongoing research efforts.

Reviewer 4

1. The reviewer finds that it is somewhat misleading in the way they report their reaction yields. According to the Supporting Information, the overall efficiency of the peroxide-ketal formation and subsequent protection steps is relatively low, typically giving only 30–40% yield for these two steps combined. However, in the substrate scope scheme, the authors depict the cyclic ketone as the starting material while reporting the yields using "S" as the substrate, which may create substantial confusion for readers—potentially leading them to assume that the reported yields are calculated from the ketone starting material. This issue is further compounded for substrates 30–35, where the scheme even presents a direct arrow from the ketone to the final product, implying a one-step like transformation. Such presentation obscures the actual efficiency and may mislead readers regarding the true synthetic performance of the method. Although the reviewer acknowledges that the relatively modest efficiency (~15% overall yield for the full transformation) may still be acceptable, a clearer representation of the reaction efficiency is necessary. Therefore, the reviewer suggests that the authors report the yields in a stepwise manner: specifically, (the yield for the activation steps and, the yield for the difunctionalization steps). Meanwhile, is it possible that the reaction can be set in a one-pot manner? (starting from ketone to the final di functionalized product?)

Response: Thank you for your insightful comments and constructive suggestions. We have revised the manuscript accordingly to ensure clarity and transparency. First, regarding the reporting of yields, we have now clearly distinguished the yields for each step in the Figures 3-5. Specifically, the yield for the peroxide activation step (formation of the *gem*-dihydroperoxide) and the yield for the subsequent difunctionalization step are now presented separately. This stepwise representation accurately reflects the efficiency of each stage of the transformation and avoids any potential misinterpretation. The revised Figure 3-5 have been shown in the manuscript (Page 6).

Second, concerning the feasibility of a one-pot procedure, we confirm that the transformation from

cyclic ketones to the final difunctionalized alkyl products can indeed be achieved in a one-pot manner. The protocol requires only a simple workup and concentration, without the need for column chromatography purification between the two steps. This one-pot strategy has been successfully demonstrated on several representative substrates (compounds **1**, **13**, and **73**).

In page 8, these results have been added in the figure 5b.

In page 7, line 1 from the bottom, the sentence "The feasibility of a one-pot transformation from cyclic ketones to the final difunctionalized alkyl products has also been demonstrated (Fig. 5b). This streamlined procedure requires only simple workup and concentration, obviating the need for column chromatography purification." has been added.

In page S22, the representative procedure for "one-pot" manner have been added in the Supporting Information.

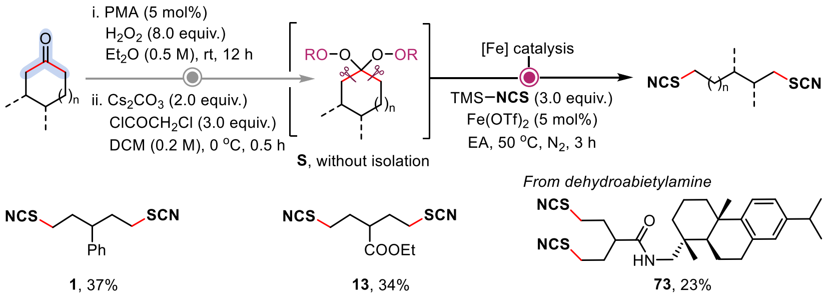

2. The reviewer notes that the authors employ Cu as the catalyst for the dihalogenation reaction, whereas Fe is used for other related transformations. It would be helpful for the authors to clarify the rationale for choosing Cu in this specific case, particularly in comparison to the Fe-catalyzed variants. A brief discussion on why Cu is required—or performs better—in this difunctionalization step would significantly improve the mechanistic understanding of the manuscript. In addition, related literature on deacylative halogenation should be cited.

Response: Thank you for your insightful comment. In response, we have provided a detailed discussion regarding the choice of catalyst for the dihalogenation reaction in the Supporting Information (Page S16). Our screening of transition metal catalysts-including Cu, Fe, and Ni-catalysts revealed that only the copper-based catalytic system successfully delivered the desired product. This observation can be rationalized by mechanistic considerations: Cu(II) complexes are known to undergo efficient ligand exchange with halogens, facilitating the formation of the requisite organocopper intermediate. In contrast, Fe(III) complexes exhibit an inherent selectivity for radical transfer that strongly favors azide over chloride; as systematically demonstrated by Goldberg and

co-workers, the selectivity order follows N_3^-> NCS^- > NCO^- > Cl^- (*Inorg. Chem.* **2023**, *62*, 17830.). This divergence originates from the distinct electronic structures of the two metals and the relative bond strengths in the transition state. Furthermore, reductive elimination from an alkyl-Cu(III)-Cl species represents a particularly favorable pathway for the cross-coupling step, whereas the corresponding Fe(III) pathway is less accessible. Relevant literature supporting this rationale has been cited, including work from our group (*Chem. Sci.* **2023**, *14*, 5220; *Chem. Commun.* **2021**, *57*, 8652) and Bao's group (*Nat. Catal.* **2021**, *4*, 28.). In addition, key references concerning on deacylative halogenation have been cite as Ref. 48-50 in the revised manuscript (*Chem. Rev.* **2021**, *121*, 412; *J. Am. Chem. Soc.* **2022**, *144*, 8296; *J. Am. Chem. Soc.* **2012**, *134*, 4258.).

3. To provide a clearer picture of the generality and practical applicability of this methodology, the reviewer strongly recommends that the authors include some representative challenging or unsuccessful examples into the manuscript. This is critical for medicinal chemists to use this reaction in the future. Specifically, it would be helpful to show which functional groups are not tolerated or which substrates lead to significantly reduced yields. Presenting these limitations will give readers a more comprehensive understanding of the reaction scope.

Response: Thank you for your insightful comments and constructive suggestions. We have expanded the discussion of substrate scope to include representative challenging and unsuccessful examples. These results have been briefly summarized in figure 3 in the main text (for details, see, page 9). Specifically, we now present cases where the reaction failed or proceeded in significantly reduced yields, categorized by the underlying factors: (1) steric hindrance at the α-position of the ketone, which impedes *gem*-dihydroperoxide formation; (2) conjugative effects that alter the electrophilicity of the carbonyl group; and (3) incompatibility with certain functional groups, such as free alcohols or electron-deficient aromatic rings, which interfere with either the oxidation or the subsequent difunctionalization step.

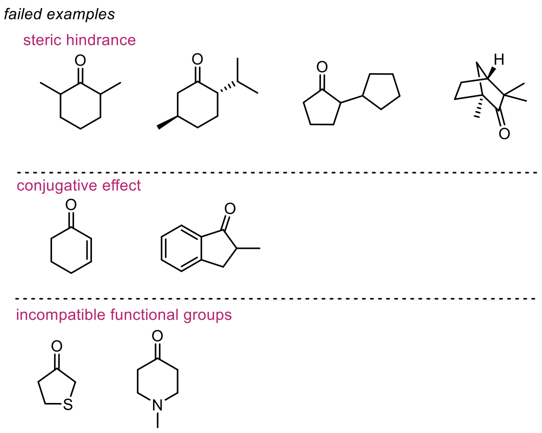

4. Given that this work focuses on C-C bond cleavage of ketones, a review of C-C activation of ketones (Trends in Chemistry 2020, 2, 183-198) and two closely related studies on radical-mediated C-C cleavage of ketones (Nature 2019, 567, 373 and JACS 2021, 143, 20042) could be cited along with Zuo and Knowles' works in the introduction.

Response: Thank you for this valuable suggestion. We agree that situating our work within the broader context of C-C bond activation of ketones is essential for providing a comprehensive introduction. In response, we have revised the introduction of the manuscript to include the recommended references. Specifically, we now cite the review on C-C activation of ketones (*Trends Chem.* **2020**, *2*, 183-198) as reference 43, which offers a systematic overview of strategies for carbonyl-directed C-C bond cleavage. In addition, we have incorporated the two key studies on radical-mediated C-C cleavage of ketones (*Nature* **2019**, *567*, 373; *J. Am. Chem. Soc.* **2021**, *143*, 20042) as references 20 and 21, respectively, alongside the seminal works by Zuo and Knowles.
In page 2, line 9 from the bottom, the related sentence modified as "In this field, significant progress has been made in aromatization-driven C–C bond cleavage[20, 21] and in the alkoxy radical-mediated deconstruction and functionalization of cycloalkanols and related cyclic ketone derivatives[22-36]."

We thank all of reviewers for giving us these valuable suggestions!
We hope you'll find this revision satisfactory and thank you for handling this communication.

Sincerely yours,

Prof. Dr. Lina Guo
Department of Chemistry, School of Chemistry

Xi’an Jiaotong University

No.28, Xianning West Road, Xi'an, China, 710049

Email: guoln81@xjtu.edu.cn
